# Curtachalasins Y1–Y13, Anti-Inflammatory Cytochalasans from the Soil Fungus *Xylaria* sp. Y01

**DOI:** 10.3390/ijms27146313

**Published:** 2026-07-15

**Authors:** Yi-Yun Yuan, Yang Xie, Xi Zhou, Liang Tu, Ying-Meng Leng, Qi-An Chen, Qing-Hui Xiao, Shao Liu, Wen-Xuan Wang, Jing Li

**Affiliations:** 1 Department of Pharmacy, National Clinical Research Center for Geriatric Disorders, Xiangya Hospital, Central South University, Changsha 410008, China; 2 Xiangya School of Pharmaceutical Sciences, Central South University, Changsha 410083, China

**Keywords:** cytochalasans, *Xylaria* sp., anti-inflammatory activity, structure elucidation

## Abstract

Twenty-seven cytochalasans, including thirteen previously undescribed analogs with a 5/6/6/6 tetracyclic skeleton (curtachalasins Y1–Y13), were isolated from the rice-based culture of a soil-derived fungus, *Xylaria* sp. Y01. Their structures were determined through comprehensive analysis of spectroscopic data and quantum chemical calculations. All isolates were evaluated for their inhibitory effects on nitric oxide (NO) production in lipopolysaccharide-stimulated RAW264.7 macrophages. Curtachalasin Y1 (**12**), 5,6-dihydro-7-oxo-18-desoxy-19,20-epoxycytochalasin C (**21**), and 7-oxo-19,20-epoxycytochalasin C (**22**) exhibited potent inhibition, with IC_50_ values of 55.4, 43.8, and 17.8 µM, respectively, compared to 14.9 μM for the positive control dexamethasone. Furthermore, these compounds suppressed lipopolysaccharide (LPS)-induced release of pro-inflammatory cytokines IL-6, MCP-1, and TNF-α, confirming their anti-inflammatory activity. Preliminary mechanistic investigation indicated that compound **22** exerts its anti-inflammatory effects in RAW264.7 cells by downregulating CXC motif chemokine ligand 10 (CXCL10) and upregulating suppressor of cytokine signaling 3 (SOCS3) expression. These results expand the structural diversity of cytochalasans from *Xylaria* species and provide a basis for further exploration of their anti-inflammatory potential.

## 1. Introduction

Fungi of the genus *Xylaria*, members of the family Xylariaceae within the order Xylariales, constitute a diverse and ecologically significant group [1]. With approximately 300 recognized species, this globally distributed genus spans temperate to tropical climates [2]. *Xylaria* species, primarily saprophytic or endophytic, represent a rich source of chemically diverse secondary metabolites. These compounds exhibit a broad spectrum of bioactivities, including anticancer, antimicrobial, cytotoxic, and anti-inflammatory properties [2].

Cytochalasans are a prominent class of fungal metabolites derived from polyketide-nonribosomal peptide synthetase hybrids and possess diverse biological functions [3]. Since the initial discovery of cytochalasins A and B in 1966 from *Phoma* sp. S 298 and *Helminthosporium dematioideum*, respectively, over 500 cytochalasans have been reported [3,4]. These compounds are predominantly isolated from fungi belonging to the genera *Xylaria*, *Chaetomium*, *Aspergillus*, *Penicillium* and *Zygosporium* [3]. Recent decades have seen significant progress not only in expanding the known chemical diversity of this class but also in elucidating their biological activities, molecular targets, and mechanisms of action [5].

Soil-derived fungi are renowned for their capacity to produce secondary metabolites with unique chemical diversity, including pyridone derivatives [6], cytochalasins [7], steroids [8], and sesquiterpenes [9]. Among these, the curtachalasins—characterized by a distinctive 5/6/6/6 tetracyclic skeleton—were first reported from the fungus *Xylaria* curta E10. To date, a total of 18 curtachalasin analogs have been identified [6,10,11], and their various activities, such as cytotoxicity and antibacterial, antifungal, and immunosuppressive effects, have been investigated.

**Figure 1:**
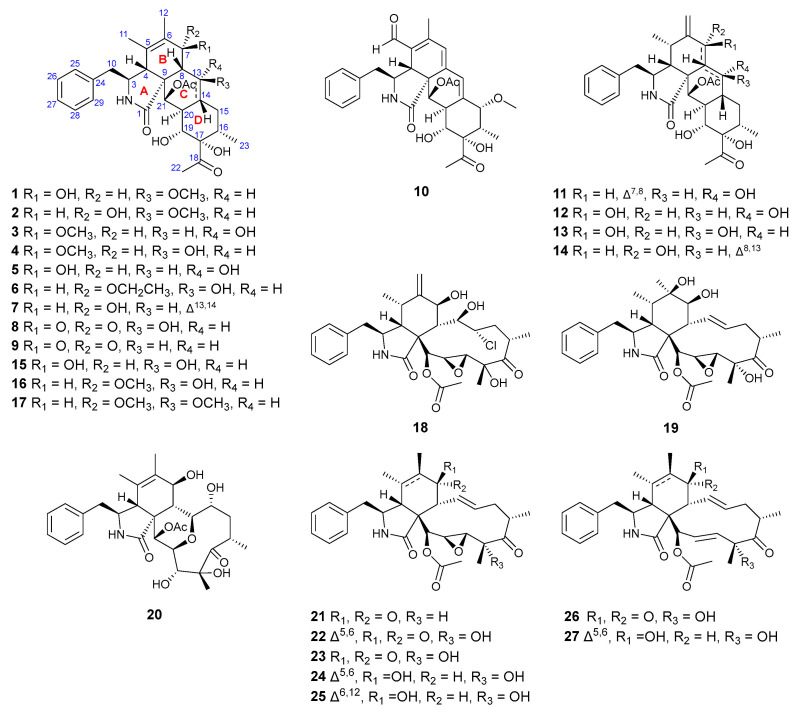
Chemical structures of compounds **1**–**27**.

During a search for novel anti-inflammatory cytochalasans, a fungal strain isolated from a soil sample collected in Kunming, China, and identified as *Xylaria* sp. Y01 was investigated. This study led to the isolation of twenty-seven cytochalasans from its rice-based culture. These include thirteen new curtachalasin-type compounds, designated curtachalasins Y1–Y13 (**1**–**13**), alongside fourteen known analogs: curtachalasin J (**14**) [10], curtachalasin X4 (**15**) [11], curtachalasin X5 (**16**) [11], curtachalasin G (**17**) [10], 13-hydroxy-14-chloro-13,14-dihydro-19,20-epoxycytochalasin D (**18**) [12], 19-epi-cytochalasin P1 (**19**) [13], cytochalasin C1 (**20**) [14], 5,6-dihydro-7-oxo-18-desoxy-19,20-epoxycytochalasin C (**21**) [12], 7-oxo-19,20-epoxycytochalasin C (**22**) [12], 5,6-dihydro-7-oxo-19,20-epoxycytochalasin C (**23**) [13], 19,20-epoxycytochalasin C (**24**) [15], 19,20-epoxycytochalasin D (**25**) [15], 6,7-dihydro-7-oxo-cytochalasin C (**26**) [16], and cytochalasin C (**27**) (Figure 1) [17]. All isolates were tested for their ability to inhibit nitric oxide (NO) production in lipopolysaccharide (LPS)-stimulated RAW264.7 macrophages. Compounds **12**, **21**, and **22** showed potent inhibition, with IC_50_ values of 55.4, 43.8, and 17.8 μM, respectively (positive control dexamethasone: 14.9 μM). These three compounds also reduced LPS-induced secretion of the pro-inflammatory cytokines IL-6, MCP-1, and TNF-α. Preliminary mechanistic studies revealed that compound **22** exerts its anti-inflammatory effect in RAW264.7 cells through downregulation of CXC motif chemokine ligand 10 (CXCL10) and upregulation of suppressor of cytokine signaling 3 (SOCS3) expression. This report describes their isolation, structural elucidation, and evaluation of anti-inflammatory properties.

## 2. Results and Discussion

**Figure 2:**
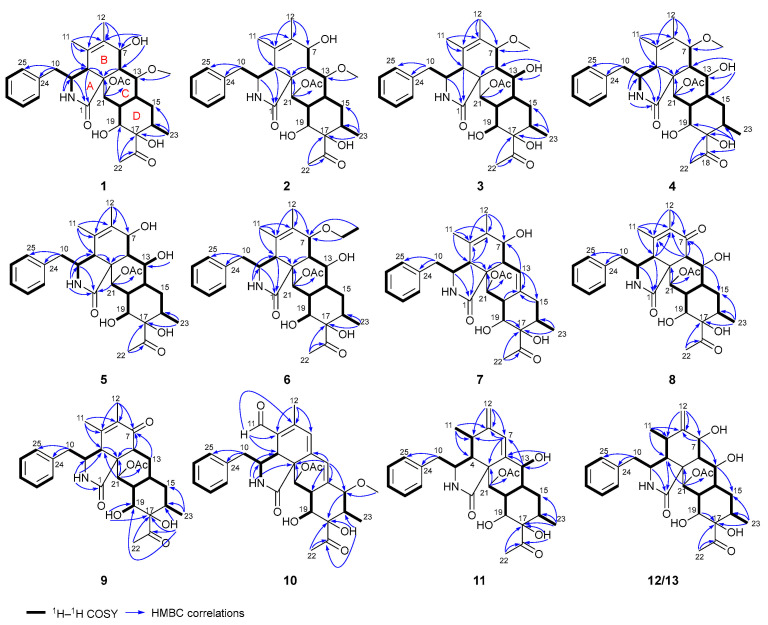
Key ^1^H–^1^H COSY and HMBC correlations of compounds **1**–**13**.

Compound **1** was obtained as a white powder. The molecular formula was determined as C_31_H_41_NO_8_ by HRESIMS (*m*/*z* 578.2724 [M + Na]^+^ Δ −0.2 ppm), corresponding to 12 degrees of unsaturation. Its UV spectrum exhibited absorptions at 210 and 260 nm. The IR spectrum displayed characteristic bands for an ester group (1733 cm^−1^) and hydroxy groups (3346 cm^−1^). The ^1^H and ^13^C NMR data of **1** (Table 1) displayed signals characteristic of a cytochalasan skeleton [7], including a monosubstituted phenyl group at *δ*_H_ 7.13 (2H, d, *J* = 7.7 Hz), 7.30 (2H, t, *J* = 7.7 Hz), and 7.23 (1H, t, *J* = 7.7 Hz); one methyl doublet at *δ*_H_ 0.79 (3H, d, *J* = 6.5 Hz), two methylene groups at *δ*_H_ 2.86 (1H, dd, *J* = 13.4, 6.8 Hz) and 2.94 (1H, dd, *J* = 13.4, 8.4 Hz), *δ*_H_ 1.85 (1H, overlapped), and 1.45 (1H, ddd, *J* = 12.5, 12.1, 12.1 Hz), a ketone carbon (*δ*_C_ 212.4), one amide carbonyl (*δ*_C_ 175.2), an ester carbonyl (*δ*_C_ 172.1), one tetrasubstituted double bond (*δ*_C_ 125.0 and 133.0), five methyl singlets (*δ*_C_ 14.2, 17.4, 21.2, 25.3, 59.9), two sp^3^-hybridized quaternary carbons (*δ*_C_ 50.4 and 83.6), an N-methyl group (*δ*_C_ 59.4), four oxygenated methines (*δ*_C_ 70.7, 71.5, 72.2, 84.5), and five high-field methines (*δ*_C_ 36.1, 39.5, 41.6, 43.2, 48.6). Analysis of the ^1^H–^1^H COSY spectrum revealed three spin-spin coupling systems, namely H_2_-10/H-3/H-4, H-25/H-26/H-27/H-28/H-29, and H-7/H-8/H-13/H-14(H_2_-15/H-16/H_3_-23)/H-20/H-21(H-19) (Figure 2). Key HMBC correlations (Figure 2) between NH and C-3/C-4/C-9, H-3 and C-1, H-4 and C-1/C-9, H-8 and C-9, H_3_-11 and C-4/C-5/C-6, as well as H_3_-12 and C-5/C-6/C-7 established the connectivity of rings A and B. Further HMBC correlations between 7-OH and C-6/C-7/C-8, H-21 and C-9, H_3_-23 and C-15/C-16/C-17, H_3_-22 and C-17/C-18/C-19, together with COSY data, completed the assembly of rings B, C, and D. The attachment of the phenyl group at C-10 was indicated by HMBC correlations from H_2_-10 to C-24 (*δ*_C_ 137.7) and to C-25/C-29 (*δ*_C_ 129.2). Thus, the planar structure of **1** was determined as shown in Figure 2.

**Figure 3:**
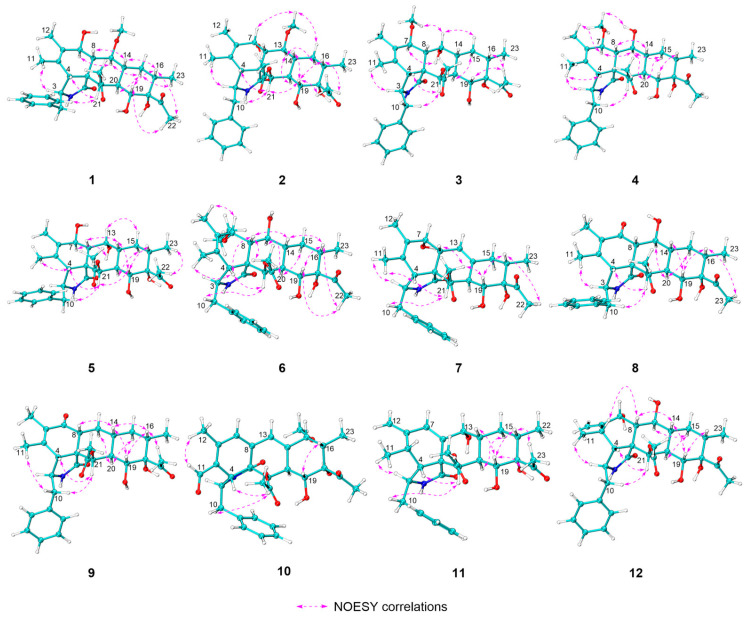
Key NOESY correlations of compounds **1**–**12**, depicted with their lowest-energy conformations (coordinates are provided in Tables S20–S32).

**Figure 4:**
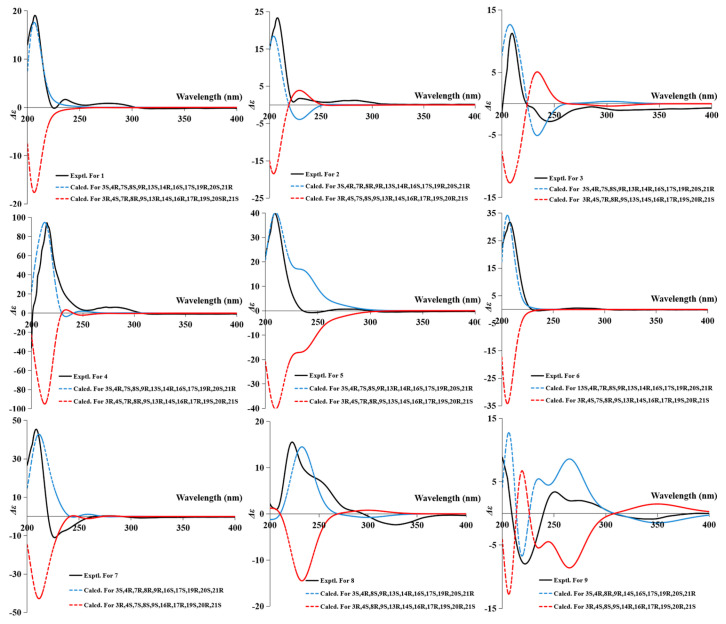

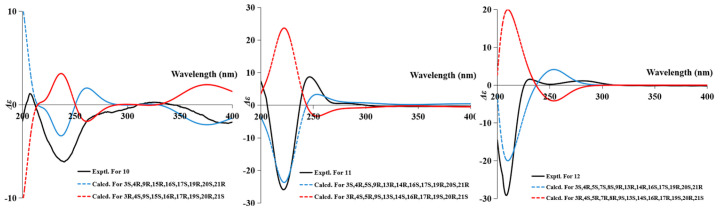
Calculated and experimental ECD spectra of compounds **1**–**9**.

Stereochemical analysis relied on the coupling constants of the cyclohexane rings (B, C, and D). The observed large vicinal couplings for H-7 (9.7 Hz), H-8 (10.0, 9.7 Hz), H-13 (10.4, 10.0 Hz), H-19 (10.5 Hz), and H-20 (12.3, 10.5, 2.1 Hz) indicated axial orientations for these protons, whereas the small coupling of H-21 (2.1 Hz) suggested an equatorial orientation. Key NOESY correlations defined the relative configuration. Correlations between H-3/H_3_-11, H-4/H-21, and H-21/H_2_-10 indicated that H-3 and the ring B are on one face of ring A, whereas H-4, H_2_-10, and H-21 lie on the opposite face. Additional NOESY cross peaks between H-8/H-14, H-14/H-16, H-16/H-19, and H-19/H_3_-22 defined one spatial cluster, while correlations between H-7/H-13 and H-13/H_ax_-15/H-20 defined another. Because H-7 and H-8 are axially oriented but point in opposite directions, these two clusters reside on opposite faces of the molecule. The resulting relative configuration was depicted in Figure 3. The absolute configuration of **1** was determined by ECD calculation (Figure 4). The calculated ECD curve for the isomer 3*S*,4*R*,7*S*,8*S*,9*R*,13*S*,14*R*,16*S*,17*S*,19*R*,20*S*,21*R-***1** at the ωB97X-D/TZVP level (IEFPCM, acetonitrile) showed excellent agreement with the measured data. Therefore, the structure of **1** was fully assigned and named curtachalasin Y1.

HRESIMS analysis of compound **2** gave an identical molecular formula of C_31_H_41_NO_8_ (*m*/*z* 1133.5575 [2M + Na]^+^ Δ 0.0 ppm) to that of **1**. The ^1^H and ^13^C NMR data of **2** closely resembled those of **1**. Analysis of 2D NMR data confirmed that **1** and **2** shared the same planar structure (Figure 2). Notably, the ^13^C NMR chemical shifts of **2** differed by more than 2 ppm from those of **1** at C-1, C-5, C-7, C-12, and C-13, implying a stereochemical difference in rings B and C. The large coupling constants of H-13 (10.3, 10.3 Hz) indicated its axial orientation. Key NOESY cross peaks between H-7/13-OCH_3_/H_eq_-15 confirmed that these protons are on the same face of the molecule. To verify the relative configuration assignment, GFN2NMR calculations were performed [18]. Comparison of the experimental NMR data for **1** and **2** with their respective GFN2NMR-calculated values supported the stereochemical deductions (Tables S1 and S2). The calculated ECD curve for 3*S*,4*R*,7*R*,8*R*,9*R*,13*S*,14*R*,16*S*,17*S*,19*R*,20*S*,21*R-***2** at the ωB97X-D/TZVP level (IEFPCM, MeOH) showed excellent agreement with the measured data. Therefore, the structure was fully assigned and named curtachalasin Y2.

Compound **3** was obtained as a white powder with the molecular formula C_31_H_41_NO_8_ determined by HRESIMS (*m*/*z* 556.2914 [M + H]^+^ Δ +1.6 ppm), identical to those of **1** and **2**. The ^1^H and ^13^C NMR spectra also showed similar signals, except that the ^13^C NMR chemical shifts of **3** at C-7 and C-13 were observed at *δ*_C_ 76.5 and 66.7, respectively (*δ*_C_ 70.7 and 84.5 for **1**, *δ*_C_ 67.8 and 78.8 for **2**). These significant discrepancies imply a structural difference at these positions. The HMBC correlation (Figure 2) between 7-OCH_3_ and C-7 indicated that the methoxy group was attached to C-7. ^1^H–^1^H COSY correlations and coupling constants indicated that the hydroxyl group at *δ*_H_ 5.93 (1H, d, *J* = 11.5 Hz) is attached to C-13. The remaining 2D NMR connections were consistent with those of **1**. Key NOESY correlations between H-8/H-14/H-16/H-19/H_3_-22 and 7-OCH_3_/H-13/H_eq_-15 established the relative configuration as shown in Figure 2. The calculated ECD curve at the M062X/6-311+G* (IEFPCM, acetonitrile) level for 3*S*,4*R*,7*S*,8*S*,9*R*,13*R*,14*R*,16*S*,17*S*,19*R*,20*S*,21*R*-**3** matched the experimental ECD well. Therefore, the structure of compound **3** was fully assigned and named curtachalasin Y3.

Compound **4** was obtained as a white powder with the molecular formula C_31_H_41_NO_8_ determined by HRESIMS (*m*/*z* 1133.5592 [2M + Na]^+^ Δ +1.5 ppm), identical to that of compounds **1**–**3**. The ^1^H and ^13^C NMR, HSQC, ^1^H–^1^H COSY, and HMBC spectra confirmed that **3** and **4** shared the same planar structures (Figure 2). However, significant differences in chemical shifts (>5 ppm) were observed at C-7 and C-13 (Table 2). The coupling constants of H-7 (9.8 Hz) and H-13 (9.5, 9.5 Hz) indicated that H-7 and H-13 were axially oriented. In contrast, the H-13 proton in 3 occupied an equatorial position. The altered orientation of the 13-OH group likely affected the chemical shifts in C-7 and C-13 (Figure 3). This speculation was supported by GFN2NMR ^13^C NMR calculations for **3** and **4** (Table S6). Moreover, key NOESY cross peaks between H-7/H-13/H_ax_-15 and 7-OCH_3_/13-OH confirmed that the difference between **3** and **4** was the configuration at position C-13 (Figure 3). To determine the absolute configuration of **4**, ECD predictions were performed. The calculated ECD curve at the M062X/6-311+G* (IEFPCM, acetonitrile) level for 3*S*,4*R*,7*S*,8*S*,9*R*,13*S*,14*R*,16*S*,17*S*,19*R*,20*S*,21*R*-**4** matched the experimental ECD well. Therefore, the structure of **4** was fully assigned and named curtachalasin Y4.

Compound **5** was obtained as a white powder with the molecular formula C_30_H_39_NO_8_ established by HRESIMS (*m*/*z* 542.2752 [M + H]^+^ Δ +0.6 ppm). Analysis of the ^1^H and ^13^C NMR data (Table 3) revealed that the structure of **5** closely resembled that of **3**, with the key difference being the replacement of the methoxy group at C-7 in **3** with a hydroxy group. The coupling constants of H-7 (*J* = 10.7 Hz), H-8 (*J* = 10.7, 3.3 Hz) supported axial configurations for both protons. Moreover, NOESY correlations of H-7/13-OH indicated that they were on the same side of the ring system (Figure 3). Compared to the known compound **14**, **5** is presumed to be its isomerized product at C-13. The ^13^C NMR chemical shifts of **14** and **5** were compared in the same solvent (CDCl_3_), and clear differences were observed near the isomerization position (Table S33). GFN2NMR was also used to calculate their ^13^C NMR chemical shifts, confirming the structural assignments. The absolute configuration of **5** was further determined to be 3*S*,4*R*,7*S*,8*S*,9*R*,13*R*,14*R*,16*S*,17*S*,19*R*,20*S*,21*R* by ECD calculation at the B3LYP/TZVP (IEFPCM, MeOH) level of theory (Figure 4).

Compound **6** was obtained as a white powder with the molecular formula C_32_H_43_NO_8_ determined by HRESIMS (*m*/*z* 592.2907 [M + Na]^+^ Δ +4.4 ppm). Compared to **4**, the ^1^H NMR data of **6** revealed the presence of an additional ethyl group, characterized by a methyl group at *δ*_H_ 1.09 (3H, dd, *J* = 7.0, 7.0 Hz) and a methylene group at *δ*_H_ 3.53 (1H, dq, *J* = 9.2, 7.0 Hz) and 3.64 (1H, dq, *J* = 9.2, 7.0 Hz). Additionally, the chemical shift in C-7 was significantly different from that of **4**, with a downfield shift of 8.9 ppm. HMBC correlations between the ethyl group and C-7 indicated that the ethoxy group is attached to C-7 (Figure 2). The observed NOE correlations between H-4 and H-8 confirmed their co-facial orientation on ring B, with both protons adopting axial positions (Figure 3). The coupling constant between H-7 and H-8 (2.1 Hz) indicated that H-7 is equatorially oriented. The remainder of the planar structure and the relative configuration of **6** were determined to be identical to those of **4** (Figure 2). Finally, the absolute configuration of **6** was determined to be 3*S*,4*R*,7*R*,8*S*,9*R*,13*S*,14*R*,16*S*,17*S*,19*R*,20*S*,21*R* by ECD calculation at the ωB97X-D/TZVP (IEFPCM, MeOH) level of theory (Figure 4).

Compound **7** was obtained as a white powder with the molecular formula C_30_H_43_NO_8_ determined by HRESIMS (*m*/*z* 1069.5055 [2M + Na]^+^ Δ +2.1 ppm). Compared to **2**, the ^1^H and ^13^C NMR data of **7** indicated that **7** lacks a methoxy group and two methine groups, and instead possesses an additional double bond signal at *δ*_C_ 120.6 and 134.5. 2D NMR analysis confirmed **7** as a 13,14-dehydrogenated product derived from **2**. The absolute configuration of **7** was further determined by ECD calculation at the B3LYP/TZVP (IEFPCM, acetonitrile) level of theory (Figure 4). Therefore, the structure of **7** was determined.

Compound **8** was obtained as a white powder with the molecular formula C_30_H_37_NO_8_ determined by HRESIMS (*m*/*z* 1101.4925 [2M + Na]^+^, Δ −0.5 ppm). Compared to **4**, the ^1^H and ^13^C NMR data of **8** indicated that it lacks a methoxy group and contains an additional carbonyl group, observed at *δ*_C_ 199.3. HMBC correlations between H_3_-12/H-8/H-13 and C-7 indicated the position of the carbonyl group. The 2D NMR data proved that **8** is a 7-oxidized product derived from **4** (Figure 3). The absolute configuration of **8** was confirmed by ECD calculation at the B3LYP/TZVP (IEFPCM, MeOH) level of theory (Figure 4).

Compound **9** was obtained as a white powder with the molecular formula C_30_H_37_NO_7_ determined by HRESIMS (*m*/*z* 1069.5045 [2M + Na]^+^, Δ +1.1 ppm). Compared to **8**, it contains one fewer oxygen atom. Comparison of the 1D and 2D NMR data of **9** with those of **8** indicated that **9** possesses an additional methylene at C-13 (*δ*_C_ 26.6) (Figure 3), indicating that **9** is a deoxygenized product at C-13 from **8**. The absolute configuration of **9** was determined to be 3*S*,4*R*,9*R*,15*R*,16*S*,17*S*,19*R*,20*S*,21*R* by ECD calculation at the M062X/6-311+G* (IEFPCM, acetonitrile) level of theory (Figure 4). Therefore, the structure of **9** was determined.

Compound **10** was obtained as a white powder. Its molecular formula was determined to be C_31_H_35_NO_8_ by HRESIMS (*m*/*z* 572.2257 [M + Na]^+^, Δ +0.3 ppm), indicating an index of hydrogen deficiency of 15. The UV spectrum of **10** in MeOH exhibited absorption maxima at 230 and 365 nm, suggesting the presence of multiple conjugated structures. The ^1^H, ^13^C NMR, DEPT, and HSQC spectra revealed characteristic features (Table 4 and Table 5), including a monosubstituted benzene ring with signals at *δ*_H_ 7.22 (2H, overlapped), 7.30 (2H, t, *J* = 7.6 Hz), and 7.23 (1H, overlapped), an aldehyde group at *δ*_H_ 10.15 (1H, s), a methoxy group at *δ*_H_ 3.40 (3H, s), one methyl doublet at *δ*_H_ 1.06 (3H, d, *J* = 7.0 Hz), three methyl groups attached to double bonds at *δ*_H_ 2.10 (3H, s), 2.30 (3H, d, *J* = 1.7 Hz), and 2.31 (3H, s), an amide group at *δ*_C_ 173.2, one ester group at *δ*_C_ 171.6, three double bonds at *δ*_C_ 128.1, 128.7, 131.2, 137.4, 137.4, and 147.5, and a methylene group at *δ*_C_ 44.2. The ^1^H–^1^H COSY and HMBC signals indicated a 5/6/6/6 fused ring system scaffold, as observed for **1**–**9** (Figure 2). The HMBC corrections of H-7 to C-5/C-6/C-9, H-13 to C-8/C-9/C-14/C-15/C-20, H-21 to C-9, and H_3_-12 to C-5/C-6/C-7 validated the planar structure of rings B and C. The aldehyde proton signal was shifted upfield, indicating its conjugation with double bonds. The HMBC correlation between the aldehyde proton and C-4/C-5/C-6 established the position of aldehyde group. The chemical shift in C-15 and the HMBC correlations of the methoxy group to C-15 suggested the position of the methoxy group (Figure 2). The remainder of the planar structure was consistent with that of **1**–**9** (Figure 2). Additionally, NOESY correlations between H-16 and H-19 indicated that they are located on the same side of ring D and both occupy axial positions (Figure 3). The coupling constants between H-15 and H-16 (*J* = 2.8 Hz) suggested that H-15 is in an equatorial position. The NOESY correlations between H-4 and H_2_-10/H-21 indicated that the benzyl group and H-4 are both located on the same side of ring A. Based on the relative configuration of **10** as shown in Figure 3, the absolute configuration was determined to be 3*S*,4*R*,9*R*,15*R*,16*S*,17*S*,19*R*,20*S*,21*R* by ECD calculation on the M062X/6-311+G* (IEFPCM, MeOH) level of theory (Figure 4).

Compound **11** was obtained as a white powder with the molecular formula of C_30_H_37_NO_7_, determined by HRESIMS (*m*/*z* 546.2460 [M + Na]^+^, Δ −0.6 ppm). Its ^1^H and ^13^C NMR spectra indicated the same basic scaffold as **1**–**10** (Figure 2). Compound **11** possesses a sp^2^ methylene group, with signals at *δ*_H_ 5.10 (1H, br s), 5.15 (1H, br s), and *δ*_C_ 113.4. The HMBC correlations between H_2_-12 and C-5/C-6/C-7, and between H-7 and C-5/C-6/C-9, as well as the ^1^H–^1^H COSY correlations of H_2_-10/H-3/H-4/H-5/H_3_-11 revealed the structure of rings A and B (Figure 2). The relative configuration of rings C and D, as supported by 2D NMR data, was consistent with that of **3** (Figure 2). NOESY correlations between H-3 and H_3_-11 indicated that they are located on the same side of ring B. The observed NOESY correlations between H-14 and H-19, together with the coupling constants of H-19, indicated that both protons adopt an axial orientation on the same side of ring D (Figure 3). Moreover, the coupling constants between H-13 and H-14 suggested that H-13 is in an equatorial position. The relative configuration of **11** was fully assigned (Figure 3). The absolute configuration of **11** was determined to be 3*S*,4*R*,5*S*,9*R*,13*R*,14*R*,16*S*,17*S*,19*R*,20*S*,21*R* by ECD calculation at the M062X/6-311+G* (IEFPCM, acetonitrile) level of theory (Figure 4).

Compound **12** was obtained as a white powder with the molecular formula C_30_H_39_NO_8_, determined by HRESIMS (*m*/*z* 542.2748 [M + H]^+^, Δ −0.2 ppm), containing one more oxygen atom than **11**. Compounds **12** and **11** share very similar structures, except for the absence of one double bond and the presence of two additional sp^3^ methine groups (*δ*_C_ 43.1, 69.4) (Table 6), as evidenced by a comparison of their ^1^H and ^13^C NMR data. 2D NMR confirmed that **12** was substituted with a hydroxyl group at C-7 (Figure 2). The coupling constants of H-7 (10.5 Hz) suggested that H-7 and H-8 are both in axial positions. The remaining relative configurations were consistent with those of **11**, as supported by coupling constants and NOESY correlations (Figure 3). The absolute configuration of **12** was confirmed by ECD calculation at the B3LYP/TZVP (IEFPCM, acetonitrile) level of theory (Figure 4).

**Figure 5:**
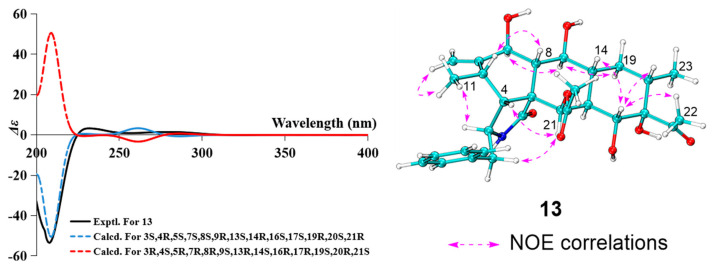
Key NOE correlations, the experimental and calculated ECD curves of **13** and its enantiomers.

Compound **13** was obtained as a white powder with the molecular formula C_30_H_39_NO_8_, determined by HRESIMS (*m*/*z* 542.2739 [M + H]^+^, Δ −1.8 ppm). Compounds **13** and **12** possess the same planar structures as revealed by NMR data. However, they are two different compounds with different retention times in the HPLC chromatogram (Figure S152). Their main difference lies in the methyl carbon at C-13 (*δ*_C_ 73.8 for **13** and 68.8 for **12**), suggesting epimerization at this position. The coupling constants of H-13 (*J* = 10.0, 10.0 Hz) suggested that H-8, H-13, and H-14 are all in axial positions (Table 5), which was confirmed by the NOESY correlations observed between H-7 and H-13. The remaining relative configurations were consistent with those of **12**, as supported by NOESY correlations (Figure 2). The absolute configuration of **13** was confirmed by ECD calculation at the B3LYP/TZVP (IEFPCM, MeOH) level of theory (Figure 5). Moreover, the ^13^C NMR chemical shift calculations of compounds **1**–**13** were performed using GFN2NMR (Figures S2–S19). As a result, all of the calculated data fitted well with the corresponding experimental data, indicating the high confidence of their structure assignment.

To determine the anti-inflammatory activity of the cytochalasins, compounds available in sufficient quantities (**1**, **5**, **7**, **8**, **12**, **13**–**16**, and **18**–**27**) were evaluated for their inhibitory effects on NO production in the LPS-induced RAW264.7 cell model, using dexamethasone as a positive control. First, CCK-8 analysis was used to assess cytotoxicity against RAW264.7 cells. Sixteen cytochalasins (**1**, **5**, **7**, **8**, **12**, **13**–**16**, **19**–**23**, **26**, and **27**) exhibited >90% cell viability after treatment at 30 μM and were selected for further evaluation. The inhibitory activity on NO production was assessed by measuring nitrite/nitrate levels in the supernatant of LPS-induced RAW264.7 cells. Compared with the vehicle-treated LPS group, treatment with compounds **12**, **21** and **22** significantly reduced NO levels, with IC_50_ values of 55.4, 43.8, and 17.8 μM, respectively. Notably, the effect of compound **22** approached that of dexamethasone (IC_50_: 14.9 μM). The remaining compounds showed IC_50_ values exceeding 60 μM (Table 7).

**Figure 6:**
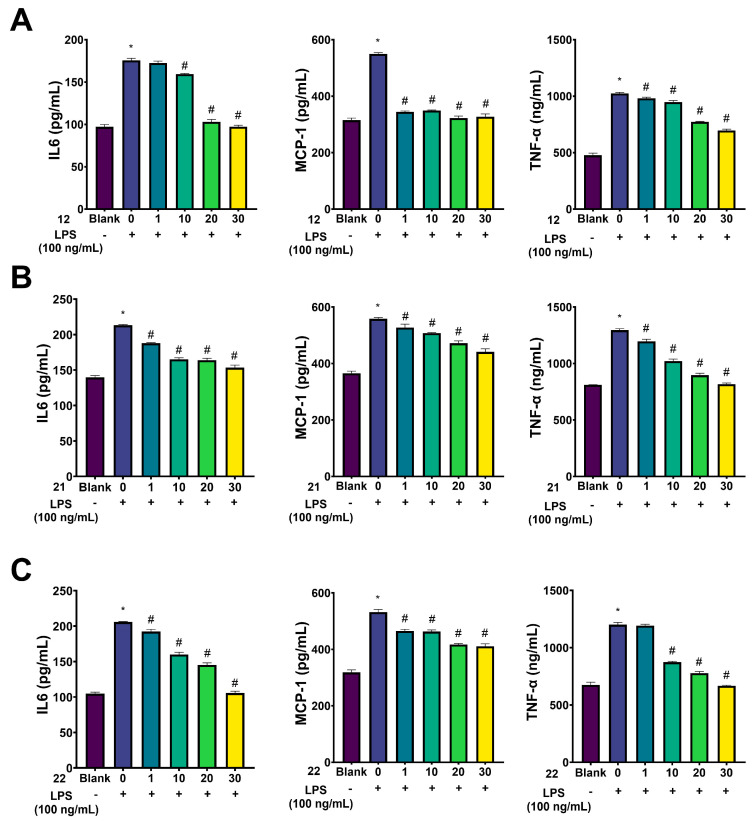
Levels of TNF-α, MCP-1, and IL-6 were measured by ELISA in cells treated with compounds **12** (**A**), **21** (**B**), and **22** (**C**), using vehicle-treated cells as the control group. * *p* < 0.05 vs. blank group, ^#^ *p* < 0.05 vs. control group.

Previous studies have shown that RAW264.7 cells release large amounts of pro-inflammatory cytokines, including IL-6, MCP-1, and TNF-α, in response to LPS-mediated inflammatory responses. Therefore, whether these compounds exert an inhibitory effect on the release of pro-inflammatory cytokines was investigated. The ELISA results indicated that IL-6, MCP-1, and TNF-α levels were significantly decreased by treatment with compounds **12**, **21**, and **22** (Figure 6), confirming their anti-inflammatory effects.

**Figure 7:**
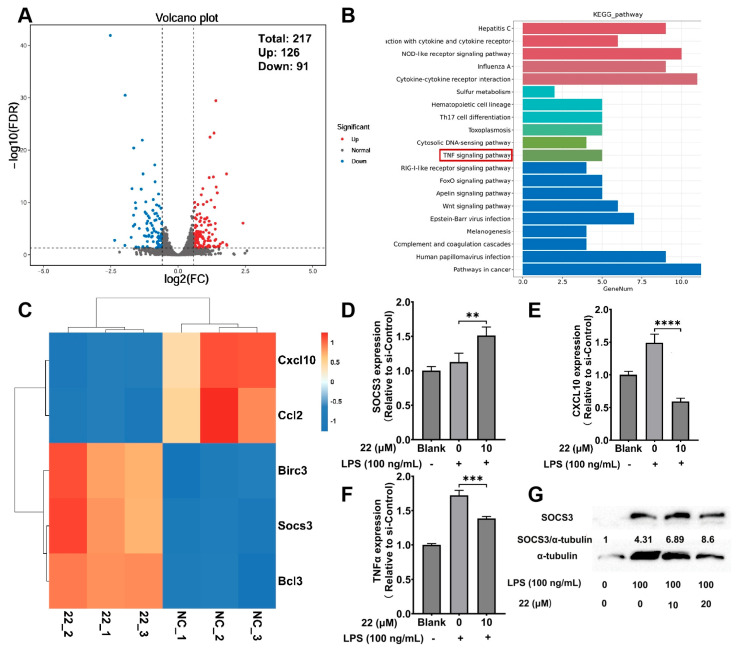
Compound **22** exhibited anti-inflammatory effects in RAW264.7 cells by upregulating SOCS3 expression. (**A**) Volcano plot of DEGs in RAW264.7 cells treated with compound **22** versus control cells. The *x*-axis represents log_2_ (fold change), and the *y*-axis represents −Log_10_ (false discovery rate, FDR). Blue dots indicate downregulated genes; red dots indicate upregulated genes. (**B**) KEGG pathway enrichment analysis of DEGs. Colors indicate the regulation status of the pathways: red represents up-regulation, blue represents down-regulation, and cyan represents normal/non-significant changes. The red box highlights the “TNF signaling pathway”. (**C**) Heatmap of DEGs in RAW264.7 cells treated with compound **22** versus control cells. (**D**–**F**) RT–qPCR analysis of SOCS3, CXCL10, and TNF-α mRNA levels in RAW264.7 cells following treatment with compound **22**. (**G**) Western blot analysis of SOCS3 protein levels in RAW264.7 cells after treatment with compound **22**. ** *p* < 0.01, *** *p* < 0.001, and **** *p* < 0.0001 vs. control group.

RNA-sequencing-based transcriptome analysis was performed on RAW264.7 cells treated with compound **22** after LPS induction to preliminarily explore the potential mechanism. This high-throughput method enabled the identification of genes and pathways altered in response to **22** treatments. A total of 217 DEGs were identified between **22**-treated and untreated RAW264.7 cells, as shown in the volcano plot (Figure 7A), with 126 genes upregulated and 91 downregulated. KEGG pathway enrichment analysis indicated that the DEGs identified in RAW264.7 cells treated with **22** (compared to untreated cells) were related to the TNF signaling pathway, which is closely involved in inflammatory responses. DEGs such as CXCL10 and C-C motif ligand 2 (CCL2) were significantly downregulated, whereas SOCS3 was significantly upregulated by treatment with **22** (Figure 7B,C). Further mRNA expression analysis showed that treatment with **22** increased SOCS3 mRNA expression in RAW264.7 cells compared to controls (Figure 7D, *p* < 0.01). Moreover, CXCL10 and TNF-α levels in RAW264.7 cells were reduced in a dose-dependent manner following **22** treatment (Figure 7E,F, *p* < 0.001). To further validate these results, Western blotting was performed, revealing that treatment with **22** increased SOCS3 protein expression in a dose-dependent manner compared to LPS-induced controls (Figure 7G). Previous research has indicated that CXCL10 plays a key role as an inflammatory mediator [19,20], whereas SOCS3 serves as an anti-inflammatory factor and negatively regulates TNF-α activation [21]. Therefore, compound **22** likely inhibits the TNF signaling pathway and reduces inflammatory responses in RAW264.7 cells through a mechanism that involves CXCL10 and SOCS3.

Due to the limited initial amount of compound **22** (approximately 2 mg), the remaining material is no longer sufficient to support further experiments after several rounds of bioactivity screening and mechanistic studies. An additional sample will be obtained through semi-synthesis from other compounds (e.g., **24**) to meet the requirements of subsequent in-depth research.

In summary, twenty-seven cytochalasins (**1**–**27**) from a soil fungus *Xylaria* sp. Y01 are reported, including thirteen previously undescribed ones (**1**–**13**). Compounds **1**–**17** possess a 5/6/6/6 tetracyclic skeleton. The structures of the new compounds were elucidated by extensive spectroscopic methods and quantum chemical calculations. The inhibitory activity of all isolates on LPS-stimulated NO production in mouse RAW264.7 macrophages was evaluated. The results indicated that compound **22** exhibited anti-inflammatory effects in RAW264.7 cells by upregulating SOCS3 expression. Therefore, compound **22** represents a potential candidate for the research and development of treatments for inflammatory diseases. Structure–activity relationship analysis correlates the superior potency of compound **22** with the C-5/C-6 double bond and C-7 ketone carbonyl group, as these moieties are either absent or modified in less active analogs. This finding reinforces the importance of these structural elements for bioactivity and guides future optimization of cytochalasan derivatives. Although the present work emphasizes anti-inflammatory activity, additional biological profiling, including cytotoxicity and immunosuppressive assays, is necessary. Further studies addressing these aspects, particularly for compound **22**, are warranted.

## 3. Materials and Methods

### 3.1. General Experiment Procedures

UV spectra were recorded on a Cary 300 spectrometer (Agilent Technologies, Santa Clara, CA, USA). Experimental ECD data were collected using a Chirascan™-plus CD instrument (Applied Photophysics, London, UK). Optical rotations were measured on a Rudolph Autopol IV automatic polarimeter (Rudolph Research Analytical, Hackettstown, NJ, USA), and IR spectra were acquired on a Shimadzu FT-IR spectrometer (Shimadzu Corporation, Kyoto, Japan) with KBr pellets. HRESIMS spectra were recorded on an Agilent 6500 series Q-TOF mass spectrometer (Agilent Technologies, Singapore). NMR experiments (^1^H at 600 MHz and ^13^C at 150 MHz) were performed on Bruker spectrometers (Bruker BioSpin, Rheinstetten, Germany). For chromatographic separation, D101 macroporous adsorption resin (Tianjin Haoju, Tianjin, China) and silica gel (200-300 and 300-400 mesh; Qingdao Marine, Qingdao, China) were used for column chromatography. Preparative HPLC was performed on an E-Classical P3500 system (Dalian Elite Analytical Instruments Co., Ltd., Dalian, China) equipped with a DAD detector, using SinoChrom ODS-AP or Supersil C8 columns (5 μm, 10 × 250 mm, Dalian Elite Analytical Instruments Co., Ltd., Dalian, China). TLC was run on precoated silica gel GF254 plates (Qingdao Marine Chemical, Qingdao, China), and spots were visualized under UV light (254 or 356 nm) or by spraying with 10% H_2_SO_4_ in EtOH followed by heating. The purity of each compound tested in the bioassays was verified to be higher than 96% by HPLC analysis.

### 3.2. Fungal Material

The fungal strain *Xylaria* sp. Y01 was isolated from a soil sample collected from Kunming, Yunnan Province, China, in 2022. Within 24 h of collection, a nutrient-limited medium (1 g/L dextrose, 15 g/L agar) containing 50 mg/L streptomycin was inoculated with 100 µL of a suspension prepared from 1 g of soil in 250 mL of sterile water containing 50 mg/L streptomycin. The mixture was incubated at 25 °C for 7 days. Pure fungal colonies were selected and cultured on potato dextrose agar (PDA). The methods for sample processing and fungal species identification followed the procedures outlined in a previous publication [18]. The fungal specimen is deposited at the Xiangya School of Pharmaceutical Sciences, Central South University, Changsha, China.

### 3.3. Fermentation and Isolation

The hyphae of *Xylaria* sp. Y01 were cultured in SDA liquid medium at 25 °C for 10 days. The resulting cultures were then inoculated onto rice medium in 500 mL Erlenmeyer flasks and incubated at 25 °C for 40 days. The rice cultures of *Xylaria* sp. Y01 (10 kg) were harvested and extracted with 20 L of ethyl acetate at room temperature. The crude extract was obtained by evaporation under vacuum. The extract was partitioned five times between water and ethyl acetate, and the combined organic layers were concentrated under reduced pressure to yield a brown residue (150 g). The crude extract was applied to a macroporous resin D101 column (20 cm × 30 cm) and eluted successively with 0% (discarded), 90%, and 100% aqueous methanol to obtain three fractions (Fr. A–C). Fr. A was further subjected to silica gel column chromatography and eluted with a petroleum ether (PE)–ethyl acetate (EA)–methanol gradient (*v*/*v*/*v*, 1:0:0 to 0:0:1) to yield five fractions (Fr. A1–A5).

Fr. A1 was purified by pre-HPLC (flow rate 2.5 or 3 mL/min) to obtain **12** (26.6 mg, 40% acetonitrile, *t_R_* = 22.0 min, SinoChrom ODS-AP column), **13** (68.2 mg, 50% methanol, *t_R_* = 35.0 min, Supersil C8: 10.0 mm × 250 mm, 5 μm), and **16** (4.1 mg, 67% acetonitrile, *t_R_* = 20.0 min, Supersil C8 column). Fr. A2 was fractionated over a Microporous resin (MCI) and eluted with methanol-H_2_O (*v*/*v* 20:80, 40:60, 60:40, 80:20, 100:0, 20 mL/min) to yield five subfractions (A2a–A2e). The remaining Fr. A2a was purified by pre-HPLC (flow rate 2.5 or 3 mL/min) to yield **5** (50.3 mg, 59% methanol, *t_R_* = 30 min, SinoChrom ODS-AP column), **6** (3.6 mg, 63% methanol, *t_R_* = 31.5 min, SinoChrom ODS-AP column), **7** (9.4 mg, 31% acetonitrile, *t_R_* = 47 min, Supersil C8 column), **8** (5.3 mg, 59% methanol, *t_R_* = 41.0 min, SinoChrom ODS-AP column), **14** (8.3 mg, 33% acetonitrile, *t_R_* = 38 min, Supersil C8 column), **15** (5.0 mg, 33% acetonitrile, *t_R_* = 36.5 min, Supersil C8 column), and **17** (1.2 mg, 59% methanol, *t_R_* = 33 min, SinoChrom ODS-AP column). The remained Fr. A2b was purified by pre-HPLC (flow rate 2.5 or 3 mL/min) successively to yield **2** (1.1 mg, 39% acetonitrile, *t_R_* = 22.0 min, Supersil C8 column), **3** (1.2 mg, 68% methanol, *t_R_* = 33.0 min, SinoChrom ODS-AP column), **4** (0.9 mg, 72% methanol, *t_R_* = 25.0 min, SinoChrom ODS-AP column), **9** (1.0 mg, 39% acetonitrile, *t_R_* = 22.0 min, SinoChrom ODS-AP column), **10** (0.8 mg, 54% methanol, *t_R_* = 28.0 min, SinoChrom ODS-AP column), and **11** (1.3 mg, 68% methanol, *t_R_* = 29.0 min, SinoChrom ODS-AP column). The remaining Fr. A2c was purified by pre-HPLC (flow rate 2.5 mL/min) to yield **1** (10.7 mg, 34% methanol, *t_R_* = 23.0 min, SinoChrom ODS-AP column). The isolation procedure for compounds **18**–**27** was carried out according to the previously reported method [12].

Curtachalasin Y1 (**1**): Colorless powder. ${\left[\alpha \right]}_{D}^{25}$ +49.4 (c 1.34, MeOH). IR (KBr): *ν*_max_ 3428.1, 2929.8, 1744.2, 1694.1, 1451.2, 1380.7, 1245.0, 1101.3, 1036.2, 705.2 cm^−1^. UV (acetonitrile) *λ*_max_ 210, 260 nm. ^1^H NMR (600 MHz in CDCl_3_) and ^13^C NMR (150 MHz in CDCl_3_) data, see Table 1 and Table 2. HRESIMS *m*/*z* 578.2724 [M + Na]^+^ (calcd. for C_31_H_41_NO_8_Na^+^, 578.2725 Δ −0.2 ppm).

Curtachalasin Y2 (**2**): Colorless powder. ${\left[\alpha \right]}_{D}^{25}$ +303.0 (c 0.14, MeOH). IR (KBr): *ν*_max_ 3380.1, 2924.3, 2862.0, 1749.7, 1706.3, 1671.0, 1451.2, 1375.3, 1234.2, 1030.7, 941.2, 737.8 cm^−1^. UV (MeOH) *λ*_max_ 205 nm. ^1^H NMR (600 MHz in CDCl_3_) and ^13^C NMR (150 MHz in CDCl_3_) data, see Table 1 and Table 2. HRESIMS *m*/*z* 1133.5575 [2M + Na]^+^ (calcd. for C_62_H_82_N_2_O_16_Na^+^, 1133.5575, Δ 0.0 ppm), *m*/*z* 1075.5551 [2M + H − 2H_2_O]^+^ (calcd. for C_62_H_79_N_2_O_14_^+^, 1075.5526, Δ +2.7 ppm).

Curtachalasin Y3 (**3**): Colorless powder. ${\left[\alpha \right]}_{D}^{25}$ +54.0 (c 0.10, MeOH). IR (KBr): *ν*_max_ 3467.0, 2401.8, 2959.6, 2924.3, 1733.4, 1700.8, 1679.1, 1646.6, 1386.1, 1356.3, 1256.0, 1106.7, 1041.6 cm^−1^. UV (acetonitrile) *λ*_max_ 205 nm. ^1^H NMR (600 MHz in CDCl_3_) and ^13^C NMR (150 MHz in CDCl_3_) data, see Table 1 and Table 2. HRESIMS *m*/*z* 556.2914 [M + H]^+^ (calcd. for C_31_H_42_NO_8_^+^, 556.2905, Δ +1.6 ppm).

Curtachalasin Y4 (**4**): Colorless powder. ${\left[\alpha \right]}_{D}^{25}$ +9.6 (c 0.08, MeOH). IR (KBr): *ν*_max_ 3439.9, 2921.6, 2346.5, 1747.0, 1696.6, 1443.1, 1378.0, 1245.0, 1038.9 cm^−1^. UV (acetonitrile) *λ*_max_ 205 nm. ^1^H NMR (600 MHz in CDCl_3_) and ^13^C NMR (150 MHz in CDCl_3_) data, see Table 1 and Table 2. HRESIMS *m*/*z* 1133.5592 [2M + Na]^+^ (calcd. for C_62_H_82_N_2_O_16_Na^+^, 1133.5575, Δ +1.5 ppm), *m*/*z* 578.2746 [M + Na]^+^ (calcd. for C_31_H_41_NO_8_Na^+^, 578.2725, Δ +3.6 ppm).

Curtachalasin Y5 (**5**): Colorless powder. ${\left[\alpha \right]}_{D}^{25}$ +31.9 (c 2.52, MeOH). IR (KBr): *ν*_max_ 3429.0, 2924.3, 2311.2, 1749.7, 1714.4, 1638.4, 1459.4, 1375.3, 1256.0, 1228.8, 1025.3 cm^−1^. UV (in MeOH) *λ*_max_ 205 and 280 nm. ^1^H NMR (600 MHz in pyridine-*d*_5_) and ^13^C NMR (150 MHz in pyridine-*d*_5_) data, see Table 3. HRESIMS *m*/*z* 542.2752 [M + H]^+^ (calcd. for C_30_H_40_NO_8_^+^, 542.2749, Δ +0.6 ppm).

Curtachalasin Y6 (**6**): Colorless powder. ${\left[\alpha \right]}_{D}^{25}$ +165.4 (c 0.44, MeOH). IR (KBr): *ν*_max_ 3467.0, 2919.0, 1749.7, 1706.3, 1673.7, 1451.2, 1378.0, 1231.5, 1022.6, 702.5 cm^−1^. UV (MeOH) *λ*_max_ 205 and 285 nm. ^1^H NMR (600 MHz in CDCl_3_) and ^13^C NMR (150 MHz in CDCl_3_) data, see Table 3. HRESIMS *m*/*z* 592.2907 [M + Na]^+^ (calcd. for C_32_H_43_NO_8_Na^+^, 592.2881, Δ +4.4 ppm).

Curtachalasin Y7 (**7**): Colorless powder. ${\left[\alpha \right]}_{D}^{25}$ +9.6 (c 1.00, MeOH). IR (KBr): *ν*_max_ 3514.5, 3449.6, 3355.0, 2965.4, 2930.3, 2854.5, 1748.3, 1699.6, 1599.5, 1437.3, 1372.4, 1231.7, 1083.0, 96.13, 707.0, 471.7 cm^−1^. UV (acetonitrile) *λ*_max_ 205 nm. ^1^H NMR (600 MHz in CD_3_OD) and ^13^C NMR (150 MHz in CD_3_OD) data, see Table 4. HRESIMS *m*/*z* 1069.5055 [2M + Na]^+^ (calcd. for C_60_H_74_N_2_O_14_Na^+^, 1069.5033, Δ +2.1 ppm).

Curtachalasin Y8 (**8**): Colorless powder. ${\left[\alpha \right]}_{D}^{25}$ +115.3 (c 0.66, MeOH). IR (KBr): *ν*_max_ 3412.7, 2956.9, 2859.2, 2859.2, 1676.4, 1581.5, 1416.0, 1264.0, 1101.3, 1036.2, 808.3 cm^−1^. UV (MeOH) *λ*_max_ 205 and 243 nm. ^1^H NMR (600 MHz in CDCl_3_) and ^13^C NMR (150 MHz in CDCl_3_) data, see Table 4. HRESIMS *m*/*z* 1101.4925 [2M + Na]^+^ (calcd. for C_60_H_74_N_2_O_16_Na ^+^, 1101.4931, Δ −0.5 ppm).

Curtachalasin Y9 (**9**): Colorless powder. ${\left[\alpha \right]}_{D}^{25}$ +350.7 (c 0.12, MeOH). UV (acetonitrile) *λ*_max_ 205 and 250 nm. ^1^H NMR (600 MHz in CDCl_3_) and ^13^C NMR (150 MHz in CDCl_3_) data, see Table 4. HRESIMS *m*/*z* 1069.5045 [2M + Na]^+^ (calcd. for C_60_H_74_N_2_O_14_Na^+^, 1069.5033, Δ +1.1 ppm), *m*/*z* 1047.5227 [2M + H]^+^ (calcd. for C_60_H_75_N_2_O_14_^+^, 1047.5213, Δ +0.9 ppm). The IR spectrum was not measured because of its low sample amount.

Curtachalasin Y10 (**10**): Colorless powder. ${\left[\alpha \right]}_{D}^{25}$ −416.0 (c 0.03, MeOH). UV (MeOH) *λ*_max_ 230 and 365 nm. ^1^H NMR (600 MHz in CDCl_3_) and ^13^C NMR (150 MHz in CDCl_3_) data, see Table 5 and Table 6. HRESIMS *m*/*z* 572.2257 [M + Na]^+^ (calcd. for C_31_H_35_NO_8_Na^+^, 572.2255, Δ +0.3 ppm). The IR spectrum was not measured because of its low sample amount.

Curtachalasin Y11 (**11**): Colorless powder. ${\left[\alpha \right]}_{D}^{25}$ +199.7 (c 0.17, MeOH). IR (KBr): *ν*_max_ 3420.8, 2967.7, 2921.6, 2875.5, 1736.0, 1709.0, 1681.8, 1432.3, 1375.3, 1228.8, 1036.2 cm^−1^. UV (acetonitrile) *λ*_max_ 210 and 263 nm. ^1^H NMR (600 MHz in CDCl_3_) and ^13^C NMR (150 MHz in CDCl_3_) data, see Table 5 and Table 6. HRESIMS *m*/*z* 546.2460 [M + Na]^+^ (calcd. for C_30_H_37_NO_7_Na^+^, 546.2463, Δ −0.6 ppm).

Curtachalasin Y12 (**12**): Colorless powder. ${\left[\alpha \right]}_{D}^{25}$ +87.7 (c 2.66, MeOH). IR (KBr): *ν*_max_ 3428.1, 3158.8, 2970.0, 2911.7, 2875.6, 1745.6, 1712.3, 1673.4, 1456.9, 1376.3, 1354.1, 1226.4, 1018.2, 943.2, 890.5, 732.2, 704.5 cm^−1^. UV (acetonitrile) *λ*_max_ 205 nm. ^1^H NMR (600 MHz in CD_3_OD) and ^13^C NMR (150 MHz in CD_3_OD) data, see Table 5 and Table 6. HRESIMS *m*/*z* 542.2748 [M + H]^+^ (calcd. for C_30_H_40_NO_8_^+^, 542.2749, Δ −0.2 ppm).

Curtachalasin Y13 (**13**): Colorless powder. ${\left[\alpha \right]}_{D}^{25}$ +4.2 (c 8.52, MeOH). IR (KBr): *ν*_max_ 3118.5, 2966.9, 2878.7, 2346.6, 1735.0, 1696.4, 1222.4, 1114.9, 1062.5, 985.4, 1010.2, 1037.7 cm^−1^. UV (MeOH) *λ*_max_ 205 nm. ^1^H NMR (600 MHz in CDCl_3_) and ^13^C NMR (150 MHz in CDCl_3_) data, see Table 5 and Table 6. HRESIMS *m*/*z* 542.2739 [M + H]^+^ (calcd. for C_30_H_40_NO_8_^+^, 542.2749, Δ −1.8 ppm).

### 3.4. Quantum Chemical Calculations

Candidate conformers were initially explored with Crest (version 2.12) at the GFN0 theoretical level [22,23], followed by optimization at the GFN2-xTB [24] level, where an energy cutoff of 4 kcal/mol was applied to discard higher-energy species. The retained conformers were then subjected to geometry optimization and vibrational frequency analysis at the B3LYP-D3(BJ)/TZVP (IEFPCM) level. The calculated ECD spectra were then simulated using SpecDis version 1.71 [25], and the contributions of conformers were Boltzmann-averaged based on Gibbs free energy. All DFT calculations were performed using the Gaussian 16 software package [26]. The optimized geometries, relative energies, and conformational populations of all calculated structures are provided in the Supplementary Material.

### 3.5. Cell Culture and Cell Viability Assay

RAW264.7 cells (Shanghai Zhong Qiao Xin Zhou Biotechnology Co., Ltd., Shanghai, China) were cultured in DMEM (NEST Biotechnology Co., Ltd., Wuxin, China) containing 10% fetal bovine serum, 2 mM L-glutamine, 1 mM sodium pyruvate, and 2 mg/mL gentamicin, and maintained at 37 °C in a humidified atmosphere with 5% CO_2_. For the assay, cells were seeded into 96-well plates at a density of 5 × 10^4^ cells per well and exposed to compounds **1**, **5**, **7**, **8**, **12**, **13**–**16**, and **18**–**27** at a concentration of 30 μM for 24 h under the same conditions. Afterwards, 10 μL of CCK-8 reagent was added to each well, and the plates were further incubated at 37 °C for 4 min. Absorbance was read at 450 nm using a microplate reader (Thermo Fisher Scientific, Waltham, MA, USA), with values obtained from untreated cells set as 100% viability. No significant cytotoxicity was observed; therefore, concentrations below 30 μM of these nineteen cytochalasins were used in subsequent experiments.

### 3.6. NO Production Inhibitory Activity

The nitrite content in culture supernatants, as an indicator of NO release, was quantified employing the Griess reagent. RAW264.7 cells were treated with lipopolysaccharide (LPS, 100 ng/mL) in the presence or absence of the nineteen cytochalasins (30 μM) for 24 h. A 100 µL aliquot of the cell culture supernatant was mixed with 100 µL of Griess reagent and incubated for 10 min at room temperature. The absorbance was then measured at 540 nm.

### 3.7. RNA Extraction and Sequencing

Total RNA was extracted using mirVana^TM^ miRNA Isolation Kit (Ambion, Thermo Fisher Scientific, Waltham, MA, USA) according to the supplier’s recommendations. RNA quality and concentration were assessed on an Agilent 2100 Bioanalyzer (Agilent Technologies, Santa Clara, CA, USA); only samples with an RNA integrity number (RIN) ≥ 7 proceeded to library construction. cDNA libraries were constructed using the TruSeq Stranded mRNA LT Sample Prep Kit (Illumina, Inc., San Diego, CA, USA; Paired-End, PE150) as per the manufacturer’s instructions, and subsequently sequenced on an Illumina HiSeq^TM^ 2500 platform, producing 125/150 bp paired-end reads.

### 3.8. Differential Expression and KEGG Pathway Analyses

Gene expression levels were calculated as FPKM values with Cufflinks (v2.2.1, University of Washington, Seattle, WA, USA), and read counts per gene were derived using HTSeq-count (v0.11.2, European Molecular Biology Laboratory, Heidelberg, Germany). Differentially expressed genes (DEGs) were identified with the DESeq R package (v4.2.1), applying the functions estimateSizeFactors and nbinomTest. Significance was defined as a *p*-value < 0.05 and an absolute fold change >2 or <0.5. Hierarchical clustering was performed to reveal expression patterns among DEGs, while KEGG pathway enrichment was carried out in R based on the hypergeometric distribution.

### 3.9. Enzyme-Linked Immunosorbent (ELISA) Assay

Levels of TNF-α, IL-6, and MCP-1 in cell culture supernatants were determined using commercial ELISA kits from Aifang (Changsha, China), following the provided protocols.

### 3.10. RT-PCR Analysis

RAW264.7 cells were cultured in DMEM supplemented with 10% heat-inactivated FBS (NEST Biotechnology Co., Ltd., Nanjing, China) and 1% penicillin/streptomycin, and keep at 37 °C with 5% CO_2_. RT-PCR was used to detect gene expression. Total RNA was purified with an RNA isolation kit (Vazyme Biotechnology, No. RC112-01, Nanjing, China), and cDNA was synthesized using a reverse transcription kit. RT-PCR was performed using ChamQ Universal SYBR qPCR Master Mix (Vazyme Biotechnology, No. Q711-02, Nanjing, China). The following primers were used:

TNF-α-F: 5′-GCCTCTTCTCATTCCTGCTTGTGG-3′

TNF-α-R: 5′-GTGGTTTGTGAGTGTGAGGGTCTG-3′

SOCS3-F: 5′-ATGGTCACCCACAGCAAGTTT-3′

SOCS3-R: 5′-TCCAGTAGAATCCGCTCTCCT-3′

Cxcl10-F: 5′-CCAAGTGCTGCCGTCATTTTC-3′

Cxcl10-R: 5′-GGCTCGCAGGGATGATTTCAA-3′

### 3.11. Western Blotting

The harvested cells were washed with PBS and lysed with protein lysis buffer. Protein concentrations were determined using a BCA Protein Assay Kit (Multisciences, Hangzhou, China). Proteins were separated by SDS-polyacrylamide gel electrophoresis, transferred to a PVDF membrane (Merck Millipore, Billerica, MA, USA), and incubated with the designated SOCS3 antibody and α-tubulin antibody overnight. After washing three times with TBST and incubating with a secondary antibody at room temperature for 2 h, protein expression was detected with an ECL Prime Western Blotting Kit (NCM Biotech, P10100, Suzhou, China).

## 4. Statistical Analysis

Data from this experiment are presented as the mean ± standard error of the mean (mean ± SEM). Analysis was conducted using one-way or two-way ANOVA with GraphPad Prism 9.0.0 software. All statistical tests were two-tailed, with *p* < 0.05 considered statistically significant. Significance levels of *p* < 0.05, *p* < 0.01, *p* < 0.001, and *p* < 0.0001 are denoted by *, **, ***, and **** (or ^#^, ^##^, ^###^, ^####^), respectively. Each experiment was performed and analyzed with a minimum of three independent replicates.

## Figures and Tables

**Table 1:** ^1^H (600 MHz) NMR data of **1**–**4** in CDCl_3_ (*δ* in ppm, *J* in Hz).

No.	1	2	3	4
3	3.38 m	3.44 dd (7.8, 7.5)	3.46 br dd (9.2, 6.4)	3.34 br dd (8.4, 6.8)
4	2.37 br s	2.37 br s	2.40 br s	2.37 br s
7	4.25 d (9.7)	4.11 d (2.7)	3.75 d (10.5)	3.94 br d (9.8)
8	1.98 dd (10.0, 9.7)	1.94 m	1.93 overlapped	1.92 dd (9.8, 9.5)
10	2.86 dd (13.4, 6.8)	2.94 dd (13.4, 7.8)	2.97 dd (13.6, 9.2)	2.95 dd (13.6, 8.4)
	2.94 dd (13.4, 8.4)	2.88 dd (13.4, 7.5)	2.88 dd (13.6, 6.4)	2.88 dd (13.6, 6.8)
11	1.42 s	1.40 s	1.46 s	1.42 s
12	1.68 s	1.78 s	1.68 s	1.65 s
13	4.65 dd (10.4, 10.0)	4.17 (dd 10.3, 10.3)	4.00 ddd (11.5, 3.5, 3.5)	4.51 dd (9.5, 9.5)
14	1.73 m	1.58 overlapped	1.68 m	1.57 m
15	1.85 overlapped	1.92 m	1.96 ddd (12.6, 12.4, 12.1)	2.04 ddd (12.5, 3.7, 3.7)
	1.45 ddd (12.5, 12.1, 12.1)	1.46 ddd (12.3, 12.3, 12.4)	1.44 overlapped	1.36 ddd (12.5, 12.5, 12.5)
16	1.84 overlapped	1.84 m	1.93 overlapped	1.85 m
19	3.39 d (10.5)	3.40 dd (10.5, 6.8)	3.39 dd (10.5, 7.5)	3.41 dd (10.4, 7.4)
20	2.41 ddd (12.3, 10.5, 2.1)	2.56 ddd (11.0, 10.5, 1.7)	2.38 ddd (12.4, 10.5, 2.0)	2.45 ddd (12.4, 10.4, 1.9)
21	5.43 d (2.1)	5.38 d (1.7)	5.53 d (2.0)	5.47 d (1.9)
22	2.26 s	2.22 s	2.26 s	2.26 s
23	0.79 d (6.5)	0.79 d (6.7)	0.78 d (6.3)	0.77 d (6.9)
25/29	7.13 d (7.7)	7.15 d (7.4)	7.14 d (7.4)	7.14 d (7.2)
26/28	7.30 t (7.7)	7.30 t (7.4)	7.32 t (7.4)	7.31 t (7.2)
27	7.23 t (7.7)	7.23 t (7.4)	7.25 t (7.4)	7.24 t (7.2)
21-OAc	2.26 s	2.26 s	2.24 s	2.26 s
7-OCH_3_/13-OCH_3_	3.57 s	3.60 s	3.62 s	3.49 s
NH	5.87 s		5.70 s	5.54 br s
7-OH	5.16 s			
13-OH			5.93 d (11.5)	4.88 s
17-OH				3.67 s
19-OH			2.83 d (7.5)	

**Table 2:** ^13^C (150 MHz) NMR data of 1–4 in CDCl_3_ (δ in ppm).

No.	1	2	3	4
1	175.2, C	177.5, C	176.8, C	174.9, C
3	59.4, CH	59.1, CH	60.2, CH	60.0, CH
4	48.6, CH	49.0, CH	49.4, CH	49.0, CH
5	125.0, C	127.8, C	125.3, C	127.0, C
6	133.0, C	131.8, C	134.0, C	133.0, C
7	70.7, CH	67.8, CH	76.5, CH	83.2, CH
8	43.2, CH	43.7, CH	43.2, CH	43.2, CH
9	50.4, C	49.5, C	51.6, C	50.8, C
10	43.8, CH_2_	44.2, CH_2_	43.5, CH_2_	43.9, CH_2_
11	17.4, CH_3_	17.4, CH_3_	17.4, CH_3_	17.4, CH_3_
12	14.2, CH_3_	18.4, CH_3_	14.7, CH_3_	13.4, CH_3_
13	84.5, CH	78.8, CH	66.7, CH	72.0, CH
14	39.5, CH	39.8, CH	37.5, CH	39.1, CH
15	31.5, CH_2_	31.9, CH_2_	31.2, CH_2_	31.6, CH_2_
16	36.1, CH	36.3, CH	36.0, CH	36.1, CH
17	83.6, C	83.9, C	83.8, C	83.7, C
18	212.4, C	213.3, C	212.4, C	212.1, C
19	72.2, CH	72.5, CH	72.6, CH	72.4, CH
20	41.6, CH	41.6, CH	38.1, CH	40.5, CH
21	71.5, CH	71.9, CH	72.0, CH	71.3, CH
22	25.3, CH_3_	25.4, CH_3_	25.0, CH_3_	24.9, CH_3_
23	15.0, CH_3_	15.0, CH_3_	14.3, CH_3_	14.6, CH_3_
24	137.7, C	137.5, C	137.2, C	137.4 C
25/29	129.2, CH	129.2, CH	129.0, CH	129.1, CH
26/28	129.0, CH	129.0, CH	128.9, CH	128.9, CH
27	127.1, CH	127.2, CH	127.1, CH	127.0, CH
21-OAc	172.1, C	171.9, C	171.6, C	171.9, C
	21.2, CH_3_	21.2, CH_3_	21.0, CH_3_	21.1, CH_3_
7-OCH_3_/13-OCH_3_	59.9, CH_3_	60.9, CH_3_	60.9, CH_3_	59.1, CH_3_

**Table 3:** ^1^H (600 MHz) and ^13^C (150 MHz) NMR data of **5**–**7** (*δ* in ppm, *J* in Hz).

No.	5 ^a^		6 ^b^		7 ^c^	
	*δ*_H_	*δ*_C_	*δ*_H_	*δ*_C_	*δ*_H_	*δ*_C_
1		178.5, C		176.1, C		176.8, C
3	3.81 dd (7.4, 7.4)	61.5, CH	3.40 m	59.3, CH	3.29 overlapped	61.4, CH
4	2.90 s	50.3, CH	2.29 br s	49.1, CH	2.53 br s	49.0, CH
5		125.9, C		129.2, C		128.4, C
6		135.8, C		130.2, C		135.3, C
7	4.83 br d (10.7)	66.2, CH	4.00 d (2.1)	74.3, CH	3.78 br d (10.8)	70.0, CH
8	2.49 dd (10.7, 3.3)	45.5, CH	1.85 overlapped	46.2, CH	2.40 m	41.0, CH
9		53.3, C		48.8, C		50.9, C
10	3.30 overlapped	44.2, CH_2_	1.84 overlapped	45.3, CH_2_	3.15 dd (13.2, 9.2)	43.8, CH_2_
	3.37 dd (13.3, 7.4)		2.86 br d (7.4)		3.00 dd (13.2, 5.5)	
11	1.46 s	17.5, CH_3_	1.35 s	19.3, CH_3_	1.15 s	17.5, CH_3_
12	1.97 s	15.4, CH_3_	1.76 s	17.4, CH_3_	1.63 s	14.8, CH_3_
13	4.80 ddd (11.4 3.6, 3.3)	67.8, CH	4.62 dd (10.9, 9.0)	69.1, CH	5.81 dd (4.4, 2.1)	120.6, CH
14	2.10 dddd (12.3, 12.3, 3.9, 3.6)	39.0, CH	1.42 m	41.3, CH		134.5, C
15	2.62 ddd (12.7, 12.4, 12.3)	32.4, CH_2_	1.42 m	31.7, CH_2_	2.28 m	38.2, CH_2_
	1.53 ddd (12.7, 3.6, 3.6)		1.86 m		2.05 dd (14.1, 4.4)	
16	2.15 overlapped	36.5, CH	1.82 m	36.2, CH	1.91 m	37.2, CH
17		85.6, C		83.8, C		85.9, C
18		215.8, C		213.1, C		214.6, C
19	4.04 d (10.4)	73.9, CH	3.41 overlpped	72.8, CH	3.69 d (10.7)	73.9, CH
20	3.29 overlapped	38.8, CH	2.79 ddd (10.8, 10.8, 2.3)	41.1, CH	2.85 m	43.6, CH
21	6.41 s	72.9, CH	5.31 d (2.3)	72.6, CH	5.80 d (4.1)	72.3, CH
22	2.44 s	26.5, CH_3_	2.27 s	25.7, CH_3_	2.26 s	25.9, CH_3_
23	0.95 d (6.6)	15.7, CH_3_	0.79 d (6.7)	14.8, CH_3_	0.75 d (6.7)	15.1, CH_3_
24		139.3, C		137.6, CH		139.9, C
25/29	7.23 overlapped	129.9, CH	7.15 d (7.3)	129.2, CH	7.30 overlapped	130.7, CH
26/28	7.26 overlapped	129.0, CH	7.29 t (7.3)	128.8, CH	7.32 overlapped	129.7, CH
27	7.27 overlapped	126.9, CH	7.22 t (7.3)	126.9, CH	7.21 t (7.7)	127.7, CH
21-OAc		170.8, C		171.9, C		172.6, C
	2.29 s	20.9, CH_3_	2.20 s	21.0, CH_3_	2.19 s	21.0, CH_3_
7-OCH_2_CH_3_			3.53 dq (9.2, 7.0)	66.7, CH_2_		
			3.64 dq (9.2, 7.0)			
7-OCH_2_CH_3_			1.09 dd (7.0, 7.0)	15.6, CH_3_		
NH	9.88 s		5.91 s			
13-OH	7.09 d (11.4)					
17-OH			3.69 br s ^d^			
19-OH	6.59 br s		3.01 s			

^a^ Measured in pyridine-*d*_5_ ^b^ Measured in CD_3_Cl ^c^ Measured in CD_3_OD ^d^ Assigned by the chemical shift value.

**Table 4:** ^1^H (600 MHz) and ^13^C (150 MHz) NMR data of **8** and **9** in CDCl_3_ (*δ* in ppm, *J* in Hz).

No.	8		9	
	*δ*_H_	*δ*_C_	*δ*_H_	*δ*_C_
1		173.8, C		174.3, C
3	3.48 br dd (7.6, 7.4)	59.0, CH	3.78 ddd (8.8, 8.8, 3.3)	57.9, CH
4	2.75 br s	48.9, CH	2.89 d (8.8)	52.9, CH
5		148.8, C		147.2, C
6		133.1, C		132.3, C
7		199.3, C		195.8, C
8	2.72 d (9.6)	48.9, CH	2.64 (overlapped)	42.7, CH
9		50.4, C		53.7, C
10	3.08 dd (13.5, 7.6)	43.5, CH_2_	3.37 dd (13.7, 3.1)	42.8, CH_2_
	2.96 dd (13.5, 7.4)		2.63 dd (13.7, 10.8)	
11	1.58 s	19.2, CH_3_	2.05 s	22.0, C
12	1.72 s	11.8, CH_3_	1.89 s	11.9, C
13	4.80 dd (9.6, 9.6)	68.2, CH	1.52 overlapped	26.6, CH
			2.35 ddd (12.8, 2.6, 2.6)	
14	1.59 overlapped	37.9, CH	1.54 overlapped	29.4, CH
15	2.06 overlapped	31.3, CH_2_	1.50 overlapped	35.8, CH_2_
	1.36 ddd (12.7, 12.7, 12.5)		1.26 br dd (12.3, 12.3)	
16	1.82 br dq (12.5, 6.7)	36.0, CH	1.85 ddq (12.3, 4.1, 6.7)	36.0 C
17		83.7, C		84.3, C
18		212.4, C		211.4, C
19	3.43 d (10.4)	72.3, CH	3.42 dd (10.1, 10.1)	72.0, CH
20	2.40 ddd (12.4, 10.3, 2.0)	41.4, CH	2.83 ddd (10.1, 10.1, 2.6)	42.0, CH
21	5.61 br s	70.8, CH	5.54 d (2.6)	72.6, CH
22	2.27 s	24.9, CH_3_	2.21 s	24.0, CH_3_
23	0.76 d (6.7)	14.6, CH_3_	0.69 d (6.7)	14.7, CH_3_
24		137.0, C		136.9, C
25/29	7.18 d (7.7)	129.1, CH	7.18 d (7.4)	128.9, CH
26/28	7.33 t (7.7)	129.0, CH	7.35 t (7.4)	129.4, CH
27	7.27 t (7.7)	127.3, CH	7.28 t (7.4)	127.6, CH
21-OAc		171.4, C		170.6, C
	2.30 s	21.1, CH_3_	1.91 s	20.6, CH_3_
NH	6.03 s		5.47 s	
17-OH			3.89 s	
19-OH			2.09 s	

**Table 5:** ^1^H (600 MHz) NMR data of **10**–**13** (*δ* in ppm, *J* in Hz).

No.	10 ^a^	11 ^a^	12 ^b^	13 ^a^
3	3.47 br d (11.4)	3.49 ddd (10.5, 7.0, 3.1)	3.34 m	3.25 br dd (7.9, 6.5)
4	3.29 br s	2.35 dd (7.0, 5.3)	2.17 br dd (6.3, 2.2)	2.05 dd (5.6, 2.1)
5		2.61 m	2.54 dq (6.8, 6.3)	2.64 m
7	5.99 s	6.52 br s	4.26 d (10.5)	4.25 d (9.4)
8			2.19 dd (10.5, 3.5)	2.20 dd (10.0, 9.4)
10	3.62 dd (11.7, 3.5)	3.16 dd (13.7, 3.1)	2.73 dd (13.1, 9.3)	2.76 dd (13.4, 7.9)
	2.84 dd (13.4, 11.7)	2.53 dd (13.7, 10.5)	2.93 dd (13.1, 5.3)	2.67 dd (13.4, 6.5)
11	10.15 s	1.29 d (7.3)	0.42 d (6.8)	0.72 d 6.5
12	2.30 d (1.7)	5.15 br s	5.15 s	5.20 br s
		5.10 br s	4.95 s	4.97 br s
13	6.31 br d (2.6)	4.00 dd (11.8, 3.2)	3.96 dd (3.2, 3.2)	4.56 dd (10.0, 10.0)
14		1.76 m	1.81 dddd (12.6, 12.3, 3.2, 3.2)	1.58 ddd (12.1, 10.0, 3.4)
15	3.63 br d (2.8)	1.38 dd (10.0, 4.3)	1.85 ddd (12.6, 12.5, 12.5)	1.90 ddd (13.0, 3.8, 3.4)
		1.91 m	1.33 ddd (12.6, 3.6, 3.3)	1.30 br dd (13.0, 12.1)
16	2.13 dq (7.1, 2.8)	1.90 m	1.99 m	1.83 m
19	3.53 dd (10.6, 8.6)	3.45 dd (10.2, 8.9)	3.49 d (10.5)	3.42 d (10.4)
20	3.19 ddd (10.6, 3.8, 3.0)	2.94 ddd (12.9, 10.2, 2.5)	2.45 ddd (12.3, 10.5, 2.2)	2.49 ddd (12.1, 10.4, 2.2)
21	5.68 d (3.8)	5.54 d (2.6)	5.51 d (2.2)	5.28 d (2.2)
22	2.31 s	2.26 s	2.26 s	2.25 s
23	1.06 d (7.0)	0.77 d (5.8)	0.76 d (6.7)	0.76 d (6.6)
25/29	7.22 overlapped	7.13 d (7.4)	7.21 overlapped	7.11 d (7.3)
26/28	7.30 t (7.6)	7.32 t (7.4)	7.30 t (7.0)	7.28 t (7.3)
27	7.23 overlapped	7.25 t (7.4)	7.21 overlapped	7.20 t (7.3)
21-OAc	2.10 s	2.17 s	2.26 s	2.25 s
15-OCH_3_	3.40 s			
NH	5.46 s	5.62 br s		6.04 s
13-OH		4.85 d (11.8)		
17-OH	4.59 s	3.72 s ^c^		
19-OH	2.75 d (8.6)			

^a^ Measured in CDCl_3_ ^b^ Measured in CD_3_OD ^c^ Assigned by the chemical shift value.

**Table 6:** ^13^C (150 MHz) NMR data of **10**–**13** (*δ* in ppm).

No.	10 ^a^	11 ^a^	12 ^b^	13 ^a^
1	173.2, C	176.1, C	178.7, C	175.3, C
3	62.0, CH	54.4, CH	55.8, CH	53.4, CH
4	42.9, CH	50.2, CH	48.3, CH	47.6, CH
5	131.2, C	34.3, CH	32.8, CH	32.1, CH
6	147.5, C	143.3, C	151.1, C	148.4, C
7	128.7, CH	132.8, CH	69.4, CH	73.9, CH
8	137.4, C	134.1, CH	43.1, CH	42.9, CH
9	52.0, C	53.5, C	54.0, C	51.3, C
10	44.2, CH_2_	44.4, CH_2_	44.2, CH_2_	44.4, CH_2_
11	190.0, CH	15.6, CH_3_	12.9, CH_3_	12.9, CH_3_
12	17.8, CH_2_	113.4, CH_2_	114.0, CH_2_	114.1, CH_2_
13	128.1, CH	75.2, CH	68.8, CH	73.8, CH
14	137.4, C	38.1, CH	39.3, CH	40.3, CH
15	85.6, CH_2_	30.9, CH_2_	32.4, CH_2_	31.3, CH_2_
16	39.1, CH	35.8, CH	36.9, CH	36.0, CH
17	84.7, C	83.8, C	85.7, C	84.0, C
18	215.2, C	212.0, C	215.2, C	212.3, C
19	73.9, CH	72.5, CH	74.0, CH	72.6, CH
20	40.4, CH	38.6, CH	38.9, CH	41.1, CH
21	71.8, CH	74.7, CH	73.6, CH	72.5, CH
22	29.3, CH_3_	24.9, CH_3_	26.0, CH_3_	25.1, CH_3_
23	11.5, CH_3_	14.8, CH_3_	15.4, CH_3_	14.9, CH_3_
24	138.6, C	137.2, C	138.8, C	137.3, C
25/29	129.2, CH	129.2, CH	130.7, CH	129.3, CH
26/28	129.0, CH	128.7, CH	129.6, CH	128.9, CH
27	126.9, CH	127.3, CH	127.8, CH	127.1, CH
21-OAc	171.6, C	171.3, C	172.4, C	171.7, C
	21.0, CH_3_	20.8, CH_3_	20.9, CH_3_	21.1, CH_3_
15-OCH_3_	56.6, CH_3_			

^a^ Measured in CDCl_3_ ^b^ Measured in CD_3_OD.

**Table 7:** The IC_50_ values of tested compounds for inhibitory effects on LPS-induced NO production of RAW264.7 macrophages.

Compounds	IC50 (μM)
**1**, **5**, **7, 8**, **13–16**, **19**, **20**, **23**, **26**, and **27**	>60
**12**	55.4 ± 0.3
**21**	43.8 ± 5.5
**22**	17.8 ± 0.7
Dexamethasone	14.9 ± 1.3

## Data Availability

The original contributions presented in this study are included in the article. Further inquiries can be directed to the corresponding author.

## Supplementary Materials

The following supporting information can be downloaded at: https://www.mdpi.com/article/10.3390/ijms27146313/s1.
