# Supplementary material for: Curtachalasins Y1–Y13, Anti-Inflammatory Cytochalasans from the Soil Fungus Xylaria sp. Y01
Source: Int J Mol Sci. 2026 Jul 15;27(14):6313. doi: 10.3390/ijms27146313 (PMC13409777; doi:10.3390/ijms27146313)
Supplement: Supplementary file 1 [file ijms-27-06313-s001.zip › ijms-4393567-supplementary.pdf]

## Supplementary Material

# Curtachalasins Y1–Y13, Anti-Inflammatory Cytochalasans from the Soil Fungus *Xylaria* sp. Y01

**Yi-Yun Yuan [1,2,†], Yang Xie [1,†], Xi Zhou [2], Liang Tu [2], Ying-Meng Leng [2], Qi-An Chen [1,2], Qing-Hui Xiao [1,2],**
**Shao Liu [1,2], Wen-Xuan Wang [2] and Jing Li [1,2,*]**

1 Department of Pharmacy, National Clinical Research Center for Geriatric Disorders, Xiangya Hospital, Central South University, Changsha 410008, China
2 Xiangya School of Pharmaceutical Sciences, Central South University, Changsha 410083, China
* Correspondence: lijingliyun@csu.edu.cn
† These authors contributed equally to this work.

# Table of contents


Figure S1. $^1$H NMR ($CDCl_3$, 600 MHz) of **1**. 7
Figure S2. Partial $^1$H NMR ($CDCl_3$, 600 MHz) of **1**. 7
Figure S3. $^{13}$C NMR ($CDCl_3$, 150 MHz) of **1**. 8
Figure S4. DEPT 135 spectra of **1** 8
Figure S5. HSQC of **1**. 9
Figure S6. $^1$H−$^1$H COSY of **1** 9
Figure S7. HMBC of **1**. 10
Figure S8. NOESY of **1** 10
Figure S9. HRESIMS of **1**. 11
Figure S10. CD spectrum (acetonitrile) of **1**. 11
Figure S11. IR spectrum of **1**. 11
Figure S12. $^1$H NMR ($CDCl_3$, 600 MHz) of **2**. 12
Figure S13. Partial $^1$H NMR ($CDCl_3$, 600 MHz) of **2**. 12
Figure S14. $^{13}$C NMR ($CDCl_3$, 150 MHz) of **2**. 13
Figure S15. DEPT 135 spectra of **2** 13
Figure S16. HSQC of **2**. 14
Figure S17. $^1$H−$^1$H COSY of **2** 14
Figure S18. HMBC of **2**. 15
Figure S19. NOESY of **2** 15
Figure S20. HRESIMS of **2**. 16
Figure S21. CD spectrum (acetonitrile) of **2**. 16
Figure S22. IR spectrum of **2**. 16
Figure S23. $^1$H NMR ($CDCl_3$, 600 MHz) of **3**. 17
Figure S24. Partial $^1$H NMR ($CDCl_3$, 600 MHz) of **3**. 17
Figure S25. $^{13}$C NMR ($CDCl_3$, 150 MHz) of **3**. 18
Figure S26. DEPT 135 spectra of **3** 18
Figure S27. HSQC of **3**. 19
Figure S28. $^1$H−$^1$H COSY of **3** 19
Figure S29. HMBC of **3**. 20
Figure S30. NOESY of **3** 20
Figure S31. HRESIMS of **3**. 21
Figure S32. CD spectrum (acetonitrile) of **3**. 21
Figure S33. IR spectrum of **3**. 21
Figure S34. $^1$H NMR ($CDCl_3$, 500 MHz) of **4**. 22
Figure S35. Partial $^1$H NMR ($CDCl_3$, 600 MHz) of **4**. 22
Figure S36. $^{13}$C NMR ($CDCl_3$, 150 MHz) of **4**. 23
Figure S37. DEPT 135 spectra of **4** 23
Figure S38. HSQC of **4**. 24
Figure S39. $^1$H−$^1$H COSY of **4** 24
Figure S40. HMBC of **4**. 25
Figure S41. NOESY of **4** 25
Figure S42. HRESIMS of **4**. 26

Figure S43. CD spectrum (methanol) of **4**. ....26
Figure S44. IR spectrum of **4**. ....26
Figure S45. $^1$H NMR (pyridine-$d_5$., 600 MHz) of **5**. ....27
Figure S46. Partial $^1$H NMR ($CDCl_3$, 600 MHz) of **5**. ....27
Figure S47. $^{13}$C NMR (pyridine-$d_5$., 150 MHz) of **5**. ....28
Figure S48. DEPT 135 spectra of **5** in pyridine-$d_5$. ....28
Figure S49. HSQC of **5** in pyridine-$d_5$. ....29
Figure S50. $^1$H−$^1$H COSY of **5** in pyridine-$d_5$. ....30
Figure S51. HMBC of **5** in pyridine-$d_5$. ....30
Figure S52. NOESY of **5** in pyridine-$d_5$. ....30
Figure S53. $^1$H NMR ($CDCl_3$, 600 MHz) of **5**. ....31
Figure S54. $^{13}$C NMR ($CDCl_3$, 150 MHz) of **5**. ....32
Figure S55. DEPT 135 spectra of **5** in $CDCl_3$. ....32
Figure S56. $^1$H−$^1$H COSY of **5** in $CDCl_3$. ....32
Figure S57. HSQC of **5** in $CDCl_3$. ....33
Figure S58. NOESY of **5** in $CDCl_3$. ....33
Figure S59. $^1$H NMR ($CD_3OD$, 600 MHz) of **5**. ....34
Figure S60. $^{13}$C NMR ($CD_3OD$, 150 MHz) of **5**. ....34
Figure S61. HSQC of **5** in $CD_3OD$. ....35
Figure S62. HRESIMS of **5**. ....35
Figure S63. CD spectrum (methanol) of **5**. ....35
Figure S64. IR spectrum of **5**. ....36
Figure S65. $^1$H NMR ($CDCl_3$, 600 MHz) of **6**. ....36
Figure S66. Partial $^1$H NMR ($CDCl_3$, 600 MHz) of **6**. ....37
Figure S67. $^{13}$C NMR ($CDCl_3$, 150 MHz) of **6**. ....37
Figure S68. DEPT 135 spectra of **6**. ....38
Figure S69. HSQC of **6**. ....38
Figure S70. $^1$H−$^1$H COSY of **6**. ....39
Figure S71. HMBC of **6**. ....39
Figure S72. NOESY of **6**. ....40
Figure S73. HRESIMS of **6**. ....40
Figure S74. CD spectrum (methanol) of **6**. ....41
Figure S75. IR spectrum of **6**. ....41
Figure S76. $^1$H NMR ($CD_3OD$, 600 MHz) of **7**. ....42
Figure S77. Partial $^1$H NMR ($CDCl_3$, 600 MHz) of **7**. ....42
Figure S78. $^{13}$C NMR ($CD_3OD$, 150 MHz) of **7**. ....43
Figure S79. DEPT 135 spectra of **7**. ....43
Figure S80. HSQC of **7**. ....44
Figure S81. $^1$H−$^1$H COSY of **7**. ....44
Figure S82. HMBC of **7**. ....45
Figure S83. NOESY of **7**. ....45
Figure S84. HRESIMS of **7**. ....46
Figure S85. CD spectrum (acetonitrile) of **7**. ....46
Figure S86. IR spectrum of **7**. ....46

Figure S87. $^{1}$H NMR ($CDCl_3$, 600 MHz) of **8**. 47
Figure S88. Partial $^{1}$H NMR ($CDCl_3$, 600 MHz) of **8**. 47
Figure S89. $^{13}$C NMR ($CDCl_3$, 150 MHz) of **8**. 48
Figure S90. DEPT 135 spectra of **8**. 48
Figure S91. HSQC of **8**. 49
Figure S92. $^{1}$H−$^{1}$H COSY of **8**. 49
Figure S93. HMBC of **8**. 50
Figure S94. NOESY of **8**. 50
Figure S95. HRESIMS of **8**. 51
Figure S96. CD spectrum (methanol) of **8**. 51
Figure S97. IR spectrum of **8**. 51
Figure S98. $^{1}$H NMR ($CDCl_3$, 600 MHz) of **9**. 52
Figure S99. Partial $^{1}$H NMR ($CDCl_3$, 600 MHz) of **9**. 52
Figure S100. $^{13}$C NMR ($CDCl_3$, 150 MHz) of **9**. 53
Figure S101. DEPT 135 spectra of **9**. 53
Figure S102. HSQC of **9**. 54
Figure S103. $^{1}$H−$^{1}$H COSY of **9**. 54
Figure S104. HMBC of **9**. 55
Figure S105. NOESY of **9**. 55
Figure S106. HRESIMS of **9**. 56
Figure S107. CD spectrum (methanol) of **9**. 56
Figure S108. $^{1}$H NMR ($CDCl_3$, 600 MHz) of **10**. 57
Figure S109. Partial $^{1}$H NMR ($CDCl_3$, 600 MHz) of **10**. 57
Figure S110. Partial $^{1}$H NMR ($CDCl_3$, 600 MHz) of **10**. 58
Figure S111. $^{13}$C NMR ($CDCl_3$, 150 MHz) of **10**. 58
Figure S112. DEPT 135 spectra of **10**. 59
Figure S113. HSQC of **10**. 59
Figure S114. $^{1}$H−$^{1}$H COSY of **10**. 60
Figure S115. HMBC of **10**. 60
Figure S116. NOESY of **10**. 61
Figure S117. HRESIMS of **10**. 61
Figure S118. CD spectrum (methanol) of **10**. 62
Figure S119. $^{1}$H NMR ($CDCl_3$, 600 MHz) of **11**. 62
Figure S120. Partial $^{1}$H NMR ($CDCl_3$, 600 MHz) of **11**. 63
Figure S121. $^{13}$C NMR ($CDCl_3$, 150 MHz) of **11**. 63
Figure S122. DEPT 135 spectra of **11**. 64
Figure S123. HSQC of **11**. 64
Figure S124. $^{1}$H−$^{1}$H COSY of **11**. 65
Figure S125. HMBC of **11**. 65
Figure S126. NOESY of **11**. 66
Figure S127. HRESIMS of **11**. 66
Figure S128. CD spectrum (acetonitrile) of **11**. 67
Figure S129. IR spectrum of **11**. 67
Figure S130. $^{1}$H NMR ($CD_3OD$, 600 MHz) of **12**. 68

Figure S131. Partial $^{1}$H NMR ($CDCl_3$, 600 MHz) of **12**. ...... 68
Figure S132. $^{13}$C NMR ($CD_3OD$, 150 MHz) of **12**. ...... 69
Figure S133. DEPT 135 spectra of **12**. ...... 69
Figure S134. HSQC of **12**. ...... 70
Figure S135. $^{1}$H–$^{1}$H COSY of **12**. ...... 70
Figure S136. HMBC of **12**. ...... 71
Figure S137. NOESY of **12**. ...... 71
Figure S138. HRESIMS of **12**. ...... 72
Figure S139. CD spectrum (acetonitrile) of **12**. ...... 72
Figure S140. IR spectrum of **12**. ...... 72
Figure S141. $^{1}$H NMR ($CDCl_3$, 600 MHz) of **13**. ...... 73
Figure S142. Partial $^{1}$H NMR spectrum ($CDCl_3$, 600 MHz) of **13**. ...... 73
Figure S143. $^{13}$C NMR ($CDCl_3$, 150 MHz) of **13**. ...... 74
Figure S144. DEPT 135 spectra of **13**. ...... 74
Figure S145. HSQC of **13**. ...... 75
Figure S146. $^{1}$H–$^{1}$H COSY of **13**. ...... 75
Figure S147. HMBC of **13**. ...... 76
Figure S148. NOESY of **13**. ...... 76
Figure S149. HRESIMS of **13**. ...... 77
Figure S150. CD spectrum (methanol) of **13**. ...... 77
Figure S151. IR spectrum of **13**. ...... 77
Figure S152. HPLC chromatography of **12** and **13**. ...... 78
Table S1. The values of MAE, RMS, $P_{mean}$, and DP4 of the calculated chemical shifts of relative structures **1** and **2** fitting to each set of the experimental $^{13}$C NMR data of **1** and **2**, respectively. These red numbers represent the best matched structures. ...... 79
Table S2. The predicted values by GFN2NMR for **1** and **2** fitting to the experimental $^{13}$C NMR chemical shifts ($CDCl_3$) of **1**. ...... 79
Table S3. The predicted values by GFN2NMR for **1** and **2** fitting to the experimental $^{13}$C NMR chemical shifts ($CDCl_3$) of **2**. ...... 80
Table S4. The values of MAE, RMS, *Pmean*, and DP4 of the calculated chemical shifts of relative structures **3** and **4** fitting to each set of the experimental $^{13}$C NMR data of **3** and **4**, respectively. These red numbers represent the best matched structures. ...... 81
Table S5. The predicted values by GFN2NMR for **3** and **4** fitting to the experimental $^{13}$C NMR chemical shifts ($CDCl_3$) of **3**. ...... 81
Table S6. The predicted values by GFN2NMR for **3** and **4** fitting to the experimental $^{13}$C NMR chemical shifts ($CDCl_3$) of **4**. ...... 82
Table S7. The values of MAE, RMS, *Pmean*, and DP4 of the calculated chemical shifts of relative structures **5** and **14** fitting to each set of the experimental $^{13}$C NMR data of **5** and **14**, respectively. These red numbers represent the best matched structures. ...... 83
Table S8. The predicted values by GFN2NMR for **5** and **14** fitting to the experimental $^{13}$C NMR chemical shifts ($CDCl_3$) of **5**. ...... 83
Table S9. The predicted values by GFN2NMR for **5** and **14** fitting to the experimental $^{13}$C NMR chemical shifts ($CDCl_3$) of **14**. ...... 84
Table S10. The values of MAE, RMS, *Pmean*, and DP4 of the calculated chemical shifts of

relative structures **12** and **13** fitting to each set of the experimental $^{13}C$ NMR data of **12** and **13**, respectively. These red numbers represent the best matched structures........................................85
Table S11. The predicted values by GFN2NMR for **12** and **13** fitting to the experimental $^{13}C$ NMR chemical shifts ($CDCl_3$) of **12**. ..................................................................................85
Table S12. The predicted values by GFN2NMR for **12** and **13** fitting to the experimental $^{13}C$ NMR chemical shifts ($CDCl_3$) of **13**. ..................................................................................86
Table S13. Linear regression analysis of the experimental versus calculated $^{13}C$ NMR chemical shifts of **1**–**13**...........................................................................................................87
Table S14. Experimental $^{13}C$ NMR chemical shifts ($CDCl_3$) of **6** and the predicted values by GFN2NMR for **6**. .............................................................................................................88
Table S15. Experimental $^{13}C$ NMR chemical shifts ($CDCl_3$) of **7** and the predicted values by GFN2NMR for **7**. .............................................................................................................89
Table S16. Experimental $^{13}C$ NMR chemical shifts ($CDCl_3$) of **8** and the predicted values by GFN2NMR for **8**. .............................................................................................................89
Table S17. Experimental $^{13}C$ NMR chemical shifts ($CDCl_3$) of **9** and the predicted values by GFN2NMR for **9**. .............................................................................................................90
Table S18. Experimental $^{13}C$ NMR chemical shifts ($CDCl_3$) of **10** and the predicted values by GFN2NMR for **10**. ...........................................................................................................91
Table S19. Experimental $^{13}C$ NMR chemical shifts ($CDCl_3$) of **11** and the predicted values by GFN2NMR for **11**. ...........................................................................................................92
Table S20. Geometry data of conformers of compound **1**.............................................................94
Table S21. Geometry data of conformers of compound **2**...........................................................101
Table S22. Geometry data of conformers of compound **3**...........................................................124
Table S23. Geometry data of conformers of compound **4**...........................................................138
Table S24. Geometry data of conformers of compound **5**...........................................................146
Table S25. Geometry data of conformers of compound **6**...........................................................157
Table S26. Geometry data of conformers of compound **7**...........................................................178
Table S27. Geometry data of conformers of compound **8**...........................................................182
Table S28. Geometry data of conformers of compound **9**...........................................................189
Table S29. Geometry data of conformers of compound **10**.........................................................197
Table S30. Geometry data of conformers of compound **11**. ........................................................200
Table S31. Geometry data of conformers of compound **12**.........................................................207
Table S32. Geometry data of conformers of compound **13**.........................................................210
Table S33. Experimental $^{13}C$ NMR chemical shifts and their deviations ($CDCl_3$) of **5** and **14**. ...226

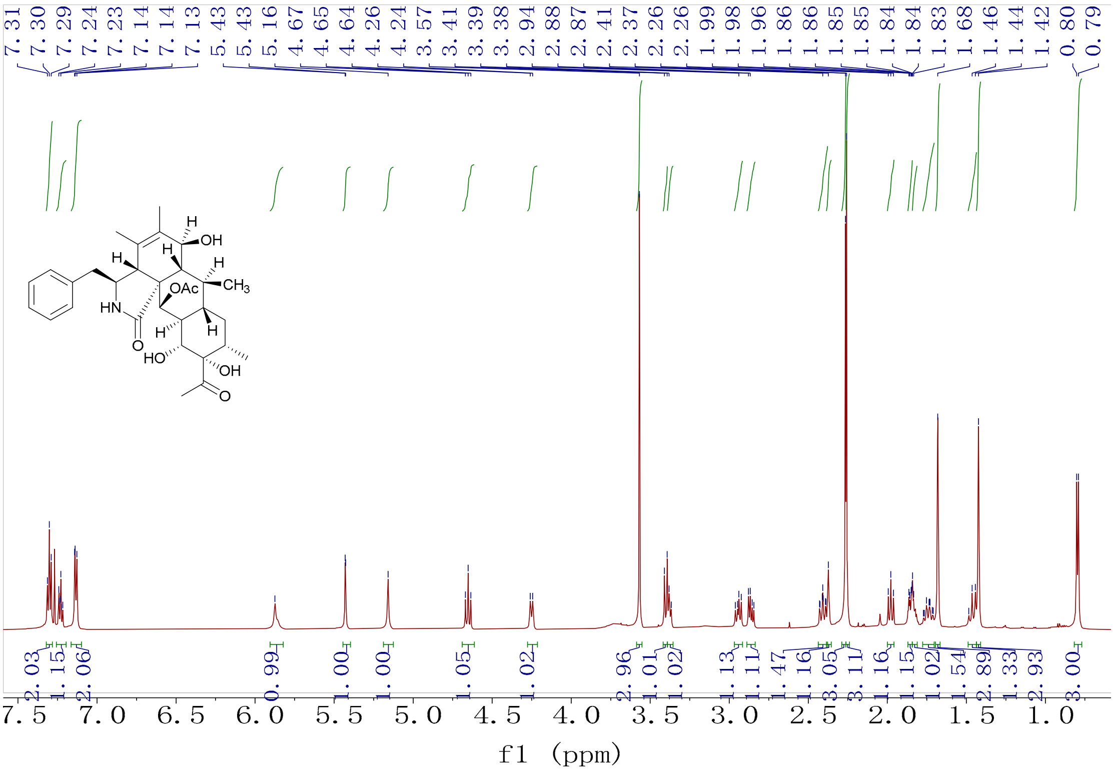


**Figure S1.** $^{1}$H NMR ($CDCl_3$, 600 MHz) of **1**.

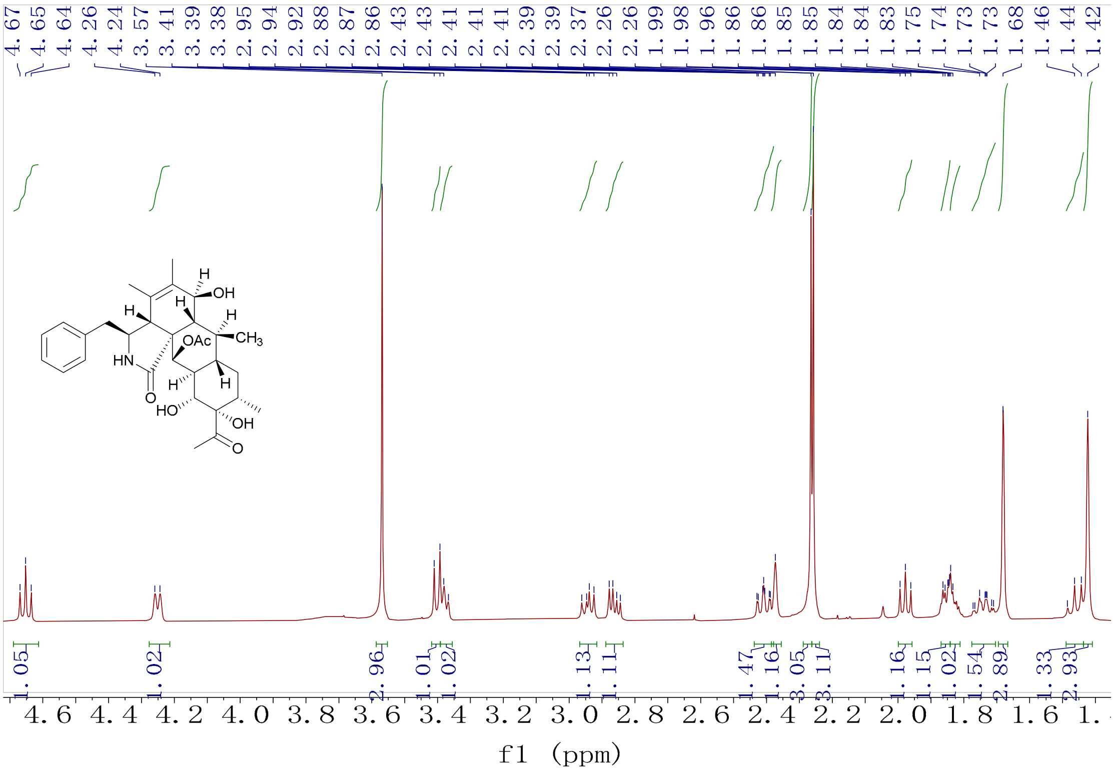


**Figure S2.** Partial $^{1}$H NMR ($CDCl_3$, 600 MHz) of **1**.

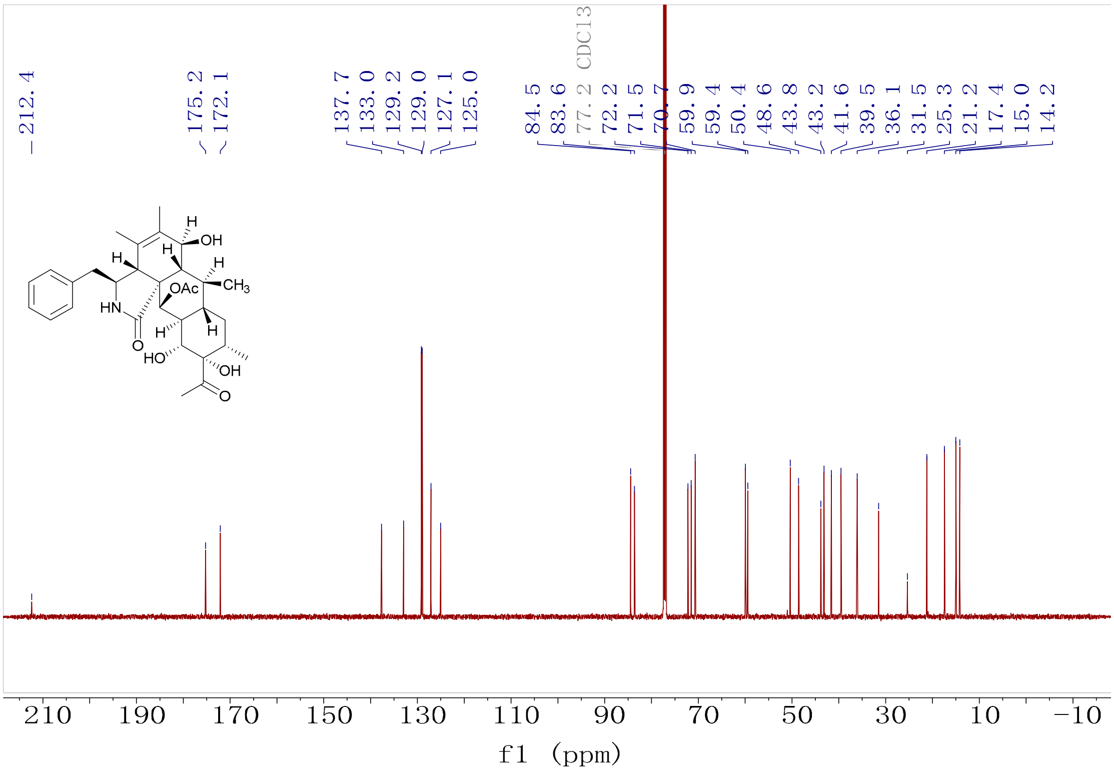


**Figure S3.** $^{13}$C NMR ($CDCl_3$, 150 MHz) of **1**.

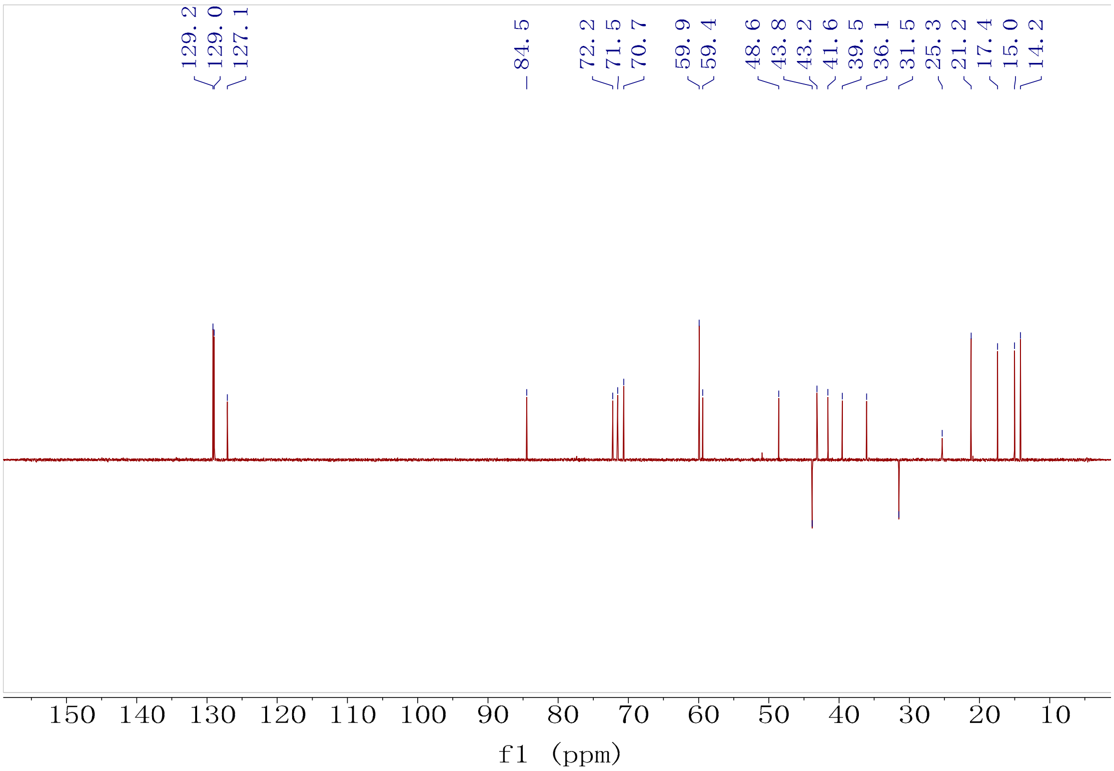


**Figure S4.** DEPT 135 spectra of **1**.

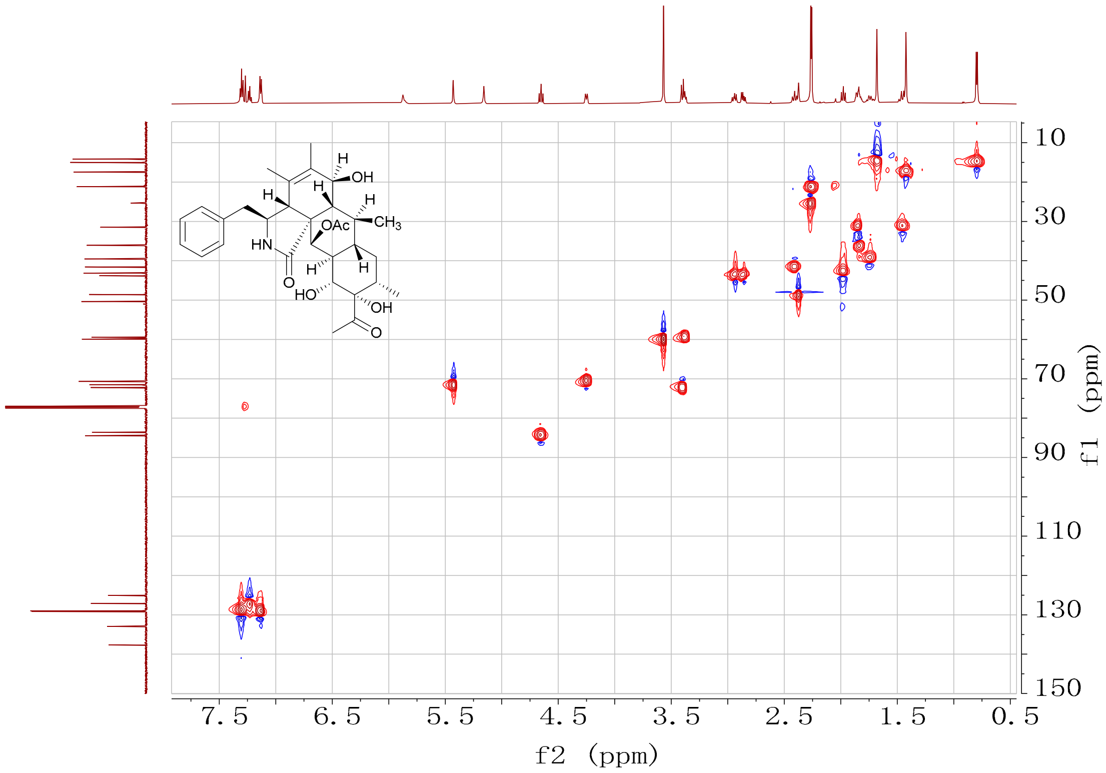


**Figure S5.** HSQC of **1.**

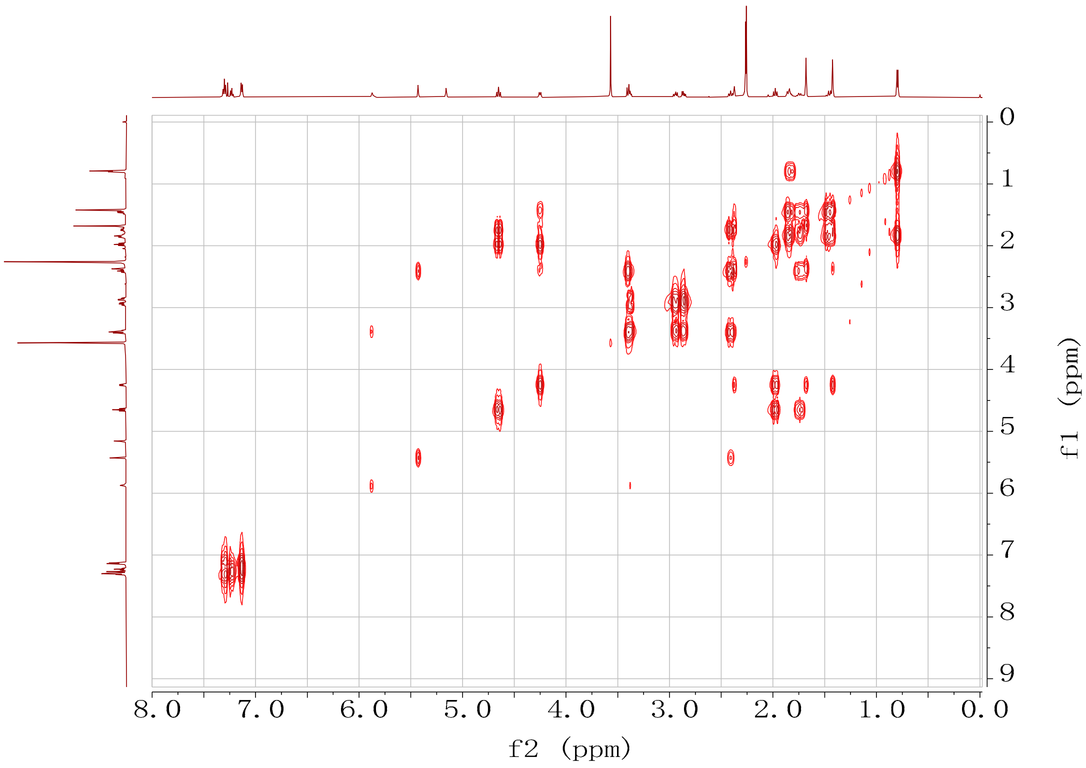


**Figure S6.** $^{1}$H−$^{1}$H COSY of **1**.

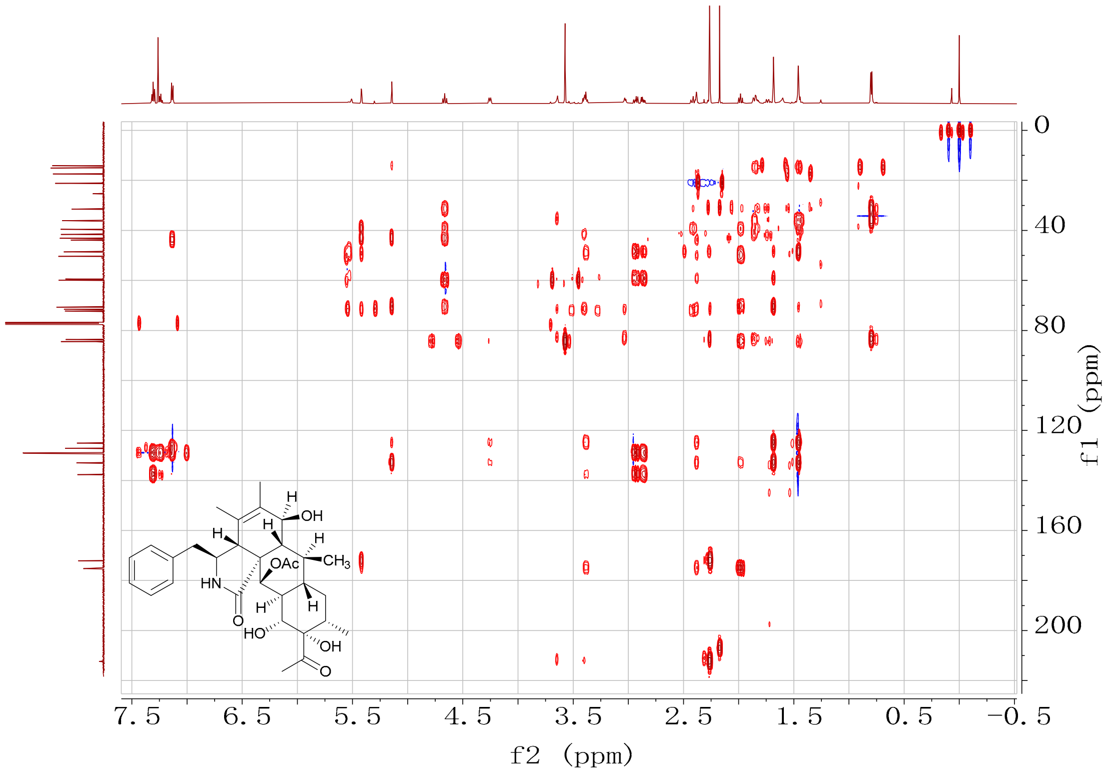


**Figure S7.** HMBC of **1.**

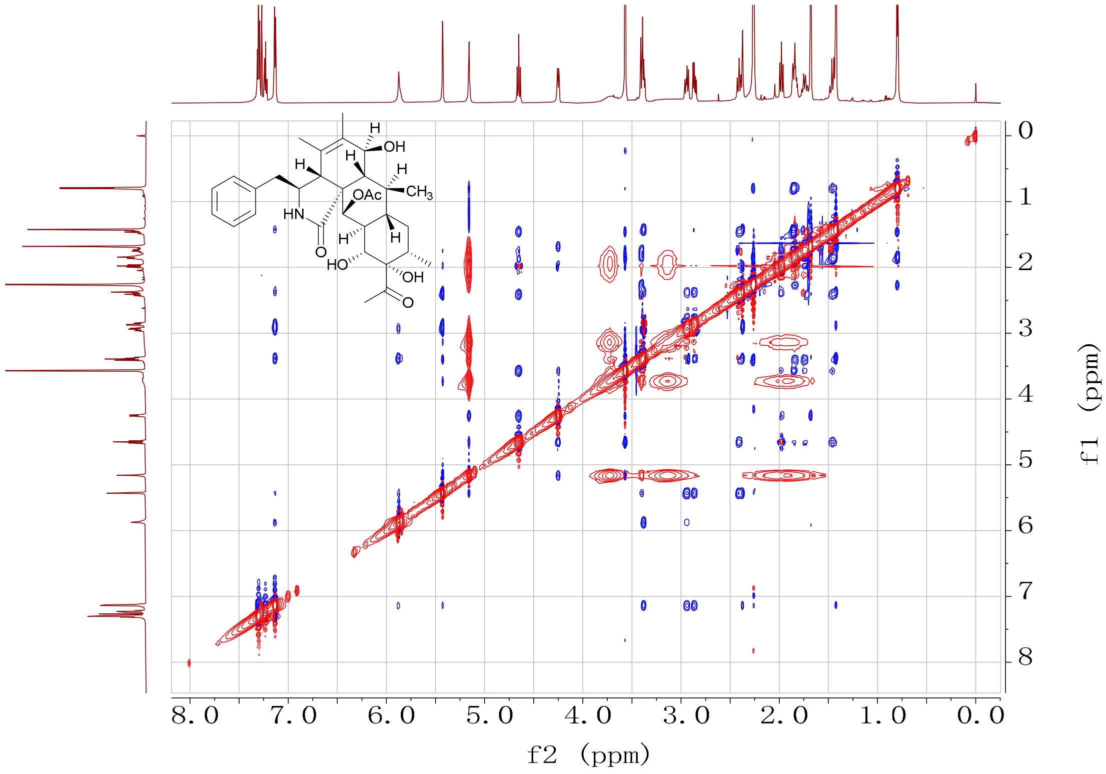


**Figure S8.** NOESY of **1**.

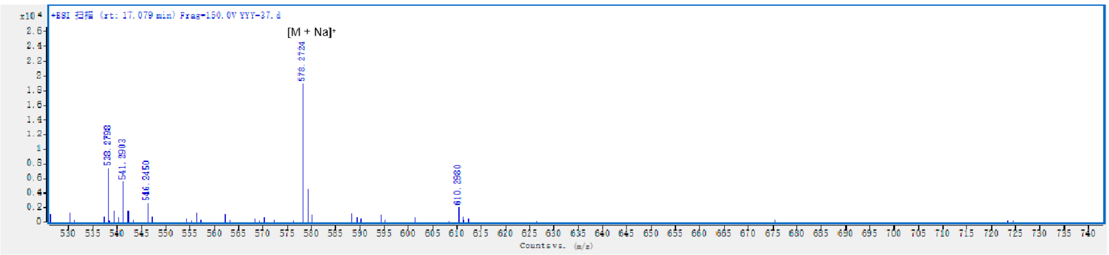


**Figure S9.** HRESIMS of **1.**

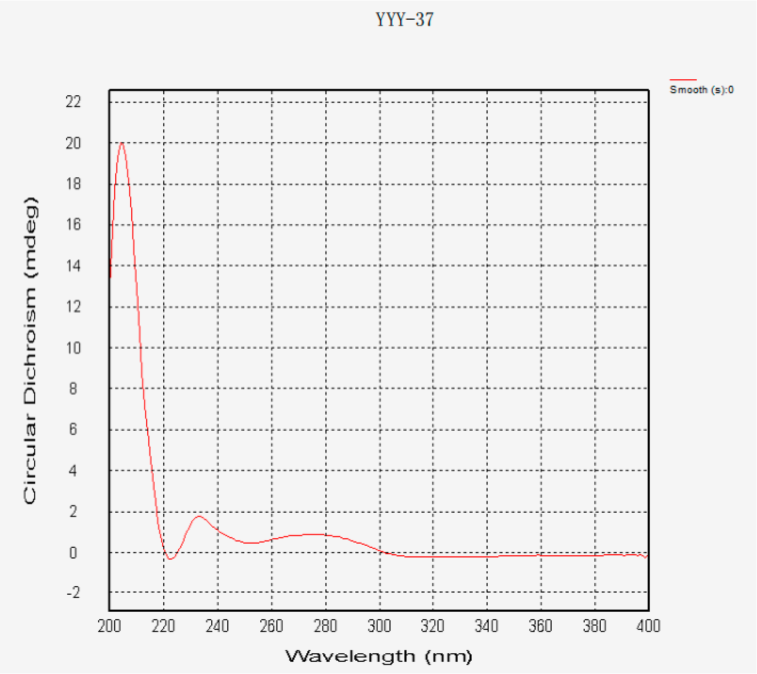


**Figure S10.** CD spectrum (acetonitrile) of **1.**

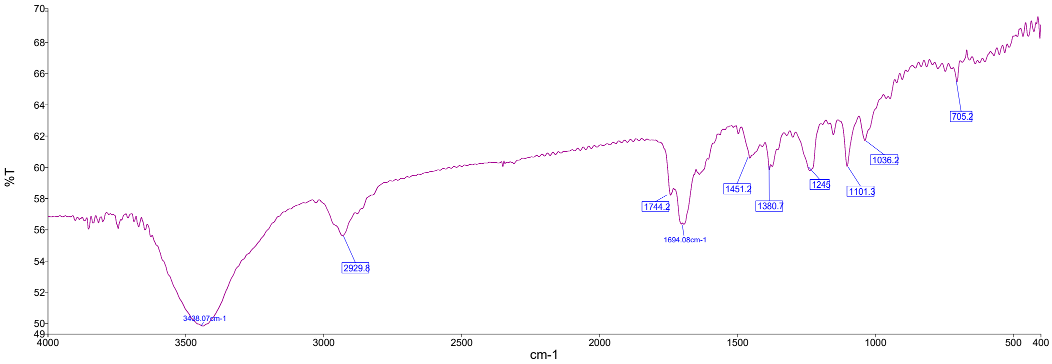


**Figure S11.** IR spectrum of **1.**

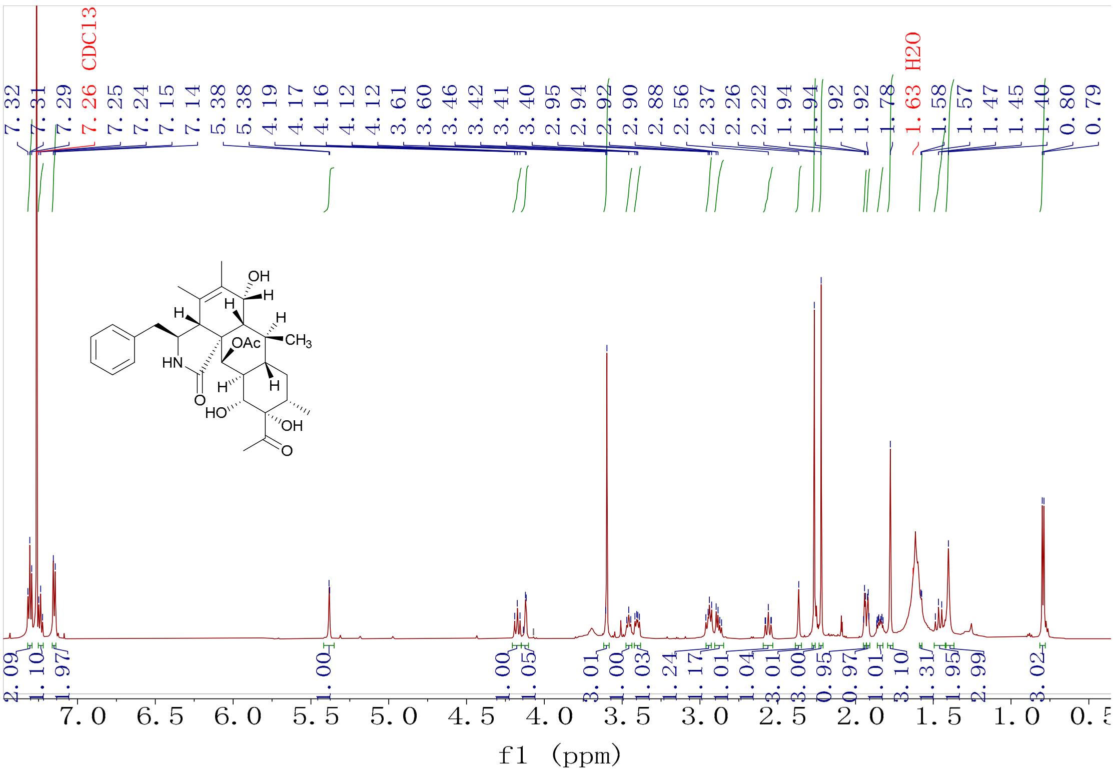


**Figure S12.** $^{1}H$ NMR ($CDCl_3$, 600 MHz) of **2**.

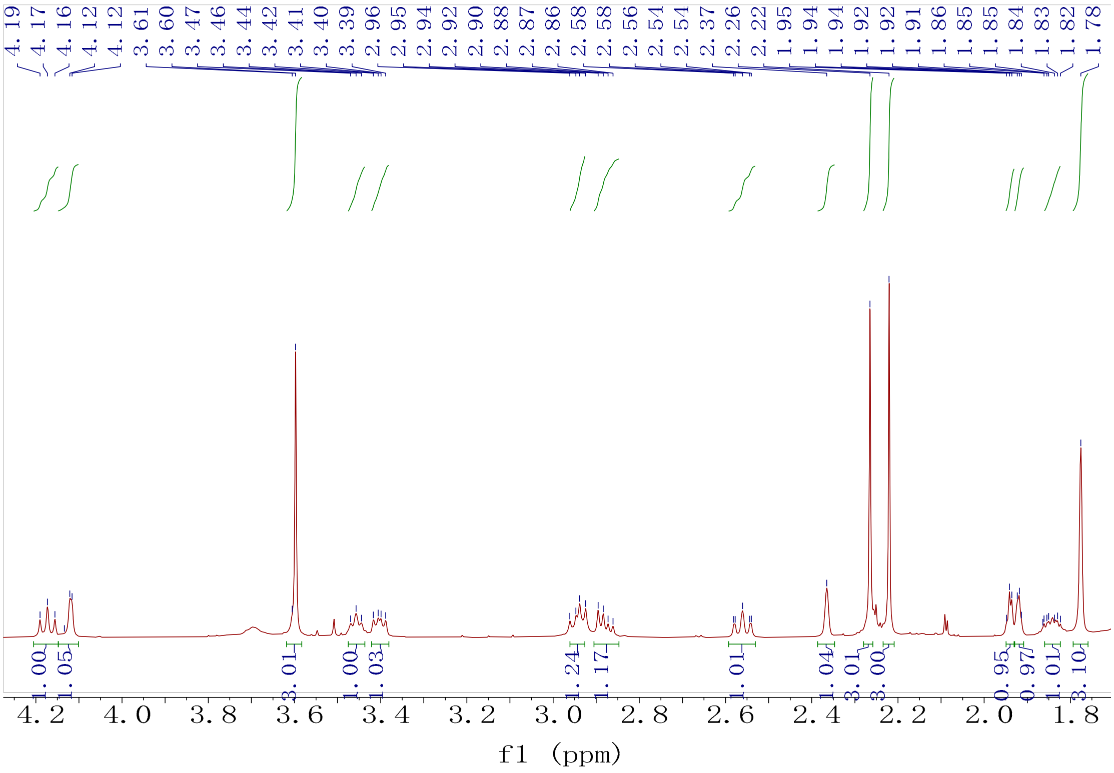


**Figure S13.** Partial $^{1}H$ NMR ($CDCl_3$, 600 MHz) of **2**.

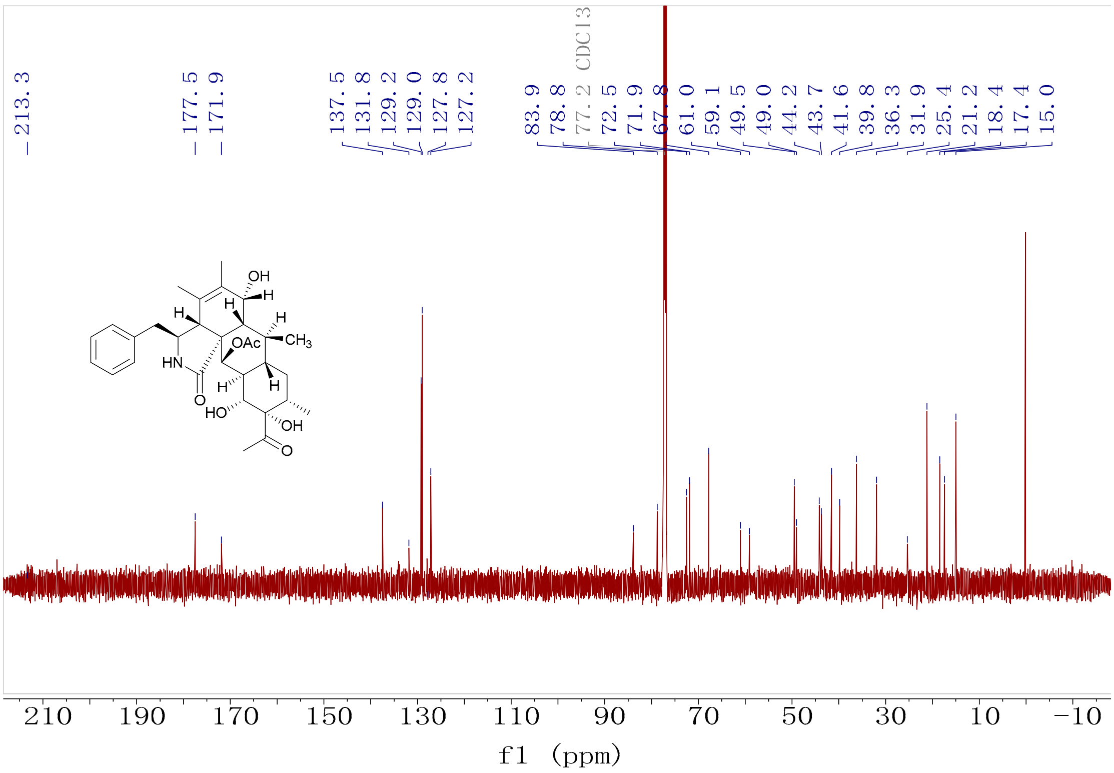


**Figure S14.** $^{13}C$ NMR ($CDCl_3$, 150 MHz) of **2**.

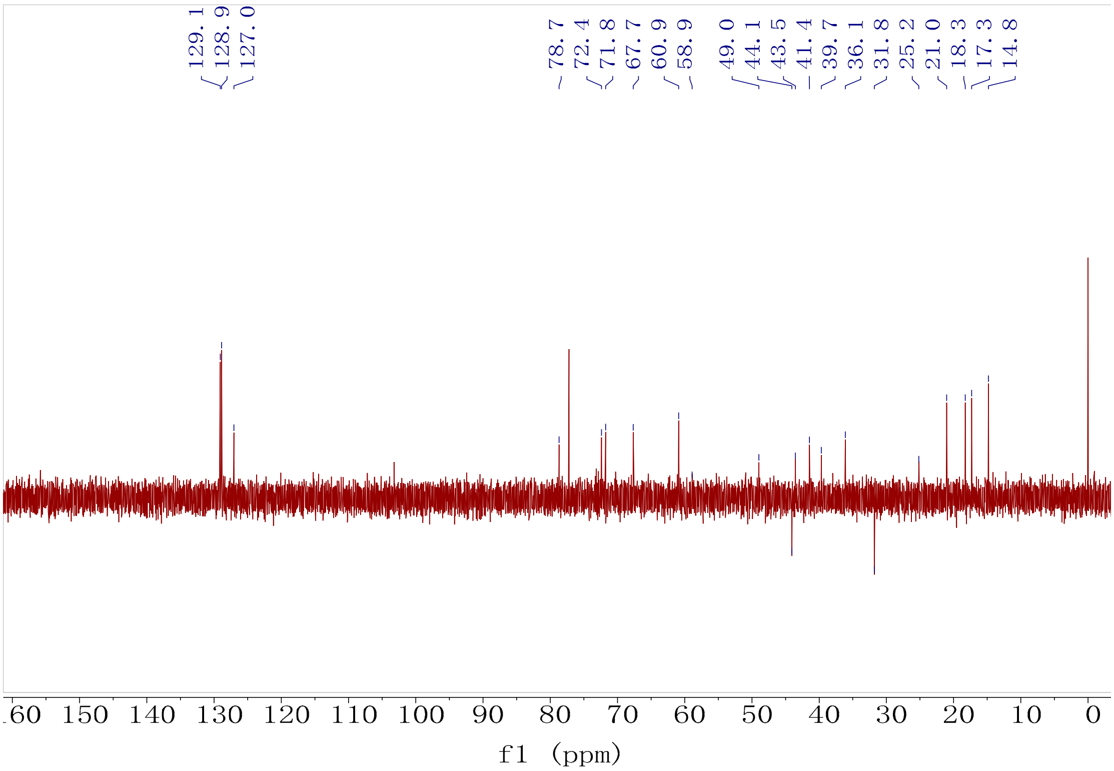


**Figure S15.** DEPT 135 spectra of **2**.

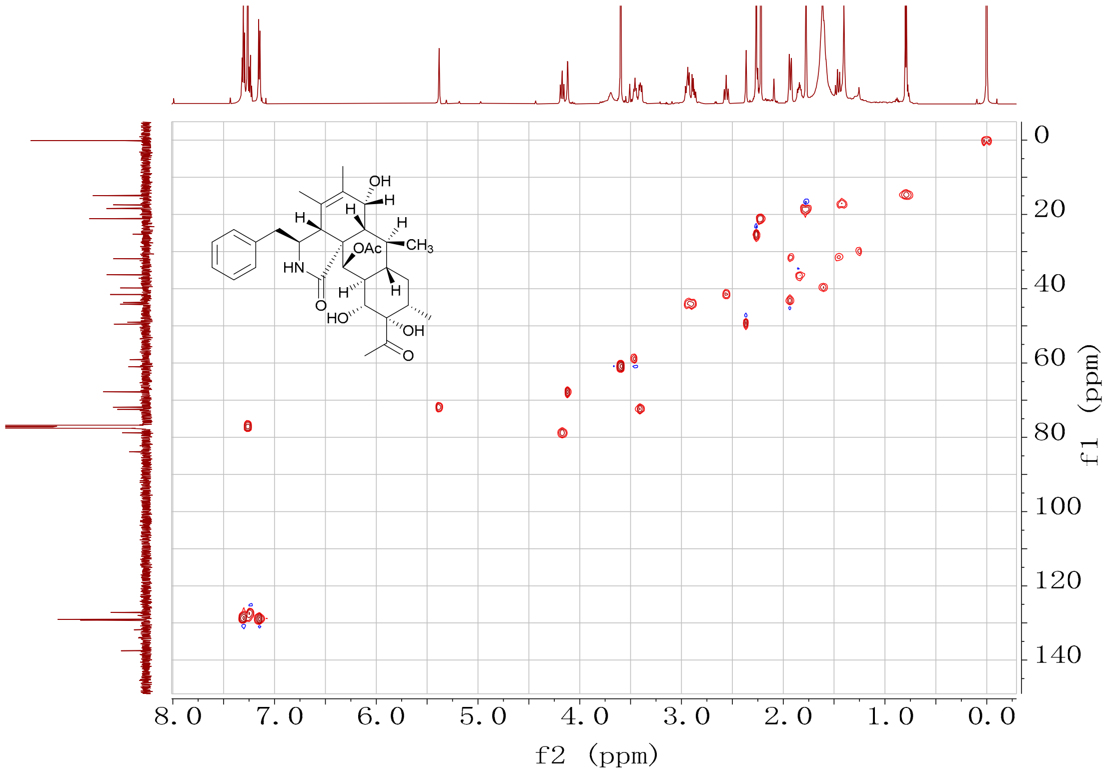


**Figure S16.** HSQC of **2**.

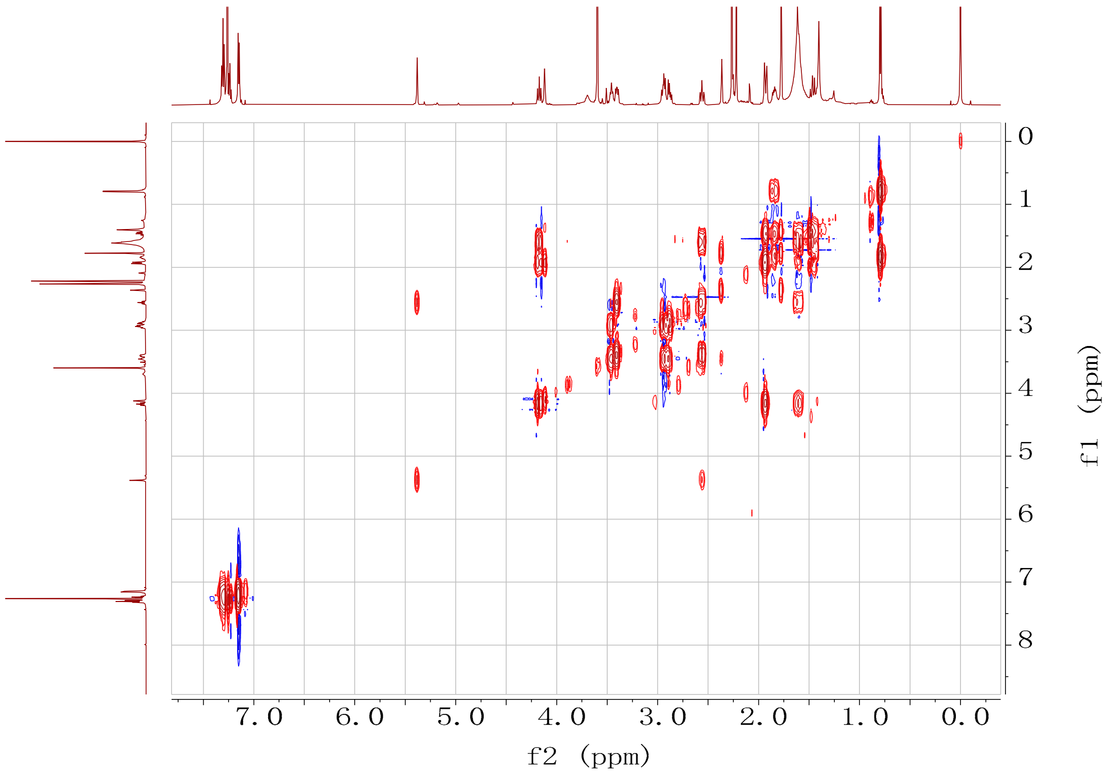


**Figure S17.** $^{1}$H–$^{1}$H COSY of **2**.

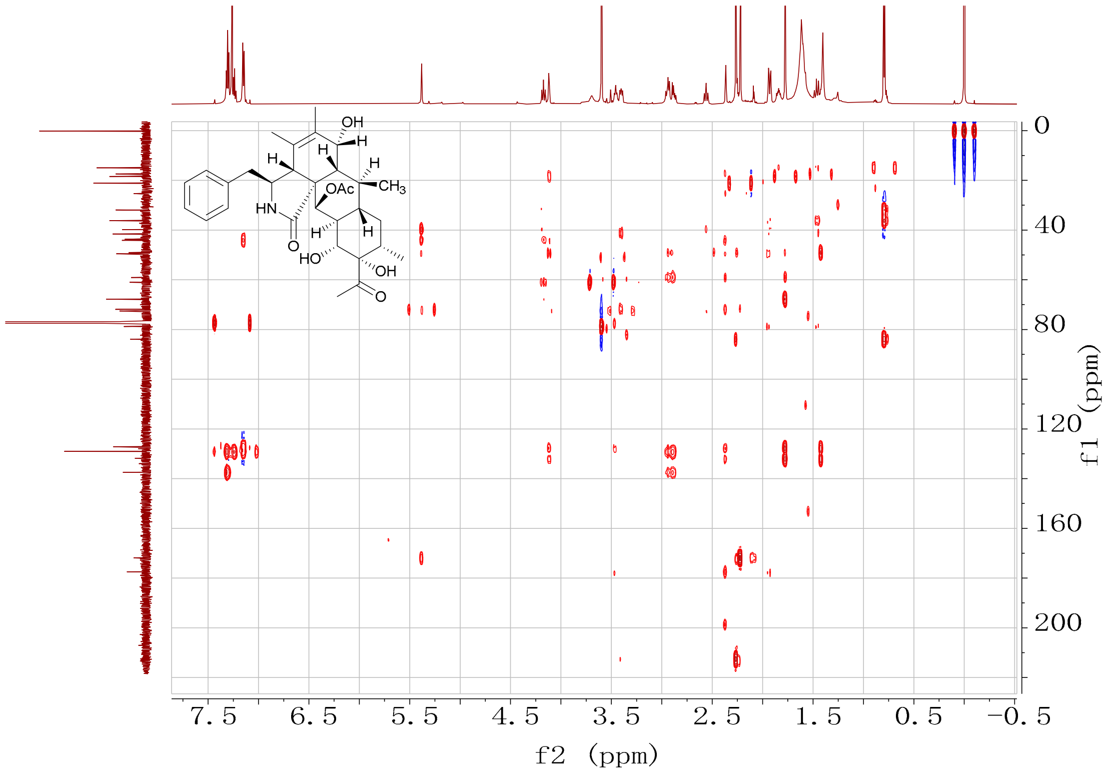


**Figure S18.** HMBC of **2**.

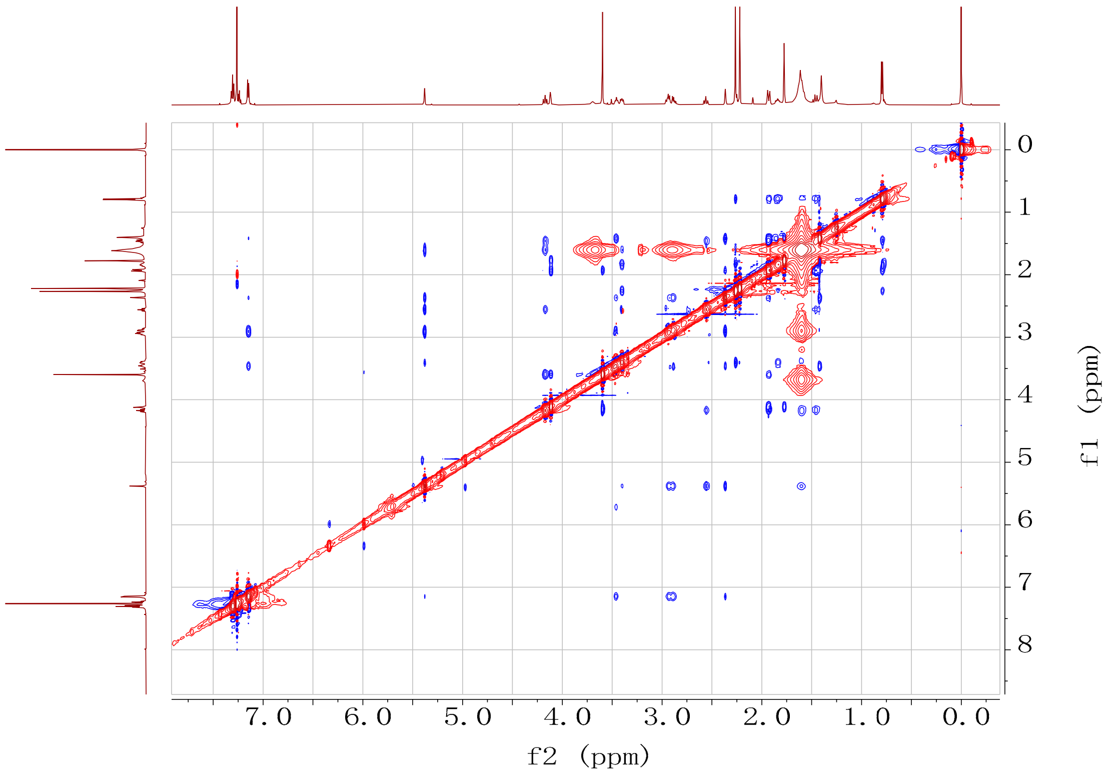


**Figure S19.** NOESY of **2**.

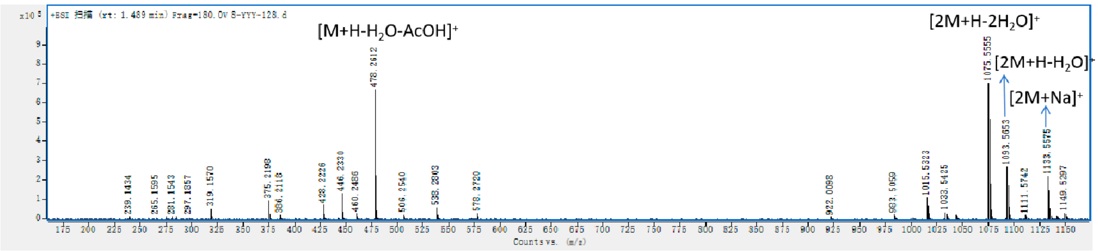


**Figure S20.** HRESIMS of **2**.

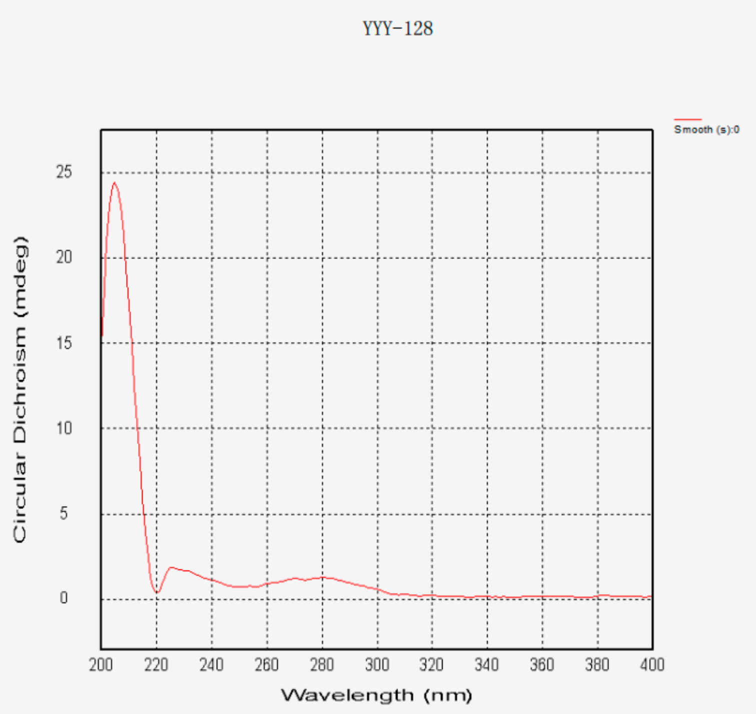


**Figure S21.** CD spectrum (acetonitrile) of **2.**

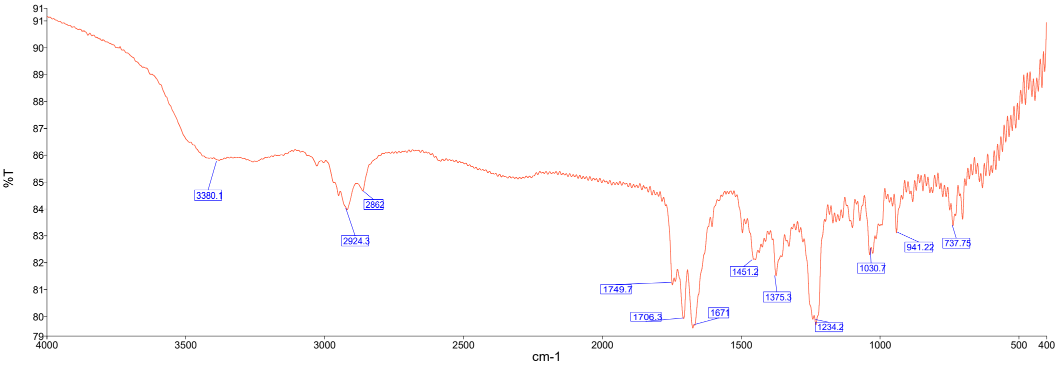


**Figure S22.** IR spectrum of **2**.

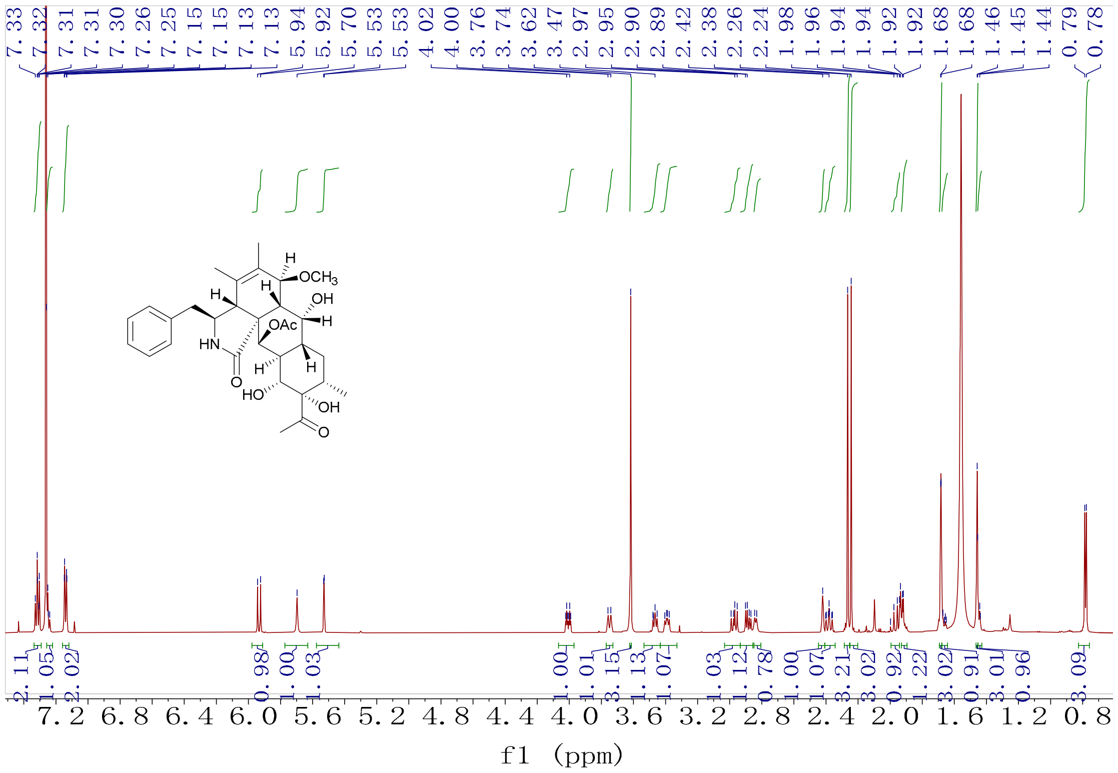

**Figure S23.** $^{1}H$ NMR ($CDCl_3$, 600 MHz) of **3**.

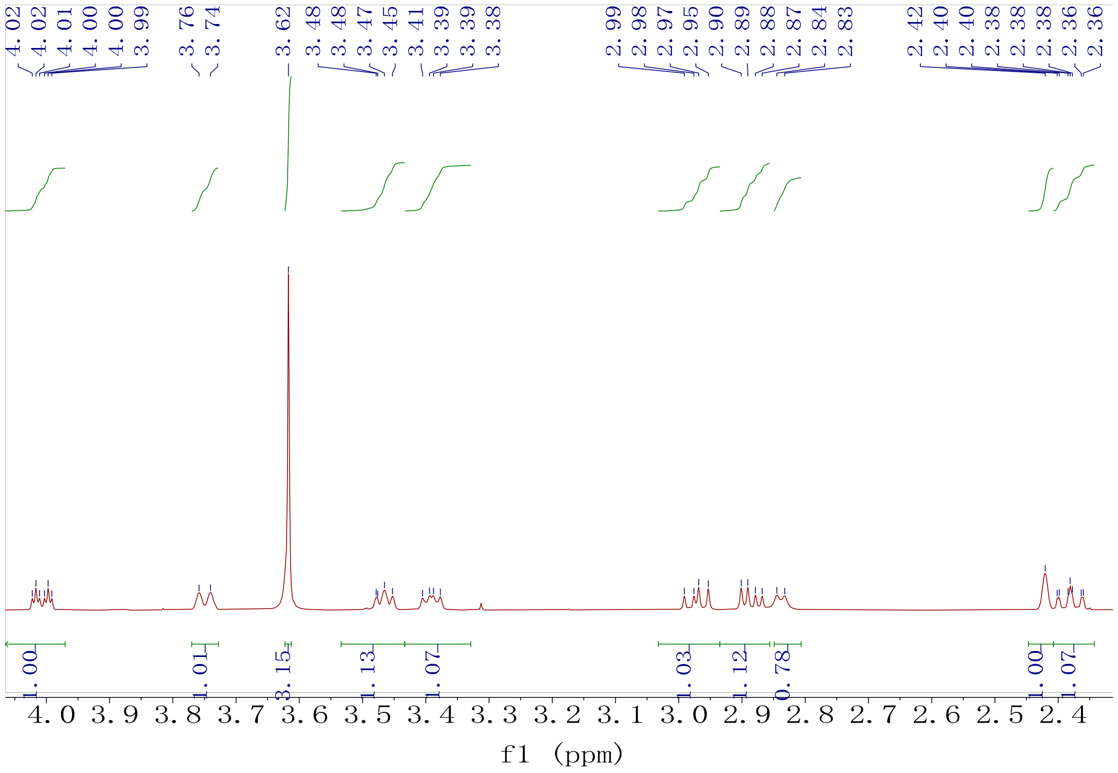

**Figure S24.** Partial $^{1}H$ NMR ($CDCl_3$, 600 MHz) of **3**.

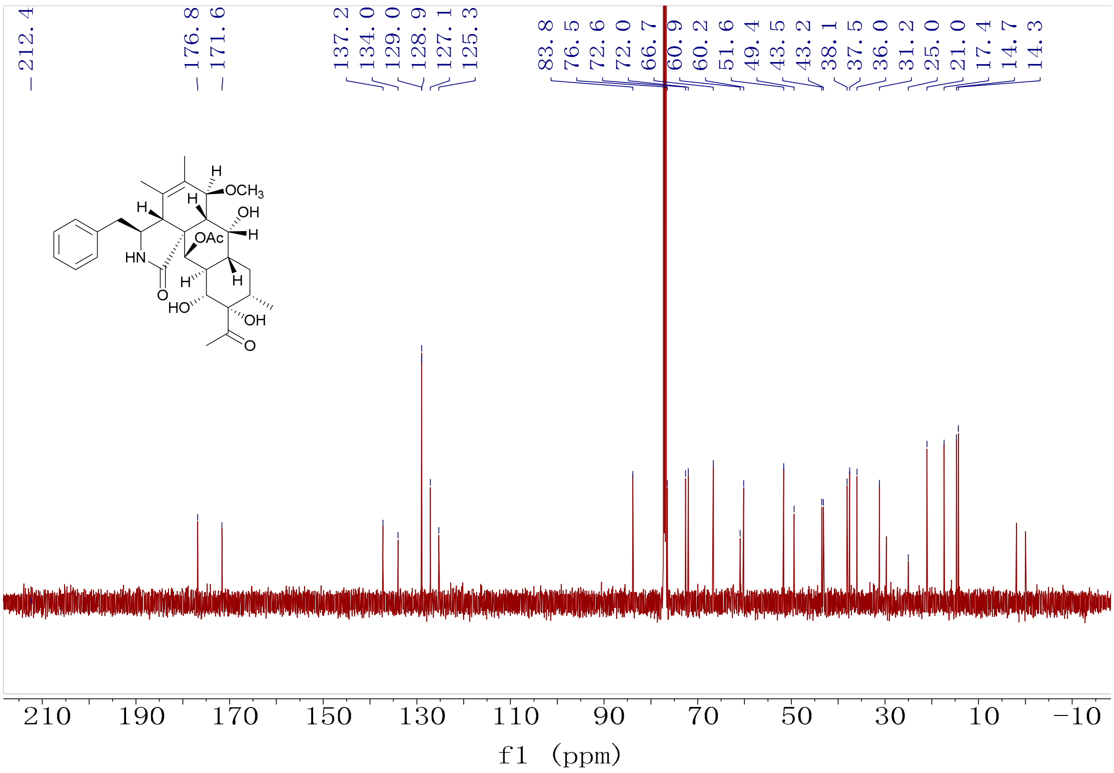


**Figure S25.** $^{13}C$ NMR ($CDCl_3$, 150 MHz) of **3**.

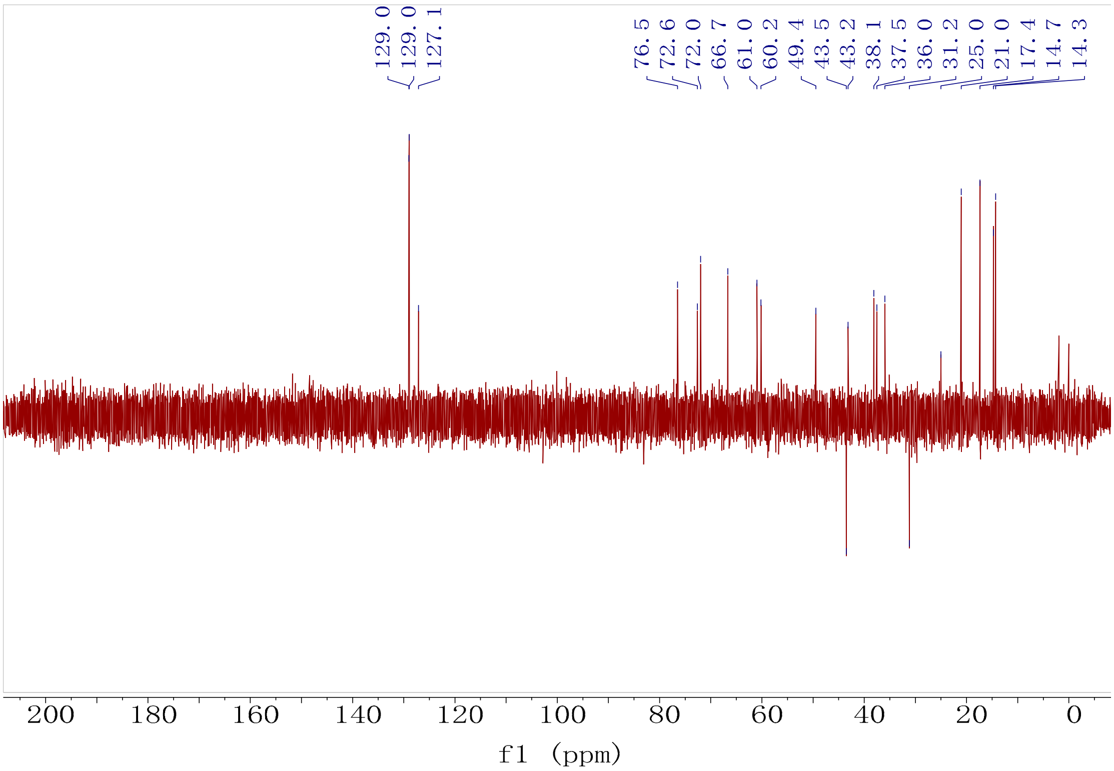


**Figure S26.** DEPT 135 spectra of **3**.

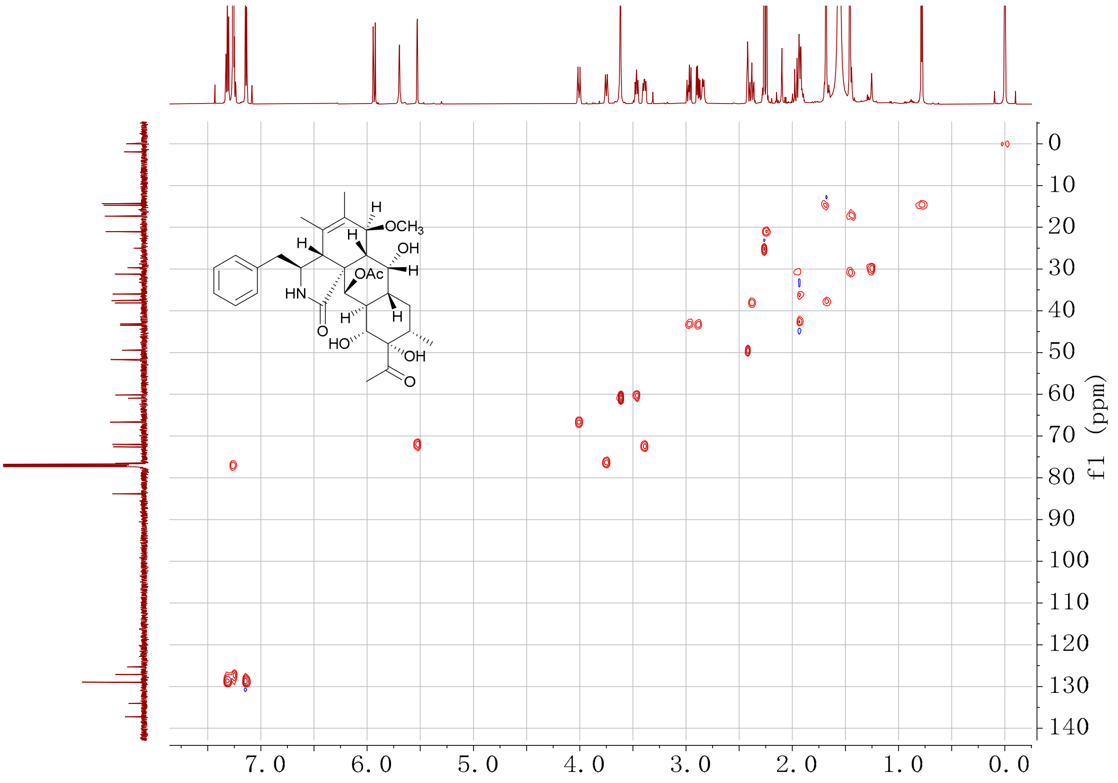

**Figure S27.** HSQC of **3**.

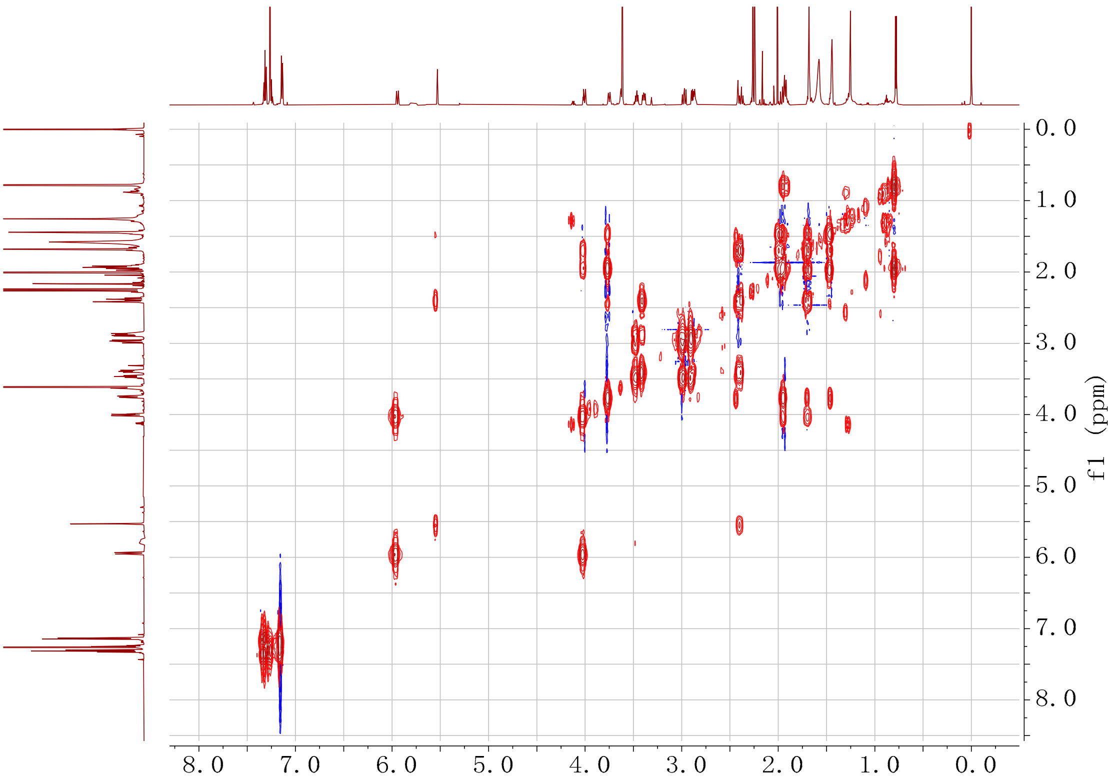

**Figure S28.** $^{1}H-^{1}H$ COSY of **3**.

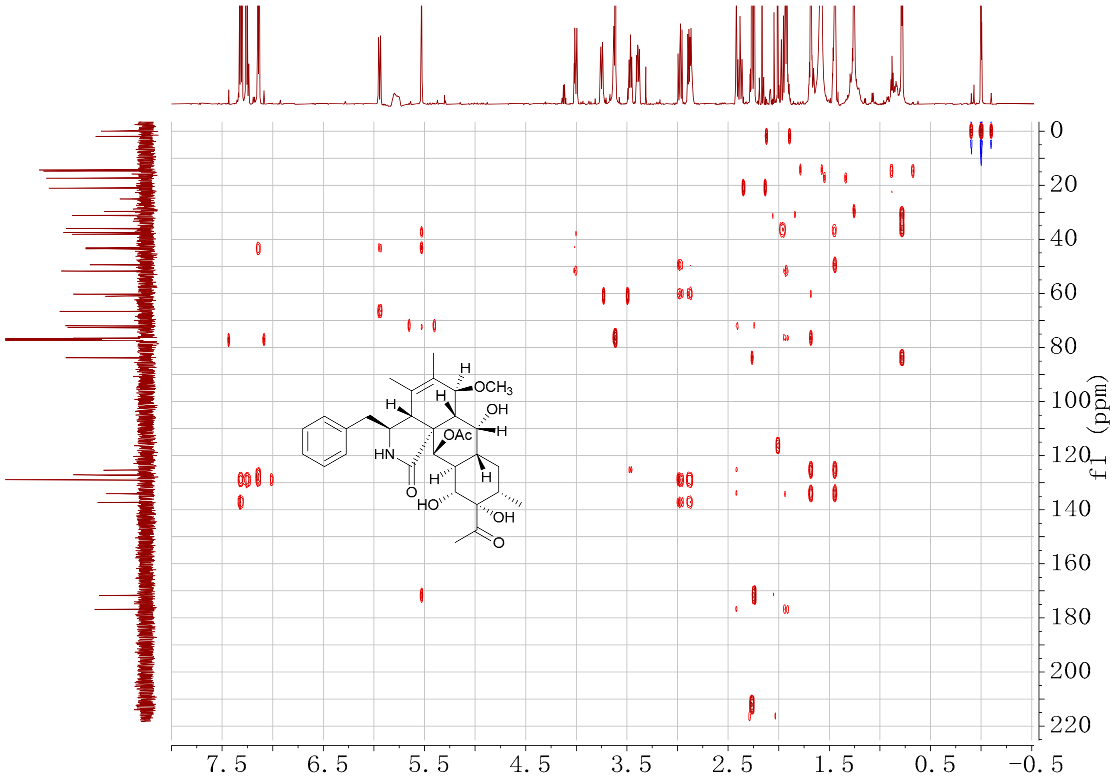


**Figure S29.** HMBC of **3**.

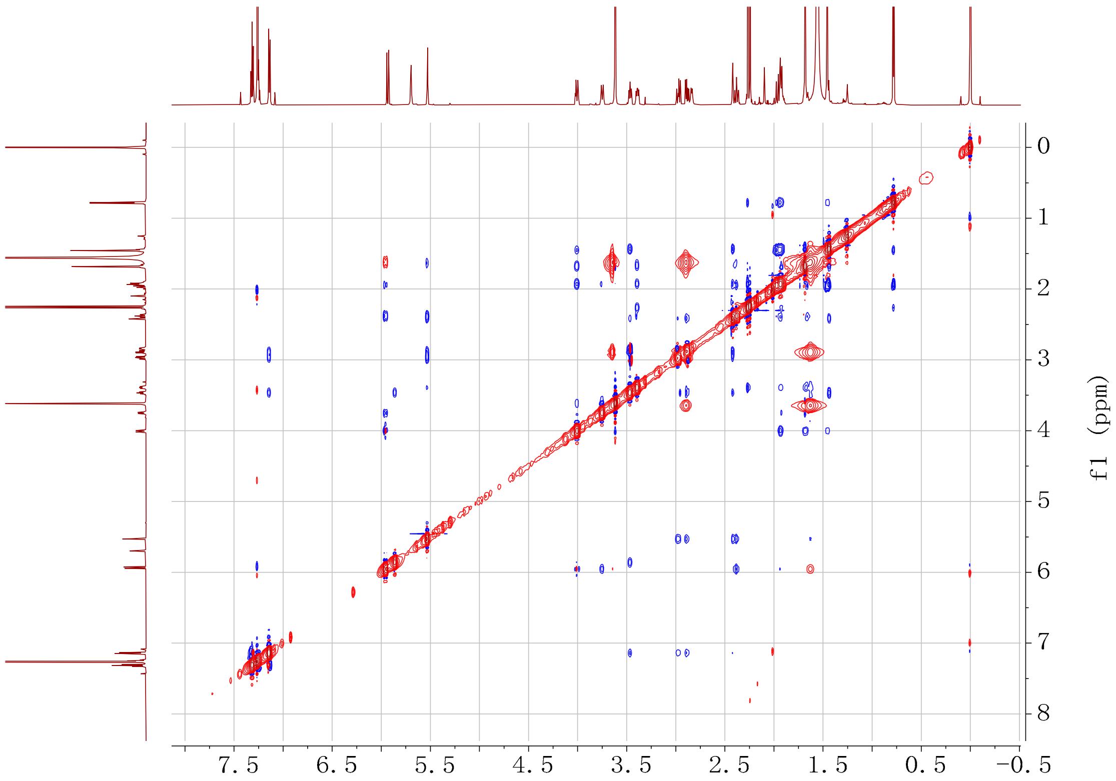


**Figure S30.** NOESY of **3**.

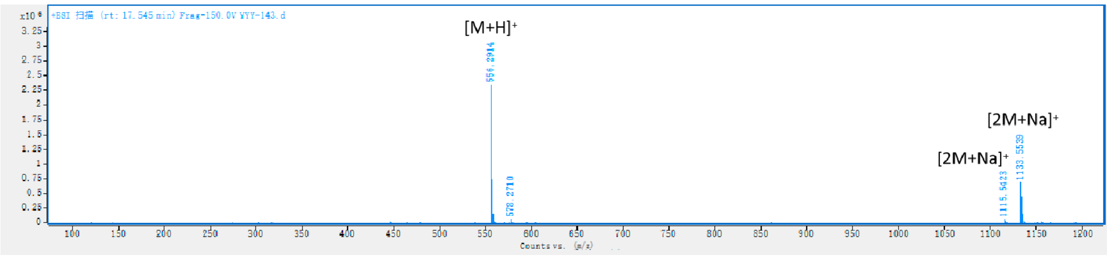


**Figure S31.** HRESIMS of **3**.

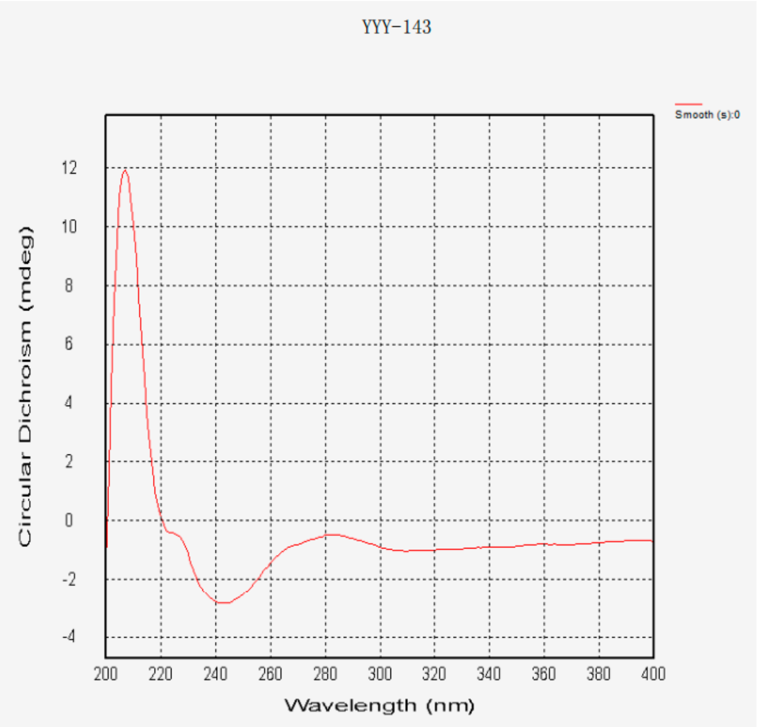


**Figure S32.** CD spectrum (acetonitrile) of **3.**

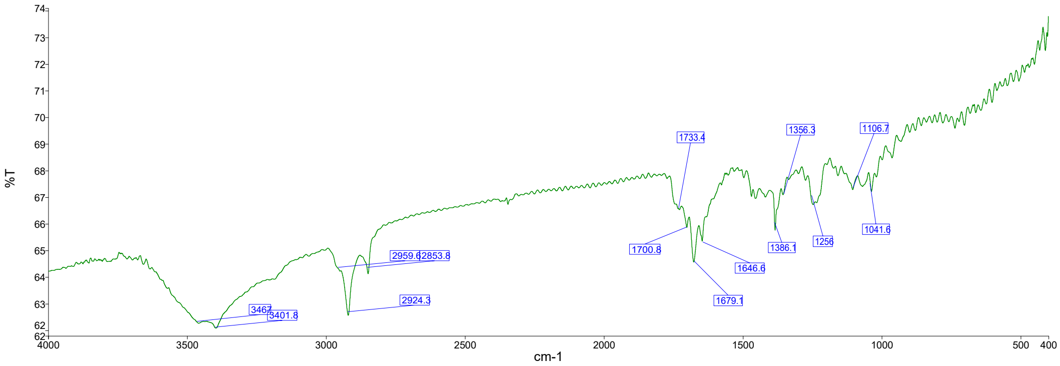


**Figure S33.** IR spectrum of **3**.

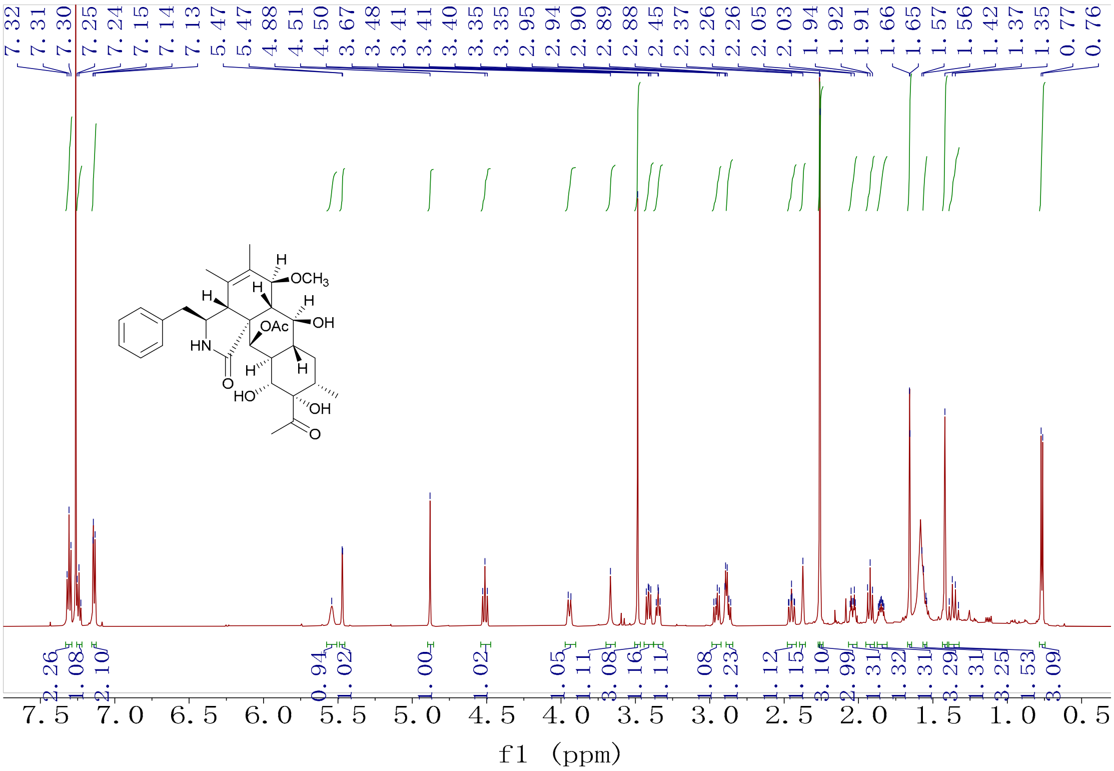

**Figure S34.** $^{1}H$ NMR ($CDCl_3$, 500 MHz) of **4**.

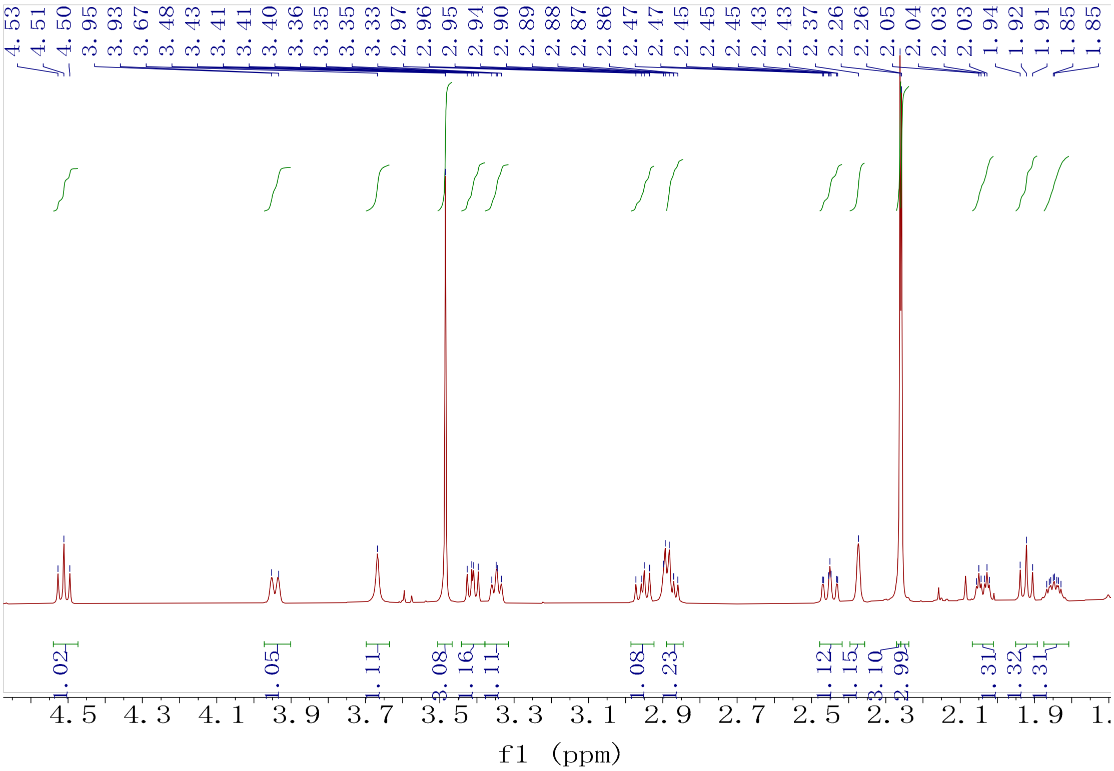

**Figure S35.** Partial $^{1}H$ NMR ($CDCl_3$, 600 MHz) of **4**.

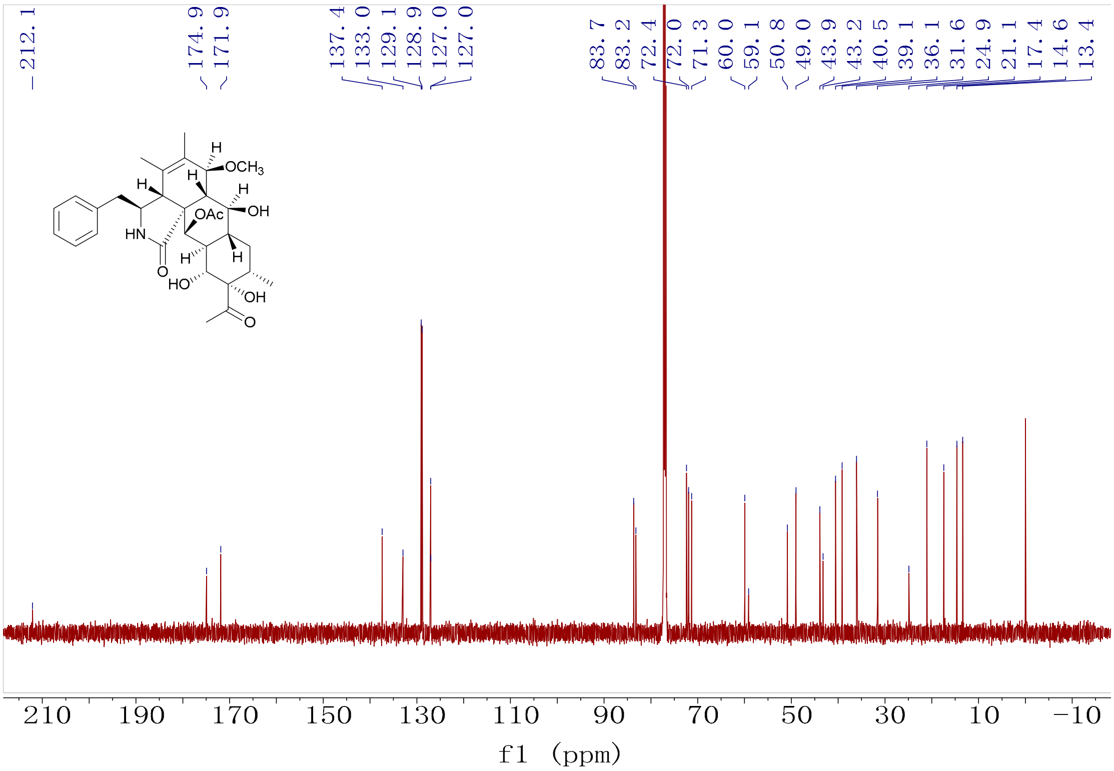


**Figure S36.** $^{13}$C NMR ($CDCl_3$, 150 MHz) of **4**.

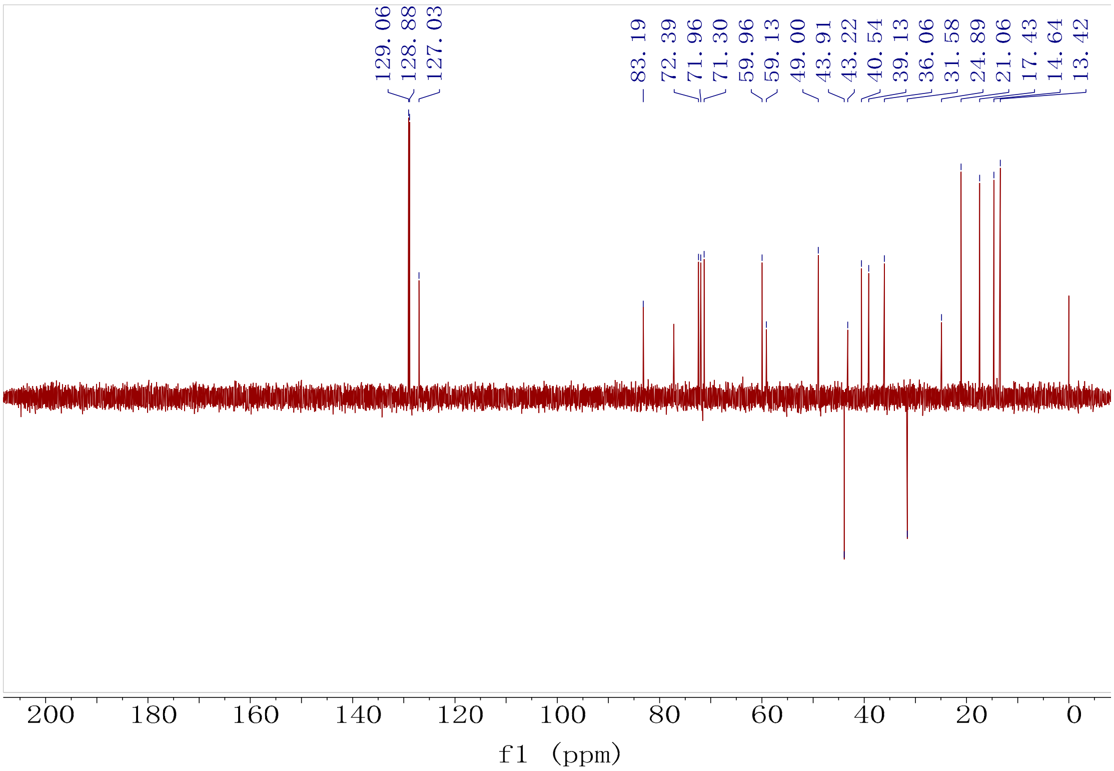


**Figure S37.** DEPT 135 spectra of **4**.

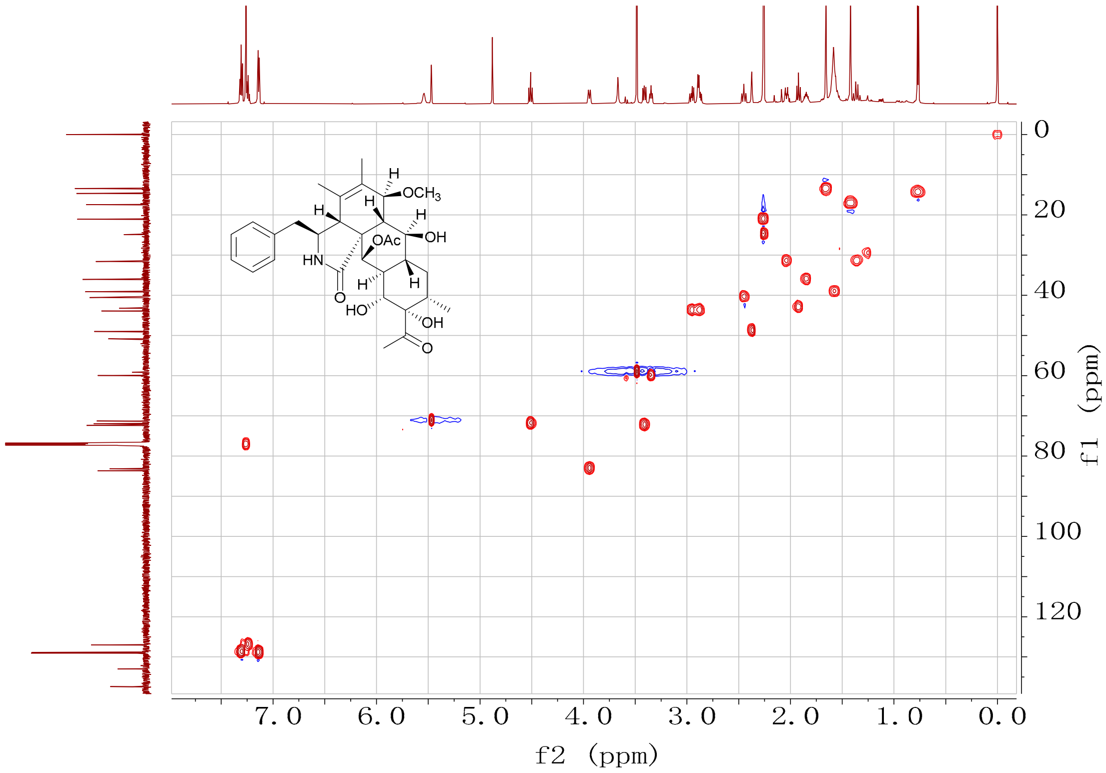


**Figure S38.** HSQC of **4**.

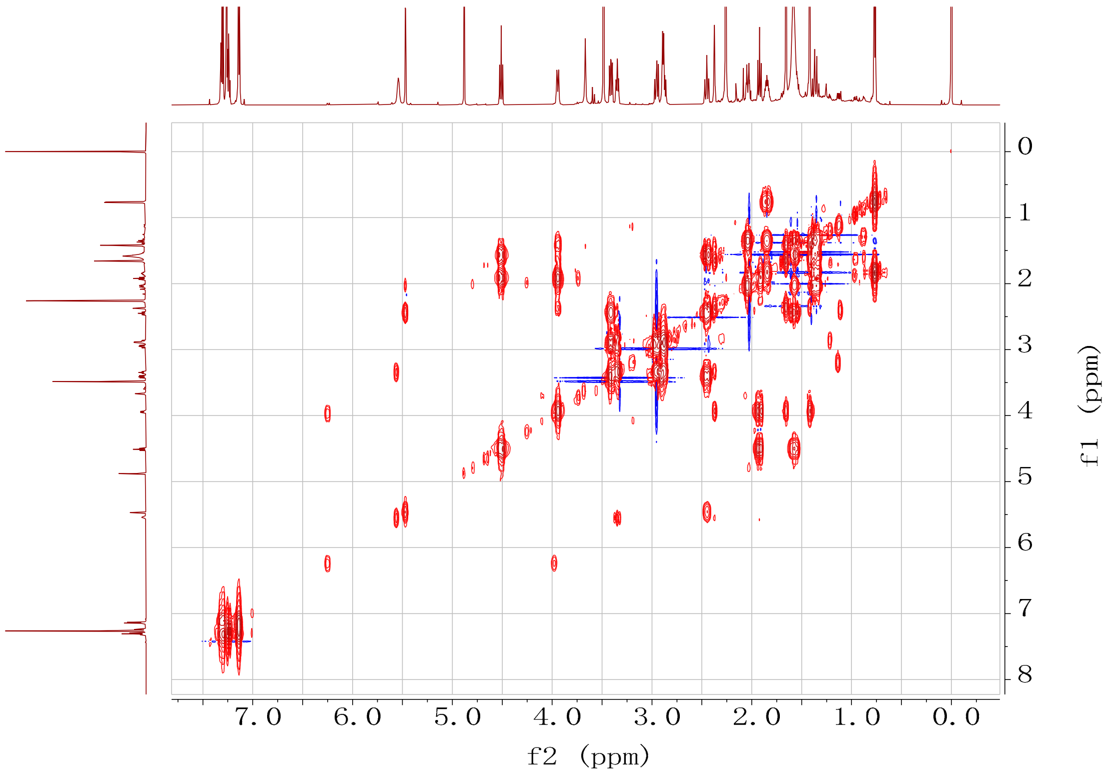


**Figure S39.** $^{1}H-^{1}H$ COSY of **4**.

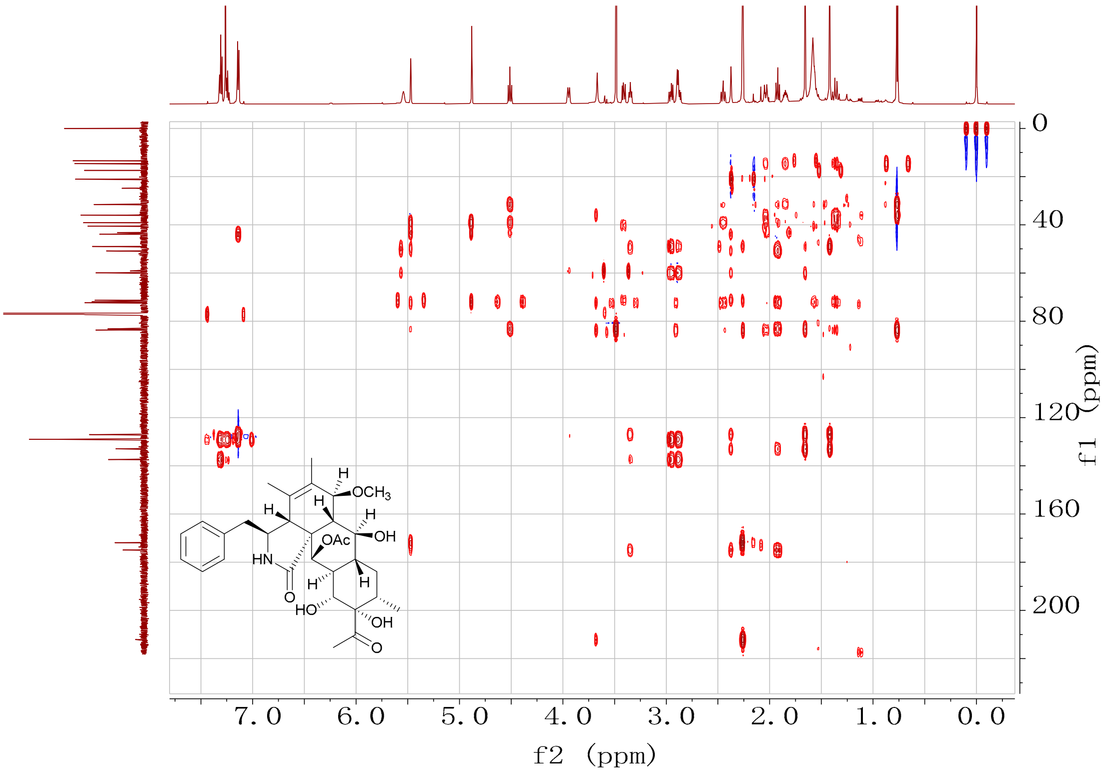


**Figure S40.** HMBC of **4**.

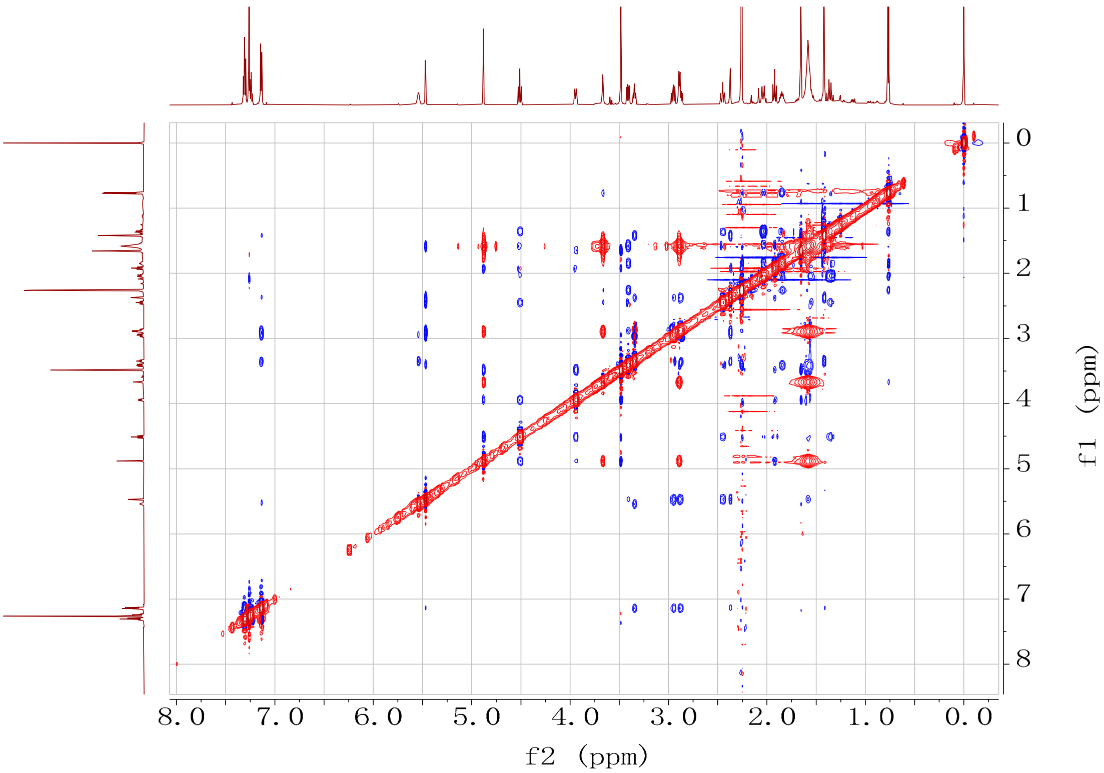


**Figure S41.** NOESY of **4**.

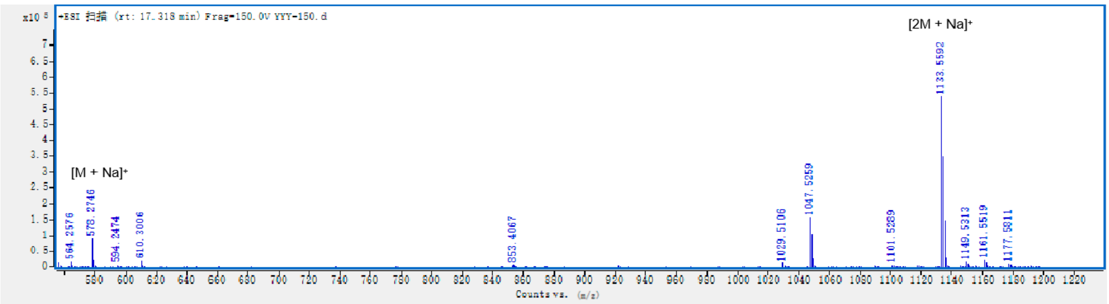


**Figure S42.** HRESIMS of **4**.

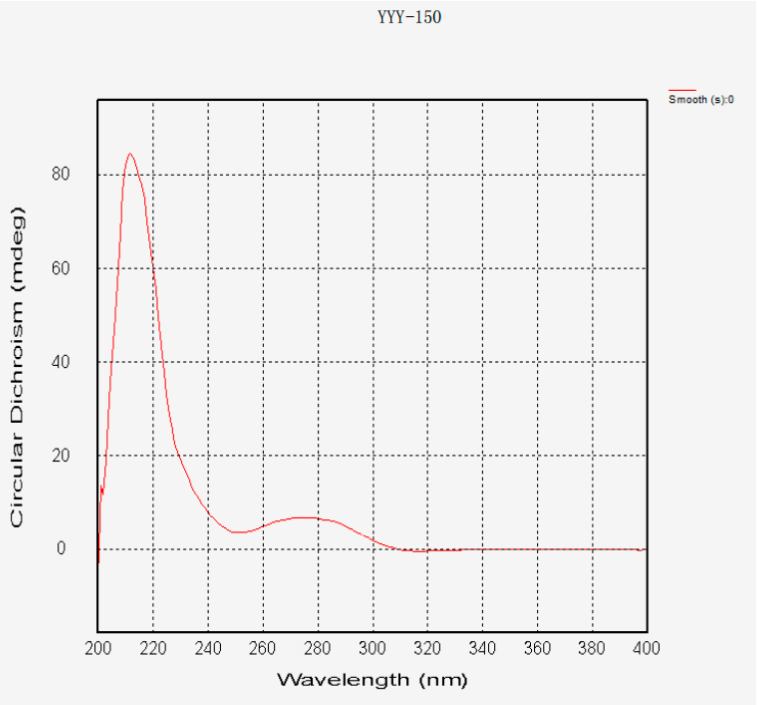


**Figure S43.** CD spectrum (methanol) of **4.**

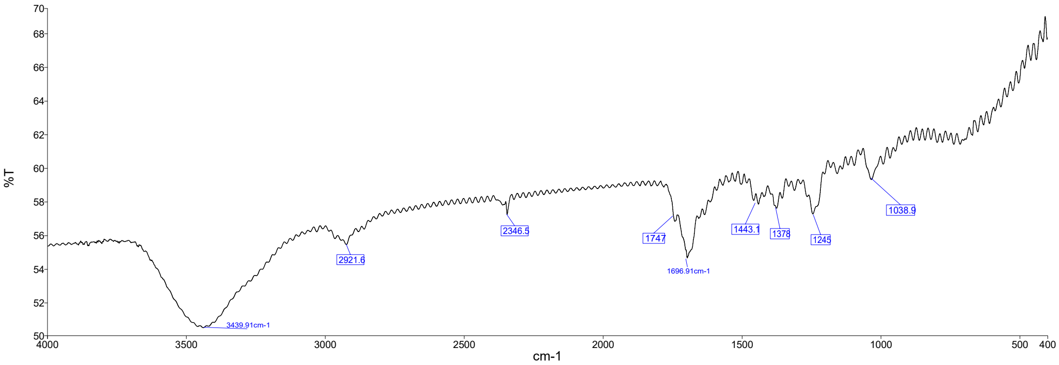


**Figure S44.** IR spectrum of **4.**

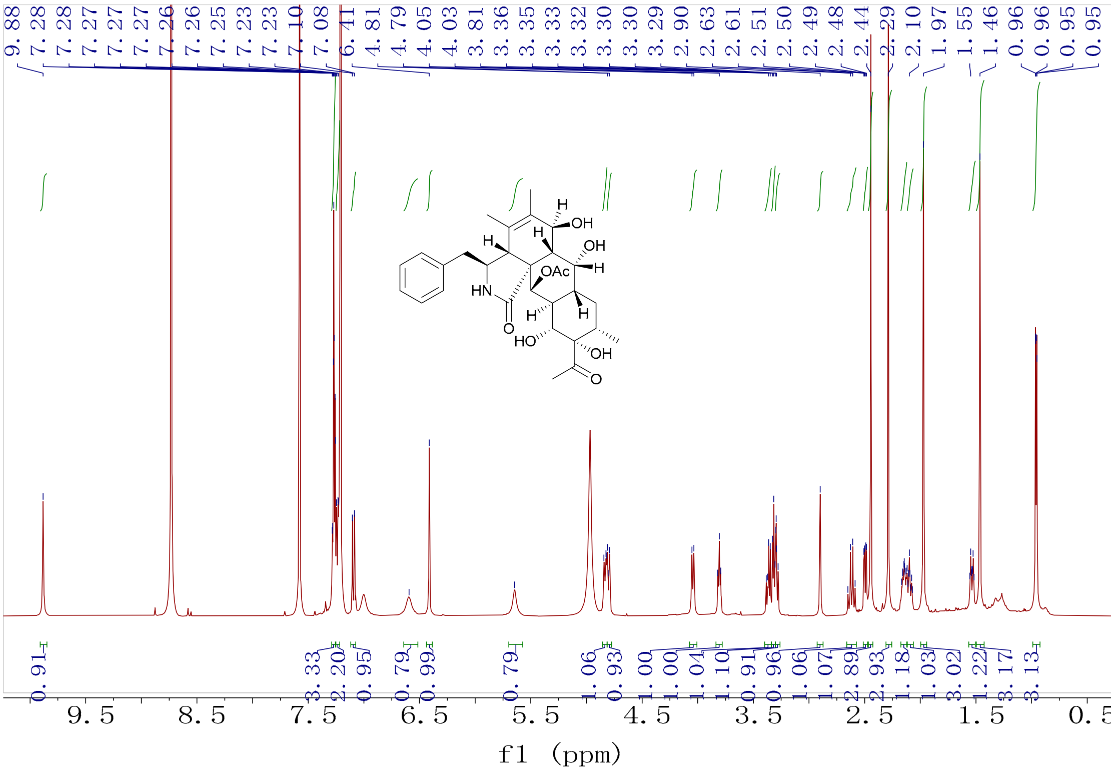


**Figure S45.** $^{1}$H NMR (pyridine-$d_5$., 600 MHz) of **5**.

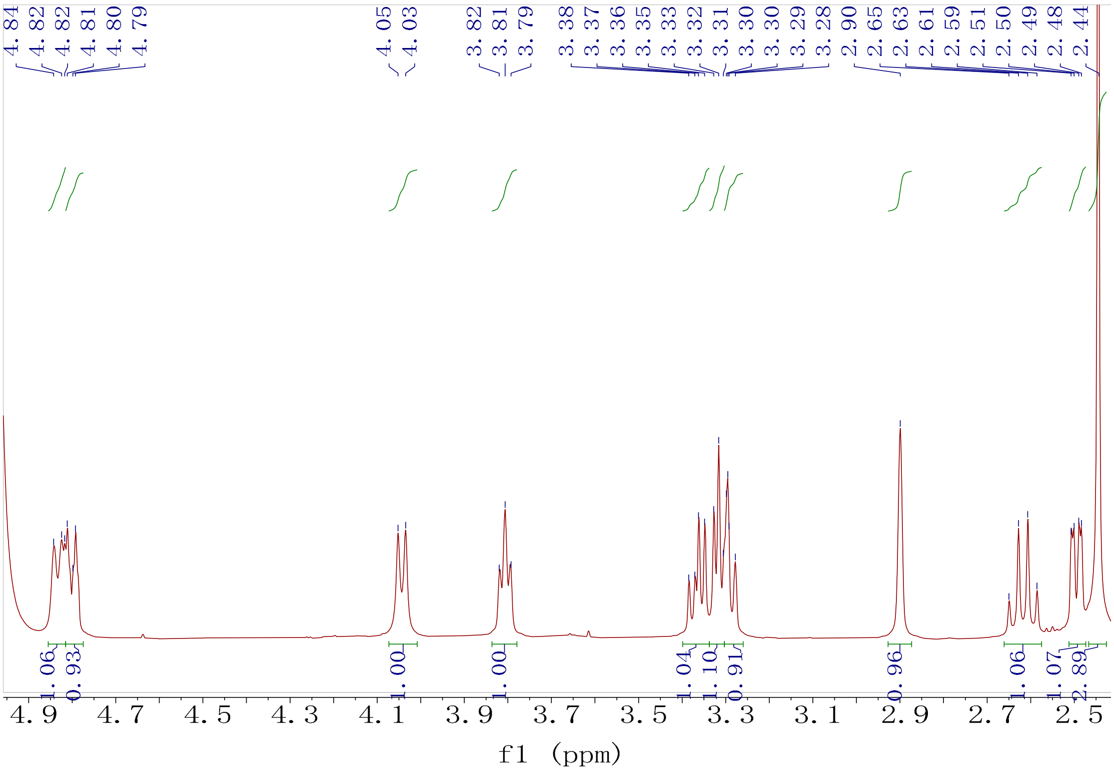


**Figure S46.** Partial $^{1}$H NMR ($CDCl_3$, 600 MHz) of **5**.

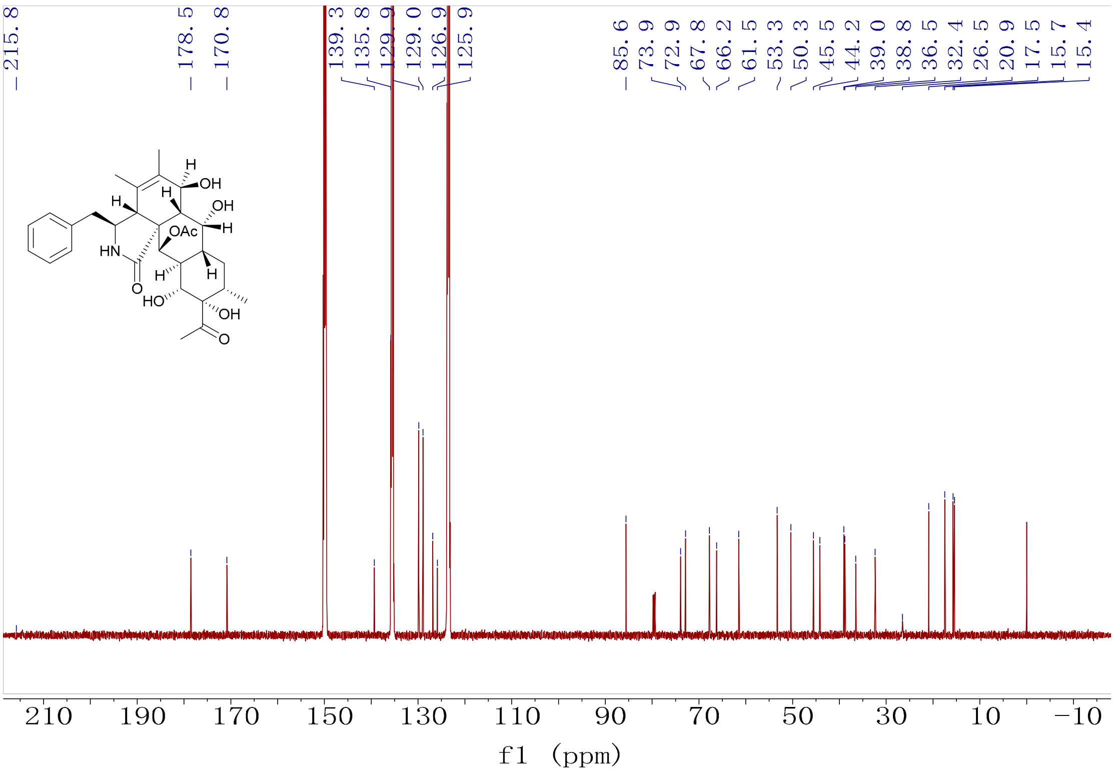


**Figure S47.** $^{13}C$ NMR (pyridine-*d*5., 150 MHz) of **5**.

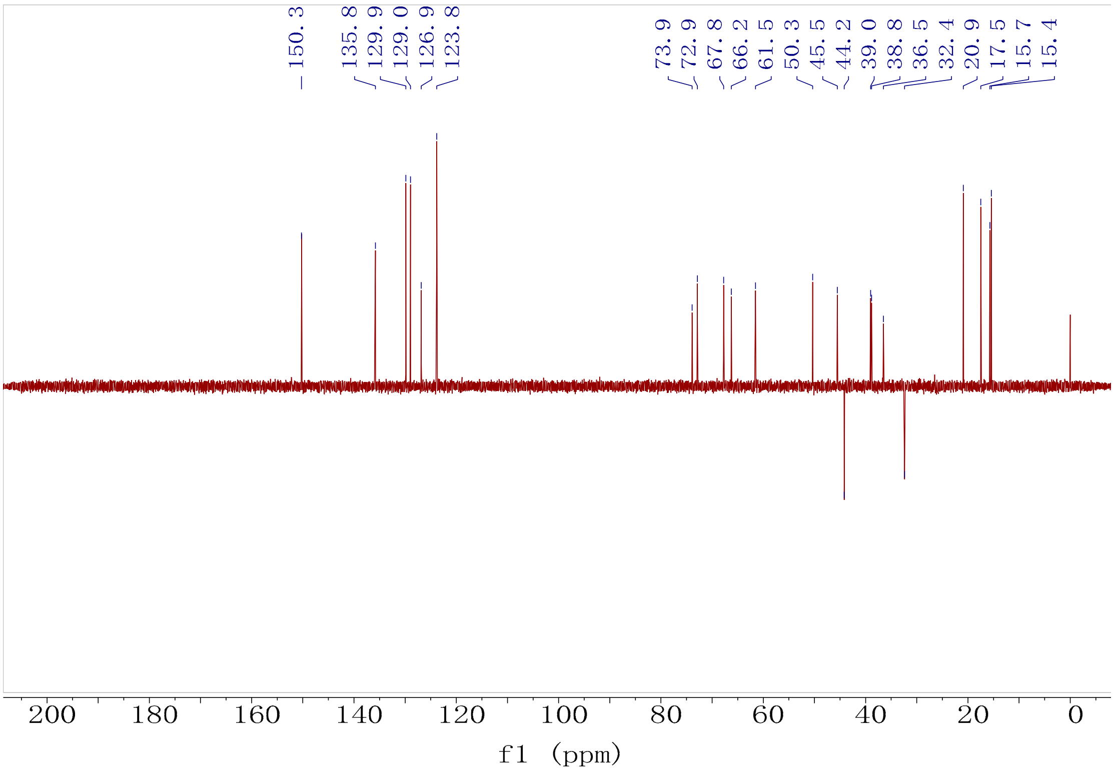


**Figure S48.** DEPT 135 spectra of **5** in pyridine-*d*5.

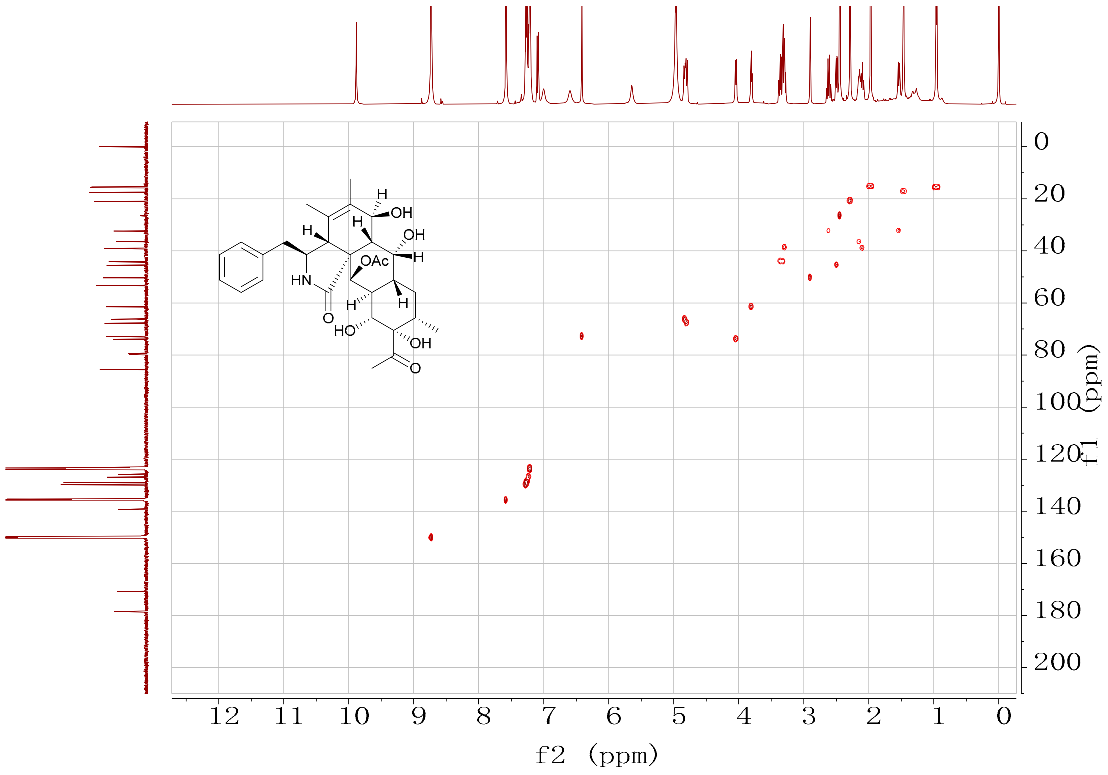


**Figure S49.** HSQC of **5** in pyridine-*d*5.

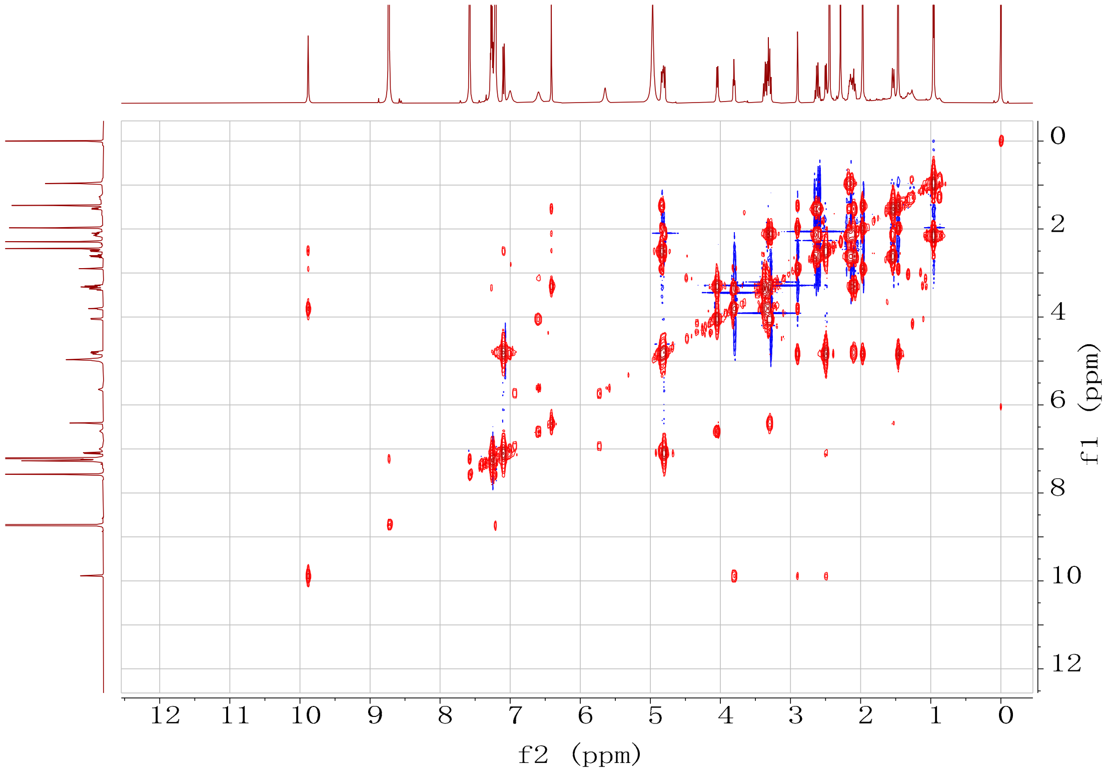

**Figure S50.** $^{1}H$–$^{1}H$ COSY of **5** in pyridine-*d*5.

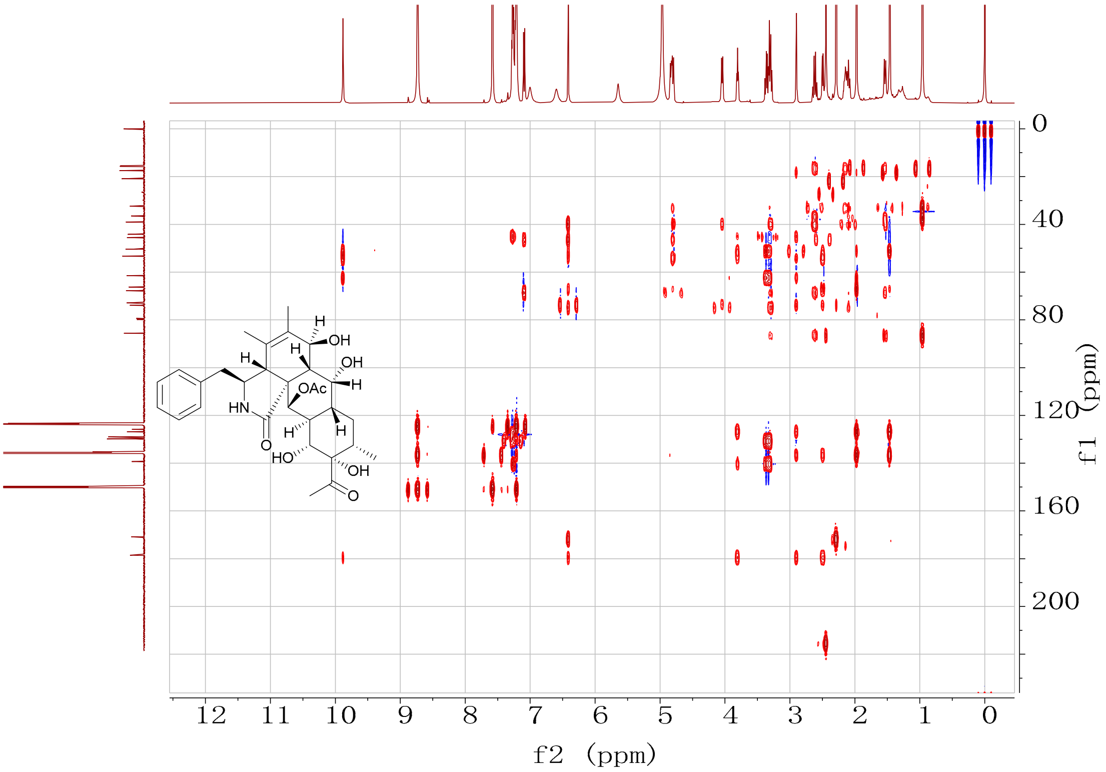


**Figure S51.** HMBC of **5** in pyridine-*d*5.

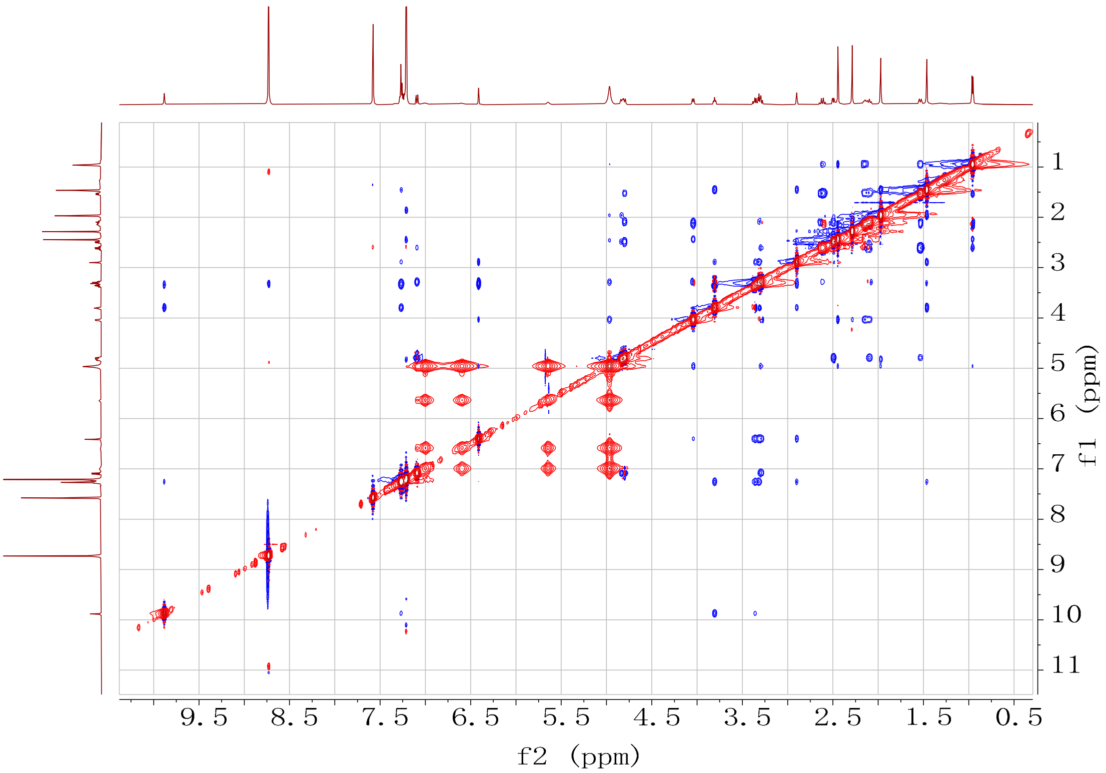


**Figure S52.** NOESY of **5** in pyridine-*d*5.

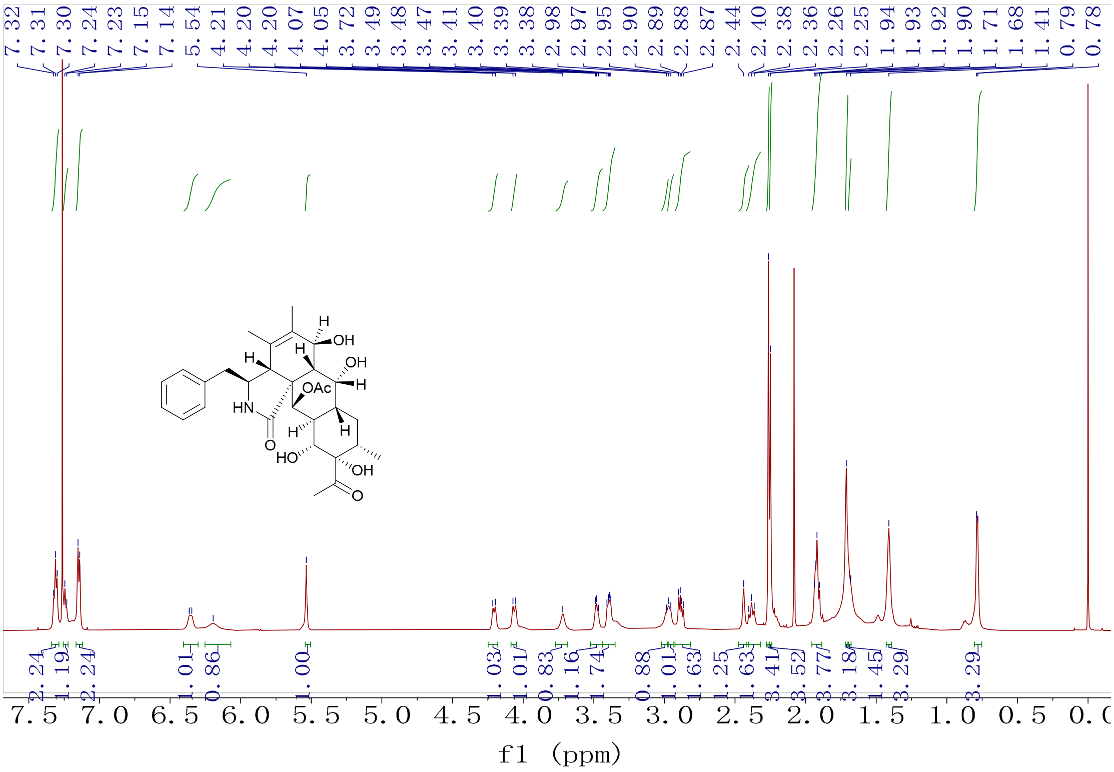


**Figure S53.** $^{1}H$ NMR ($CDCl_3$, 600 MHz) of **5**.

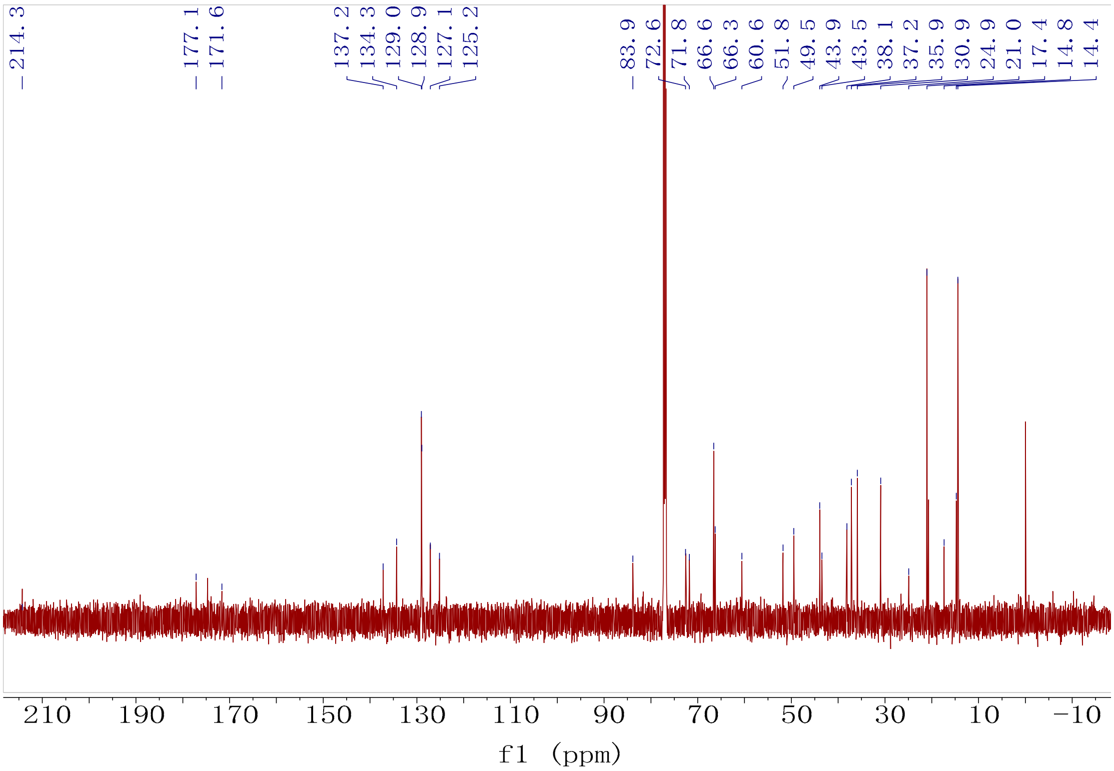

**Figure S54.** $^{13}$C NMR ($CDCl_3$, 150 MHz) of **5**.

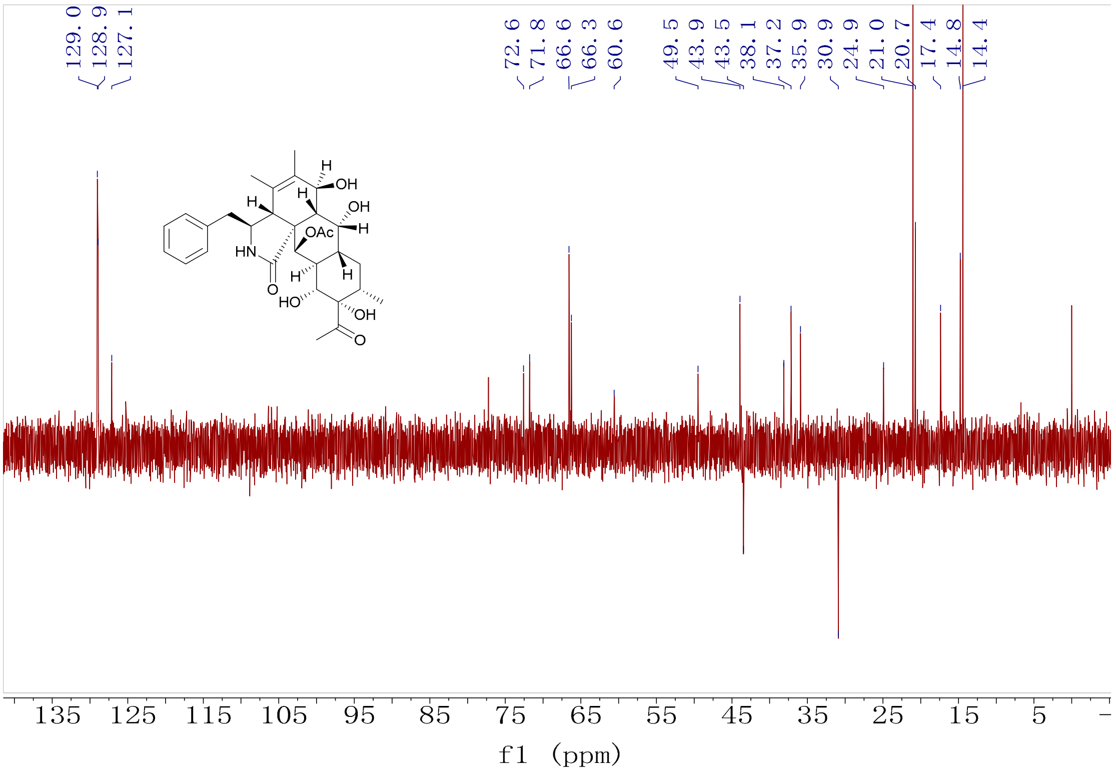


**Figure S55.** DEPT 135 spectra of **5** in $CDCl_3$.

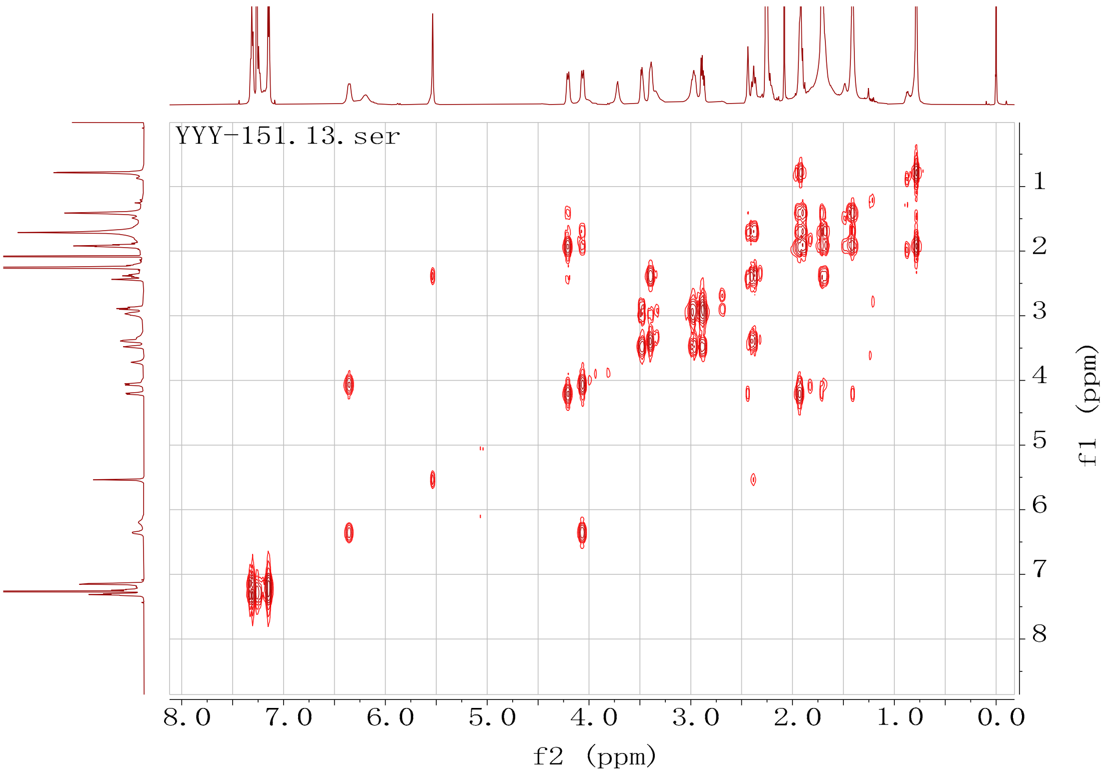


**Figure S56.** $^{1}$H−$^{1}$H COSY of **5** in $CDCl_3$.

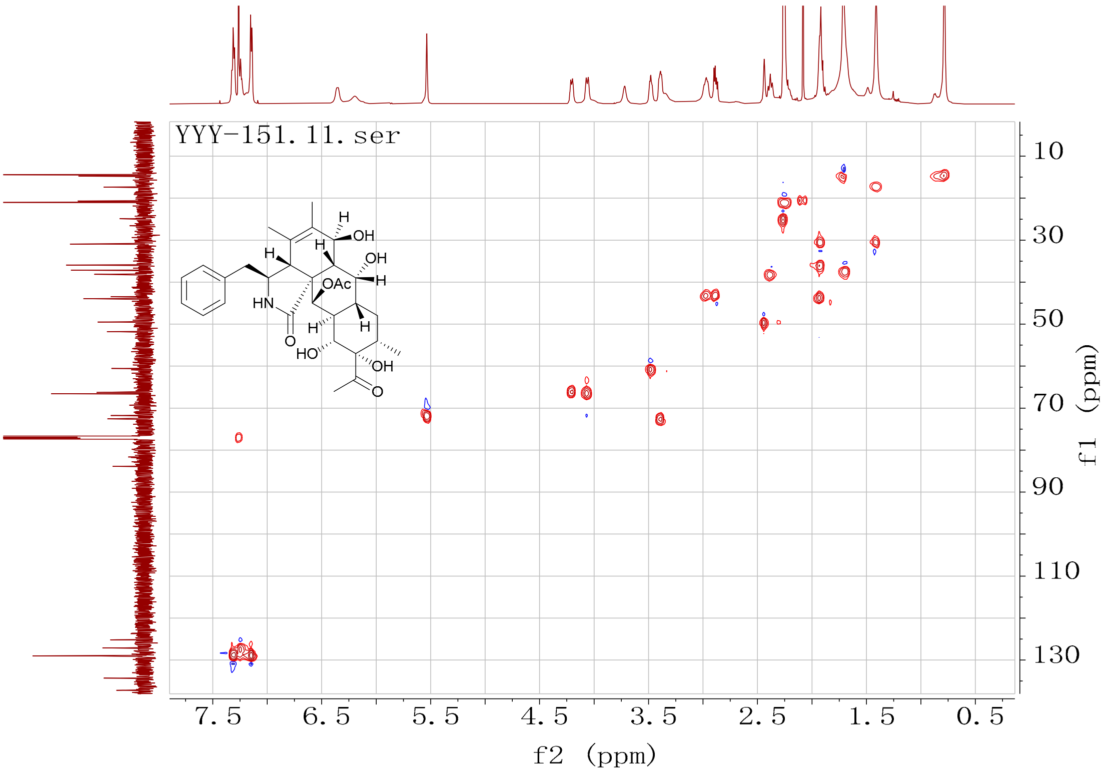


**Figure S57.** HSQC of **5** in $CDCl_3$.

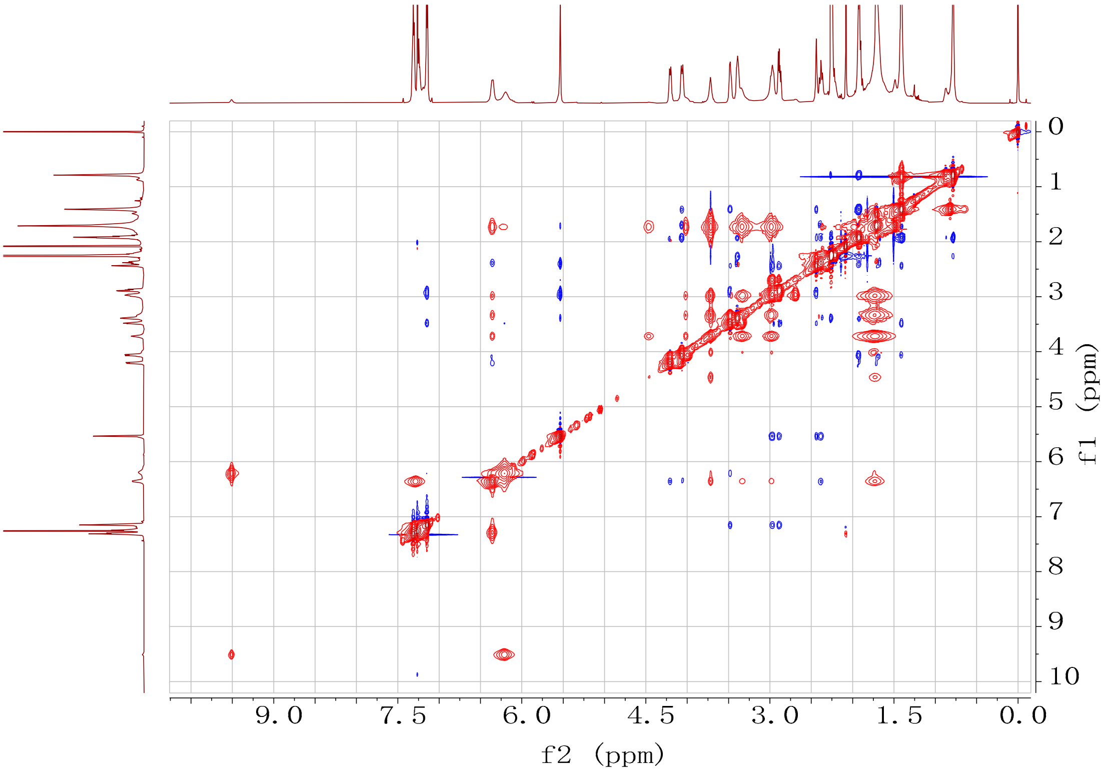


**Figure S58.** NOESY of **5** in $CDCl_3$.

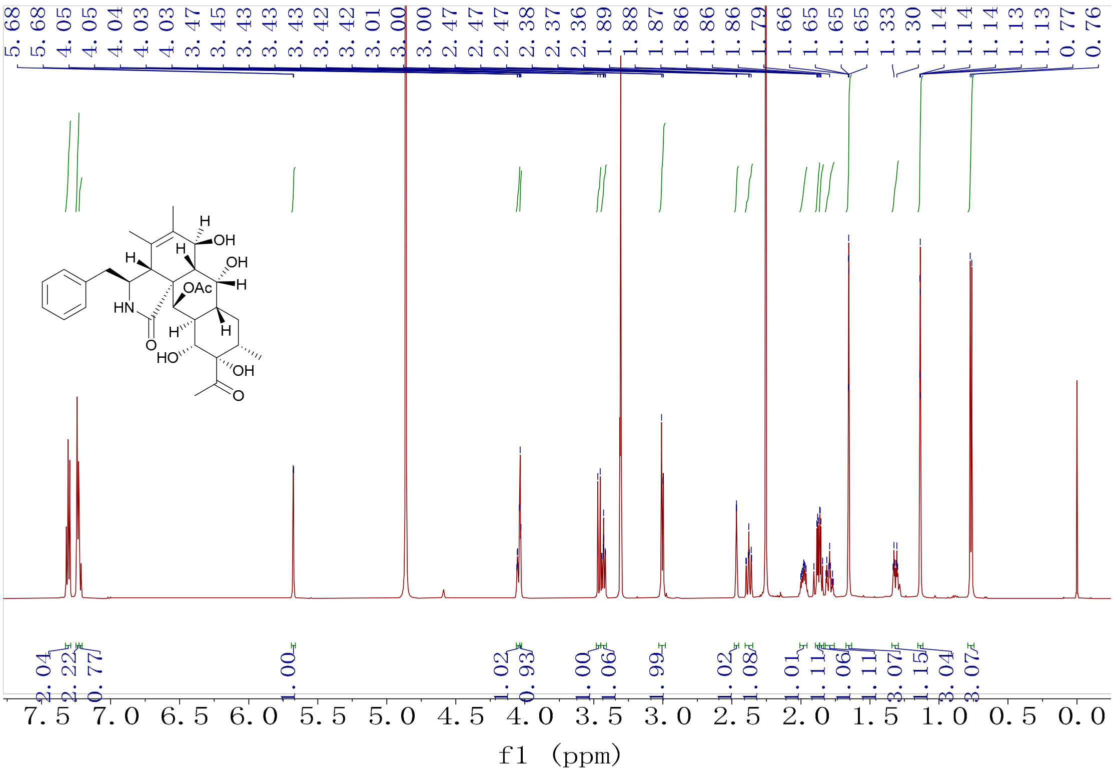

**Figure S59.** $^{1}H$ NMR ($CD_3OD$, 600 MHz) of **5**.

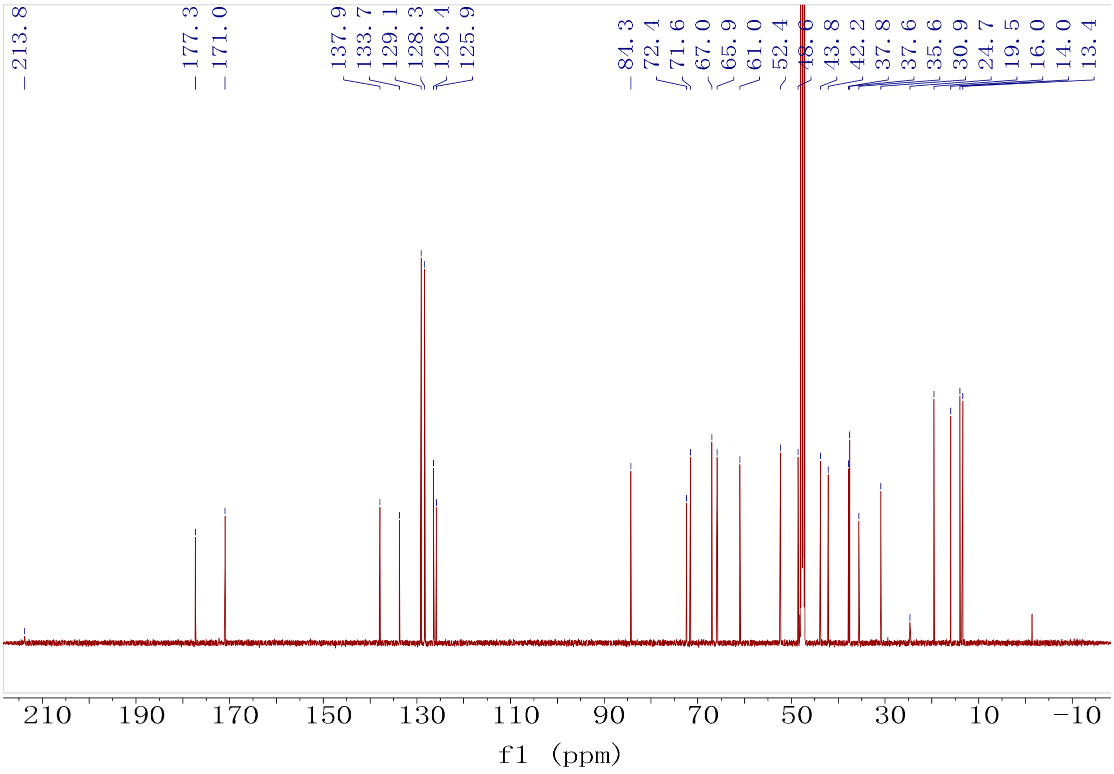

**Figure S60.** $^{13}C$ NMR ($CD_3OD$, 150 MHz) of **5**.

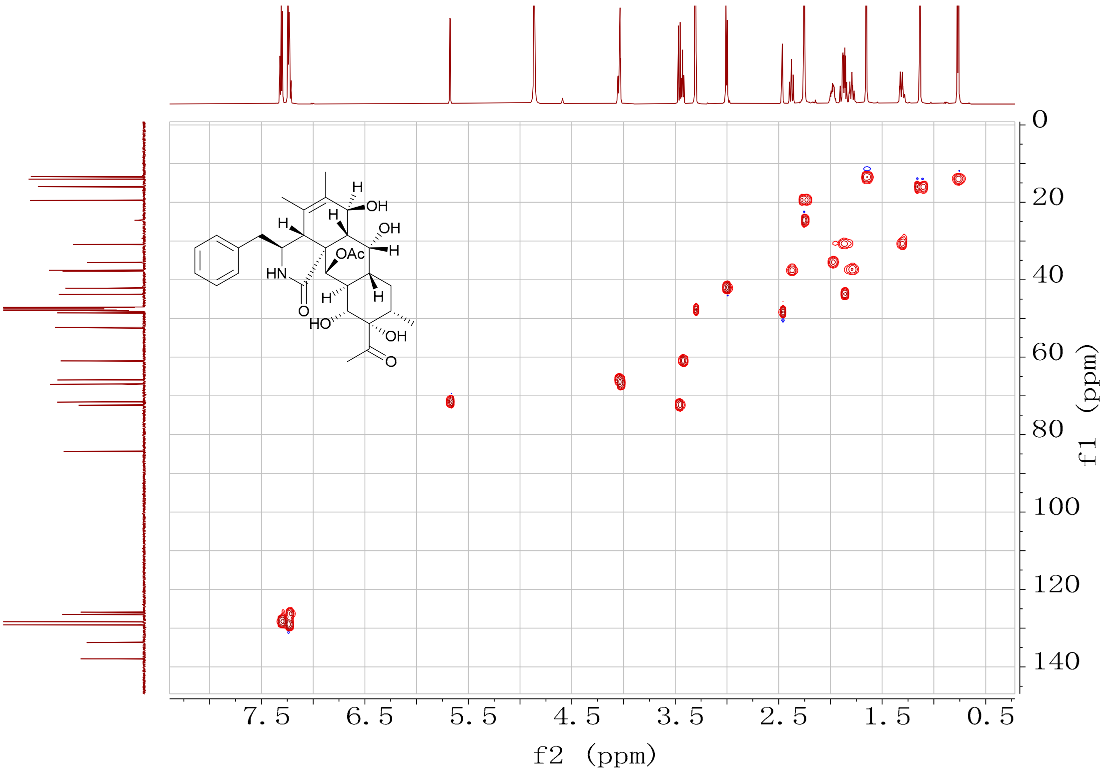


**Figure S61.** HSQC of **5** in CD3OD.

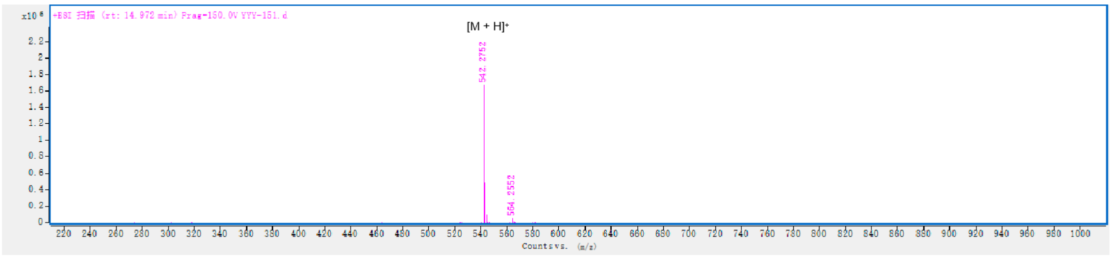


**Figure S62.** HRESIMS of **5**.

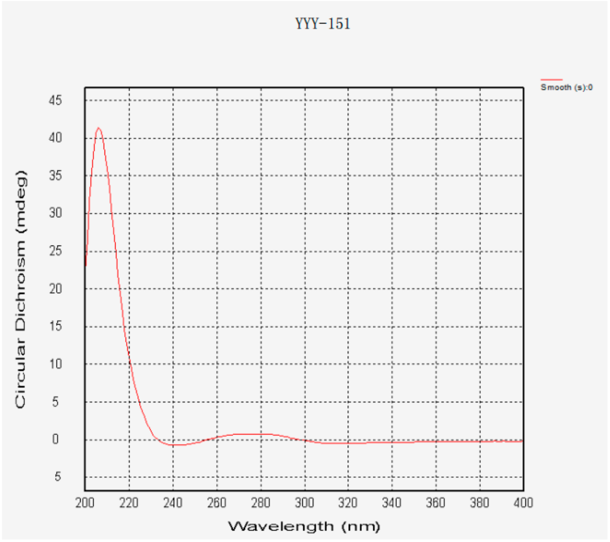


**Figure S63.** CD spectrum (methanol) of **5.**

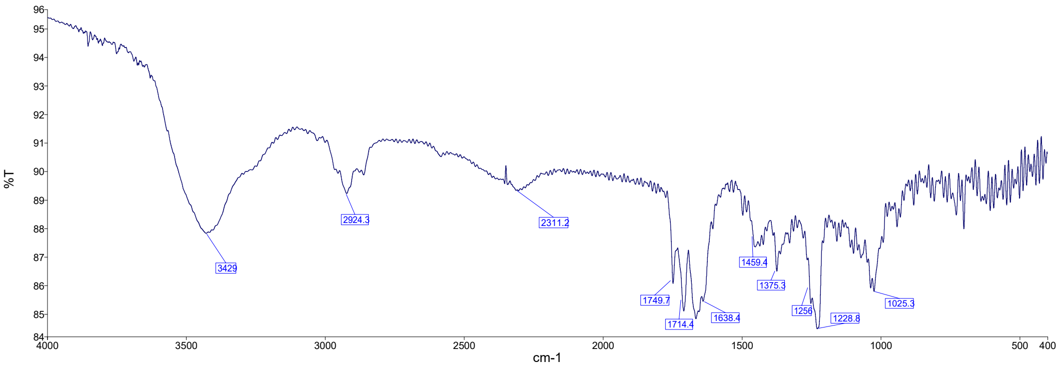

**Figure S64.** IR spectrum of **5.**

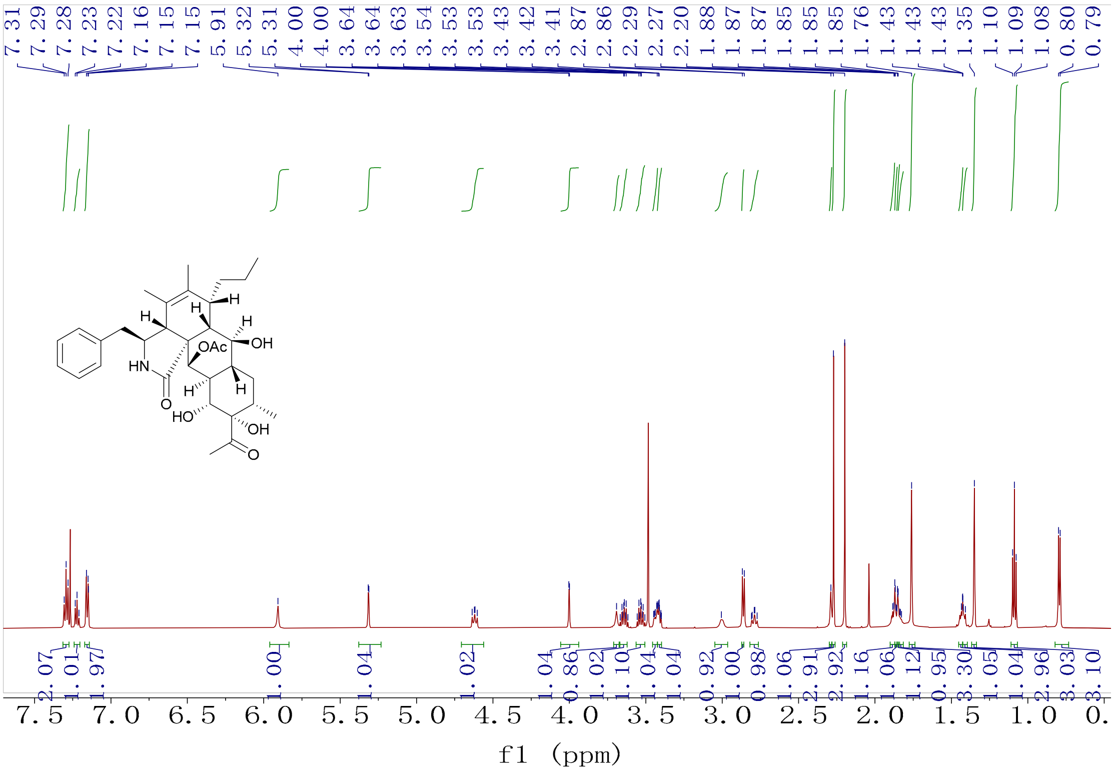

**Figure S65.** $^{1}$H NMR ($CDCl_3$, 600 MHz) of **6**.

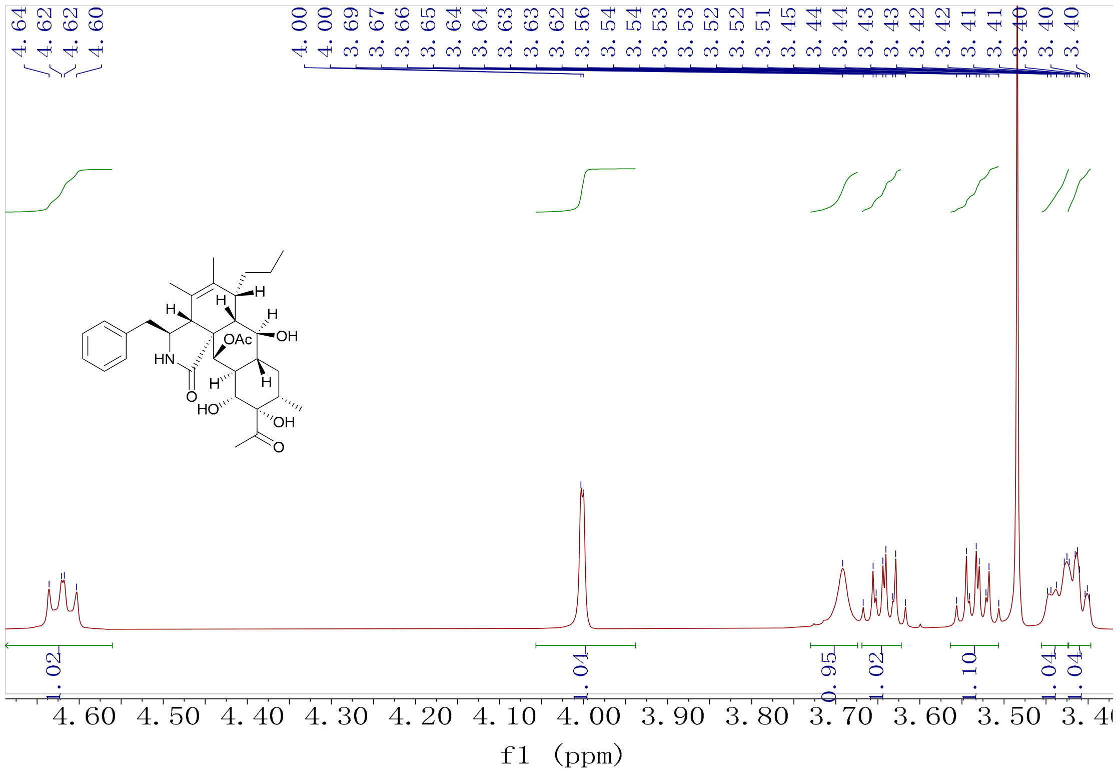


**Figure S66.** Partial $^{1}$H NMR ($CDCl_3$, 600 MHz) of **6**.

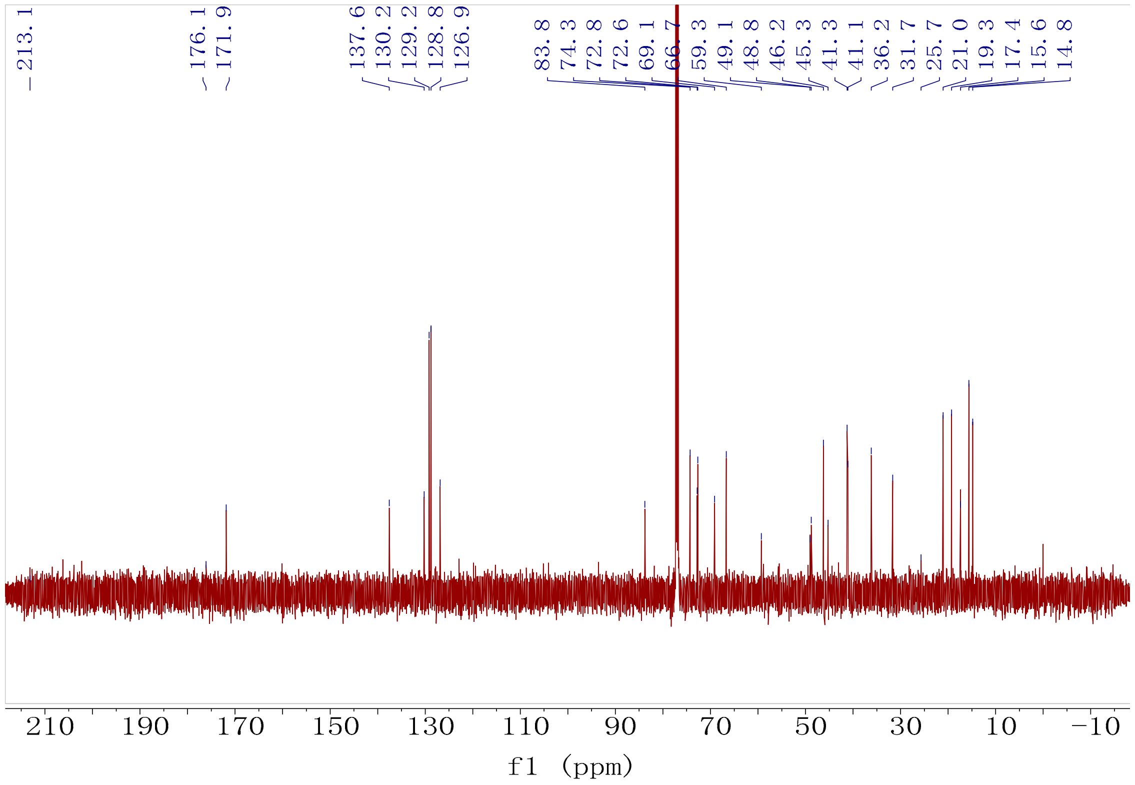


**Figure S67.** $^{13}$C NMR ($CDCl_3$, 150 MHz) of **6**.

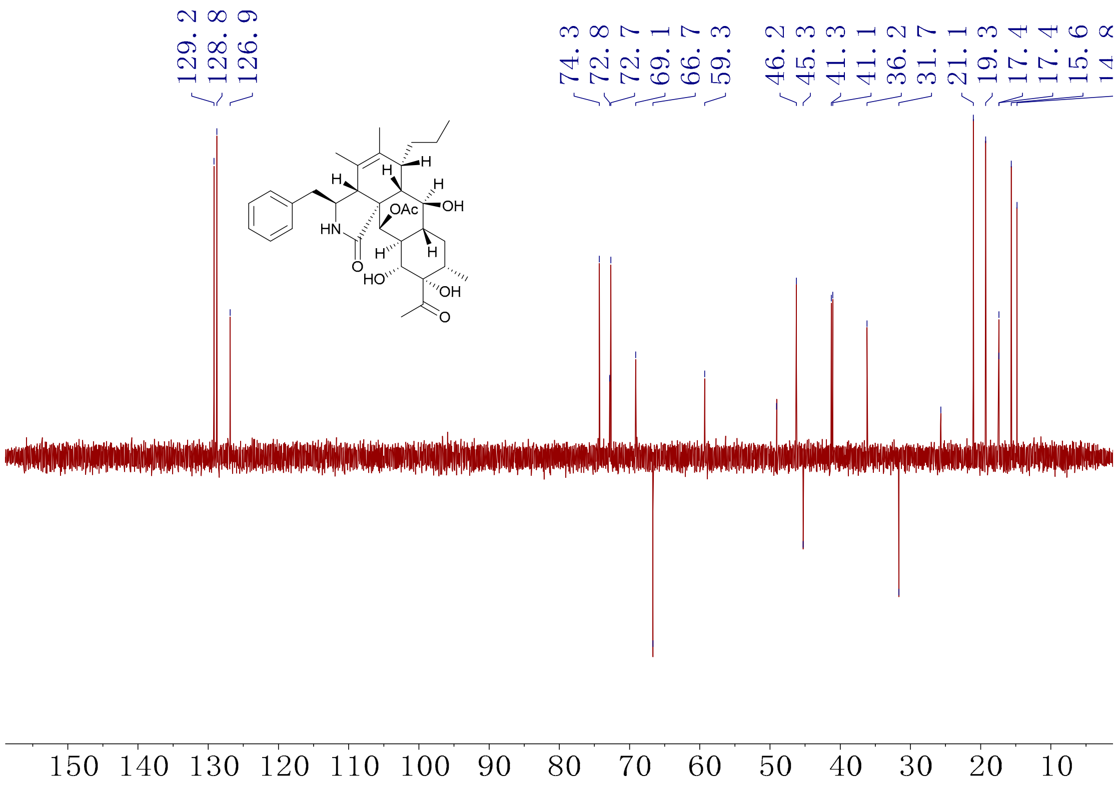


**Figure S68.** DEPT 135 spectra of **6**.

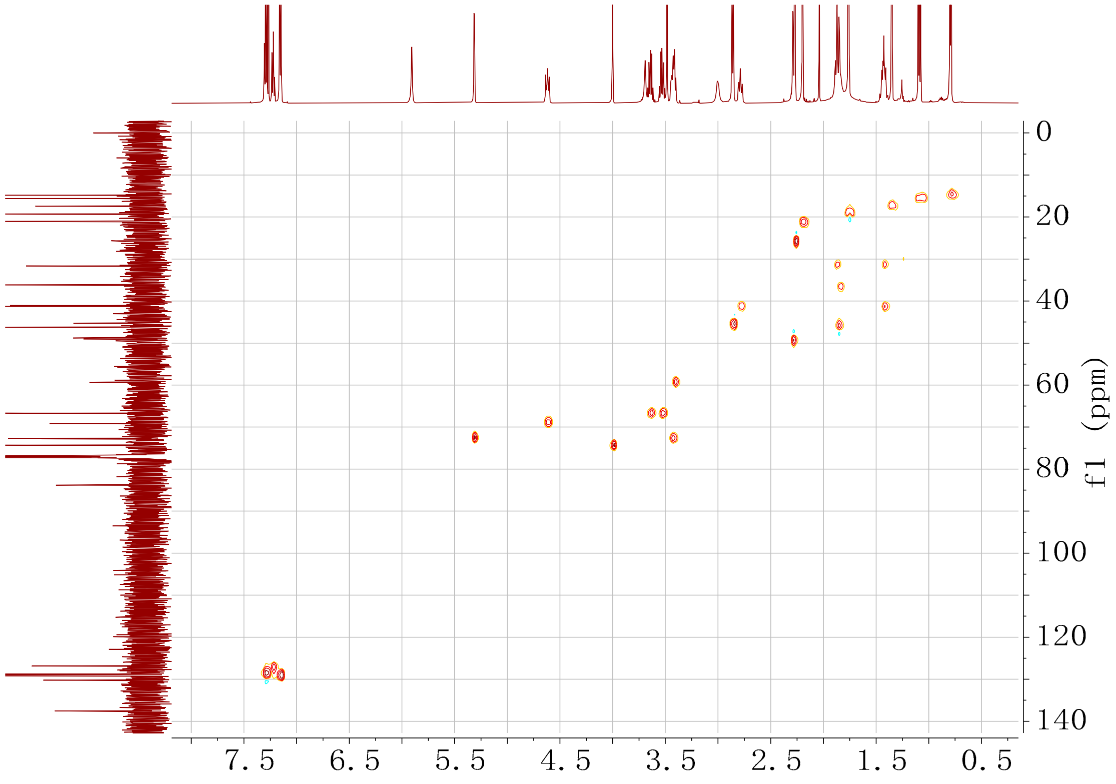


**Figure S69.** HSQC of **6**.

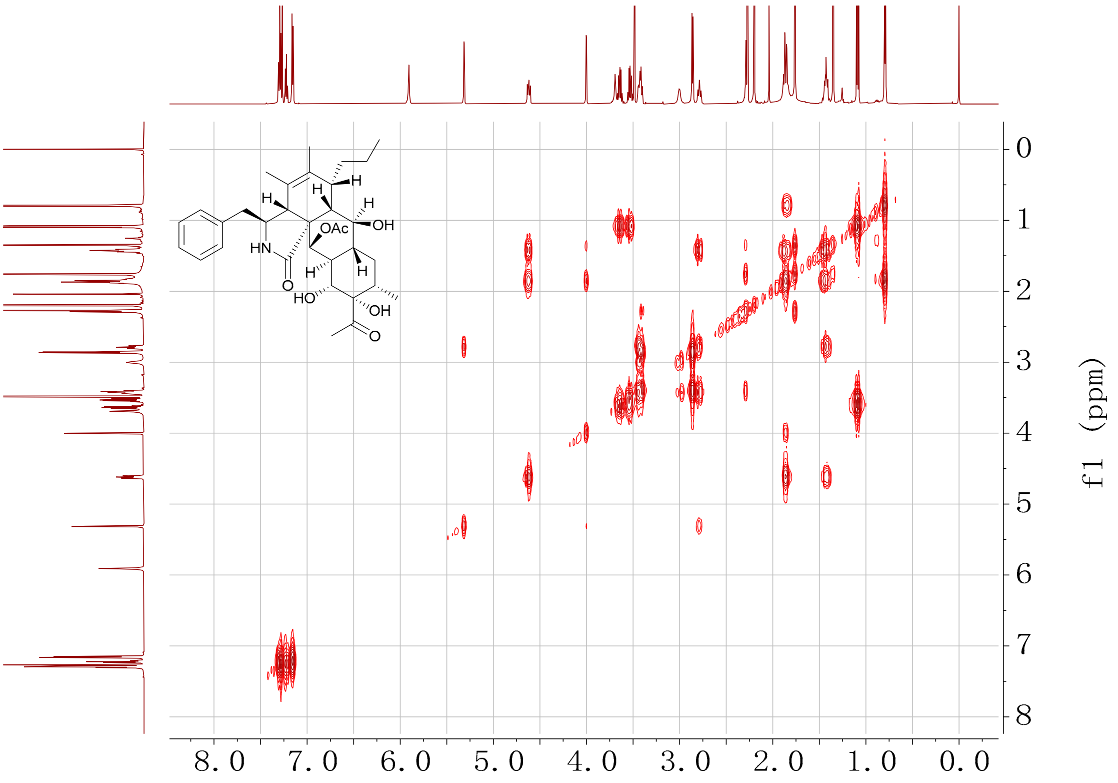


**Figure S70.** $^1$H–$^1$H COSY of **6**.

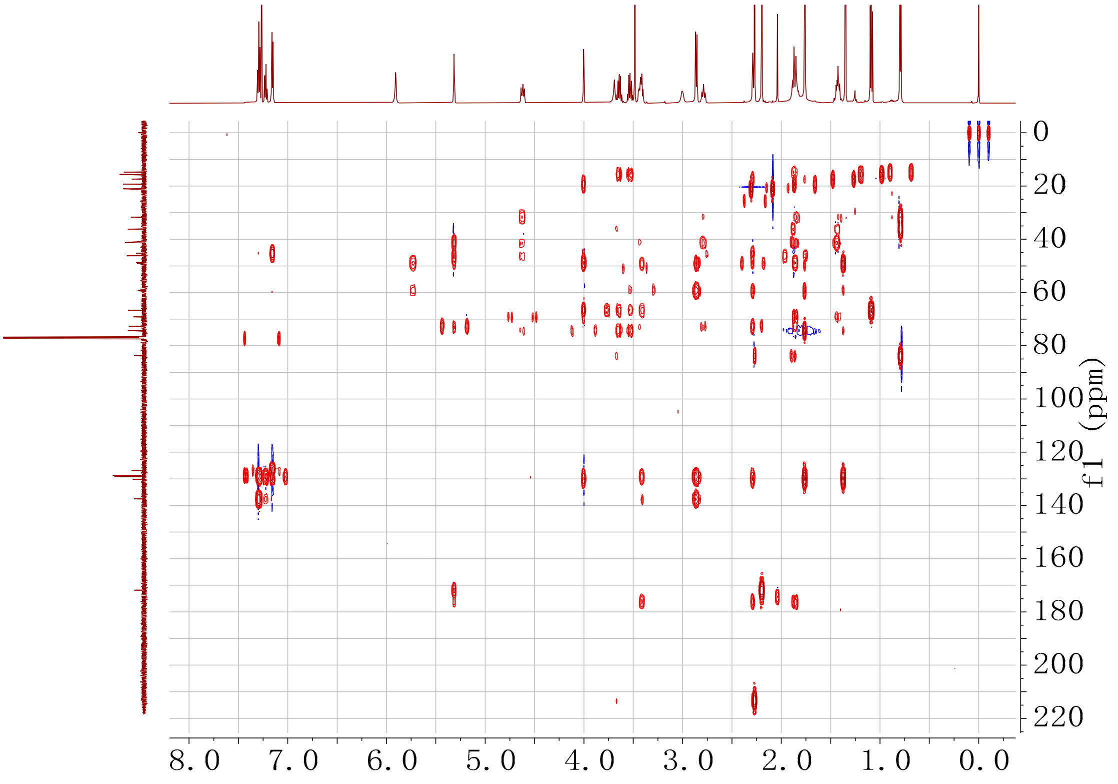


**Figure S71.** HMBC of **6**.

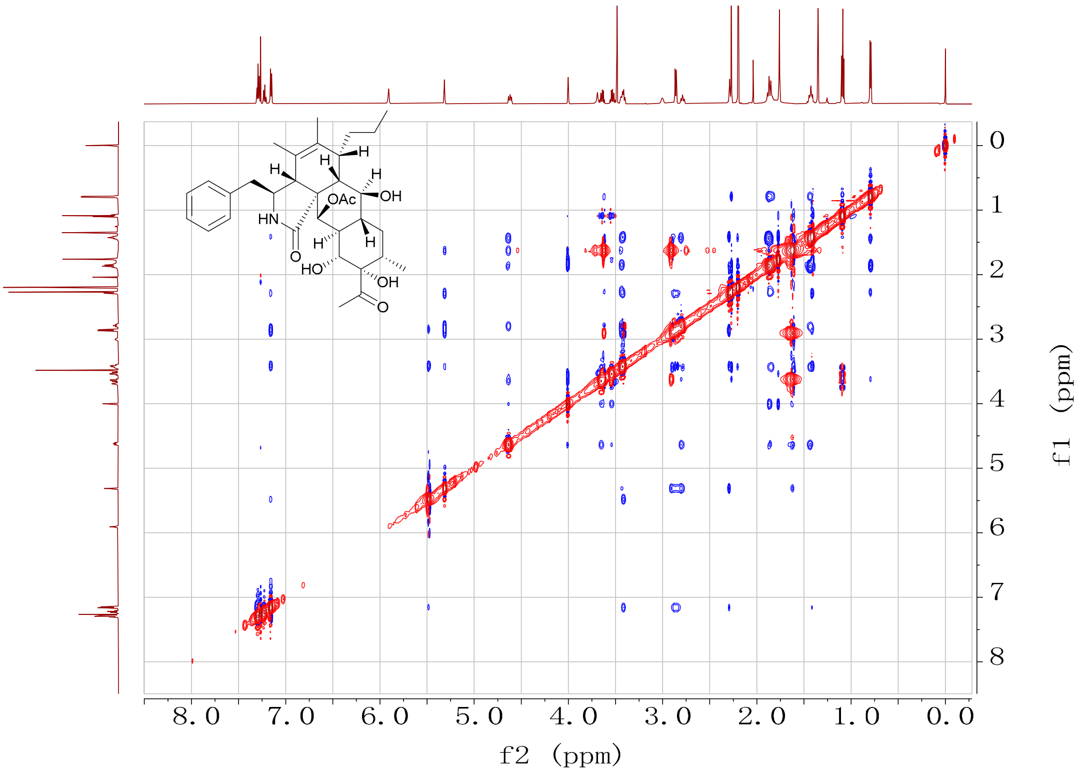


**Figure S72.** NOESY of **6**.

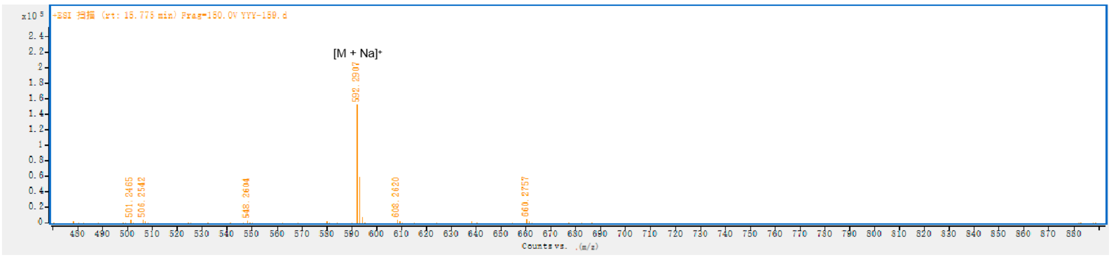


**Figure S73.** HRESIMS of **6**.

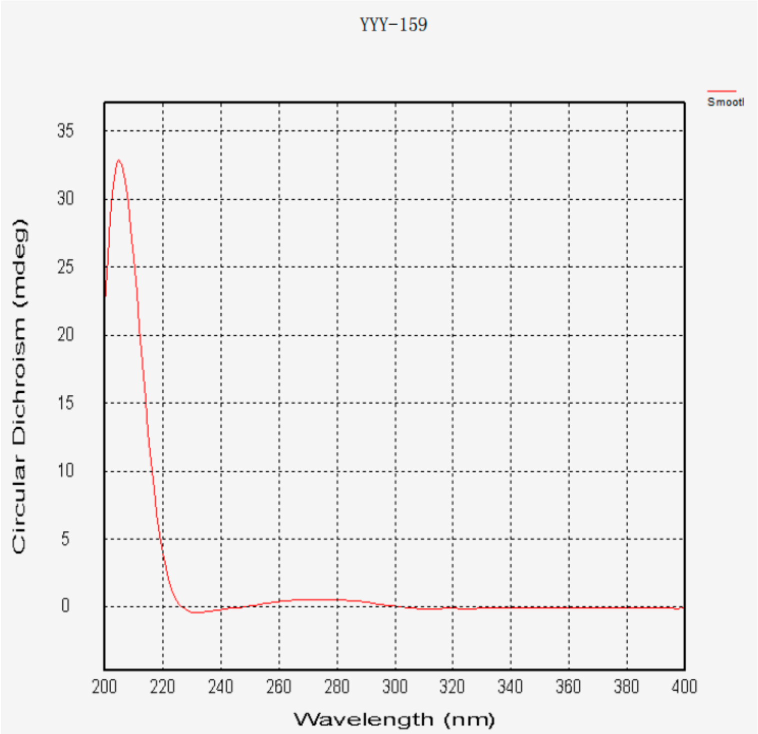


**Figure S74.** CD spectrum (methanol) of **6.**

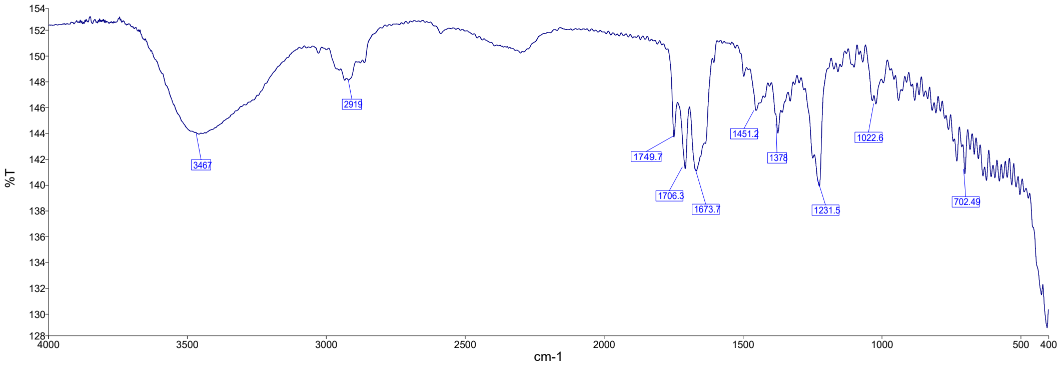


**Figure S75.** IR spectrum of **6.**

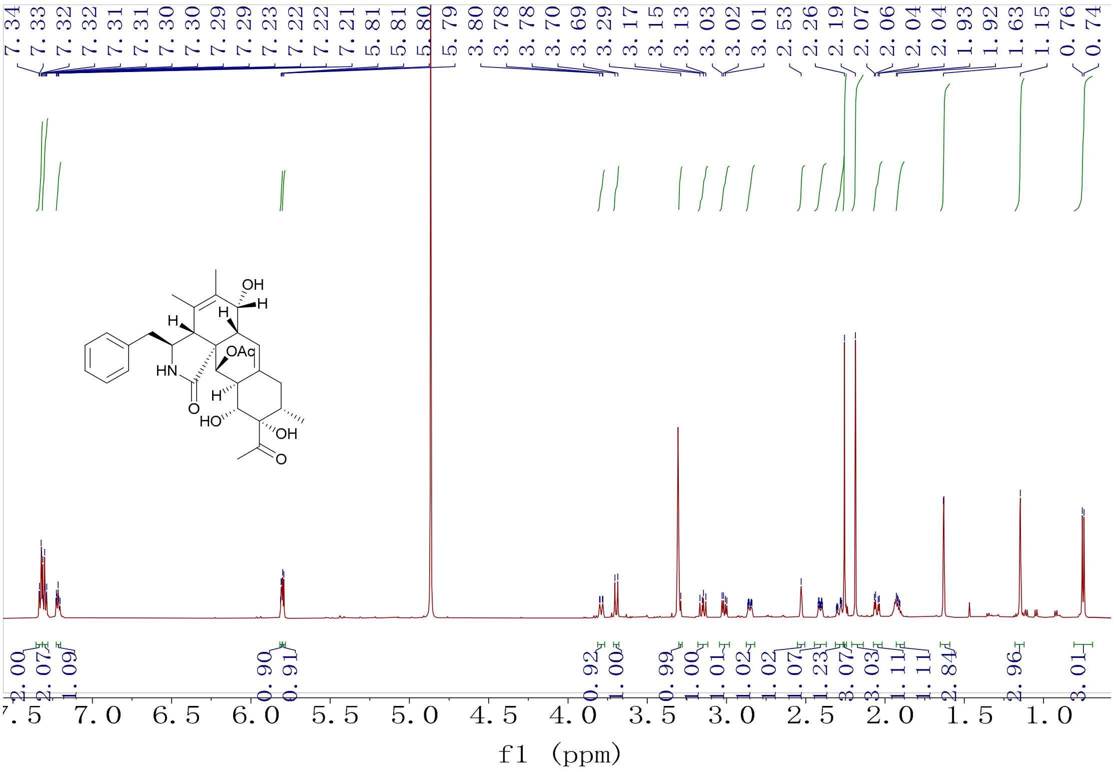

**Figure S76.** $^1$H NMR ($CD_3OD$, 600 MHz) of **7**.

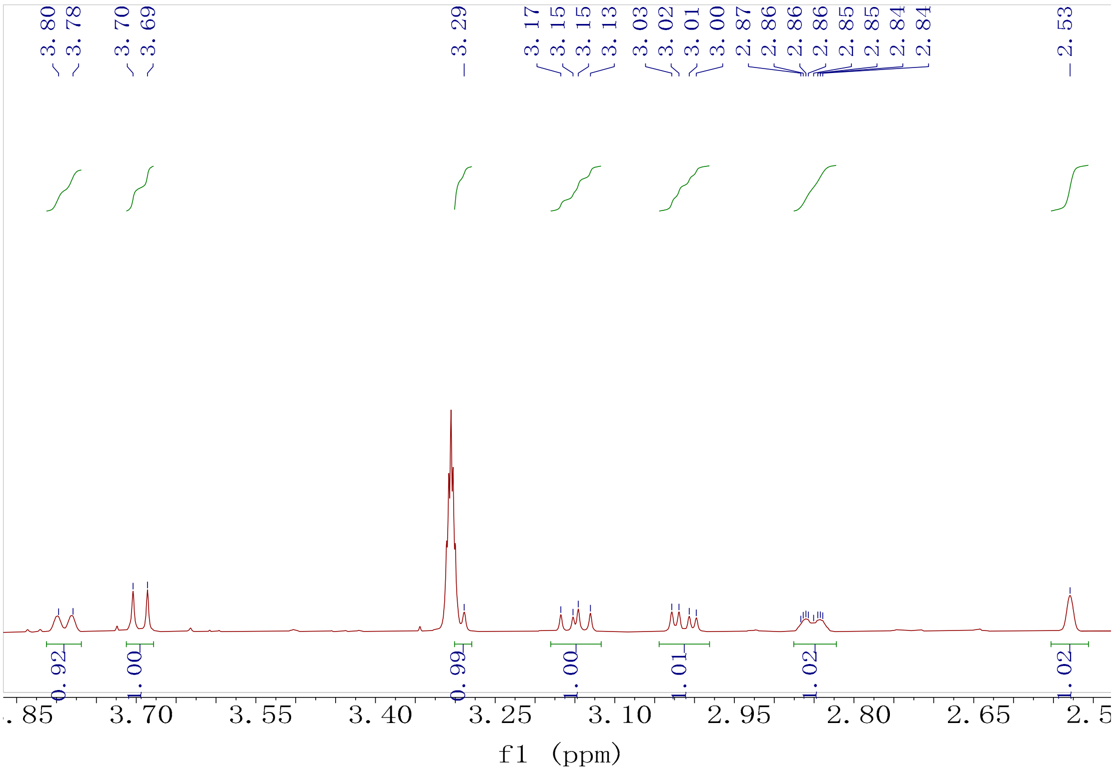

**Figure S77.** Partial $^1$H NMR ($CDCl_3$, 600 MHz) of **7**.

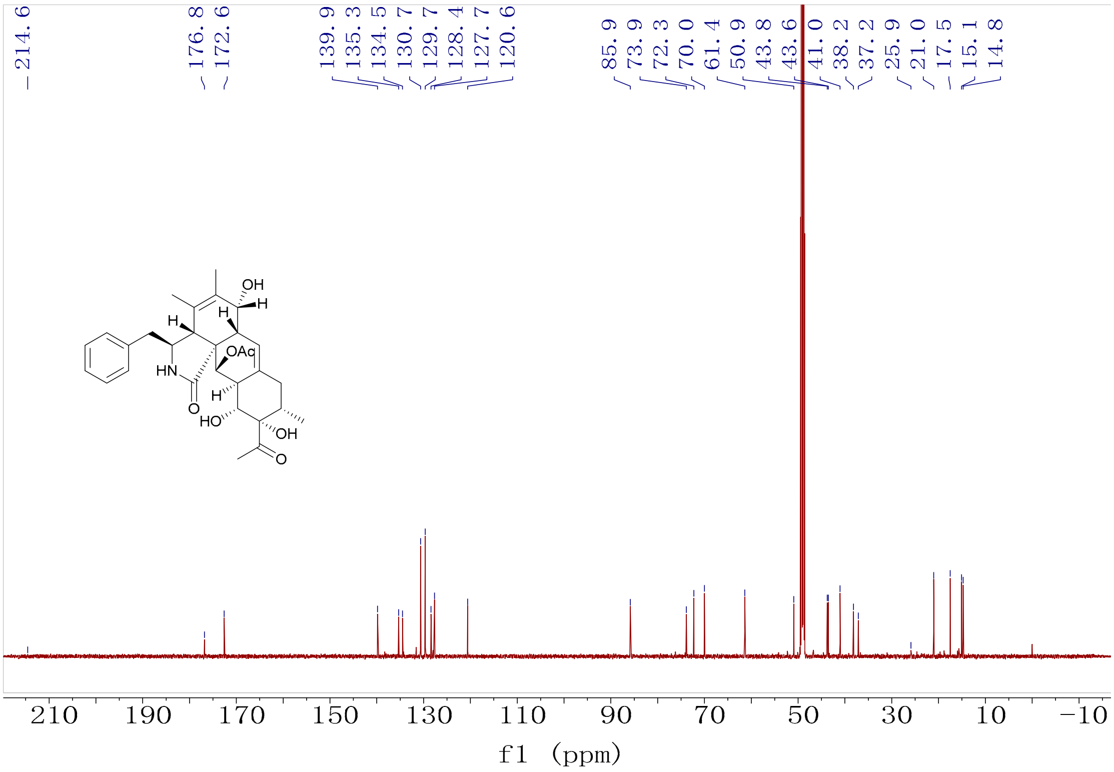


**Figure S78.** $^{13}$C NMR ($CD_3OD$, 150 MHz) of **7**.

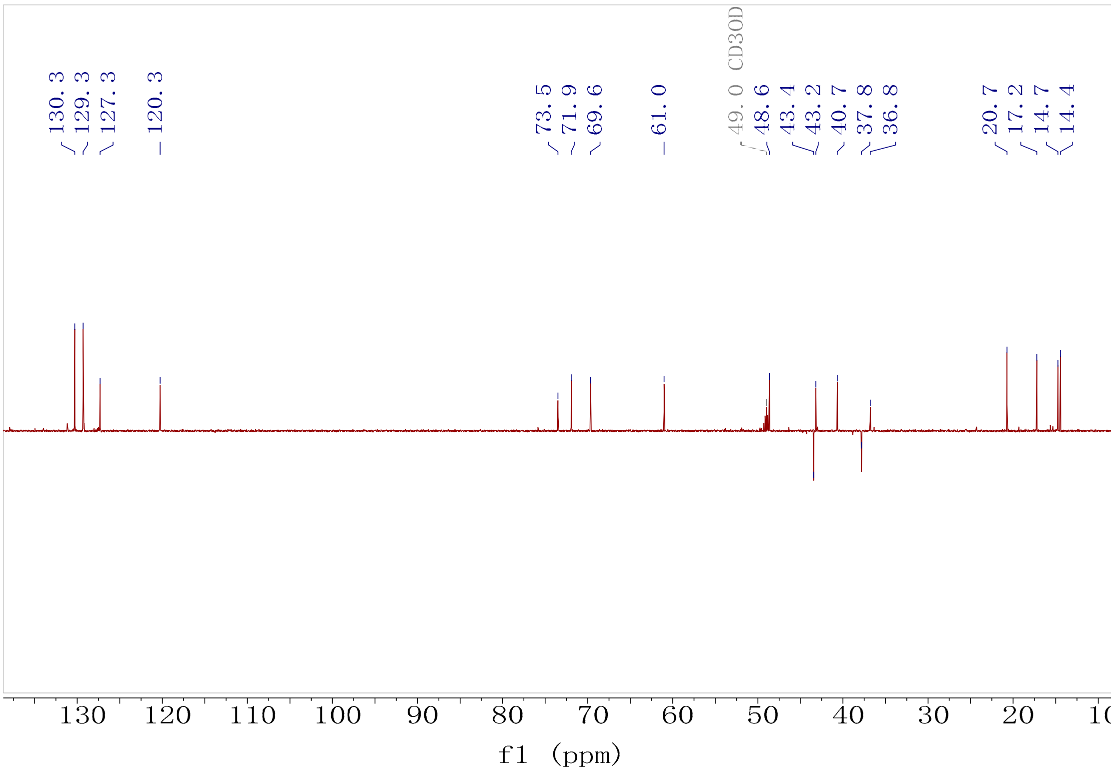


**Figure S79.** DEPT 135 spectra of **7**.

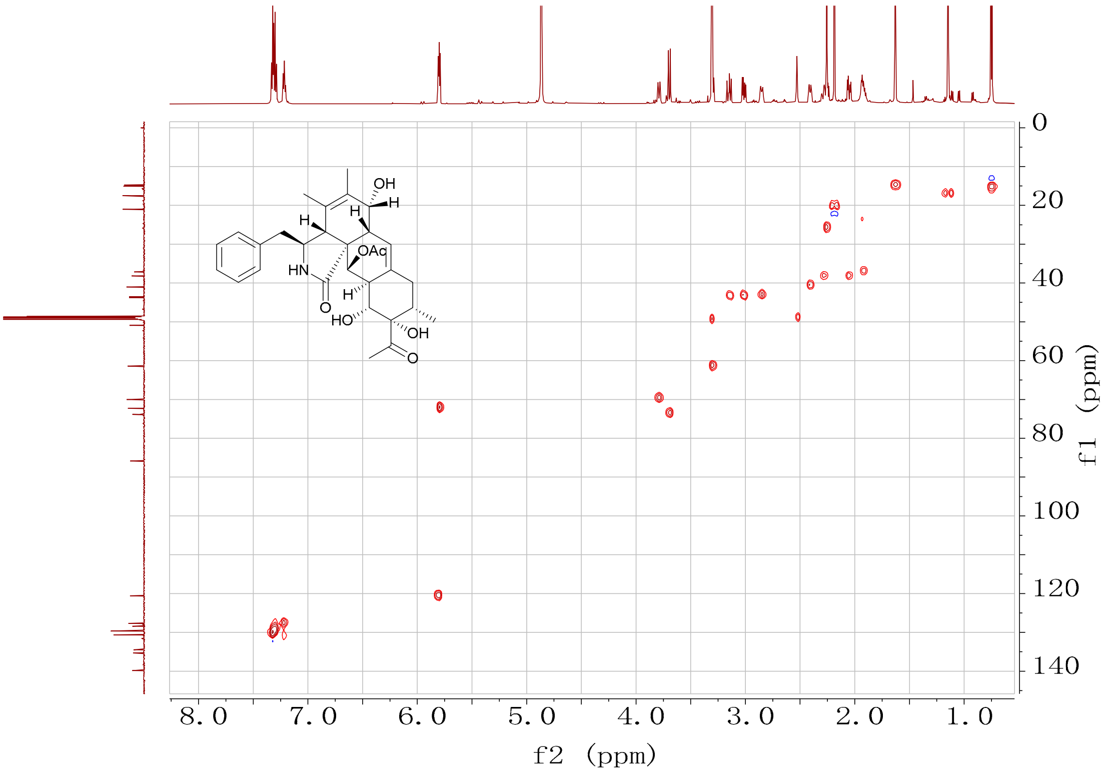


**Figure S80.** HSQC of **7**.

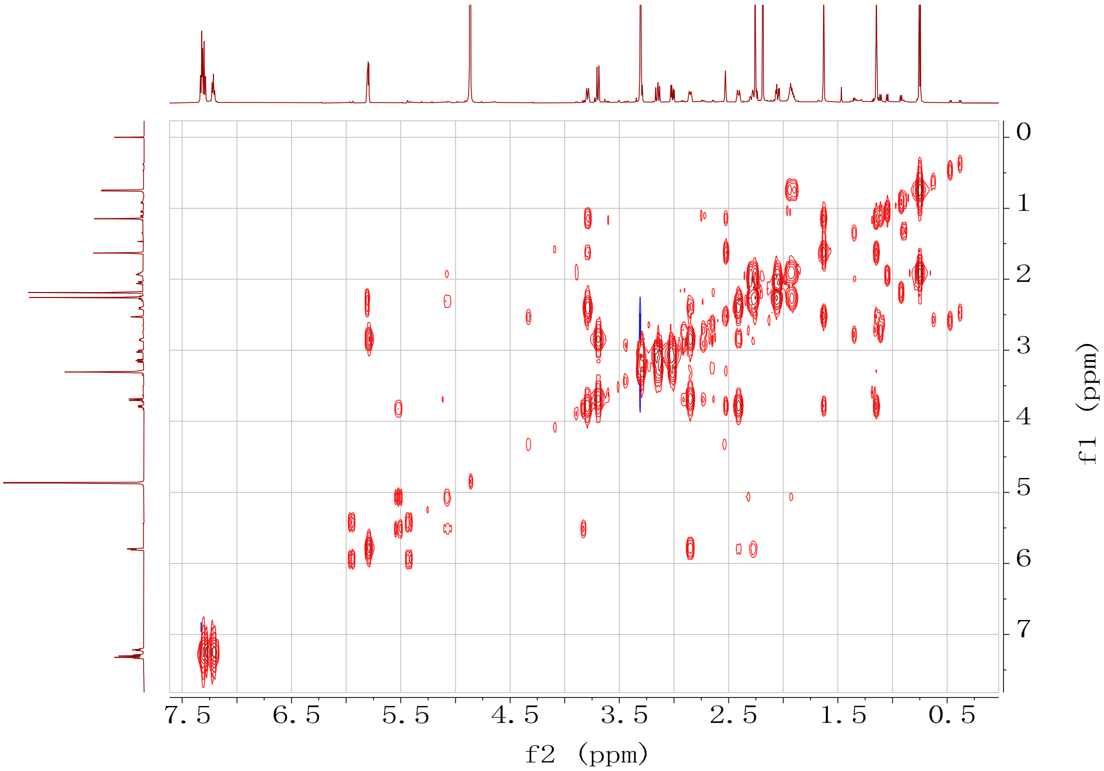


**Figure S81.** $^1$H−$^1$H COSY of **7**.

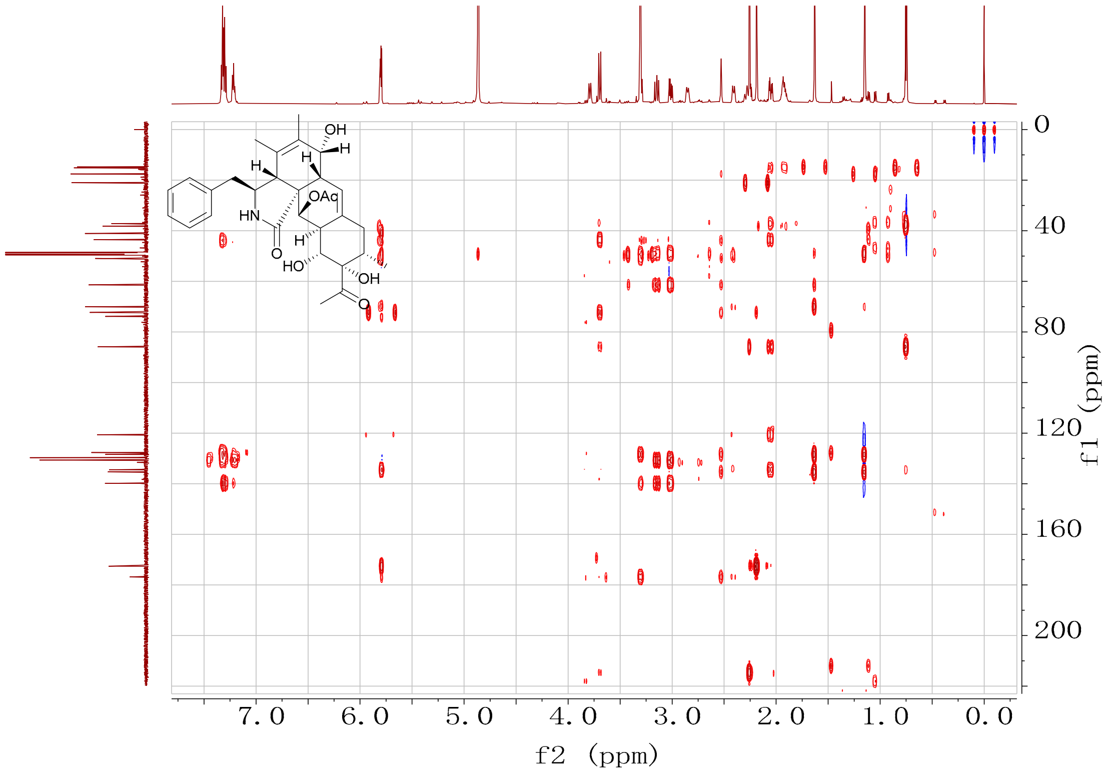


**Figure S82.** HMBC of **7**.

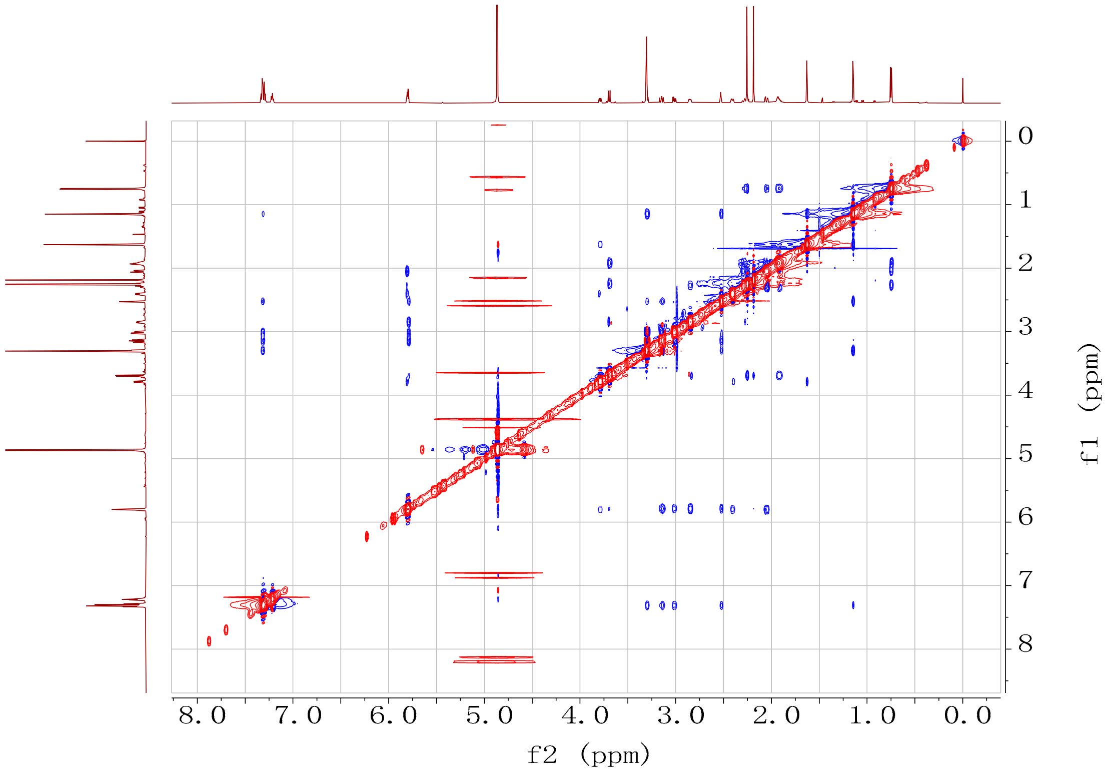


**Figure S83.** NOESY of **7**.

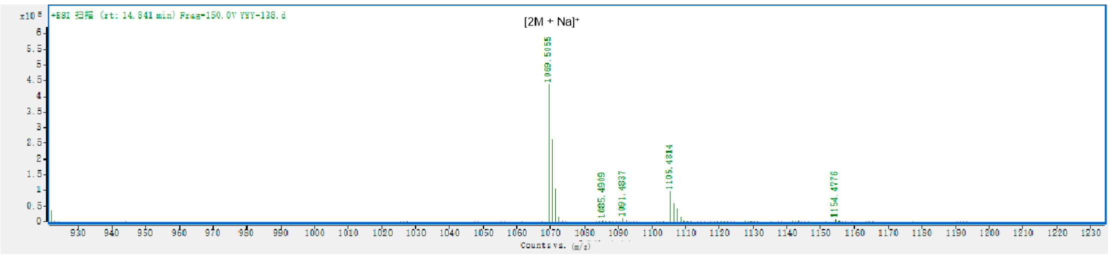


**Figure S84.** HRESIMS of **7**.

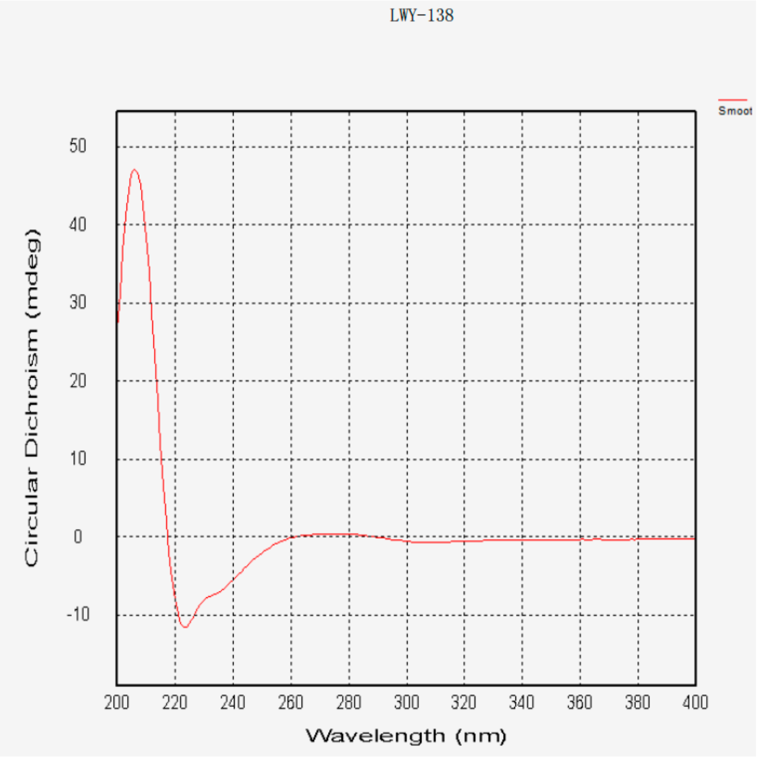


**Figure S85.** CD spectrum (acetonitrile) of **7.**

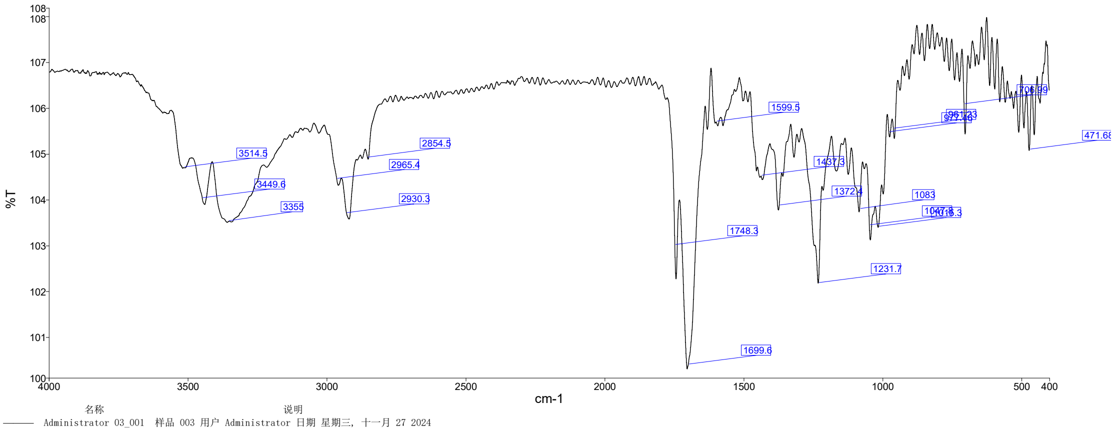


**Figure S86.** IR spectrum of **7.**

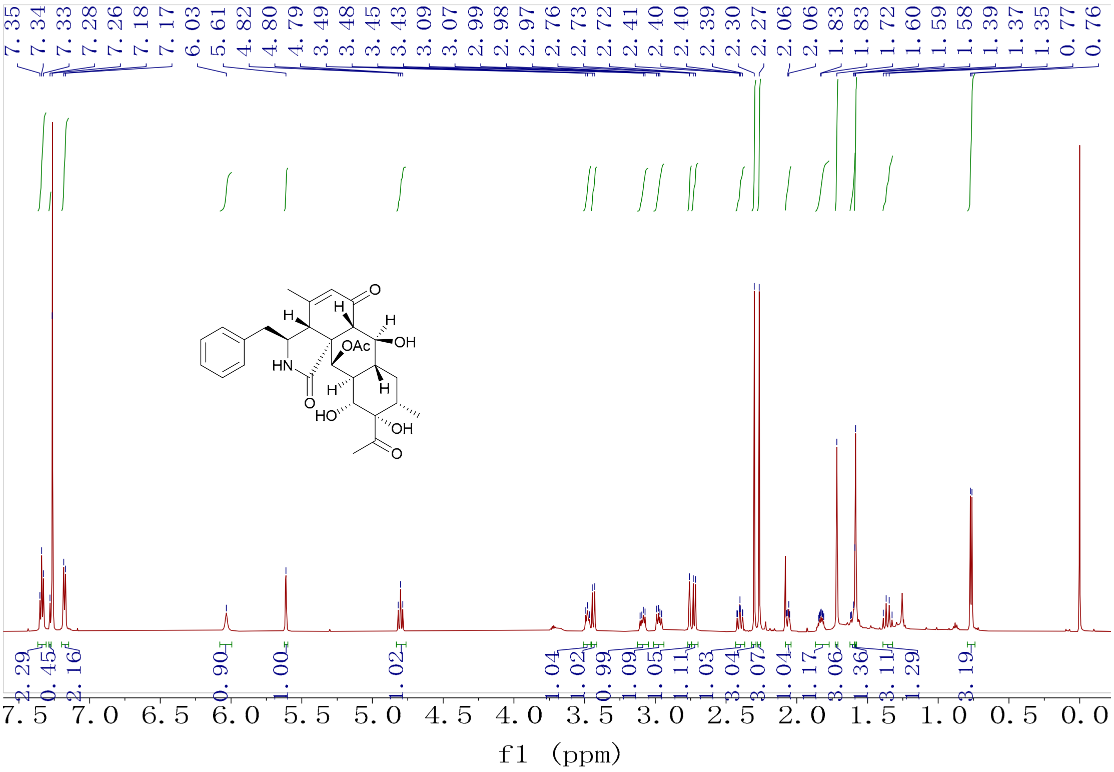


**Figure S87.** $^1$H NMR ($CDCl_3$, 600 MHz) of **8**.

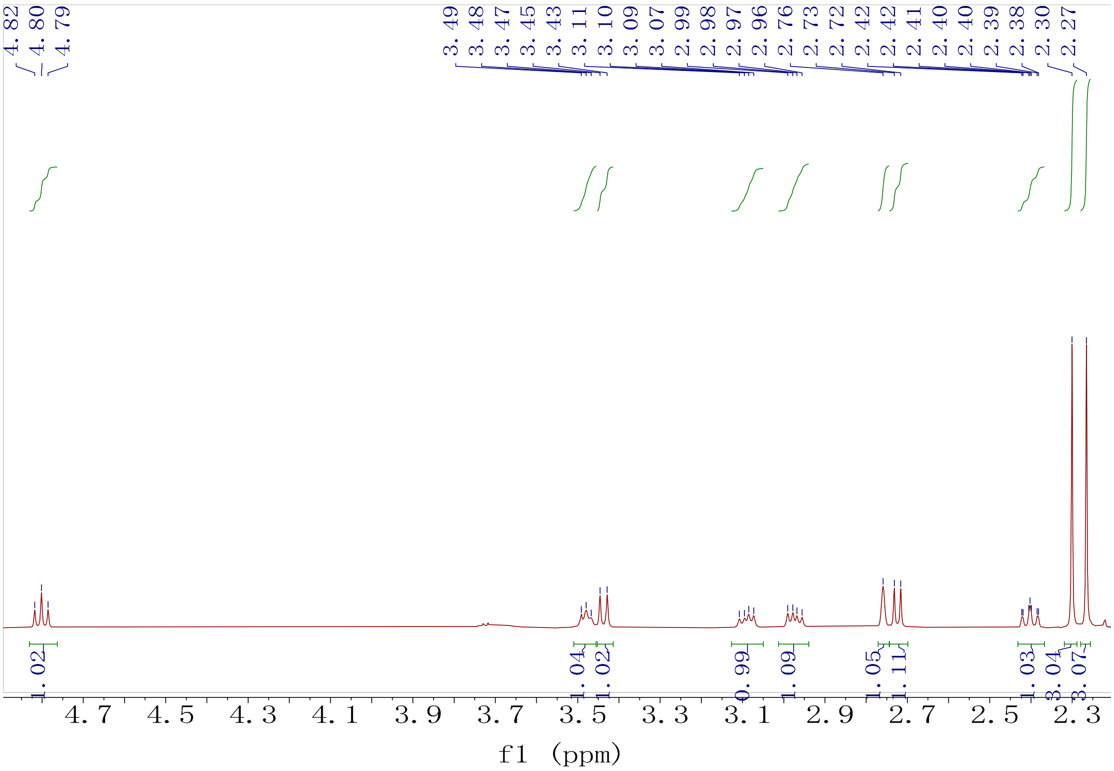


**Figure S88.** Partial $^1$H NMR ($CDCl_3$, 600 MHz) of **8**.

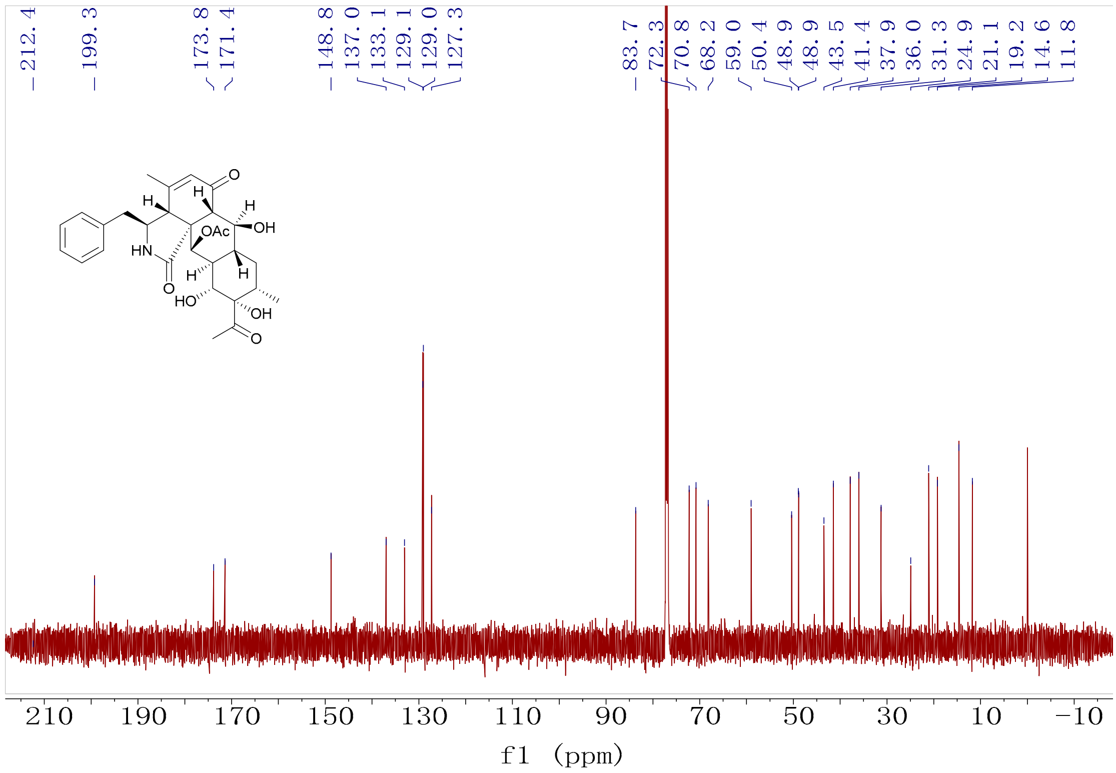


**Figure S89.** $^{13}C$ NMR ($CDCl_3$, 150 MHz) of **8**.

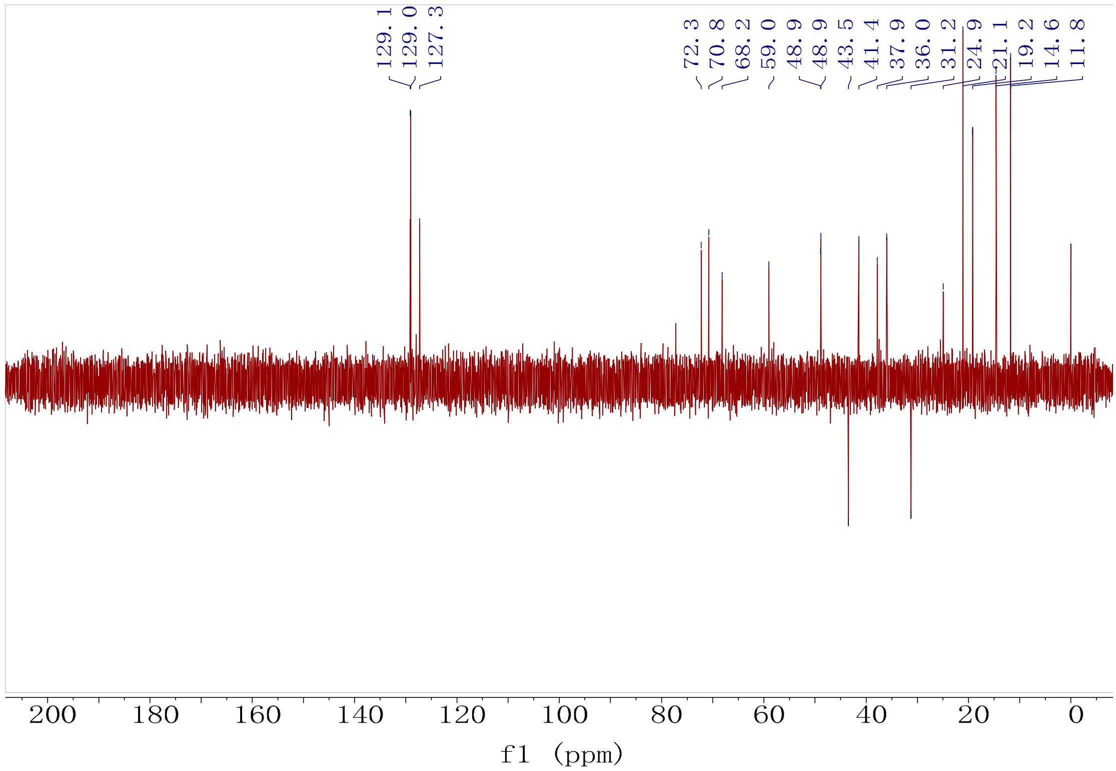


**Figure S90.** DEPT 135 spectra of **8**.

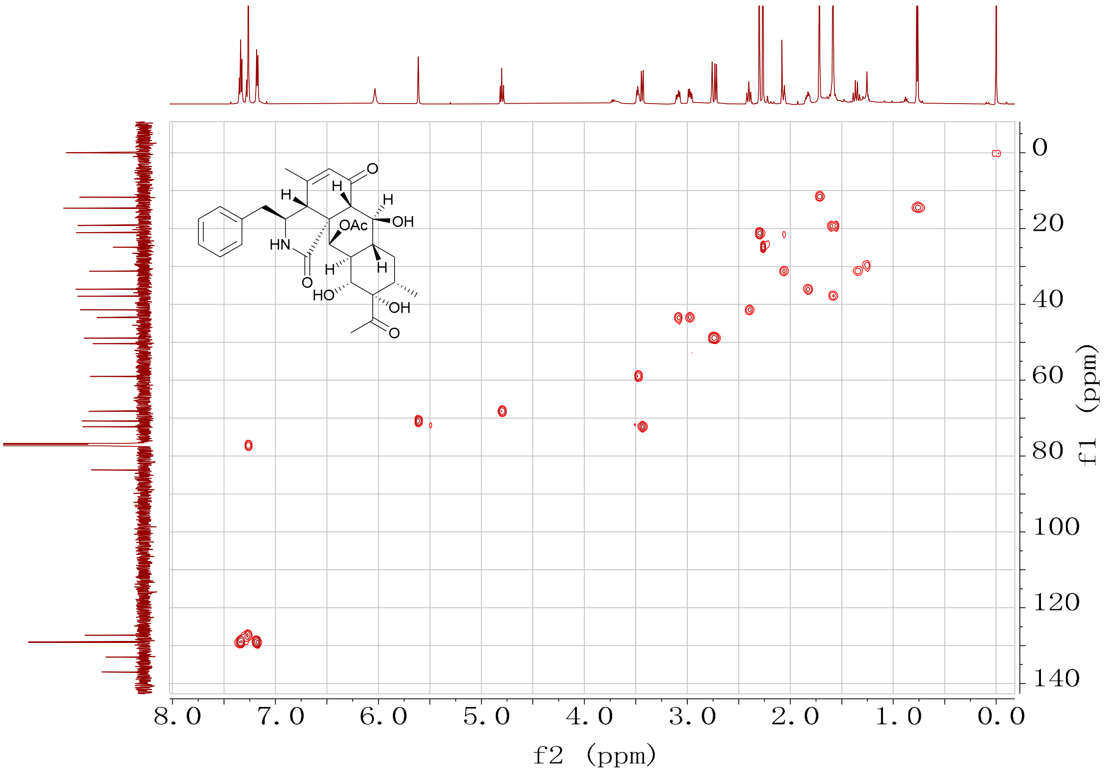


**Figure S91.** HSQC of **8**.

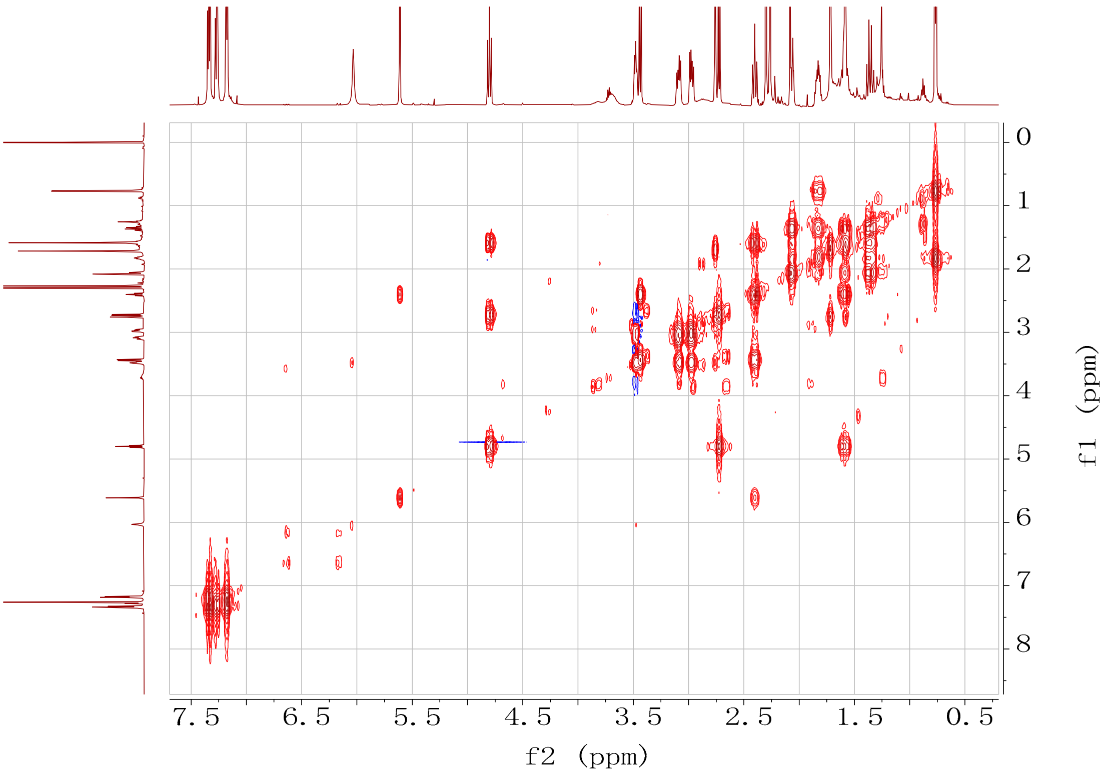


**Figure S92.** $^{1}H-^{1}H$ COSY of **8**.

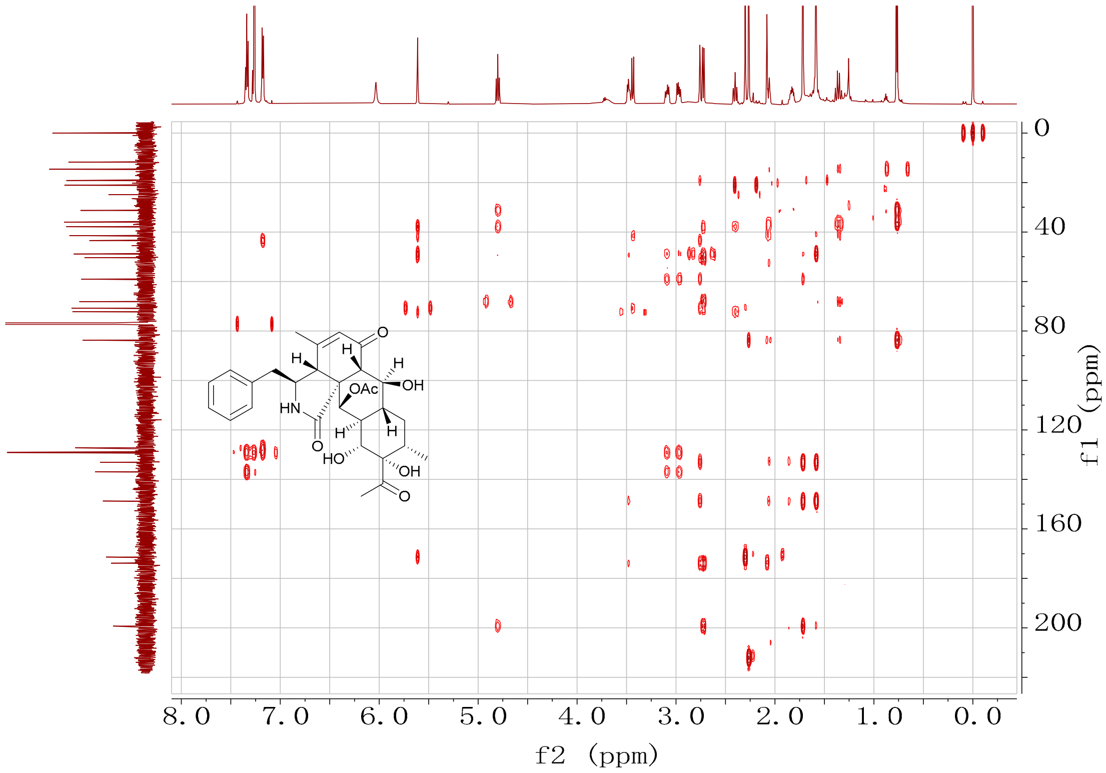


**Figure S93.** HMBC of **8**.

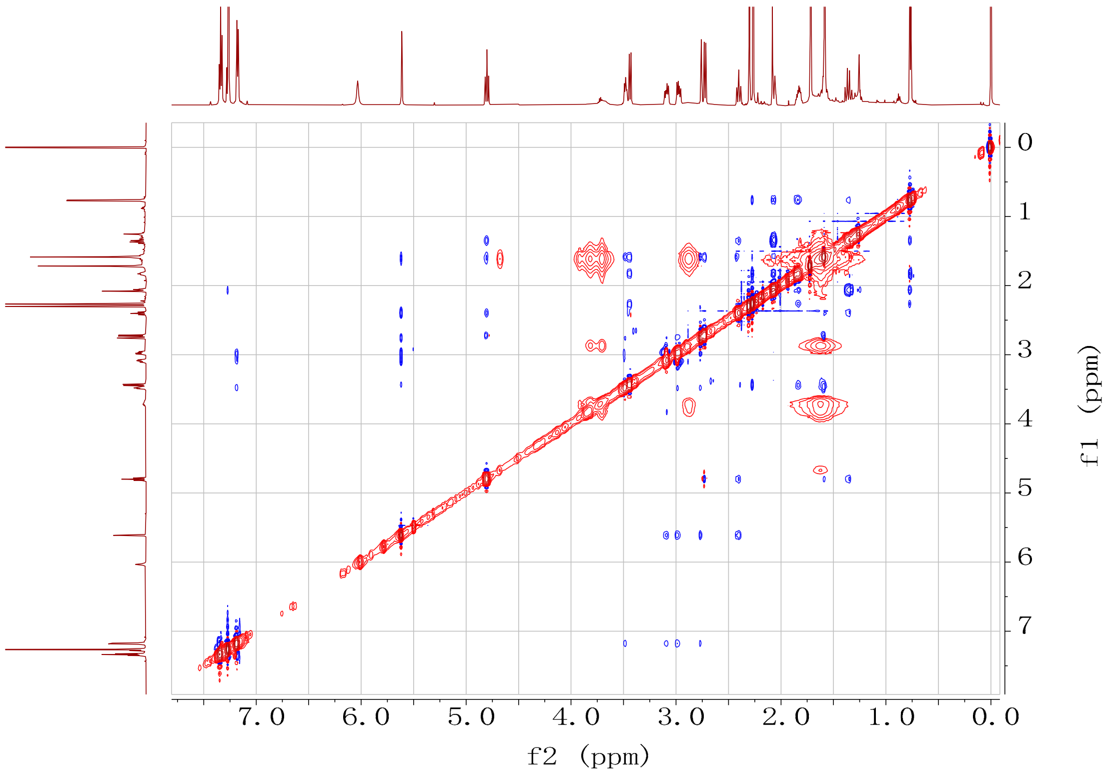


**Figure S94.** NOESY of **8**.

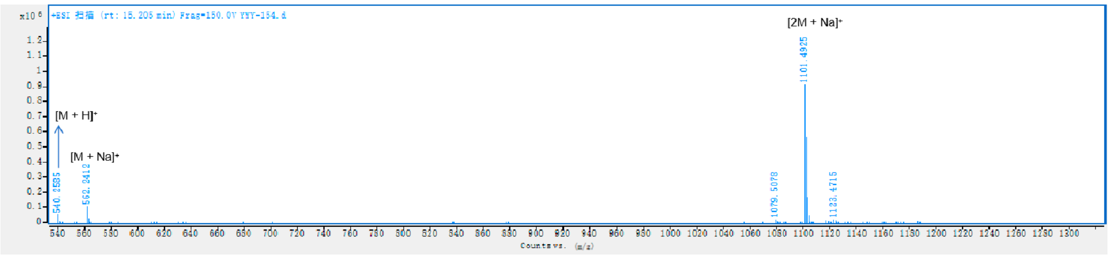


**Figure S95.** HRESIMS of **8**.

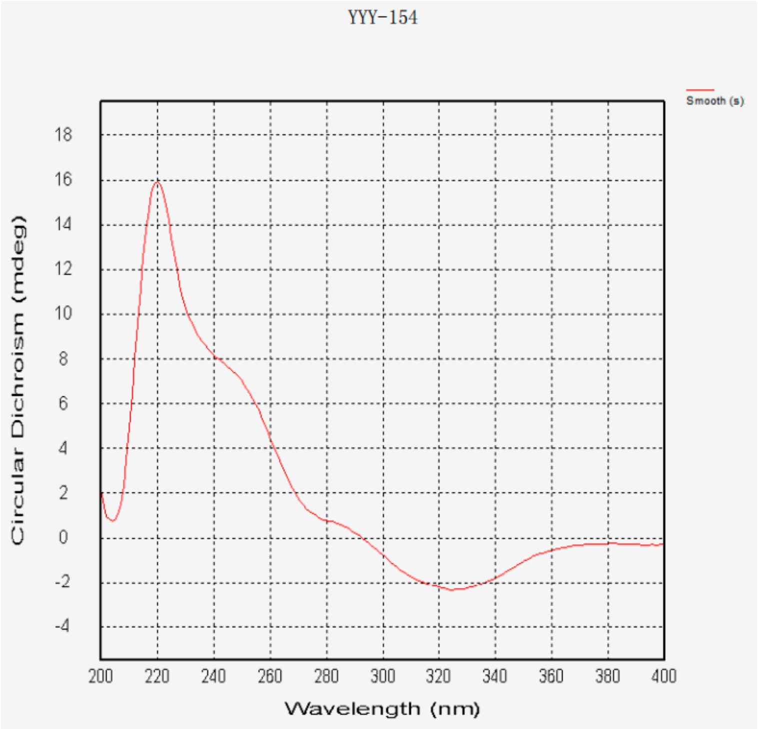


**Figure S96.** CD spectrum (methanol) of **8.**

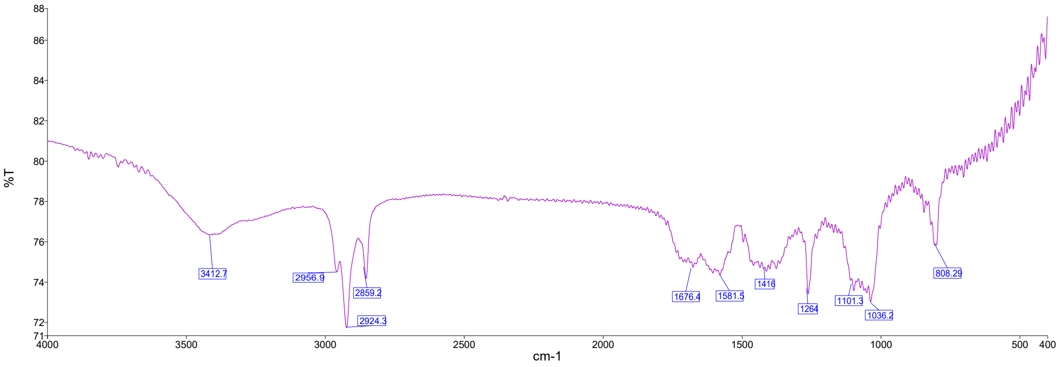


**Figure S97.** IR spectrum of **8.**

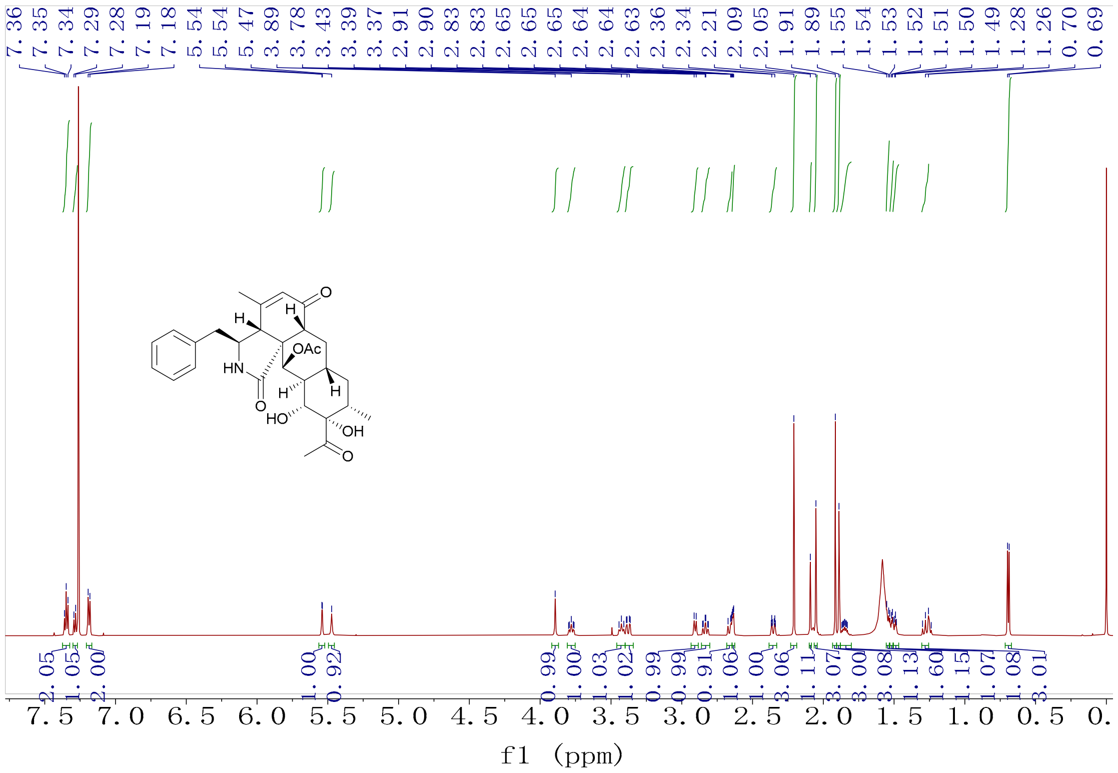

**Figure S98.** $^{1}H$ NMR ($CDCl_3$, 600 MHz) of **9**.

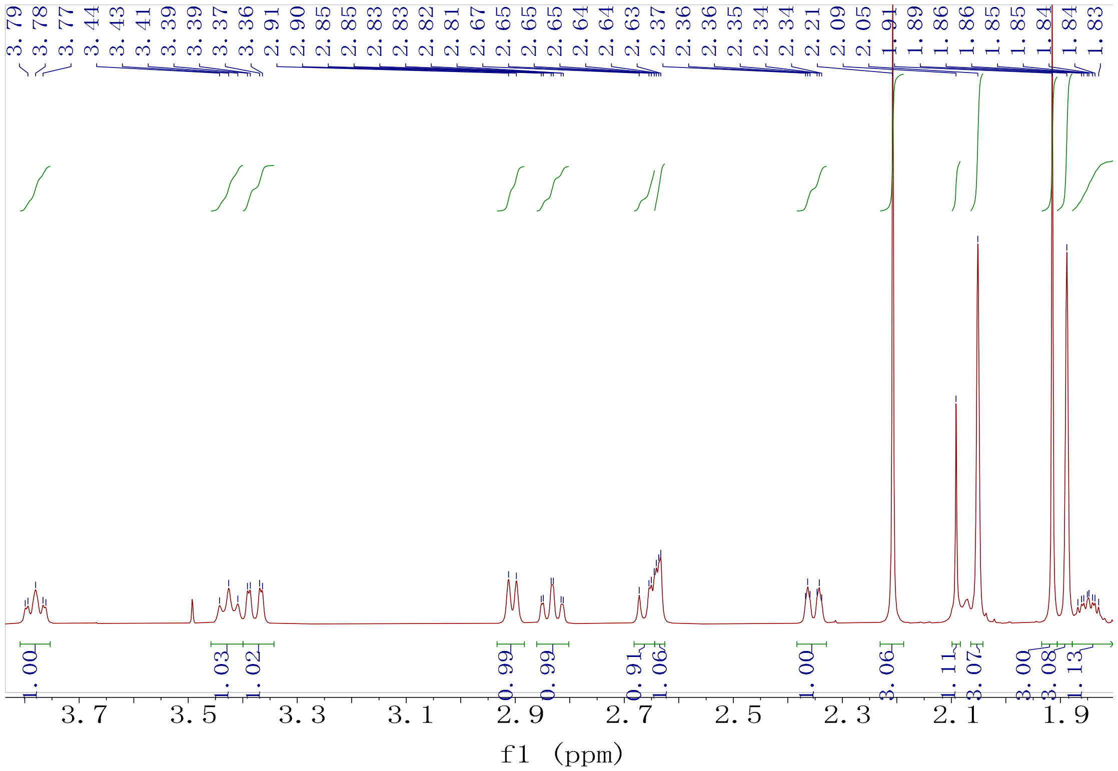

**Figure S99.** Partial $^{1}H$ NMR ($CDCl_3$, 600 MHz) of **9**.

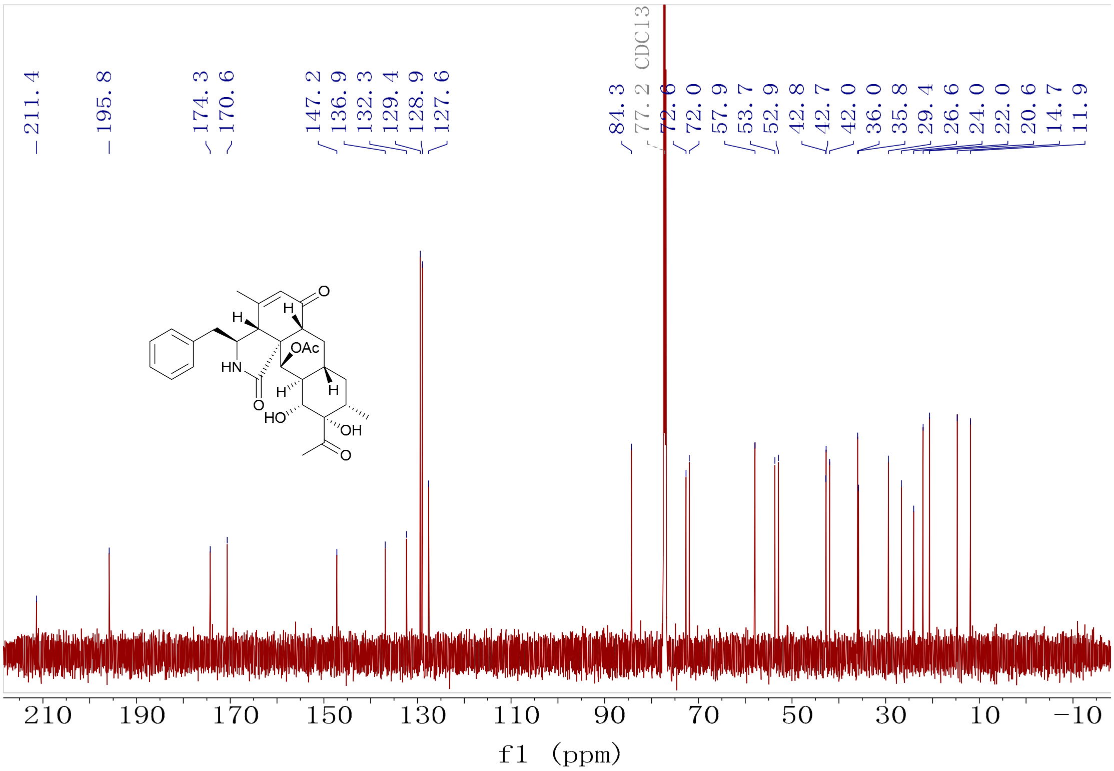


**Figure S100.** $^{13}C$ NMR ($CDCl_3$, 150 MHz) of **9**.

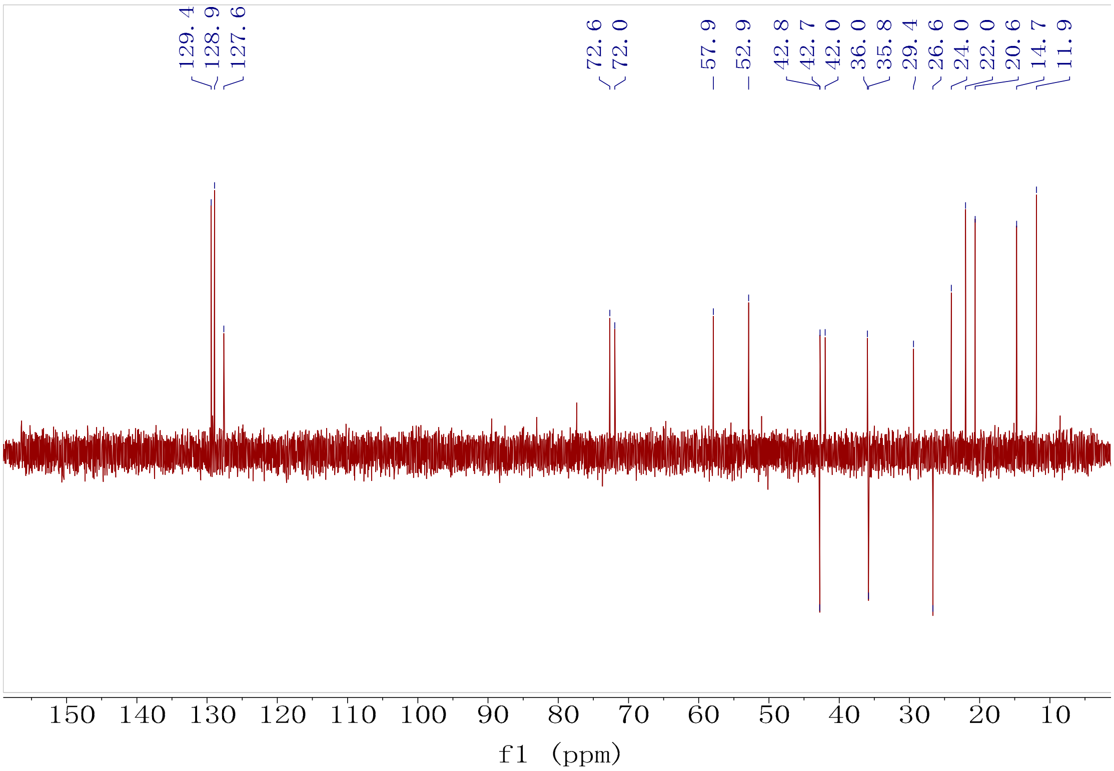


**Figure S101.** DEPT 135 spectra of **9**.

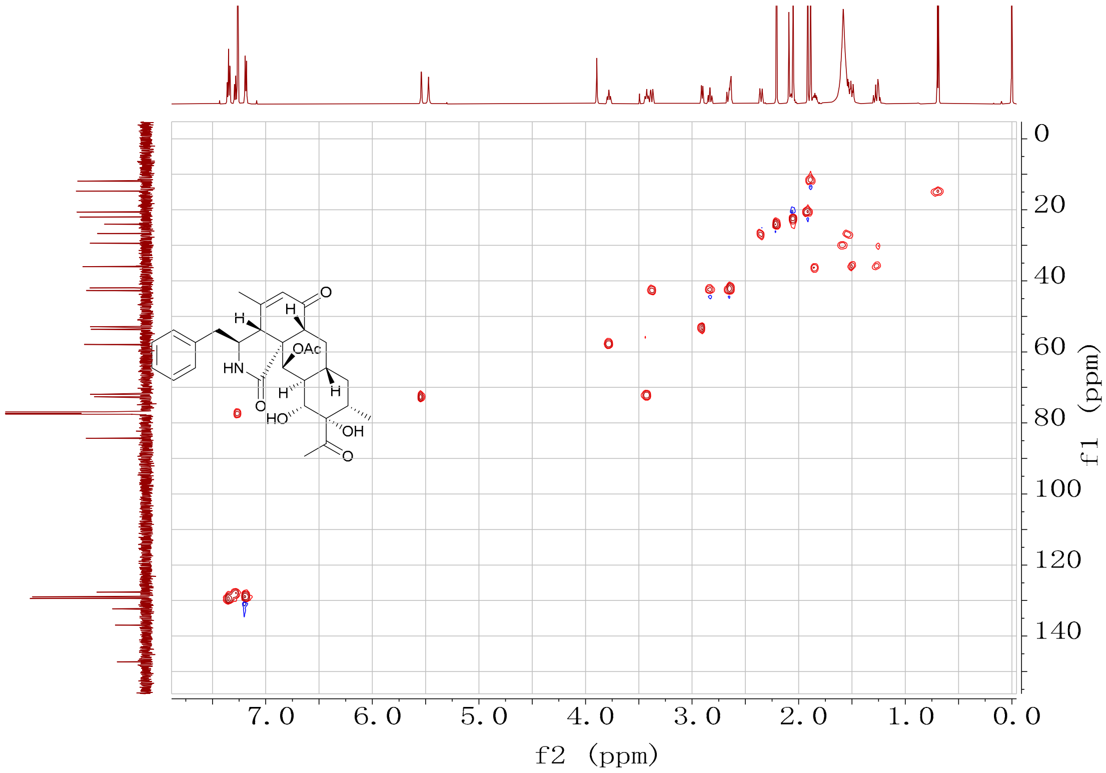


**Figure S102.** HSQC of **9**.

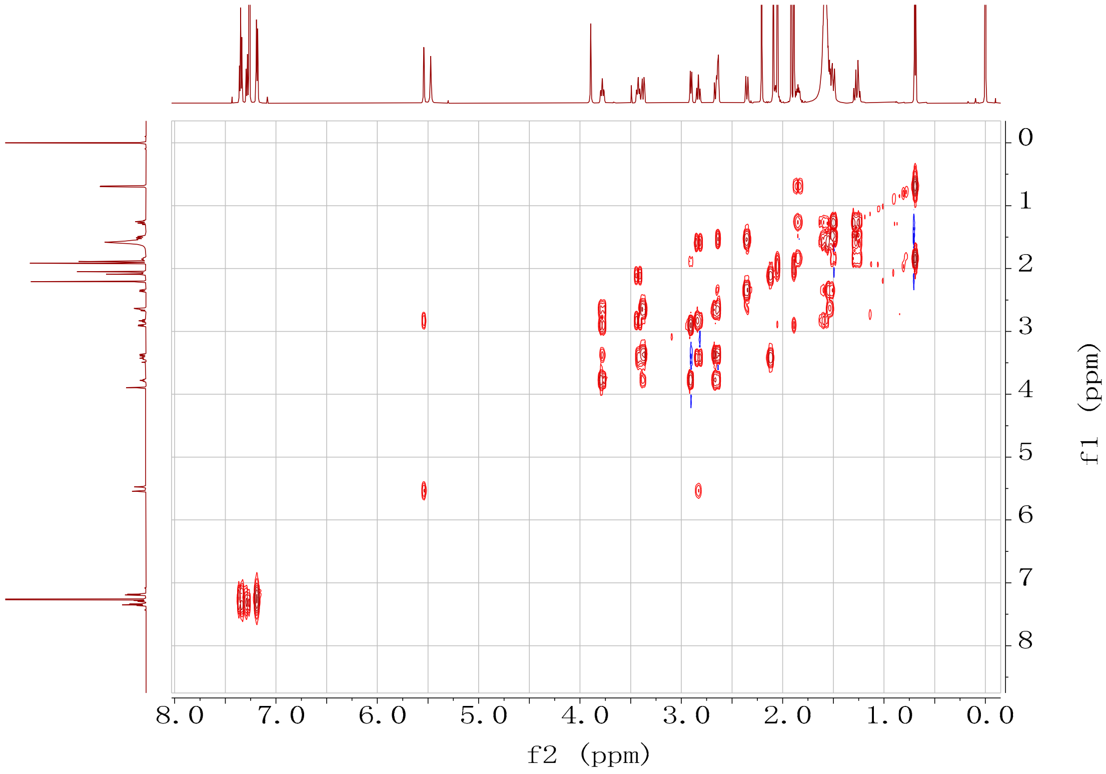


**Figure S103.** $^{1}$H–$^{1}$H COSY of **9**.

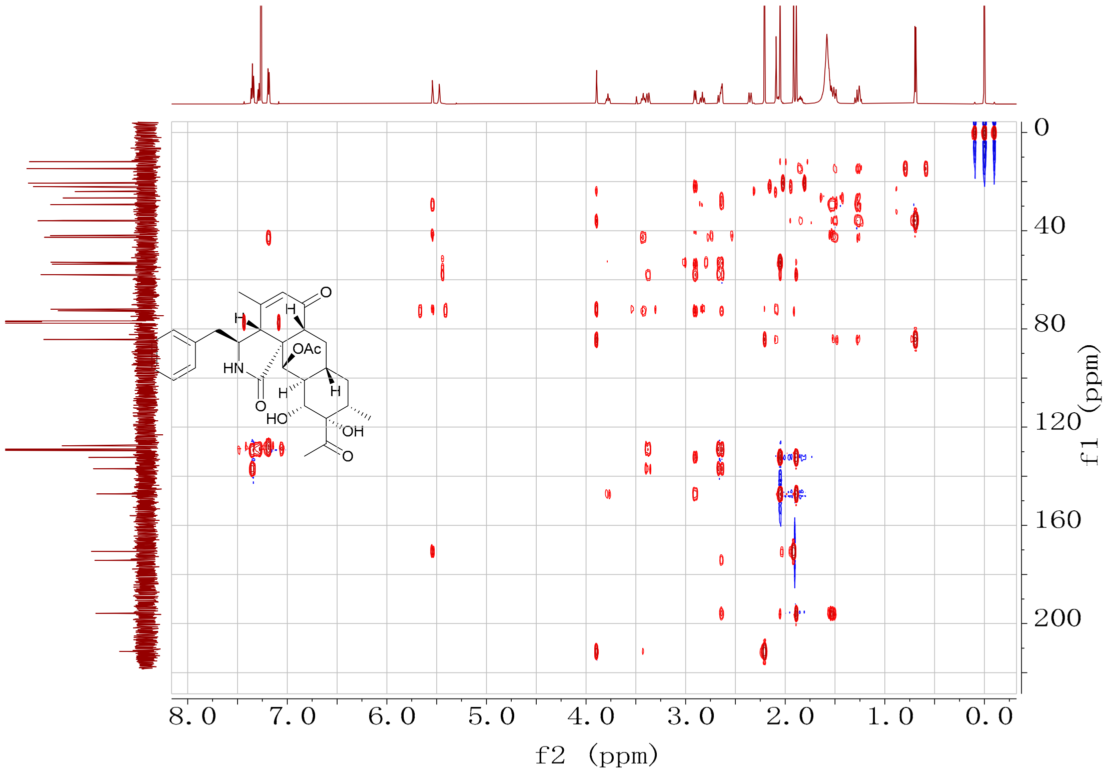


**Figure S104.** HMBC of **9**.

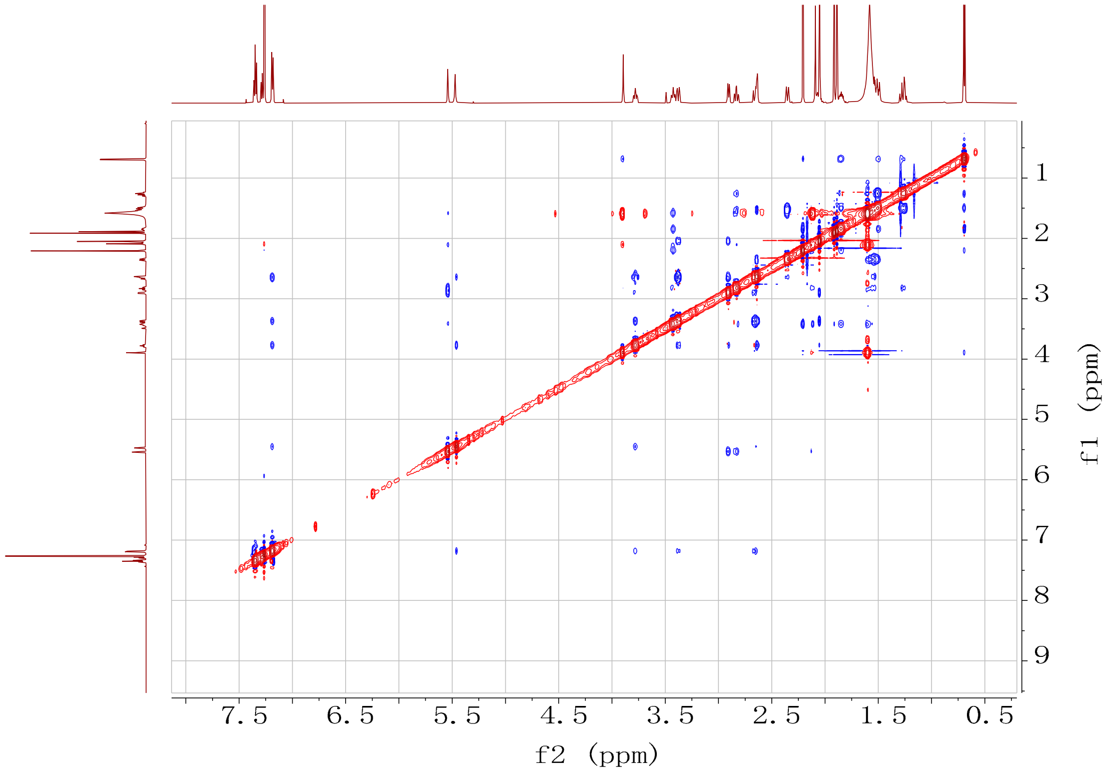


**Figure S105.** NOESY of **9**.

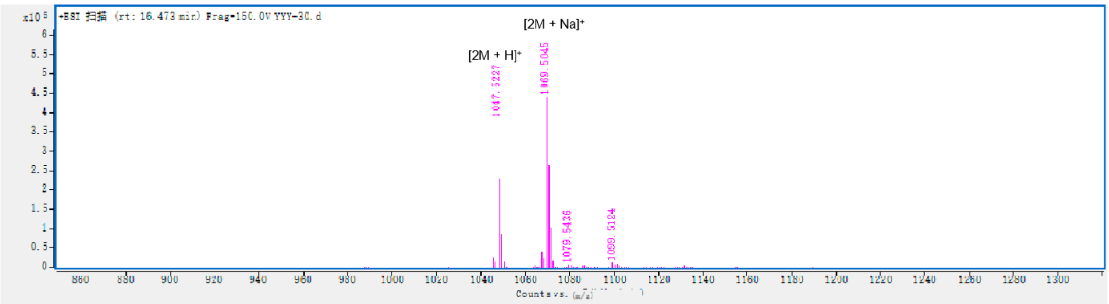


**Figure S106.** HRESIMS of **9**.

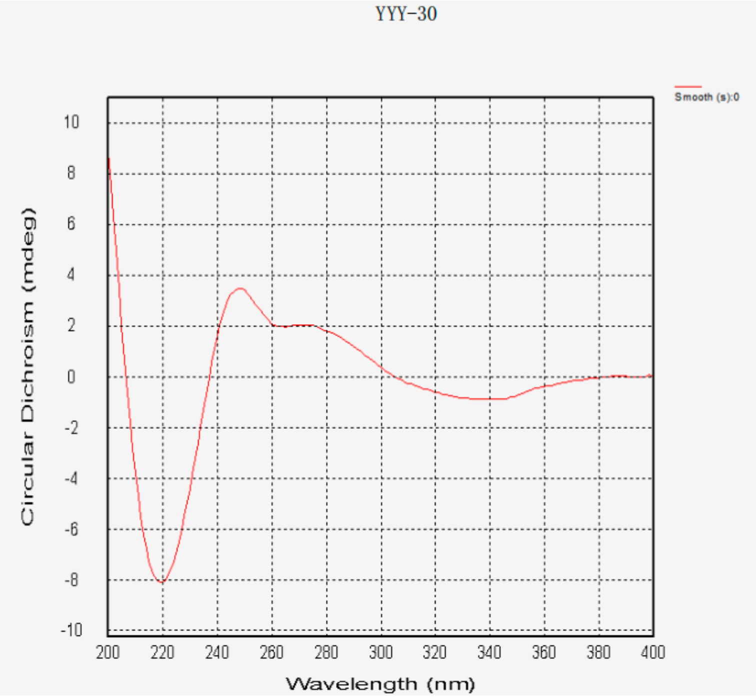


**Figure S107.** CD spectrum (methanol) of **9.**

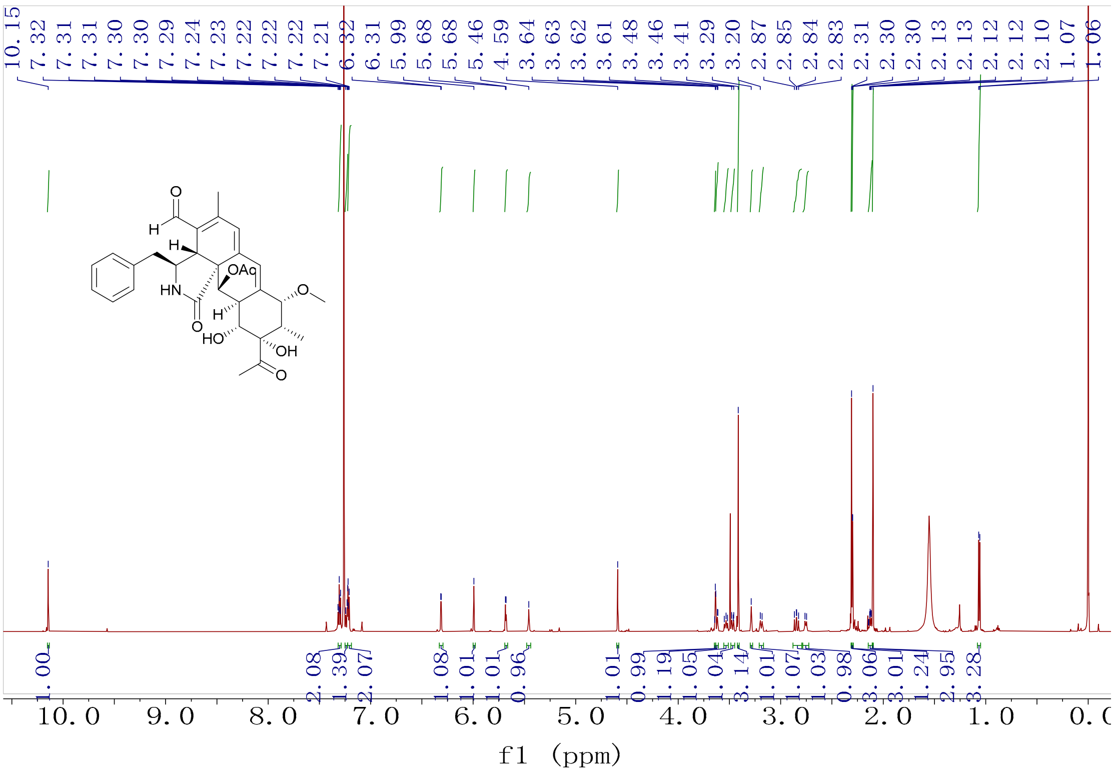

**Figure S108.** $^{1}H$ NMR ($CDCl_3$, 600 MHz) of **10**.

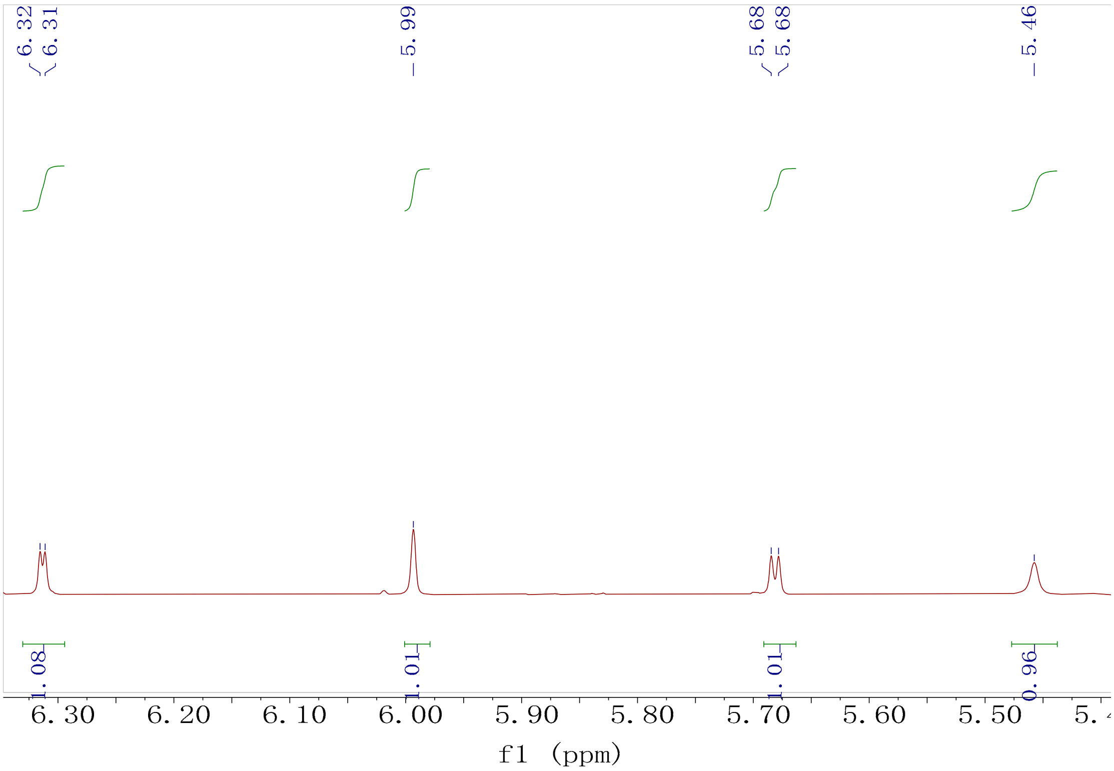

**Figure S109.** Partial $^{1}H$ NMR ($CDCl_3$, 600 MHz) of **10**.

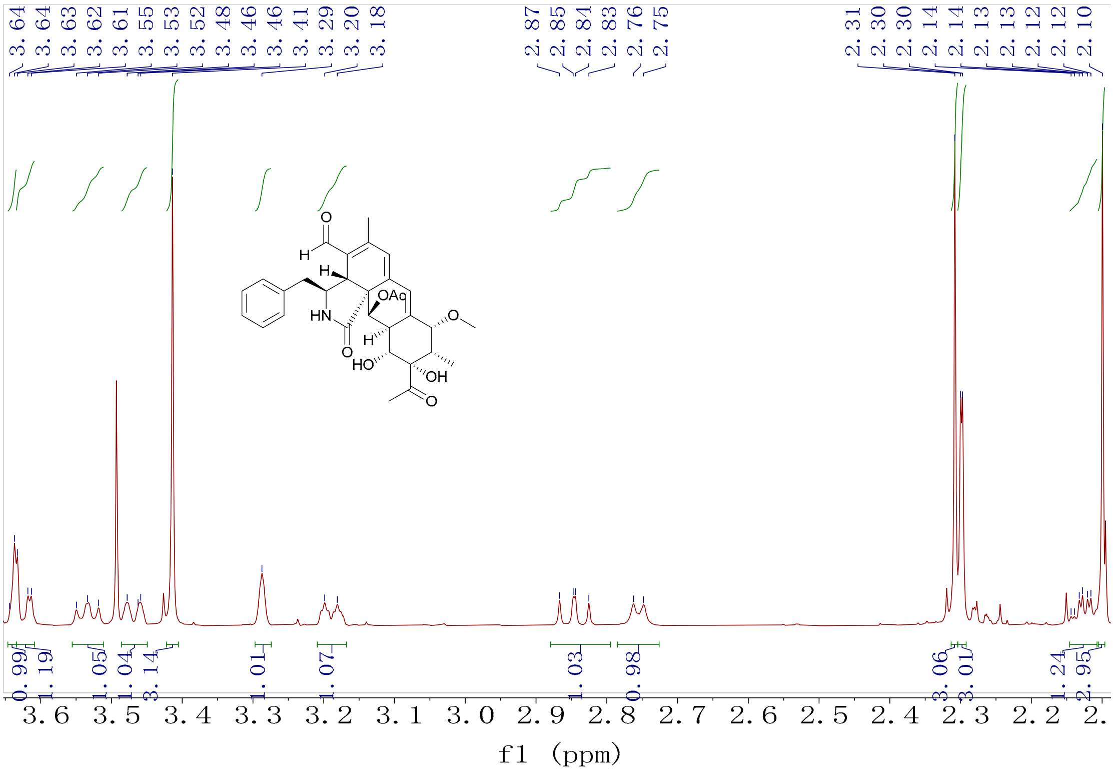


**Figure S110.** Partial $^{1}H$ NMR ($CDCl_3$, 600 MHz) of **10**.

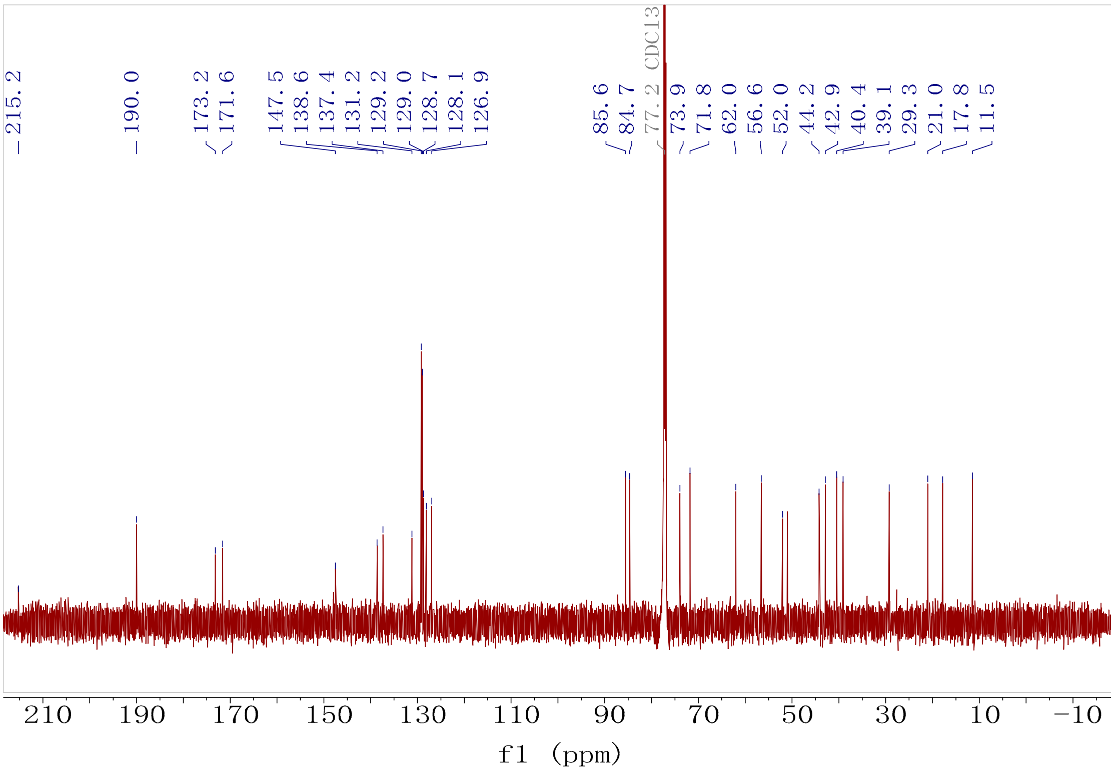


**Figure S111.** $^{13}C$ NMR ($CDCl_3$, 150 MHz) of **10**.

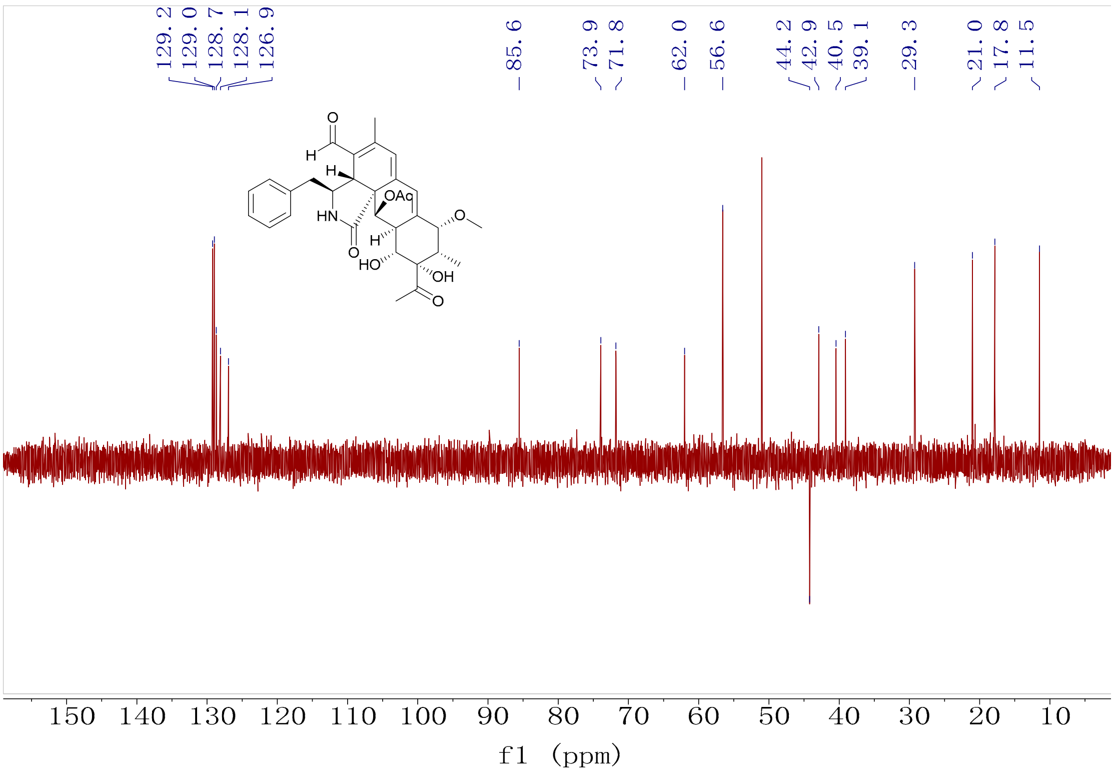


**Figure S112.** DEPT 135 spectra of **10**.

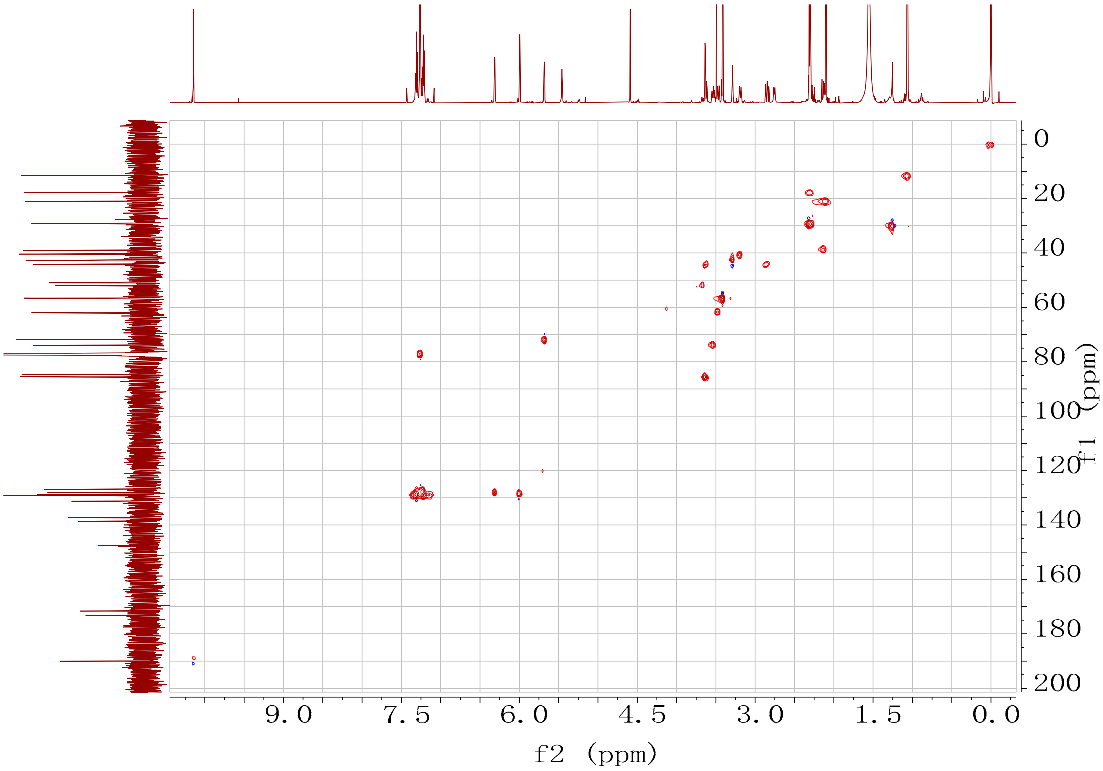


**Figure S113.** HSQC of **10**.

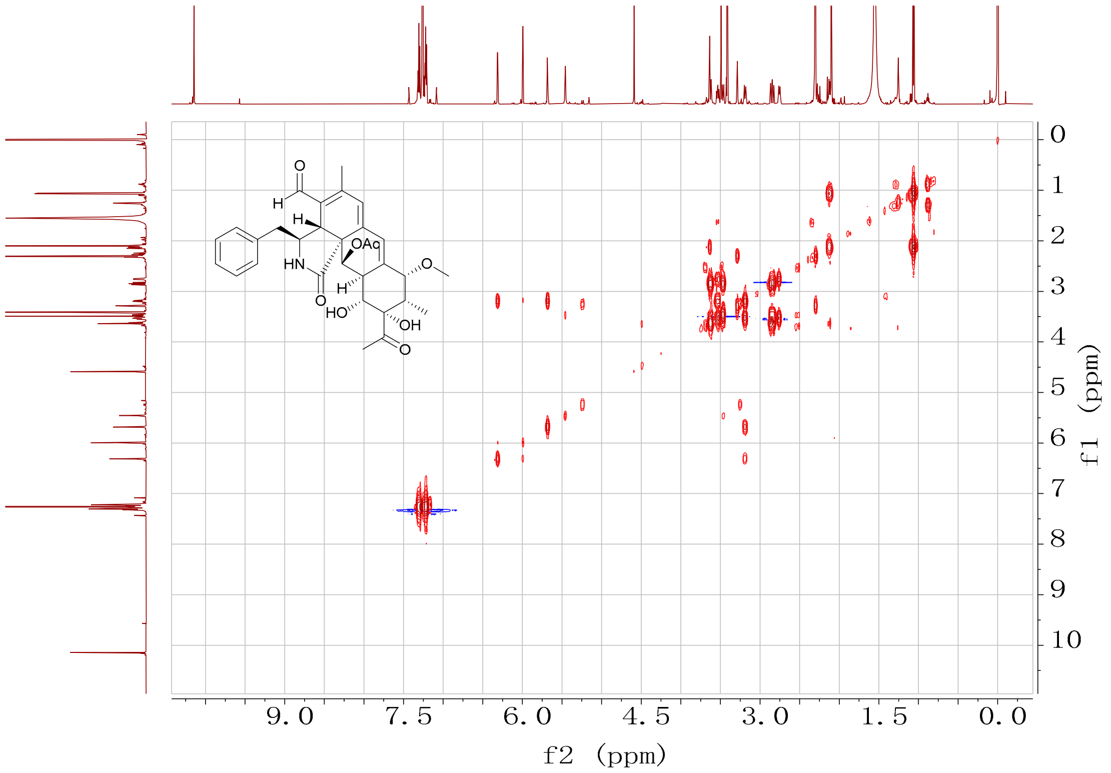


**Figure S114.** $^{1}H-^{1}H$ COSY of **10**.

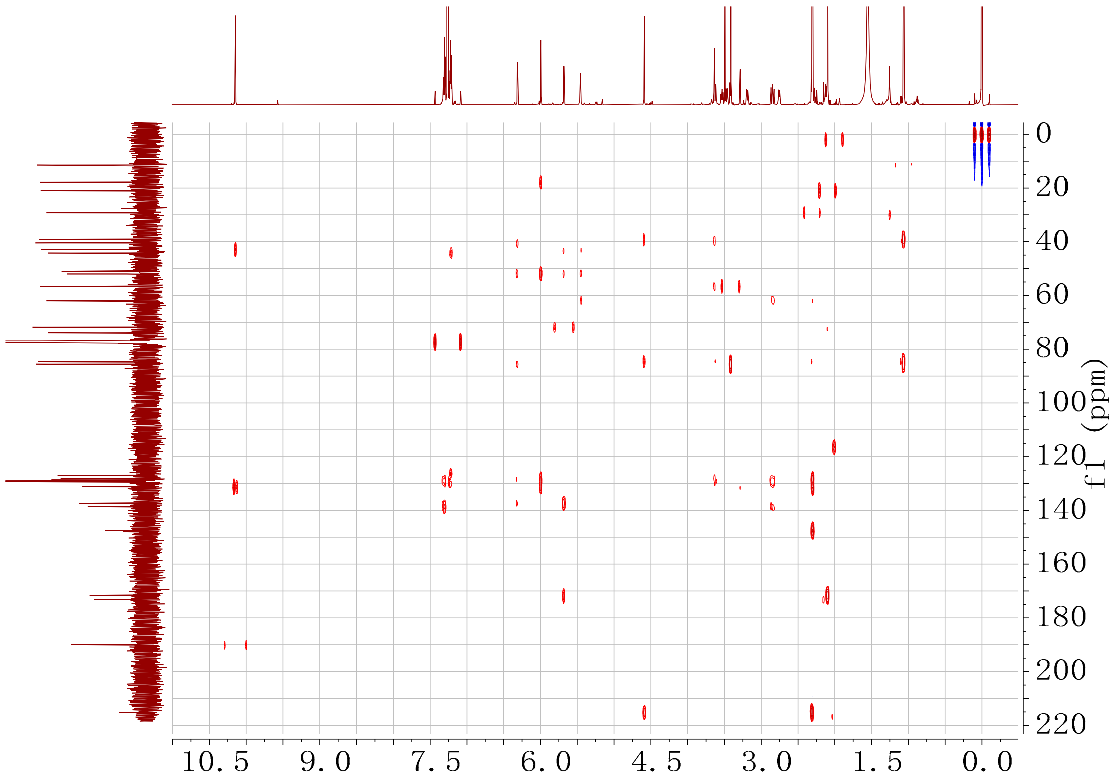


**Figure S115.** HMBC of **10**.

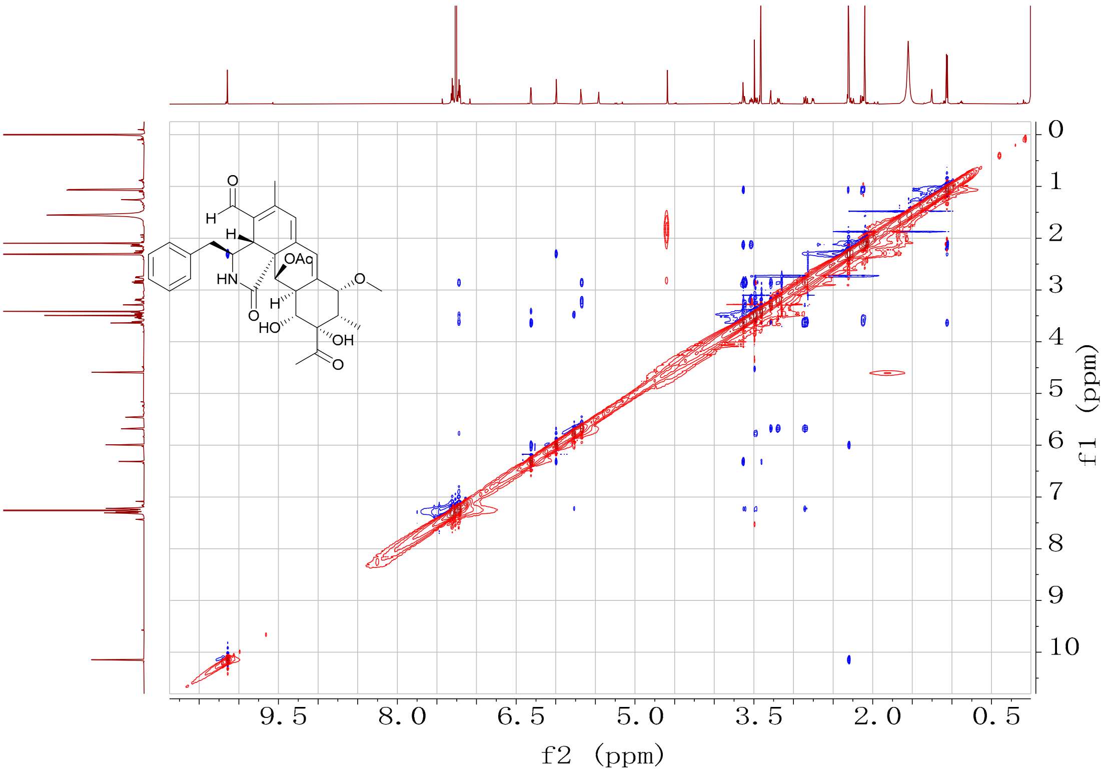


**Figure S116.** NOESY of **10**.

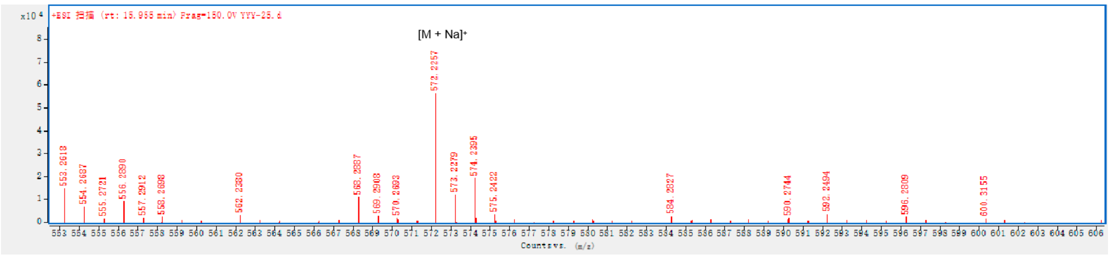


**Figure S117.** HRESIMS of **10**.

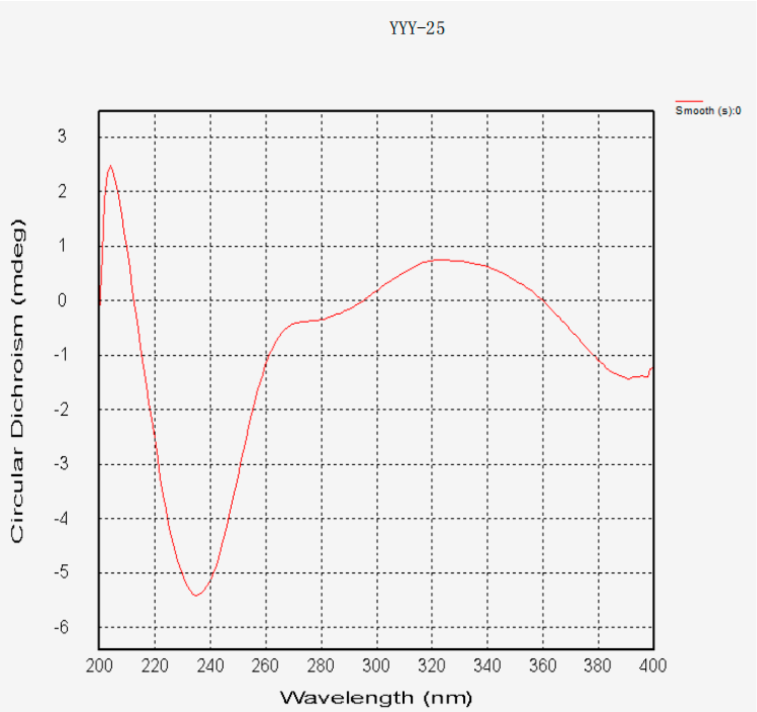


**Figure S118.** CD spectrum (methanol) of **10.**

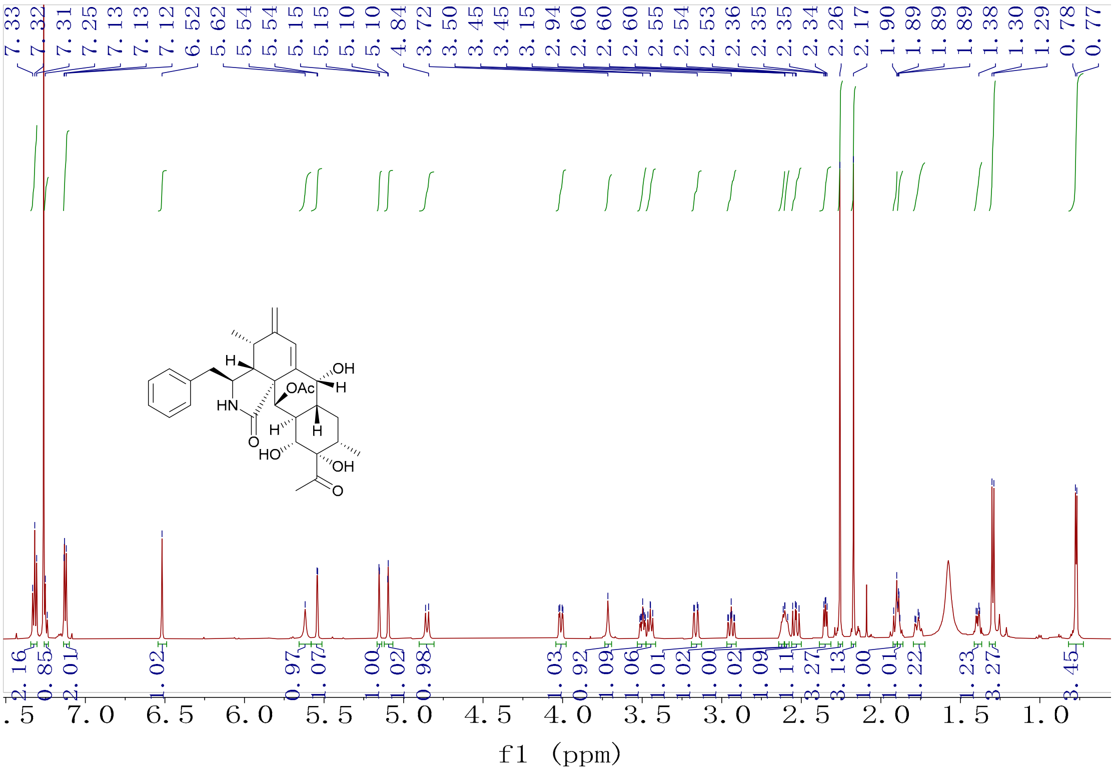


**Figure S119.** $^{1}$H NMR ($CDCl_3$, 600 MHz) of **11**.

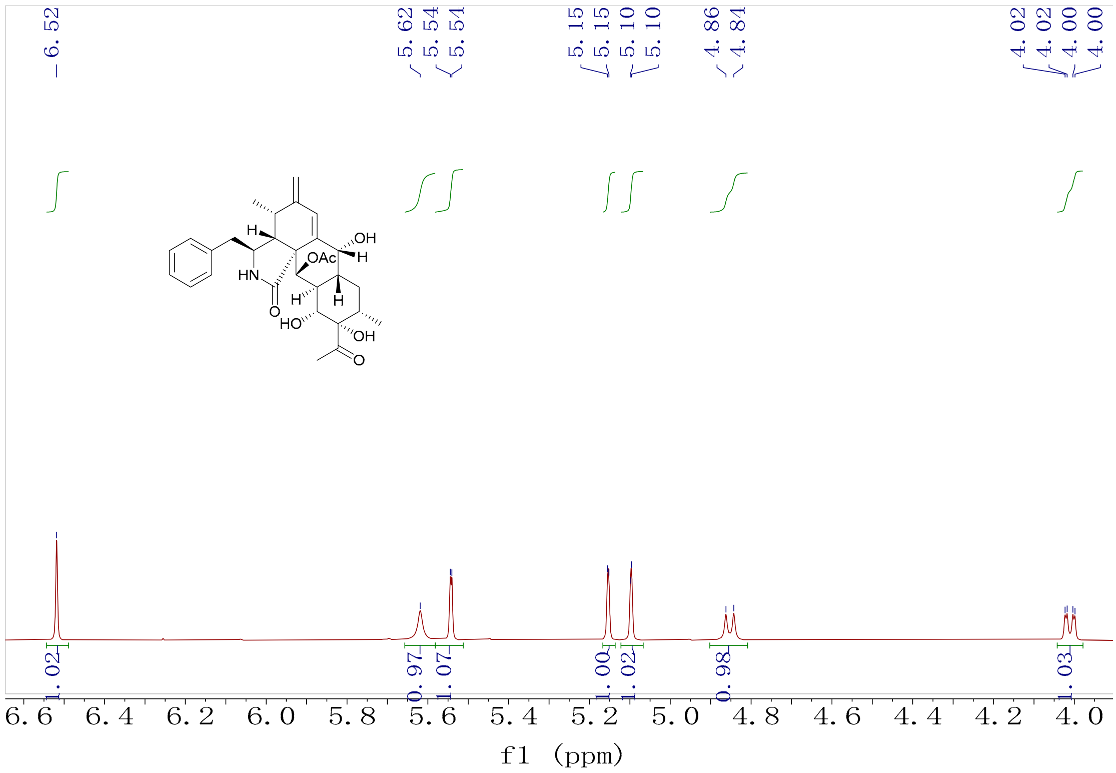


**Figure S120.** Partial $^1$H NMR ($CDCl_3$, 600 MHz) of **11**.

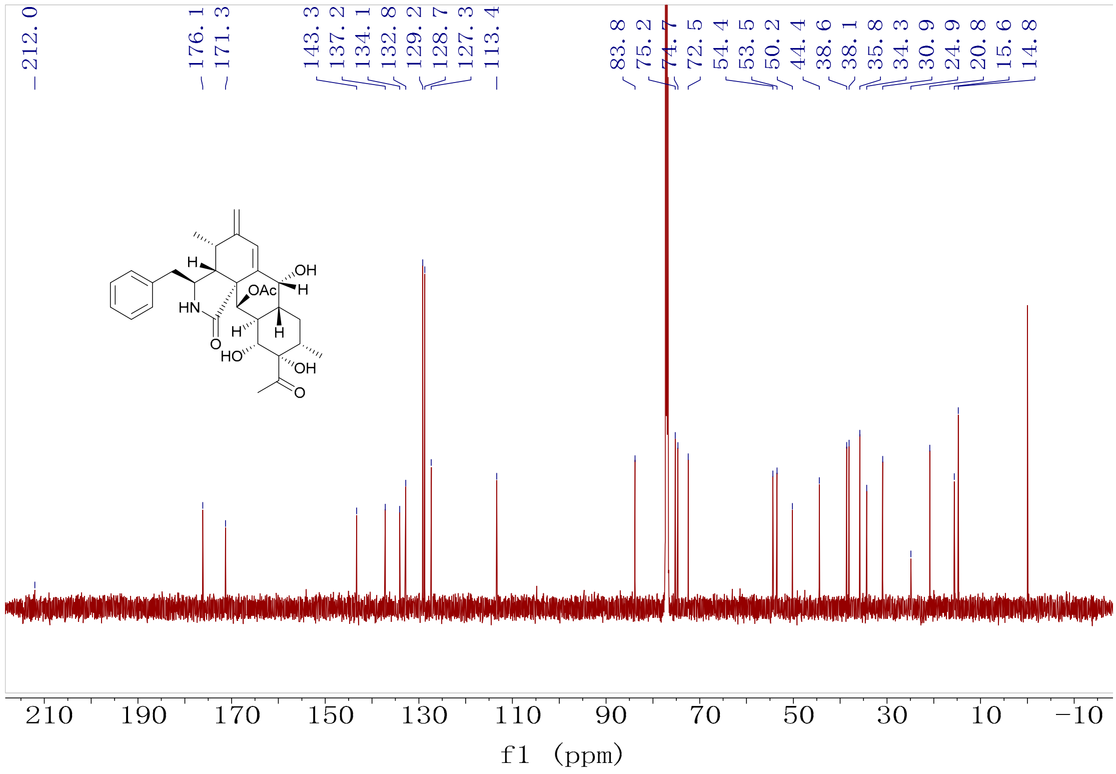


**Figure S121.** $^{13}$C NMR ($CDCl_3$, 150 MHz) of **11**.

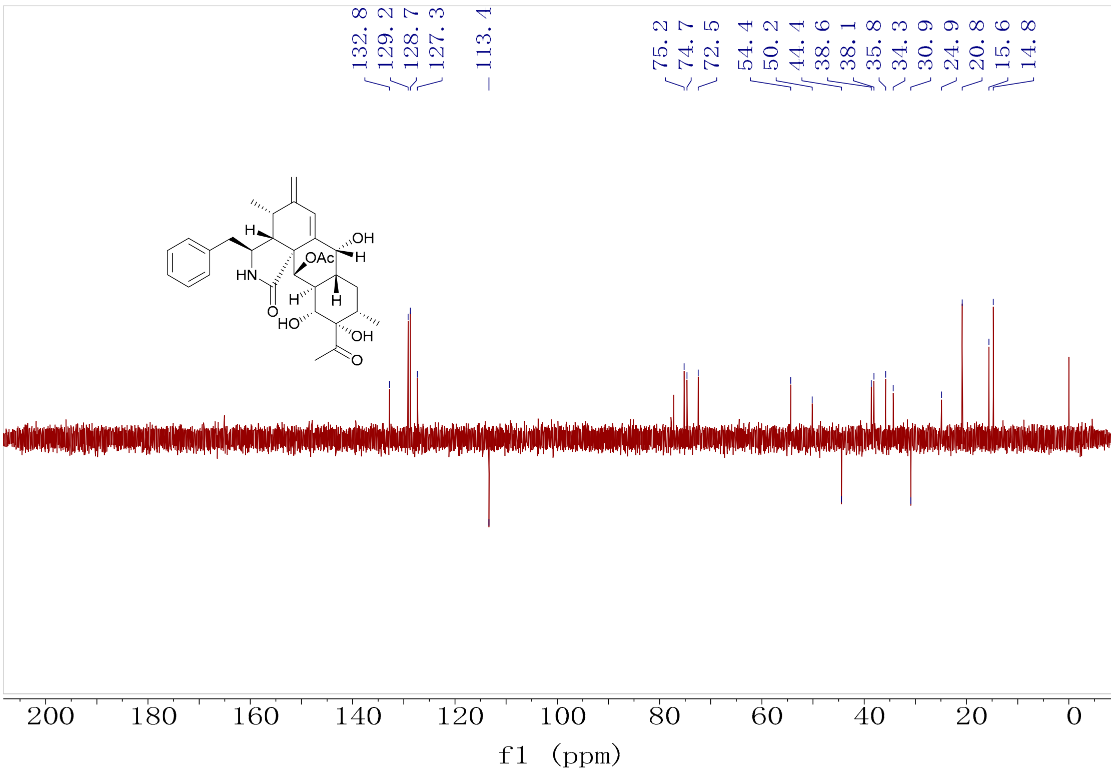


**Figure S122.** DEPT 135 spectra of **11**.

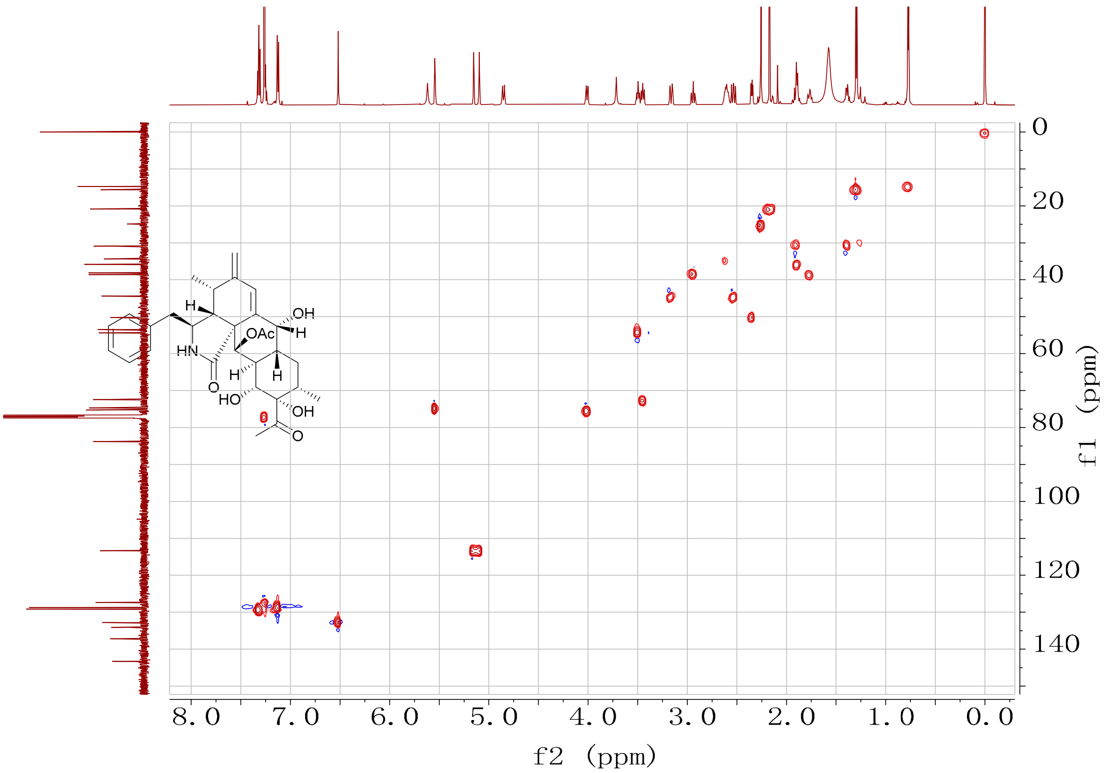


**Figure S123.** HSQC of **11**.

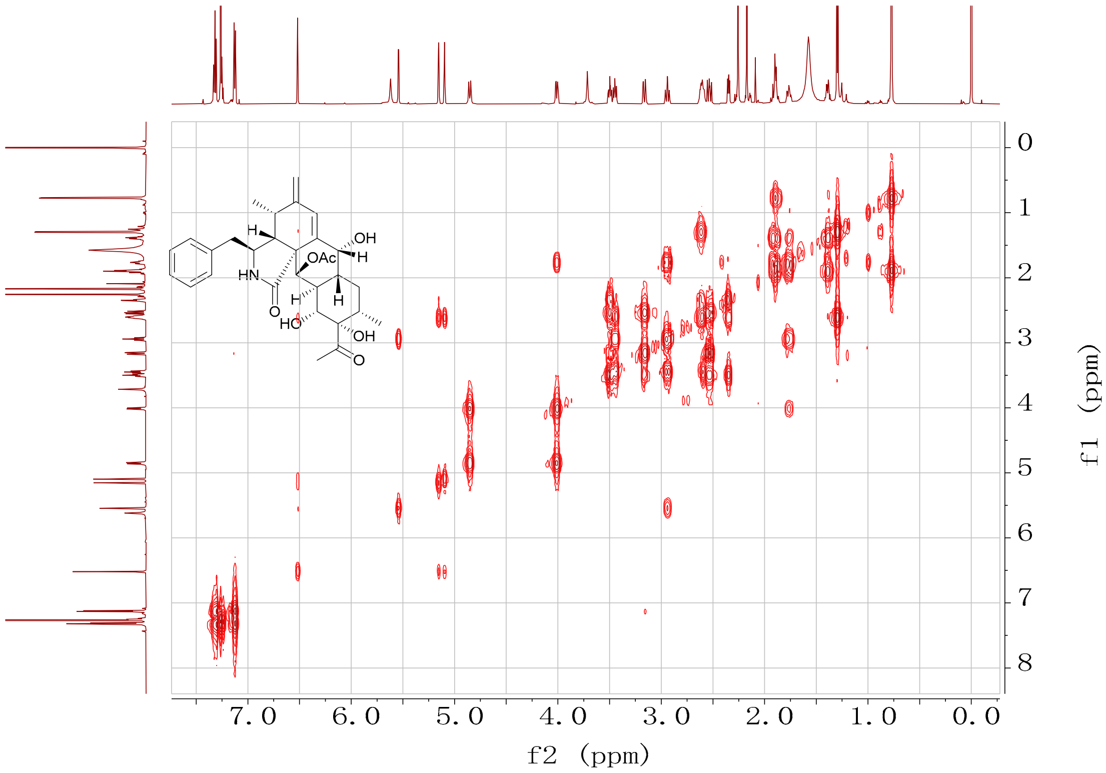


**Figure S124.** $^{1}H$–$^{1}H$ COSY of **11**.

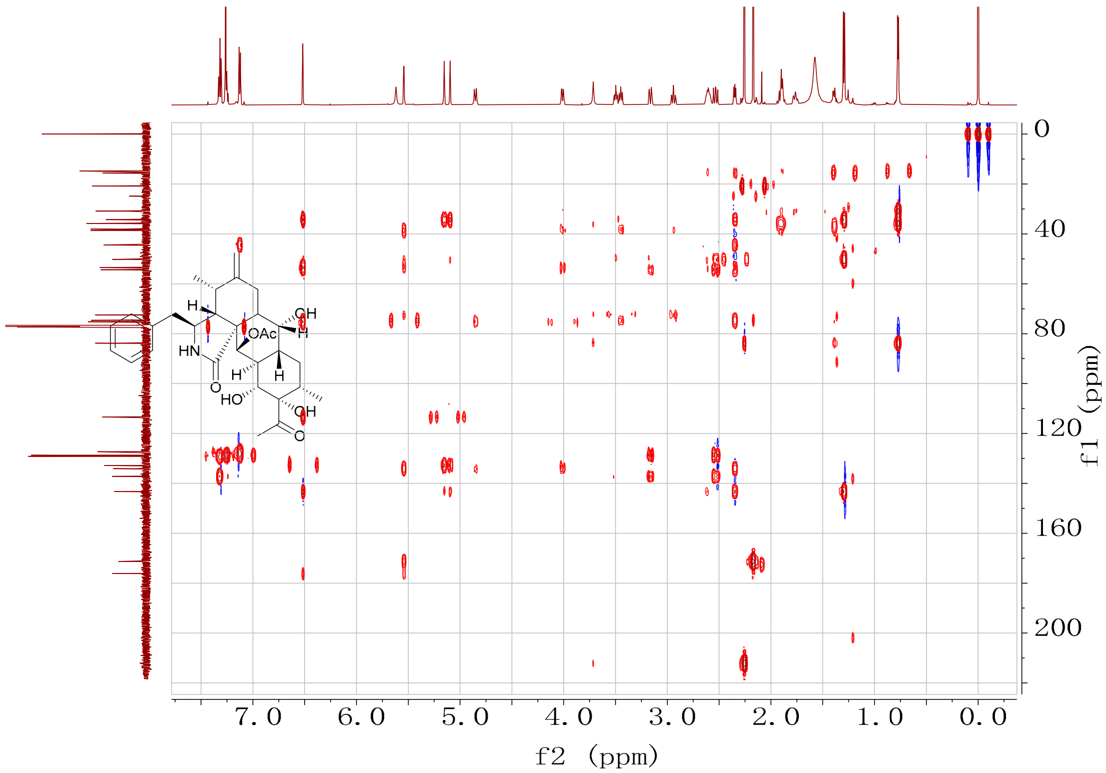


**Figure S125.** HMBC of **11**.

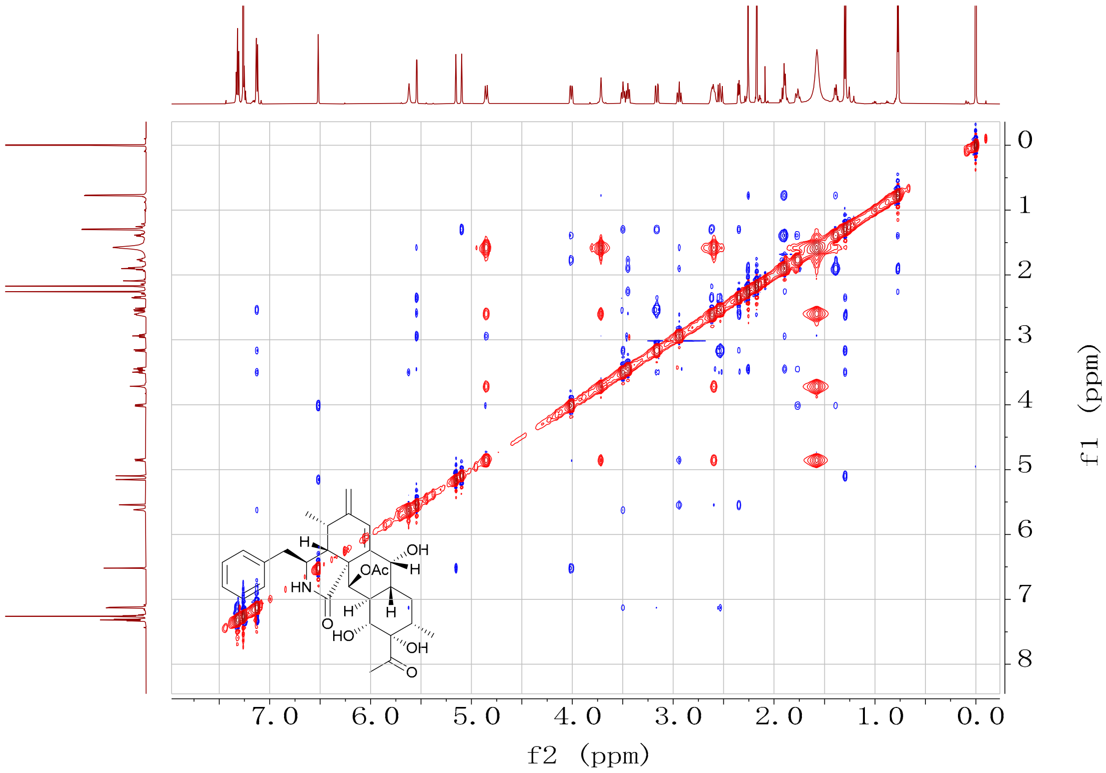


**Figure S126.** NOESY of **11**.

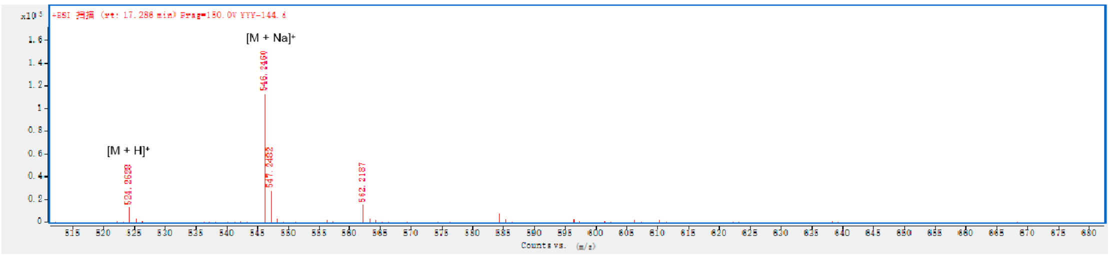


**Figure S127.** HRESIMS of **11**.

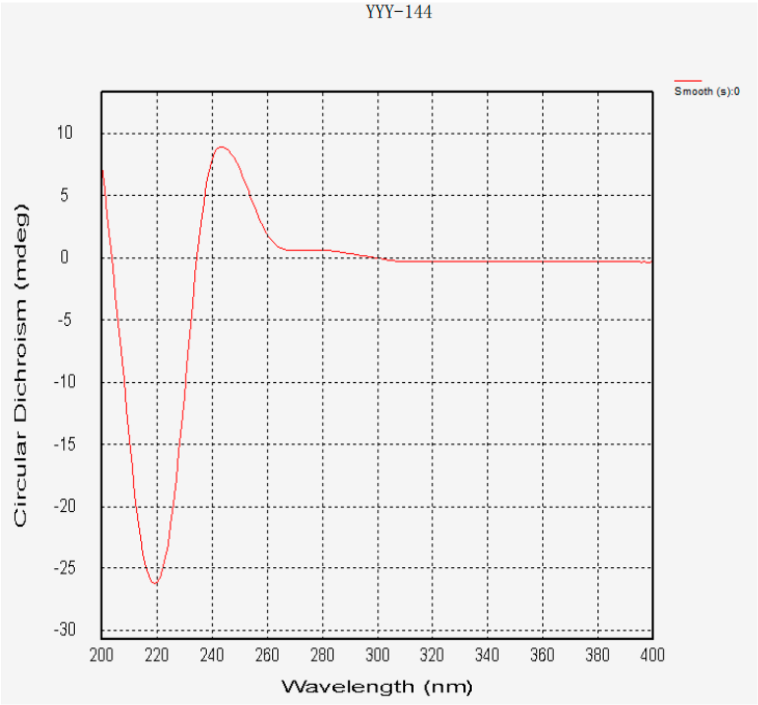


**Figure S128.** CD spectrum (acetonitrile) of **11.**

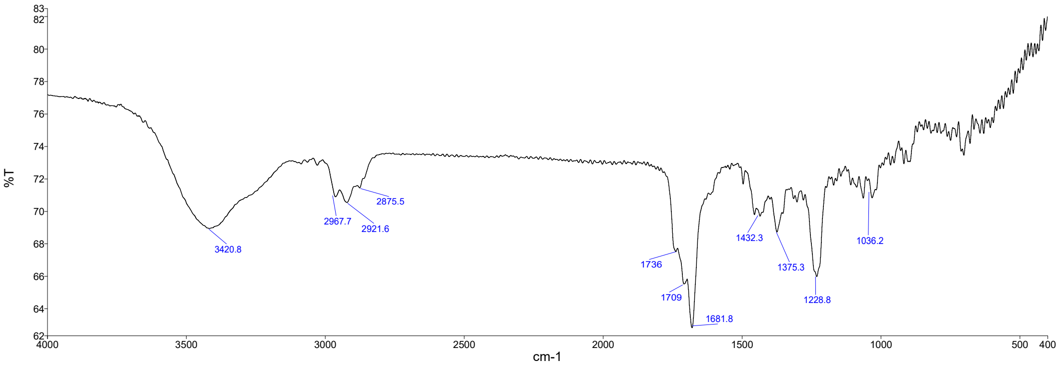


**Figure S129.** IR spectrum of **11.**

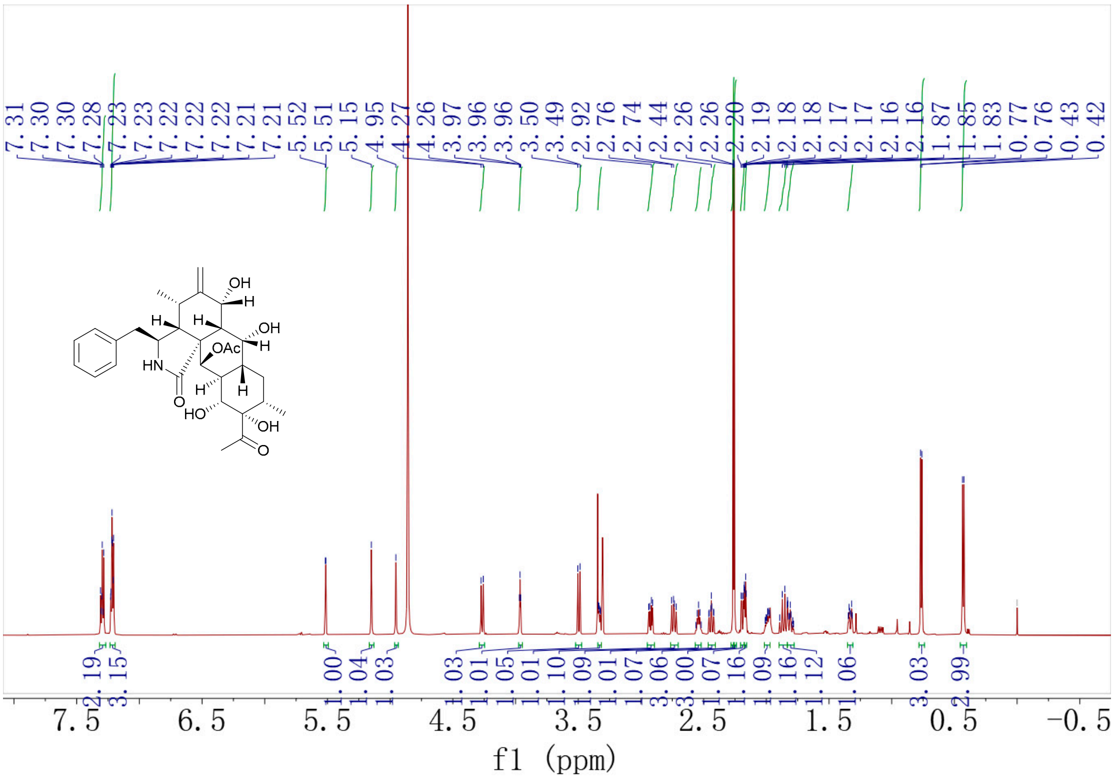

**Figure S130.** $^{1}H$ NMR ($CD_3OD$, 600 MHz) of **12**.

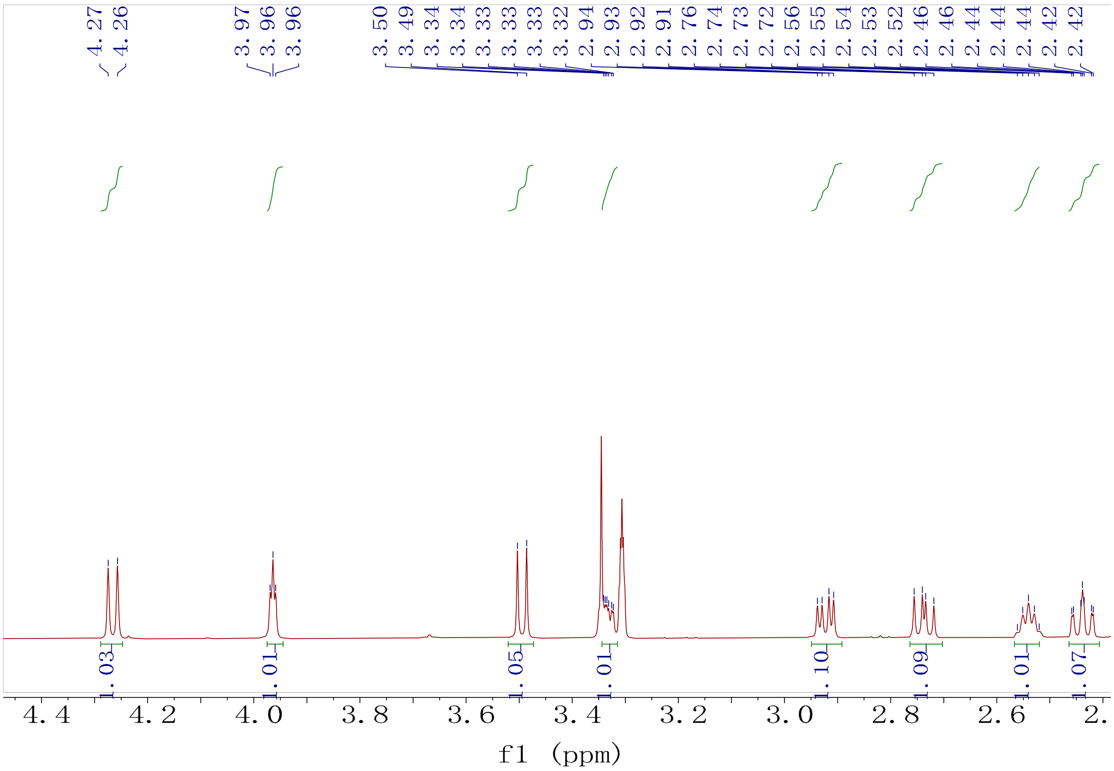

**Figure S131.** Partial $^{1}H$ NMR ($CDCl_3$, 600 MHz) of **12**.

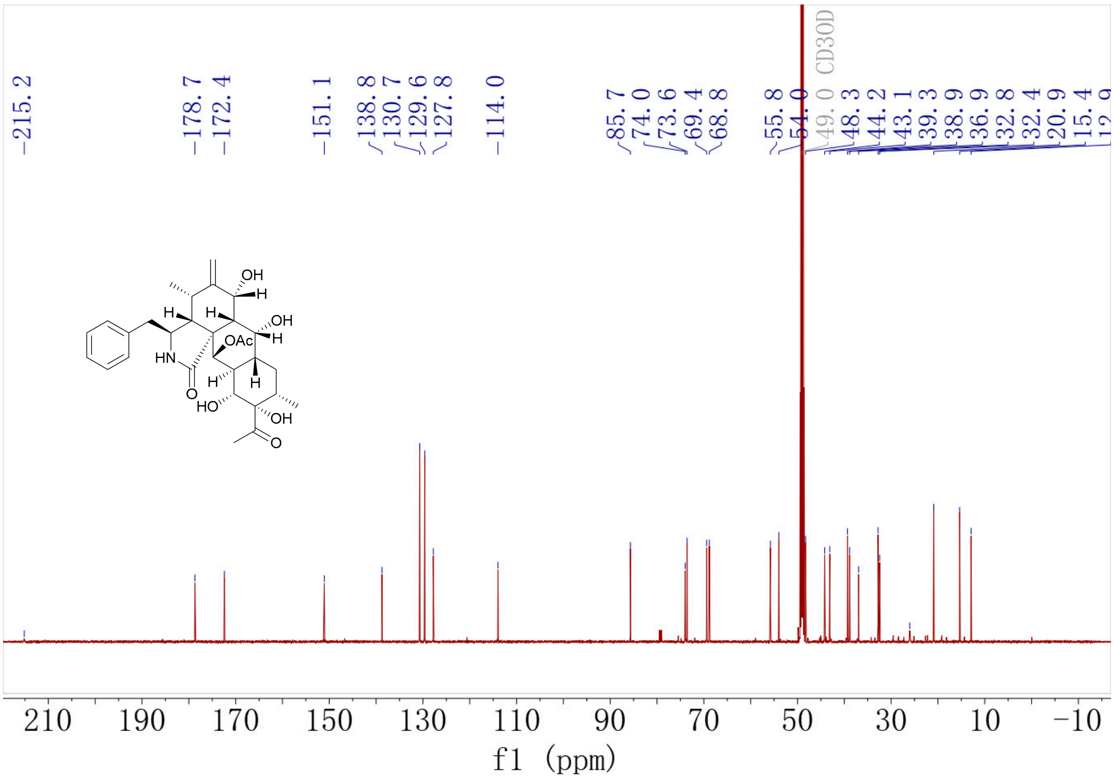


**Figure S132.** $^{13}C$ NMR ($CD_3OD$, 150 MHz) of **12**.

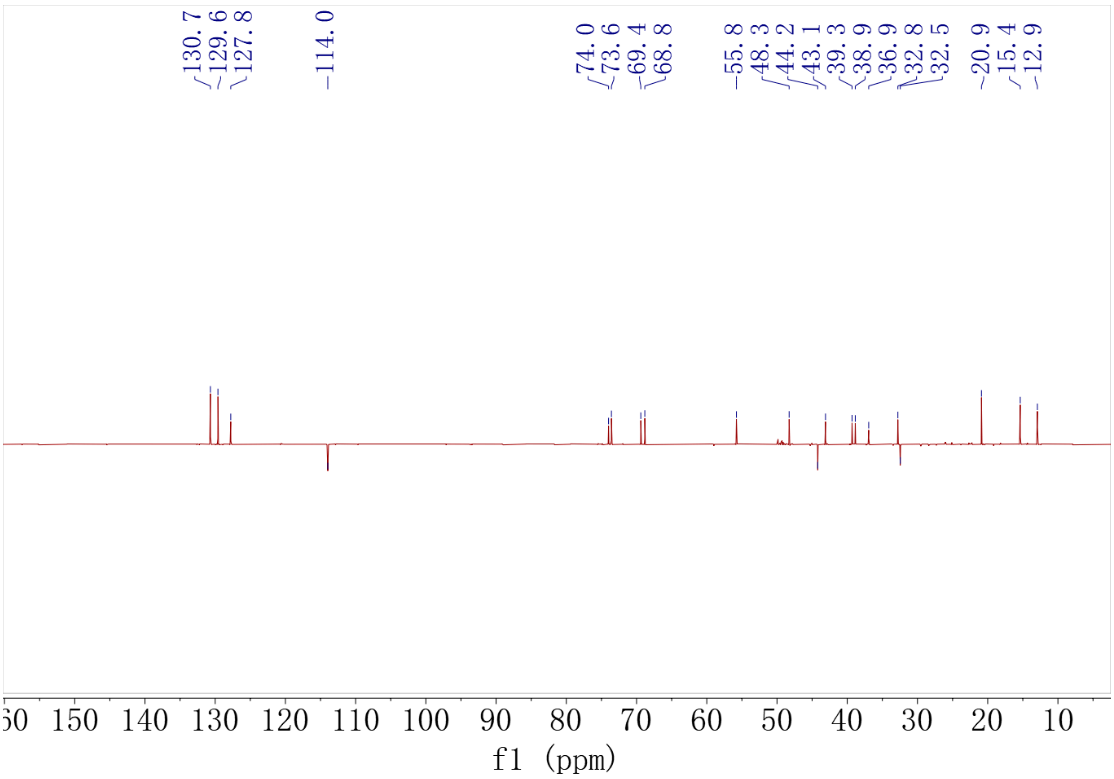


**Figure S133.** DEPT 135 spectra of **12**.

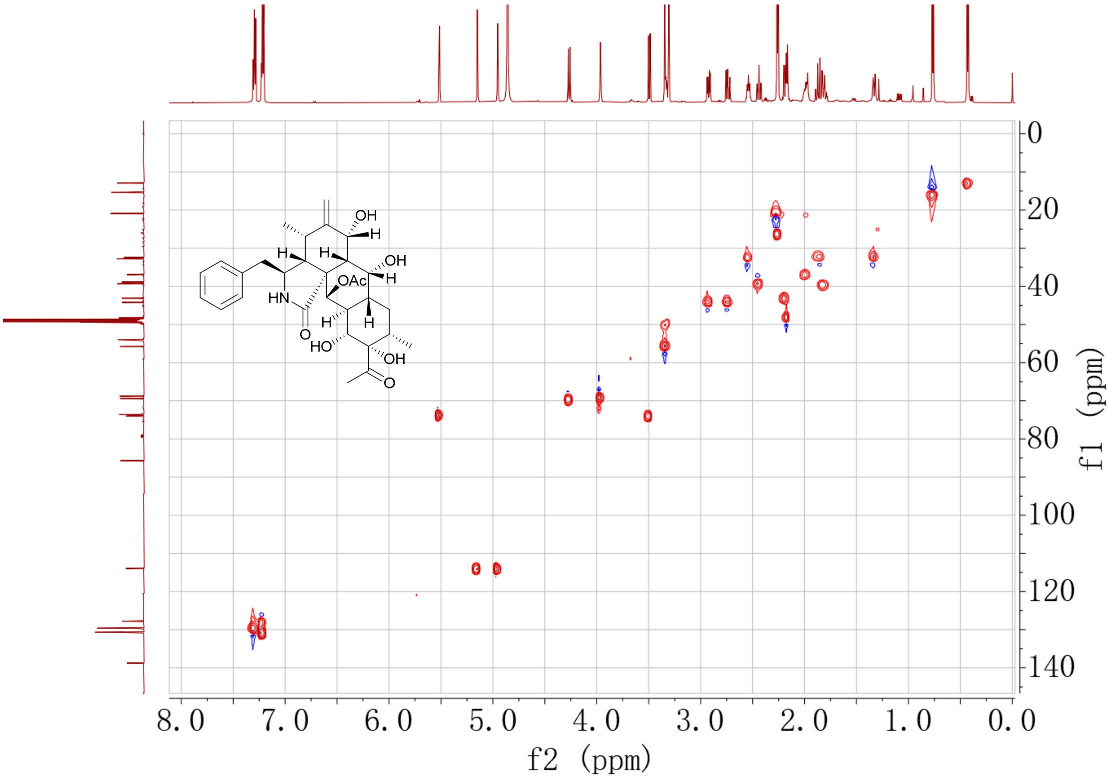


**Figure S134.** HSQC of **12**.

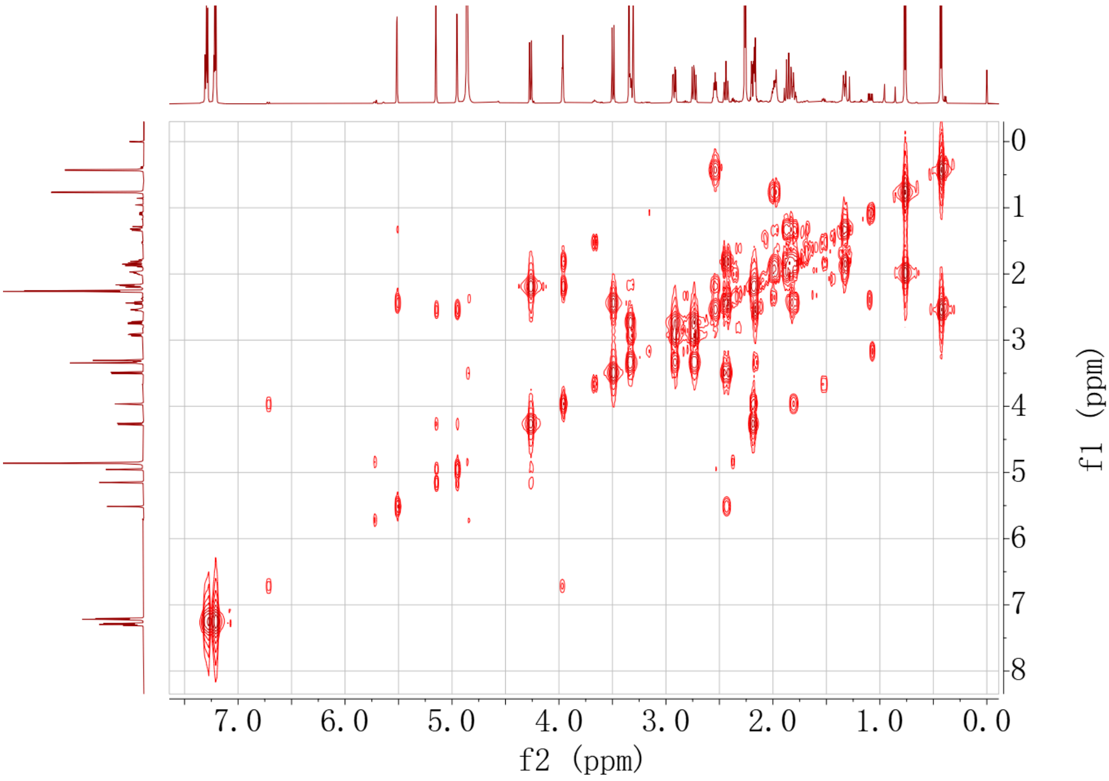


**Figure S135.** $^{1}H-^{1}H$ COSY of **12**.

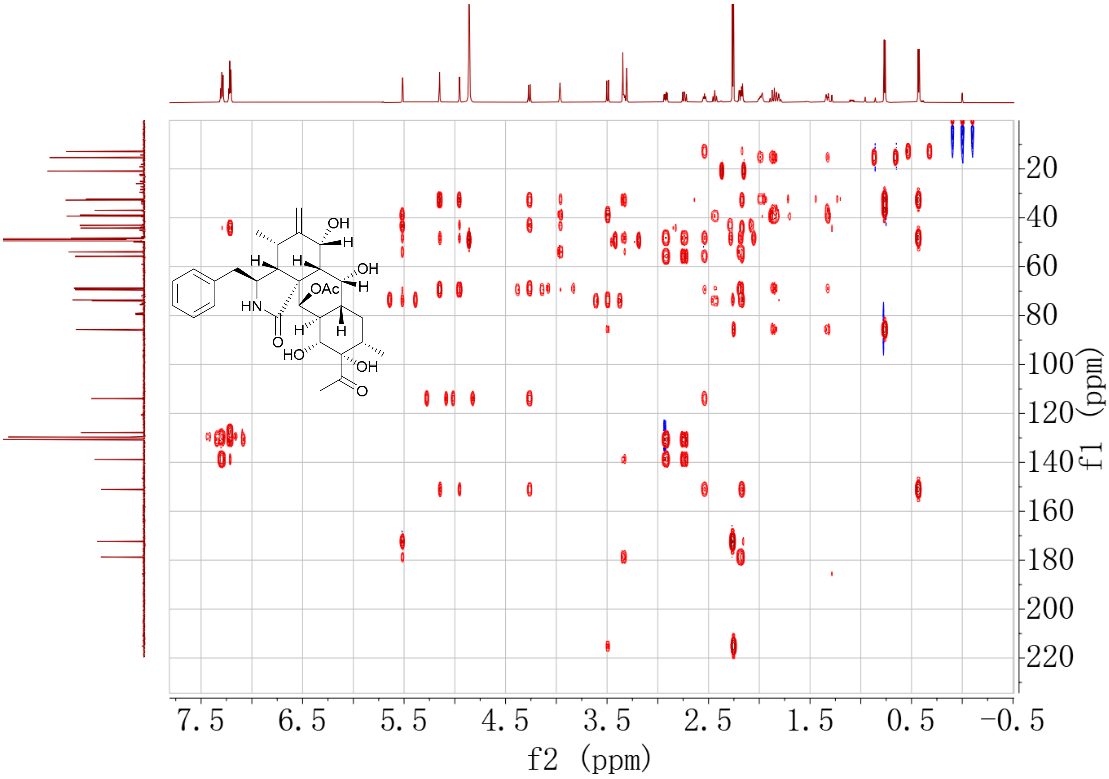


**Figure S136.** HMBC of **12**.

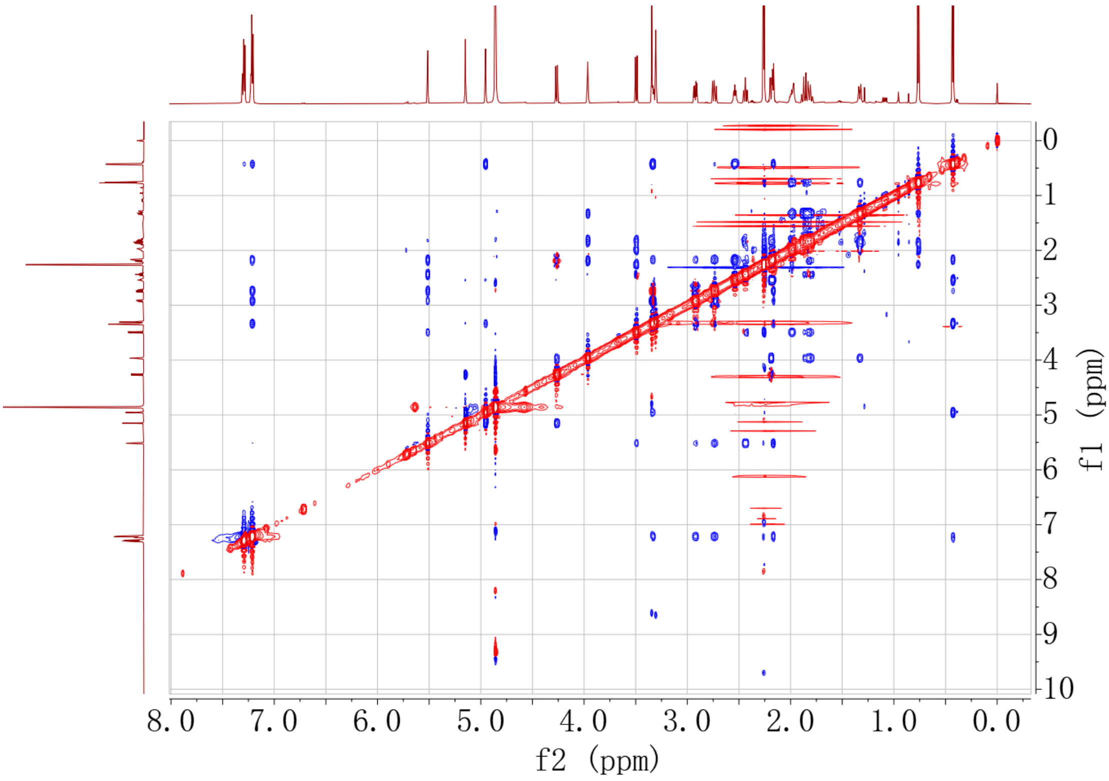


**Figure S137.** NOESY of **12**.

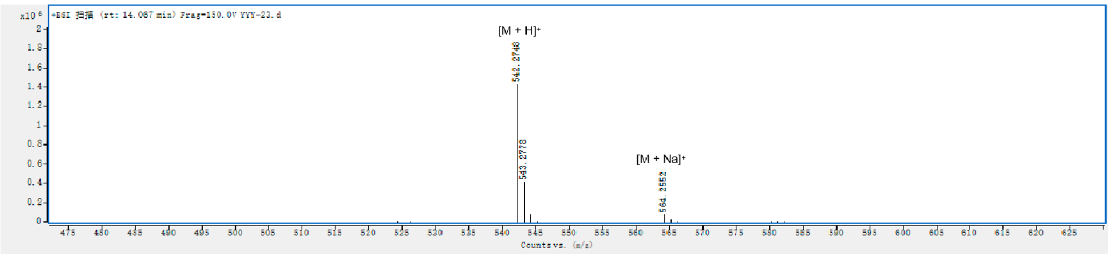


**Figure S138.** HRESIMS of **12**.

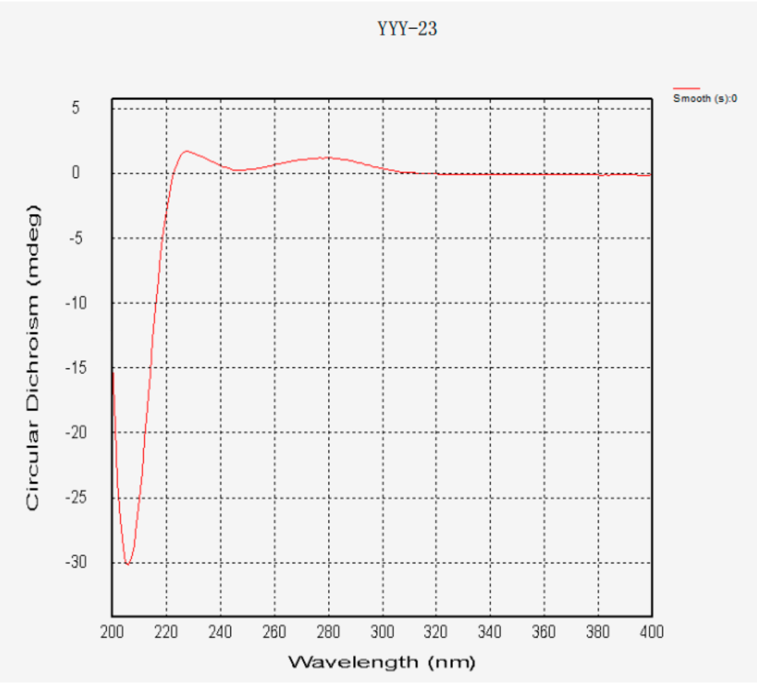


**Figure S139.** CD spectrum (acetonitrile) of **12.**

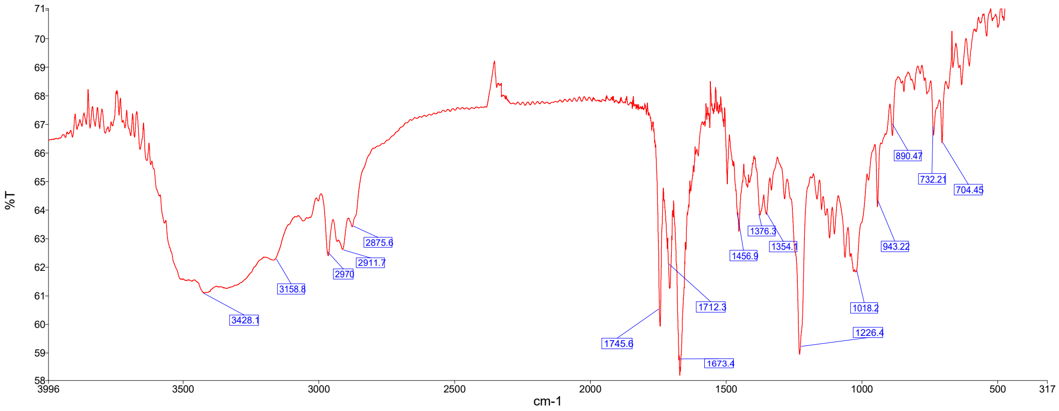


**Figure S140.** IR spectrum of **12.**

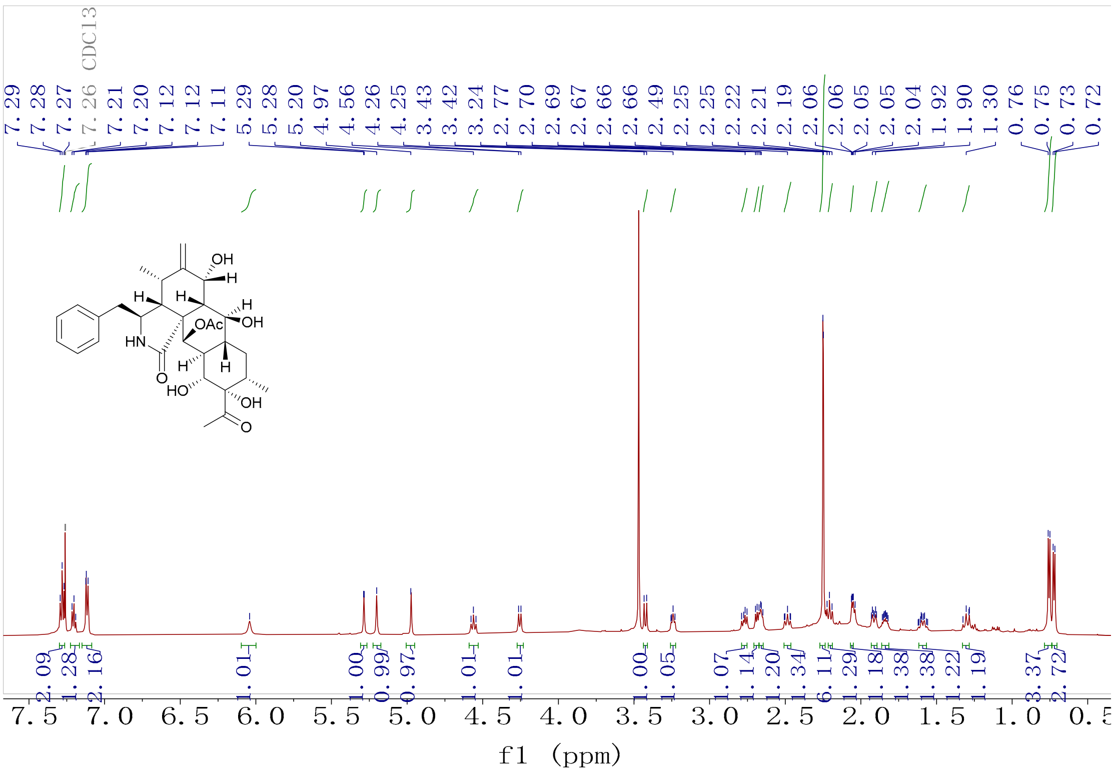

**Figure S141.** $^{1}$H NMR ($CDCl_3$, 600 MHz) of **13**.

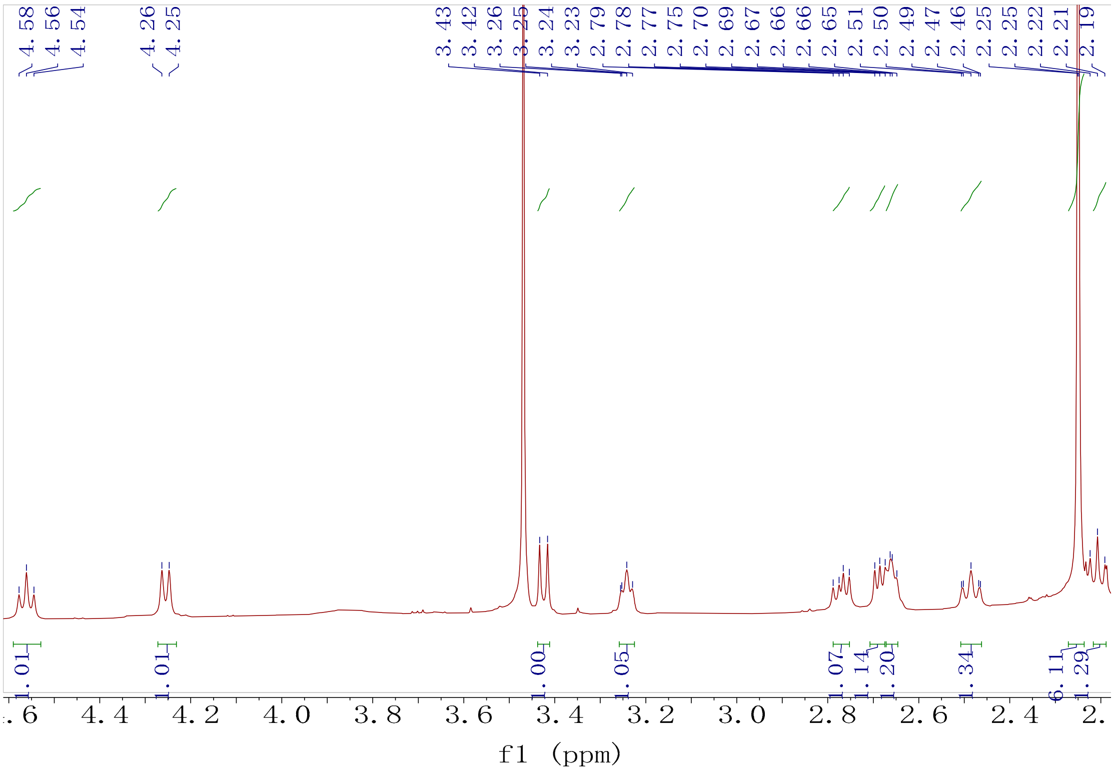

**Figure S142.** Partial $^{1}$H NMR spectrum ($CDCl_3$, 600 MHz) of **13**.

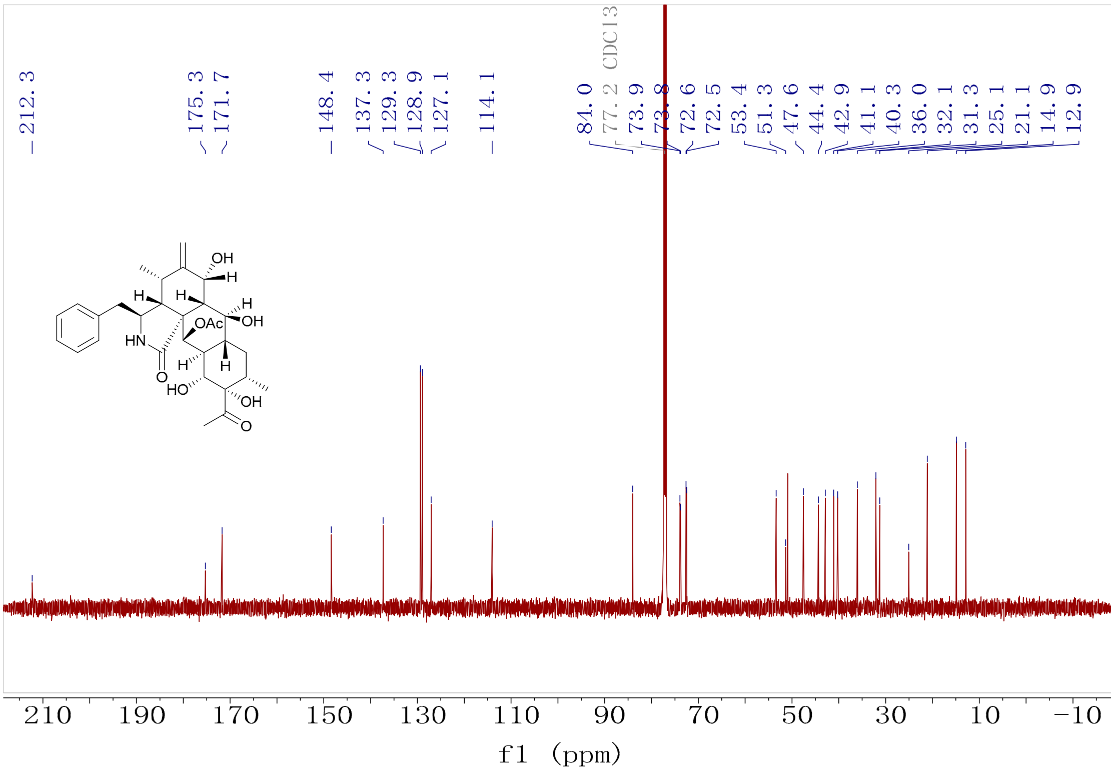


**Figure S143.** $^{13}C$ NMR ($CDCl_3$, 150 MHz) of **13**.

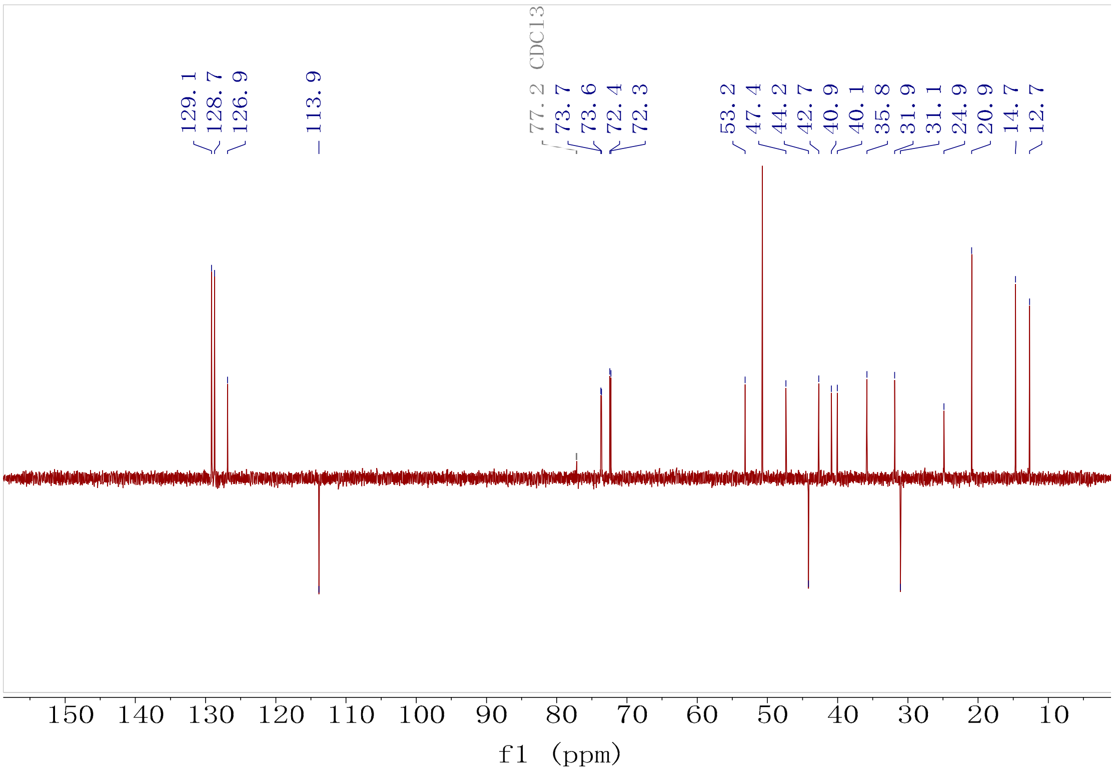


**Figure S144.** DEPT 135 spectra of **13**.

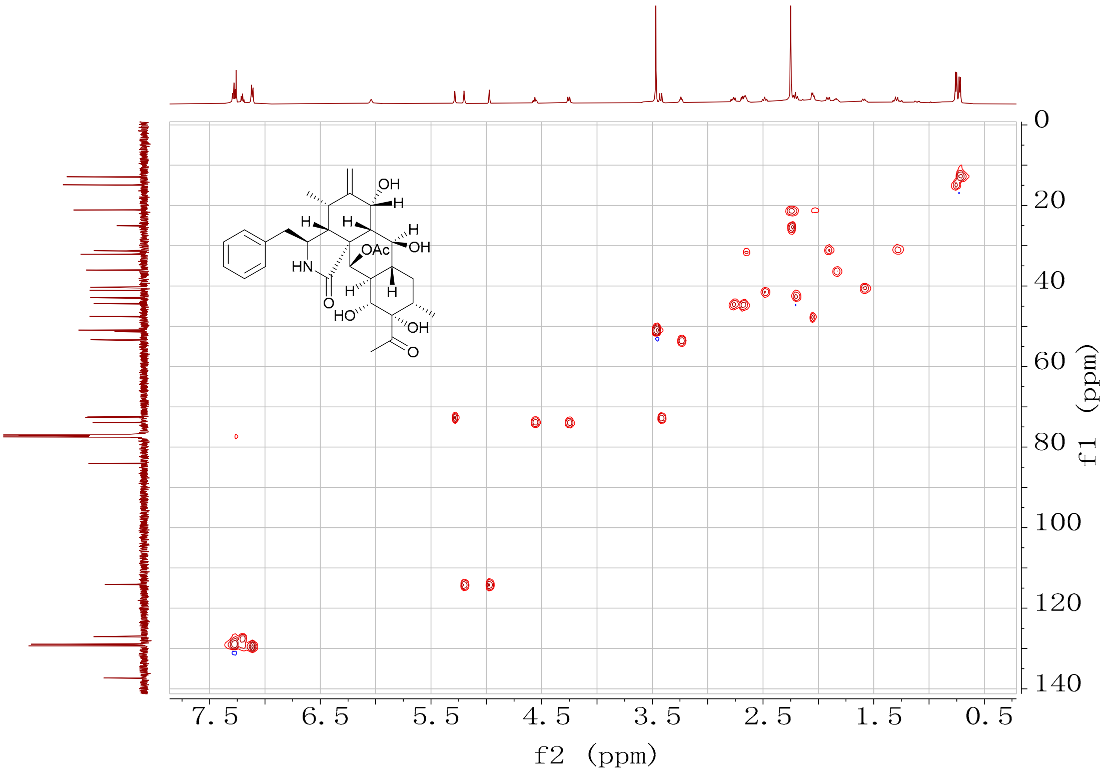


**Figure S145.** HSQC of **13**.

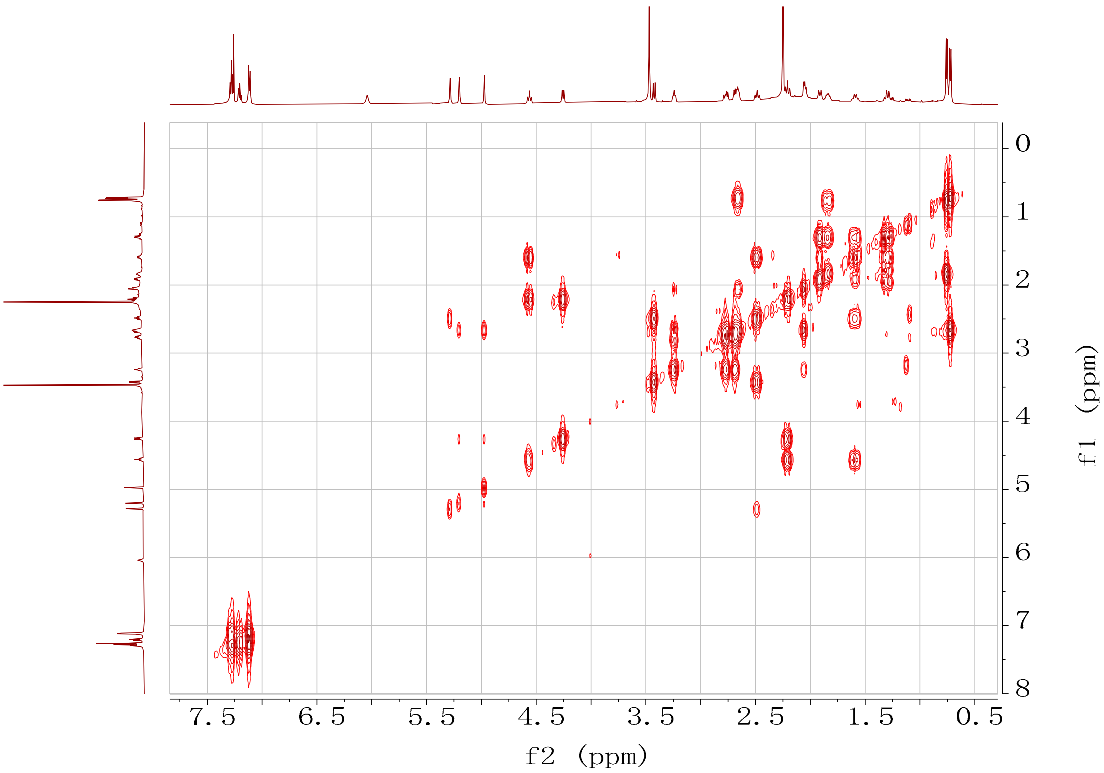


**Figure S146.** $^{1}H-^{1}H$ COSY of **13**.

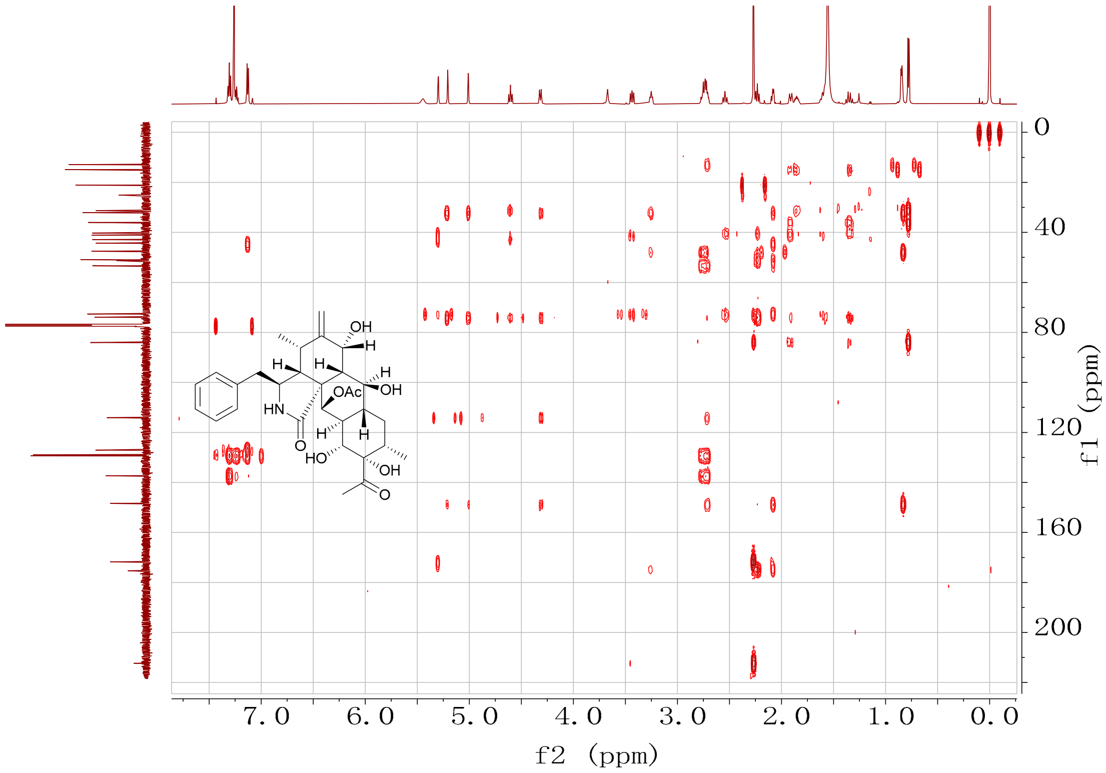


**Figure S147.** HMBC of **13**.

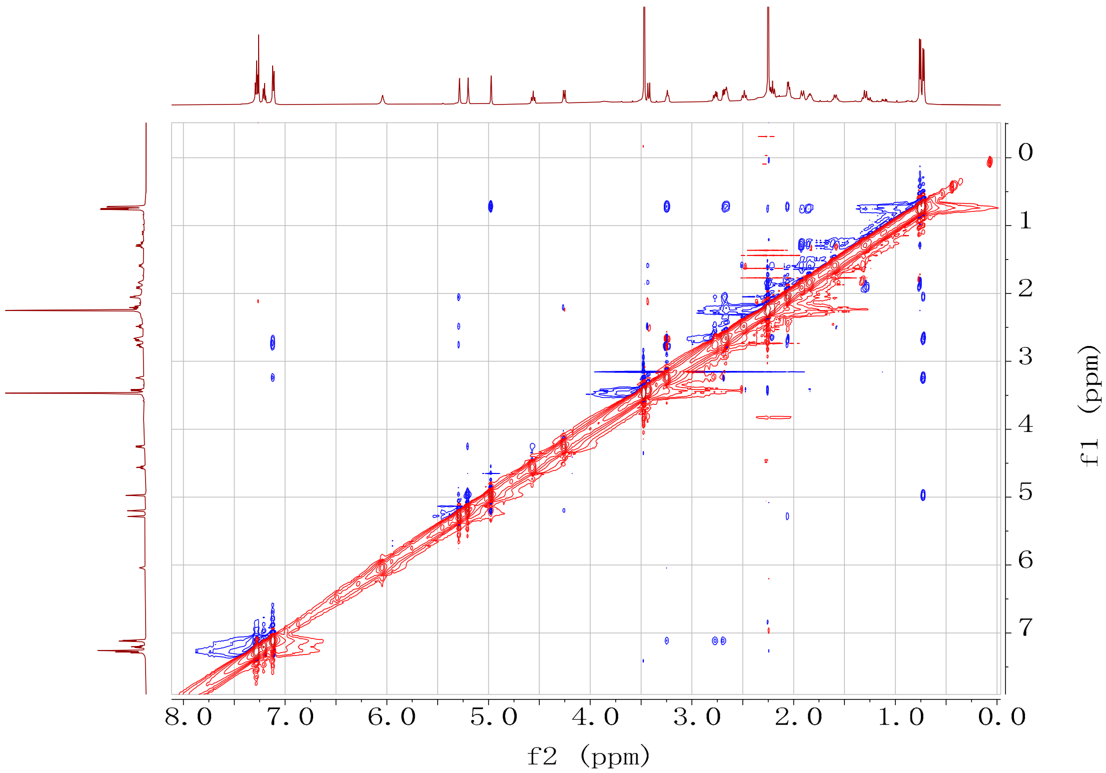


**Figure S148.** NOESY of **13**.

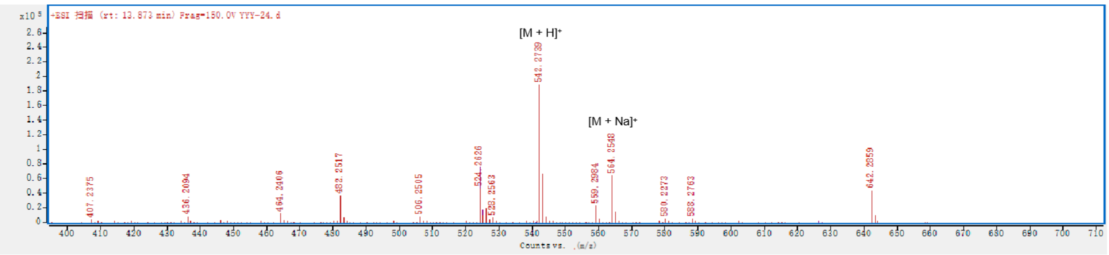


**Figure S149.** HRESIMS of **13**.

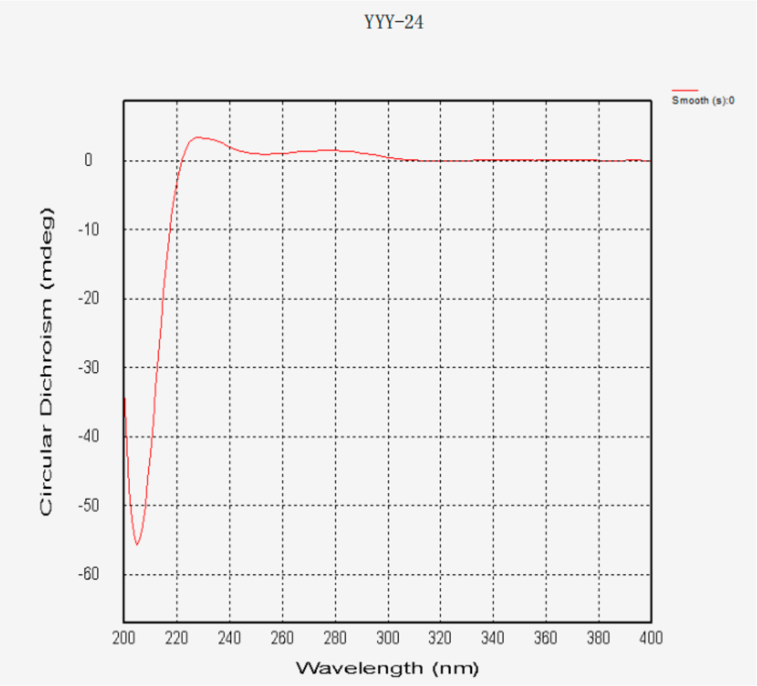


**Figure S150.** CD spectrum (methanol) of **13.**

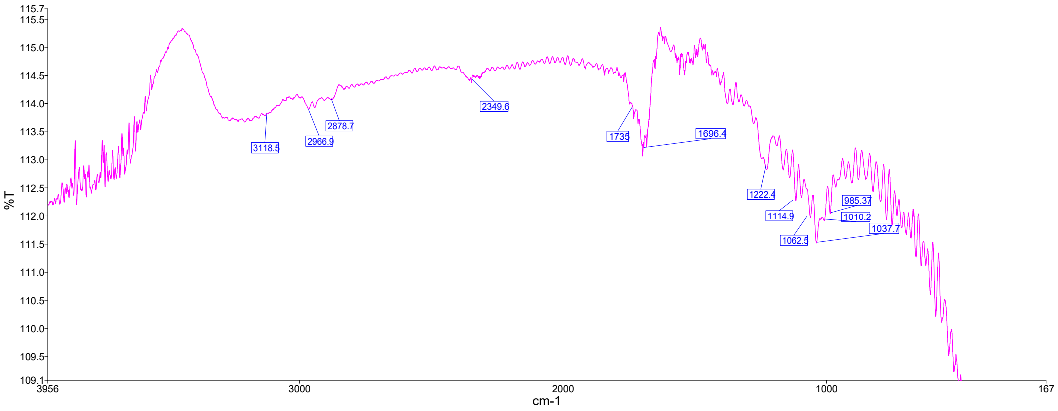


**Figure S151.** IR spectrum of **13.**

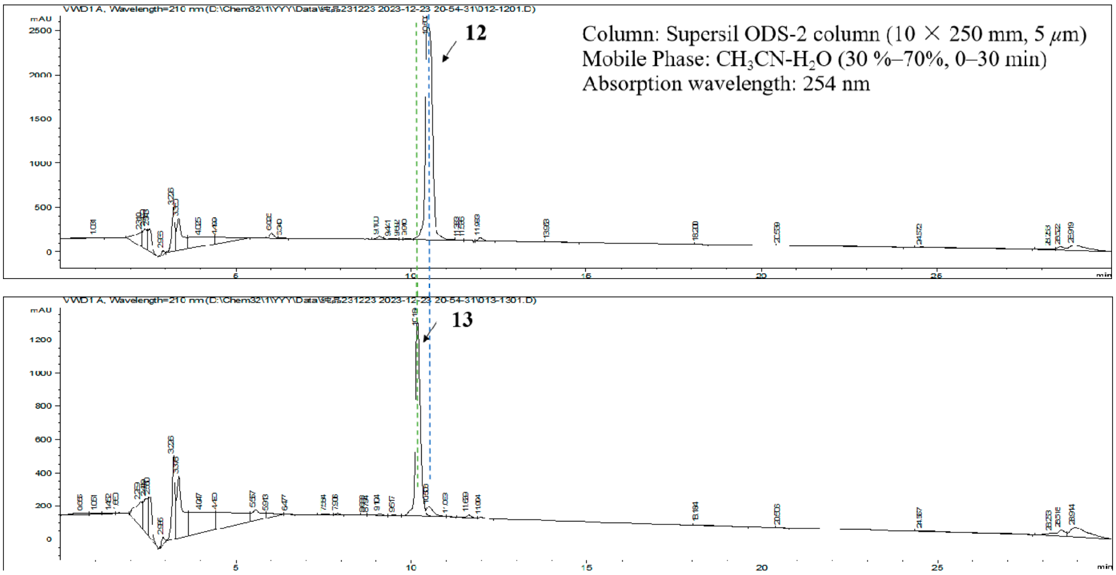


**Figure S152.** HPLC chromatography of **12** and **13**.

**Table S1.** The values of MAE, RMS, $P_{mean}$, and DP4 of the calculated chemical shifts of relative structures **1** and **2** fitting to each set of the experimental $^{13}$C NMR data of **1** and **2**, respectively. These red numbers represent the best matched structures.

| | **MAE (ppm)** | | **RMS (ppm)** | |
|---|---|---|---|---|
| Comp. / Calcd. | **1** | **2** | **1** | **2** |
| **1** | 1.38 | 2.20 | 1.77 | 3.50 |
| **2** | 2.08 | 1.19 | 3.72 | 1.52 |
| | ***Pmean*** | | **DP4** | |
| Comp. / Calcd. | **1** | **2** | **1** | **2** |
| **1** | 50.9% | 19.5% | 100.0% | 0.0% |
| **2** | 17.5% | 57.6% | 0.0% | 100.0% |

**Table S2.** The predicted values by GFN2NMR for **1** and **2** fitting to the experimental $^{13}$C NMR chemical shifts ($CDCl_3$) of **1**.

| No. | Exptl $\delta$ | Calcd. 1 | abs dev | Calcd. 2 | abs dev |
|---|---|---|---|---|---|
| 1 | 175.24 | 176.49 | 1.25 | 176.83 | 1.59 |
| 3 | 59.92 | 58.73 | 1.19 | 59.74 | 0.18 |
| 4 | 48.58 | 48.80 | 0.22 | 50.36 | 1.78 |
| 5 | 125.03 | 126.84 | 1.81 | 128.56 | 3.53 |
| 6 | 133.00 | 137.37 | 4.37 | 133.37 | 0.37 |
| 7 | 70.70 | 69.31 | 1.39 | 76.73 | 6.03 |
| 8 | 43.15 | 46.64 | 3.49 | 46.57 | 3.42 |
| 9 | 50.36 | 52.59 | 2.23 | 51.53 | 1.17 |
| 10 | 43.82 | 41.04 | 2.78 | 41.45 | 2.37 |
| 11 | 17.43 | 17.16 | 0.27 | 16.77 | 0.66 |
| 12 | 14.16 | 15.01 | 0.85 | 18.31 | 4.15 |
| 13 | 84.47 | 80.51 | 3.96 | 67.22 | 17.25 |
| 14 | 39.53 | 38.58 | 0.95 | 40.35 | 0.82 |
| 15 | 31.48 | 31.88 | 0.40 | 31.93 | 0.45 |
| 16 | 36.08 | 34.83 | 1.25 | 35.67 | 0.41 |
| 17 | 83.63 | 83.04 | 0.59 | 82.84 | 0.79 |
| 18 | 212.37 | 212.64 | 0.27 | 213.07 | 0.70 |
| 19 | 72.20 | 72.73 | 0.53 | 73.44 | 1.24 |
| 20 | 41.58 | 43.25 | 1.67 | 42.86 | 1.28 |
| 21 | 71.60 | 74.38 | 2.78 | 74.44 | 2.84 |
| 22 | 25.32 | 25.34 | 0.02 | 27.31 | 1.99 |
| 23 | 15.01 | 16.05 | 1.04 | 15.56 | 0.55 |
| 24 | 137.66 | 138.08 | 0.42 | 138.01 | 0.35 |
| 25 | 129.16 | 127.95 | 1.21 | 128.27 | 0.89 |
| 26 | 128.97 | 128.33 | 0.64 | 127.88 | 1.09 |
| 27 | 127.10 | 126.04 | 1.06 | 126.14 | 0.96 |

| | | | | | |
|---|---|---|---|---|---|
| 28 | 128.97 | 127.72 | 1.25 | 128.44 | 0.53 |
| 29 | 129.16 | 128.97 | 0.19 | 128.89 | 0.27 |
| 21-OAc | 172.10 | 169.38 | 2.72 | 170.82 | 1.28 |
| | 21.19 | 20.04 | 1.15 | 19.24 | 1.95 |
| 13-OCH3 | 59.92 | 59.18 | 0.74 | 56.28 | 3.64 |

**Table S3.** The predicted values by GFN2NMR for **1** and **2** fitting to the experimental $^{13}C$ NMR chemical shifts ($CDCl_3$) of **2**.

| No. | Exptl δ | Calcd δ | abs dev | Calcd δ | abs dev |
|---|---|---|---|---|---|
| 1 | 177.5 | 177.28 | 0.19 | 176.58 | 0.89 |
| 3 | 60.9 | 59.78 | 1.12 | 58.86 | 2.04 |
| 4 | 49.5 | 50.37 | 0.87 | 48.92 | 0.58 |
| 5 | 128.8 | 128.84 | 0.04 | 126.94 | 1.86 |
| 6 | 131.8 | 133.67 | 1.87 | 137.47 | 5.67 |
| 7 | 78.8 | 76.83 | 1.97 | 69.43 | 9.37 |
| 8 | 43.7 | 46.57 | 2.87 | 46.77 | 3.07 |
| 9 | 49.0 | 51.55 | 2.52 | 52.72 | 3.69 |
| 10 | 44.2 | 41.43 | 2.74 | 41.17 | 3.00 |
| 11 | 17.4 | 16.66 | 0.74 | 17.30 | 0.10 |
| 12 | 18.4 | 18.21 | 0.19 | 15.14 | 3.26 |
| 13 | 67.8 | 67.29 | 0.51 | 80.62 | 12.82 |
| 14 | 41.6 | 40.32 | 1.28 | 38.70 | 2.90 |
| 15 | 31.9 | 31.87 | 0.03 | 32.01 | 0.11 |
| 16 | 36.3 | 35.63 | 0.62 | 34.96 | 1.29 |
| 17 | 83.9 | 82.96 | 0.94 | 83.15 | 0.75 |
| 18 | 213.3 | 213.64 | 0.34 | 212.73 | 0.57 |
| 19 | 72.5 | 73.53 | 1.03 | 72.85 | 0.35 |
| 20 | 39.8 | 42.85 | 3.05 | 43.37 | 3.57 |
| 21 | 71.9 | 74.53 | 2.63 | 74.50 | 2.60 |
| 22 | 25.4 | 27.24 | 1.84 | 25.47 | 0.07 |
| 23 | 14.9 | 15.45 | 0.55 | 16.18 | 1.28 |
| 24 | 137.5 | 138.32 | 0.82 | 138.18 | 0.68 |
| 25 | 129.2 | 128.55 | 0.65 | 128.06 | 1.14 |
| 26 | 129.0 | 128.16 | 0.84 | 128.44 | 0.56 |
| 27 | 127.2 | 126.41 | 0.79 | 126.15 | 1.05 |
| 28 | 129.0 | 128.72 | 0.28 | 127.82 | 1.18 |
| 29 | 129.2 | 129.17 | 0.03 | 129.07 | 0.13 |
| 21-OAc | 171.9 | 171.25 | 0.65 | 169.48 | 2.42 |
| | 21.2 | 19.14 | 2.06 | 20.18 | 1.02 |

| | | | | | |
|---|---|---|---|---|---|
| 13-$OCH_3$ | 59.1 | 56.31 | 2.79 | 59.30 | 0.20 |

**Table S4.** The values of MAE, RMS, *Pmean*, and DP4 of the calculated chemical shifts of relative structures **3** and **4** fitting to each set of the experimental $^{13}C$ NMR data of **3** and **4**, respectively. These red numbers represent the best matched structures.

| | MAE (ppm) | | RMS (ppm) | |
|---|---|---|---|---|
| Comp. / Calcd. | **3** | **4** | **3** | **4** |
| **3** | 1.08 | 1.42 | 1.39 | 1.88 |
| **4** | 1.18 | 0.98 | 1.77 | 1.27 |
| | ***Pmean*** | | **DP4** | |
| Comp. / Calcd. | **3** | **4** | **3** | **4** |
| **3** | 61.1% | 48.9% | 96.6% | 0.0% |
| **4** | 54.4% | 64.7% | 3.4% | 100.0% |

**Table S5.** The predicted values by GFN2NMR for **3** and **4** fitting to the experimental $^{13}C$ NMR chemical shifts ($CDCl_3$) of **3**.

| No. | Exptl. | Calcd. 3 | Dev. | Calcd. 4 | Dev. |
|---|---|---|---|---|---|
| 1 | 176.8 | 177.18 | 0.38 | 175.93 | 0.87 |
| 3 | 60.2 | 59.95 | 0.25 | 59.98 | 0.22 |
| 4 | 49.4 | 50.00 | 0.60 | 49.11 | 0.29 |
| 5 | 125.3 | 125.73 | 0.43 | 126.76 | 1.46 |
| 6 | 134.0 | 136.16 | 2.16 | 133.90 | 0.10 |
| 7 | 76.5 | 78.80 | 2.30 | 81.05 | 4.55 |
| 8 | 43.2 | 42.99 | 0.21 | 43.01 | 0.19 |
| 9 | 51.6 | 52.13 | 0.53 | 52.33 | 0.73 |
| 10 | 43.5 | 39.77 | 3.73 | 40.11 | 3.39 |
| 11 | 17.4 | 17.25 | 0.15 | 16.54 | 0.86 |
| 12 | 14.7 | 15.90 | 1.20 | 14.70 | 0.00 |
| 13 | 66.7 | 67.27 | 0.57 | 69.51 | 2.81 |
| 14 | 37.5 | 38.17 | 0.67 | 38.86 | 1.36 |
| 15 | 31.2 | 29.28 | 1.92 | 31.58 | 0.38 |
| 16 | 36.0 | 35.63 | 0.37 | 34.89 | 1.11 |
| 17 | 83.8 | 82.72 | 1.08 | 83.06 | 0.74 |
| 18 | 212.4 | 212.02 | 0.38 | 211.79 | 0.61 |
| 19 | 72.6 | 72.71 | 0.11 | 72.17 | 0.43 |
| 20 | 38.1 | 40.24 | 2.14 | 41.58 | 3.48 |
| 21 | 72.0 | 73.52 | 1.52 | 72.01 | 0.01 |
| 22 | 25.0 | 25.46 | 0.46 | 25.06 | 0.06 |
| 23 | 14.3 | 15.79 | 1.49 | 15.02 | 0.72 |
| 24 | 137.2 | 138.31 | 1.11 | 138.73 | 1.53 |
| 25 | 129.0 | 128.34 | 0.66 | 128.79 | 0.21 |

| | | | | | |
|---|---|---|---|---|---|
| 26 | 128.9 | 128.24 | 0.66 | 128.88 | 0.02 |
| 27 | 127.1 | 126.45 | 0.65 | 126.43 | 0.67 |
| 21-OAc | 171.6 | 169.97 | 1.63 | 170.42 | 1.18 |
| | 21.0 | 19.94 | 1.06 | 19.55 | 1.45 |
| $OCH_3$ | 60.9 | 57.98 | 2.92 | 56.13 | 4.77 |

**Table S6.** The predicted values by GFN2NMR for **3** and **4** fitting to the experimental $^{13}C$ NMR chemical shifts ($CDCl_3$) of **4**.

| No. | Exptl. | Calcd. 4 | Dev. | Calcd. 3 | Dev. |
|---|---|---|---|---|---|
| 1 | 174.9 | 176.02 | 1.12 | 177.22 | 2.32 |
| 3 | 60.0 | 60.40 | 0.40 | 60.38 | 0.38 |
| 4 | 49.0 | 49.56 | 0.56 | 50.46 | 1.46 |
| 5 | 127.0 | 127.00 | 0.00 | 125.94 | 1.06 |
| 6 | 133.0 | 134.12 | 1.12 | 136.34 | 3.34 |
| 7 | 83.2 | 81.41 | 1.79 | 79.16 | 4.04 |
| 8 | 43.2 | 43.47 | 0.27 | 43.48 | 0.28 |
| 9 | 50.8 | 52.77 | 1.97 | 52.58 | 1.78 |
| 10 | 43.9 | 40.58 | 3.32 | 40.27 | 3.63 |
| 11 | 17.4 | 17.08 | 0.32 | 17.81 | 0.41 |
| 12 | 13.4 | 15.24 | 1.84 | 16.47 | 3.07 |
| 13 | 72.0 | 69.90 | 2.10 | 67.67 | 4.33 |
| 14 | 39.1 | 39.34 | 0.24 | 38.67 | 0.43 |
| 15 | 31.6 | 32.08 | 0.48 | 29.81 | 1.79 |
| 16 | 36.1 | 35.38 | 0.72 | 36.14 | 0.04 |
| 17 | 83.7 | 83.42 | 0.28 | 83.07 | 0.63 |
| 18 | 212.1 | 211.80 | 0.30 | 211.95 | 0.15 |
| 19 | 72.4 | 72.56 | 0.16 | 73.10 | 0.70 |
| 20 | 40.5 | 42.05 | 1.55 | 40.73 | 0.23 |
| 21 | 71.3 | 72.39 | 1.09 | 73.90 | 2.60 |
| 22 | 24.9 | 25.57 | 0.67 | 26.00 | 1.10 |
| 23 | 14.6 | 15.57 | 0.97 | 16.36 | 1.76 |
| 24 | 137.4 | 138.93 | 1.53 | 138.48 | 1.08 |
| 25/29 | 129.1 | 129.02 | 0.08 | 128.55 | 0.55 |
| 26/28 | 128.9 | 129.11 | 0.21 | 128.45 | 0.45 |
| 27 | 127.0 | 126.67 | 0.33 | 126.66 | 0.34 |
| 21-OAc | 171.9 | 170.54 | 1.36 | 170.04 | 1.86 |
| | 21.1 | 20.08 | 1.02 | 20.50 | 0.60 |
| OCH3 | 59.1 | 56.56 | 2.54 | 58.41 | 0.69 |

**Table S7.** The values of MAE, RMS, *Pmean*, and DP4 of the calculated chemical shifts of relative structures **5** and **14** fitting to each set of the experimental $^{13}C$ NMR data of **5** and **14**, respectively. These red numbers represent the best matched structures.

| | MAE (ppm) | | RMS (ppm) | |
|---|---|---|---|---|
| Comp. / Calcd. | **5** | **14** | **5** | **14** |
| **5** | 1.43 | 1.75 | 1.73 | 2.12 |
| **14** | 1.61 | 1.32 | 2.08 | 1.75 |
| | ***Pmean*** | | **DP4** | |
| Comp. / Calcd. | **5** | **14** | **5** | **14** |
| **5** | 50.7% | 41.1% | 99.0% | 0.1% |
| **14** | 43.4% | 52.2% | 1.0% | 99.9% |

**Table S8.** The predicted values by GFN2NMR for **5** and **14** fitting to the experimental $^{13}C$ NMR chemical shifts ($CDCl_3$) of **5**.

| No. | Exptl. | Calcd. 5 | Dev. | Calcd. 14 | Dev. |
|---|---|---|---|---|---|
| 1 | 177.1 | 179.31 | 2.19 | 177.51 | 0.39 |
| 3 | 60.6 | 57.87 | 2.68 | 57.04 | 3.51 |
| 4 | 49.5 | 49.82 | 0.34 | 46.94 | 2.54 |
| 5 | 125.2 | 127.21 | 2.04 | 126.99 | 1.82 |
| 6 | 134.3 | 135.83 | 1.51 | 136.87 | 2.55 |
| 7 | 66.3 | 69.78 | 3.53 | 69.80 | 3.55 |
| 8 | 43.9 | 44.35 | 0.42 | 47.46 | 3.53 |
| 9 | 51.8 | 53.28 | 1.50 | 50.90 | 0.88 |
| 10 | 43.5 | 40.32 | 3.14 | 38.35 | 5.11 |
| 11 | 17.4 | 17.53 | 0.17 | 16.41 | 0.95 |
| 12 | 14.4 | 13.77 | 0.64 | 13.13 | 1.28 |
| 13 | 66.6 | 67.61 | 1.04 | 70.75 | 4.18 |
| 14 | 37.2 | 38.24 | 1.08 | 38.81 | 1.65 |
| 15 | 30.9 | 30.01 | 0.90 | 31.02 | 0.11 |
| 16 | 35.9 | 37.36 | 1.46 | 35.57 | 0.33 |
| 17 | 83.9 | 80.99 | 2.86 | 82.71 | 1.14 |
| 18 | 214.3 | 211.85 | 2.44 | 213.94 | 0.35 |
| 19 | 72.6 | 72.25 | 0.34 | 72.80 | 0.21 |
| 20 | 38.1 | 38.85 | 0.72 | 41.14 | 3.01 |
| 21 | 71.8 | 71.97 | 0.20 | 72.63 | 0.86 |
| 22 | 24.9 | 25.87 | 0.97 | 25.62 | 0.72 |
| 23 | 14.8 | 15.75 | 0.95 | 15.37 | 0.57 |
| 24 | 137.2 | 140.49 | 3.30 | 138.26 | 1.07 |
| 25 | 129.0 | 128.52 | 0.50 | 128.31 | 0.71 |
| 26 | 128.9 | 127.62 | 1.30 | 128.47 | 0.45 |

| | | | | | |
|---|---|---|---|---|---|
| 27 | 127.1 | 125.63 | 1.48 | 125.89 | 1.22 |
| 28 | 128.9 | 128.71 | 0.21 | 127.73 | 1.19 |
| 29 | 129.0 | 127.56 | 1.46 | 128.14 | 0.88 |
| 21-OAc | 171.6 | 170.62 | 0.98 | 168.98 | 2.62 |
| | 21.0 | 18.53 | 2.49 | 19.96 | 1.06 |

**Table S9.** The predicted values by GFN2NMR for **5** and **14** fitting to the experimental $^{13}$C NMR chemical shifts ($CDCl_3$) of **14**.

| No. | Exptl. | Calcd. 14 | Dev. | Calcd. 5 | Dev. |
|---|---|---|---|---|---|
| 1 | 175.6 | 177.02 | 1.47 | 178.77 | 3.22 |
| 3 | 59.8 | 57.55 | 2.26 | 58.38 | 1.43 |
| 4 | 48.5 | 47.54 | 0.96 | 50.40 | 1.90 |
| 5 | 125.7 | 126.93 | 1.28 | 127.13 | 1.48 |
| 6 | 133.0 | 136.72 | 3.69 | 135.67 | 2.64 |
| 7 | 71.9 | 70.21 | 1.71 | 70.18 | 1.74 |
| 8 | 43.8 | 48.05 | 4.23 | 44.98 | 1.16 |
| 9 | 50.7 | 51.46 | 0.76 | 53.83 | 3.13 |
| 10 | 43.4 | 39.02 | 4.42 | 40.98 | 2.46 |
| 11 | 17.3 | 17.26 | 0.02 | 18.39 | 1.11 |
| 12 | 13.9 | 14.00 | 0.09 | 14.66 | 0.75 |
| 13 | **72.4** | **71.15** | **1.26** | **68.04** | **4.37** |
| 14 | 39.7 | 39.47 | 0.25 | 38.92 | 0.80 |
| 15 | 31.3 | 31.75 | 0.42 | 30.76 | 0.57 |
| 16 | 35.9 | 36.25 | 0.34 | 38.05 | 2.14 |
| 17 | 83.8 | 83.01 | 0.80 | 81.30 | 2.51 |
| 18 | 212.0 | 213.15 | 1.19 | 211.04 | 0.92 |
| 19 | 73.2 | 73.18 | 0.04 | 72.64 | 0.58 |
| 20 | 40.9 | 41.78 | 0.84 | 39.52 | 1.42 |
| 21 | 71.2 | 73.01 | 1.83 | 72.36 | 1.18 |
| 22 | 24.9 | 26.39 | 1.54 | 26.66 | 1.81 |
| 23 | 14.7 | 16.23 | 1.50 | 16.62 | 1.89 |
| 24 | 137.6 | 138.09 | 0.54 | 140.29 | 2.74 |
| 25 | 129.1 | 128.23 | 0.86 | 128.42 | 0.67 |
| 26 | 128.8 | 128.39 | 0.41 | 127.53 | 1.27 |
| 27 | 126.9 | 125.83 | 1.08 | 125.56 | 1.35 |
| 28 | 128.8 | 127.66 | 1.14 | 128.61 | 0.19 |
| 29 | 129.1 | 128.06 | 1.03 | 127.47 | 1.62 |
| 21-OAc | 171.8 | 168.56 | 3.20 | 170.16 | 1.60 |
| | 21.1 | 20.77 | 0.28 | 19.38 | 1.67 |

**Table S10.** The values of MAE, RMS, *Pmean*, and DP4 of the calculated chemical shifts of relative structures **12** and **13** fitting to each set of the experimental $^{13}C$ NMR data of **12** and **13**, respectively. These red numbers represent the best matched structures.

| | MAE (ppm) | | RMS (ppm) | |
|---|---|---|---|---|
| Comp. / Calcd. | **12** | **13** | **12** | **13** |
| **12** | 0.97 | 1.48 | 1.18 | 1.99 |
| **13** | 1.49 | 1.37 | 1.81 | 1.68 |
| | ***Pmean*** | | **DP4** | |
| Comp. / Calcd. | **12** | **13** | **12** | **13** |
| **12** | 65.8% | 46.4% | 100.0% | 2.6% |
| **13** | 48.8% | 52.3% | 0.0% | 97.4% |

**Table S11.** The predicted values by GFN2NMR for **12** and **13** fitting to the experimental $^{13}C$ NMR chemical shifts ($CDCl_3$) of **12**.

| No. | Exptl. | Calcd. 12 | Dev. | Calcd. 13 | Dev. |
|---|---|---|---|---|---|
| 1 | 178.7 | 179.04 | 0.34 | 178.21 | 0.49 |
| 3 | 55.78 | 54.01 | 1.77 | 52.84 | 2.94 |
| 4 | 48.27 | 50.81 | 2.54 | 47.05 | 1.22 |
| 5 | 32.79 | 32.48 | 0.31 | 34.45 | 1.66 |
| 6 | 151.05 | 149.74 | 1.31 | 152.45 | 1.40 |
| 7 | 69.39 | 70.24 | 0.85 | 72.69 | 3.30 |
| 8 | 43.09 | 43.88 | 0.79 | 46.34 | 3.25 |
| 9 | 53.98 | 52.90 | 1.08 | 53.85 | 0.13 |
| 10 | 44.2 | 41.72 | 2.48 | 41.77 | 2.43 |
| 11 | 12.92 | 14.99 | 2.07 | 14.45 | 1.53 |
| 12 | 113.97 | 112.26 | 1.71 | 111.07 | 2.90 |
| 13 | 68.82 | 68.30 | 0.52 | 72.26 | 3.44 |
| 14 | 39.31 | 38.27 | 1.04 | 40.52 | 1.21 |
| 15 | 32.45 | 30.65 | 1.80 | 31.52 | 0.93 |
| 16 | 36.94 | 36.36 | 0.58 | 34.75 | 2.19 |
| 17 | 85.69 | 85.11 | 0.58 | 82.55 | 3.14 |
| 18 | 215.15 | 215.65 | 0.50 | 215.92 | 0.77 |
| 19 | 73.98 | 74.82 | 0.84 | 72.95 | 1.03 |
| 20 | 38.85 | 39.46 | 0.61 | 41.34 | 2.49 |
| 21 | 73.58 | 74.01 | 0.43 | 74.43 | 0.85 |
| 22 | 26.01 | 27.48 | 1.47 | 24.87 | 1.14 |
| 23 | 15.37 | 16.96 | 1.59 | 16.10 | 0.73 |
| 24 | 138.76 | 140.04 | 1.28 | 139.32 | 0.56 |
| 25 | 130.68 | 130.82 | 0.14 | 130.99 | 0.31 |
| 26 | 129.61 | 130.02 | 0.41 | 129.65 | 0.04 |

| | | | | | |
|---|---|---|---|---|---|
| 27 | 127.78 | 127.66 | 0.12 | 127.57 | 0.21 |
| 28 | 129.61 | 130.17 | 0.56 | 130.40 | 0.79 |
| 29 | 130.68 | 130.16 | 0.52 | 129.51 | 1.17 |
| 21-OAc | 172.4 | 172.52 | 0.12 | 171.55 | 0.85 |
| | 20.89 | 20.16 | 0.73 | 19.35 | 1.54 |

**Table S12.** The predicted values by GFN2NMR for **12** and **13** fitting to the experimental $^{13}$C NMR chemical shifts ($CDCl_3$) of **13**.

| No. | Exptl. | Calcd. 13 | Dev. | Calcd. 12 | Dev. |
|---|---|---|---|---|---|
| 1 | 175.2 | 176.45 | 1.25 | 177.22 | 2.02 |
| 3 | 53.3 | 52.59 | 0.71 | 53.76 | 0.46 |
| 4 | 47.5 | 46.87 | 0.63 | 50.60 | 3.10 |
| 5 | 32.0 | 34.42 | 2.42 | 32.51 | 0.51 |
| 6 | 148.4 | 151.00 | 2.60 | 148.29 | 0.11 |
| 7 | 73.8 | 72.20 | 1.60 | 69.79 | 4.01 |
| 8 | 42.9 | 46.17 | 3.27 | 43.76 | 0.86 |
| 9 | 51.3 | 53.58 | 2.28 | 52.67 | 1.37 |
| 10 | 44.3 | 41.65 | 2.65 | 41.62 | 2.68 |
| 11 | 12.8 | 14.66 | 1.86 | 15.24 | 2.44 |
| 12 | 114.0 | 110.12 | 3.88 | 111.28 | 2.72 |
| 13 | 73.9 | 71.78 | 2.12 | 67.87 | 6.03 |
| 14 | 40.2 | 40.42 | 0.22 | 38.22 | 1.98 |
| 15 | 31.2 | 31.52 | 0.32 | 30.70 | 0.50 |
| 16 | 36.0 | 34.72 | 1.28 | 36.34 | 0.34 |
| 17 | 84.0 | 81.94 | 2.06 | 84.47 | 0.47 |
| 18 | 212.2 | 213.71 | 1.51 | 213.37 | 1.17 |
| 19 | 72.6 | 72.46 | 0.14 | 74.31 | 1.71 |
| 20 | 41.0 | 41.22 | 0.22 | 39.39 | 1.61 |
| 21 | 72.4 | 73.92 | 1.52 | 73.51 | 1.11 |
| 22 | 25.0 | 24.95 | 0.05 | 27.57 | 2.57 |
| 23 | 14.8 | 16.29 | 1.49 | 17.18 | 2.38 |
| 24 | 137.3 | 138.03 | 0.73 | 138.71 | 1.41 |
| 25 | 129.3 | 129.80 | 0.50 | 129.60 | 0.30 |
| 26 | 128.9 | 128.48 | 0.42 | 128.82 | 0.08 |
| 27 | 127.0 | 126.42 | 0.58 | 126.49 | 0.51 |
| 28 | 128.9 | 129.21 | 0.31 | 128.96 | 0.06 |
| 29 | 129.3 | 128.34 | 0.96 | 128.95 | 0.35 |
| 21-OAc | 171.7 | 169.87 | 1.83 | 170.78 | 0.92 |
| | 21.1 | 19.50 | 1.60 | 20.33 | 0.77 |

**Table S13.** Linear regression analysis of the experimental versus calculated $^{13}$C NMR chemical shifts of **1–13**.

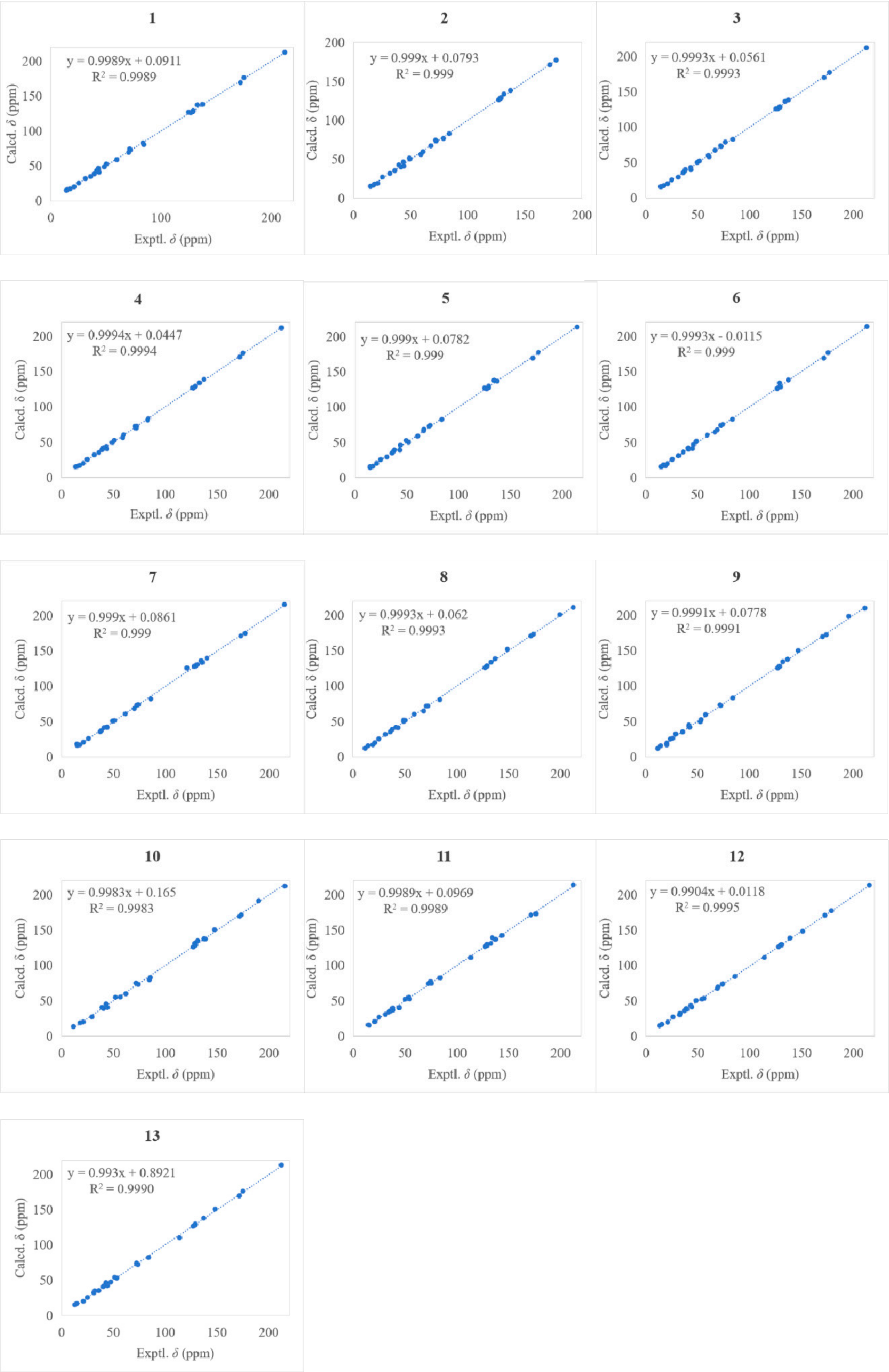

**Table S14.** Experimental $^{13}C$ NMR chemical shifts ($CDCl_3$) of **6** and the predicted values by GFN2NMR for **6**.

| No. | Exptl. | Calcd. | Dev. |
|---|---|---|---|
| 1 | 176 | 176.75 | 0.75 |
| 3 | 59.3 | 60.23 | 0.93 |
| 4 | 49.1 | 51.73 | 2.63 |
| 5 | 130.2 | 134.12 | 3.92 |
| 6 | 129.2 | 128.33 | 0.87 |
| 7 | 74.3 | 75.4 | 1.1 |
| 8 | 46.2 | 46.85 | 0.65 |
| 9 | 48.8 | 50.99 | 2.19 |
| 10 | 45.3 | 41.27 | 4.03 |
| 11 | 19.3 | 16.81 | 2.49 |
| 12 | 17.4 | 18.35 | 0.95 |
| 13 | 69.1 | 67.56 | 1.54 |
| 14 | 41.3 | 40.35 | 0.95 |
| 15 | 31.7 | 31.24 | 0.46 |
| 16 | 36.2 | 36.38 | 0.18 |
| 17 | 83.8 | 82.72 | 1.08 |
| 18 | 213.4 | 213.77 | 0.37 |
| 19 | 72.8 | 73.81 | 1.01 |
| 20 | 41.1 | 42.08 | 0.98 |
| 21 | 72.6 | 74.07 | 1.47 |
| 22 | 25.7 | 25.94 | 0.24 |
| 23 | 14.8 | 15.64 | 0.84 |
| 24 | 137.6 | 138.24 | 0.64 |
| 25 | 129.2 | 128.28 | 0.92 |
| 26 | 128.8 | 128.36 | 0.44 |
| 27 | 126.9 | 126.1 | 0.8 |
| 28 | 128.8 | 128.4 | 0.4 |
| 29 | 129.2 | 128.28 | 0.92 |
| 21-OAc | 171.9 | 169.05 | 2.85 |
| | 21 | 19.93 | 1.07 |
| 7-O*CH*$_2$CH$_3$ | 66.7 | 64.59 | 2.57 |
| 7-OCH$_2$*CH*$_3$ | 15.6 | 15.61 | 0.31 |
| | | MAE | 1.34 |
| | | RMS | 1.72 |
| | | $P_{mean}$ | 52.19% |

**Table S15.** Experimental $^{13}$C NMR chemical shifts ($CDCl_3$) of **7** and the predicted values by GFN2NMR for **7**.

| No. | Exptl. | Calcd. | Dev. |
|---|---|---|---|
| 1 | 176.8 | 174.94 | 1.86 |
| 3 | 61.4 | 61.06 | 0.34 |
| 4 | 49 | 50.82 | 1.82 |
| 5 | 128.4 | 128.18 | 0.22 |
| 6 | 135.3 | 133.64 | 1.66 |
| 7 | 70 | 68.49 | 1.51 |
| 8 | 41 | 41.49 | 0.49 |
| 9 | 50.9 | 51.4 | 0.5 |
| 10 | 43.8 | 41.7 | 2.1 |
| 11 | 17.5 | 17.04 | 0.46 |
| 12 | 14.8 | 18.41 | 3.61 |
| 13 | 120.6 | 125.93 | 5.33 |
| 14 | 134.5 | 136.18 | 1.68 |
| 15 | 38.2 | 37.13 | 1.07 |
| 16 | 37.2 | 35.74 | 1.46 |
| 17 | 85.9 | 82.18 | 3.72 |
| 18 | 214.6 | 215.71 | 1.11 |
| 19 | 73.9 | 73.96 | 0.06 |
| 20 | 43.6 | 42.14 | 1.46 |
| 21 | 72.3 | 73.12 | 0.82 |
| 22 | 25.9 | 26.19 | 0.29 |
| 23 | 15.1 | 16.21 | 1.11 |
| 24 | 139.9 | 139.8 | 0.1 |
| 25/29 | 130.7 | 130.65 | 0.05 |
| 26/28 | 129.7 | 129.84 | 0.14 |
| 27 | 127.7 | 128.14 | 0.44 |
| 21-OAc | 172.6 | 171.23 | 1.37 |
| | 21 | 20.98 | 0.02 |
| | | MAE | 1.24 |
| | | RMS | 1.75 |
| | | $P_{mean}$ | 53.46% |

**Table S16.** Experimental $^{13}$C NMR chemical shifts ($CDCl_3$) of **8** and the predicted values by GFN2NMR for **8**.

| No. | Exptl. | Calcd. | Dev. |
|---|---|---|---|
| 1 | 173.84 | 173.17 | 0.67 |
| 3 | 59.01 | 60.4 | 1.39 |

| | | | |
|---|---|---|---|
| 4 | 48.87 | 50.07 | 1.2 |
| 5 | 148.75 | 151.96 | 3.21 |
| 6 | 133.05 | 133.82 | 0.77 |
| 7 | 199.28 | 200.86 | 1.58 |
| 8 | 48.92 | 52.04 | 3.12 |
| 9 | 50.37 | 51.65 | 1.28 |
| 10 | 43.46 | 41.18 | 2.28 |
| 11 | 19.19 | 17.06 | 2.13 |
| 12 | 11.77 | 12.15 | 0.38 |
| 13 | 68.17 | 64.86 | 3.31 |
| 14 | 37.85 | 38.84 | 0.99 |
| 15 | 31.25 | 31.94 | 0.69 |
| 16 | 36 | 35.44 | 0.56 |
| 17 | 83.69 | 80.85 | 2.84 |
| 18 | 212.1 | 211.01 | 1.09 |
| 19 | 72.25 | 72.01 | 0.24 |
| 20 | 41.43 | 41.73 | 0.3 |
| 21 | 70.77 | 71.91 | 1.14 |
| 22 | 24.93 | 25.58 | 0.65 |
| 23 | 14.62 | 15.82 | 1.2 |
| 24 | 136.98 | 138.56 | 1.58 |
| 25 | 129.13 | 128.31 | 0.82 |
| 26 | 129.02 | 127.81 | 1.21 |
| 27 | 127.27 | 126.09 | 1.18 |
| 28 | 129.02 | 128.14 | 0.88 |
| 29 | 129.13 | 128.48 | 0.65 |
| 21-OAc | 171.41 | 170.76 | 0.65 |
| | 21.1 | 20.15 | 0.95 |
| | | MAE | 1.3 |
| | | RMS | 1.55 |
| | | $P_{mean}$ | 55.07% |

**Table S17.** Experimental $^{13}C$ NMR chemical shifts ($CDCl_3$) of **9** and the predicted values by GFN2NMR for **9**.

| No. | Exptl. | Calcd. | Dev. |
|---|---|---|---|
| 1 | 174.2 | 172.58 | 1.62 |
| 3 | 57.9 | 59.73 | 1.83 |
| 4 | 52.9 | 49.65 | 3.25 |
| 5 | 147.2 | 150.23 | 3.03 |

| | | | |
|---|---|---|---|
| 6 | 132.3 | 134.46 | 2.16 |
| 7 | 195.8 | 198.59 | 2.79 |
| 8 | 41.9 | 45.01 | 3.11 |
| 9 | 53.6 | 52.84 | 0.76 |
| 10 | 42.7 | 41.81 | 0.89 |
| 11 | 20.6 | 16.92 | 3.68 |
| 12 | 11.9 | 11.92 | 0.02 |
| 13 | 26.4 | 26.31 | 0.09 |
| 14 | 29.3 | 32.42 | 3.12 |
| 15 | 35.8 | 35.39 | 0.41 |
| 16 | 35.9 | 35.28 | 0.62 |
| 17 | 84.2 | 83.35 | 0.85 |
| 18 | 211.3 | 210.1 | 1.2 |
| 19 | 72.6 | 72.57 | 0.03 |
| 20 | 41.8 | 43.26 | 1.46 |
| 21 | 71.9 | 73.41 | 1.51 |
| 22 | 24 | 25.41 | 1.41 |
| 23 | 14.7 | 15.72 | 1.02 |
| 24 | 136.9 | 137.82 | 0.92 |
| 25 | 129.2 | 127.87 | 1.33 |
| 26 | 128.8 | 127.67 | 1.13 |
| 27 | 127.4 | 125.6 | 1.8 |
| 28 | 128.8 | 127.75 | 1.05 |
| 29 | 129.2 | 127.47 | 1.73 |
| 21-OAc | 170.6 | 169.76 | 0.84 |
| | 20.6 | 19.49 | 1.11 |
| | | MAE | 1.49 |
| | | RMS | 1.79 |
| | | Pmean | 48.92% |

**Table S18.** Experimental $^{13}C$ NMR chemical shifts ($CDCl_3$) of **10** and the predicted values by GFN2NMR for **10**.

| No. | Exptl. | Calcd. | Dev. |
|---|---|---|---|
| 1 | 173.17 | 171.55 | 1.62 |
| 3 | 62 | 59.91 | 2.09 |
| 4 | 42.9 | 45.5 | 2.6 |
| 5 | 131.19 | 134.97 | 3.78 |
| 6 | 147.52 | 150.65 | 3.13 |
| 7 | 128.96 | 131.84 | 2.88 |
| 8 | 137.36 | 137.84 | 0.48 |

| | | | |
|---|---|---|---|
| 9 | 52 | 54.99 | 2.99 |
| 10 | 44.21 | 40.58 | 3.63 |
| 11 | 190 | 191.46 | 1.46 |
| 12 | 17.83 | 19.03 | 1.2 |
| 13 | 128.09 | 130.81 | 2.72 |
| 14 | 137.36 | 137.69 | 0.33 |
| 15 | 85.55 | 82.86 | 2.69 |
| 16 | 39.08 | 40.78 | 1.7 |
| 17 | 84.67 | 79.72 | 4.95 |
| 18 | 215.2 | 212.23 | 2.97 |
| 19 | 73.92 | 73.15 | 0.77 |
| 20 | 40.44 | 39.22 | 1.22 |
| 21 | 71.77 | 75.17 | 3.4 |
| 22 | 29.25 | 27.32 | 1.93 |
| 23 | 11.47 | 13.66 | 2.19 |
| 24 | 138.61 | 137.44 | 1.17 |
| 25 | 129.2 | 128.96 | 0.24 |
| 26 | 128.96 | 128.52 | 0.44 |
| 27 | 126.94 | 126.27 | 0.67 |
| 28 | 128.96 | 128.71 | 0.25 |
| 29 | 129.2 | 129.25 | 0.05 |
| 30 | 171.59 | 169.44 | 2.15 |
| 21-OAc | 21.01 | 20.18 | 0.83 |
| | 56.6 | 55.33 | 1.27 |
| | | MAE | 1.86 |
| | | RMS | 2.23 |
| | | Pmean | 38.23% |

**Table S19.** Experimental $^{13}C$ NMR chemical shifts ($CDCl_3$) of **11** and the predicted values by GFN2NMR for **11**.

| No. | Exptl. | Calcd. | Dev. |
|---|---|---|---|
| 1 | 176.1 | 173.03 | 3.07 |
| 3 | 54.36 | 52.62 | 1.74 |
| 4 | 50.18 | 52.15 | 1.97 |
| 5 | 34.32 | 33.92 | 0.4 |
| 6 | 143.3 | 142.32 | 0.98 |
| 7 | 132.8 | 131.18 | 1.62 |
| 8 | 134.1 | 139.38 | 5.28 |
| 9 | 53.49 | 54.99 | 1.5 |
| 10 | 44.45 | 40.23 | 4.22 |

| | | | |
|---|---|---|---|
| 11 | 15.62 | 15.3 | 0.32 |
| 12 | 113.36 | 111.09 | 2.27 |
| 13 | 75.22 | 74.73 | 0.49 |
| 14 | 38.1 | 39.69 | 1.59 |
| 15 | 30.91 | 30.79 | 0.12 |
| 16 | 35.81 | 35.55 | 0.26 |
| 17 | 83.81 | 82.47 | 1.34 |
| 18 | 212.3 | 214.04 | 1.74 |
| 19 | 72.46 | 74.33 | 1.87 |
| 20 | 38.62 | 37.24 | 1.38 |
| 21 | 74.65 | 77.02 | 2.37 |
| 22 | 24.89 | 26.84 | 1.95 |
| 23 | 14.77 | 15.89 | 1.12 |
| 24 | 137.16 | 136.98 | 0.18 |
| 25 | 129.17 | 129.15 | 0.02 |
| 26 | 128.72 | 128.14 | 0.58 |
| 27 | 127.33 | 126.89 | 0.44 |
| 28 | 128.72 | 128.34 | 0.38 |
| 29 | 129.17 | 129.73 | 0.56 |
| 21-OAc | 171.28 | 171.37 | 0.09 |
| | 20.84 | 20.59 | 0.25 |
| | | MAE | 1.34 |
| | | RMS | 1.81 |
| | | $P_{mean}$ | 51.14% |

**Table S20.** Geometry data of conformers of compound **1**.

81

1-c11.out delta G = 0.0000 kcal/mol

conformer 1

O -0.190617 -0.740959 -1.309812
C 0.182680 -1.982162 -1.676592
C 1.113621 3.191248 -0.311322
O -0.187141 1.641814 2.548171
C 0.653411 1.036278 1.891555
N 1.740931 0.429991 2.396103
O 0.835426 4.173731 -1.326982
C 2.674448 -0.132887 1.435564
O -1.706605 3.452484 -0.993370
C -2.339633 4.514969 -0.275891
O -4.255657 -0.587160 1.825097
C 2.118608 0.402059 0.074978
C 2.990476 1.544031 -0.391679
O -2.275254 -2.362465 1.042278
C 2.564866 2.808020 -0.473898
O -5.742772 -2.558281 1.098074
C 0.170633 1.995654 -0.463366
C 2.804549 -1.663256 1.548144
C 0.646170 0.787002 0.365095
C 4.419641 1.150478 -0.695583
C 3.489364 3.961678 -0.762854
C -1.303589 2.332783 -0.168725
C -2.217879 1.138551 -0.450250
C -3.690177 1.449504 -0.190934
C -4.588960 0.239636 -0.452120
C -4.139388 -0.935980 0.449301
C -5.011850 -2.176138 0.201900
C -2.661674 -1.285475 0.191383
C -0.301710 -0.406898 0.101675
C -1.759973 -0.064353 0.387110
C 4.903175 -3.180569 -1.248224
C 3.804609 -2.794813 -0.483940
C 3.980114 -2.165934 0.749078
C -6.064469 0.583723 -0.259535
C -4.930903 -2.882930 -1.116568
C 6.380446 -2.329807 0.448426
C 5.281864 -1.945634 1.207728
C 6.194183 -2.945052 -0.787518
O 0.447796 -2.866794 -0.894737
C 0.250064 -2.096484 -3.170949
H 0.962578 3.655905 0.674725
H 1.930301 0.452878 3.387553
H -0.130364 4.143346 -1.448698
H 3.659180 0.292793 1.623970
H -2.597025 5.278011 -1.008422
H -1.661884 4.942028 0.469273
H -3.248753 4.174407 0.222215
H -4.979917 -1.136318 2.173632
H 2.169853 -0.395171 -0.665628
H -2.539960 -2.106822 1.938198
H 0.235159 1.692728 -1.512560
H 2.944920 -1.907770 2.604124
H 1.885601 -2.146896 1.218472
H 5.095010 1.352005 0.140776
H 4.804563 1.688802 -1.560940
H 4.488015 0.083237 -0.906871
H 3.408203 4.291128 -1.801046
H 4.528723 3.711266 -0.561846
H 3.221398 4.819904 -0.142133
H -1.396854 2.622122 0.877949
H -2.104901 0.885549 -1.509632
H -4.011692 2.263690 -0.840890
H -3.828242 1.778891 0.842499
H -4.433735 -0.071077 -1.489574
H -2.558789 -1.654338 -0.828721
H -0.002062 -1.275804 0.678694
H -1.821773 0.212351 1.441654
H 4.748086 -3.666201 -2.203869
H 2.802301 -2.986813 -0.843250
H -6.719936 -0.244360 -0.536684
H -6.332652 1.439878 -0.880196
H -6.266774 0.843300 0.780839
H -4.053136 -3.535242 -1.100771
H -4.809673 -2.192258 -1.949823
H -5.817174 -3.496749 -1.261014
H 7.381860 -2.150127 0.819672
H 5.435640 -1.464008 2.166829
H 7.048847 -3.242687 -1.381978
H 1.093109 -1.504455 -3.533197
H -0.655275 -1.695344 -3.625573
H 0.389472 -3.135547 -3.455587

81

1-c10.out delta G = 0.1851 kcal/mol

conformer 2
O -0.214391 -0.648718 -1.313734
C 0.183831 -1.861005 -1.745947
C 1.117317 3.207080 -0.001332
O -0.248975 1.536554 2.638597
C 0.613696 0.974455 1.972217
N 1.711434 0.377758 2.465622
O 0.806559 4.376934 -0.771826
C 2.662986 -0.137707 1.495456
O -1.723920 3.520666 -0.877904
C -1.877725 3.340073 -2.287754
O -4.238751 -0.645386 1.886013
C 2.104324 0.417880 0.144502
C 2.970502 1.571053 -0.309818
O -2.296915 -2.384918 0.965002
C 2.557455 2.843214 -0.292894
O -5.759066 -2.557726 1.075760
C 0.178411 2.041255 -0.335548
C 2.822281 -1.667275 1.566337
C 0.627895 0.787052 0.437869
C 4.372772 1.165534 -0.703196
C 3.471839 4.010222 -0.553614
C -1.312228 2.362484 -0.107576
C -2.235492 1.170377 -0.367351
C -3.689764 1.500241 -0.036223
C -4.618774 0.321431 -0.328119
C -4.156591 -0.910956 0.489301
C -5.046265 -2.126533 0.187299
C -2.686434 -1.259277 0.181808
C -0.317768 -0.389831 0.114274
C -1.771588 -0.056814 0.426209
C 6.390772 -2.241574 0.392078
C 5.298944 -1.895802 1.179188
C 3.993727 -2.127986 0.736858
C -6.081065 0.673592 -0.063796
C -5.003724 -2.751613 -1.173703
C 4.899786 -3.077547 -1.299745
C 3.808078 -2.730235 -0.507932
C 6.194156 -2.829456 -0.855442
O 0.476778 -2.777258 -1.011915
C 0.236838 -1.899185 -3.244724
H 1.007024 3.457549 1.064203
H 1.889697 0.366768 3.459415
H -0.164718 4.437735 -0.776335
H 3.637103 0.305015 1.700001
H -2.003902 4.336050 -2.708097
H -2.762030 2.744502 -2.521391
H -1.000040 2.874741 -2.740958
H -4.958985 -1.209949 2.217842
H 2.158441 -0.368400 -0.607216
H -2.543781 -2.176322 1.878109
H 0.323751 1.811487 -1.393601
H 2.980978 -1.938158 2.613212
H 1.905887 -2.156217 1.236076
H 4.377289 0.147199 -1.094385
H 5.063817 1.176947 0.144468
H 4.783444 1.820635 -1.469285
H 3.294523 4.794457 0.186250
H 3.281754 4.459372 -1.530681
H 4.520283 3.727351 -0.499641
H -1.442749 2.686029 0.923491
H -2.169662 0.909842 -1.427499
H -4.021225 2.362768 -0.615247
H -3.772056 1.774083 1.019183
H -4.506267 0.067456 -1.386360
H -2.604207 -1.572135 -0.858964
H -0.014916 -1.287940 0.643342
H -1.824785 0.178866 1.490118
H 7.395116 -2.053051 0.750837
H 5.461341 -1.436041 2.147556
H -6.756977 -0.133938 -0.352067
H -6.360653 1.559497 -0.635748
H -6.240835 0.888745 0.993816
H -4.135613 -3.415418 -1.217638
H -4.890837 -2.013009 -1.965942
H -5.901706 -3.343794 -1.335203
H 4.736824 -3.542417 -2.264385
H 2.802995 -2.929622 -0.855635
H 7.043627 -3.097014 -1.471292
H 0.420806 -2.915451 -3.581537
H 1.044328 -1.249478 -3.588168
H -0.693800 -1.521613 -3.667738
81
1-c9.out delta G = 0.5190 kcal/mol
conformer 3
O -0.352891 -0.258026 -1.981799
C -0.304136 -1.429425 -2.632293
C 0.959635 3.477377 -0.344881

O 0.590660 1.149846 2.239914
C 1.216940 0.801248 1.245813
N 2.406491 0.174604 1.254074
O 0.394804 4.631271 -0.995779
C 3.017354 -0.065542 -0.043808
O -1.931728 3.606767 -0.157102
C -2.343414 4.398822 0.959613
O -3.490630 -1.374953 2.012710
C 2.122102 0.795282 -0.989886
C 2.823287 2.100666 -1.281586
O -1.804388 -2.567683 0.232781
C 2.341506 3.293985 -0.922569
O -4.910001 -2.460930 -1.085372
C 0.055073 2.262019 -0.558347
C 3.105914 -1.555223 -0.420630
C 0.781193 0.945981 -0.232941
C 4.158728 1.946292 -1.976957
C 3.125661 4.569653 -1.083492
C -1.282000 2.363508 0.198559
C -2.209465 1.192624 -0.128333
C -3.525398 1.261617 0.646025
C -4.450370 0.081713 0.347368
C -3.735599 -1.258540 0.612930
C -4.624514 -2.413447 0.095395
C -2.406797 -1.326538 -0.152307
C -0.157014 -0.241934 -0.536823
C -1.502320 -0.135938 0.169648
C 5.941362 -3.099641 1.592863
C 5.249533 -2.405148 0.606129
C 3.853492 -2.356263 0.611868
C -5.763679 0.195250 1.122086
C -5.108130 -3.436025 1.080833
C 3.853663 -3.713823 2.619954
C 3.165039 -3.019285 1.629880
C 5.244508 -3.755399 2.604489
O -0.190259 -2.504802 -2.073875
C -0.385532 -1.239556 -4.114913
H 1.040270 3.687761 0.732217
H 2.858848 -0.067879 2.122990
H -0.565678 4.550615 -0.859584
H 4.033071 0.326802 -0.023584
H -3.061968 3.865425 1.584048
H -2.815513 5.291683 0.553360
H -1.484594 4.690066 1.571591
H -2.815099 -2.056791 2.130940
H 1.977246 0.261160 -1.929103
H -1.146145 -2.796530 -0.444605
H -0.169175 2.234686 -1.629235
H 2.109019 -1.972077 -0.558961
H 3.613099 -1.614069 -1.386180
H 4.290683 2.696136 -2.756500
H 4.237949 0.964992 -2.447274
H 5.001712 2.043137 -1.286927
H 2.980424 5.211464 -0.211335
H 2.786320 5.140512 -1.950763
H 4.192029 4.383001 -1.191529
H -1.077181 2.373784 1.269134
H -2.430755 1.238640 -1.200612
H -4.052545 2.181677 0.391373
H -3.314375 1.292050 1.718953
H -4.677194 0.085654 -0.722029
H -2.648374 -1.342411 -1.217296
H 0.326208 -1.171970 -0.257944
H -1.286423 -0.174894 1.239374
H 7.023615 -3.131920 1.570746
H 5.797578 -1.899111 -0.180480
H -6.459750 -0.604734 0.862628
H -6.251001 1.143995 0.891663
H -5.585865 0.156617 2.197333
H -4.252516 -3.980221 1.488957
H -5.784248 -4.131541 0.588588
H -5.600214 -2.950879 1.924768
H 3.303967 -4.224503 3.400891
H 2.081933 -2.991923 1.644758
H 5.781671 -4.297809 3.372310
H 0.562366 -0.829508 -4.469959
H -1.169316 -0.525344 -4.363998
H -0.569768 -2.193847 -4.599928
81
1-c2.out delta G = 0.5924 kcal/mol
conformer 4
O -0.002337 -0.742064 -1.267901
C 0.117981 -2.033970 -1.596138
C 1.093098 3.255705 -0.307321
O -0.214512 1.686139 2.558834
C 0.664762 1.120166 1.918993
N 1.784273 0.591290 2.441130
O 0.793739 4.219756 -1.333673

C 2.722966 0.003459 1.501452
O -1.731934 3.439655 -0.992273
C -2.374786 4.497001 -0.275589
O -4.216723 -0.812951 1.777433
C 2.169577 0.500734 0.126968
C 3.013842 1.658902 -0.350356
O -2.193167 -2.429201 0.913355
C 2.555073 2.909325 -0.457695
O -4.771421 -2.655764 -1.237321
C 0.182922 2.034177 -0.452767
C 2.843926 -1.523013 1.671033
C 0.684037 0.849506 0.393900
C 4.453542 1.297773 -0.642385
C 3.449101 4.082568 -0.762250
C -1.299715 2.333745 -0.163916
C -2.184514 1.118051 -0.444159
C -3.656274 1.387719 -0.133055
C -4.546542 0.173467 -0.398487
C -4.044406 -1.056882 0.383173
C -4.839515 -2.301573 -0.076361
C -2.563312 -1.322603 0.083639
C -0.218039 -0.375834 0.128879
C -1.696731 -0.092474 0.362673
C 6.243818 -2.328417 0.173653
C 5.257574 -1.857530 1.031961
C 3.906359 -2.103698 0.774470
C -6.014133 0.483191 -0.102279
C -5.677414 -3.024089 0.936763
C 4.552885 -3.321399 -1.219285
C 3.568366 -2.849335 -0.355393
C 5.893694 -3.057664 -0.960298
O -0.022081 -2.946908 -0.802418
C 0.480552 -2.206825 -3.038053
H 0.924140 3.725008 0.673775
H 1.955597 0.625648 3.435534
H -0.170463 4.164089 -1.458691
H 3.708420 0.431994 1.677636
H -1.694133 4.946338 0.453630
H -3.268268 4.142952 0.240934
H -2.659900 5.246344 -1.011958
H -3.658513 -1.444828 2.251056
H 2.250339 -0.303714 -0.602396
H -1.395183 -2.827573 0.527458
H 0.261612 1.722619 -1.498601
H 3.091879 -1.718916 2.717502
H 1.883989 -2.001581 1.477692
H 5.120123 1.525693 0.194456
H 4.829148 1.834979 -1.512377
H 4.549159 0.230134 -0.840722
H 3.159700 4.940871 -0.151282
H 3.357379 4.397427 -1.804036
H 4.494803 3.861725 -0.559951
H -1.402299 2.622925 0.881502
H -2.090015 0.886951 -1.511134
H -4.015926 2.214200 -0.746740
H -3.759123 1.688214 0.913745
H -4.454961 -0.093864 -1.454667
H -2.493987 -1.604423 -0.969143
H 0.101769 -1.211083 0.744652
H -1.798437 0.140821 1.424365
H 7.285826 -2.126175 0.388256
H 5.539713 -1.286961 1.909256
H -6.336209 1.349899 -0.681709
H -6.158892 0.707821 0.954986
H -6.667355 -0.350900 -0.366181
H -6.340132 -2.326962 1.450767
H -5.028383 -3.459620 1.700910
H -6.248234 -3.811317 0.449250
H 4.270484 -3.896736 -2.092233
H 2.528453 -3.074637 -0.553757
H 6.660927 -3.422718 -1.631355
H 0.307809 -3.235434 -3.341555
H 1.540039 -1.969569 -3.159713
H -0.088239 -1.519902 -3.662640
81
1-c1.out delta G = 0.7348 kcal/mol conformer 5
O -0.017794 -0.666661 -1.256772
C 0.093912 -1.947104 -1.632726
C 1.098175 3.282299 -0.007842
O -0.250866 1.601722 2.647633
C 0.643192 1.072331 1.997309
N 1.770333 0.546389 2.506569
O 0.760074 4.430054 -0.798426
C 2.719273 -0.000800 1.551468
O -1.747129 3.506593 -0.887440
C -1.904611 3.312488 -2.295188
O -4.190192 -0.832808 1.857929

C 2.162878 0.525535 0.189728
C 3.001209 1.697035 -0.270069
O -2.200326 -2.428908 0.891393
C 2.551274 2.956578 -0.280042
O -4.830075 -2.552136 -1.212204
C 0.195310 2.085006 -0.332182
C 2.856688 -1.530534 1.673093
C 0.675244 0.857829 0.464531
C 4.415775 1.323006 -0.648526
C 3.431953 4.146058 -0.553695
C -1.303833 2.365128 -0.110714
C -2.192950 1.146546 -0.363595
C -3.648460 1.433201 0.005809
C -4.563734 0.240654 -0.269838
C -4.051530 -1.023459 0.451816
C -4.866948 -2.243887 -0.036775
C -2.578215 -1.290412 0.110577
C -0.224093 -0.355314 0.154494
C -1.697750 -0.076438 0.414680
C 4.493760 -3.202204 -1.330072
C 3.532367 -2.772311 -0.419660
C 3.898701 -2.071598 0.729961
C -6.016621 0.556064 0.086138
C -5.684695 -3.000734 0.967761
C 6.218699 -2.257815 0.055111
C 5.255169 -1.828800 0.960095
C 5.840045 -2.940584 -1.098335
O -0.052008 -2.887326 -0.872819
C 0.459141 -2.070744 -3.078963
H 0.970954 3.544772 1.052954
H 1.936936 0.547654 3.502401
H -0.212427 4.467254 -0.803159
H 3.698758 0.435514 1.741452
H -2.048390 4.303051 -2.722599
H -2.780661 2.701447 -2.519728
H -1.021561 2.857563 -2.748714
H -3.623627 -1.484181 2.293754
H 2.245292 -0.261474 -0.557764
H -1.409543 -2.814231 0.478307
H 0.353516 1.844805 -1.386375
H 3.131329 -1.757528 2.706457
H 1.895282 -2.009535 1.487166
H 4.446419 0.303478 -1.035706
H 5.099538 1.353637 0.204652
H 4.818216 1.983511 -1.414156
H 3.233323 4.931913 0.179166
H 3.226886 4.580123 -1.534423
H 4.488103 3.894048 -0.498595
H -1.444741 2.690788 0.918107
H -2.139561 0.895566 -1.427368
H -4.016031 2.294229 -0.553967
H -3.701540 1.698115 1.065659
H -4.510815 0.009385 -1.337251
H -2.532473 -1.534150 -0.953198
H 0.099712 -1.214498 0.734550
H -1.785382 0.131884 1.481939
H 4.189388 -3.742632 -2.217820
H 2.487796 -2.993791 -0.597270
H -6.687454 -0.263848 -0.177796
H -6.348905 1.441771 -0.457690
H -6.121970 0.752487 1.153597
H -5.020051 -3.469069 1.698244
H -6.272616 -3.765089 0.464448
H -6.329783 -2.320133 1.524546
H 7.265479 -2.058764 0.248484
H 5.559487 -1.294616 1.852781
H 6.589708 -3.272208 -1.805628
H 1.537840 -1.922948 -3.173432
H -0.037133 -1.304899 -3.671925
H 0.206486 -3.063870 -3.440015
81
1-c5.out delta G = 1.4897 kcal/mol
conformer 6
O -0.347503 -0.139603 -1.954518
C -0.315266 -1.278727 -2.661615
C 1.012471 3.463343 0.001540
O 0.543589 1.044003 2.329633
C 1.194764 0.752230 1.333407
N 2.392742 0.141612 1.331631
O 0.468020 4.736756 -0.374982
C 3.023789 -0.036455 0.032595
O -1.956904 3.649652 -0.029983
C -2.500736 3.804290 -1.343359
O -3.474152 -1.434846 2.007174
C 2.135060 0.857466 -0.888117
C 2.832674 2.177868 -1.116640
O -1.798592 -2.558162 0.168020
C 2.369197 3.343411 -0.654288

O -4.916470 -2.387109 -1.124233
C 0.083748 2.313077 -0.406263
C 3.127319 -1.507868 -0.406104
C 0.783426 0.968851 -0.142637
C 4.146811 2.061634 -1.858109
C 3.152595 4.625627 -0.739957
C -1.301578 2.390015 0.264953
C -2.207692 1.203057 -0.064420
C -3.508434 1.256182 0.736941
C -4.441750 0.091121 0.409785
C -3.727181 -1.260550 0.615067
C -4.622536 -2.390843 0.055447
C -2.401991 -1.301914 -0.160854
C -0.151504 -0.199514 -0.511096
C -1.494110 -0.125243 0.202043
C 3.862830 -3.793757 2.543286
C 3.178341 -3.063886 1.575921
C 3.871286 -2.345603 0.599545
C -5.743796 0.178199 1.206854
C -5.100714 -3.453924 0.999647
C 5.955578 -3.103361 1.576497
C 5.267856 -2.374168 0.612359
C 5.254218 -3.815165 2.546423
O -0.209608 -2.380721 -2.156527
C -0.415133 -1.014222 -4.131708
H 1.132200 3.449762 1.094821
H 2.832796 -0.141370 2.194682
H -0.469926 4.702296 -0.118629
H 4.036567 0.361788 0.082932
H -3.404292 3.206379 -1.475880
H -1.779208 3.544118 -2.120720
H -2.759088 4.857017 -1.441894
H -2.799464 -2.122172 2.094006
H 2.004191 0.362046 -1.850420
H -1.145966 -2.757628 -0.524045
H -0.049059 2.380105 -1.489201
H 2.134473 -1.925466 -0.570828
H 3.643787 -1.522468 -1.368356
H 4.205085 1.111627 -2.391463
H 5.009076 2.108931 -1.187048
H 4.263232 2.858299 -2.592359
H 4.198646 4.450198 -0.980411
H 3.107885 5.155393 0.214800
H 2.732787 5.300914 -1.488536
H -1.157727 2.427473 1.343546
H -2.446681 1.234235 -1.131895
H -4.033093 2.191990 0.540110
H -3.270354 1.246389 1.804319
H -4.683388 0.135239 -0.655576
H -2.650668 -1.273893 -1.224128
H 0.332446 -1.142594 -0.280552
H -1.278284 -0.196694 1.269155
H 3.309578 -4.347220 3.291875
H 2.094850 -3.050971 1.575870
H -6.447795 -0.607202 0.924858
H -6.229435 1.137816 1.022431
H -5.550751 0.094638 2.276819
H -5.785346 -4.124107 0.484539
H -5.580948 -3.004289 1.869563
H -4.243110 -4.019398 1.373201
H 7.038416 -3.119070 1.569312
H 5.819451 -1.824589 -0.141872
H 5.788549 -4.384734 3.296333
H -0.488786 -1.953213 -4.672289
H 0.470026 -0.466586 -4.460009
H -1.284201 -0.389473 -4.338691
81
1-c4.out delta G = 2.0425 kcal/mol
conformer 7
O 0.361617 -0.287233 -1.720858
C 0.475164 -1.448151 -2.377975
C 1.125541 3.516908 -0.308051
O 0.243326 1.246628 2.485998
C 1.160913 1.097220 1.685104
N 2.453062 0.980538 2.025055
O 0.476279 4.621152 -0.954652
C 3.402873 0.754406 0.956746
O -1.830745 3.440207 -0.462721
C -2.465899 4.272978 0.511855
O -3.329170 -1.545229 1.780378
C 2.530524 0.939205 -0.330433
C 3.056343 2.146490 -1.068920
O -1.332397 -2.678298 0.317467
C 2.484315 3.350550 -0.965818
O -4.208551 -2.838341 -1.435662
C 0.297516 2.234700 -0.453241
C 4.154021 -0.583579 1.088017
C 1.050374 1.018501 0.141508

C 4.340076 1.863916 -1.802273
C 3.100046 4.624909 -1.479705
C -1.140551 2.294383 0.095460
C -1.914501 1.024608 -0.272900
C -3.333554 1.031790 0.296103
C -4.127027 -0.221790 -0.071986
C -3.371694 -1.494311 0.355498
C -4.091324 -2.731658 -0.230153
C -1.942022 -1.495220 -0.204262
C 0.280867 -0.259739 -0.260768
C -1.172943 -0.234966 0.199951
C 2.509958 -3.808285 -0.081518
C 3.278655 -2.648982 -0.060299
C 3.304644 -1.825408 1.066002
C -5.542897 -0.173256 0.502982
C -4.625983 -3.759812 0.722714
C 1.756516 -3.342887 2.152898
C 2.535947 -2.192635 2.175774
C 1.742389 -4.156435 1.023026
O 0.384459 -2.539315 -1.852819
C 0.747803 -1.222368 -3.834438
H 1.249684 3.761682 0.758231
H 2.725903 1.015374 2.996636
H -0.475409 4.410648 -0.943264
H 4.158314 1.540815 0.989959
H -2.949393 5.081203 -0.034044
H -1.731240 4.692344 1.205669
H -3.217437 3.720719 1.077664
H -2.617648 -2.154219 2.021927
H 2.657376 0.062808 -0.963932
H -0.562733 -2.873392 -0.244210
H 0.224998 2.054967 -1.528732
H 4.884971 -0.623701 0.278457
H 4.724659 -0.545983 2.020124
H 5.134269 1.543584 -1.120446
H 4.708498 2.716560 -2.365523
H 4.184231 1.040488 -2.505875
H 4.158607 4.510508 -1.698723
H 2.995674 5.414244 -0.731666
H 2.595263 4.983305 -2.378506
H -1.114499 2.405146 1.177831
H -1.978010 0.998470 -1.366733
H -3.876221 1.898935 -0.081327
H -3.287516 1.123463 1.385191
H -4.196359 -0.275084 -1.161641
H -2.020892 -1.561968 -1.291263
H 0.793116 -1.139751 0.121183
H -1.135790 -0.216497 1.289741
H 2.499436 -4.429488 -0.967267
H 3.870571 -2.382202 -0.927892
H -6.051263 0.732248 0.167544
H -5.519279 -0.166081 1.593278
H -6.142224 -1.027044 0.180389
H -5.156744 -4.533167 0.171819
H -5.281875 -3.291569 1.458012
H -3.798365 -4.204300 1.280993
H 1.164214 -3.608031 3.019715
H 2.554444 -1.580594 3.067919
H 1.136360 -5.053405 1.005525
H 0.092773 -0.446864 -4.229422
H 0.614889 -2.149954 -4.383946
H 1.777435 -0.876731 -3.949385
81
1-c3.out delta G = 2.5948 kcal/mol
conformer 8
O 0.343590 -0.194963 -1.663942
C 0.431424 -1.326899 -2.374160
C 1.133882 3.525976 0.014261
O 0.211993 1.195349 2.587985
C 1.137408 1.050186 1.795776
N 2.423146 0.893224 2.144763
O 0.487126 4.728044 -0.414072
C 3.387289 0.733794 1.075579
O -1.853405 3.477195 -0.334265
C -2.207092 3.472375 -1.719711
O -3.287749 -1.610052 1.860091
C 2.529392 0.983593 -0.209759
C 3.049545 2.235816 -0.879675
O -1.331953 -2.681520 0.309645
C 2.473023 3.429584 -0.698155
O -4.248897 -2.749002 -1.390882
C 0.304089 2.270892 -0.297675
C 4.150036 -0.602337 1.140683
C 1.042923 1.028157 0.250201
C 4.325473 1.996377 -1.640344
C 3.056044 4.737088 -1.158289
C -1.158443 2.310861 0.179451
C -1.921599 1.027549 -0.156256

C -3.314531 1.033888 0.472954
C -4.130198 -0.200303 0.091929
C -3.365582 -1.493148 0.441075
C -4.108704 -2.696963 -0.184154
C -1.947557 -1.477214 -0.151784
C 0.280227 -0.235124 -0.203562
C -1.165820 -0.233717 0.280406
C 2.563397 -3.750180 -0.286001
C 3.327321 -2.594404 -0.163715
C 3.313786 -1.846540 1.014615
C -5.522688 -0.169334 0.722247
C -4.637584 -3.761924 0.730996
C 1.736849 -3.434003 1.948662
C 2.511504 -2.286765 2.072619
C 1.761961 -4.171445 0.768213
O 0.333406 -2.439168 -1.897237
C 0.685751 -1.038902 -3.822990
H 1.292486 3.592142 1.101609
H 2.688543 0.896942 3.119080
H -0.466231 4.589103 -0.270497
H 4.135534 1.523522 1.161532
H -2.515606 4.489036 -1.955985
H -3.041132 2.796417 -1.916731
H -1.364146 3.202281 -2.359265
H -2.575742 -2.235360 2.054566
H 2.668507 0.143707 -0.888694
H -0.571690 -2.851728 -0.272329
H 0.304023 2.164972 -1.383546
H 4.901393 -0.583226 0.349199
H 4.696795 -0.619269 2.087629
H 5.118988 1.621215 -0.986056
H 4.701831 2.883359 -2.142363
H 4.160216 1.226058 -2.399727
H 2.465623 5.175639 -1.964604
H 4.083632 4.633968 -1.496149
H 3.041423 5.459933 -0.338816
H -1.179772 2.463728 1.255749
H -2.025958 0.974417 -1.244507
H -3.861232 1.925746 0.163275
H -3.213383 1.085463 1.560715
H -4.242109 -0.211551 -0.995562
H -2.050250 -1.501734 -1.238504
H 0.800922 -1.129116 0.131727
H -1.112152 -0.245064 1.368962
H 2.582383 -4.311220 -1.210908
H 3.946549 -2.270272 -0.991937
H -6.135318 -1.012483 0.396712
H -6.041722 0.746383 0.434177
H -5.457629 -0.195882 1.810491
H -5.217655 -4.483669 0.160098
H -5.241483 -3.320220 1.524306
H -3.800464 -4.267224 1.219595
H 1.115555 -3.754650 2.775397
H 2.496773 -1.731864 3.001370
H 1.158690 -5.065238 0.671517
H 0.043430 -0.230518 -4.170002
H 0.521996 -1.936298 -4.412914
H 1.721715 -0.713095 -3.939133

**Table S21.** Geometry data of conformers of compound **2**.

81

2-c20.out delta G = 0.0000 kcal/mol conformer 1

O -0.341508 0.104184 -2.046223
C -0.433163 -0.955694 -2.875850
C 2.336571 2.742459 0.022354
O 0.842567 0.714024 2.317255
C 1.299423 0.133069 1.331204
N 2.075120 -0.956871 1.362834
O 2.435089 3.013081 1.437984
C 2.670131 -1.351787 0.092282
O -0.223362 4.086119 -0.308678
C -0.131205 5.081431 0.704080
O -3.833247 0.316520 1.719031
C 2.314430 -0.135887 -0.824517
C 3.524197 0.764700 -0.947292
O -2.710062 -1.546379 0.002279
C 3.559786 2.024968 -0.500106
O -5.989652 -0.663114 0.712533
C 1.043641 2.045912 -0.393179
C 2.182452 -2.718427 -0.414214
C 1.094079 0.527516 -0.143862
C 4.720765 0.100855 -1.593707
C 4.796865 2.879713 -0.528021
C -0.206787 2.735349 0.173989
C -1.501423 2.048316 -0.259106
C -2.725045 2.685523 0.395157

C -4.027680 2.010782 -0.035905
C -3.963187 0.501760 0.312939
C -5.244242 -0.214566 -0.139983
C -2.739168 -0.163277 -0.346118
C -0.235385 -0.107175 -0.611951
C -1.443103 0.546589 0.049383
C 1.590830 -5.128907 2.467143
C 1.377937 -4.168108 1.483080
C 2.401590 -3.804334 0.605548
C -5.248232 2.691877 0.578986
C -5.534022 -0.351526 -1.603377
C 3.862136 -5.388670 1.715987
C 3.645542 -4.426698 0.734888
C 2.834565 -5.741589 2.586968
O -0.398163 -2.107365 -2.508919
C -0.587258 -0.499640 -4.297454
H 2.326072 3.734575 -0.433947
H 2.322766 -1.393871 2.238448
H 2.010996 2.293283 1.928822
H 3.748538 -1.415842 0.231734
H -0.988944 5.038129 1.383673
H -0.130550 6.046450 0.198411
H 0.789054 4.971868 1.283340
H -4.678152 -0.068954 2.010744
H 2.054621 -0.501626 -1.818540
H -2.791329 -1.583527 0.966741
H 0.985896 2.161485 -1.478920
H 1.130769 -2.665813 -0.688318
H 2.737410 -2.939839 -1.328746
H 5.234814 0.779367 -2.273805
H 4.414082 -0.773666 -2.169687
H 5.454365 -0.238142 -0.857009
H 4.732338 3.630680 -1.323029
H 5.709259 2.306455 -0.676918
H 4.888668 3.423181 0.415138
H -0.160097 2.740851 1.263549
H -1.586259 2.172201 -1.343672
H -2.770439 3.742082 0.127839
H -2.633691 2.627950 1.483634
H -4.095924 2.087589 -1.125155
H -2.855116 -0.127539 -1.429160
H -0.246060 -1.175654 -0.426811
H -1.323338 0.419158 1.127907
H 0.785153 -5.400398 3.138039
H 0.407730 -3.694225 1.392578
H -6.184794 2.276405 0.201792
H -5.239641 3.756984 0.342907
H -5.242032 2.585586 1.664914
H -6.592388 -0.553560 -1.752284
H -4.959090 -1.199158 -1.987103
H -5.233845 0.529188 -2.169040
H 4.831275 -5.864799 1.799295
H 4.448752 -4.157664 0.058315
H 3.001218 -6.490960 3.350535
H 0.213976 0.192861 -4.556432
H -1.532610 0.034672 -4.406101
H -0.572429 -1.357335 -4.963887
81
2-c27.out delta G = 0.3508 kcal/mol
conformer 2
O -0.381552 0.109940 -2.042552
C -0.335478 -0.937443 -2.891095
C 2.313826 2.583032 0.156702
O 0.402614 0.442401 2.358989
C 1.075712 0.021129 1.422762
N 1.990936 -0.956887 1.505901
O 2.618295 2.414871 1.558468
C 2.678751 -1.313962 0.278318
O -0.212056 4.058351 -0.034919
C -0.072305 4.970062 1.044251
O -4.065351 0.272092 1.491768
C 2.214773 -0.206461 -0.719680
C 3.370667 0.712163 -1.060874
O -2.911643 -1.478800 -0.335393
C 3.451500 1.974608 -0.626813
O -6.189189 -0.519870 0.273068
C 0.978026 1.976517 -0.256674
C 2.348629 -2.739515 -0.192854
C 0.978066 0.455555 -0.056284
C 4.400867 0.039605 -1.927366
C 4.606214 2.909965 -0.850927
C -0.249641 2.673990 0.342517
C -1.547774 2.064651 -0.192639
C -2.779895 2.698457 0.448590
C -4.081559 2.121894 -0.110342
C -4.098674 0.586952 0.103063
C -5.375847 -0.028299 -0.488952
C -2.861686 -0.067849 -0.541949

C -0.342052 -0.128937 -0.608031
C -1.568403 0.541532 0.002916
C 4.372511 -5.124947 1.971494
C 4.028564 -4.206463 0.985876
C 2.707112 -3.776634 0.837369
C -5.307616 2.802012 0.494929
C -5.580636 -0.017902 -1.973091
C 2.078121 -5.208006 2.691000
C 1.738021 -4.288887 1.702176
C 3.397133 -5.628626 2.828862
O -0.198631 -2.086927 -2.539695
C -0.477102 -0.473358 -4.311470
H 2.277090 3.652897 -0.067828
H 2.173044 -1.422699 2.382440
H 1.864455 2.711658 2.082570
H 3.754059 -1.246631 0.448009
H 0.888111 4.851143 1.559081
H -0.879505 4.855479 1.774471
H -0.115470 5.972648 0.621014
H -4.938460 -0.106974 1.695187
H 1.917094 -0.698696 -1.644344
H -3.040826 -1.609227 0.615430
H 0.910387 2.144700 -1.335248
H 1.288604 -2.798994 -0.441258
H 2.904942 -2.915667 -1.116468
H 4.786844 -0.870176 -1.459363
H 5.249119 0.677177 -2.159495
H 3.942402 -0.268399 -2.872415
H 4.901733 3.362486 0.099728
H 4.314418 3.731617 -1.513035
H 5.482484 2.428107 -1.277131
H -0.248863 2.592018 1.432017
H -1.571571 2.274194 -1.267803
H -2.768489 3.775125 0.274002
H -2.753059 2.542455 1.530842
H -4.082907 2.296043 -1.190380
H -2.903189 0.079113 -1.620628
H -0.382433 -1.202427 -0.448417
H -1.535444 0.335275 1.073533
H 5.400745 -5.450820 2.069011
H 4.792410 -3.821203 0.319975
H -6.236913 2.459349 0.035496
H -5.244808 3.881521 0.350147
H -5.366964 2.606996 1.566813
H -4.974253 -0.816179 -2.409999
H -5.260480 0.919400 -2.426632
H -6.626763 -0.208390 -2.201613
H 1.312360 -5.596644 3.350910
H 0.708765 -3.966495 1.597467
H 3.663466 -6.345289 3.595545
H -1.466925 -0.036048 -4.453062
H -0.347047 -1.312534 -4.988915
H 0.259186 0.301458 -4.526550
81
2-c7.out delta G = 0.4650 kcal/mol
conformer 3
O -0.199563 0.020484 -2.040535
C -0.583079 -1.035076 -2.774013
C 2.326767 2.798231 0.094036
O 0.826237 0.742863 2.355780
C 1.300390 0.167814 1.375872
N 2.070512 -0.927538 1.410798
O 2.379003 3.077514 1.509609
C 2.689871 -1.308179 0.146740
O -0.245392 4.105245 -0.290936
C -0.187052 5.097959 0.727295
O -3.937302 0.212285 1.578514
C 2.362048 -0.078221 -0.758104
C 3.568783 0.831877 -0.830967
O -2.719267 -1.537785 -0.124634
C 3.574690 2.093287 -0.385439
O -5.338113 -0.118262 -1.696037
C 1.053644 2.084344 -0.354967
C 2.204809 -2.662458 -0.396395
C 1.120653 0.569431 -0.102135
C 4.793634 0.179183 -1.433529
C 4.802050 2.962140 -0.369816
C -0.218778 2.752667 0.184660
C -1.493355 2.050401 -0.282130
C -2.733134 2.651327 0.379116
C -4.029369 1.974811 -0.066286
C -3.962561 0.451557 0.172840
C -5.188541 -0.215223 -0.493401
C -2.707150 -0.148811 -0.475022
C -0.183283 -0.098548 -0.587811
C -1.430922 0.546109 0.002381
C 3.888387 -5.385704 1.659614
C 3.669961 -4.396560 0.706573

C 2.423488 -3.776212 0.593190
C -5.250094 2.606397 0.603179
C -6.157682 -0.956856 0.379245
C 1.614381 -5.157450 2.412952
C 1.399321 -4.169059 1.457042
C 2.860304 -5.768218 2.517375
O -0.992676 -2.075046 -2.292829
C -0.429093 -0.770502 -4.239355
H 2.317992 3.787696 -0.367652
H 2.302576 -1.372622 2.286653
H 1.969924 2.343816 1.992563
H 3.764205 -1.384884 0.307115
H -1.054622 5.036686 1.392818
H -0.196072 6.064614 0.224917
H 0.725665 5.002586 1.320619
H -3.621714 -0.692128 1.711066
H 2.130050 -0.424481 -1.765496
H -2.153508 -2.004598 -0.761669
H 1.020917 2.200044 -1.442292
H 1.150688 -2.609593 -0.665524
H 2.760090 -2.857848 -1.316401
H 4.514131 -0.690183 -2.030923
H 5.496683 -0.165728 -0.670246
H 5.333298 0.866342 -2.084249
H 5.726324 2.398998 -0.478493
H 4.849439 3.512195 0.572715
H 4.760363 3.707384 -1.171705
H -0.197825 2.751879 1.275003
H -1.565334 2.195234 -1.365812
H -2.793583 3.714215 0.140716
H -2.636948 2.567853 1.465779
H -4.125823 2.097214 -1.148366
H -2.816440 -0.035184 -1.555730
H -0.168500 -1.154947 -0.342570
H -1.366981 0.400033 1.083350
H 4.859338 -5.859953 1.731115
H 4.473534 -4.104864 0.039960
H -5.218170 2.465595 1.684151
H -6.183915 2.180053 0.231363
H -5.272907 3.678247 0.399836
H -5.657736 -1.821346 0.823628
H -7.007016 -1.289759 -0.213211
H -6.486610 -0.327207 1.206829
H 0.808462 -5.451805 3.073738
H 0.426801 -3.697791 1.378867
H 3.028439 -6.539043 3.258894
H -0.863647 -1.586241 -4.809606
H 0.632101 -0.678756 -4.478024
H -0.908343 0.172767 -4.501114
81
2-c15.out delta G = 0.6288 kcal/mol
conformer 4
O -0.504353 -0.537397 -1.363321
C -0.569564 -1.814340 -1.786764
C 2.309791 2.389945 -0.095754
O 0.230792 1.305267 2.653717
C 0.891108 0.540023 1.956757
N 1.751532 -0.379759 2.419422
O 2.590858 2.706705 1.284826
C 2.435550 -1.194958 1.432444
O -0.103817 3.847592 -0.884326
C 0.066974 5.073696 -0.189653
O -4.242583 1.110575 1.724031
C 2.037136 -0.518615 0.080305
C 3.242940 0.148933 -0.554279
O -3.182492 -1.250412 0.721124
C 3.405705 1.476424 -0.586175
O -6.394069 0.058179 0.779821
C 0.941481 1.755575 -0.312236
C 2.039333 -2.679268 1.549970
C 0.836987 0.410209 0.418589
C 4.230174 -0.834929 -1.121450
C 4.617999 2.201138 -1.100935
C -0.243145 2.691742 -0.044780
C -1.570770 2.009367 -0.380908
C -2.766154 2.904984 -0.066000
C -4.093113 2.243254 -0.439190
C -4.224546 0.897026 0.316066
C -5.529899 0.184784 -0.069252
C -3.026494 -0.023534 0.010675
C -0.513698 -0.253018 0.064016
C -1.701023 0.663772 0.346835
C 5.047041 -4.604521 0.249189
C 4.217671 -3.844052 1.065071
C 2.900562 -3.566469 0.689082
C -5.279762 3.169253 -0.180151
C -5.698823 -0.342532 -1.461582
C 3.256149 -4.843328 -1.338389

C 2.428081 -4.081006 -0.518416
C 4.569266 -5.103122 -0.960603
O -0.630147 -2.770360 -1.046649
C -0.520972 -1.882294 -3.284813
H 2.345625 3.313171 -0.681192
H 1.911204 -0.482329 3.410956
H 1.858649 3.222229 1.644357
H 3.511762 -1.111587 1.587702
H 0.101646 5.862449 -0.939888
H 1.003472 5.093686 0.379760
H -0.764955 5.267958 0.494557
H -5.146650 0.890624 2.009909
H 1.715728 -1.304281 -0.600994
H -3.343554 -1.009376 1.645152
H 0.898002 1.530266 -1.381240
H 2.142926 -2.971257 2.598169
H 0.988284 -2.793390 1.281293
H 4.667772 -1.466254 -0.344733
H 5.041505 -0.354084 -1.660520
H 3.720295 -1.513802 -1.810589
H 4.386155 2.734844 -2.028508
H 5.468792 1.550865 -1.287694
H 4.928050 2.956652 -0.373736
H -0.263602 3.000759 1.002767
H -1.559019 1.817600 -1.459608
H -2.673908 3.842909 -0.615054
H -2.774361 3.149060 1.000192
H -4.057354 2.011247 -1.507790
H -3.040589 -0.284858 -1.047110
H -0.631799 -1.195520 0.590932
H -1.696737 0.856430 1.420641
H 6.065371 -4.808715 0.556226
H 4.597273 -3.458265 2.004572
H -5.378210 3.380628 0.885792
H -6.221234 2.740689 -0.529733
H -5.133960 4.116848 -0.700832
H -5.193896 -1.310831 -1.522374
H -5.248348 0.308597 -2.209151
H -6.755429 -0.488166 -1.675105
H 2.873546 -5.233490 -2.273616
H 1.406120 -3.884188 -0.815310
H 5.214130 -5.694462 -1.598556
H 0.498238 -1.664283 -3.611620
H -1.178274 -1.134416 -3.726358
H -0.798107 -2.878558 -3.618086
81
2-c4.out delta G = 0.8302 kcal/mol
conformer 5
O -0.355300 -0.609479 -1.303157
C -0.790127 -1.814035 -1.690852
C 2.343195 2.462756 -0.219908
O 0.387750 1.389859 2.639607
C 1.053287 0.634044 1.939332
N 1.972182 -0.235344 2.391297
O 2.658497 2.844222 1.136378
C 2.594490 -1.097423 1.402780
O -0.147513 3.833039 -0.933571
C 0.013922 5.079810 -0.274388
O -4.181352 0.942308 1.867194
C 2.154857 -0.449795 0.052022
C 3.329367 0.231606 -0.622347
O -3.055756 -1.309471 0.835958
C 3.451256 1.561158 -0.704881
O -5.493421 -0.243247 -1.241556
C 0.985721 1.785303 -0.364501
C 2.158984 -2.565422 1.585886
C 0.947867 0.459621 0.406418
C 4.337290 -0.742007 -1.172902
C 4.633371 2.299294 -1.268161
C -0.213494 2.692239 -0.064972
C -1.537026 1.968728 -0.325441
C -2.736251 2.826201 0.074526
C -4.069473 2.140655 -0.223280
C -4.137756 0.756339 0.453989
C -5.392689 0.010306 -0.056731
C -2.916043 -0.097939 0.084536
C -0.387694 -0.256070 0.114307
C -1.602516 0.619487 0.402287
C 2.706861 -4.747152 -1.484139
C 2.088882 -3.960605 -0.516193
C 2.804368 -3.483914 0.583046
C -5.254826 3.026235 0.161574
C -6.449542 -0.368803 0.938257
C 4.773754 -4.615938 -0.260704
C 4.152464 -3.829637 0.702498
C 4.053850 -5.072308 -1.362230
O -1.307984 -2.619143 -0.938066
C -0.529568 -2.053085 -3.145653

H 2.334690 3.361170 -0.843821
H 2.158482 -0.318978 3.379971
H 1.950522 3.403878 1.477664
H 3.677703 -1.044286 1.510933
H -0.016280 5.851856 -1.041922
H 0.976503 5.146128 0.246244
H -0.789032 5.261407 0.446738
H -3.934479 0.100340 2.274080
H 1.831454 -1.245706 -0.616187
H -2.494979 -1.981452 0.413148
H 0.905916 1.530721 -1.424899
H 2.427516 -2.870054 2.600596
H 1.072247 -2.628854 1.511276
H 5.087429 -0.262562 -1.795583
H 3.830526 -1.500612 -1.775517
H 4.856331 -1.283388 -0.378586
H 4.369540 2.795645 -2.207798
H 5.497033 1.664787 -1.449860
H 4.937259 3.085941 -0.572163
H -0.193932 3.022227 0.976001
H -1.583479 1.783237 -1.404721
H -2.704028 3.774736 -0.463596
H -2.676246 3.055698 1.142616
H -4.119087 1.943195 -1.297529
H -2.982714 -0.311567 -0.984628
H -0.446939 -1.179661 0.684808
H -1.585833 0.810372 1.476051
H 2.134806 -5.105025 -2.331172
H 1.036162 -3.724661 -0.610379
H -6.208412 2.575777 -0.121237
H -5.181055 3.988621 -0.347492
H -5.270842 3.210134 1.236331
H -6.750290 0.499772 1.525257
H -6.039667 -1.096008 1.643853
H -7.304228 -0.800320 0.421925
H 5.820342 -4.872568 -0.152948
H 4.721539 -3.475956 1.554628
H 4.538013 -5.682519 -2.114233
H -0.777288 -1.167728 -3.729358
H -1.101582 -2.910779 -3.487755
H 0.535813 -2.253305 -3.280294

81

2-c12.out delta G = 0.9086 kcal/mol

conformer 6

O -0.497345 -0.536860 -1.363966
C -0.710691 -1.801204 -1.777197
C 2.324267 2.471385 -0.211160
O 0.610006 1.482734 2.567986
C 1.044634 0.563782 1.871837
N 1.723224 -0.498593 2.322549
O 2.417953 3.200143 1.032497
C 2.349381 -1.332643 1.304721
O -0.111862 3.773485 -1.134266
C 0.058058 5.054754 -0.538935
O -4.069007 1.235670 1.898130
C 2.095463 -0.510003 -0.001477
C 3.369231 0.206431 -0.397927
O -3.049903 -1.191395 1.035933
C 3.495862 1.538346 -0.410809
O -6.268407 0.099866 1.190608
C 0.990441 1.760826 -0.422200
C 1.823224 -2.778447 1.301744
C 0.913407 0.428361 0.343211
C 4.512447 -0.720288 -0.748109
C 4.791544 2.248954 -0.691059
C -0.214997 2.687011 -0.203741
C -1.547495 1.975224 -0.436992
C -2.733750 2.883189 -0.122679
C -4.073158 2.184212 -0.356978
C -4.142428 0.905538 0.514599
C -5.464103 0.158560 0.277852
C -2.958884 -0.033468 0.208779
C -0.450922 -0.241311 0.059034
C -1.618939 0.683552 0.387033
C 3.103386 -4.835370 -1.640610
C 2.265405 -4.079985 -0.823940
C 2.686122 -3.665574 0.440008
C -5.251883 3.120975 -0.100692
C -5.719790 -0.482629 -1.052069
C 4.799931 -4.791774 0.063642
C 3.961091 -4.037577 0.875151
C 4.374822 -5.189498 -1.202002
O -0.860503 -2.740066 -1.028626
C -0.701139 -1.879859 -3.275290
H 2.397995 3.246633 -0.976836
H 1.927214 -0.601587 3.306388
H 1.919035 2.727438 1.715371
H 3.417531 -1.373208 1.513103

H 0.145621 5.771827 -1.354433
H 0.961005 5.089754 0.076053
H -0.805319 5.324434 0.078652
H -4.950123 1.036866 2.261062
H 1.825390 -1.193022 -0.806279
H -3.162274 -0.866853 1.941534
H 0.970870 1.489247 -1.481033
H 1.837799 -3.139242 2.333327
H 0.789780 -2.806212 0.958799
H 4.137862 -1.699497 -1.047482
H 5.187502 -0.883527 0.096705
H 5.108949 -0.323612 -1.568594
H 4.792405 2.673966 -1.700809
H 5.662776 1.605086 -0.593311
H 4.908442 3.083805 0.003784
H -0.200599 3.079555 0.813888
H -1.590247 1.707832 -1.497754
H -2.686147 3.773885 -0.750503
H -2.680622 3.214371 0.918364
H -4.102462 1.863867 -1.402669
H -3.046534 -0.388250 -0.817831
H -0.550420 -1.175870 0.601366
H -1.531749 0.944323 1.444431
H 2.760037 -5.147745 -2.619188
H 1.273395 -3.816172 -1.165579
H -5.151135 4.021867 -0.707718
H -5.284613 3.421259 0.947810
H -6.208397 2.657756 -0.351162
H -5.331752 0.110638 -1.878684
H -6.786009 -0.655991 -1.179341
H -5.204453 -1.447201 -1.070561
H 5.784842 -5.070295 0.417586
H 4.301141 -3.729639 1.857485
H 5.027877 -5.775202 -1.836705
H 0.322227 -1.736820 -3.628893
H -1.313779 -1.087847 -3.704462
H -1.059358 -2.854222 -3.595402

81

2-c28.out delta G = 0.9500 kcal/mol

conformer 7

O -0.416691 0.164010 -1.974476
C -0.399389 -0.865346 -2.846106
C 2.334652 2.598050 0.186541
O 0.462347 0.352857 2.410866
C 1.130975 -0.013102 1.450092
N 2.075528 -0.967763 1.489015
O 2.638479 2.477880 1.590778
C 2.700659 -1.320452 0.228381
O -0.136131 4.080208 0.423943
C -0.272108 4.664478 -0.866085
O -4.025120 0.261266 1.636553
C 2.211928 -0.189941 -0.729590
C 3.366032 0.732137 -1.065712
O -2.921227 -1.439342 -0.260705
C 3.465350 1.980479 -0.596252
O -6.180578 -0.468939 0.433100
C 0.992643 1.984638 -0.192810
C 2.322657 -2.732620 -0.249284
C 0.994790 0.459214 -0.016826
C 4.375709 0.081342 -1.972721
C 4.633048 2.904258 -0.805497
C -0.208173 2.647271 0.497063
C -1.527117 2.088075 -0.044916
C -2.737742 2.709839 0.646441
C -4.055306 2.162227 0.096084
C -4.081357 0.621879 0.259897
C -5.375062 0.037058 -0.327644
C -2.862877 -0.023920 -0.428816
C -0.344332 -0.105305 -0.546501
C -1.553532 0.559222 0.107027
C 2.106048 -5.232295 2.611980
C 1.750087 -4.290163 1.650832
C 2.695691 -3.792666 0.751893
C -5.262792 2.832766 0.748030
C -5.604071 0.093902 -1.807299
C 4.369216 -5.202625 1.795819
C 4.009631 -4.260832 0.838123
C 3.417347 -5.691051 2.687647
O -0.273704 -2.024060 -2.522220
C -0.542814 -0.367017 -4.254709
H 2.296510 3.661866 -0.062951
H 2.268967 -1.465576 2.345318
H 2.007848 3.021343 2.079138
H 3.783340 -1.275534 0.349724
H -1.272940 4.515180 -1.280943
H 0.463215 4.279706 -1.578187
H -0.104198 5.732291 -0.736426
H -4.900918 -0.108208 1.845903

H 1.892158 -0.656615 -1.660263
H -3.036924 -1.594795 0.688166
H 0.889312 2.153761 -1.267821
H 1.253407 -2.767286 -0.459669
H 2.841106 -2.906763 -1.195106
H 5.234336 0.712872 -2.182532
H 3.900721 -0.167239 -2.927177
H 4.746658 -0.859751 -1.557957
H 5.522725 2.403627 -1.178950
H 4.891403 3.385356 0.141350
H 4.374063 3.704711 -1.506345
H -0.166375 2.442983 1.565604
H -1.583627 2.313564 -1.114774
H -2.724053 3.792665 0.517191
H -2.684770 2.516737 1.721503
H -4.075994 2.371752 -0.977657
H -2.923964 0.153159 -1.502010
H -0.389657 -1.181370 -0.408084
H -1.499907 0.322864 1.170361
H 1.358509 -5.609233 3.299013
H 0.726564 -3.938694 1.594432
H -5.301785 2.603977 1.814206
H -6.204507 2.512984 0.297579
H -5.193128 3.915826 0.636643
H -5.036445 -0.716576 -2.273093
H -5.254862 1.028802 -2.243343
H -6.660229 -0.051447 -2.022853
H 5.391028 -5.558207 1.844815
H 4.754861 -3.887519 0.144915
H 3.695556 -6.425802 3.432685
H -0.583322 -1.207805 -4.941319
H 0.306858 0.271185 -4.502865
H -1.446138 0.236706 -4.346714
81
2-c9.out delta G = 1.0655 kcal/mol
conformer 8
O -0.276189 0.077731 -2.049623
C -0.665126 -0.916171 -2.859847
C 2.324847 2.599074 0.159712
O 0.408925 0.443115 2.363854
C 1.092628 0.031795 1.432170
N 2.018871 -0.936618 1.517507
O 2.633771 2.429541 1.559899
C 2.703609 -1.295452 0.288916
O -0.214295 4.060140 -0.010227
C -0.075246 4.967235 1.072834
O -4.139787 0.191042 1.436771
C 2.235230 -0.192594 -0.710475
C 3.385974 0.729642 -1.057681
O -2.940440 -1.460918 -0.360363
C 3.461691 1.993744 -0.627461
O -5.423183 0.241437 -1.900799
C 0.989184 1.987974 -0.249136
C 2.372902 -2.723253 -0.176026
C 0.998022 0.467855 -0.047907
C 4.416428 0.059860 -1.925789
C 4.610609 2.934284 -0.859375
C -0.239418 2.673392 0.358841
C -1.537381 2.060566 -0.174329
C -2.765058 2.660838 0.508332
C -4.075434 2.096669 -0.040279
C -4.093860 0.557566 0.059650
C -5.322300 0.018697 -0.709801
C -2.844121 -0.050838 -0.594925
C -0.312311 -0.127098 -0.604834
C -1.554516 0.533935 -0.016119
C 4.490388 -5.097649 1.906762
C 4.103143 -4.181207 0.935629
C 2.774127 -3.760293 0.838692
C -5.288187 2.728814 0.643551
C -6.353062 -0.761968 0.051292
C 2.225586 -5.196391 2.713312
C 1.841617 -4.279262 1.738771
C 3.551709 -5.607870 2.800465
O -1.141988 -1.962938 -2.461996
C -0.421145 -0.579607 -4.298480
H 2.284483 3.668770 -0.064403
H 2.202157 -1.401717 2.394262
H 1.900272 2.767761 2.087743
H 3.779562 -1.228026 0.453178
H 0.892489 4.859342 1.576451
H -0.872418 4.837278 1.811322
H -0.136790 5.971561 0.656134
H -3.864836 -0.734318 1.495844
H 1.932240 -0.682010 -1.635928
H -2.352716 -1.904045 -0.995333
H 0.917026 2.157410 -1.327268
H 1.303415 -2.795247 -0.382099

H 2.898004 -2.892776 -1.118683
H 3.955697 -0.254536 -2.867638
H 4.810504 -0.845464 -1.455904
H 5.259278 0.702202 -2.163946
H 5.486414 2.456514 -1.291075
H 4.910048 3.388899 0.089052
H 4.310286 3.753982 -1.520043
H -0.232246 2.583816 1.447460
H -1.578635 2.299457 -1.243337
H -2.765790 3.743210 0.371055
H -2.710280 2.469854 1.584121
H -4.123620 2.321068 -1.109119
H -2.907458 0.155363 -1.665892
H -0.337428 -1.199298 -0.424841
H -1.540195 0.301695 1.049305
H 5.523695 -5.416672 1.964832
H 4.838493 -3.790401 0.241551
H -6.227344 2.389043 0.202263
H -5.249311 3.814484 0.540814
H -5.304687 2.487751 1.707005
H -5.902799 -1.681662 0.433319
H -7.187990 -1.005686 -0.601978
H -6.694549 -0.197856 0.920175
H 1.488223 -5.590640 3.401545
H 0.806790 -3.964320 1.673386
H 3.851960 -6.322909 3.556009
H -0.917733 -1.304278 -4.937233
H 0.653758 -0.601542 -4.489687
H -0.774990 0.427498 -4.516307
81
2-c5.out delta G = 1.0680 kcal/mol
conformer 9
O -0.358559 -0.624762 -1.317114
C -0.823783 -1.828871 -1.673001
C 2.334417 2.534014 -0.254736
O 0.669234 1.549110 2.561249
C 1.129463 0.635201 1.876395
N 1.860155 -0.388923 2.335060
O 2.428038 3.286690 0.974477
C 2.462861 -1.245583 1.322538
O -0.152620 3.770279 -1.137371
C 0.001390 5.058703 -0.552585
O -4.089733 1.025554 1.925551
C 2.181100 -0.448509 0.007705
C 3.432949 0.291910 -0.412933
O -2.988856 -1.281807 0.958491
C 3.525787 1.626079 -0.453272
O -5.507307 -0.329808 -1.066010
C 1.014310 1.789403 -0.435454
C 1.923769 -2.686607 1.371288
C 0.982309 0.468173 0.350329
C 4.593391 -0.612492 -0.762842
C 4.799338 2.363404 -0.764168
C -0.209256 2.686258 -0.200325
C -1.528876 1.943230 -0.406401
C -2.723222 2.815416 -0.021512
C -4.062378 2.108982 -0.229908
C -4.097716 0.763417 0.523843
C -5.365147 -0.015115 0.099680
C -2.885345 -0.102267 0.152945
C -0.360078 -0.248688 0.092678
C -1.565454 0.634439 0.390055
C 2.732665 -4.800865 -1.691276
C 2.035271 -4.027994 -0.766931
C 2.649998 -3.585947 0.404699
C -5.236650 3.010310 0.152727
C -6.380550 -0.350793 1.151540
C 4.677536 -4.724560 -0.277814
C 3.977719 -3.951651 0.640905
C 4.058161 -5.147248 -1.451713
O -1.317970 -2.618236 -0.888773
C -0.632424 -2.082110 -3.135890
H 2.379228 3.297405 -1.034260
H 2.075903 -0.472019 3.318220
H 1.955470 2.813397 1.675021
H 3.535303 -1.289084 1.504879
H 0.051360 5.772818 -1.373798
H 0.917924 5.118631 0.039674
H -0.852866 5.310940 0.084659
H -3.836184 0.205532 2.370988
H 1.917929 -1.146420 -0.785942
H -2.458804 -1.973266 0.528882
H 0.983207 1.504827 -1.490786
H 2.053874 -3.053545 2.392681
H 0.854171 -2.698496 1.163476
H 4.237619 -1.601834 -1.051739
H 5.277622 -0.754465 0.078621
H 5.175832 -0.210561 -1.590661

H 4.777327 2.766363 -1.782648
H 5.687964 1.744340 -0.663283
H 4.902531 3.215621 -0.088588
H -0.185005 3.082899 0.815159
H -1.598925 1.700850 -1.472596
H -2.714441 3.729730 -0.616753
H -2.629294 3.111189 1.027676
H -4.153566 1.852463 -1.288740
H -2.988183 -0.362070 -0.902781
H -0.413440 -1.160219 0.679567
H -1.513083 0.874552 1.454468
H 2.238327 -5.133122 -2.595690
H 0.998399 -3.778210 -0.950159
H -5.211010 3.252922 1.215640
H -6.198239 2.541900 -0.066889
H -5.188397 3.943572 -0.410734
H -5.937992 -1.040118 1.875304
H -7.250219 -0.813263 0.689976
H -6.666855 0.543146 1.706806
H 5.706491 -4.997270 -0.078934
H 4.469759 -3.624131 1.549433
H 4.603196 -5.747451 -2.169269
H -1.145489 -2.995156 -3.423076
H 0.435042 -2.180736 -3.343036
H -1.003761 -1.237268 -3.715347
81
2-c3.out delta G = 1.5380 kcal/mol
conformer 10
O 0.204498 -0.334820 -1.700333
C -0.170852 -1.414455 -2.396042
C 2.410999 2.764295 -0.113071
O 0.587546 0.955204 2.560692
C 1.387115 0.465213 1.768588
N 2.466481 -0.250933 2.116306
O 2.780710 2.911234 1.275875
C 3.337617 -0.696633 1.048992
O -0.271760 3.886526 -0.509383
C -0.224219 5.013048 0.352044
O -3.750270 -0.042520 1.748041
C 2.653277 -0.124386 -0.236473
C 3.628430 0.791006 -0.947599
O -2.390687 -1.851362 0.238159
C 3.569671 2.122706 -0.838391
O -5.050476 -0.720005 -1.513591
C 1.144526 1.945650 -0.319710
C 3.568570 -2.218885 1.064368
C 1.312867 0.516766 0.225983
C 4.688219 0.037098 -1.702619
C 4.568139 3.098214 -1.393362
C -0.149041 2.615653 0.149374
C -1.369863 1.770750 -0.226000
C -2.652890 2.354269 0.362800
C -3.898066 1.567220 -0.043966
C -3.749837 0.077919 0.326530
C -4.916697 -0.715771 -0.304986
C -2.444584 -0.503431 -0.238919
C 0.098292 -0.315412 -0.241674
C -1.223719 0.304744 0.206596
C 1.044029 -4.522794 -0.605036
C 2.163688 -3.728911 -0.382871
C 2.346744 -3.067635 0.833667
C -5.167301 2.182630 0.546263
C -5.852174 -1.452326 0.608043
C 0.243989 -4.001345 1.600176
C 1.372091 -3.219503 1.824626
C 0.077472 -4.658070 0.384671
O -0.743673 -2.370928 -1.914028
C 0.227944 -1.298653 -3.836278
H 2.245143 3.756933 -0.539883
H 2.707747 -0.363299 3.090054
H 2.070819 3.371131 1.740681
H 4.314870 -0.225215 1.173838
H -0.403986 5.893336 -0.263680
H 0.755185 5.121560 0.833042
H -0.992632 4.957838 1.129470
H -3.362581 -0.903092 1.959658
H 2.432453 -0.956276 -0.903771
H -1.767311 -2.326690 -0.338510
H 1.047004 1.852441 -1.403680
H 4.317590 -2.443288 0.303185
H 4.019466 -2.471372 2.028103
H 5.373688 0.685165 -2.241600
H 4.218098 -0.636056 -2.425931
H 5.281734 -0.592175 -1.032487
H 4.887047 3.784939 -0.603863
H 4.115379 3.711773 -2.178939
H 5.457629 2.626251 -1.803150
H -0.137882 2.764538 1.230348

H -1.439128 1.801844 -1.319666
H -2.769939 3.387725 0.032600
H -2.573429 2.368568 1.453861
H -3.976142 1.590230 -1.134182
H -2.531266 -0.496001 -1.327361
H 0.183985 -1.340201 0.113044
H -1.201003 0.275919 1.296235
H 0.919582 -5.022757 -1.556833
H 2.911503 -3.626950 -1.160663
H -6.067947 1.674147 0.196096
H -5.244529 3.230608 0.251861
H -5.151767 2.135956 1.635745
H -6.658958 -1.896688 0.029302
H -6.248741 -0.779762 1.369819
H -5.302702 -2.234445 1.137683
H -0.503204 -4.101093 2.377537
H 1.497560 -2.727054 2.779748
H -0.800798 -5.266428 0.209647
H 1.305933 -1.457246 -3.913543
H 0.011964 -0.300345 -4.214352
H -0.289435 -2.050450 -4.425622

81

2-c2.out delta G = 1.6824 kcal/mol

conformer 11

O -0.345665 -0.539799 -1.288053
C -0.785633 -1.730360 -1.712018
C 2.343880 2.514472 -0.145152
O 0.388818 1.299479 2.730845
C 1.064868 0.589616 1.995442
N 1.994799 -0.287989 2.412324
O 2.604475 2.981621 1.193276
C 2.593764 -1.132757 1.395912
O -0.092180 4.011896 -0.503720
C -0.204237 4.085066 -1.920256
O -4.161058 0.920246 1.938907
C 2.170335 -0.423401 0.071920
C 3.361633 0.288982 -0.533981
O -3.044967 -1.299724 0.833180
C 3.479881 1.620567 -0.567639
O -5.489271 -0.167188 -1.198414
C 0.996064 1.818054 -0.286621
C 2.128814 -2.596669 1.525302
C 0.963105 0.473211 0.454523
C 4.413210 -0.652800 -1.064616
C 4.697446 2.366749 -1.039209
C -0.197538 2.707022 0.087347
C -1.525715 2.012775 -0.229634
C -2.721987 2.858060 0.202989
C -4.056601 2.183973 -0.113090
C -4.124045 0.778790 0.520457
C -5.382840 0.051048 -0.007049
C -2.905400 -0.065871 0.119113
C -0.376169 -0.229958 0.139521
C -1.589919 0.639116 0.455218
C 2.684622 -4.703066 -1.596464
C 2.063688 -3.941368 -0.610552
C 2.776626 -3.489553 0.500642
C -5.239831 3.058170 0.302842
C -6.436841 -0.353538 0.981047
C 4.749423 -4.597271 -0.367188
C 4.125342 -3.835600 0.613704
C 4.032057 -5.028277 -1.480604
O -1.305588 -2.556229 -0.983638
C -0.525937 -1.927592 -3.173145
H 2.339336 3.381987 -0.811085
H 2.164604 -0.422578 3.398403
H 1.995713 3.708286 1.374968
H 3.677337 -1.109768 1.505883
H 0.526980 3.450799 -2.429137
H -0.011202 5.122283 -2.188751
H -1.205379 3.813857 -2.266276
H -3.913236 0.065717 2.318068
H 1.857103 -1.182285 -0.642852
H -2.487479 -1.959603 0.387562
H 0.909821 1.568630 -1.347299
H 2.379223 -2.938901 2.532694
H 1.042547 -2.641803 1.433829
H 5.028814 -1.074886 -0.266023
H 5.078620 -0.168453 -1.774956
H 3.940212 -1.499755 -1.566362
H 5.577585 1.736636 -1.141505
H 4.935200 3.159550 -0.325312
H 4.509984 2.854097 -2.001826
H -0.168207 2.908414 1.156487
H -1.581370 1.847456 -1.310833
H -2.690736 3.828282 -0.294896
H -2.655006 3.050496 1.277736
H -4.109238 2.020491 -1.193036

H -2.976611 -0.245702 -0.956004
H -0.439968 -1.170225 0.681444
H -1.569924 0.796902 1.534116
H 2.114355 -5.041939 -2.452476
H 1.010747 -3.705634 -0.699607
H -5.167055 4.035481 -0.177053
H -5.251342 3.209387 1.382728
H -6.194808 2.617304 0.010036
H -6.026156 -1.100676 1.664908
H -7.294613 -0.768536 0.456390
H -6.733016 0.499399 1.592794
H 5.796395 -4.854287 -0.264141
H 4.693251 -3.501614 1.474464
H 4.518558 -5.619052 -2.246492
H -0.745858 -1.017635 -3.729181
H -1.119246 -2.758068 -3.545048
H 0.534019 -2.154420 -3.309256
81
2-c13.out delta G = 1.7087 kcal/mol
conformer 12
O -0.305696 -0.602616 -1.316186
C -0.714443 -1.807971 -1.727069
C 2.330196 2.471347 -0.188217
O 0.255979 1.324219 2.674043
C 0.984087 0.622797 1.982500
N 1.936438 -0.202750 2.454496
O 2.493121 2.914614 1.177133
C 2.579613 -1.071552 1.487015
O -0.141463 3.857288 -0.860553
C 0.091395 5.068215 -0.153164
O -4.248759 0.886948 1.738471
C 2.156531 -0.442510 0.123318
C 3.338395 0.235393 -0.539953
O -3.078821 -1.333412 0.704555
C 3.451762 1.564391 -0.640354
O -5.444137 -0.230079 -1.441591
C 0.982139 1.788957 -0.327726
C 2.146347 -2.540153 1.676602
C 0.934394 0.460730 0.444079
C 4.358633 -0.741436 -1.061656
C 4.630800 2.296450 -1.222312
C -0.219681 2.691011 -0.028408
C -1.533269 1.972195 -0.342533
C -2.746331 2.816806 0.041642
C -4.066623 2.135782 -0.317585
C -4.153323 0.735121 0.323614
C -5.386695 -0.001999 -0.248635
C -2.915804 -0.107995 -0.020120
C -0.388606 -0.258885 0.102274
C -1.617407 0.608193 0.356321
C 4.795977 -4.600765 -0.107910
C 4.155981 -3.809671 0.839017
C 2.810248 -3.465209 0.692104
C -5.267961 3.008242 0.047080
C -6.478541 -0.403838 0.698726
C 2.752833 -4.739674 -1.369700
C 2.116002 -3.948347 -0.417984
C 4.097434 -5.063357 -1.220524
O -1.253768 -2.622681 -0.999782
C -0.394717 -2.037540 -3.171580
H 2.323140 3.351317 -0.838031
H 2.075993 -0.303386 3.449335
H 3.381914 3.276313 1.270886
H 3.660870 -1.013447 1.611010
H 0.154530 5.862759 -0.895906
H 1.025616 5.027718 0.414210
H -0.730855 5.290156 0.535745
H -4.007463 0.037381 2.132852
H 1.852009 -1.249560 -0.540313
H -2.494392 -1.994718 0.296888
H 0.907926 1.528134 -1.386831
H 2.399441 -2.835387 2.698036
H 1.061006 -2.606934 1.584855
H 4.862804 -1.273982 -0.252031
H 5.120264 -0.268327 -1.675046
H 3.864402 -1.507147 -1.665659
H 5.499689 1.664155 -1.383995
H 4.940535 3.110522 -0.559303
H 4.362752 2.761934 -2.176276
H -0.207519 2.988205 1.019972
H -1.550895 1.808415 -1.426613
H -2.697816 3.779666 -0.469082
H -2.720808 3.017435 1.116873
H -4.078534 1.963856 -1.397366
H -2.944509 -0.302662 -1.094671
H -0.462993 -1.187215 0.663558
H -1.639693 0.780954 1.432729
H 5.840450 -4.856192 0.021146

H 4.708457 -3.450940 1.699941
H -5.180058 3.982813 -0.435920
H -5.321650 3.166252 1.124722
H -6.209740 2.562253 -0.279189
H -6.093207 -1.145023 1.403518
H -7.312857 -0.825458 0.142413
H -6.802299 0.451850 1.292349
H 2.197240 -5.102229 -2.225658
H 1.065345 -3.713093 -0.534509
H 4.596122 -5.677195 -1.959988
H -0.601910 -1.143683 -3.757806
H -0.963490 -2.883704 -3.546454
H 0.672302 -2.254741 -3.259877
81
2-c6.out delta G = 1.9566 kcal/mol
conformer 13
O 0.216086 -0.299106 -1.784940
C -0.143875 -1.347671 -2.533801
C 2.420820 2.742078 -0.133823
O 0.602353 0.693687 2.530779
C 1.444412 0.360382 1.708244
N 2.663834 -0.130221 2.018439
O 2.755880 3.022020 1.243497
C 3.428798 -0.701445 0.925457
O -0.255751 3.866384 -0.319485
C -0.128304 4.916519 0.630569
O -3.724768 -0.209124 1.676966
C 2.684342 -0.151791 -0.336948
C 3.624635 0.793948 -1.057356
O -2.381575 -1.907112 0.048185
C 3.567613 2.121690 -0.898098
O -5.034794 -0.645441 -1.621263
C 1.157631 1.910218 -0.309880
C 3.559431 -2.234004 1.018665
C 1.339589 0.453450 0.169117
C 4.660165 0.068386 -1.870526
C 4.552126 3.115654 -1.446444
C -0.120369 2.549465 0.235190
C -1.347740 1.734116 -0.178968
C -2.623058 2.279070 0.461047
C -3.873966 1.527494 0.008097
C -3.726891 0.014909 0.268147
C -4.895992 -0.730872 -0.416200
C -2.423852 -0.531005 -0.338898
C 0.119875 -0.354135 -0.325902
C -1.200280 0.242296 0.159132
C 0.705811 -4.392152 -0.270828
C 1.900594 -3.682941 -0.203909
C 2.267453 -3.002027 0.957923
C -5.137262 2.099388 0.651976
C -5.826175 -1.535698 0.443197
C 0.203348 -3.746945 1.989478
C 1.403912 -3.050341 2.057398
C -0.149600 -4.421367 0.823835
O -0.687535 -2.342706 -2.097131
C 0.229465 -1.141523 -3.970887
H 2.219561 3.724866 -0.558933
H 2.902095 -0.299493 2.985527
H 3.170353 2.236556 1.622314
H 4.441199 -0.297118 0.955327
H 0.842921 4.888682 1.133076
H -0.918760 4.864704 1.387023
H -0.222926 5.853712 0.083178
H -3.330453 -1.080227 1.823767
H 2.469717 -0.986239 -1.002521
H -1.742335 -2.349020 -0.537002
H 1.023370 1.850288 -1.392358
H 4.215863 -2.552008 0.206603
H 4.083708 -2.461569 1.950721
H 5.372985 0.733007 -2.350396
H 4.167029 -0.519722 -2.650879
H 5.222957 -0.641487 -1.257026
H 4.094124 3.716838 -2.238621
H 5.455070 2.661047 -1.846636
H 4.848229 3.810913 -0.656079
H -0.075440 2.618016 1.322012
H -1.431778 1.833464 -1.267800
H -2.735985 3.334251 0.207356
H -2.534736 2.214601 1.549677
H -3.960196 1.630523 -1.076863
H -2.510971 -0.449431 -1.424757
H 0.206719 -1.395088 -0.024323
H -1.176091 0.144372 1.244251
H 0.437047 -4.906392 -1.183792
H 2.558911 -3.660552 -1.064317
H -5.111044 1.976008 1.735249
H -6.041224 1.616007 0.275600
H -5.217302 3.165452 0.432719

H -6.632000 -1.941553 -0.164478
H -6.224687 -0.921569 1.251862
H -5.271549 -2.350635 0.914919
H -0.455185 -3.767324 2.848707
H 1.674901 -2.548354 2.976966
H -1.085233 -4.963313 0.770667
H -0.052283 -0.141227 -4.297563
H -0.251079 -1.893622 -4.590085
H 1.313738 -1.228564 -4.068362
81
2-c8.out delta G = 1.9716 kcal/mol conformer 14
O -0.295047 0.127147 -1.988212
C -0.709805 -0.850449 -2.805570
C 2.352708 2.631665 0.180801
O 0.498296 0.369201 2.410691
C 1.172427 0.009392 1.452556
N 2.124837 -0.938285 1.491493
O 2.653534 2.523438 1.586377
C 2.741232 -1.295366 0.227625
O -0.135714 4.083749 0.428151
C -0.289922 4.671049 -0.858659
O -4.068772 0.178684 1.596525
C 2.256167 -0.162157 -0.726958
C 3.406244 0.766634 -1.057595
O -2.914838 -1.441846 -0.257082
C 3.491270 2.018484 -0.594503
O -5.432527 0.287109 -1.707378
C 1.016176 2.004497 -0.196351
C 2.348372 -2.704264 -0.249550
C 1.035782 0.480651 -0.016001
C 4.426199 0.120949 -1.956609
C 4.651785 2.951418 -0.803492
C -0.191119 2.650162 0.496916
C -1.506159 2.077450 -0.041836
C -2.715742 2.664068 0.682909
C -4.038232 2.110563 0.153433
C -4.055915 0.570163 0.225740
C -5.304326 0.046938 -0.522384
C -2.823371 -0.027981 -0.470125
C -0.291468 -0.103318 -0.546966
C -1.518961 0.546728 0.085928
C 2.165956 -5.216052 2.603516
C 1.798560 -4.267110 1.653450
C 2.731294 -3.771636 0.740259
C -5.234198 2.732137 0.875554
C -6.319835 -0.739749 0.252662
C 4.414456 -5.195888 1.747075
C 4.043770 -4.247534 0.800268
C 3.475546 -5.682981 2.653253
O -1.171997 -1.905875 -2.413704
C -0.522236 -0.478068 -4.243845
H 2.306315 3.693129 -0.076679
H 2.317912 -1.438441 2.346609
H 2.032873 3.084987 2.066978
H 3.824680 -1.262098 0.342844
H 0.435011 4.287527 -1.582023
H -0.119701 5.738488 -0.729087
H -1.296739 4.522932 -1.258943
H -3.794300 -0.748017 1.631724
H 1.936258 -0.619770 -1.662962
H -2.343307 -1.874813 -0.913585
H 0.910324 2.171275 -1.271492
H 1.273221 -2.736625 -0.434704
H 2.847215 -2.877241 -1.205875
H 3.958657 -0.134662 -2.912929
H 4.803352 -0.815360 -1.536750
H 5.280428 0.759142 -2.163858
H 4.899642 3.441929 0.141261
H 4.389302 3.743981 -1.511950
H 5.548510 2.456632 -1.167826
H -0.143849 2.443020 1.564475
H -1.579994 2.322978 -1.106624
H -2.721121 3.749691 0.575973
H -2.631974 2.452165 1.752678
H -4.109566 2.354363 -0.910065
H -2.914022 0.196175 -1.535521
H -0.313742 -1.178039 -0.384187
H -1.475849 0.294386 1.145818
H 1.428561 -5.591739 3.302023
H 0.776298 -3.909206 1.617469
H -5.225053 2.473580 1.934945
H -6.184226 2.400821 0.451505
H -5.196235 3.819364 0.790063
H -6.629187 -0.190985 1.142953
H -5.866888 -1.671359 0.601367
H -7.176051 -0.962478 -0.380171
H 5.434795 -5.557685 1.776334

H 4.778794 -3.875142 0.095832
H 3.762610 -6.422982 3.389652
H -0.990936 0.486164 -4.441644
H -0.951771 -1.243463 -4.883410
H 0.543854 -0.375354 -4.453694
81
2-c11.out delta G = 1.9792 kcal/mol conformer 15
O 0.204360 -0.333568 -1.701156
C -0.161205 -1.412438 -2.401899
C 2.401419 2.759687 -0.162808
O 0.557920 0.880060 2.571736
C 1.383109 0.446217 1.774777
N 2.497112 -0.218252 2.118966
O 2.674053 2.945655 1.243721
C 3.358570 -0.672282 1.049420
O -0.272351 3.900628 -0.412995
C -0.123434 4.985479 0.493147
O -3.767291 -0.069665 1.728038
C 2.649689 -0.132616 -0.235951
C 3.607360 0.767552 -0.986897
O -2.404878 -1.850620 0.199512
C 3.545080 2.101117 -0.904904
O -5.048955 -0.674501 -1.554943
C 1.135604 1.939614 -0.315094
C 3.597781 -2.194078 1.087420
C 1.310508 0.509087 0.231204
C 4.651087 -0.001829 -1.748809
C 4.517145 3.065236 -1.526609
C -0.144510 2.605935 0.193483
C -1.369332 1.776760 -0.201292
C -2.650463 2.351244 0.399578
C -3.897264 1.578801 -0.029612
C -3.756927 0.081112 0.309351
C -4.922583 -0.695096 -0.345636
C -2.450054 -0.495366 -0.258707
C 0.093768 -0.318561 -0.242101
C -1.229082 0.301782 0.205123
C 1.048295 -4.497203 -0.545166
C 2.175883 -3.711008 -0.336082
C 2.374443 -3.045755 0.875921
C -5.167258 2.185982 0.567487
C -5.866436 -1.446477 0.546526
C 0.272661 -3.962388 1.665629
C 1.408443 -3.187979 1.876728
C 0.089533 -4.621144 0.453590
O -0.732071 -2.373795 -1.926476
C 0.246077 -1.291080 -3.839421
H 2.220870 3.739318 -0.613712
H 2.733372 -0.342056 3.092333
H 3.523927 3.392960 1.328466
H 4.334063 -0.191653 1.151417
H 0.852057 4.965572 0.988081
H -0.906960 4.973038 1.258618
H -0.212405 5.901810 -0.089512
H -3.377652 -0.933311 1.923165
H 2.423728 -0.982283 -0.878464
H -1.774125 -2.319201 -0.374986
H 1.007225 1.837827 -1.394790
H 4.342635 -2.428407 0.325198
H 4.054496 -2.430197 2.052520
H 5.324712 0.633676 -2.316825
H 4.165729 -0.691405 -2.446091
H 5.259616 -0.615621 -1.077667
H 5.382568 2.583310 -1.973971
H 4.887692 3.771379 -0.776105
H 4.023619 3.665123 -2.297816
H -0.109636 2.713641 1.276742
H -1.440493 1.828563 -1.294486
H -2.760762 3.393270 0.095102
H -2.572812 2.338303 1.490795
H -3.969917 1.625971 -1.119457
H -2.530983 -0.472146 -1.347563
H 0.175884 -1.345204 0.108394
H -1.211081 0.256187 1.294088
H 0.911283 -4.999345 -1.494041
H 2.916873 -3.616884 -1.121383
H -5.157814 2.114832 1.655723
H -6.068243 1.689449 0.201238
H -5.238430 3.240656 0.296337
H -6.668367 -1.880677 -0.046538
H -6.269534 -0.786057 1.315506
H -5.321898 -2.236984 1.068600
H -0.467967 -4.053951 2.450241
H 1.546026 -2.693059 2.828934
H -0.795184 -5.222896 0.288465
H -0.269712 -2.038805 -4.435340
H 1.324055 -1.452889 -3.910766

H 0.035537 -0.290460 -4.214277
81
2-c14.out delta G = 2.1028 kcal/mol
conformer 16
O -0.537305 -0.462364 -1.320251
C -0.614368 -1.725355 -1.782022
C 2.324063 2.413701 -0.087696
O 0.295092 1.219527 2.740142
C 0.955366 0.502998 1.994877
N 1.852186 -0.409802 2.402504
O 2.627056 2.770328 1.276140
C 2.487542 -1.213166 1.376133
O -0.055934 4.017092 -0.453027
C -0.133988 4.107952 -1.870700
O -4.187018 1.094276 1.908861
C 2.044015 -0.506048 0.054832
C 3.235461 0.171315 -0.594079
O -3.167351 -1.226270 0.785029
C 3.408420 1.497814 -0.593487
O -6.365406 0.095371 0.969051
C 0.948239 1.781286 -0.253703
C 2.080142 -2.695571 1.480024
C 0.860082 0.420034 0.453484
C 4.202790 -0.802914 -1.210583
C 4.624713 2.221253 -1.102209
C -0.214265 2.709907 0.122167
C -1.560006 2.065689 -0.225674
C -2.737693 2.956433 0.161232
C -4.078685 2.320271 -0.205609
C -4.201817 0.942523 0.492882
C -5.520626 0.256093 0.106358
C -3.017624 0.027744 0.122089
C -0.506201 -0.222265 0.115299
C -1.680272 0.693018 0.455985
C 3.198428 -4.811464 -1.482707
C 2.399966 -4.057701 -0.626214
C 2.908712 -3.573943 0.579197
C -5.252325 3.240743 0.122362
C -5.726261 -0.205284 -1.304335
C 5.032519 -4.622575 0.061832
C 4.232539 -3.871139 0.914527
C 4.518069 -5.092199 -1.144530
O -0.651651 -2.704363 -1.071252
C -0.612488 -1.744824 -3.282261
H 2.347644 3.325737 -0.690071
H 2.027171 -0.552659 3.386434
H 2.034005 3.486483 1.534742
H 3.569815 -1.145081 1.491023
H -1.130756 3.856871 -2.243689
H 0.598463 3.467816 -2.370159
H 0.081691 5.144930 -2.122421
H -5.085280 0.865361 2.205714
H 1.697910 -1.273774 -0.634905
H -3.306493 -1.022068 1.721451
H 0.859738 1.559816 -1.320136
H 2.211718 -3.006190 2.519650
H 1.020654 -2.795197 1.240238
H 3.670770 -1.456313 -1.907763
H 4.652502 -1.461550 -0.464136
H 5.004900 -0.312238 -1.754595
H 5.479866 1.571125 -1.269535
H 4.921668 2.984096 -0.377668
H 4.405293 2.744570 -2.038802
H -0.193572 2.898062 1.194226
H -1.591184 1.898329 -1.307188
H -2.654341 3.921301 -0.340421
H -2.711717 3.152464 1.236852
H -4.070805 2.134855 -1.283905
H -3.056219 -0.189947 -0.944995
H -0.618766 -1.179650 0.616042
H -1.650532 0.846911 1.535324
H 2.787618 -5.178583 -2.415295
H 1.373195 -3.843140 -0.892654
H -5.116151 4.207000 -0.365555
H -5.320001 3.411133 1.197921
H -6.205182 2.829481 -0.216915
H -5.222221 -1.168284 -1.425418
H -5.297036 0.481958 -2.031936
H -6.788162 -0.342615 -1.495823
H 6.056380 -4.842862 0.337831
H 4.640380 -3.508006 1.851235
H 5.139897 -5.677041 -1.810706
H -1.298729 -0.996862 -3.677528
H -0.881728 -2.735089 -3.638953
H 0.390716 -1.495226 -3.634739
81
2-c1.out delta G = 2.6424 kcal/mol
conformer 17

O 0.211876 -0.256136 -1.669633
C -0.168110 -1.308067 -2.404760
C 2.415010 2.798308 0.001005
O 0.608314 0.846017 2.641337
C 1.414194 0.413408 1.823396
N 2.512645 -0.290367 2.137200
O 2.745612 2.966385 1.395412
C 3.359974 -0.723777 1.047457
O -0.222344 3.997660 -0.052979
C -0.383254 4.292031 -1.435487
O -3.731283 -0.076393 1.795742
C 2.670528 -0.097903 -0.210014
C 3.651067 0.848846 -0.871463
O -2.377044 -1.836724 0.221358
C 3.592336 2.174901 -0.706907
O -5.042249 -0.650723 -1.481348
C 1.158431 1.969741 -0.219963
C 3.561233 -2.250162 1.012915
C 1.331300 0.524604 0.282203
C 4.721713 0.127467 -1.642841
C 4.608740 3.167478 -1.196608
C -0.129487 2.610221 0.309891
C -1.356554 1.799656 -0.122842
C -2.639335 2.363233 0.485459
C -3.884815 1.588776 0.056432
C -3.734828 0.088547 0.378732
C -4.901673 -0.688209 -0.274077
C -2.430918 -0.473402 -0.208666
C 0.111701 -0.288590 -0.210410
C -1.208805 0.318424 0.261974
C 0.223206 -4.001271 1.565437
C 1.364664 -3.238730 1.787946
C 2.321227 -3.071348 0.781615
C -5.152868 2.183590 0.669789
C -5.827185 -1.461570 0.618342
C 0.971710 -4.469854 -0.669665
C 2.105379 -3.695698 -0.448777
C 0.024268 -4.622038 0.335902
O -0.738995 -2.282281 -1.957668
C 0.223370 -1.135987 -3.841492
H 2.246306 3.788018 -0.430507
H 2.753271 -0.447865 3.104799
H 2.073298 3.533671 1.792858
H 4.348823 -0.277954 1.172398
H 0.405376 3.849857 -2.050609
H -0.327243 5.375650 -1.525277
H -1.352903 3.958924 -1.815458
H -3.340029 -0.941503 1.980235
H 2.450232 -0.897998 -0.915107
H -1.754546 -2.292680 -0.371517
H 1.061510 1.889072 -1.304188
H 4.290668 -2.463703 0.229748
H 4.027418 -2.541352 1.958417
H 5.292795 -0.546879 -0.997804
H 5.426111 0.797069 -2.128535
H 4.261732 -0.496818 -2.414950
H 4.886652 3.838073 -0.378468
H 4.188588 3.795871 -1.988645
H 5.519228 2.709615 -1.575131
H -0.103298 2.634476 1.397114
H -1.430737 1.848484 -1.214450
H -2.757939 3.408596 0.196075
H -2.555428 2.343454 1.575959
H -3.966117 1.647026 -1.032250
H -2.519786 -0.427969 -1.296119
H 0.195712 -1.325214 0.108404
H -1.182249 0.254220 1.349807
H -0.509315 -4.114075 2.354854
H 1.515468 -2.776220 2.754325
H -6.054213 1.687955 0.303333
H -5.230221 3.241272 0.412465
H -5.135302 2.098610 1.756921
H -6.628892 -1.900118 0.028224
H -6.231755 -0.814267 1.397691
H -5.267451 -2.249933 1.127455
H 0.821487 -4.941011 -1.632263
H 2.838160 -3.580371 -1.238945
H -0.864170 -5.216019 0.162573
H -0.294394 -1.866670 -4.456434
H 1.301447 -1.287546 -3.930283
H 0.002021 -0.124880 -4.180531

81

2-c24.out delta G = 3.0936 kcal/mol

conformer 18

O -0.342116 0.093406 -2.045625
C -0.437538 -0.964626 -2.878402
C 2.332860 2.732855 0.024068
O 0.844805 0.686148 2.314868

C 1.307718 0.113941 1.326183
N 2.092598 -0.969145 1.352589
O 2.436247 2.996242 1.440782
C 2.685251 -1.357396 0.078863
O -0.238821 4.066921 -0.294208
C -0.124391 5.062022 0.716966
O -3.967495 0.178996 1.702633
C 2.319310 -0.141497 -0.833619
C 3.524186 0.765143 -0.959952
O -2.695683 -1.574904 0.019510
C 3.556593 2.023682 -0.507901
O -5.391399 -0.167996 -1.505576
C 1.041038 2.033117 -0.389698
C 2.203134 -2.725380 -0.429160
C 1.098181 0.513910 -0.146743
C 4.719595 0.109117 -1.615991
C 4.789293 2.884685 -0.539084
C -0.209307 2.715851 0.185357
C -1.503024 2.023759 -0.242951
C -2.724645 2.651592 0.422621
C -4.029715 1.976151 -0.001802
C -3.976668 0.461105 0.296475
C -5.219924 -0.219753 -0.303779
C -2.732610 -0.193581 -0.341935
C -0.229855 -0.124426 -0.614100
C -1.439484 0.520536 0.054129
C 1.645988 -5.149767 2.447482
C 1.419881 -4.188114 1.467184
C 2.436072 -3.812840 0.585871
C -5.249053 2.651331 0.626205
C -6.176043 -0.913505 0.618741
C 3.915803 -5.387455 1.684572
C 3.686016 -4.424715 0.707303
C 2.895632 -5.751761 2.559587
O -0.394806 -2.116835 -2.514586
C -0.607337 -0.504729 -4.296654
H 2.316350 3.727541 -0.426404
H 2.342389 -1.409934 2.225738
H 2.022431 2.269791 1.930230
H 3.764467 -1.415756 0.214160
H -0.965829 5.016484 1.416603
H -0.138161 6.027018 0.211673
H 0.809268 4.954225 1.274473
H -3.412932 0.825803 2.154660
H 2.057168 -0.506475 -1.827255
H -2.817001 -1.608713 0.979760
H 0.978525 2.152873 -1.474650
H 1.149348 -2.678626 -0.696359
H 2.753772 -2.940110 -1.347862
H 5.454852 -0.237717 -0.884601
H 5.231829 0.795156 -2.289735
H 4.411759 -0.759240 -2.200654
H 5.704191 2.316069 -0.690511
H 4.880995 3.428357 0.403937
H 4.718888 3.635440 -1.333789
H -0.156657 2.719105 1.274804
H -1.598711 2.154525 -1.325326
H -2.778510 3.709802 0.164884
H -2.609057 2.615121 1.514127
H -4.110722 2.055437 -1.089035
H -2.858720 -0.165074 -1.423547
H -0.236220 -1.193596 -0.432566
H -1.302398 0.384367 1.131154
H 0.845949 -5.430377 3.121369
H 0.445062 -3.722687 1.382406
H -6.183528 2.224472 0.257986
H -5.251887 3.714424 0.381261
H -5.239702 2.557260 1.713361
H -7.014662 -1.308034 0.049565
H -6.524557 -0.222737 1.388151
H -5.659823 -1.721386 1.141485
H 4.889449 -5.855337 1.761768
H 4.483525 -4.146854 0.027575
H 3.072577 -6.501758 3.320212
H 0.176547 0.206960 -4.556282
H -1.565429 0.008219 -4.397267
H -0.577669 -1.358673 -4.967422
81
2-c19.out delta G = 3.1608 kcal/mol
conformer 19
O 0.213495 -0.247687 -1.659019
C -0.159080 -1.302011 -2.394003
C 2.418690 2.790410 0.014979
O 0.568749 0.818615 2.653256
C 1.392211 0.410589 1.841486
N 2.498340 -0.279480 2.161457
O 2.667830 2.892599 1.433352
C 3.361248 -0.695210 1.077775

O -0.229409 4.007272 -0.070236
C -0.381839 4.279809 -1.457282
O -3.742606 -0.065639 1.790570
C 2.663780 -0.093599 -0.186377
C 3.631746 0.838399 -0.885705
O -2.384844 -1.824600 0.216913
C 3.570551 2.167259 -0.744922
O -5.045739 -0.629429 -1.491301
C 1.155434 1.979180 -0.201352
C 3.589240 -2.218812 1.048930
C 1.322825 0.531779 0.300269
C 4.680165 0.102094 -1.672569
C 4.548363 3.158478 -1.312121
C -0.132144 2.626931 0.318526
C -1.357814 1.811477 -0.114349
C -2.642420 2.375610 0.489417
C -3.887753 1.603755 0.054729
C -3.741230 0.102489 0.373868
C -4.907045 -0.671368 -0.283921
C -2.435953 -0.459728 -0.210361
C 0.105864 -0.279014 -0.200081
C -1.215229 0.329419 0.267141
C 0.266557 -4.004757 1.581194
C 1.403371 -3.238019 1.813370
C 2.360059 -3.054119 0.810181
C -5.156869 2.198885 0.665687
C -5.833747 -1.448467 0.604090
C 1.020513 -4.444999 -0.657885
C 2.149445 -3.666861 -0.427078
C 0.072515 -4.613006 0.344702
O -0.730681 -2.276745 -1.948436
C 0.242596 -1.134042 -3.828399
H 2.249631 3.797274 -0.375530
H 2.730842 -0.440692 3.130278
H 3.527159 3.311597 1.557203
H 4.340520 -0.228443 1.204196
H -0.326122 5.362159 -1.563287
H -1.348619 3.941215 -1.840437
H 0.411357 3.830343 -2.061531
H -3.351131 -0.930762 1.974478
H 2.439425 -0.909790 -0.871400
H -1.759645 -2.279179 -0.374371
H 1.061072 1.895421 -1.284955
H 4.326968 -2.424199 0.271379
H 4.052057 -2.501026 1.998713
H 4.200385 -0.533138 -2.423427
H 5.263805 -0.564006 -1.029945
H 5.376202 0.760737 -2.184677
H 4.064840 3.785537 -2.067900
H 5.424155 2.698747 -1.762887
H 4.900664 3.836854 -0.528138
H -0.101763 2.669986 1.403833
H -1.429895 1.860861 -1.206186
H -2.758056 3.421440 0.200495
H -2.562700 2.354681 1.580167
H -3.965768 1.664845 -1.034072
H -2.520704 -0.412118 -1.298147
H 0.187507 -1.315860 0.119250
H -1.192771 0.262452 1.354555
H -0.466262 -4.130394 2.368446
H 1.550000 -2.784252 2.784535
H -5.231653 3.257401 0.411026
H -5.142772 2.110955 1.752641
H -6.058027 1.705910 0.295096
H -6.634960 -1.884373 0.011339
H -6.238781 -0.804578 1.385951
H -5.274390 -2.239085 1.110126
H 0.874039 -4.906686 -1.625626
H 2.882365 -3.538175 -1.215077
H -0.812632 -5.209578 0.163646
H 1.318673 -1.303139 -3.910625
H 0.039445 -0.119443 -4.168049
H -0.282255 -1.856579 -4.447019
81
2-c26.out delta G = 3.2480 kcal/mol
conformer 20
O -0.340769 -0.524552 -1.273128
C -0.772208 -1.714713 -1.706783
C 2.354431 2.494789 -0.113288
O 0.343699 1.259244 2.761948
C 1.049231 0.580870 2.025575
N 2.005038 -0.269875 2.441901
O 2.546893 2.881163 1.263707
C 2.618673 -1.105702 1.427649
O -0.088926 4.011835 -0.517799
C -0.202376 4.052196 -1.934486
O -4.178163 0.923277 1.931576
C 2.163807 -0.426664 0.098804

C 3.333337 0.265122 -0.572537
O -3.057777 -1.290113 0.816260
C 3.457514 1.596042 -0.625516
O -5.479371 -0.132834 -1.227507
C 0.999551 1.823998 -0.252415
C 2.184068 -2.578200 1.576837
C 0.958124 0.474189 0.483484
C 4.328913 -0.700239 -1.158978
C 4.629757 2.338318 -1.208110
C -0.189933 2.722795 0.108351
C -1.518894 2.025301 -0.205782
C -2.715310 2.871926 0.223493
C -4.050084 2.205532 -0.107924
C -4.127496 0.794783 0.512239
C -5.383544 0.075804 -0.033469
C -2.907383 -0.049744 0.115128
C -0.380452 -0.222628 0.156401
C -1.592975 0.648234 0.469552
C 2.735128 -4.673137 -1.552015
C 2.116684 -3.912528 -0.563980
C 2.829118 -3.472003 0.552100
C -5.233127 3.080431 0.307053
C -6.448785 -0.332278 0.941145
C 4.796527 -4.589741 -0.315161
C 4.174654 -3.828917 0.668029
C 4.079838 -5.008731 -1.433511
O -1.298339 -2.544988 -0.987917
C -0.496810 -1.902955 -3.166329
H 2.342680 3.402662 -0.722716
H 2.169170 -0.408117 3.428400
H 3.426618 3.266698 1.345840
H 3.703134 -1.055842 1.525801
H -1.205109 3.780148 -2.276169
H 0.524969 3.403291 -2.430941
H -0.003317 5.081979 -2.227042
H -3.932741 0.065593 2.305120
H 1.833029 -1.206852 -0.584265
H -2.495345 -1.946895 0.372231
H 0.915046 1.574593 -1.312546
H 2.453722 -2.906809 2.583693
H 1.097094 -2.639534 1.501093
H 5.075606 -0.210447 -1.777641
H 3.810035 -1.439325 -1.775388
H 4.852593 -1.266336 -0.385213
H 5.488740 1.704565 -1.412119
H 4.962186 3.124864 -0.523257
H 4.345259 2.840530 -2.138412
H -0.152866 2.949612 1.170864
H -1.573135 1.862811 -1.287576
H -2.676038 3.845180 -0.267908
H -2.655892 3.056800 1.300003
H -4.095342 2.051922 -1.189678
H -2.968532 -0.219740 -0.962276
H -0.449498 -1.166246 0.692290
H -1.580338 0.797591 1.549422
H 2.165339 -5.003074 -2.411825
H 1.065878 -3.667762 -0.655407
H -5.153565 4.061316 -0.164435
H -5.251177 3.223082 1.388041
H -6.187794 2.645564 0.004350
H -6.747479 0.517249 1.556393
H -6.047550 -1.086028 1.623299
H -7.303039 -0.739899 0.405063
H 5.841102 -4.855477 -0.209902
H 4.741311 -3.504104 1.533180
H 4.564507 -5.598928 -2.201005
H -1.053710 -2.757062 -3.540620
H 0.572488 -2.082302 -3.299026
H -0.753897 -1.002901 -3.723103
81
2-c16.out delta G = 3.5097 kcal/mol
conformer 21
O -0.493496 -0.542950 -1.365690
C -0.699694 -1.804291 -1.793725
C 2.311629 2.470232 -0.187972
O 0.609637 1.431614 2.586027
C 1.053601 0.526855 1.876760
N 1.745243 -0.533289 2.311553
O 2.405056 3.184216 1.064252
C 2.373669 -1.350051 1.281107
O -0.141142 3.765258 -1.084341
C 0.030782 5.040490 -0.476003
O -4.199657 1.076536 1.941711
C 2.105934 -0.514977 -0.014251
C 3.372105 0.215895 -0.408955
O -3.026860 -1.240967 1.053536
C 3.489124 1.548736 -0.404944
O -5.577018 -0.296988 -1.001495

C 0.982255 1.752303 -0.402221
C 1.859990 -2.800265 1.263473
C 0.918163 0.409819 0.347118
C 4.520105 -0.697903 -0.776737
C 4.778090 2.272582 -0.682145
C -0.228389 2.667247 -0.166664
C -1.557092 1.948367 -0.400799
C -2.746520 2.844866 -0.067456
C -4.086511 2.142519 -0.290975
C -4.156110 0.836036 0.528398
C -5.432651 0.063137 0.149988
C -2.951209 -0.081050 0.224716
C -0.442218 -0.266520 0.059650
C -1.616711 0.644921 0.404551
C 3.149736 -4.799249 -1.714298
C 2.307397 -4.063986 -0.883928
C 2.726875 -3.668261 0.386448
C -5.266897 3.072005 -0.007279
C -6.449937 -0.213229 1.215010
C 4.847859 -4.773376 -0.011151
C 4.004776 -4.038956 0.813984
C 4.424074 -5.152026 -1.283064
O -0.835574 -2.753117 -1.055457
C -0.701387 -1.863277 -3.292496
H 2.376134 3.255741 -0.943908
H 1.952861 -0.647444 3.293418
H 1.917953 2.696486 1.744837
H 3.443110 -1.383446 1.483686
H 0.108851 5.766598 -1.284295
H 0.938901 5.071095 0.131281
H -0.827807 5.300936 0.152192
H -3.598070 1.798063 2.160307
H 1.836888 -1.191127 -0.825143
H -3.174522 -0.921107 1.955670
H 0.959832 1.493899 -1.464244
H 1.883122 -3.173533 2.290420
H 0.825044 -2.833119 0.925441
H 4.150717 -1.674611 -1.090348
H 5.198480 -0.870107 0.063626
H 5.111776 -0.284764 -1.592528
H 4.771698 2.709105 -1.686934
H 5.654584 1.634360 -0.595174
H 4.891357 3.100227 0.021833
H -0.210822 3.047395 0.855720
H -1.607988 1.696078 -1.464406
H -2.714059 3.741771 -0.686803
H -2.665019 3.195895 0.969852
H -4.133505 1.831681 -1.337947
H -3.051666 -0.432122 -0.801593
H -0.533515 -1.207882 0.591674
H -1.513647 0.888715 1.466272
H 2.807595 -5.096949 -2.697850
H 1.313293 -3.801037 -1.220109
H -6.221864 2.598613 -0.242090
H -5.185144 3.972654 -0.617437
H -5.289473 3.376997 1.040332
H -7.303232 -0.730387 0.782081
H -6.764321 0.718347 1.688076
H -5.998897 -0.821303 2.002028
H 5.835046 -5.051206 0.336932
H 4.343723 -3.745488 1.801124
H 5.080462 -5.722085 -1.928480
H -1.044707 -2.839731 -3.622322
H 0.315722 -1.696081 -3.653340
H -1.333025 -1.077469 -3.705346

81

2-c17.out delta G = 3.5279 kcal/mol

conformer 22

O -0.538996 -0.539648 -1.353218
C -0.610343 -1.813648 -1.786224
C 2.308068 2.379508 -0.156356
O 0.305288 1.270823 2.651052
C 0.954098 0.517058 1.930574
N 1.836668 -0.396014 2.362484
O 2.634639 2.684180 1.216798
C 2.492706 -1.205367 1.351955
O -0.137146 3.838678 -0.854117
C 0.066435 5.060966 -0.161336
O -4.322226 0.975284 1.863303
C 2.046549 -0.528997 0.014773
C 3.227881 0.143113 -0.659387
O -3.147391 -1.277380 0.813346
C 3.387439 1.470988 -0.690417
O -5.595766 -0.115152 -1.239445
C 0.933734 1.745039 -0.331757
C 2.107345 -2.691638 1.479600
C 0.854963 0.395056 0.394211
C 4.194889 -0.836886 -1.266258

C 4.579198 2.200738 -1.244346
C -0.241482 2.677802 -0.016882
C -1.580013 1.995874 -0.309132
C -2.762607 2.887260 0.060528
C -4.106000 2.231290 -0.261867
C -4.222725 0.859032 0.437215
C -5.493521 0.144175 -0.056748
C -3.018068 -0.046638 0.100863
C -0.503722 -0.269492 0.075238
C -1.685041 0.640598 0.403980
C 3.244043 -4.843519 -1.450016
C 2.438584 -4.085478 -0.603959
C 2.944977 -3.573914 0.590952
C -5.279856 3.154170 0.065815
C -6.559518 -0.199048 0.939248
C 5.080151 -4.605070 0.085178
C 4.273232 -3.849137 0.927351
C 4.568233 -5.101372 -1.111530
O -0.635819 -2.776192 -1.052774
C -0.618183 -1.866826 -3.285410
H 2.323856 3.307438 -0.735039
H 2.025671 -0.503130 3.348380
H 1.933264 3.229564 1.593261
H 3.572983 -1.116531 1.472229
H 0.066016 5.853593 -0.908164
H 1.028653 5.078424 0.363450
H -0.732390 5.251700 0.562199
H -3.701906 1.646084 2.172655
H 1.704622 -1.315138 -0.655895
H -3.322030 -1.041946 1.736279
H 0.854508 1.526716 -1.400118
H 2.244931 -2.984620 2.523571
H 1.048863 -2.809828 1.243769
H 3.662624 -1.505037 -1.949009
H 4.651203 -1.479712 -0.510008
H 4.992604 -0.352564 -1.822220
H 4.313910 2.737348 -2.161213
H 5.424377 1.553231 -1.463416
H 4.912674 2.954107 -0.525347
H -0.223433 2.980296 1.032670
H -1.612188 1.817926 -1.389275
H -2.695621 3.830189 -0.483063
H -2.709358 3.150784 1.124998
H -4.123572 2.013194 -1.332911
H -3.075505 -0.300334 -0.956815
H -0.603652 -1.216405 0.598056
H -1.633743 0.815842 1.481274
H 2.835141 -5.231726 -2.374856
H 1.408119 -3.889549 -0.870698
H -6.234571 2.719573 -0.235591
H -5.166080 4.101868 -0.462490
H -5.328569 3.368904 1.134594
H -7.405316 -0.654969 0.429569
H -6.872207 0.695586 1.479776
H -6.154361 -0.884662 1.686542
H 6.107525 -4.807667 0.361637
H 4.679168 -3.465154 1.856517
H 5.195506 -5.689346 -1.769798
H 0.380580 -1.617255 -3.650306
H -1.313109 -1.133277 -3.692273
H -0.882104 -2.866967 -3.617636
81
2-c22.out delta G = 3.6734 kcal/mol
conformer 23
O -0.407635 0.163141 -1.985820
C -0.379876 -0.859896 -2.865426
C 2.331598 2.588063 0.193739
O 0.449410 0.320191 2.400228
C 1.128865 -0.034115 1.442094
N 2.080865 -0.980533 1.482386
O 2.632524 2.461934 1.598196
C 2.712016 -1.326233 0.222735
O -0.145345 4.060407 0.440061
C -0.273014 4.653610 -0.847156
O -4.154775 0.130948 1.589718
C 2.219593 -0.196348 -0.734017
C 3.370209 0.731456 -1.065986
O -2.909925 -1.463306 -0.276094
C 3.464923 1.977963 -0.590901
O -5.473624 0.281779 -1.677372
C 0.991990 1.972413 -0.190890
C 2.342898 -2.739478 -0.258487
C 0.997823 0.445709 -0.022678
C 4.382824 0.087752 -1.974665
C 4.628944 2.907133 -0.796775
C -0.210165 2.627263 0.502902
C -1.528705 2.067340 -0.040275
C -2.737359 2.679094 0.662869

C -4.060103 2.129035 0.126654
C -4.093280 0.588704 0.231943
C -5.343709 0.055869 -0.490522
C -2.858547 -0.046004 -0.445067
C -0.338216 -0.118209 -0.561447
C -1.551426 0.537358 0.095020
C 2.127969 -5.243571 2.599150
C 1.770376 -4.303911 1.636159
C 2.717512 -3.798431 0.743358
C -5.261923 2.786550 0.805630
C -6.355825 -0.717495 0.299258
C 4.395752 -5.195636 1.796736
C 4.034551 -4.256339 0.837230
C 3.442410 -5.691791 2.682725
O -0.246213 -2.019561 -2.548573
C -0.522256 -0.352470 -4.270536
H 2.290987 3.652928 -0.050325
H 2.271596 -1.482394 2.336977
H 2.013648 3.019050 2.086169
H 3.794028 -1.275320 0.347380
H -1.267463 4.497987 -1.274733
H 0.473976 4.281734 -1.553813
H -0.117238 5.721851 -0.706969
H -3.558280 0.660583 2.131827
H 1.904067 -0.663338 -1.665868
H -3.064657 -1.621053 0.666737
H 0.888932 2.146903 -1.265009
H 1.274560 -2.778826 -0.472827
H 2.865742 -2.909949 -1.202521
H 3.908670 -0.161424 -2.929365
H 4.758672 -0.852257 -1.561739
H 5.238090 0.724130 -2.183483
H 5.520914 2.410995 -1.170693
H 4.884583 3.387178 0.151318
H 4.367127 3.708008 -1.496087
H -0.165878 2.415947 1.570005
H -1.594096 2.303740 -1.106819
H -2.733157 3.762379 0.539251
H -2.656324 2.509335 1.744183
H -4.103589 2.346443 -0.943900
H -2.932950 0.133980 -1.516524
H -0.382612 -1.195331 -0.430928
H -1.482395 0.288602 1.156507
H 1.379313 -5.626699 3.281530
H 0.744446 -3.960583 1.573523
H -5.205859 3.870040 0.690959
H -5.285501 2.562445 1.873417
H -6.206165 2.455970 0.369449
H -7.189504 -0.993953 -0.342229
H -6.703380 -0.125244 1.147194
H -5.888285 -1.610807 0.718470
H 5.420025 -5.543142 1.851801
H 4.780963 -3.876840 0.148665
H 3.721949 -6.424444 3.429329
H -1.431357 0.242660 -4.361513
H -0.551591 -1.188503 -4.963470
H 0.321993 0.296099 -4.510199
81
2-c21.out delta G = 3.6992 kcal/mol
conformer 24
O 0.224076 -0.219334 -1.722042
C -0.131924 -1.241272 -2.509211
C 2.432285 2.761295 0.037357
O 0.617868 0.612446 2.626899
C 1.458863 0.314399 1.790250
N 2.681083 -0.183341 2.078868
O 2.763159 2.979964 1.425342
C 3.440695 -0.718628 0.963832
O -0.217618 3.948545 0.059057
C -0.412199 4.274202 -1.311411
O -3.740151 -0.251643 1.714958
C 2.694596 -0.122509 -0.275774
C 3.633997 0.850163 -0.962174
O -2.375618 -1.889080 0.045538
C 3.577976 2.171064 -0.752780
O -4.999387 -0.558728 -1.617829
C 1.170425 1.935370 -0.178207
C 3.566049 -2.254054 1.001916
C 1.350010 0.464250 0.254952
C 4.666889 0.156432 -1.805834
C 4.560591 3.185552 -1.265211
C -0.108633 2.554329 0.392331
C -1.339167 1.755180 -0.053010
C -2.615517 2.280672 0.600295
C -3.863640 1.547925 0.111834
C -3.721990 0.026172 0.316344
C -4.884986 -0.685435 -0.413814
C -2.412609 -0.500432 -0.293632

C 0.129234 -0.326071 -0.265765
C -1.191035 0.253562 0.239765
C 0.195272 -3.784243 1.893267
C 1.397583 -3.094738 1.994025
C 2.272303 -3.015876 0.905329
C -5.131320 2.098661 0.765472
C -5.841166 -1.507565 0.399495
C 0.719124 -4.363746 -0.379769
C 1.915150 -3.660377 -0.280064
C -0.147589 -4.422804 0.704698
O -0.676543 -2.251239 -2.110476
C 0.248833 -0.983806 -3.935950
H 2.235011 3.764414 -0.338561
H 2.921298 -0.391089 3.038015
H 3.176132 2.178193 1.769959
H 4.454442 -0.318906 1.004634
H -0.347388 5.359047 -1.378749
H -1.394778 3.959904 -1.674217
H 0.355151 3.839169 -1.958167
H -3.357486 -1.132391 1.832683
H 2.478878 -0.931048 -0.972027
H -1.733908 -2.311616 -0.551168
H 1.053948 1.892421 -1.262396
H 4.225956 -2.544300 0.182405
H 4.084380 -2.517686 1.927827
H 4.170234 -0.422058 -2.590902
H 5.251267 -0.557577 -1.217360
H 5.362533 0.841302 -2.282817
H 4.867927 3.842366 -0.446596
H 4.095479 3.824850 -2.022603
H 5.457400 2.747462 -1.696357
H -0.057909 2.558080 1.478337
H -1.432037 1.864016 -1.138981
H -2.727059 3.345473 0.389800
H -2.530895 2.178348 1.686044
H -3.940424 1.691551 -0.969370
H -2.488038 -0.381343 -1.376746
H 0.215420 -1.376830 -0.000096
H -1.169879 0.117357 1.320890
H -0.472356 -3.826981 2.744575
H 1.660440 -2.620746 2.930508
H -5.209426 3.171400 0.580828
H -5.112882 1.939492 1.844154
H -6.032755 1.628658 0.366988
H -6.655621 -1.856996 -0.231328
H -6.223498 -0.928919 1.240949
H -5.311243 -2.364118 0.824005
H 0.458214 -4.849954 -1.310269
H 2.582482 -3.613844 -1.132618
H -1.084520 -4.959297 0.625626
H 1.331416 -1.090886 -4.034083
H -0.009989 0.034927 -4.221831
H -0.244975 -1.699941 -4.586607

**Table S22.** Geometry data of conformers of compound **3**.

81
3-c10-gfn0.out delta G = 0.0000 kcal/mol conformer 1
O -0.512089 -0.359432 -1.927880
C -0.205907 -1.482557 -2.610421
C 0.995851 3.346052 -0.314783
O 0.469233 1.044643 2.268001
C 1.115126 0.698204 1.274485
N 2.301945 0.086856 1.317450
O 0.411033 4.541510 -0.854008
C 2.928247 -0.193420 0.033356
O -1.373742 2.770921 1.371191
O -3.841603 -0.939433 1.935365
C 2.036307 0.627001 -0.950509
C 2.761842 1.901895 -1.322453
C -0.551858 -1.326640 -4.062391
O -2.043815 -2.479189 0.476296
C 2.339793 3.121982 -0.972848
O -5.425062 -2.815530 1.164296
C 0.044460 2.183447 -0.578745
C 3.024730 -1.694251 -0.278746
C 0.694449 0.838866 -0.201888
C 4.057081 1.671624 -2.070204
C 3.148008 4.369893 -1.205284
C -1.365888 2.421461 -0.011074
C -2.289177 1.233639 -0.324186
C -3.690273 1.437451 0.243784
C -4.615280 0.256922 -0.055529
C -3.992140 -1.037911 0.522761
C -4.887565 -2.250969 0.228236
C -2.590866 -1.280106 -0.070660
C -0.306907 -0.310966 -0.489797

C -1.669513 -0.080778 0.163955
O 0.274771 -2.470459 -2.105343
C 3.903637 -3.734513 2.809979
C 3.172478 -3.094530 1.813077
C 3.817416 -2.434613 0.765379
C -6.028442 0.500886 0.471303
C -5.063651 -2.712231 -1.186418
C 5.948594 -3.068072 1.731115
C 5.214457 -2.428795 0.738042
C 5.294456 -3.722787 2.771840
H 1.128709 3.483265 0.764267
H 2.731589 -0.154150 2.198863
C 0.081631 5.532918 0.111829
H 3.939837 0.209492 0.053141
H -0.787458 2.144668 1.844581
H -4.478288 -1.573144 2.310293
H 1.881395 0.044149 -1.857629
H -0.391225 6.352568 -0.427923
H -0.203427 -2.191287 -4.620058
H -0.103280 -0.415577 -4.458723
H -1.634297 -1.233910 -4.167380
H -2.120125 -2.396655 1.438182
H -0.082934 2.142422 -1.664266
H 2.027557 -2.120946 -0.371808
H 3.504784 -1.789880 -1.255319
H 4.052273 0.694053 -2.555567
H 4.928771 1.698895 -1.410057
H 4.213662 2.421601 -2.844671
H 2.748412 4.955609 -2.035971
H 3.105978 5.013014 -0.322671
H -1.766946 3.294713 -0.527306
H -2.369911 1.179418 -1.414601
H -4.125068 2.345290 -0.183156
H -3.631957 1.587160 1.322285
H -4.662861 0.133479 -1.141664
H -2.689209 -1.456783 -1.141325
H 0.099665 -1.267362 -0.177020
H -1.505710 -0.013222 1.241806
H 0.978032 5.911919 0.615065
H 4.193858 4.151122 -1.408082
H 3.386779 -4.244135 3.613678
H 2.089405 -3.108578 1.845119
H -6.719535 -0.294188 0.183600
H -6.418559 1.438622 0.073001
H -6.026082 0.571094 1.560267
H -4.191489 -3.312072 -1.461504
H -5.123141 -1.881650 -1.888232
H -5.952085 -3.335098 -1.262690
H 7.030854 -3.058335 1.691593
H 5.729802 -1.923239 -0.070790
H 5.865084 -4.222481 3.544494
H -0.610306 5.140040 0.861133
81
3-c14.out delta G = 0.7009 kcal/mol
conformer 2
O -0.054549 -0.826305 -1.223642
C 0.005851 -2.135954 -1.490804
C 1.113303 3.194819 -0.360783
O -0.271277 1.730294 2.514041
C 0.610858 1.137666 1.887126
N 1.712813 0.621294 2.438007
O 0.768503 4.173767 -1.352339
C 2.650183 -0.018060 1.528523
O -1.660318 3.097394 0.688705
O -4.335492 -0.321518 1.624237
C 2.113457 0.433069 0.133046
C 2.986662 1.547195 -0.402196
C 0.396588 -2.389750 -2.913680
O -2.375493 -2.303911 1.000155
C 2.565901 2.809117 -0.546647
O -5.750431 -2.419081 1.130484
C 0.194012 1.990459 -0.535184
C 2.735825 -1.537649 1.760042
C 0.631655 0.827876 0.375213
C 4.403505 1.117947 -0.709119
C 3.479278 3.946591 -0.917384
C -1.300948 2.353636 -0.472475
C -2.177103 1.105946 -0.674313
C -3.665064 1.438027 -0.571419
C -4.563176 0.210024 -0.736782
C -4.162291 -0.853115 0.320180
C -5.029111 -2.115630 0.197190
C -2.690147 -1.239759 0.104763
C -0.309657 -0.378450 0.142865
C -1.785403 -0.019577 0.293945
O -0.206054 -3.003839 -0.663691
C 4.354545 -3.516085 -1.062390
C 3.397013 -2.978412 -0.206627

C 3.771441 -2.182385 0.876182
C -6.041962 0.578238 -0.647413
C -4.952303 -2.943968 -1.050005
C 6.091302 -2.487978 0.246221
C 5.131767 -1.952030 1.096714
C 5.705079 -3.267516 -0.841656
H 0.966490 3.639110 0.630085
H 1.869063 0.679527 3.434229
C 0.258280 5.396599 -0.834441
H 3.639940 0.402226 1.699333
H -1.281741 2.640131 1.468037
H -4.963259 -0.918488 2.065937
H 2.175164 -0.405149 -0.558316
H -0.639324 5.228247 -0.234209
H -0.172952 -1.748329 -3.585025
H 0.239272 -3.436062 -3.159032
H 1.453462 -2.143544 -3.036889
H -1.597790 -2.757024 0.634808
H 0.363080 1.633339 -1.554835
H 2.989112 -1.698923 2.811146
H 1.760892 -1.996426 1.594836
H 4.432777 0.057645 -0.963273
H 5.076486 1.255945 0.141973
H 4.818706 1.674376 -1.547517
H 4.527487 3.698669 -0.767716
H 3.338304 4.251942 -1.956457
H -1.492605 3.026440 -1.309469
H -1.977628 0.750081 -1.691201
H -3.923908 2.172504 -1.338970
H -3.867278 1.901063 0.394548
H -4.368685 -0.222322 -1.722510
H -2.599294 -1.591182 -0.927726
H -0.048646 -1.191729 0.814053
H -1.924443 0.319295 1.321666
H 0.012487 6.022174 -1.691743
H 3.252817 4.821795 -0.303302
H 4.043496 -4.129322 -1.899002
H 2.348968 -3.191105 -0.373699
H -6.283534 0.954155 0.347743
H -6.690206 -0.276155 -0.854279
H -6.279868 1.357679 -1.372986
H -5.791193 -3.635318 -1.084275
H -4.022247 -3.517878 -1.027846
H -4.934952 -2.329260 -1.949399
H 7.141160 -2.297181 0.430987
H 5.442675 -1.343389 1.937948
H 6.452056 -3.682803 -1.506330
H 1.006713 5.913042 -0.223326
81
3-c5.out delta G = 0.8195 kcal/mol
conformer 3
O -0.247659 -0.844677 -1.244069
C 0.092812 -2.121704 -1.504671
C 1.114986 3.131330 -0.367959
O -0.246642 1.673582 2.520622
C 0.598910 1.053411 1.868818
N 1.679118 0.469899 2.393816
O 0.775589 4.130429 -1.341703
C 2.607180 -0.139675 1.454189
O -1.642290 3.094881 0.736327
O -4.466055 -0.291639 1.642910
C 2.049150 0.339779 0.075356
C 2.940714 1.434535 -0.469983
C 0.218855 -2.347848 -2.982207
O -2.497020 -2.194823 1.177353
C 2.552242 2.709352 -0.589510
O -5.961372 -2.294485 1.027201
C 0.159994 1.953892 -0.534445
C 2.718282 -1.663816 1.632314
C 0.582713 0.768730 0.354287
C 4.338651 0.969729 -0.809993
C 3.488090 3.827414 -0.963637
C -1.322573 2.358221 -0.441709
C -2.235982 1.138250 -0.642949
C -3.712344 1.505713 -0.530657
C -4.627065 0.296660 -0.728644
C -4.271960 -0.785966 0.321508
C -5.163875 -2.024974 0.146847
C -2.792790 -1.198454 0.199846
C -0.404136 -0.406579 0.133539
C -1.862187 0.009310 0.326220
O 0.293408 -2.954951 -0.650345
C 6.264115 -2.412732 0.484048
C 5.187679 -1.966201 1.240882
C 3.873588 -2.221869 0.839509
C -6.102139 0.688895 -0.674661
C -5.021400 -2.867695 -1.083833
C 4.739540 -3.397907 -1.093776

C 3.663211 -2.949051 -0.332207
C 6.042932 -3.126930 -0.691413
H 1.002348 3.569226 0.630223
H 1.858056 0.510696 3.387103
C 0.309309 5.360157 -0.799667
H 3.594097 0.287958 1.624844
H -1.250494 2.624533 1.501373
H -5.223699 -0.788640 1.998542
H 2.075492 -0.495460 -0.622423
H 1.084976 5.851200 -0.201664
H 0.308438 -3.411163 -3.185842
H 1.112767 -1.833994 -3.342322
H -0.638367 -1.929280 -3.508324
H -2.801365 -1.838817 2.025012
H 0.299157 1.601976 -1.560470
H 2.868497 -1.865846 2.696010
H 1.786918 -2.144751 1.334778
H 4.329540 -0.082510 -1.097615
H 5.025757 1.056934 0.036280
H 4.761642 1.538788 -1.635723
H 4.532133 3.549688 -0.839510
H 3.333873 4.151815 -1.994995
H -1.509577 3.047565 -1.266182
H -2.053479 0.777655 -1.660409
H -3.955416 2.261172 -1.282740
H -3.908580 1.950379 0.445386
H -4.413875 -0.129500 -1.713610
H -2.642332 -1.673230 -0.769279
H -0.164637 -1.243536 0.782570
H -1.959985 0.378531 1.349318
H -0.577714 5.208070 -0.179629
H 3.297905 4.699155 -0.332480
H 7.275590 -2.204383 0.810281
H 5.369462 -1.408776 2.152866
H -6.306383 1.476715 -1.401199
H -6.363811 1.065632 0.315487
H -6.760579 -0.151654 -0.903388
H -5.913880 -3.474073 -1.221186
H -4.164667 -3.533140 -0.944798
H -4.829569 -2.270065 -1.973803
H 4.557390 -3.960091 -2.001453
H 2.651256 -3.170280 -0.644300
H 6.880383 -3.473636 -1.283776
H 0.058924 6.000844 -1.644423

81

3-c10.out delta G = 1.1979 kcal/mol

conformer 4

O -0.260059 -0.739747 -1.225246
C 0.066987 -2.007725 -1.541695
C 1.074400 3.205718 -0.222401
O -0.248077 1.632260 2.636646
C 0.589848 1.031041 1.958552
N 1.661608 0.408804 2.456625
O 0.742718 4.283065 -1.118409
C 2.587170 -0.159370 1.488821
O -1.659689 3.109384 0.910074
O -4.468079 -0.326119 1.698740
C 2.036921 0.398989 0.135981
C 2.928538 1.535023 -0.316829
C 0.198764 -2.169182 -3.027256
O -2.490536 -2.200616 1.151878
C 2.526375 2.809496 -0.378453
O -5.956308 -2.311455 1.015041
C 0.134635 2.020646 -0.414540
C 2.691218 -1.691749 1.587161
C 0.569572 0.809783 0.433861
C 4.354292 1.128975 -0.614610
C 3.482628 3.951946 -0.606177
C -1.349083 2.417432 -0.295480
C -2.258474 1.202123 -0.537107
C -3.736192 1.556615 -0.405129
C -4.644154 0.350259 -0.647430
C -4.277503 -0.769557 0.358953
C -5.164287 -2.005433 0.141715
C -2.796616 -1.168469 0.215793
C -0.413481 -0.360494 0.170279
C -1.872846 0.039280 0.386176
O 0.255551 -2.880115 -0.724731
C 4.734108 -3.235459 -1.235892
C 3.651353 -2.834886 -0.456937
C 3.851056 -2.207093 0.772832
C -6.121531 0.730522 -0.572864
C -5.024780 -2.800248 -1.120732
C 6.244579 -2.404970 0.441400
C 5.161485 -2.004979 1.214765
C 6.033898 -3.016965 -0.792230
H 0.911375 3.651526 0.761917
H 1.843983 0.408237 3.450075

C 0.805219 3.976539 -2.506964
H 3.576544 0.251082 1.684161
H -1.263122 2.610523 1.654261
H -5.223703 -0.837586 2.037764
H 2.072462 -0.389790 -0.613970
H 0.835015 4.928729 -3.034558
H 1.123232 -1.685782 -3.351205
H -0.628479 -1.683523 -3.543072
H 0.239738 -3.224824 -3.280701
H -2.794293 -1.881197 2.014215
H 0.263126 1.678388 -1.444184
H 2.836412 -1.948608 2.639530
H 1.761443 -2.156582 1.260973
H 4.408743 0.069287 -0.864619
H 5.021440 1.289537 0.237106
H 4.757836 1.694429 -1.453780
H 4.493343 3.701289 -0.289562
H 3.526701 4.254225 -1.656221
H -1.549644 3.137711 -1.089677
H -2.078703 0.881173 -1.568412
H -3.986096 2.339266 -1.126575
H -3.931649 1.961806 0.588157
H -4.431707 -0.035260 -1.649107
H -2.646571 -1.603848 -0.771766
H -0.167968 -1.222053 0.783614
H -1.969410 0.366685 1.423685
H -0.074258 3.418357 -2.842461
H 3.155289 4.827951 -0.043812
H 4.559666 -3.719609 -2.188886
H 2.642399 -3.018020 -0.801359
H -6.775655 -0.103295 -0.836016
H -6.332492 1.547619 -1.264269
H -6.382528 1.062752 0.433274
H -4.850953 -2.167660 -1.990094
H -5.911184 -3.412488 -1.270861
H -4.158173 -3.459155 -1.016384
H 7.253075 -2.239527 0.799780
H 5.334890 -1.526077 2.171830
H 6.876691 -3.326173 -1.397593
H 1.700271 3.402059 -2.760383

81

3-c3.out delta G = 1.5311 kcal/mol conformer 5

O -0.418091 -0.425104 -1.910636
C -0.405671 -1.653434 -2.450300
C 0.987978 3.395416 -0.417925
O 0.572656 1.150716 2.227984
C 1.187350 0.780086 1.224683
N 2.369095 0.155892 1.242788
O 0.381533 4.565467 -0.988207
C 2.950590 -0.154607 -0.055854
O -1.318340 2.829065 1.339472
O -3.770387 -1.020805 2.016217
C 2.049064 0.674122 -1.020423
C 2.766450 1.949665 -1.404222
C -0.473616 -1.595633 -3.944632
O -2.012521 -2.469415 0.481007
C 2.330808 3.171190 -1.077217
O -5.020586 -2.442425 -1.018529
C 0.047536 2.214064 -0.635172
C 2.998893 -1.660870 -0.359930
C 0.724504 0.890077 -0.243110
C 4.063038 1.720373 -2.150245
C 3.124332 4.423281 -1.335291
C -1.349224 2.445997 -0.033201
C -2.267952 1.240697 -0.291344
C -3.638215 1.429978 0.355693
C -4.575655 0.244950 0.124748
C -3.920866 -1.068508 0.598940
C -4.807449 -2.259236 0.164369
C -2.550395 -1.258420 -0.064725
C -0.257808 -0.286187 -0.468735
C -1.623171 -0.066221 0.179534
O -0.338410 -2.675397 -1.793552
C 5.908679 -3.136975 1.596525
C 5.178946 -2.479821 0.611838
C 3.784494 -2.421833 0.674695
C -5.937303 0.475736 0.781571
C -5.376843 -3.148875 1.229877
C 3.863691 -3.693457 2.736410
C 3.136599 -3.036358 1.748008
C 5.252248 -3.745280 2.663410
H 1.127671 3.567698 0.655064
H 2.822326 -0.075850 2.114764
C 0.043366 5.580173 -0.049684
H 3.972634 0.220551 -0.071209
H -0.711236 2.221707 1.810959
H -3.130382 -1.702452 2.262281

H 1.869525 0.095583 -1.925580
H -0.447007 6.375357 -0.609927
H 0.435222 -1.129341 -4.329125
H -1.316844 -0.978477 -4.255153
H -0.571461 -2.598646 -4.349310
H -1.342150 -2.795574 -0.141738
H -0.105896 2.147832 -1.716467
H 1.988938 -2.063962 -0.428194
H 3.457368 -1.775391 -1.344583
H 4.199460 2.445903 -2.951501
H 4.077445 0.726563 -2.601107
H 4.936579 1.790066 -1.495867
H 3.086806 5.077542 -0.460571
H 4.169601 4.212054 -1.548376
H -1.777683 3.301411 -0.557177
H -2.402492 1.177369 -1.377006
H -4.106790 2.333018 -0.045377
H -3.510713 1.588502 1.427022
H -4.726013 0.123080 -0.951266
H -2.733016 -1.381216 -1.134313
H 0.180631 -1.206177 -0.095019
H -1.451199 -0.008425 1.256608
H 0.937274 5.988320 0.434675
H 2.708018 4.993778 -2.168296
H 6.988839 -3.177159 1.529892
H 5.695691 -2.010842 -0.217739
H -5.838172 0.550929 1.865114
H -6.640070 -0.329648 0.558148
H -6.374812 1.405837 0.415124
H -5.900652 -2.558025 1.982178
H -4.561943 -3.662143 1.747000
H -6.044287 -3.881288 0.781173
H 3.345454 -4.167200 3.560850
H 2.055104 -3.001601 1.807618
H 5.819108 -4.258815 3.429698
H -0.636096 5.199100 0.716875
81
3-c1.out delta G = 1.8505 kcal/mol conformer 6
O -0.061863 -0.841329 -1.205001
C 0.003754 -2.157412 -1.443958
C 1.110466 3.194428 -0.386749
O -0.258675 1.730244 2.519482
C 0.624480 1.147599 1.884315
N 1.737271 0.645484 2.425925
O 0.750995 4.167513 -1.378655
C 2.667398 0.002611 1.511910
O -1.640740 3.116353 0.709201
O -4.409413 -0.518241 1.634876
C 2.112665 0.437000 0.118191
C 2.979421 1.543341 -0.442679
C 0.381560 -2.441002 -2.864390
O -2.389815 -2.283733 1.040102
C 2.558877 2.804451 -0.593712
O -4.861025 -2.643430 -1.206815
C 0.185205 1.991139 -0.538195
C 2.761963 -1.514249 1.757682
C 0.632731 0.833208 0.372957
C 4.390036 1.106925 -0.767262
C 3.467725 3.936385 -0.991555
C -1.306432 2.361786 -0.452428
C -2.195210 1.119260 -0.633307
C -3.674482 1.454126 -0.455939
C -4.586850 0.243105 -0.649032
C -4.166907 -0.910376 0.285374
C -4.969356 -2.178658 -0.088543
C -2.680770 -1.243648 0.098536
C -0.309852 -0.374729 0.155260
C -1.787014 -0.019963 0.308407
O -0.195465 -3.006356 -0.594534
C 4.362396 -3.510173 -1.062598
C 3.410708 -2.968772 -0.202695
C 3.792018 -2.163172 0.870502
C -6.058444 0.617979 -0.469874
C -5.858961 -2.792010 0.952267
C 6.107211 -2.466681 0.222989
C 5.153442 -1.927035 1.077608
C 5.713977 -3.255706 -0.855516
H 0.980400 3.645007 0.603649
H 1.901266 0.707201 3.420727
C 0.257689 5.397251 -0.860842
H 3.657069 0.429067 1.667187
H -1.264011 2.653837 1.486269
H -3.904724 -1.114373 2.204659
H 2.166406 -0.409718 -0.563238
H 1.020760 5.914680 -0.268991
H 0.235186 -3.494959 -3.082075
H 1.433222 -2.183382 -3.007062

H -0.205237 -1.824055 -3.543826
H -1.600633 -2.750215 0.719756
H 0.336468 1.626560 -1.558137
H 3.025807 -1.664095 2.807885
H 1.787263 -1.978485 1.605983
H 5.075036 1.245337 0.074104
H 4.796216 1.657909 -1.613554
H 4.411302 0.045426 -1.017248
H 3.306916 4.233683 -2.030063
H 3.255711 4.817174 -0.380273
H -1.508867 3.030368 -1.290103
H -2.038859 0.774529 -1.661642
H -3.960176 2.228190 -1.173585
H -3.830193 1.872495 0.539049
H -4.448266 -0.139397 -1.663785
H -2.568432 -1.622220 -0.919564
H -0.045038 -1.179504 0.835299
H -1.922842 0.306531 1.341477
H -0.628760 5.238601 -0.241700
H 4.518053 3.686997 -0.860514
H 4.045991 -4.130832 -1.891681
H 2.361926 -3.185949 -0.358675
H -6.251375 0.960712 0.547348
H -6.721520 -0.224371 -0.677521
H -6.323850 1.423690 -1.156312
H -6.419490 -3.617029 0.518214
H -6.533418 -2.043774 1.370172
H -5.246891 -3.158829 1.780361
H 7.158044 -2.271335 0.397144
H 5.469659 -1.310969 1.911430
H 6.456467 -3.673816 -1.523435
H -0.001745 6.017070 -1.718295
81
3-c8.out delta G = 2.1040 kcal/mol
conformer 7
O -0.403012 -0.280732 -1.875334
C -0.378364 -1.476786 -2.483353
C 0.955624 3.466129 -0.191370
O 0.561150 1.077210 2.349581
C 1.177792 0.755799 1.330963
N 2.352521 0.118275 1.319735
O 0.368884 4.696456 -0.653809
C 2.941824 -0.116722 0.008699
O -1.331533 2.791386 1.539996
O -3.764071 -1.129501 2.001881
C 2.054478 0.779706 -0.908776
C 2.783568 2.078545 -1.178524
C -0.425799 -1.336369 -3.972851
O -1.994420 -2.466620 0.390558
C 2.334804 3.274543 -0.782270
O -4.999348 -2.365656 -1.118928
C 0.035203 2.280845 -0.463280
C 2.987593 -1.602626 -0.385780
C 0.723374 0.945917 -0.131032
C 4.113453 1.910445 -1.879706
C 3.163287 4.528720 -0.880469
C -1.363028 2.475733 0.151750
C -2.274386 1.279357 -0.167518
C -3.643281 1.421024 0.493973
C -4.574871 0.247323 0.192697
C -3.911729 -1.089441 0.584269
C -4.790589 -2.255475 0.073768
C -2.538721 -1.229761 -0.086307
C -0.250904 -0.221959 -0.427044
C -1.619984 -0.047577 0.228124
O -0.310016 -2.533540 -1.884587
C 5.860711 -3.210561 1.520535
C 5.148450 -2.499599 0.560675
C 3.754620 -2.424905 0.615581
C -5.936517 0.429695 0.864291
C -5.358234 -3.212616 1.080221
C 3.798742 -3.789633 2.617952
C 3.089250 -3.078918 1.654317
C 5.187179 -3.856823 2.553903
H 1.034761 3.626733 0.886382
H 2.802507 -0.160560 2.179505
C 0.082799 4.760557 -2.046692
H 3.966793 0.249457 0.024898
H -0.727224 2.158815 1.980910
H -3.119130 -1.820199 2.206352
H 1.886359 0.269881 -1.856626
H -0.067442 5.812416 -2.284880
H 0.528088 -0.935508 -4.321818
H -1.205673 -0.632517 -4.261493
H -0.599637 -2.306261 -4.429717
H -1.322600 -2.754243 -0.249041
H -0.120860 2.235275 -1.544169
H 1.978463 -1.998533 -0.493769

H 3.461880 -1.660797 -1.367732
H 4.270016 2.690871 -2.623718
H 4.160704 0.949233 -2.393838
H 4.958229 1.948622 -1.186224
H 3.011112 5.143802 0.008884
H 4.225657 4.309541 -0.964299
H -1.800686 3.354208 -0.324561
H -2.413018 1.274451 -1.254702
H -4.117900 2.344557 0.150862
H -3.512223 1.514153 1.572620
H -4.726791 0.190779 -0.888547
H -2.718258 -1.292400 -1.161625
H 0.190751 -1.158894 -0.103428
H -1.449947 -0.049712 1.307187
H -0.827874 4.210343 -2.302591
H 2.878097 5.145549 -1.736738
H 6.940765 -3.262723 1.460809
H 5.678410 -2.000790 -0.242746
H -5.836055 0.439540 1.950235
H -6.635375 -0.364283 0.593129
H -6.379664 1.377961 0.555636
H -6.021717 -3.918316 0.585219
H -5.885532 -2.672502 1.867339
H -4.541676 -3.753939 1.565073
H 3.267061 -4.292665 3.416054
H 2.007952 -3.031045 1.706422
H 5.740617 -4.411597 3.301010
H 0.906625 4.372805 -2.652453
81
3-c6.out delta G = 2.5922 kcal/mol
conformer 8
O -0.066692 -0.745780 -1.187984
C 0.004933 -2.051732 -1.477170
C 1.068626 3.268388 -0.233347
O -0.275262 1.688017 2.632253
C 0.604695 1.123813 1.976996
N 1.707550 0.582092 2.501103
O 0.718026 4.325865 -1.144528
C 2.640002 -0.019101 1.561389
O -1.665650 3.128267 0.870919
O -4.417427 -0.556997 1.658673
C 2.100048 0.496243 0.188866
C 2.967472 1.647129 -0.273059
C 0.382991 -2.279120 -2.907594
O -2.387711 -2.285896 0.997274
C 2.532232 2.908548 -0.367731
O -4.854942 -2.574733 -1.262807
C 0.161443 2.056513 -0.419474
C 2.725833 -1.547819 1.726553
C 0.617239 0.872643 0.453971
C 4.406427 1.272618 -0.546616
C 3.460040 4.071554 -0.607655
C -1.332391 2.418395 -0.317119
C -2.214683 1.179222 -0.543688
C -3.695773 1.498675 -0.354595
C -4.600490 0.290358 -0.594389
C -4.172793 -0.895508 0.295110
C -4.967934 -2.153048 -0.128041
C -2.684646 -1.212317 0.096226
C -0.318959 -0.331928 0.188744
C -1.798553 0.007965 0.354043
O -0.189283 -2.933159 -0.660477
C 4.361497 -3.377076 -1.184832
C 3.398944 -2.884050 -0.308220
C 3.766726 -2.149454 0.819091
C -6.074485 0.648670 -0.401887
C -5.857473 -2.808362 0.886827
C 6.090738 -2.427814 0.191567
C 5.125950 -1.935844 1.062568
C 5.710850 -3.145076 -0.940470
H 0.882953 3.719702 0.744371
H 1.869584 0.600039 3.497947
C 0.801474 4.004878 -2.528937
H 3.631128 0.390570 1.747279
H -1.287526 2.637667 1.629800
H -3.908995 -1.171491 2.205155
H 2.168377 -0.303500 -0.546171
H 0.810530 4.951338 -3.067393
H 1.432759 -2.009463 -3.042070
H -0.209023 -1.641238 -3.562776
H 0.242982 -3.325039 -3.164641
H -1.594737 -2.733980 0.660498
H 0.309515 1.702594 -1.442657
H 2.976430 -1.753457 2.770421
H 1.752919 -2.002322 1.539026
H 4.491358 0.209723 -0.773452
H 5.060125 1.468730 0.308314
H 4.804739 1.830243 -1.393467

H 4.474718 3.852471 -0.280624
H 3.503169 4.358573 -1.661953
H -1.544452 3.118092 -1.126452
H -2.056602 0.873937 -1.584277
H -3.985109 2.297554 -1.043035
H -3.855178 1.878739 0.655140
H -4.458798 -0.051691 -1.623054
H -2.569473 -1.550602 -0.935713
H -0.051222 -1.160161 0.838606
H -1.937426 0.293211 1.398924
H -0.059618 3.419409 -2.865509
H 3.106166 4.947329 -0.061201
H 4.055320 -3.943337 -2.055635
H 2.351723 -3.084102 -0.493557
H -6.344165 1.479672 -1.055735
H -6.270593 0.949353 0.627924
H -6.732267 -0.188542 -0.644028
H -6.413951 -3.618613 0.420926
H -6.535574 -2.078362 1.330303
H -5.246343 -3.203316 1.702504
H 7.139624 -2.251117 0.394800
H 5.431608 -1.375325 1.938467
H 6.461844 -3.525889 -1.621048
H 1.713926 3.451884 -2.767417
81
3-c2.out delta G = 3.2016 kcal/mol
conformer 9
O 0.302497 -0.443480 -1.637429
C 0.375000 -1.662491 -2.189134
C 1.098745 3.458205 -0.405466
O 0.192288 1.341213 2.477904
C 1.100454 1.111607 1.673028
N 2.368524 0.903951 2.028330
O 0.420035 4.523346 -1.079370
C 3.325113 0.670924 0.964344
O -1.451778 2.907872 1.093518
O -3.581519 -1.220152 1.806930
C 2.462672 0.861371 -0.327659
C 3.022995 2.044108 -1.088596
C 0.709783 -1.576430 -3.647549
O -1.525098 -2.585504 0.599684
C 2.464985 3.260088 -1.041192
O -4.301141 -2.910746 -1.261813
C 0.271770 2.181265 -0.542523
C 4.074849 -0.664736 1.105014
C 0.980003 0.991565 0.138835
C 4.317456 1.721420 -1.785797
C 3.094147 4.509191 -1.595400
C -1.215679 2.350285 -0.196342
C -1.978611 1.033803 -0.442024
C -3.433046 1.136950 0.012256
C -4.233290 -0.138510 -0.250961
C -3.537758 -1.357311 0.387382
C -4.255170 -2.646713 -0.075703
C -2.080132 -1.456145 -0.081881
C 0.174519 -0.291090 -0.190428
C -1.296280 -0.174830 0.211964
O 0.207021 -2.697323 -1.577842
C 2.571265 -3.931100 -0.131831
C 3.308860 -2.753943 -0.074764
C 3.241401 -1.916913 1.040878
C -5.681983 0.005719 0.216716
C -4.878181 -3.528576 0.966388
C 1.655933 -3.456874 2.039068
C 2.405908 -2.287382 2.099679
C 1.739570 -4.285253 0.923936
H 1.206859 3.728382 0.652006
H 2.637745 0.952954 3.000930
C 0.006920 5.592586 -0.237324
H 4.080544 1.457091 1.007450
H -0.985756 2.348028 1.746572
H -2.911061 -1.812770 2.173304
H 2.566312 -0.028847 -0.945572
H -0.511157 6.310723 -0.871823
H 0.114960 -0.802410 -4.130929
H 0.542529 -2.538595 -4.123281
H 1.761243 -1.299013 -3.749740
H -0.756749 -2.874951 0.079233
H 0.285244 1.940561 -1.608174
H 4.831154 -0.691017 0.318694
H 4.617999 -0.633489 2.053843
H 5.102519 1.438475 -1.077230
H 4.695189 2.543320 -2.387153
H 4.171812 0.862060 -2.447095
H 2.571667 4.859490 -2.487341
H 3.024744 5.315646 -0.860615
H -1.612061 3.088975 -0.893299
H -1.966452 0.873829 -1.526186

H -3.916398 1.970165 -0.505508
H -3.461329 1.369463 1.077426
H -4.233940 -0.334431 -1.326470
H -2.105683 -1.646354 -1.156014
H 0.638662 -1.154681 0.280473
H -1.301168 -0.038499 1.294309
H 0.867334 6.087626 0.226907
H 4.144826 4.373433 -1.838269
H 2.635905 -4.564323 -1.007079
H 3.954306 -2.484324 -0.902456
H -6.282590 -0.869724 -0.038936
H -6.143242 0.872271 -0.260015
H -5.728798 0.147390 1.297065
H -4.098434 -3.923659 1.622396
H -5.405722 -4.350171 0.486771
H -5.554986 -2.952237 1.598408
H 1.010492 -3.724093 2.866337
H 2.348579 -1.665860 2.983569
H 1.157000 -5.196619 0.877182
H -0.670119 5.237964 0.543665
81
3-c12.out delta G = 3.2304 kcal/mol
conformer 10
O -0.365628 -0.377407 -1.948968
C -0.335721 -1.585061 -2.533712
C 0.980793 3.400443 -0.310402
O 0.507243 1.100465 2.251926
C 1.151442 0.750217 1.259594
N 2.333650 0.128370 1.297498
O 0.384811 4.584066 -0.862453
C 2.954475 -0.146593 0.008609
O -1.392667 2.761573 1.343752
O -3.809908 -1.052624 1.938259
C 2.077758 0.703712 -0.960221
C 2.798279 1.994428 -1.284369
C -0.370348 -1.470281 -4.026007
O -1.973036 -2.504986 0.355858
C 2.345446 3.203237 -0.933483
O -4.984703 -2.550324 -1.027701
C 0.056795 2.220890 -0.595823
C 3.019404 -1.644040 -0.333163
C 0.730069 0.890904 -0.217565
C 4.117917 1.794599 -1.997506
C 3.138864 4.466808 -1.127759
C -1.363465 2.424954 -0.041572
C -2.256960 1.217740 -0.368377
C -3.667995 1.396986 0.188564
C -4.572114 0.185819 -0.054717
C -3.911883 -1.093191 0.514895
C -4.759799 -2.322521 0.145162
C -2.530655 -1.287983 -0.137979
C -0.237577 -0.285095 -0.500926
C -1.618355 -0.086936 0.122142
O -0.276048 -2.630423 -1.915108
C 3.809724 -3.753605 2.731337
C 3.106452 -3.073673 1.741151
C 3.780313 -2.428404 0.702456
C -5.974022 0.414749 0.509789
C -5.275719 -3.194894 1.248595
C 5.882185 -3.158906 1.662481
C 5.176208 -2.479044 0.675905
C 5.199991 -3.797791 2.694776
H 1.085486 3.539138 0.771435
H 2.764656 -0.118951 2.176454
C 0.013845 5.568164 0.096189
H 3.974626 0.233026 0.033415
H -0.780415 2.160241 1.817082
H -3.475740 -0.188715 2.205358
H 1.929173 0.150243 -1.886543
H -0.685146 5.160589 0.831018
H -1.191324 -0.822263 -4.332516
H -0.480223 -2.455369 -4.469893
H 0.558383 -1.012771 -4.372051
H -1.325479 -2.804385 -0.302852
H -0.056987 2.185333 -1.683318
H 2.014127 -2.050312 -0.440320
H 3.505746 -1.730142 -1.307280
H 4.970565 1.839821 -1.314085
H 4.277916 2.549974 -2.766038
H 4.147937 0.818498 -2.484892
H 2.743757 5.062032 -1.953721
H 3.071979 5.092088 -0.233767
H -1.779187 3.292492 -0.555675
H -2.328052 1.162555 -1.459576
H -4.128094 2.277515 -0.266932
H -3.608644 1.618890 1.258566
H -4.652869 0.023606 -1.132280
H -2.717366 -1.377992 -1.211581

H 0.195076 -1.214425 -0.144539
H -1.456384 -0.031578 1.202088
H -0.465256 6.377298 -0.453665
H 4.191130 4.267773 -1.316584
H 3.271619 -4.251164 3.528565
H 2.023589 -3.045093 1.771965
H -6.404663 1.322028 0.083340
H -5.946925 0.532048 1.594281
H -6.645544 -0.413127 0.274781
H -5.879542 -2.606196 1.941202
H -4.434662 -3.584386 1.825182
H -5.860723 -4.010889 0.830015
H 6.963928 -3.193027 1.624137
H 5.712935 -1.986491 -0.126905
H 5.748337 -4.329128 3.462400
H 0.891008 5.966088 0.618086
81
3-c16-gfn0.out delta G = 3.2480 kcal/mol
conformer 11
O -0.524621 -0.391612 -1.926957
C -0.222457 -1.519701 -2.602934
C 1.007618 3.332865 -0.391283
O 0.521682 1.064607 2.235170
C 1.153660 0.707769 1.236220
N 2.343933 0.102966 1.269871
O 0.411984 4.519299 -0.938357
C 2.950248 -0.197219 -0.019073
O -1.328364 2.783460 1.345890
O -3.761847 -1.036222 1.981725
C 2.040914 0.605527 -1.001739
C 2.758097 1.875142 -1.406245
C -0.606233 -1.386755 -4.047671
O -2.007419 -2.489700 0.506347
C 2.340214 3.100129 -1.069082
O -5.071380 -2.441545 -1.038249
C 0.052983 2.165538 -0.620625
C 3.043662 -1.703099 -0.307477
C 0.710351 0.827061 -0.235431
C 4.040845 1.634410 -2.172062
C 3.142270 4.345144 -1.335672
C -1.346895 2.411274 -0.030530
C -2.275772 1.218286 -0.306160
C -3.660492 1.427137 0.297589
C -4.594604 0.246937 0.035435
C -3.970969 -1.062219 0.566714
C -4.843944 -2.264936 0.142478
C -2.570525 -1.290659 -0.032823
C -0.294539 -0.326709 -0.493363
C -1.646283 -0.091179 0.181339
O 0.281397 -2.495996 -2.097791
C 5.975352 -3.053233 1.707837
C 5.237524 -2.430282 0.707038
C 3.840755 -2.427347 0.744588
C -5.989148 0.500082 0.612761
C -5.375246 -3.178626 1.207651
C 3.934523 -3.685340 2.814899
C 3.199731 -3.062054 1.810219
C 5.325102 -3.682305 2.766585
H 1.159218 3.486625 0.683060
H 2.786924 -0.125317 2.148056
C 0.097344 5.524824 0.017716
H 3.961356 0.207430 -0.022052
H -0.736753 2.161382 1.818340
H -4.554198 -0.720238 2.429858
H 1.872954 0.007529 -1.896555
H -0.384140 6.336330 -0.526693
H -0.236460 -2.240804 -4.608016
H -0.205840 -0.460760 -4.460191
H -1.693968 -1.340674 -4.127143
H -2.034732 -2.404631 1.469454
H -0.094206 2.107953 -1.702849
H 2.045344 -2.129513 -0.388006
H 3.517857 -1.815297 -1.285110
H 4.923715 1.675504 -1.527692
H 4.182100 2.370786 -2.962388
H 4.030162 0.648323 -2.639735
H 2.727293 4.916862 -2.168578
H 3.114721 5.002447 -0.462983
H -1.758383 3.275990 -0.552852
H -2.385257 1.151051 -1.393263
H -4.107234 2.332482 -0.122444
H -3.567519 1.590895 1.372398
H -4.694057 0.107108 -1.043989
H -2.701792 -1.475898 -1.097886
H 0.118530 -1.279112 -0.177116
H -1.463382 -0.013952 1.255315
H 1.001395 5.911306 0.501193
H 4.184727 4.124341 -1.553246

H 7.057323 -3.050607 1.660375
H 5.749694 -1.944592 -0.115852
H -6.677832 -0.320696 0.404113
H -6.412348 1.405498 0.176139
H -5.958978 0.657113 1.694880
H -4.549389 -3.578798 1.797972
H -5.942079 -3.985847 0.749391
H -6.016325 -2.627187 1.899890
H 3.420819 -4.175342 3.632711
H 2.116858 -3.069367 1.850099
H 5.898455 -4.169131 3.545415
H -0.582608 5.142954 0.783509
81
3-c7.out delta G = 3.4080 kcal/mol
conformer 12
O -0.052847 -0.837517 -1.223804
C 0.013883 -2.149126 -1.487219
C 1.108862 3.190145 -0.362628
O -0.255236 1.716797 2.514148
C 0.626501 1.127952 1.882240
N 1.732604 0.614853 2.426423
O 0.754086 4.171250 -1.348027
C 2.666983 -0.020427 1.510671
O -1.663342 3.075834 0.700789
O -4.422828 -0.489528 1.657414
C 2.120923 0.430555 0.118787
C 2.987226 1.548508 -0.419842
C 0.389650 -2.403921 -2.913771
O -2.348677 -2.308165 1.029734
C 2.561590 2.809448 -0.558833
O -4.902315 -2.709000 -1.037095
C 0.192830 1.982846 -0.535693
C 2.759112 -1.540128 1.739457
C 0.639418 0.820034 0.370035
C 4.403217 1.123666 -0.736385
C 3.468691 3.950974 -0.932276
C -1.302857 2.340383 -0.465713
C -2.174596 1.089477 -0.667061
C -3.662815 1.415979 -0.558445
C -4.563544 0.187680 -0.711253
C -4.160228 -0.899201 0.315246
C -4.968132 -2.182682 0.056859
C -2.675805 -1.258864 0.119924
C -0.299153 -0.389158 0.142101
C -1.776338 -0.034067 0.299609
O -0.180997 -3.014320 -0.654056
C 6.115954 -2.471975 0.217632
C 5.155534 -1.944196 1.072201
C 3.795766 -2.177637 0.851557
C -6.041727 0.562082 -0.608686
C -5.806918 -2.736541 1.167839
C 4.381207 -3.497929 -1.095378
C 3.422794 -2.968530 -0.235489
C 5.731117 -3.246166 -0.874546
H 0.967229 3.630235 0.630945
H 1.894631 0.672925 3.421780
C 0.244714 5.391143 -0.822234
H 3.656157 0.403002 1.676521
H -1.267129 2.625557 1.475885
H -4.101582 0.410929 1.780192
H 2.181572 -0.406354 -0.574158
H -0.649619 5.218971 -0.218106
H 1.443914 -2.152772 -3.049234
H -0.190668 -1.766873 -3.579984
H 0.234728 -3.451541 -3.154923
H -1.584743 -2.774297 0.653343
H 0.358122 1.629026 -1.557137
H 3.015611 -1.702466 2.789597
H 1.785899 -2.002970 1.575245
H 4.432982 0.064782 -0.996485
H 5.080264 1.258144 0.112017
H 4.813098 1.685291 -1.573869
H 4.518786 3.705215 -0.792815
H 3.317717 4.260727 -1.968600
H -1.500963 3.015573 -1.299129
H -1.979836 0.733184 -1.683950
H -3.930682 2.146711 -1.325814
H -3.854297 1.913428 0.397531
H -4.384485 -0.252791 -1.695214
H -2.591876 -1.624470 -0.907119
H -0.035236 -1.200780 0.814010
H -1.893231 0.310191 1.330569
H -0.006565 6.019849 -1.675587
H 3.245289 4.822720 -0.312121
H 7.165382 -2.278991 0.402551
H 5.465262 -1.339659 1.916824
H -6.690326 -0.296247 -0.793465
H -6.285521 1.326550 -1.348127

H -6.279763 0.960254 0.378886
H -6.305363 -3.644431 0.835330
H -6.540045 -1.994165 1.488367
H -5.174198 -2.940703 2.033222
H 4.071321 -4.107337 -1.935206
H 2.375326 -3.183781 -0.402701
H 6.478739 -3.655072 -1.542437
H 0.995493 5.906107 -0.212849
81
3-c9-gfn0.out delta G = 3.8855 kcal/mol
conformer 13
O -0.240573 -0.861798 -1.246151
C 0.117511 -2.133403 -1.509324
C 1.109225 3.120841 -0.379235
O -0.224132 1.655372 2.519364
C 0.620247 1.040486 1.860815
N 1.707703 0.463623 2.377858
O 0.755861 4.120319 -1.347502
C 2.633950 -0.138086 1.431502
O -1.637506 3.070909 0.745951
O -4.414359 -0.417715 1.696362
C 2.061155 0.334803 0.056396
C 2.942134 1.433238 -0.499176
C 0.226715 -2.360205 -2.988105
O -2.453899 -2.226872 1.190298
C 2.546488 2.706281 -0.614803
O -5.015872 -2.619551 -1.058555
C 0.158922 1.938599 -0.539168
C 2.761157 -1.660898 1.610158
C 0.594447 0.755797 0.346449
C 4.338294 0.974142 -0.853129
C 3.472716 3.829590 -0.997144
C -1.324531 2.335287 -0.434847
C -2.234351 1.110924 -0.627390
C -3.709380 1.474076 -0.495126
C -4.625744 0.264738 -0.673563
C -4.256941 -0.841733 0.339098
C -5.082283 -2.114551 0.045017
C -2.770957 -1.228833 0.216373
C -0.388022 -0.424699 0.132568
C -1.846851 -0.016822 0.337132
O 0.344300 -2.961601 -0.656550
C 4.789503 -3.368672 -1.127398
C 3.711413 -2.931898 -0.361288
C 3.918722 -2.206854 0.812305
C -6.101132 0.662891 -0.589087
C -5.932478 -2.687366 1.140440
C 6.309815 -2.374928 0.449152
C 5.231648 -1.940632 1.210622
C 6.091610 -3.087283 -0.727995
H 1.003658 3.556026 0.620944
H 1.893891 0.506230 3.369733
C 0.289724 5.347006 -0.798570
H 3.618051 0.299029 1.594071
H -1.237525 2.601513 1.507479
H -5.241495 0.065452 1.799871
H 2.086320 -0.502269 -0.639136
H 0.029280 5.988539 -1.639636
H 0.334246 -3.421991 -3.191018
H 1.104884 -1.829522 -3.362264
H -0.646386 -1.960040 -3.502282
H -2.708446 -1.869304 2.052117
H 0.292156 1.587585 -1.566312
H 2.918927 -1.860484 2.673227
H 1.833461 -2.151739 1.317342
H 5.029973 1.049696 -0.009481
H 4.756696 1.554061 -1.673479
H 4.327767 -0.074290 -1.154545
H 3.305714 4.155184 -2.026119
H 3.284901 4.699209 -0.362299
H -1.521835 3.023618 -1.257738
H -2.063040 0.751776 -1.647176
H -3.966633 2.227087 -1.245020
H -3.885177 1.928862 0.481147
H -4.449262 -0.170601 -1.660455
H -2.640733 -1.706909 -0.753494
H -0.138157 -1.260171 0.779613
H -1.937625 0.352510 1.360722
H 1.068792 5.839856 -0.206485
H 4.519412 3.557073 -0.884596
H 4.609690 -3.929502 -2.036377
H 2.700494 -3.160413 -0.671573
H -6.327405 1.408641 -1.352169
H -6.349315 1.115563 0.375458
H -6.767183 -0.186788 -0.749259
H -5.313283 -2.907208 2.011619
H -6.427732 -3.589020 0.787227
H -6.680184 -1.959603 1.465157

H 7.320338 -2.158480 0.773083
H 5.411100 -1.384631 2.123955
H 6.930434 -3.424405 -1.323936
H -0.591104 5.190168 -0.170910
81
3-c20-gfn0.out delta G = 3.9113 kcal/mol
conformer 14
O -0.466498 -0.213153 -1.900213
C -0.165018 -1.295426 -2.649238
C 0.959013 3.416004 -0.092604
O 0.478747 0.967901 2.375470
C 1.128139 0.673537 1.367326
N 2.311539 0.055651 1.380607
O 0.384363 4.661744 -0.529417
C 2.944893 -0.147694 0.085386
O -1.388058 2.701613 1.563122
O -3.876195 -1.206725 1.915614
C 2.064274 0.742268 -0.848562
C 2.792012 2.046204 -1.094625
C -0.477999 -1.038363 -4.093957
O -1.962167 -2.495367 0.416041
C 2.344288 3.233969 -0.673060
O -4.946929 -2.448695 -1.208311
C 0.040183 2.242495 -0.414274
C 3.038788 -1.626845 -0.320298
C 0.714071 0.892830 -0.100671
C 4.125903 1.891976 -1.791279
C 3.179793 4.486111 -0.734456
C -1.374522 2.428711 0.165188
C -2.278227 1.243919 -0.211932
C -3.680904 1.391295 0.367649
C -4.587209 0.211315 0.012681
C -3.952064 -1.113684 0.486915
C -4.802033 -2.296317 -0.011430
C -2.531980 -1.288119 -0.093142
C -0.268035 -0.252796 -0.462285
C -1.635274 -0.084664 0.201742
O 0.295552 -2.319135 -2.200794
C 3.783618 -3.838157 2.683695
C 3.096431 -3.134496 1.698818
C 3.786371 -2.439202 0.703749
C -6.005016 0.404594 0.552520
C -5.422041 -3.212847 0.999529
C 5.873640 -3.168284 1.696966
C 5.183190 -2.464998 0.715752
C 5.175020 -3.856511 2.685760
H 1.024427 3.546796 0.990217
H 2.740022 -0.230795 2.248821
C 0.120213 4.765809 -1.924347
H 3.959115 0.244865 0.138175
H -0.783395 2.066042 2.000660
H -3.645338 -0.343184 2.278194
H 1.925731 0.232692 -1.801722
H -0.019467 5.824780 -2.136043
H -0.211305 -1.906827 -4.689342
H 0.073588 -0.163642 -4.441030
H -1.541520 -0.823021 -4.206233
H -2.080296 -2.468247 1.376834
H -0.088902 2.224554 -1.499055
H 2.043779 -2.039413 -0.477013
H 3.554037 -1.664190 -1.282675
H 4.177171 0.939353 -2.320615
H 4.967295 1.920600 -1.093277
H 4.284388 2.684985 -2.521584
H 3.009492 5.089767 0.159224
H 4.242881 4.262373 -0.796192
H -1.794172 3.322022 -0.299518
H -2.363566 1.246779 -1.302911
H -4.136975 2.310201 -0.009330
H -3.617232 1.520972 1.452851
H -4.637566 0.135028 -1.076712
H -2.630988 -1.428818 -1.168701
H 0.149689 -1.220437 -0.204259
H -1.457869 -0.070338 1.281004
H -0.789702 4.228859 -2.209643
H 2.918991 5.116119 -1.589027
H 3.232398 -4.373717 3.446675
H 2.012932 -3.124125 1.699177
H -6.413565 1.350136 0.193034
H -6.014526 0.428281 1.643599
H -6.676173 -0.391378 0.225165
H -4.637627 -3.682944 1.596362
H -6.015646 -3.970786 0.493385
H -6.038770 -2.643050 1.696223
H 6.956523 -3.181901 1.688808
H 5.733059 -1.933484 -0.052634
H 5.711536 -4.405496 3.449344
H 0.950780 4.388943 -2.527522

81
3-c12-gfn0.out delta G = 3.9201 kcal/mol
conformer 15
O -0.245954 -0.758375 -1.219870
C 0.091067 -2.024519 -1.536305
C 1.066287 3.197912 -0.220891
O -0.231281 1.610160 2.642101
C 0.609236 1.017258 1.959170
N 1.689003 0.405084 2.450976
O 0.720264 4.274705 -1.111748
C 2.614135 -0.154856 1.477791
O -1.663391 3.076088 0.927244
O -4.538296 -0.499493 1.729518
C 2.050607 0.397710 0.127798
C 2.930762 1.540124 -0.331507
C 0.205206 -2.188146 -3.022820
O -2.461546 -2.219918 1.187564
C 2.519739 2.811988 -0.388145
O -4.954084 -2.584112 -1.086727
C 0.134077 2.006194 -0.409767
C 2.733412 -1.686047 1.575672
C 0.581946 0.797213 0.434649
C 4.356724 1.143847 -0.640941
C 3.466455 3.961365 -0.620873
C -1.350936 2.392616 -0.282905
C -2.253021 1.171718 -0.522554
C -3.730721 1.519626 -0.379663
C -4.640301 0.311846 -0.609018
C -4.265384 -0.830161 0.361068
C -5.087068 -2.084676 0.013103
C -2.773063 -1.203482 0.233520
C -0.394669 -0.379838 0.174967
C -1.856482 0.010070 0.396998
O 0.297032 -2.891818 -0.718383
C 4.778619 -3.202278 -1.260548
C 3.695390 -2.815360 -0.475383
C 3.894401 -2.188897 0.755178
C -6.118661 0.692353 -0.520579
C -6.025288 -2.638758 1.042135
C 6.288229 -2.361081 0.412210
C 5.204673 -1.974611 1.191722
C 6.078120 -2.971470 -0.822311
H 0.907045 3.640154 0.765721
H 1.876714 0.405539 3.443472
C 0.776025 3.971996 -2.501631
H 3.600526 0.265314 1.667356
H -1.250293 2.583644 1.667218
H -4.318965 0.427463 1.882129
H 2.087256 -0.391649 -0.621447
H 0.793239 4.925692 -3.026986
H 1.085015 -1.643371 -3.371944
H -0.666059 -1.765001 -3.521711
H 0.310653 -3.240556 -3.270486
H -2.802932 -1.907498 2.038252
H 0.259309 1.666350 -1.440601
H 2.886604 -1.941681 2.627185
H 1.806573 -2.159667 1.253813
H 5.028831 1.304732 0.206785
H 4.751023 1.715180 -1.480483
H 4.415556 0.085577 -0.895822
H 4.481167 3.717006 -0.312280
H 3.500669 4.266064 -1.670566
H -1.560525 3.114200 -1.073427
H -2.081378 0.852186 -1.555104
H -3.993334 2.299606 -1.098780
H -3.909855 1.959230 0.606841
H -4.441683 -0.079957 -1.610026
H -2.630345 -1.653650 -0.748031
H -0.142163 -1.238916 0.789046
H -1.937036 0.338589 1.437168
H -0.100496 3.406410 -2.832332
H 3.137468 4.834012 -0.054227
H 4.604793 -3.685195 -2.214266
H 2.686780 -3.007906 -0.815666
H -6.770163 -0.149135 -0.762485
H -6.338417 1.494137 -1.226947
H -6.378215 1.045609 0.478925
H -6.561247 -3.490619 0.629678
H -6.723061 -1.867020 1.370439
H -5.460715 -2.940040 1.927071
H 7.296618 -2.186158 0.766366
H 5.377526 -1.496530 2.149292
H 6.921228 -3.269921 -1.432612
H 1.674619 3.406488 -2.762426

**Table S23.** Geometry data of conformers of compound **4**.

4-c8 , delta G = 0.0000 kcal/mol, population =

30.25 %
O 0.500721 0.096245 1.986455
C 0.553713 -0.925560 2.852720
C -1.156786 3.232166 -0.491069
O -0.544972 0.533415 -2.395346
C -1.164313 0.379943 -1.349558
N -2.342311 -0.256440 -1.222720
O -0.602625 4.566163 -0.488340
C -2.928066 -0.272754 0.108733
O 1.878757 3.607599 -0.287424
O 3.651160 -1.551181 -1.819816
C -2.050485 0.770742 0.866994
C -2.802955 2.076495 0.971108
C 0.653648 -0.441553 4.266037
O 2.086563 -2.504249 0.205751
C -2.443382 3.185919 0.314828
O 5.203392 -1.945981 1.378180
C -0.121117 2.257555 0.082962
C -2.950260 -1.670340 0.750648
C -0.731291 0.846558 0.060794
C -4.050114 1.991899 1.824631
C -3.278714 4.436015 0.308692
C 1.257937 2.350406 -0.595339
C 2.231196 1.269904 -0.125316
C 3.520405 1.294032 -0.947274
C 4.536154 0.250663 -0.481121
C 3.912203 -1.160319 -0.473177
C 4.887383 -2.140087 0.220274
C 2.604843 -1.174520 0.331714
C 0.289358 -0.188681 0.571783
C 1.613064 -0.127542 -0.177525
O 0.514577 -2.094873 2.517301
C -3.755340 -4.369573 -1.805440
C -3.047390 -3.490559 -0.990931
C -3.713459 -2.663801 -0.084049
C 5.817849 0.304310 -1.313100
C 5.411726 -3.301978 -0.571081
C -5.817380 -3.615308 -0.820639
C -5.106650 -2.737096 -0.009696
C -5.142859 -4.434077 -1.722765
H -1.353119 2.966914 -1.536229
H -2.784724 -0.693485 -2.017716
C -0.807327 5.299153 -1.697040
H -3.957502 0.077865 0.038949
H 1.160713 4.263332 -0.319057
H 3.018977 -2.282302 -1.789979
H -1.858913 0.407078 1.876186
H -1.868651 5.467385 -1.888645
H 1.482245 0.260465 4.360966
H 0.795139 -1.284080 4.936527
H -0.261489 0.091587 4.529680
H 1.458515 -2.641409 0.934319
H 0.026268 2.520361 1.134740
H -1.931863 -2.024875 0.909527
H -3.412208 -1.567837 1.735045
H -4.961654 1.960208 1.222083
H -4.136142 2.844586 2.498340
H -4.036851 1.090712 2.439052
H -4.127045 4.363724 0.983758
H -2.686242 5.307939 0.587453
H 1.125224 2.259412 -1.677971
H 2.473360 1.500877 0.917920
H 3.976018 2.282616 -0.884484
H 3.276211 1.116678 -1.999151
H 4.790785 0.462609 0.560699
H 2.865901 -0.973373 1.373110
H -0.131473 -1.186446 0.495370
H 1.386332 -0.371462 -1.216863
H -0.367210 4.774082 -2.550043
H -3.675645 4.625417 -0.693845
H -3.223001 -5.005649 -2.501703
H -1.966658 -3.445958 -1.056914
H 6.237946 1.311043 -1.285346
H 5.617566 0.047899 -2.354009
H 6.578720 -0.380947 -0.933645
H 6.140445 -3.852385 0.019926
H 5.853844 -2.958117 -1.506974
H 4.582419 -3.960933 -0.840838
H -6.896763 -3.663106 -0.746678
H -5.637383 -2.104354 0.692543
H -5.694728 -5.119572 -2.353485
H -0.311332 6.259408 -1.568462

4-c17 , delta G = 0.2322 kcal/mol, population = 20.43 %
O 0.581828 0.382102 1.855492
C 0.277967 -0.361099 2.938992
C -0.999970 3.261167 -0.872808

O -0.549966 0.299916 -2.545768
C -1.152684 0.247522 -1.479825
N -2.313594 -0.398168 -1.272677
O -0.465967 4.609872 -0.773508
C -2.916406 -0.234717 0.040772
O 1.982347 3.454805 -0.923609
O 3.519681 -1.831449 -1.732186
C -2.053305 0.917713 0.651972
C -2.814351 2.212370 0.482595
C 0.686175 0.350001 4.196410
O 1.918951 -2.540996 0.415312
C -2.390832 3.218683 -0.288975
O 5.198409 -3.291541 -0.438830
C -0.045223 2.242394 -0.248283
C -2.959791 -1.531013 0.866516
C -0.718145 0.863714 -0.129601
C -4.155159 2.256772 1.179530
C -3.268008 4.395602 -0.634187
C 1.312730 2.186275 -0.978334
C 2.262687 1.162101 -0.363620
C 3.565090 1.048602 -1.153644
C 4.530643 0.042204 -0.525177
C 3.844497 -1.344002 -0.433618
C 4.775720 -2.367941 0.233584
C 2.525732 -1.251126 0.358824
C 0.279621 -0.120069 0.524523
C 1.587116 -0.209008 -0.251952
O -0.259554 -1.443235 2.889169
C -5.746328 -3.701452 -0.551461
C -5.076811 -2.718007 0.169138
C -3.682822 -2.631686 0.137051
C 5.856336 -0.019468 -1.281023
C 5.126634 -2.211439 1.681745
C -3.640205 -4.538881 -1.359423
C -2.974138 -3.554397 -0.635449
C -5.029232 -4.614933 -1.319875
H -1.046355 3.104840 -1.954680
H -2.764592 -0.907339 -2.018058
C -0.330314 5.132243 0.552877
H -3.944674 0.097242 -0.094976
H 1.311137 4.129321 -1.119927
H 4.110746 -2.590282 -1.881479
H -1.898715 0.728657 1.714600
H -1.197467 4.893213 1.170051
H 0.104469 1.267891 4.297511
H 1.738140 0.631223 4.143649
H 0.511765 -0.290799 5.056040
H 1.887998 -2.863774 -0.497367
H 0.151485 2.559377 0.778557
H -1.952863 -1.849726 1.127222
H -3.470882 -1.299069 1.803630
H -4.987282 2.076619 0.493163
H -4.325413 3.228304 1.643821
H -4.210958 1.499415 1.962595
H -3.012791 4.776266 -1.624055
H -4.321744 4.121450 -0.635203
H 1.132439 1.917170 -2.022616
H 2.501034 1.512534 0.646340
H 4.049864 2.023644 -1.201044
H 3.345131 0.744973 -2.181294
H 4.728006 0.370563 0.499721
H 2.748983 -0.976714 1.389520
H -0.156779 -1.106950 0.628182
H 1.334857 -0.552122 -1.257268
H -0.249632 6.212753 0.449912
H -3.143917 5.228014 0.064197
H -6.827185 -3.757082 -0.511241
H -5.641139 -2.011606 0.767374
H 6.583314 -0.666488 -0.785947
H 6.293021 0.977963 -1.349061
H 5.705748 -0.392632 -2.295271
H 5.285346 -1.169623 1.956974
H 6.010957 -2.801115 1.912540
H 4.290590 -2.584209 2.280143
H -3.074343 -5.247537 -1.951449
H -1.892391 -3.499507 -0.667999
H -5.549141 -5.381853 -1.880121
H 0.569214 4.749629 1.039827

4-c9 , delta G = 0.2472 kcal/mol, population = 19.92 %

O -0.084139 -0.803016 -1.270453
C -0.036543 -2.103719 -1.582969
C 1.188226 3.132389 -0.107657
O -0.319761 1.637389 2.524894
C 0.571115 1.050264 1.922336
N 1.675172 0.524726 2.481179
O 0.814134 4.305532 -0.861353

C 2.616603 -0.115789 1.578331
O -1.604697 3.302368 -1.367037
O -4.356004 -0.598302 1.713125
C 2.107490 0.355046 0.178942
C 3.006040 1.455104 -0.339718
C 0.358248 -2.315006 -3.011355
O -2.423236 -2.341706 0.891935
C 2.623932 2.736167 -0.411903
O -4.997990 -2.433970 -1.288652
C 0.232871 1.982964 -0.451764
C 2.674456 -1.640668 1.787447
C 0.630501 0.766107 0.402070
C 4.396894 0.980133 -0.694744
C 3.558248 3.857223 -0.772297
C -1.256756 2.355046 -0.345445
C -2.187837 1.159234 -0.541767
C -3.638871 1.537187 -0.242512
C -4.609520 0.379920 -0.478261
C -4.184655 -0.868090 0.323229
C -5.043753 -2.071967 -0.128818
C -2.717433 -1.225453 0.043626
C -0.320089 -0.412533 0.116640
C -1.784073 -0.044027 0.312975
O -0.258929 -2.997243 -0.786015
C 4.281716 -3.590862 -1.059927
C 3.326272 -3.050780 -0.203422
C 3.705781 -2.291385 0.903796
C -6.052702 0.787673 -0.180653
C -5.912033 -2.750542 0.889514
C 6.025660 -2.638602 0.295849
C 5.068215 -2.100088 1.147187
C 5.634915 -3.381292 -0.815905
H 1.077193 3.382934 0.953550
H 1.816840 0.563320 3.480176
C 0.777387 5.514009 -0.101285
H 3.611855 0.286443 1.763885
H -0.853565 3.919259 -1.410129
H -3.835385 -1.252685 2.198816
H 2.167368 -0.477519 -0.519275
H 0.053837 5.438126 0.715894
H 1.430758 -2.130155 -3.106011
H -0.159100 -1.610543 -3.660822
H 0.143998 -3.338209 -3.306559
H -1.641305 -2.785938 0.523953
H 0.415233 1.709925 -1.495106
H 2.912955 -1.822851 2.838613
H 1.692455 -2.077125 1.604063
H 5.100547 1.104676 0.133019
H 4.803420 1.515752 -1.551019
H 4.385053 -0.082048 -0.940396
H 3.160998 4.455550 -1.592825
H 3.687913 4.531412 0.080222
H -1.444154 2.790763 0.640508
H -2.109159 0.873904 -1.596617
H -3.934424 2.378303 -0.870405
H -3.713664 1.868842 0.797651
H -4.541926 0.087486 -1.529490
H -2.654310 -1.526861 -1.004406
H -0.059005 -1.257781 0.747285
H -1.890954 0.223391 1.365318
H 0.470545 6.304095 -0.784046
H 4.544658 3.496416 -1.049564
H 3.967064 -4.175432 -1.915511
H 2.275342 -3.231329 -0.390208
H -6.175808 1.045233 0.871882
H -6.759009 -0.009562 -0.421098
H -6.323534 1.659663 -0.778202
H -5.283051 -3.201117 1.661441
H -6.510933 -3.520682 0.408420
H -6.550368 -2.023247 1.392749
H 7.077360 -2.478568 0.498568
H 5.382232 -1.520247 2.007421
H 6.380279 -3.798668 -1.481089
H 1.758682 5.757593 0.310310

4-c7 , delta G = 0.8013 kcal/mol, population = 7.81 %
O 0.258177 -0.613053 1.321754
C -0.097442 -1.822024 1.796428
C -1.034546 3.189033 -0.123864
O 0.317092 1.438195 -2.734458
C -0.510343 0.845684 -2.051982
N -1.539985 0.122636 -2.528329
O -0.777925 4.333420 0.736795
C -2.506328 -0.345717 -1.547577
O 1.784773 3.459543 0.830537
O 4.362837 -0.698316 -1.782190
C -2.030418 0.375479 -0.241221

C -2.929152 1.568002 -0.008458
C -0.190220 -1.798289 3.293755
O 2.407497 -2.418519 -0.847719
C -2.500363 2.833127 -0.071414
O 5.871977 -2.574345 -0.872574
C -0.112291 2.020182 0.225597
C -2.604034 -1.880120 -1.469087
C -0.550048 0.741712 -0.510189
C -4.386915 1.232946 0.210411
C -3.445887 4.007448 -0.083826
C 1.376744 2.358137 0.005923
C 2.294271 1.187632 0.348163
C 3.758091 1.508207 0.052421
C 4.682656 0.343362 0.408672
C 4.247846 -0.917697 -0.379418
C 5.135856 -2.117321 -0.016352
C 2.772872 -1.263428 -0.094891
C 0.399433 -0.411734 -0.112495
C 1.858102 -0.076375 -0.401516
O -0.328938 -2.781890 1.096813
C -6.223120 -2.483302 -0.475941
C -5.091963 -2.175047 -1.222064
C -3.813224 -2.300353 -0.672093
C 6.151089 0.691661 0.174294
C 5.064444 -2.696719 1.363619
C -4.825196 -3.064119 1.393047
C -3.694007 -2.756230 0.641246
C -6.093178 -2.924298 0.839099
H -0.778222 3.575797 -1.114516
H -1.703332 0.053840 -3.522322
C -1.071801 4.147402 2.126280
H -3.488571 0.025538 -1.835990
H 1.079316 4.124814 0.768997
H 5.093033 -1.270528 -2.076971
H -2.119924 -0.307964 0.602851
H -0.264182 3.615966 2.634378
H 0.720029 -1.380075 3.723162
H -0.357435 -2.804003 3.668755
H -1.021342 -1.155100 3.589479
H 2.675468 -2.239307 -1.761091
H -0.243285 1.804721 1.288663
H -2.684757 -2.260093 -2.490585
H -1.698358 -2.298603 -1.032603
H -4.826928 1.877120 0.971550
H -4.502195 0.198657 0.534281
H -4.982894 1.352166 -0.698757
H -4.413305 3.736543 -0.503194
H -3.620523 4.414937 0.915859
H 1.518890 2.620482 -1.045933
H 2.191931 1.003134 1.422844
H 4.060186 2.388018 0.620222
H 3.875429 1.749765 -1.008057
H 4.541111 0.122684 1.470963
H 2.669160 -1.541130 0.953776
H 0.122739 -1.333684 -0.612437
H 1.933176 0.116818 -1.473586
H -2.003002 3.597780 2.270208
H -3.028407 4.817890 -0.683247
H -7.205902 -2.380065 -0.919114
H -5.201988 -1.828778 -2.243519
H 6.411147 1.598671 0.721942
H 6.341257 0.870028 -0.885240
H 6.821145 -0.102210 0.510585
H 4.931613 -1.932266 2.127738
H 5.960153 -3.280069 1.565008
H 4.197182 -3.361445 1.410325
H -4.713656 -3.414402 2.411845
H -2.709446 -2.878093 1.072597
H -6.973315 -3.162459 1.423108
H -1.171611 5.142738 2.555257

4-c1 , delta G = 0.8817 kcal/mol, population = 6.82 %

O -0.091618 0.641092 1.269766
C -0.054277 1.911538 1.689775
C 1.162368 -3.211964 -0.065607
O -0.290496 -1.565311 -2.703378
C 0.552912 -0.951570 -2.061448
N 1.579664 -0.257846 -2.585695
O 0.940702 -4.333387 0.832137
C 2.530857 0.300032 -1.638347
O -1.637525 -3.504678 0.939481
O -4.360639 0.665729 -1.722241
C 2.100543 -0.385802 -0.299563
C 3.026900 -1.552883 -0.044675
C 0.323858 2.003866 3.135236
O -2.436446 2.342756 -0.758465
C 2.621764 -2.827338 -0.050859

O -5.014608 2.267488 1.407707
C 0.223654 -2.049685 0.263504
C 2.541675 1.840060 -1.640329
C 0.622897 -0.790471 -0.523492
C 4.480899 -1.182815 0.135479
C 3.589347 -3.983599 -0.037046
C -1.260235 -2.423318 0.074642
C -2.201093 -1.264046 0.389165
C -3.650924 -1.616548 0.056689
C -4.622038 -0.482342 0.384293
C -4.196490 0.826039 -0.314615
C -5.062793 1.987902 0.225448
C -2.731150 1.162712 -0.002058
C -0.331569 0.361698 -0.142997
C -1.796115 0.007181 -0.362867
O -0.275037 2.866910 0.967553
C 4.305087 3.440435 1.331984
C 3.302991 2.995143 0.474244
C 3.619203 2.390873 -0.742668
C -6.064270 -0.866503 0.053283
C -5.943172 2.726291 -0.739417
C 5.969682 2.703943 -0.240334
C 4.965762 2.259545 -1.092480
C 5.642160 3.290798 0.979745
H 0.896703 -3.632145 -1.040218
H 1.716435 -0.214245 -3.585005
C 1.249882 -4.098882 2.211112
H 3.529943 -0.030752 -1.916832
H -0.923944 -4.161711 0.885202
H -3.840114 1.357701 -2.152833
H 2.193999 0.325298 0.520171
H 0.437929 -3.569336 2.714172
H 0.153295 3.013382 3.497702
H 1.382243 1.754749 3.238545
H -0.244550 1.284504 3.723442
H -1.657373 2.758950 -0.353535
H 0.368131 -1.797159 1.316776
H 2.711494 2.165461 -2.669931
H 1.568860 2.229205 -1.341239
H 5.060919 -1.309677 -0.783127
H 4.950316 -1.799549 0.901514
H 4.580165 -0.139260 0.433696
H 3.787579 -4.351588 0.973456
H 3.177629 -4.822547 -0.600088
H -1.408579 -2.723488 -0.965839
H -2.123469 -1.070806 1.465125
H -3.946955 -2.505908 0.613293
H -3.724544 -1.862409 -1.007063
H -4.555727 -0.274722 1.455794
H -2.670649 1.379667 1.066761
H -0.078261 1.254538 -0.705573
H -1.904531 -0.175361 -1.433745
H 2.171002 -3.525622 2.324619
H 4.544499 -3.710407 -0.482103
H 4.039075 3.905911 2.272890
H 2.264351 3.131253 0.746163
H -6.185940 -1.035866 -1.017147
H -6.772743 -0.094200 0.359474
H -6.333025 -1.785924 0.575919
H -6.569178 2.027584 -1.295627
H -5.322072 3.243148 -1.475344
H -6.554138 3.448877 -0.202909
H 7.007903 2.592446 -0.527308
H 5.230631 1.801661 -2.038563
H 6.423513 3.635033 1.645465
H 1.376969 -5.078305 2.668446

4-c3 , delta G = 0.9833 kcal/mol, population = 5.74 %

O 0.117774 0.339870 1.714575
C -0.460665 1.273309 2.478959
C 2.942458 -2.015295 -0.207455
O 0.638249 -0.647666 -2.612169
C 1.346558 -0.033105 -1.822239
N 2.309614 0.833173 -2.175695
O 3.120976 -3.424890 0.010565
C 3.009431 1.519489 -1.108785
O 0.517707 -3.804324 0.398420
O -3.792233 -0.675896 -1.643453
C 2.492522 0.789708 0.174450
C 3.659786 0.095243 0.842303
C -0.003583 1.168262 3.902598
O -2.829281 1.315278 -0.065225
C 3.933085 -1.195031 0.612883
O -5.082825 -0.504636 1.687435
C 1.521381 -1.591020 0.193363
C 2.807109 3.046276 -1.145309
C 1.320104 -0.133041 -0.277638

C 4.497245 1.034511 1.665099
C 5.138409 -1.925231 1.132179
C 0.386022 -2.526283 -0.244420
C -0.982705 -1.983761 0.172834
C -2.112422 -2.823684 -0.423063
C -3.492642 -2.369875 0.050137
C -3.705955 -0.869100 -0.232580
C -4.996818 -0.401363 0.478785
C -2.547938 -0.030067 0.330791
C -0.033163 0.372360 0.259840
C -1.192300 -0.509523 -0.193140
O -1.242101 2.105616 2.063719
C -0.333057 4.528105 0.407612
C 0.975896 4.090594 0.235044
C 1.389231 3.518612 -0.969286
C -4.604442 -3.229473 -0.552137
C -6.106089 0.165720 -0.357598
C -0.858898 3.818741 -1.827685
C 0.453217 3.394444 -2.001543
C -1.256393 4.388708 -0.621551
H 3.087220 -1.834520 -1.279001
H 2.482937 1.027801 -3.151332
C 3.810173 -4.102913 -1.038359
H 4.080294 1.339212 -1.216935
H 1.463746 -4.023137 0.336809
H -3.622977 0.261558 -1.811422
H 2.105550 1.539713 0.862229
H 4.819280 -3.707081 -1.173615
H -0.049242 0.133459 4.240382
H -0.617105 1.802074 4.536496
H 1.038131 1.490412 3.962636
H -2.298160 1.895632 0.506899
H 1.495815 -1.573818 1.284854
H 3.437594 3.475865 -0.364662
H 3.203014 3.406074 -2.098993
H 3.880920 1.493969 2.443670
H 4.885251 1.854369 1.052005
H 5.345310 0.554775 2.145354
H 5.751887 -1.302779 1.776996
H 4.844090 -2.816248 1.687865
H 0.410218 -2.657020 -1.328852
H -1.020407 -2.071832 1.264723
H -1.972727 -3.870248 -0.149831
H -2.063625 -2.765367 -1.514558
H -3.529044 -2.469004 1.138276
H -2.585997 -0.115125 1.418913
H -0.192563 1.406475 -0.036753
H -1.215024 -0.423982 -1.279680
H 3.261766 -4.018695 -1.981361
H 5.770594 -2.265241 0.306470
H -0.634192 4.962515 1.351654
H 1.688633 4.198958 1.044130
H -4.433591 -4.280471 -0.313026
H -4.630184 -3.127320 -1.637616
H -5.587112 -2.957692 -0.161004
H -6.971469 0.372482 0.268300
H -6.367205 -0.521732 -1.162997
H -5.765477 1.089246 -0.832442
H -1.570937 3.707101 -2.635722
H 0.752971 2.969791 -2.950552
H -2.278562 4.718685 -0.485929
H 3.873619 -5.149776 -0.747973

4-c26 , delta G = 0.9996 kcal/mol, population = 5.59 %

O 0.095671 -0.637655 1.271058
C 0.054427 -1.900931 1.708304
C -1.138801 3.213782 -0.056483
O 0.295080 1.585088 -2.695246
C -0.545994 0.963218 -2.058418
N -1.572515 0.272395 -2.587551
O -0.914633 4.329488 0.848142
C -2.526232 -0.286934 -1.643376
O 1.656203 3.476999 0.974829
O 4.272139 -0.496702 -1.785065
C -2.096339 0.394043 -0.302013
C -3.015586 1.567442 -0.049681
C -0.325994 -1.972048 3.154726
O 2.403248 -2.374454 -0.759778
C -2.601073 2.839039 -0.051514
O 5.757133 -2.469144 -1.043223
C -0.211107 2.043147 0.273215
C -2.540802 -1.826867 -1.651152
C -0.616269 0.790695 -0.521870
C -4.473270 1.207971 0.122274
C -3.560594 4.002033 -0.040841
C 1.278206 2.404495 0.099311
C 2.202515 1.232200 0.415230

C 3.666369 1.583355 0.143629
C 4.614550 0.418010 0.436852
C 4.183319 -0.811637 -0.404402
C 5.087619 -2.022674 -0.129000
C 2.733459 -1.184818 -0.048706
C 0.331885 -0.368268 -0.145771
C 1.795872 -0.018880 -0.373241
O 0.271243 -2.868241 1.000771
C -4.291343 -3.438155 1.322514
C -3.293112 -2.989732 0.461931
C -3.614843 -2.379368 -0.750532
C 6.072308 0.798685 0.191308
C 5.108382 -2.630345 1.241413
C -5.963081 -2.691918 -0.237600
C -4.962987 -2.244544 -1.092746
C -5.630055 -3.285304 0.977805
H -0.864545 3.636624 -1.027430
H -1.710943 0.237788 -3.586964
C -1.235874 4.091368 2.223699
H -3.524217 0.046942 -1.922124
H 0.948485 4.140086 0.917745
H 4.877787 -1.156783 -2.163071
H -2.196747 -0.317353 0.516726
H -1.358340 5.069716 2.684605
H -0.126654 -2.967678 3.540483
H -1.392958 -1.756439 3.246155
H 0.214413 -1.221567 3.729875
H 1.639125 -2.770805 -0.308679
H -0.365152 1.787301 1.324221
H -2.716318 -2.147367 -2.681347
H -1.567472 -2.219742 -1.359085
H -4.582039 0.164102 0.416210
H -5.048026 1.342643 -0.798540
H -4.941608 1.825539 0.888332
H -3.760912 4.370445 0.969109
H -3.140600 4.838635 -0.601280
H 1.439677 2.709427 -0.937975
H 2.090778 1.015325 1.483654
H 3.953598 2.438452 0.755339
H 3.779605 1.880800 -0.902811
H 4.495391 0.150713 1.490733
H 2.699869 -1.379141 1.028316
H 0.071124 -1.262248 -0.702862
H 1.901625 0.184102 -1.439829
H -0.432068 3.552884 2.730383
H -4.515597 3.735921 -0.490487
H -4.021115 -3.908688 2.259710
H -2.253149 -3.127939 0.727879
H 6.758689 0.001869 0.486235
H 6.330208 1.688293 0.768032
H 6.239772 1.016558 -0.864301
H 4.204713 -3.232693 1.366354
H 5.107709 -1.874153 2.025474
H 5.977083 -3.276792 1.343604
H -7.002620 -2.577895 -0.518768
H -5.232128 -1.782024 -2.035355
H -6.408423 -3.632152 1.645670
H -2.162425 3.525146 2.327843

4-c4 , delta G = 1.2858 kcal/mol, population = 3.44 %

O 0.435768 0.321864 1.883455
C 0.467626 -0.610563 2.845910
C -1.102941 3.300815 -0.796487
O -0.585140 0.409958 -2.556114
C -1.178287 0.299677 -1.490409
N -2.303812 -0.410453 -1.290125
O -0.607658 4.658911 -0.651023
C -2.912521 -0.291571 0.026300
O 1.872347 3.570618 -0.826248
O 3.605781 -1.732159 -1.706452
C -2.102341 0.887656 0.653138
C -2.906419 2.158053 0.497302
C 0.536597 0.007122 4.207922
O 2.021559 -2.449281 0.390183
C -2.500230 3.200061 -0.235470
O 5.138262 -1.796774 1.524063
C -0.125905 2.290149 -0.192364
C -2.898667 -1.597373 0.840218
C -0.764047 0.894855 -0.123123
C -4.260597 2.139566 1.168612
C -3.406686 4.360817 -0.559616
C 1.235155 2.287879 -0.916029
C 2.209800 1.272372 -0.326217
C 3.499583 1.192852 -1.143423
C 4.502134 0.194743 -0.563541
C 3.864721 -1.201567 -0.408517
C 4.828874 -2.109155 0.390503

C 2.553138 -1.118708 0.386065
C 0.242746 -0.095595 0.499531
C 1.576310 -0.118435 -0.234271
O 0.428628 -1.806879 2.625697
C -3.482323 -4.623016 -1.389151
C -2.847243 -3.619326 -0.663648
C -3.585131 -2.719896 0.108458
C 5.787331 0.147058 -1.390637
C 5.351251 -3.350189 -0.271451
C -5.614027 -3.850721 -0.583080
C -4.975837 -2.848150 0.139350
C -4.868331 -4.741182 -1.351325
H -1.131308 3.174837 -1.882909
H -2.737823 -0.919632 -2.045569
C -0.502115 5.145310 0.691736
H -3.954947 -0.005554 -0.104288
H 1.184630 4.233805 -1.003022
H 2.970495 -2.453422 -1.599691
H -1.938923 0.695189 1.713643
H -0.446450 6.229934 0.621137
H 1.291606 0.792223 4.229165
H 0.758710 -0.755361 4.948834
H -0.426685 0.467645 4.436978
H 1.385652 -2.507823 1.122626
H 0.054954 2.581986 0.845220
H -1.877657 -1.887239 1.083812
H -3.407345 -1.393599 1.784995
H -5.071722 1.936133 0.463672
H -4.478119 3.096837 1.642284
H -4.301370 1.369399 1.940167
H -3.138216 4.788983 -1.526111
H -4.450335 4.053022 -0.600388
H 1.063728 2.040926 -1.967317
H 2.454317 1.612293 0.686705
H 3.966464 2.177042 -1.186527
H 3.255139 0.907005 -2.170934
H 4.754705 0.513901 0.451140
H 2.810135 -0.816023 1.403663
H -0.182334 -1.093022 0.510662
H 1.357020 -0.465632 -1.245958
H 0.400112 4.770597 1.179927
H -3.328826 5.167594 0.174751
H -2.894454 -5.313576 -1.981040
H -1.767573 -3.532529 -0.694918
H 6.539008 -0.503343 -0.938625
H 6.218470 1.146648 -1.465824
H 5.586932 -0.213920 -2.400053
H 5.794226 -3.110462 -1.238808
H 4.520310 -4.032367 -0.468693
H 6.078096 -3.835868 0.375865
H -6.692688 -3.939306 -0.544262
H -5.562140 -2.159959 0.737602
H -5.363931 -5.522978 -1.912992
H -1.371265 4.867491 1.289739

**Table S24.** Geometry data of conformers of compound **5**.

78

5-c5.out delta G = 0.0000 kcal/mol

conformer 1

O -0.256338 -0.775124 -1.256591
C 0.110302 -2.055013 -1.464311
C 1.135406 3.223778 -0.475495
O -0.265672 1.907463 2.379558
C 0.593050 1.265362 1.767153
N 1.684701 0.734658 2.322707
C 0.216293 -2.346764 -2.931819
O 0.781260 4.317395 -1.334570
C 2.623940 0.088293 1.418221
O -1.603631 3.305952 0.504352
O -4.436867 -0.087076 1.653193
C 2.055435 0.468153 0.012749
C 2.935946 1.524746 -0.624653
O 0.345519 -2.842450 -0.576110
O -2.491541 -2.023023 1.213064
C 2.553903 2.798146 -0.787071
O -5.959029 -2.095015 1.125835
C 0.175776 2.050254 -0.665587
C 2.759937 -1.420143 1.687030
C 0.585498 0.900851 0.270016
C 4.309108 1.016781 -0.995200
C 3.470452 3.897961 -1.247770
C -1.304737 2.463264 -0.608901
C -2.232718 1.243162 -0.719747
C -3.702705 1.632417 -0.594824
C -4.633569 0.427470 -0.732859
C -4.269330 -0.624498 0.345196
C -5.174125 -1.860505 0.224470

C -2.795410 -1.052743 0.212293
C -0.400978 -0.280086 0.102559
C -1.854973 0.151585 0.289786
C 4.772877 -3.282963 -0.958308
C 3.699312 -2.803776 -0.211636
C 3.913607 -2.010136 0.915369
C -6.103053 0.837677 -0.663002
C -5.058428 -2.742960 -0.980821
C 6.302310 -2.195830 0.546113
C 5.228557 -1.718991 1.288242
C 6.077176 -2.976252 -0.585571
H 1.081604 3.580629 0.560005
H 1.862655 0.834018 3.312055
H 1.097624 -1.838173 -3.328705
H -0.654550 -1.964024 -3.462705
H 0.317984 -3.416975 -3.088285
H -0.007691 4.722559 -0.954257
H 3.602506 0.545235 1.560279
H -1.245600 2.871648 1.306383
H -5.191613 -0.566722 2.037666
H 2.082801 -0.415182 -0.622963
H -2.776368 -1.639792 2.055598
H 0.338133 1.674944 -1.679451
H 2.923206 -1.556744 2.759165
H 1.830860 -1.927906 1.428372
H 4.756029 1.596941 -1.799288
H 4.249984 -0.022452 -1.323943
H 5.002027 1.033610 -0.149282
H 3.374398 4.760244 -0.583050
H 4.513129 3.591195 -1.251818
H 3.205229 4.249454 -2.247085
H -1.495209 3.083794 -1.486450
H -2.075815 0.832512 -1.722565
H -3.951847 2.363041 -1.368971
H -3.876188 2.114961 0.367609
H -4.444918 -0.033266 -1.707283
H -2.662397 -1.555931 -0.745088
H -0.158887 -1.090558 0.783632
H -1.941621 0.558878 1.298885
H 4.587944 -3.896080 -1.831825
H 2.686571 -3.051299 -0.500849
H -6.774236 -0.002553 -0.852306
H -6.313220 1.604288 -1.410253
H -6.341657 1.248164 0.319467
H -5.958729 -3.344170 -1.085934
H -4.206284 -3.412378 -0.832939
H -4.873814 -2.175578 -1.891814
H 7.314638 -1.959114 0.849548
H 5.413144 -1.110093 2.166108
H 6.912372 -3.346689 -1.166672
78
5-c14.out delta G = 0.0370 kcal/mol
conformer 2
O -0.036315 -0.751230 -1.231924
C 0.029399 -2.071664 -1.440083
C 1.121363 3.308491 -0.461286
O -0.293813 1.971419 2.373054
C 0.602713 1.358774 1.787198
N 1.711507 0.892565 2.368485
C 0.452207 -2.391609 -2.839950
O 0.758241 4.372163 -1.353826
C 2.662930 0.220002 1.496436
O -1.637288 3.300971 0.453464
O -4.308046 -0.120499 1.610769
C 2.125556 0.585144 0.076482
C 2.987958 1.675229 -0.527502
O -0.195419 -2.902507 -0.579002
O -2.362812 -2.134796 1.046883
C 2.564077 2.931461 -0.714646
O -5.733566 -2.231389 1.214166
C 0.210837 2.098509 -0.662826
C 2.766556 -1.283280 1.810502
C 0.638339 0.973134 0.293685
C 4.396313 1.226387 -0.842121
C 3.459632 4.058299 -1.150260
C -1.284416 2.457457 -0.642539
C -2.165642 1.203482 -0.761107
C -3.650934 1.548204 -0.658629
C -4.555883 0.319071 -0.766167
C -4.149255 -0.704082 0.327290
C -5.022152 -1.966557 0.261775
C -2.679827 -1.104319 0.114632
C -0.295130 -0.245498 0.113294
C -1.770715 0.119804 0.252491
C 4.330763 -3.414835 -0.928194
C 3.390734 -2.831212 -0.083316
C 3.786542 -1.972106 0.942220
C -6.031928 0.698148 -0.676498

C -4.964088 -2.840713 -0.954629
C 6.092344 -2.305328 0.276979
C 5.150139 -1.723875 1.117034
C 5.684852 -3.148860 -0.753830
H 1.017597 3.682387 0.564172
H 1.862586 1.004558 3.360981
H 0.161291 -3.408266 -3.089867
H 1.540688 -2.311998 -2.895677
H 0.027341 -1.681469 -3.546682
H -0.052957 4.761057 -1.004887
H 3.645815 0.664019 1.646949
H -1.288384 2.885321 1.268998
H -4.930706 -0.698889 2.083295
H 2.196816 -0.290237 -0.566246
H -1.585284 -2.600162 0.696308
H 0.411204 1.721284 -1.669151
H 3.041936 -1.383888 2.863537
H 1.790951 -1.755259 1.691798
H 4.411373 0.160947 -1.075383
H 5.078254 1.371661 0.000738
H 4.810931 1.761170 -1.694408
H 4.511874 3.789664 -1.102433
H 3.228191 4.384837 -2.166403
H 3.301720 4.926071 -0.504888
H -1.477559 3.061332 -1.531088
H -1.979592 0.797392 -1.761769
H -3.913428 2.252135 -1.452956
H -3.842149 2.052046 0.289091
H -4.373542 -0.151998 -1.736359
H -2.596539 -1.493723 -0.904992
H -0.031262 -1.028024 0.819223
H -1.905734 0.500613 1.265589
H 4.003713 -4.077269 -1.720005
H 2.339649 -3.055708 -0.212227
H -6.686217 -0.159857 -0.845951
H -6.272933 1.451617 -1.428051
H -6.262493 1.111378 0.306326
H -4.983125 -2.259558 -1.876232
H -5.792051 -3.545727 -0.939933
H -4.023938 -3.397816 -0.938975
H 7.145234 -2.100199 0.425447
H 5.477166 -1.065812 1.913780
H 6.418279 -3.599913 -1.410226

78

5-c1.out delta G = 0.2454 kcal/mol

conformer 3

O -0.047166 -0.770385 -1.206708
C 0.024379 -2.096651 -1.380653
C 1.117000 3.307084 -0.485748
O -0.275953 1.976281 2.378265
C 0.618855 1.369775 1.782990
N 1.737719 0.913933 2.352520
C 0.428566 -2.451613 -2.777301
O 0.739941 4.369399 -1.373616
C 2.680965 0.239110 1.473495
O -1.621866 3.318465 0.473375
O -4.390925 -0.318781 1.629790
C 2.123031 0.588219 0.057439
C 2.977526 1.669304 -0.573422
O -0.182962 -2.903847 -0.493288
O -2.377725 -2.108865 1.100381
C 2.553718 2.924986 -0.764409
O -4.858512 -2.557280 -1.120731
C 0.199333 2.098681 -0.666023
C 2.795880 -1.260877 1.799026
C 0.638236 0.978389 0.290654
C 4.377749 1.211366 -0.909403
C 3.442760 4.046318 -1.226566
C -1.293357 2.464635 -0.622352
C -2.185295 1.215781 -0.719343
C -3.661676 1.564760 -0.544912
C -4.578457 0.349647 -0.682000
C -4.155638 -0.765820 0.296233
C -4.962566 -2.046284 -0.022448
C -2.670646 -1.109645 0.116757
C -0.296779 -0.241364 0.129658
C -1.773753 0.120137 0.270989
C 4.353151 -3.412323 -0.928356
C 3.415948 -2.826636 -0.081739
C 3.813607 -1.951095 0.929020
C -6.047721 0.736197 -0.509027
C -5.851769 -2.612816 1.044899
C 6.115925 -2.272179 0.246035
C 5.176532 -1.688706 1.087754
C 5.706325 -3.132037 -0.770371
H 1.033344 3.683731 0.540620
H 1.899188 1.029934 3.342945
H -0.019981 -1.769136 -3.496627

H 0.148617 -3.479272 -2.992038
H 1.514439 -2.356854 -2.855149
H -0.064610 4.759794 -1.011110
H 3.663304 0.689658 1.607143
H -1.269314 2.901609 1.286665
H -3.885637 -0.893342 2.220864
H 2.184701 -0.295066 -0.575301
H -1.589390 -2.588783 0.797792
H 0.381858 1.714565 -1.673282
H 3.079519 -1.351499 2.850814
H 1.822132 -1.739428 1.690925
H 4.380594 0.147404 -1.150280
H 5.071438 1.344086 -0.074119
H 4.786354 1.748371 -1.763008
H 3.184748 4.372831 -2.236199
H 3.307313 4.915736 -0.578149
H 4.494524 3.772587 -1.207220
H -1.499023 3.063746 -1.511135
H -2.040977 0.818927 -1.730616
H -3.950564 2.310015 -1.291007
H -3.808730 2.023505 0.433474
H -4.447530 -0.074471 -1.681116
H -2.563216 -1.531127 -0.884945
H -0.028728 -1.012579 0.846503
H -1.905209 0.492169 1.288757
H 4.024562 -4.087497 -1.708680
H 2.365881 -3.062553 -0.197498
H -6.314651 1.515383 -1.224874
H -6.233512 1.118728 0.495197
H -6.714541 -0.111409 -0.680039
H -6.523531 -1.845760 1.431921
H -5.239145 -2.946152 1.886608
H -6.415311 -3.453827 0.646953
H 7.168276 -2.055839 0.381867
H 5.505233 -1.017914 1.873117
H 6.437526 -3.584570 -1.428226
78
5-c7.out delta G = 0.6470 kcal/mol
conformer 4
O -0.048281 -0.741982 -1.205951
C 0.022579 -2.067891 -1.382993
C 1.095334 3.343088 -0.564120
O -0.244226 1.991705 2.405695
C 0.632964 1.376344 1.794558
N 1.738045 0.877621 2.355572
C 0.411536 -2.418982 -2.784997
O 0.728893 4.313719 -1.560401
C 2.665814 0.200754 1.463464
O -1.643645 3.288448 0.537236
O -4.395619 -0.307735 1.624001
C 2.121405 0.607637 0.057078
C 2.986444 1.714524 -0.504575
O -0.175302 -2.876621 -0.494955
O -2.369824 -2.090313 1.107545
C 2.554826 2.966548 -0.696239
O -4.839905 -2.564242 -1.115583
C 0.185809 2.122444 -0.662840
C 2.745257 -1.309672 1.749323
C 0.639756 1.005409 0.295952
C 4.417030 1.306103 -0.774495
C 3.477116 4.096789 -1.074811
C -1.309310 2.487953 -0.591719
C -2.191242 1.234154 -0.718333
C -3.671837 1.569993 -0.554277
C -4.578996 0.347920 -0.692048
C -4.153429 -0.760125 0.293468
C -4.952216 -2.046560 -0.021215
C -2.666162 -1.095885 0.119706
C -0.298121 -0.215180 0.131609
C -1.776820 0.139543 0.271650
C 6.097829 -2.324516 0.268494
C 5.140942 -1.742724 1.091398
C 3.780480 -1.993340 0.894456
C -6.051970 0.724549 -0.528613
C -5.844715 -2.610199 1.044940
C 4.358557 -3.439581 -0.963248
C 3.403686 -2.855085 -0.135589
C 5.709032 -3.171369 -0.766957
H 0.919583 3.864343 0.380119
H 1.902362 0.972032 3.347701
H 0.171034 -3.459419 -2.984683
H 1.489553 -2.275660 -2.890959
H -0.084322 -1.763572 -3.498834
H 0.886963 3.923219 -2.429849
H 3.658665 0.621273 1.613180
H -1.262811 2.862227 1.332503
H -3.889784 -0.876827 2.219826
H 2.185437 -0.247863 -0.612969

H -1.581676 -2.570602 0.805717
H 0.338428 1.706801 -1.664750
H 2.994447 -1.432670 2.806637
H 1.769733 -1.771874 1.598017
H 4.488515 0.228186 -0.921175
H 5.086612 1.558612 0.052957
H 4.807041 1.793231 -1.667270
H 3.460195 4.299643 -2.149272
H 3.161335 5.016959 -0.579818
H 4.506987 3.892031 -0.789356
H -1.517991 3.120506 -1.455031
H -2.034158 0.845474 -1.730897
H -3.961832 2.310416 -1.304873
H -3.828024 2.030857 0.421559
H -4.439773 -0.079942 -1.688500
H -2.554275 -1.520465 -0.880093
H -0.030475 -0.987563 0.846561
H -1.911168 0.511830 1.289299
H 7.147772 -2.117600 0.434323
H 5.454310 -1.083119 1.892278
H -6.712118 -0.128152 -0.700381
H -6.320362 1.499548 -1.248463
H -6.245868 1.109281 0.473255
H -6.403599 -3.455245 0.648971
H -6.520693 -1.843240 1.424697
H -5.234996 -2.937007 1.891328
H 4.045511 -4.104649 -1.758495
H 2.355798 -3.083441 -0.279998
H 6.454084 -3.622992 -1.409707

78

5-c4.out delta G = 0.7543 kcal/mol

conformer 5

O -0.385701 -0.288494 -1.946129
C -0.349380 -1.515530 -2.488645
C 0.986211 3.515519 -0.347626
O 0.479326 1.289494 2.209536
C 1.133973 0.930303 1.227498
N 2.330900 0.338125 1.279784
C -0.375888 -1.455363 -3.984058
O 0.407183 4.761196 -0.764845
C 2.954797 0.037278 -0.002181
O -1.371431 3.011323 1.261373
O -3.795346 -0.930341 1.928972
C 2.057406 0.834292 -0.997335
C 2.754706 2.119750 -1.391695
O -0.287691 -2.538282 -1.832730
O -2.009305 -2.353108 0.408488
C 2.309348 3.336254 -1.055609
O -4.997040 -2.328243 -1.136168
C 0.042679 2.353050 -0.652731
C 3.046973 -1.469409 -0.295205
C 0.711586 1.029418 -0.252727
C 4.045134 1.901170 -2.150023
C 3.081539 4.600756 -1.312492
C -1.373949 2.575858 -0.098065
C -2.276875 1.357199 -0.347492
C -3.652657 1.536825 0.290618
C -4.582713 0.349829 0.040651
C -3.926530 -0.965151 0.509535
C -4.801055 -2.155868 0.051254
C -2.546059 -1.141909 -0.137100
C -0.252361 -0.154062 -0.501268
C -1.628883 0.053615 0.127626
C 3.852274 -3.454601 2.847060
C 3.144618 -2.821956 1.829120
C 3.814302 -2.207386 0.769332
C -5.951553 0.568981 0.686167
C -5.380347 -3.058761 1.100340
C 5.921225 -2.874254 1.763608
C 5.210754 -2.241379 0.749331
C 5.243157 -3.481928 2.817242
H 1.160345 3.572028 0.733326
H 2.766550 0.119790 2.164225
H -1.163407 -0.781723 -4.320487
H -0.527064 -2.450861 -4.391169
H 0.575862 -1.056086 -4.340393
H -0.264611 4.990080 -0.111583
H 3.967029 0.438765 0.006644
H -0.793312 2.402926 1.766668
H -3.154396 -1.610267 2.177361
H 1.909781 0.238615 -1.897214
H -1.326644 -2.670725 -0.205113
H -0.064800 2.312621 -1.740451
H 2.047747 -1.893664 -0.392794
H 3.538984 -1.579999 -1.263928
H 4.922095 1.944711 -1.497721
H 4.185462 2.645661 -2.932563
H 4.047993 0.919315 -2.626679

H 2.615671 5.200788 -2.097315
H 3.087956 5.221336 -0.413022
H 4.112728 4.402360 -1.594221
H -1.808045 3.407078 -0.656670
H -2.405120 1.288865 -1.433758
H -4.121693 2.441732 -0.105420
H -3.534331 1.685654 1.364497
H -4.722309 0.237107 -1.037821
H -2.713025 -1.258752 -1.210006
H 0.187928 -1.072005 -0.123915
H -1.472443 0.107420 1.207069
H 3.317316 -3.928317 3.660762
H 2.061375 -2.805859 1.854502
H -6.648227 -0.237924 0.449753
H -6.390045 1.499713 0.322445
H -5.863354 0.636452 1.771131
H -5.919988 -2.478033 1.849341
H -4.569732 -3.570512 1.625578
H -6.035593 -3.791975 0.635278
H 7.003458 -2.895888 1.730498
H 5.744464 -1.772754 -0.069650
H 5.795191 -3.976213 3.606657
78
5-c13.out delta G = 1.5600 kcal/mol
conformer 6
O -0.368709 -0.264667 -1.924548
C -0.323185 -1.490836 -2.468244
C 0.941465 3.574365 -0.396077
O 0.500297 1.306169 2.243830
C 1.142371 0.946121 1.254410
N 2.327257 0.328836 1.297633
C -0.345283 -1.429025 -3.963695
O 0.337363 4.766134 -0.928994
C 2.944653 0.033908 0.012131
O -1.385842 2.968070 1.314306
O -3.810436 -0.933919 1.915833
C 2.060340 0.863053 -0.968595
C 2.776287 2.150522 -1.313052
O -0.258006 -2.513992 -1.813409
O -1.994454 -2.341516 0.417138
C 2.319303 3.364057 -0.983721
O -4.963293 -2.339238 -1.164642
C 0.028603 2.374324 -0.631035
C 3.017426 -1.469672 -0.301958
C 0.714779 1.058615 -0.224062
C 4.101543 1.950280 -2.014142
C 3.127477 4.620385 -1.181483
C -1.384930 2.586728 -0.057990
C -2.281076 1.369299 -0.338669
C -3.666540 1.535327 0.281643
C -4.584022 0.341499 0.018340
C -3.923821 -0.968642 0.494894
C -4.783379 -2.165841 0.025216
C -2.534432 -1.134487 -0.135130
C -0.246045 -0.129773 -0.478410
C -1.629513 0.068240 0.138623
C 3.807230 -3.496524 2.817523
C 3.104531 -2.844542 1.808420
C 3.778694 -2.228932 0.752067
C -5.962901 0.549145 0.646143
C -5.368686 -3.073819 1.066573
C 5.879932 -2.934099 1.731775
C 5.174426 -2.281832 0.726346
C 5.197513 -3.542529 2.782130
H 1.014939 3.780609 0.674433
H 2.762044 0.097516 2.179102
H -0.481964 -2.425926 -4.372459
H 0.601775 -1.016254 -4.317008
H -1.141218 -0.765973 -4.301522
H 0.267486 4.663110 -1.887075
H 3.963283 0.417949 0.026148
H -0.788877 2.360995 1.798583
H -3.167277 -1.609021 2.171521
H 1.912001 0.293915 -1.885359
H -1.305782 -2.656568 -0.190900
H -0.102635 2.294235 -1.715908
H 2.014768 -1.883587 -0.404219
H 3.507700 -1.572500 -1.272432
H 4.142115 0.968673 -2.489157
H 4.949270 2.011566 -1.325788
H 4.259726 2.698914 -2.789650
H 4.192932 4.411754 -1.252871
H 2.828911 5.161484 -2.083482
H 2.968351 5.301941 -0.343381
H -1.814181 3.438069 -0.587328
H -2.393469 1.308741 -1.427178
H -4.137748 2.437022 -0.119339
H -3.562636 1.683661 1.356999

H -4.708574 0.228299 -1.061973
H -2.688689 -1.253388 -1.209633
H 0.195521 -1.045549 -0.098366
H -1.483260 0.121829 1.219799
H 3.268859 -3.970617 3.628746
H 2.021748 -2.813654 1.838316
H -6.650165 -0.263031 0.400320
H -6.403998 1.476737 0.277570
H -5.888965 0.616490 1.732179
H -5.921005 -2.497707 1.809842
H -4.560631 -3.580463 1.600641
H -6.013257 -3.811150 0.593163
H 6.961639 -2.970185 1.694315
H 5.711431 -1.812634 -0.090149
H 5.745637 -4.051810 3.564705
78
5-c16.out delta G = 2.1059 kcal/mol
conformer 7
O -0.423664 -0.309772 -1.946145
C -0.406125 -1.536798 -2.490566
C 0.996848 3.498252 -0.418595
O 0.531289 1.313368 2.172011
C 1.165880 0.932256 1.184745
N 2.358294 0.330750 1.224735
C -0.470616 -1.472992 -3.984835
O 0.416741 4.737604 -0.853577
C 2.958757 0.011191 -0.064225
O -1.350517 3.003884 1.228793
O -3.764527 -0.863199 2.055571
C 2.049867 0.803212 -1.052871
C 2.747485 2.082096 -1.467889
O -0.331227 -2.560358 -1.838357
O -1.976929 -2.354222 0.466033
C 2.312130 3.303818 -1.137468
O -5.021150 -2.417233 -0.846125
C 0.047672 2.333714 -0.695792
C 3.037941 -1.499014 -0.342100
C 0.717973 1.012533 -0.288724
C 4.026819 1.851568 -2.241256
C 3.088497 4.561492 -1.415354
C -1.362488 2.564811 -0.129556
C -2.266052 1.343870 -0.361382
C -3.653788 1.549290 0.243007
C -4.573277 0.339443 0.058800
C -3.904372 -0.931243 0.636701
C -4.769346 -2.162501 0.315626
C -2.542103 -1.149534 -0.048281
C -0.255329 -0.169922 -0.506280
C -1.616956 0.053575 0.150893
C 5.946879 -2.895562 1.672892
C 5.218671 -2.271056 0.666057
C 3.823186 -2.228418 0.715283
C -5.955068 0.591828 0.661716
C -5.269396 -3.002193 1.451296
C 3.898060 -3.449930 2.807104
C 3.172546 -2.825736 1.796535
C 5.287828 -3.485985 2.748189
H 1.181844 3.573927 0.659338
H 2.809327 0.123825 2.104209
H 0.473324 -1.076598 -4.364446
H -1.263777 -0.795584 -4.299704
H -0.635981 -2.467029 -4.390004
H -0.242288 4.986753 -0.194933
H 3.973361 0.406504 -0.076683
H -0.751106 2.411189 1.728685
H -3.407175 -0.001053 2.296564
H 1.885123 0.200585 -1.945231
H -1.348263 -2.676728 -0.199808
H -0.073211 2.280369 -1.781456
H 2.035286 -1.920011 -0.415601
H 3.510539 -1.622353 -1.318913
H 4.157506 2.586964 -3.033993
H 4.020429 0.864345 -2.706545
H 4.912892 1.900319 -1.601843
H 3.097361 5.196823 -0.526362
H 4.118877 4.354735 -1.694207
H 2.624879 5.150156 -2.210114
H -1.800286 3.393212 -0.689425
H -2.381217 1.245526 -1.445978
H -4.124170 2.422700 -0.215676
H -3.554509 1.795296 1.305043
H -4.689446 0.153277 -1.011657
H -2.758465 -1.269606 -1.113487
H 0.186575 -1.088037 -0.131823
H -1.423275 0.141359 1.223184
H 7.028021 -2.924222 1.617094
H 5.737491 -1.815953 -0.169951
H -6.392576 1.492844 0.228945

H -5.893158 0.732437 1.741999
H -6.639418 -0.235710 0.465692
H -5.859184 -2.391609 2.137156
H -4.420393 -3.378894 2.024474
H -5.865058 -3.826460 1.065289
H 3.377752 -3.910344 3.637754
H 2.090209 -2.802871 1.844417
H 5.853666 -3.973805 3.531834
78
5-c10.out delta G = 2.5596 kcal/mol conformer 8
O -0.038019 -0.759794 -1.230072
C 0.035071 -2.081553 -1.436499
C 1.112106 3.305759 -0.460846
O -0.275369 1.952143 2.381535
C 0.620225 1.345645 1.786846
N 1.734712 0.881994 2.357794
C 0.439202 -2.400513 -2.841930
O 0.739793 4.367434 -1.351929
C 2.681155 0.214462 1.476131
O -1.643294 3.276641 0.480170
O -4.402164 -0.302356 1.654949
C 2.131708 0.583389 0.061692
C 2.985238 1.679698 -0.543313
O -0.168953 -2.910947 -0.569776
O -2.333498 -2.141313 1.082400
C 2.554892 2.934764 -0.723001
O -4.895818 -2.615665 -0.956180
C 0.205308 2.092066 -0.657697
C 2.790800 -1.289507 1.784233
C 0.645013 0.965342 0.291952
C 4.393789 1.238734 -0.868355
C 3.443756 4.066367 -1.159948
C -1.291096 2.444578 -0.624816
C -2.166777 1.186685 -0.743684
C -3.652506 1.523351 -0.632082
C -4.556385 0.292484 -0.734591
C -4.147488 -0.758743 0.326667
C -4.959177 -2.048628 0.117326
C -2.664292 -1.126731 0.136042
C -0.285329 -0.256391 0.115951
C -1.762594 0.103330 0.265276
C 4.360676 -3.412614 -0.958170
C 3.419269 -2.834049 -0.111297
C 3.812466 -1.971466 0.912298
C -6.033193 0.672996 -0.635152
C -5.800556 -2.556459 1.248320
C 6.118482 -2.290736 0.240983
C 5.174986 -1.714445 1.083099
C 5.713506 -3.137934 -0.787833
H 1.012688 3.679564 0.565129
H 1.892896 0.990646 3.349584
H 0.143238 -3.416499 -3.088770
H 1.527164 -2.323380 -2.910983
H 0.006676 -1.689272 -3.542751
H -0.066578 4.759575 -0.995801
H 3.663866 0.660672 1.620671
H -1.274987 2.864696 1.289443
H -4.073125 0.598922 1.747061
H 2.201506 -0.288850 -0.585477
H -1.570199 -2.620191 0.720649
H 0.398874 1.718889 -1.666873
H 3.067904 -1.393149 2.836550
H 1.817063 -1.764952 1.664787
H 4.414296 0.172282 -1.096323
H 5.082349 1.393696 -0.032668
H 4.796518 1.772047 -1.727299
H 3.206506 4.394080 -2.174357
H 3.285262 4.932092 -0.511979
H 4.497264 3.801781 -1.117460
H -1.493764 3.052965 -1.507991
H -1.986844 0.782528 -1.745478
H -3.925977 2.227805 -1.421581
H -3.834118 2.054403 0.307692
H -4.384994 -0.182266 -1.703883
H -2.585930 -1.529933 -0.877319
H -0.016579 -1.038353 0.820437
H -1.873787 0.487859 1.282180
H 4.035592 -4.077948 -1.748347
H 2.369349 -3.065595 -0.237133
H -6.684262 -0.189685 -0.788322
H -6.280129 1.413366 -1.397740
H -6.264958 1.103413 0.340270
H -6.303312 -3.473783 0.950045
H -6.530336 -1.798978 1.539917
H -5.169416 -2.730712 2.121362
H 7.170470 -2.078881 0.386395
H 5.500266 -1.053852 1.878450

H 6.447969 -3.585031 -1.445772
78
5-c6.out delta G = 2.6117 kcal/mol
conformer 9
O -0.228639 -0.783095 -1.256913
C 0.137421 -2.062780 -1.470640
C 1.115975 3.228638 -0.453076
O -0.262891 1.878707 2.393555
C 0.601075 1.246942 1.777015
N 1.695691 0.719195 2.328738
C 0.247838 -2.346104 -2.939236
O 0.752919 4.318714 -1.313078
C 2.639486 0.086825 1.418563
O -1.625941 3.263809 0.529106
O -4.509742 -0.266188 1.675340
C 2.069523 0.478072 0.016952
C 2.939554 1.553159 -0.603360
O 0.368960 -2.854406 -0.585431
O -2.454167 -2.045312 1.246080
C 2.541484 2.822552 -0.757325
O -4.970457 -2.550793 -0.973237
C 0.172285 2.043519 -0.650912
C 2.785190 -1.423257 1.672293
C 0.595540 0.892905 0.277600
C 4.324373 1.071019 -0.966868
C 3.448375 3.936795 -1.202178
C -1.312980 2.437710 -0.592872
C -2.225910 1.206890 -0.713382
C -3.699506 1.580953 -0.589196
C -4.623094 0.368471 -0.716424
C -4.251370 -0.701417 0.333516
C -5.087058 -1.970621 0.088100
C -2.763729 -1.097659 0.222981
C -0.378263 -0.297773 0.103851
C -1.837368 0.117181 0.293396
C 6.328946 -2.160372 0.509966
C 5.254623 -1.697532 1.260026
C 3.940543 -1.998188 0.891635
C -6.096718 0.770108 -0.643599
C -6.018389 -2.438516 1.165183
C 4.802112 -3.252182 -0.993711
C 3.727919 -2.786952 -0.239112
C 6.105386 -2.936125 -0.625246
H 1.051690 3.583351 0.582562
H 1.873168 0.812096 3.318823
H 0.359167 -3.414543 -3.100902
H 1.124283 -1.826982 -3.333213
H -0.626563 -1.969038 -3.468417
H -0.037425 4.720848 -0.932754
H 3.615115 0.548140 1.565863
H -1.250082 2.834108 1.325869
H -4.271643 0.664834 1.758138
H 2.107001 -0.396236 -0.630585
H -2.782697 -1.667733 2.075091
H 0.339989 1.677246 -1.667209
H 2.952282 -1.569185 2.742639
H 1.858972 -1.934892 1.411253
H 4.752676 1.644192 -1.786260
H 4.292343 0.023469 -1.271040
H 5.019432 1.127871 -0.124414
H 3.333194 4.794904 -0.535132
H 4.494967 3.643502 -1.195470
H 3.190296 4.288865 -2.203200
H -1.510712 3.063819 -1.464703
H -2.065644 0.801367 -1.717056
H -3.960402 2.304287 -1.365676
H -3.866802 2.099736 0.360512
H -4.439177 -0.100681 -1.686554
H -2.630800 -1.618831 -0.724221
H -0.129289 -1.108664 0.781918
H -1.913954 0.525880 1.304399
H 7.340564 -1.916412 0.810002
H 5.438104 -1.092146 2.140539
H -6.315450 1.520104 -1.405062
H -6.344181 1.197842 0.329449
H -6.758326 -0.080335 -0.817540
H -5.450729 -2.657863 2.071729
H -6.551694 -3.325148 0.829812
H -6.720123 -1.644471 1.425701
H 4.618397 -3.861831 -1.869885
H 2.716144 -3.042193 -0.524919
H 6.941074 -3.295583 -1.212487
78
5-c2.out delta G = 2.7573 kcal/mol
conformer 10
O 0.365955 -0.319619 -1.653344
C 0.465549 -1.542519 -2.192279
C 1.080629 3.591748 -0.344958

O 0.079594 1.541510 2.419317
C 1.023291 1.296653 1.661292
N 2.280922 1.121322 2.068715
C 0.839435 -1.466623 -3.641608
O 0.410640 4.741646 -0.872941
C 3.282487 0.893058 1.043696
O -1.511429 3.107373 0.935205
O -3.585554 -1.124276 1.713178
C 2.467125 1.047854 -0.282920
C 3.018307 2.238727 -1.044247
O 0.290814 -2.573450 -1.576074
O -1.501424 -2.470672 0.544194
C 2.434629 3.444322 -1.012870
O -4.245377 -2.822815 -1.364403
C 0.270223 2.316470 -0.583956
C 4.050562 -0.426226 1.230030
C 0.964746 1.138846 0.127579
C 4.324018 1.927884 -1.723978
C 3.019982 4.702993 -1.587668
C -1.231169 2.468667 -0.311225
C -1.973017 1.136795 -0.523698
C -3.433126 1.232808 -0.086658
C -4.218223 -0.050340 -0.355172
C -3.519370 -1.262087 0.294693
C -4.218059 -2.558208 -0.177917
C -2.053179 -1.348473 -0.151061
C 0.194715 -0.158120 -0.211846
C -1.287301 -0.058926 0.154013
C 1.646501 -3.243763 2.122385
C 2.378247 -2.063344 2.192510
C 3.238816 -1.691699 1.154775
C -5.673890 0.081845 0.093406
C -4.845980 -3.445674 0.856269
C 2.633045 -3.727676 -0.014878
C 3.352466 -2.539678 0.051285
C 1.774462 -4.082092 1.018937
H 1.215423 3.748017 0.733184
H 2.515005 1.206290 3.047920
H 0.701557 -2.436769 -4.110484
H 1.888310 -1.171948 -3.718540
H 0.244747 -0.708624 -4.149898
H -0.355582 4.890559 -0.304081
H 4.020342 1.694670 1.103776
H -1.072090 2.581305 1.633710
H -2.918480 -1.714462 2.089703
H 2.617692 0.156206 -0.889205
H -0.717523 -2.751137 0.042046
H 0.347775 2.096808 -1.650858
H 4.833462 -0.450047 0.470115
H 4.559879 -0.372988 2.196349
H 4.186388 1.096361 -2.421930
H 5.083973 1.606538 -1.004786
H 4.730765 2.766692 -2.281455
H 4.039168 4.565776 -1.938263
H 2.416034 5.079125 -2.415682
H 3.025632 5.489887 -0.828963
H -1.618353 3.160198 -1.061359
H -1.949796 0.955681 -1.604475
H -3.917388 2.059436 -0.613732
H -3.477148 1.469376 0.977173
H -4.203077 -0.249231 -1.430050
H -2.058041 -1.542673 -1.225024
H 0.659268 -1.010437 0.278976
H -1.320295 0.082303 1.235037
H 0.980788 -3.511623 2.933194
H 2.286141 -1.432401 3.066721
H -6.140060 0.935820 -0.401015
H -5.735981 0.238015 1.170992
H -6.259718 -0.804873 -0.157301
H -4.068818 -3.840468 1.515561
H -5.366786 -4.267568 0.369911
H -5.528909 -2.873736 1.485668
H 2.732249 -4.368420 -0.881310
H 4.019055 -2.270226 -0.759532
H 1.206407 -5.002139 0.964786

78

5-c8.out delta G = 3.2361 kcal/mol

conformer 11

O -0.233951 -0.783544 -1.265780
C 0.150647 -2.056523 -1.482548
C 1.117514 3.223522 -0.457542
O -0.269651 1.877295 2.387361
C 0.594192 1.244956 1.771767
N 1.688353 0.717614 2.325598
C 0.258551 -2.336923 -2.952135
O 0.754510 4.317872 -1.312090
C 2.635305 0.087356 1.417653
O -1.620440 3.271938 0.519010

O -4.400411 -0.241203 1.671498
C 2.065821 0.473703 0.014346
C 2.937155 1.545085 -0.610968
O 0.399072 -2.846345 -0.599970
O -2.448371 -2.069494 1.200724
C 2.541528 2.815193 -0.766091
O -5.031952 -2.508648 -1.023196
C 0.171113 2.040825 -0.656822
C 2.786389 -1.421652 1.674682
C 0.591380 0.890004 0.272385
C 4.318800 1.057187 -0.977768
C 3.448445 3.927701 -1.215195
C -1.313437 2.438218 -0.599084
C -2.230280 1.209832 -0.715495
C -3.701685 1.587160 -0.581066
C -4.626266 0.378391 -0.714165
C -4.253704 -0.701832 0.325155
C -5.083153 -1.980350 0.070175
C -2.769142 -1.096249 0.203553
C -0.383512 -0.299574 0.096585
C -1.841770 0.116511 0.287194
C 6.341343 -2.147233 0.540007
C 5.259604 -1.688319 1.281792
C 3.949412 -1.992538 0.902583
C -6.098183 0.788011 -0.625479
C -5.916852 -2.531083 1.189573
C 4.829907 -3.242152 -0.976869
C 3.748232 -2.780679 -0.230662
C 6.129258 -2.922621 -0.597664
H 1.056262 3.574402 0.579610
H 1.864458 0.812237 3.315742
H 0.377058 -3.404311 -3.115833
H 1.130745 -1.811506 -3.347311
H -0.619498 -1.964956 -3.478778
H -0.039438 4.713169 -0.931824
H 3.609112 0.552580 1.565225
H -1.256438 2.837122 1.318178
H -5.234213 0.231056 1.771910
H 2.103096 -0.403557 -0.629146
H -2.696460 -1.689379 2.054762
H 0.338873 1.673903 -1.672885
H 2.947675 -1.564943 2.746280
H 1.864081 -1.937650 1.408460
H 4.278023 0.014870 -1.298945
H 5.011258 1.092959 -0.132026
H 4.755591 1.639307 -1.786140
H 4.494295 3.631825 -1.216500
H 3.184084 4.282730 -2.213498
H 3.340384 4.784667 -0.545427
H -1.510095 3.061582 -1.473233
H -2.073785 0.806643 -1.721233
H -3.962152 2.318485 -1.350898
H -3.865846 2.072884 0.382361
H -4.463242 -0.085897 -1.690091
H -2.644928 -1.599206 -0.754509
H -0.132765 -1.111976 0.772145
H -1.930654 0.519305 1.297785
H 7.349780 -1.900550 0.848448
H 5.434113 -1.083281 2.164383
H -6.770670 -0.061957 -0.754188
H -6.328313 1.512768 -1.407423
H -6.333294 1.269765 0.328136
H -6.663914 -1.799619 1.507437
H -5.285821 -2.728848 2.057512
H -6.412785 -3.442390 0.863160
H 4.655079 -3.851486 -1.855095
H 2.739419 -3.037832 -0.525173
H 6.970739 -3.279090 -1.178428
78
5-c11.out delta G = 3.9357 kcal/mol
conformer 12
O 0.348818 -0.309806 -1.626343
C 0.450805 -1.539272 -2.150358
C 1.041668 3.635540 -0.462340
O 0.102197 1.558712 2.449179
C 1.032309 1.315305 1.674729
N 2.289129 1.107315 2.068938
C 0.822175 -1.480699 -3.601144
O 0.365469 4.701033 -1.147649
C 3.277696 0.873910 1.034759
O -1.523626 3.075113 0.989297
O -3.611254 -1.085085 1.732643
C 2.451854 1.052588 -0.283644
C 3.024501 2.243044 -1.021376
O 0.279653 -2.562616 -1.521190
O -1.512554 -2.438577 0.595760
C 2.449831 3.452305 -1.001761
O -4.240120 -2.825793 -1.327757

C 0.248795 2.336290 -0.585775
C 4.036991 -0.452513 1.202373
C 0.956219 1.169448 0.139602
C 4.355365 1.944729 -1.658987
C 3.113957 4.709623 -1.500938
C -1.250349 2.489648 -0.278978
C -1.988117 1.158126 -0.514474
C -3.453179 1.249271 -0.093621
C -4.230633 -0.039872 -0.354977
C -3.533278 -1.240577 0.316554
C -4.223862 -2.545007 -0.144751
C -2.063906 -1.326994 -0.117279
C 0.178691 -0.130999 -0.186758
C -1.303919 -0.031152 0.175401
C 1.629017 -3.282361 2.049315
C 2.347226 -2.094173 2.130394
C 3.227487 -1.718703 1.110860
C -5.691299 0.091750 0.077358
C -4.858187 -3.420502 0.895753
C 2.667330 -3.765466 -0.063292
C 3.372832 -2.569941 0.013431
C 1.790599 -4.124654 0.953486
H 1.065697 3.970921 0.578111
H 2.531818 1.175266 3.047349
H 0.231811 -0.724196 -4.116543
H 0.677222 -2.454900 -4.059401
H 1.873084 -1.194593 -3.683015
H 0.325688 4.479164 -2.086895
H 4.025227 1.666317 1.096148
H -1.067925 2.536668 1.666960
H -2.945873 -1.668822 2.121931
H 2.583726 0.165101 -0.899903
H -0.726655 -2.724887 0.100240
H 0.293833 2.064638 -1.644885
H 4.819925 -0.468253 0.442348
H 4.547804 -0.413467 2.168843
H 5.109342 1.661039 -0.918328
H 4.749507 2.779040 -2.232432
H 4.250273 1.092897 -2.337433
H 4.193309 4.601693 -1.572298
H 2.740259 5.017060 -2.481054
H 2.901619 5.533757 -0.816888
H -1.644875 3.204899 -1.000873
H -1.950940 0.977937 -1.595043
H -3.935959 2.068063 -0.634416
H -3.507889 1.497369 0.966861
H -4.203956 -0.252416 -1.427064
H -2.061484 -1.535101 -1.188510
H 0.643857 -0.977421 0.313529
H -1.339886 0.121089 1.255070
H 0.947922 -3.552948 2.846296
H 2.229623 -1.461261 2.999994
H -5.764874 0.261440 1.152208
H -6.271187 -0.800436 -0.167827
H -6.155902 0.937523 -0.432412
H -5.374181 -4.249228 0.415887
H -5.546347 -2.841961 1.513256
H -4.085098 -3.805940 1.565332
H 2.793312 -4.409623 -0.923879
H 4.054819 -2.297307 -0.783405
H 1.233510 -5.050853 0.891266

**Table S25.** Geometry data of conformers of compound **6**.

84
6-c51.out delta G = 0.0000 kcal/mol conformer 1
O -0.642378 -0.774660 -1.347575
C -1.262092 -1.936223 -1.586833
C 2.536756 1.912823 -0.618235
O 0.389491 1.526822 2.345291
C 2.942529 3.848693 0.781145
C 0.950069 0.615492 1.748250
N 1.741676 -0.311049 2.317467
C 3.469393 4.207465 2.153835
O 2.913993 2.424533 0.670130
C 2.199303 -1.390274 1.462611
O 0.330410 3.672689 -1.401766
O -4.142651 1.758290 1.557083
C 1.858100 -0.867227 0.032130
C 3.124304 -0.491135 -0.710632
O -3.418977 -0.752853 0.799692
C 3.466844 0.776350 -0.963946
O -5.663888 0.475501 -1.414111
C 1.079421 1.463905 -0.686018
C 1.526376 -2.727761 1.833717
C 0.822340 0.269430 0.245177
C 3.955752 -1.683932 -1.104067

C 4.762366 1.224769 -1.579357
C 0.052208 2.585962 -0.506189
C -1.377335 2.071015 -0.707501
C -2.413984 3.156587 -0.422879
C -3.846489 2.675291 -0.654508
C -4.141191 1.408532 0.174624
C -5.506198 0.825715 -0.260450
C -3.082031 0.325087 -0.081471
C -0.613842 -0.246249 0.014883
C -1.665844 0.845933 0.171297
C 1.753243 -5.389809 -0.878605
C 1.253737 -4.388595 -0.050457
C 2.026041 -3.869677 0.989739
C -4.861416 3.786260 -0.382911
C -6.593210 0.714799 0.767733
C 3.807301 -5.393217 0.373288
C 3.304746 -4.392856 1.196703
C 3.034600 -5.890579 -0.673349
C -1.036549 -2.406721 -2.989958
O -1.903557 -2.543730 -0.748353
H 2.675211 2.706735 -1.356898
H 3.592186 4.259468 -0.001592
H 1.943328 4.266487 0.630542
H 1.901873 -0.304335 3.314203
H 4.470988 3.799659 2.302229
H 2.815827 3.814876 2.934430
H 3.519748 5.292680 2.262856
H 3.277277 -1.504233 1.574255
H 0.216588 3.355225 -2.307124
H -4.036322 0.938916 2.059846
H 1.398025 -1.679721 -0.526132
H -2.970864 -1.550448 0.470616
H 0.946485 1.084318 -1.705367
H 1.731690 -2.924669 2.889033
H 0.444321 -2.627105 1.732083
H 3.321300 -2.442419 -1.570104
H 4.416578 -2.165142 -0.238147
H 4.746837 -1.429499 -1.804300
H 5.200008 2.015656 -0.963197
H 5.499718 0.431288 -1.671797
H 4.594956 1.655650 -2.571875
H 0.139627 3.009254 0.491445
H -1.466158 1.765409 -1.758802
H -2.224986 4.021717 -1.059368
H -2.306312 3.490032 0.613509
H -3.940063 2.368752 -1.699685
H -3.189968 0.005770 -1.120812
H -0.824422 -1.070405 0.691851
H -1.613893 1.160331 1.213953
H 1.139793 -5.778124 -1.682150
H 0.249584 -4.014926 -0.208868
H -4.827633 4.097451 0.661756
H -5.881420 3.470628 -0.611588
H -4.637592 4.654972 -1.004210
H -6.283477 0.015720 1.548697
H -7.509160 0.360416 0.300082
H -6.756427 1.676010 1.256672
H 4.802090 -5.784543 0.546592
H 3.913860 -4.008764 2.007010
H 3.425982 -6.667797 -1.317573
H -1.781145 -3.152563 -3.253805
H -0.043584 -2.859301 -3.046031
H -1.062412 -1.570926 -3.686884
84
6-c18.out delta G = 0.2259 kcal/mol
conformer 2
O -0.636968 -0.814310 -1.312492
C -1.257193 -1.982708 -1.512641
C 2.540572 1.920111 -0.699704
O 0.481092 1.548854 2.319021
C 2.918387 3.856327 0.712351
C 1.030130 0.634589 1.715500
N 1.841040 -0.282394 2.272736
C 3.567767 4.223386 2.029472
O 2.952276 2.435502 0.577178
C 2.286644 -1.364715 1.415619
O 0.342465 3.567443 -1.531391
O -4.069962 1.759374 1.632160
C 1.902744 -0.858123 -0.009932
C 3.145124 -0.479018 -0.790577
O -3.349924 -0.771586 0.919893
C 3.469938 0.788840 -1.063966
O -5.674381 0.375412 -1.248738
C 1.085962 1.456681 -0.731665
C 1.637677 -2.705352 1.817040
C 0.863018 0.272034 0.219745
C 3.974042 -1.668717 -1.198134
C 4.742196 1.242687 -1.722292

C 0.052731 2.564558 -0.541095
C -1.374495 2.030490 -0.714988
C -2.419410 3.111820 -0.441397
C -3.853522 2.609271 -0.614749
C -4.109646 1.367064 0.261763
C -5.482613 0.759915 -0.111389
C -3.048818 0.286868 0.002816
C -0.574461 -0.259735 0.038268
C -1.631581 0.826771 0.202070
C 1.814295 -5.386735 -0.879731
C 1.328606 -4.383772 -0.045541
C 2.124410 -3.849745 0.968946
C -4.870805 3.718993 -0.347364
C -6.533832 0.669463 0.955180
C 3.901968 -5.361901 0.315038
C 3.413077 -4.359763 1.144511
C 3.105423 -5.874246 -0.706194
C -1.073008 -2.475045 -2.914383
O -1.869101 -2.579309 -0.644605
H 2.652747 2.713187 -1.442762
H 3.457645 4.313355 -0.126170
H 1.888232 4.222874 0.680227
H 2.028780 -0.264034 3.264480
H 3.026041 3.777374 2.865080
H 3.564722 5.307520 2.158974
H 4.601782 3.875236 2.060840
H 3.368364 -1.467074 1.499313
H -0.077205 4.391940 -1.264098
H -3.941463 0.956978 2.156597
H 1.434917 -1.679636 -0.548047
H -2.906597 -1.573553 0.595180
H 0.933815 1.069620 -1.744095
H 1.874860 -2.890954 2.867719
H 0.552227 -2.615987 1.745442
H 4.458961 -2.142305 -0.341227
H 4.746024 -1.413195 -1.919000
H 3.333146 -2.433691 -1.644385
H 5.187678 2.047291 -1.130060
H 5.485683 0.456154 -1.824946
H 4.541399 1.657301 -2.715567
H 0.151467 2.991377 0.457041
H -1.468465 1.703361 -1.756804
H -2.274398 3.954345 -1.121213
H -2.288600 3.490245 0.576930
H -3.973934 2.268940 -1.646438
H -3.183622 -0.059196 -1.024659
H -0.759004 -1.072527 0.736295
H -1.552813 1.164775 1.235758
H 1.182593 -5.786668 -1.663180
H 0.317019 -4.020433 -0.178538
H -4.673258 4.571237 -0.999631
H -4.811085 4.060388 0.686643
H -5.893394 3.387433 -0.538417
H -6.689340 1.643060 1.421604
H -6.191975 -0.004403 1.744861
H -7.461514 0.294281 0.528707
H 4.904756 -5.742830 0.463668
H 4.040741 -3.963813 1.934702
H 3.486171 -6.652734 -1.355239
H -1.151373 -1.653724 -3.624696
H -1.805800 -3.246235 -3.134484
H -0.069961 -2.899055 -3.002954

84

6-c1.out delta G = 0.4185 kcal/mol

conformer 3

O -0.119915 -0.668250 -1.750243
C -0.699903 -1.758632 -2.264378
C 2.656410 2.195689 -0.727673
O 0.711940 1.102015 2.264458
C 2.965137 3.742081 1.114496
C 1.388986 0.401861 1.518316
N 2.380457 -0.406856 1.922166
C 3.572846 3.794104 2.499551
O 3.151681 2.422567 0.601389
C 3.096766 -1.141076 0.901320
O 0.183450 3.771917 -1.027937
O -3.755973 0.744746 1.765500
C 2.413890 -0.672716 -0.425812
C 3.458569 -0.055860 -1.331799
O -2.789765 -1.459098 0.490266
C 3.619262 1.267609 -1.432389
O -5.346670 -0.173030 -1.303186
C 1.253835 1.595394 -0.752581
C 3.092869 -2.661351 1.151918
C 1.225980 0.245656 -0.011955
C 4.325965 -1.081311 -2.008554
C 4.714789 1.969314 -2.181863
C 0.111986 2.522773 -0.324329

C -1.249150 1.850148 -0.544978
C -2.391372 2.709338 -0.004040
C -3.765139 2.082702 -0.242130
C -3.826105 0.658050 0.343323
C -5.138631 -0.018991 -0.114964
C -2.664531 -0.200860 -0.178990
C -0.130750 -0.430016 -0.306217
C -1.308623 0.454312 0.093721
C 0.114693 -4.721189 -0.013772
C 1.363808 -4.111865 0.030791
C 1.739178 -3.319225 1.117778
C -4.887433 2.971834 0.294364
C -6.117328 -0.459930 0.933369
C -0.428654 -3.749292 2.115909
C 0.826124 -3.151459 2.163296
C -0.788033 -4.538040 1.027100
C -0.390138 -1.900253 -3.724192
O -1.376715 -2.537612 -1.623501
H 2.633212 3.150631 -1.259034
H 3.451772 4.463261 0.446176
H 1.902258 3.994402 1.154443
H 2.667578 -0.409989 2.889758
H 4.636235 3.550054 2.464409
H 3.074563 3.083542 3.160929
H 3.462536 4.794664 2.922238
H 4.140574 -0.819788 0.900610
H 0.055899 3.596987 -1.969488
H -3.495404 -0.127924 2.091228
H 2.015639 -1.552283 -0.929639
H -2.281728 -2.105176 -0.030798
H 1.071603 1.364289 -1.806821
H 3.740478 -3.119329 0.402443
H 3.567162 -2.835942 2.121684
H 5.041767 -0.646665 -2.701144
H 3.701404 -1.787054 -2.564290
H 4.887918 -1.672164 -1.278247
H 4.306972 2.543129 -3.020338
H 5.205982 2.688520 -1.519307
H 5.478173 1.298533 -2.568394
H 0.209430 2.782976 0.725813
H -1.378095 1.734206 -1.629777
H -2.364929 3.692967 -0.474785
H -2.243339 2.862446 1.069142
H -3.904491 1.961498 -1.319746
H -2.816630 -0.331716 -1.252493
H -0.187030 -1.396033 0.190857
H -1.222147 0.577028 1.173704
H -0.158051 -5.326823 -0.868285
H 2.060845 -4.256915 -0.786390
H -5.874289 2.567907 0.059148
H -4.822237 3.965102 -0.152880
H -4.813597 3.080696 1.376980
H -5.664351 -1.242326 1.547425
H -7.018733 -0.841553 0.458804
H -6.355055 0.365467 1.605651
H -1.125129 -3.601101 2.931717
H 1.098589 -2.550845 3.020788
H -1.765608 -5.001812 0.989201
H -0.544253 -0.951865 -4.238047
H -1.013339 -2.674833 -4.161879
H 0.661792 -2.170605 -3.836939
84
6-c40.out delta G = 0.7204 kcal/mol
conformer 4
O -0.807558 -0.715273 -1.356448
C -1.084515 -2.013680 -1.581411
C 2.506085 1.850725 -0.667553
O 0.402925 1.519380 2.318811
C 2.922726 3.717726 0.825801
C 0.929635 0.591307 1.714488
N 1.702421 -0.353651 2.275040
C 3.616251 4.018429 2.137226
O 2.942574 2.304221 0.623476
C 2.183614 -1.411362 1.407845
O 0.331933 3.665894 -1.419355
O -4.065425 1.931230 1.573823
C 1.783105 -0.908827 -0.017653
C 3.021185 -0.562892 -0.822546
O -3.437318 -0.684759 0.893477
C 3.389260 0.697540 -1.075673
O -6.400667 1.116575 0.865047
C 1.036409 1.439408 -0.713547
C 1.584291 -2.777391 1.796270
C 0.767174 0.246232 0.216765
C 3.789306 -1.775407 -1.274203
C 4.661716 1.123709 -1.751888
C 0.032665 2.582645 -0.526570
C -1.403590 2.089367 -0.727284

C -2.427159 3.199863 -0.503242
C -3.858405 2.714459 -0.735228
C -4.157576 1.524984 0.211761
C -5.577178 0.987737 -0.023332
C -3.134918 0.389719 0.004830
C -0.689028 -0.231144 0.011957
C -1.702668 0.895465 0.192388
C 2.275954 -5.518208 -0.755414
C 1.624915 -4.519934 -0.035480
C 2.242008 -3.914258 1.059332
C -4.874108 3.844221 -0.579859
C -5.905344 0.314607 -1.320845
C 4.173237 -5.338090 0.711845
C 3.520399 -4.341909 1.428204
C 3.553990 -5.926418 -0.388589
C -1.154569 -2.295948 -3.053468
O -1.228452 -2.837313 -0.705898
H 2.649003 2.665559 -1.382596
H 3.439195 4.209767 -0.007165
H 1.895050 4.090985 0.843205
H 1.900074 -0.334124 3.264719
H 3.624322 5.095163 2.318235
H 4.648477 3.663983 2.118925
H 3.098655 3.536071 2.967968
H 3.269391 -1.474216 1.490943
H 0.225839 3.348345 -2.325660
H -4.974606 1.893392 1.919498
H 1.289859 -1.731825 -0.531285
H -3.500784 -0.294313 1.777399
H 0.881536 1.066048 -1.732309
H 1.719779 -2.908408 2.872960
H 0.511283 -2.765465 1.600094
H 4.154474 -2.363252 -0.429246
H 4.638966 -1.526426 -1.903774
H 3.130619 -2.439436 -1.840998
H 5.377682 0.316988 -1.887783
H 4.453570 1.564704 -2.732268
H 5.144779 1.901819 -1.153669
H 0.125489 3.001514 0.472063
H -1.487224 1.747082 -1.767080
H -2.220651 4.031045 -1.177908
H -2.336081 3.581752 0.517654
H -3.915043 2.332031 -1.758837
H -3.247895 -0.012791 -1.001389
H -0.921201 -1.056639 0.678674
H -1.607995 1.234828 1.224534
H 1.783177 -5.975807 -1.604564
H 0.630254 -4.205976 -0.324852
H -4.611451 4.675442 -1.235801
H -4.886180 4.213268 0.446837
H -5.885994 3.525065 -0.837838
H -5.537843 -0.714450 -1.274917
H -5.421861 0.795894 -2.169850
H -6.983526 0.293120 -1.462691
H 5.164537 -5.655712 1.010935
H 4.010101 -3.886516 2.281580
H 4.061857 -6.701057 -0.949381
H -1.747363 -1.536931 -3.562456
H -1.576151 -3.283267 -3.220331
H -0.144026 -2.260839 -3.466319
84
6-c30.out delta G = 0.8873 kcal/mol
conformer 5
O -0.623440 -0.780063 -1.308531
C -1.197122 -1.971092 -1.510622
C 2.454754 2.025328 -0.680670
O 0.314853 1.612236 2.335326
C 4.011717 2.960746 0.914981
C 0.939934 0.744278 1.738349
N 1.810206 -0.108349 2.311104
C 4.040521 3.529394 2.318254
O 2.689113 2.517218 0.644380
C 2.296089 -1.193333 1.481399
O 0.236139 3.636118 -1.543963
O -4.193564 1.714643 1.539377
C 1.890035 -0.740247 0.046652
C 3.100324 -0.359974 -0.783328
O -3.388607 -0.790906 0.850419
C 3.387676 0.906029 -1.107509
O -5.683367 0.289973 -1.383013
C 1.013853 1.539704 -0.728935
C 1.684685 -2.540183 1.924681
C 0.811981 0.361877 0.241441
C 3.896177 -1.557803 -1.224632
C 4.532539 1.338451 -1.983133
C -0.036843 2.631952 -0.548899
C -1.449367 2.062073 -0.739624
C -2.526994 3.115751 -0.486905

C -3.941725 2.575238 -0.699743
C -4.186757 1.324633 0.167673
C -5.533753 0.683701 -0.242438
C -3.091688 0.272383 -0.062830
C -0.606441 -0.210020 0.037733
C -1.694526 0.850394 0.170199
C 1.844688 -5.267732 -0.724359
C 1.365333 -4.249792 0.095123
C 2.167912 -3.698607 1.095300
C -4.996193 3.656202 -0.459827
C -6.613830 0.575714 0.793023
C 3.939896 -5.223665 0.456659
C 3.457567 -4.205896 1.271312
C 3.136492 -5.753587 -0.550172
C -0.944347 -2.481391 -2.895226
O -1.818537 -2.575564 -0.654723
H 2.568956 2.858221 -1.381189
H 4.713007 2.123113 0.829381
H 4.307389 3.721886 0.180755
H 1.967326 -0.078744 3.307813
H 3.353339 4.372352 2.408918
H 5.046259 3.875649 2.563881
H 3.751333 2.768292 3.044974
H 3.381503 -1.260547 1.562082
H -0.155744 4.465693 -1.252495
H -4.049922 0.915187 2.064500
H 1.444438 -1.594636 -0.459046
H -2.915440 -1.582420 0.542553
H 0.872191 1.138932 -1.736269
H 1.946526 -2.695990 2.974329
H 0.596584 -2.468725 1.874996
H 4.769992 -1.298936 -1.815041
H 3.266401 -2.223579 -1.822864
H 4.231157 -2.148992 -0.369825
H 4.152120 1.695558 -2.946097
H 5.072190 2.176128 -1.536529
H 5.251455 0.547966 -2.181505
H 0.053517 3.068703 0.445435
H -1.517685 1.734454 -1.783638
H -2.384797 3.964910 -1.158895
H -2.432046 3.491563 0.536309
H -4.024417 2.233812 -1.734895
H -3.190743 -0.080051 -1.092190
H -0.785426 -1.020102 0.740606
H -1.649305 1.190877 1.204743
H 1.207585 -5.680075 -1.496935
H 0.353011 -3.887997 -0.037753
H -4.974181 3.998662 0.575300
H -6.003922 3.297614 -0.678968
H -4.804090 4.514060 -1.106335
H -7.522551 0.185311 0.340130
H -6.797759 1.546667 1.254432
H -6.283479 -0.092493 1.592372
H 4.943243 -5.603004 0.605650
H 4.090628 -3.796204 2.050119
H 3.512659 -6.544012 -1.187349
H -0.949632 -1.666891 -3.616941
H -1.685486 -3.232733 -3.153729
H 0.046953 -2.941255 -2.913671

84

6-c33.out delta G = 0.9388 kcal/mol

conformer 6

O -0.114533 -0.668857 -1.742343
C -0.694099 -1.759228 -2.256274
C 2.667061 2.183198 -0.716775
O 0.724259 1.113288 2.270508
C 2.930825 3.666463 1.190312
C 1.391575 0.400008 1.527283
N 2.373138 -0.419380 1.933551
C 3.763491 3.788073 2.448913
O 3.192656 2.392943 0.604402
C 3.093880 -1.148275 0.911294
O 0.150076 3.715748 -1.136839
O -3.760161 0.759417 1.755983
C 2.411089 -0.679417 -0.415507
C 3.452133 -0.070046 -1.330209
O -2.792214 -1.447854 0.488180
C 3.616132 1.252396 -1.437215
O -5.340660 -0.160464 -1.317314
C 1.259494 1.594123 -0.739771
C 3.093700 -2.669120 1.158397
C 1.227151 0.244291 -0.001600
C 4.306605 -1.100923 -2.014619
C 4.700697 1.949392 -2.206342
C 0.115084 2.525807 -0.324345
C -1.242332 1.855592 -0.550354
C -2.384032 2.717758 -0.013070
C -3.758701 2.094258 -0.254032

C -3.824848 0.670749 0.333536
C -5.136794 -0.004631 -0.128610
C -2.662722 -0.190767 -0.182799
C -0.130636 -0.427008 -0.299158
C -1.306567 0.462506 0.093309
C 0.130465 -4.736183 -0.032264
C 1.377356 -4.123241 0.022684
C 1.742182 -3.330937 1.113574
C -4.880686 2.986723 0.277579
C -6.120442 -0.442082 0.916648
C -0.431995 -3.768796 2.094446
C 0.820641 -3.167197 2.152187
C -0.780692 -4.557222 1.002002
C -0.379139 -1.903692 -3.714730
O -1.374971 -2.535853 -1.616604
H 2.650847 3.143139 -1.241760
H 3.189135 4.457425 0.475538
H 1.869351 3.764153 1.438828
H 2.662099 -0.421208 2.900658
H 3.502499 3.000304 3.157473
H 3.583953 4.752811 2.927246
H 4.826956 3.709012 2.217009
H 4.136550 -0.823124 0.913098
H 0.568242 4.423067 -0.636190
H -3.502284 -0.113303 2.083701
H 2.007562 -1.558869 -0.915102
H -2.284704 -2.095768 -0.031279
H 1.076469 1.371099 -1.793747
H 3.747593 -3.123637 0.412335
H 3.562258 -2.844410 2.130824
H 3.672660 -1.798046 -2.570695
H 4.864695 -1.700238 -1.288324
H 5.024562 -0.671067 -2.707955
H 5.207090 2.667405 -1.554073
H 5.453683 1.274870 -2.606369
H 4.279663 2.523400 -3.038124
H 0.197812 2.794578 0.726266
H -1.363543 1.743453 -1.634618
H -2.353078 3.699894 -0.486298
H -2.239373 2.871617 1.060724
H -3.894933 1.971745 -1.331863
H -2.810818 -0.323183 -1.256651
H -0.192448 -1.391551 0.200162
H -1.224238 0.587248 1.173618
H -0.133938 -5.341435 -0.889698
H 2.081007 -4.265099 -0.789363
H -5.867757 2.584541 0.040158
H -4.812077 3.979054 -0.171244
H -4.809927 3.097294 1.360255
H -5.671379 -1.224396 1.533668
H -7.020669 -0.822793 0.439111
H -6.359321 0.384786 1.586698
H -1.135077 -3.623751 2.905149
H 1.084717 -2.566722 3.012402
H -1.756586 -5.023766 0.955957
H 0.676181 -2.161582 -3.824617
H -0.543486 -0.958983 -4.232341
H -0.992763 -2.686772 -4.150787

84

6-c16.out delta G = 1.0668 kcal/mol

conformer 7

O -0.622903 -0.770150 -1.317193
C -1.196733 -1.960276 -1.526588
C 2.452977 2.026233 -0.686094
O 0.311597 1.622636 2.326523
C 4.011335 2.962592 0.907406
C 0.936981 0.751594 1.734573
N 1.807489 -0.098274 2.310862
C 4.040254 3.532782 2.309985
O 2.687760 2.521024 0.636893
C 2.295182 -1.185563 1.485064
O 0.194434 3.716652 -1.474139
O -4.198927 1.700258 1.546292
C 1.889228 -0.738067 0.048429
C 3.098857 -0.359363 -0.783351
O -3.389852 -0.794190 0.835624
C 3.385377 0.905844 -1.111812
O -5.681027 0.306551 -1.394654
C 1.011786 1.541536 -0.731553
C 1.684808 -2.531464 1.932413
C 0.810627 0.363879 0.239351
C 3.895597 -1.558027 -1.220708
C 4.529246 1.336395 -1.989557
C -0.040379 2.639997 -0.554170
C -1.454460 2.072406 -0.732064
C -2.528190 3.125109 -0.461699
C -3.942381 2.587725 -0.682122
C -4.188952 1.326418 0.170134

C -5.534657 0.689754 -0.249987
C -3.093015 0.276348 -0.069005
C -0.607270 -0.207075 0.032041
C -1.695479 0.852535 0.168542
C 1.853678 -5.268163 -0.706693
C 1.371295 -4.247578 0.107730
C 2.170815 -3.692364 1.108143
C -4.997048 3.665801 -0.429921
C -6.618171 0.572516 0.780947
C 3.945730 -5.218303 0.479718
C 3.460427 -4.197983 1.289364
C 3.145313 -5.752532 -0.527225
C -0.952361 -2.455326 -2.918180
O -1.814244 -2.571573 -0.672980
H 2.568711 2.858232 -1.388715
H 4.710921 2.123467 0.823043
H 4.308314 3.722352 0.172392
H 1.965754 -0.063909 3.307231
H 5.046415 3.877820 2.555558
H 3.749686 2.772915 3.037433
H 3.354238 4.376789 2.399263
H 3.380623 -1.251170 1.566701
H 0.082910 3.377215 -2.371838
H -4.056317 0.894588 2.062132
H 1.444317 -1.594743 -0.454190
H -2.915139 -1.583142 0.523649
H 0.870391 1.134631 -1.737958
H 1.945224 -2.683015 2.983028
H 0.596700 -2.461395 1.880879
H 3.266353 -2.226262 -1.816737
H 4.230645 -2.146026 -0.363724
H 4.769480 -1.300646 -1.811641
H 5.246309 0.544655 -2.189396
H 4.147744 1.694247 -2.951827
H 5.071297 2.173053 -1.543911
H 0.055208 3.086199 0.432519
H -1.539937 1.746090 -1.777645
H -2.372116 3.985333 -1.113679
H -2.430468 3.479466 0.568661
H -4.025531 2.258883 -1.721501
H -3.191750 -0.067894 -1.101251
H -0.787166 -1.020377 0.730843
H -1.650898 1.188100 1.204605
H 1.219033 -5.683884 -1.479472
H 0.359317 -3.886564 -0.030162
H -4.804566 4.530673 -1.066875
H -4.974588 3.997080 0.608833
H -6.005126 3.310113 -0.652531
H -6.801252 1.538374 1.253171
H -6.291573 -0.105649 1.573457
H -7.526391 0.189265 0.320991
H 4.948948 -5.596363 0.632769
H 4.091053 -3.784988 2.068404
H 3.523683 -6.545083 -1.160452
H 0.067307 -2.844138 -2.971933
H -1.037232 -1.642140 -3.637104
H -1.650383 -3.253308 -3.155057
84
6-c50.out delta G = 1.1496 kcal/mol
conformer 8
O -0.554256 -0.264581 -2.147133
C -1.147918 -1.292829 -2.766766
C 2.544000 2.124658 -0.504825
O 0.442647 0.629576 2.117539
C 2.858167 3.387208 1.543005
C 1.027873 0.013570 1.234396
N 1.846075 -1.033688 1.435613
C 3.447565 3.225232 2.927775
O 2.924102 2.124194 0.880721
C 2.358614 -1.698834 0.253364
O 0.310565 3.921317 -0.729163
O -4.087055 1.003335 1.458280
C 1.972079 -0.718531 -0.896539
C 3.213159 -0.057568 -1.458438
O -3.288111 -1.091468 -0.094268
C 3.508343 1.228466 -1.244489
O -5.620294 0.690836 -1.757526
C 1.101928 1.680429 -0.740121
C 1.776097 -3.111268 0.071001
C 0.897446 0.226186 -0.293755
C 4.063572 -1.009621 -2.255906
C 4.771619 1.922933 -1.667551
C 0.033609 2.619444 -0.182692
C -1.374308 2.154881 -0.576684
C -2.455345 3.042659 0.039919
C -3.871207 2.602169 -0.334394
C -4.102356 1.123799 0.037282
C -5.454897 0.661569 -0.553358

C -3.009215 0.231445 -0.568761
C -0.521754 -0.234127 -0.687472
C -1.610108 0.696055 -0.162704
C 1.475299 -4.980983 3.371535
C 1.163636 -4.195306 2.265366
C 2.088367 -4.012397 1.235485
C -4.923765 3.521518 0.286011
C -6.521559 0.195615 0.393053
C 3.649246 -5.422816 2.439396
C 3.334695 -4.636079 1.336985
C 2.719656 -5.596636 3.461964
C -0.958515 -1.222126 -4.250770
O -1.752029 -2.174453 -2.183985
H 2.648127 3.141007 -0.891752
H 3.422223 4.129707 0.965527
H 1.823509 3.736340 1.606717
H 2.009618 -1.386339 2.366978
H 2.878910 2.494939 3.505658
H 3.423102 4.178250 3.459979
H 4.484501 2.889522 2.868844
H 3.442721 -1.781619 0.335698
H -0.155192 4.578741 -0.201616
H -3.938652 0.070291 1.664985
H 1.530289 -1.303390 -1.703155
H -2.821259 -1.713845 -0.677405
H 0.974966 1.689607 -1.827344
H 0.695483 -3.038411 -0.065753
H 2.193587 -3.523658 -0.850322
H 4.370951 -1.874179 -1.660776
H 4.961616 -0.547806 -2.656478
H 3.484032 -1.402832 -3.097287
H 5.541523 1.248026 -2.033402
H 4.566198 2.661508 -2.449132
H 5.185193 2.472008 -0.816725
H 0.102965 2.653676 0.904922
H -1.440532 2.224987 -1.668428
H -2.327081 4.075372 -0.292069
H -2.348912 3.036703 1.128908
H -3.966890 2.648828 -1.422377
H -3.125611 0.270409 -1.654261
H -0.692008 -1.247881 -0.333022
H -1.550769 0.638242 0.924506
H 0.745224 -5.113838 4.160307
H 0.192019 -3.720196 2.197950
H -4.892201 3.468921 1.374800
H -5.933275 3.262482 -0.039132
H -4.737306 4.555004 -0.010531
H -6.701237 0.945772 1.164194
H -6.181391 -0.706195 0.908338
H -7.435378 -0.018036 -0.156999
H 4.618202 -5.902739 2.499893
H 4.061923 -4.506191 0.543633
H 2.962859 -6.210214 4.320257
H -1.217418 -0.228180 -4.615094
H -1.570189 -1.975450 -4.738580
H 0.093732 -1.395622 -4.484516

84

6-c24.out delta G = 1.2789 kcal/mol

conformer 9

O -0.550724 -0.253756 -2.154094
C -1.141297 -1.277330 -2.784443
C 2.545873 2.116706 -0.513060
O 0.437036 0.637746 2.112332
C 2.874385 3.394363 1.522096
C 1.022675 0.015390 1.234010
N 1.840563 -1.030683 1.441825
C 3.447838 3.236055 2.913787
O 2.927359 2.123704 0.871393
C 2.355562 -1.701423 0.263737
O 0.267161 3.965013 -0.633867
O -4.094539 0.977548 1.455678
C 1.968887 -0.728058 -0.892242
C 3.209657 -0.070917 -1.459231
O -3.295508 -1.089151 -0.124542
C 3.507135 1.215678 -1.251169
O -5.615196 0.728891 -1.771492
C 1.102255 1.675148 -0.742135
C 1.774902 -3.115475 0.088571
C 0.895441 0.221100 -0.294783
C 4.058262 -1.027863 -2.252729
C 4.771145 1.906201 -1.678536
C 0.034959 2.622564 -0.182590
C -1.376270 2.158277 -0.560545
C -2.449364 3.038370 0.078944
C -3.866044 2.611117 -0.304709
C -4.103651 1.125869 0.037229
C -5.455453 0.679907 -0.567100
C -3.011347 0.239205 -0.580024

C -0.523629 -0.234148 -0.693784
C -1.611298 0.694185 -0.165057
C 1.475830 -4.967081 3.399474
C 1.163316 -4.188085 2.288827
C 2.087932 -4.009900 1.258033
C -4.916046 3.521306 0.333433
C -6.529230 0.205090 0.366836
C 3.650402 -5.411565 2.470052
C 3.335006 -4.631491 1.363161
C 2.720931 -5.580670 3.493521
C -0.940879 -1.197805 -4.266544
O -1.750622 -2.161668 -2.211423
H 2.653153 3.131415 -0.905644
H 3.456710 4.121775 0.943370
H 1.845174 3.760573 1.570065
H 2.004668 -1.377245 2.375381
H 3.434675 4.194846 3.435922
H 4.479462 2.882270 2.868487
H 2.861377 2.521785 3.493860
H 3.439705 -1.782089 0.347812
H 0.166244 3.982722 -1.594533
H -3.948544 0.040173 1.643948
H 1.526037 -1.318227 -1.694539
H -2.825993 -1.705406 -0.712115
H 0.973829 1.677990 -1.830602
H 0.694213 -3.044268 -0.048812
H 2.193162 -3.532326 -0.830383
H 4.955129 -0.568559 -2.658727
H 3.476561 -1.426981 -3.089791
H 4.367445 -1.888217 -1.652454
H 4.565803 2.644105 -2.460787
H 5.188316 2.455513 -0.829616
H 5.538397 1.228748 -2.045128
H 0.112417 2.668963 0.900830
H -1.462369 2.235873 -1.652732
H -2.300282 4.076047 -0.221650
H -2.340191 3.000474 1.166855
H -3.962324 2.679961 -1.391685
H -3.124819 0.293554 -1.665271
H -0.696737 -1.250097 -0.347173
H -1.555196 0.626416 0.921653
H 0.745868 -5.096260 4.188954
H 0.191193 -3.714421 2.218687
H -4.724996 4.559779 0.058021
H -4.885320 3.446814 1.420996
H -5.926577 3.273039 0.002770
H -7.441657 0.005079 -0.190639
H -6.708392 0.944025 1.148844
H -6.196262 -0.706390 0.869634
H 4.619951 -5.889915 2.533394
H 4.062197 -4.505140 0.569206
H 2.964832 -6.188994 4.355346
H -1.554081 -1.943584 -4.763905
H 0.111841 -1.377531 -4.493598
H -1.189804 -0.199638 -4.626162
84
6-c29.out delta G = 1.2902 kcal/mol
conformer 10
O -0.119331 -0.638139 -1.728056
C -0.651643 -1.751016 -2.243675
C 2.550637 2.287158 -0.709251
O 0.549808 1.137412 2.301924
C 4.186368 3.046979 0.896345
C 1.285069 0.486061 1.567302
N 2.311765 -0.265390 1.994725
C 4.336739 3.293007 2.382566
O 2.878073 2.540740 0.661428
C 3.077775 -0.976635 0.995743
O 0.012533 3.773450 -1.109838
O -3.862145 0.692657 1.706728
C 2.387175 -0.567002 -0.344463
C 3.400409 0.045357 -1.286326
O -2.823532 -1.467035 0.432245
C 3.520636 1.369121 -1.435477
O -5.369957 -0.198267 -1.411176
C 1.165579 1.663148 -0.731654
C 3.134389 -2.493090 1.270836
C 1.164820 0.322918 0.032446
C 4.249131 -0.985698 -1.976757
C 4.488908 2.061034 -2.353639
C 0.013603 2.577186 -0.306889
C -1.329799 1.882003 -0.542284
C -2.495349 2.711218 -0.004438
C -3.852941 2.064035 -0.277805
C -3.898693 0.628784 0.281863
C -5.186003 -0.068205 -0.216052
C -2.708536 -0.201074 -0.225372
C -0.169099 -0.385362 -0.287323

C -1.372665 0.478603 0.082329
C -0.367446 -3.698788 2.164617
C 0.866997 -3.062350 2.238625
C 1.807468 -3.201639 1.213525
C -5.001207 2.922983 0.253130
C -6.169956 -0.560950 0.804129
C 0.251504 -4.653196 0.047839
C 1.480434 -4.006666 0.119780
C -0.679280 -4.497275 1.068449
C -0.292707 -1.904301 -3.690960
O -1.324569 -2.541897 -1.612536
H 2.527854 3.244541 -1.242986
H 4.933126 2.323342 0.551910
H 4.332998 3.975617 0.328906
H 2.569955 -0.259081 2.970253
H 4.194438 2.364608 2.938287
H 3.602464 4.020969 2.731716
H 5.334799 3.676871 2.601856
H 4.106521 -0.608967 1.001213
H 0.598584 4.415499 -0.695899
H -3.585598 -0.178874 2.022370
H 2.016177 -1.474308 -0.818697
H -2.285402 -2.098284 -0.077575
H 0.980636 1.420693 -1.779725
H 3.817513 -2.935822 0.544166
H 3.591711 -2.633062 2.254178
H 4.780396 -1.606388 -1.248637
H 4.990513 -0.558768 -2.646207
H 3.614373 -1.662983 -2.556492
H 3.944039 2.609601 -3.128889
H 5.081559 2.802330 -1.811150
H 5.178308 1.381643 -2.848320
H 0.108483 2.844636 0.744229
H -1.442462 1.781439 -1.629040
H -2.477807 3.702379 -0.459008
H -2.368929 2.847759 1.073748
H -3.970314 1.959284 -1.359674
H -2.834763 -0.326650 -1.303167
H -0.217972 -1.347936 0.217589
H -1.314880 0.595234 1.164479
H -1.085461 -3.572074 2.965254
H 1.101394 -2.453973 3.101841
H -4.949876 3.923056 -0.180664
H -4.945309 3.019610 1.337984
H -5.976389 2.502438 -0.001247
H -5.704407 -1.344384 1.407340
H -7.051984 -0.956550 0.305203
H -6.442254 0.241252 1.491127
H 0.016105 -5.266510 -0.812256
H 2.199474 -4.131661 -0.681475
H -1.641657 -4.989603 1.008803
H -0.892465 -2.690395 -4.140771
H 0.765110 -2.165795 -3.764311
H -0.437806 -0.963267 -4.220419

84

6-c9.out delta G = 1.3849 kcal/mol

conformer 11

O -0.123284 -0.629710 -1.720341
C -0.646360 -1.749330 -2.231905
C 2.538592 2.308345 -0.694144
O 0.526465 1.165424 2.301872
C 4.172541 3.046887 0.919408
C 1.266177 0.511340 1.574399
N 2.288520 -0.241593 2.009592
C 4.325240 3.273640 2.408519
O 2.866459 2.538234 0.680352
C 3.069844 -0.941991 1.014552
O 0.044002 3.830048 -1.044862
O -3.878156 0.695932 1.701940
C 2.381765 -0.542346 -0.329933
C 3.392818 0.070320 -1.274739
O -2.825952 -1.468434 0.441154
C 3.507898 1.394355 -1.426804
O -5.375359 -0.221166 -1.413612
C 1.154548 1.681118 -0.727214
C 3.144361 -2.457510 1.290135
C 1.153420 0.342570 0.039647
C 4.244315 -0.959468 -1.963659
C 4.470943 2.089255 -2.347790
C -0.003566 2.592898 -0.317172
C -1.346500 1.883582 -0.545730
C -2.518526 2.710742 -0.019516
C -3.871900 2.053773 -0.291627
C -3.911400 0.622049 0.277684
C -5.193975 -0.084964 -0.218793
C -2.716102 -0.204948 -0.221982
C -0.175510 -0.374078 -0.280196
C -1.384700 0.483162 0.085729

C -0.347116 -3.707212 2.163120
C 0.879067 -3.055813 2.244699
C 1.826562 -3.181970 1.224436
C -5.025927 2.910156 0.230874
C -6.175742 -0.579957 0.802274
C 0.294528 -4.650124 0.047861
C 1.515066 -3.988756 0.127397
C -0.643349 -4.507577 1.064019
C -0.285326 -1.905884 -3.678228
O -1.313071 -2.542594 -1.597455
H 2.509235 3.274463 -1.208674
H 4.922075 2.331335 0.563722
H 4.313933 3.983721 0.364263
H 2.543563 -0.229530 2.985886
H 5.321940 3.659323 2.630899
H 4.188580 2.337217 2.952021
H 3.588135 3.993203 2.768888
H 4.093554 -0.560513 1.025568
H -0.073548 3.633010 -1.983483
H -3.599464 -0.172233 2.024828
H 2.015635 -1.453929 -0.799726
H -2.283320 -2.099057 -0.064579
H 0.982675 1.428457 -1.777038
H 3.837567 -2.891743 0.567901
H 3.597101 -2.591598 2.276397
H 4.980541 -0.531045 -2.637910
H 3.611754 -1.643715 -2.537595
H 4.782264 -1.573193 -1.234482
H 5.067445 2.827482 -1.805133
H 5.156527 1.411558 -2.850229
H 3.921724 2.642513 -3.116625
H 0.091612 2.877639 0.727169
H -1.459894 1.768021 -1.632686
H -2.506257 3.698774 -0.481780
H -2.395500 2.857594 1.057530
H -3.986211 1.940974 -1.373010
H -2.839043 -0.336470 -1.299472
H -0.218850 -1.335815 0.226871
H -1.329555 0.605907 1.167206
H -1.070757 -3.590782 2.960257
H 1.101637 -2.446136 3.110129
H -4.979539 3.907262 -0.210225
H -4.972455 3.015130 1.315045
H -5.998197 2.482158 -0.022129
H -7.055016 -0.982542 0.304090
H -6.453214 0.222816 1.486479
H -5.705901 -1.358725 1.408229
H 0.071024 -5.264737 -0.814493
H 2.239935 -4.103279 -0.670228
H -1.599211 -5.011709 0.998556
H -0.435091 -0.967738 -4.211471
H -0.880198 -2.697045 -4.125630
H 0.773972 -2.161933 -3.749549
84
6-c31.out delta G = 1.4728 kcal/mol
conformer 12
O -0.114618 -0.672876 -1.742258
C -0.697307 -1.767083 -2.244600
C 2.679800 2.189062 -0.736024
O 0.749392 1.095302 2.268152
C 2.997008 3.718981 1.119216
C 1.417441 0.394965 1.513914
N 2.405146 -0.422985 1.908497
C 3.659292 3.775769 2.479040
O 3.186598 2.406614 0.591057
C 3.114407 -1.152959 0.879728
O 0.262287 3.716011 -1.119665
O -3.724298 0.785780 1.781843
C 2.424232 -0.677353 -0.440882
C 3.465335 -0.064089 -1.353365
O -2.775124 -1.437401 0.519640
C 3.632940 1.258645 -1.450460
O -5.339517 -0.158507 -1.266154
C 1.274286 1.595182 -0.756557
C 3.110508 -2.674360 1.123238
C 1.243449 0.245936 -0.016081
C 4.318948 -1.092423 -2.042927
C 4.725590 1.957003 -2.207153
C 0.141812 2.523089 -0.323229
C -1.223220 1.857411 -0.549366
C -2.364608 2.724990 -0.018031
C -3.742168 2.099434 -0.241088
C -3.804142 0.682699 0.361334
C -5.122311 0.005535 -0.081029
C -2.649055 -0.184626 -0.159874
C -0.118176 -0.424593 -0.300047
C -1.289796 0.467612 0.101211
C -0.410376 -3.771642 2.080475

C 0.842535 -3.169798 2.130276
C 1.757494 -3.333453 1.085895
C -4.858296 2.998986 0.290808
C -6.094241 -0.422982 0.978613
C 0.138753 -4.738930 -0.049752
C 1.385648 -4.125389 -0.002943
C -0.765703 -4.560468 0.990500
C -0.370149 -1.939720 -3.697130
O -1.384535 -2.532170 -1.597914
H 2.656481 3.145851 -1.262389
H 3.441944 4.453359 0.436515
H 1.931805 3.950806 1.205509
H 2.697465 -0.432558 2.874502
H 3.545660 4.771360 2.912476
H 4.725102 3.554024 2.398988
H 3.203623 3.051509 3.156145
H 4.158618 -0.832861 0.874205
H -0.242837 4.417267 -0.695058
H -3.465274 -0.084090 2.116139
H 2.017877 -1.553605 -0.943778
H -2.275108 -2.088950 -0.002550
H 1.084772 1.370758 -1.809437
H 3.758207 -3.128882 0.371771
H 3.585115 -2.853408 2.092070
H 5.036999 -0.659040 -2.734000
H 3.684195 -1.785297 -2.603433
H 4.876775 -1.696818 -1.320636
H 4.313598 2.533761 -3.041536
H 5.224778 2.673186 -1.547300
H 5.483179 1.283655 -2.600667
H 0.241482 2.774196 0.731952
H -1.341592 1.741819 -1.632989
H -2.355180 3.700721 -0.509419
H -2.214175 2.899708 1.051687
H -3.886485 1.966469 -1.316471
H -2.806924 -0.323111 -1.231508
H -0.176793 -1.387202 0.203381
H -1.196797 0.595469 1.180181
H -1.108317 -3.626672 2.895617
H 1.112065 -2.569549 2.988985
H -4.792259 3.987045 -0.167749
H -4.779044 3.119248 1.371824
H -5.847419 2.595795 0.064401
H -6.325336 0.409594 1.644345
H -5.638682 -1.200378 1.597038
H -6.999887 -0.807101 0.514271
H -0.131001 -5.344255 -0.905478
H 2.084106 -4.266923 -0.819522
H -1.741651 -5.027475 0.950802
H -0.416336 -0.984701 -4.218316
H -1.051337 -2.655868 -4.148182
H 0.652699 -2.314718 -3.778877

84

6-c22.out delta G = 1.4753 kcal/mol

conformer 13

O -0.126857 -0.635341 -1.718034
C -0.653497 -1.755051 -2.225092
C 2.540613 2.305554 -0.696045
O 0.531829 1.160203 2.303455
C 4.176233 3.037685 0.919186
C 1.269067 0.507160 1.572390
N 2.289587 -0.250022 2.004647
C 4.330906 3.259816 2.408860
O 2.869726 2.531079 0.679826
C 3.070221 -0.947031 1.006883
O 0.092059 3.766481 -1.144469
O -3.871214 0.710994 1.706425
C 2.380299 -0.544279 -0.335667
C 3.391365 0.067613 -1.280846
O -2.824034 -1.466401 0.455798
C 3.508517 1.391642 -1.430404
O -5.380473 -0.230270 -1.396251
C 1.155950 1.679412 -0.731163
C 3.146989 -2.463108 1.278426
C 1.153515 0.341947 0.037100
C 4.240145 -0.962567 -1.972572
C 4.472122 2.086779 -2.350601
C 0.001543 2.587435 -0.321455
C -1.342315 1.878672 -0.555319
C -2.516841 2.709599 -0.038934
C -3.871572 2.049339 -0.299897
C -3.909134 0.623792 0.283098
C -5.193394 -0.087714 -0.203141
C -2.715600 -0.206338 -0.214273
C -0.176044 -0.375388 -0.278731
C -1.384024 0.483594 0.086933
C 0.301678 -4.660936 0.035126
C 1.520207 -3.995865 0.114299

C 1.830513 -3.189928 1.212324
C -5.023630 2.911187 0.217861
C -6.168717 -0.579564 0.825515
C -0.340692 -3.722959 2.152344
C 0.883602 -3.067910 2.233597
C -0.635502 -4.522879 1.052562
C -0.297025 -1.915951 -3.672101
O -1.319973 -2.545628 -1.586834
H 2.510761 3.272074 -1.207804
H 4.924951 2.322627 0.560670
H 4.318286 3.976087 0.366720
H 2.545364 -0.241145 2.980779
H 4.194196 2.321842 2.949688
H 3.594720 3.978780 2.772389
H 5.328111 3.644149 2.631374
H 4.093613 -0.564586 1.017490
H -0.400356 4.473395 -0.714833
H -3.591314 -0.153945 2.036799
H 2.011805 -1.454614 -0.805965
H -2.283634 -2.099438 -0.049339
H 0.982967 1.430762 -1.780279
H 3.840123 -2.894441 0.554396
H 3.601034 -2.599118 2.263852
H 3.605510 -1.645978 -2.545204
H 4.779801 -1.577169 -1.245364
H 4.974705 -0.534221 -2.648723
H 3.923273 2.641180 -3.118836
H 5.068981 2.824075 -1.807030
H 5.157436 1.409168 -2.853613
H 0.095904 2.867390 0.727062
H -1.443991 1.764089 -1.641053
H -2.522087 3.690723 -0.519521
H -2.393967 2.876185 1.035440
H -3.988253 1.926234 -1.379697
H -2.841096 -0.343522 -1.290685
H -0.218416 -1.335738 0.231091
H -1.326303 0.610921 1.167805
H 0.079100 -5.274983 -0.827888
H 2.244571 -4.106807 -0.684301
H -4.966747 3.026563 1.300787
H -5.996435 2.480414 -0.028009
H -4.979288 3.903943 -0.233205
H -7.049356 -0.987168 0.333876
H -6.445049 0.226247 1.506613
H -5.693850 -1.353827 1.433299
H -1.063880 -3.609854 2.950383
H 1.105248 -2.458799 3.099676
H -1.589816 -5.029976 0.987422
H 0.761772 -2.173298 -3.746002
H -0.447462 -0.979044 -4.207318
H -0.894102 -2.707701 -4.115550
84
6-c49.out delta G = 1.5456 kcal/mol
conformer 14
O -0.815594 -0.680928 -1.360066
C -1.066014 -1.972439 -1.645952
C 2.448534 1.888097 -0.504665
O 0.277536 1.423931 2.426397
C 2.799272 3.711611 1.066297
C 0.832380 0.527481 1.799875
N 1.601565 -0.429722 2.344976
C 4.091845 4.387823 0.638677
O 2.818652 2.300684 0.821574
C 2.115676 -1.450386 1.452967
O 0.266969 3.696459 -1.257091
O -4.158615 1.807376 1.616671
C 1.746817 -0.899084 0.037322
C 3.000978 -0.511217 -0.722087
O -3.492290 -0.770290 0.839176
C 3.358754 0.761250 -0.927532
O -6.473423 1.000257 0.835346
C 0.985921 1.458516 -0.601307
C 1.519717 -2.834705 1.776195
C 0.711697 0.234839 0.286307
C 3.795822 -1.700677 -1.190088
C 4.639708 1.218547 -1.565437
C -0.033354 2.585222 -0.398293
C -1.462159 2.085332 -0.635564
C -2.499246 3.177134 -0.383497
C -3.923771 2.690119 -0.652998
C -4.224047 1.458393 0.237352
C -5.633994 0.915940 -0.043389
C -3.186288 0.342588 0.000631
C -0.733401 -0.252793 0.029605
C -1.763079 0.854752 0.232863
C 2.304754 -5.485715 -0.842901
C 1.625144 -4.516819 -0.109305
C 2.208080 -3.940086 1.019361

C -4.949325 3.805320 -0.459683
C -5.932676 0.291621 -1.372139
C 4.162470 -5.333415 0.677291
C 3.481127 -4.366790 1.407359
C 3.577291 -5.892933 -0.456311
C -1.097871 -2.192486 -3.129994
O -1.217162 -2.834614 -0.809584
H 2.597821 2.726729 -1.188579
H 1.945859 4.172700 0.565193
H 2.652462 3.816744 2.142526
H 1.769552 -0.447089 3.340177
H 4.062009 5.449415 0.896043
H 4.243843 4.308851 -0.439537
H 4.948243 3.934778 1.141549
H 3.199428 -1.506560 1.561894
H 0.181591 3.402450 -2.173469
H -5.073477 1.749589 1.944041
H 1.277570 -1.708231 -0.518914
H -3.577924 -0.416633 1.736620
H 0.856570 1.114372 -1.633540
H 1.627369 -3.002602 2.850929
H 0.452060 -2.825965 1.552232
H 4.174256 -2.291999 -0.353076
H 4.640419 -1.425085 -1.815290
H 3.152344 -2.371612 -1.765858
H 5.352773 0.416496 -1.737577
H 4.442476 1.712197 -2.522695
H 5.122230 1.960683 -0.923767
H 0.042085 2.978875 0.612644
H -1.528135 1.782754 -1.688773
H -2.291182 4.035685 -1.022580
H -2.424649 3.519855 0.652578
H -3.966205 2.353067 -1.693084
H -3.278548 -0.019273 -1.023135
H -0.970929 -1.107916 0.656051
H -1.690462 1.156235 1.278292
H 1.838732 -5.921171 -1.718410
H 0.635098 -4.203043 -0.414288
H -5.957202 3.490155 -0.737137
H -4.688060 4.664057 -1.079736
H -4.970781 4.132860 0.580898
H -5.555501 -0.734875 -1.360113
H -5.439041 0.810774 -2.192401
H -7.007873 0.265731 -1.534437
H 5.149155 -5.650686 0.991606
H 3.944014 -3.933907 2.287087
H 4.107069 -6.644753 -1.027812
H -1.493116 -3.180648 -3.348276
H -0.079965 -2.116774 -3.518479
H -1.697215 -1.425196 -3.618662

84

6-c43.out delta G = 1.6842 kcal/mol

conformer 15

O -0.629001 -0.789566 -1.307542
C -1.214964 -1.972473 -1.521151
C 2.460734 1.993886 -0.686006
O 0.333035 1.578604 2.351648
C 4.010673 3.063200 0.824945
C 0.941976 0.700482 1.753075
N 1.798255 -0.167767 2.323973
C 4.052906 3.698784 2.198311
O 2.695382 2.566706 0.606608
C 2.270572 -1.255252 1.489250
O 0.172433 3.654098 -1.490211
O -4.188818 1.708547 1.552491
C 1.878892 -0.783748 0.055783
C 3.103313 -0.394756 -0.741196
O -3.399129 -0.790378 0.840267
C 3.407738 0.869301 -1.053457
O -5.677719 0.328426 -1.391243
C 1.019926 1.507834 -0.711083
C 1.643245 -2.600618 1.911048
C 0.812620 0.327448 0.254861
C 3.949858 -1.565853 -1.159234
C 4.640270 1.243302 -1.846211
C -0.024648 2.612537 -0.516620
C -1.436682 2.062976 -0.719601
C -2.503588 3.122830 -0.448896
C -3.921165 2.596939 -0.674330
C -4.179838 1.336725 0.175571
C -5.530345 0.712369 -0.246946
C -3.092269 0.278218 -0.063464
C -0.611842 -0.228262 0.042342
C -1.690158 0.842126 0.176731
C 1.781688 -5.280409 -0.788069
C 1.308604 -4.279080 0.055207
C 2.120146 -3.744472 1.057179
C -4.968148 3.682989 -0.424260

C -6.618079 0.608601 0.781167
C 3.889404 -5.251296 0.370676
C 3.412977 -4.250753 1.209630
C 3.076747 -5.765263 -0.636924
C -0.963200 -2.474442 -2.908968
O -1.845948 -2.577171 -0.672444
H 2.576361 2.784007 -1.437596
H 4.735706 2.244394 0.761507
H 4.265641 3.795734 0.047919
H 1.954682 -0.144551 3.321095
H 3.343676 4.525563 2.264987
H 5.053116 4.084267 2.404708
H 3.801119 2.964751 2.965694
H 3.354037 -1.337259 1.576931
H 0.836139 4.262078 -1.147797
H -4.054098 0.900624 2.066929
H 1.434046 -1.628395 -0.466439
H -2.934085 -1.584032 0.525513
H 0.864573 1.112686 -1.718206
H 1.902726 -2.778826 2.957674
H 0.555867 -2.519578 1.860893
H 4.722615 -1.285465 -1.870548
H 3.326327 -2.340059 -1.614288
H 4.438338 -2.035605 -0.302021
H 5.555851 0.866604 -1.386294
H 4.599914 0.844488 -2.863849
H 4.734941 2.325357 -1.929142
H 0.060248 3.030198 0.485546
H -1.511529 1.748771 -1.767906
H -2.338088 3.981791 -1.099517
H -2.406731 3.474297 0.582736
H -4.003558 2.269896 -1.714350
H -3.192540 -0.064440 -1.095976
H -0.802468 -1.040690 0.739406
H -1.645577 1.174300 1.214159
H 1.137390 -5.679996 -1.561391
H 0.294598 -3.916519 -0.060883
H -4.765696 4.547666 -1.058384
H -4.947541 4.011785 0.615362
H -5.978212 3.336351 -0.652155
H -7.529035 0.234340 0.319222
H -6.792086 1.577311 1.251001
H -6.301196 -0.071476 1.575955
H 4.895640 -5.629758 0.501349
H 4.053645 -3.853437 1.988558
H 3.448477 -6.542295 -1.292908
H -1.715131 -3.211328 -3.177769
H 0.020328 -2.950731 -2.926075
H -0.951229 -1.653787 -3.623536
84
6-c2.out delta G = 1.9377 kcal/mol
conformer 16
O -0.096292 -0.642434 -1.712172
C -0.663256 -1.717026 -2.271838
C 2.662096 2.186173 -0.561873
O 0.653549 0.984974 2.374292
C 2.956799 3.743664 1.278919
C 1.349681 0.316926 1.616387
N 2.336687 -0.501870 2.011536
C 4.105704 4.644846 0.856096
O 3.105588 2.406129 0.788534
C 3.069010 -1.206361 0.981471
O 0.206995 3.772828 -0.849846
O -3.812127 0.651231 1.770951
C 2.412009 -0.692349 -0.341819
C 3.478684 -0.050018 -1.203615
O -2.829680 -1.485300 0.420437
C 3.645569 1.275625 -1.260179
O -5.325265 -0.074098 -1.386866
C 1.262430 1.583504 -0.622366
C 3.057057 -2.733911 1.180433
C 1.218390 0.214037 0.078749
C 4.356063 -1.059151 -1.892006
C 4.759763 1.992783 -1.966160
C 0.124290 2.505552 -0.178520
C -1.238580 1.850395 -0.431130
C -2.378174 2.694438 0.137481
C -3.753905 2.100310 -0.157953
C -3.842225 0.644847 0.344789
C -5.145606 0.004917 -0.186717
C -2.673505 -0.199859 -0.188744
C -0.136065 -0.447846 -0.262009
C -1.318989 0.427857 0.145135
C -0.478596 -3.866307 2.033878
C 0.773053 -3.267482 2.127780
C 1.704721 -3.388983 1.092470
C -4.875379 2.970618 0.409952
C -6.148904 -0.498429 0.808976

C 0.104667 -4.746155 -0.125017
C 1.350575 -4.135559 -0.033506
C -0.816268 -4.609983 0.907170
C -0.298802 -1.830352 -3.721324
O -1.366060 -2.507353 -1.674124
H 2.641505 3.143154 -1.087022
H 2.003094 4.163136 0.951281
H 2.926256 3.651044 2.365779
H 2.599364 -0.544306 2.985184
H 4.140023 4.764177 -0.228532
H 5.060209 4.232184 1.187884
H 3.985479 5.636747 1.298381
H 4.113626 -0.889036 1.010400
H 0.083224 3.622638 -1.796217
H -3.565559 -0.240595 2.052972
H 2.022426 -1.552915 -0.883465
H -2.314894 -2.113906 -0.115695
H 1.091540 1.381986 -1.684329
H 3.721617 -3.166465 0.430688
H 3.508796 -2.942476 2.154189
H 5.096828 -0.608057 -2.546549
H 3.740983 -1.736886 -2.491656
H 4.889472 -1.682744 -1.167819
H 5.504947 1.327225 -2.394436
H 4.366548 2.626474 -2.767574
H 5.270916 2.658277 -1.264322
H 0.218297 2.739998 0.878456
H -1.361228 1.782316 -1.521072
H -2.332047 3.701530 -0.278903
H -2.246625 2.787105 1.219668
H -3.876153 2.042338 -1.242904
H -2.799579 -0.281269 -1.270652
H -0.203483 -1.428731 0.203731
H -1.257669 0.505119 1.230865
H -1.188936 -3.755274 2.843633
H 1.029519 -2.705374 3.015897
H -4.791689 3.986807 0.021011
H -4.818500 3.017035 1.498010
H -5.862501 2.592466 0.136183
H -5.706567 -1.312417 1.388801
H -7.036449 -0.855121 0.290935
H -6.407194 0.286619 1.520932
H -0.151079 -5.316616 -1.008477
H 2.062641 -4.245193 -0.843259
H -1.790935 -5.075494 0.833429
H -0.366571 -0.859418 -4.210185
H -0.946939 -2.551217 -4.211634
H 0.737721 -2.167331 -3.793936
84
6-c23.out delta G = 1.9534 kcal/mol
conformer 17
O -0.564422 -0.207951 -2.115006
C -1.161572 -1.208444 -2.775257
C 2.561658 2.070113 -0.409071
O 0.426688 0.548259 2.175467
C 2.888436 3.234975 1.700703
C 1.010248 -0.051688 1.280140
N 1.820460 -1.108799 1.459909
C 4.166218 4.041023 1.534280
O 2.928794 1.998674 0.978287
C 2.333852 -1.750400 0.265469
O 0.306315 3.956589 -0.461759
O -4.105935 0.956386 1.519121
C 1.947235 -0.749056 -0.866271
C 3.187993 -0.090820 -1.432356
O -3.326295 -1.063290 -0.125871
C 3.502875 1.186974 -1.194711
O -5.609899 0.851129 -1.723661
C 1.110077 1.659201 -0.650811
C 1.749954 -3.158890 0.058136
C 0.885175 0.196242 -0.242988
C 4.011359 -1.038575 -2.262573
C 4.761486 1.877069 -1.636586
C 0.058133 2.601893 -0.056150
C -1.359862 2.168982 -0.446558
C -2.420925 3.041125 0.222809
C -3.842533 2.648875 -0.179623
C -4.103302 1.155914 0.107146
C -5.457633 0.752168 -0.521372
C -3.020441 0.275211 -0.535009
C -0.539104 -0.230336 -0.654409
C -1.615613 0.695828 -0.099734
C 3.629502 -5.513117 2.379019
C 3.310677 -4.709028 1.290420
C 2.065503 -4.080534 1.205636
C -4.881459 3.550319 0.488288
C -6.542377 0.253762 0.387301
C 1.462269 -5.079840 3.330447

C 1.146308 -4.276796 2.238047
C 2.705557 -5.699827 3.404417
C -0.951117 -1.093612 -4.253622
O -1.783238 -2.101207 -2.228991
H 2.682371 3.099077 -0.754894
H 2.023152 3.825564 1.393274
H 2.749142 2.954264 2.745992
H 1.982406 -1.480469 2.384083
H 4.122732 4.942778 2.149898
H 4.309611 4.350586 0.497232
H 5.033966 3.454645 1.841966
H 3.418240 -1.834473 0.345480
H 0.212258 4.008075 -1.421914
H -3.975401 0.010660 1.674128
H 1.493695 -1.319219 -1.677124
H -2.859466 -1.666834 -0.728729
H 0.977867 1.696898 -1.738045
H 0.668836 -3.081311 -0.073210
H 2.163927 -3.555223 -0.871716
H 4.300878 -1.925487 -1.691924
H 4.918619 -0.588778 -2.655578
H 3.418025 -1.395730 -3.110411
H 5.482717 1.213879 -2.107205
H 4.532681 2.684412 -2.339644
H 5.248132 2.341272 -0.774477
H 0.139315 2.610939 1.028142
H -1.446283 2.283528 -1.535438
H -2.255953 4.086747 -0.039516
H -2.315390 2.961875 1.308913
H -3.934227 2.758394 -1.263617
H -3.127324 0.367995 -1.618385
H -0.724582 -1.253810 -0.337412
H -1.564203 0.591942 0.984314
H 4.597633 -5.996146 2.426724
H 4.033916 -4.568685 0.495220
H -4.676194 4.595115 0.249427
H -4.853812 3.437579 1.572628
H -5.894578 3.327052 0.147786
H -6.716153 0.962441 1.197983
H -6.223204 -0.681692 0.853763
H -7.454345 0.088158 -0.182079
H 0.736494 -5.222452 4.121469
H 0.175786 -3.797794 2.183813
H 2.952351 -6.326538 4.252131
H -1.166991 -0.079044 -4.587320
H -1.583097 -1.807179 -4.774272
H 0.096972 -1.300200 -4.479710
84
6-c19.out delta G = 2.0375 kcal/mol
conformer 18
O -0.605986 -0.785250 -1.294014
C -1.218630 -1.946763 -1.549931
C 2.547526 1.926181 -0.501085
O 0.385169 1.468509 2.440337
C 2.861631 3.800761 1.019310
C 0.958066 0.575695 1.826763
N 1.750282 -0.358191 2.383212
C 4.151283 4.477415 0.583521
O 2.895802 2.384203 0.816756
C 2.227163 -1.413290 1.509632
O 0.376373 3.595818 -1.366527
O -4.147430 1.700932 1.599768
C 1.887849 -0.866246 0.088022
C 3.152131 -0.468350 -0.646537
O -3.391862 -0.807117 0.843715
C 3.486097 0.805984 -0.878032
O -5.635054 0.405469 -1.383380
C 1.093651 1.465587 -0.597179
C 1.569201 -2.765840 1.852841
C 0.841597 0.257644 0.315919
C 3.986943 -1.651197 -1.062388
C 4.772986 1.270135 -1.498365
C 0.055388 2.569285 -0.409685
C -1.365978 2.040946 -0.641514
C -2.420872 3.114125 -0.371891
C -3.847193 2.616054 -0.611033
C -4.132745 1.348534 0.218259
C -5.487718 0.749237 -0.226515
C -3.060384 0.277308 -0.031496
C -0.588913 -0.269505 0.073062
C -1.651685 0.812143 0.232256
C 1.809848 -5.361452 -0.920943
C 1.305795 -4.382901 -0.068878
C 2.075577 -3.885191 0.983388
C -4.875465 3.716881 -0.349022
C -6.577154 0.612806 0.795935
C 3.863503 -5.385653 0.331481
C 3.356270 -4.407699 1.178704

C 3.093529 -5.861037 -0.727343
C -0.990683 -2.391975 -2.961088
O -1.859230 -2.569070 -0.721601
H 2.684661 2.746087 -1.208596
H 2.008171 4.238854 0.497713
H 2.703777 3.936354 2.090580
H 1.902079 -0.371200 3.381141
H 4.108437 5.545783 0.808994
H 4.314383 4.367784 -0.490318
H 5.007074 4.048212 1.107845
H 3.306198 -1.516200 1.624905
H -0.109815 4.393576 -1.131908
H -4.036035 0.884241 2.105659
H 1.436718 -1.672851 -0.485867
H -2.938771 -1.600078 0.510432
H 0.973037 1.105326 -1.623390
H 1.779734 -2.983090 2.903148
H 0.485895 -2.673067 1.756975
H 4.779973 -1.382995 -1.755077
H 3.354873 -2.401881 -1.544206
H 4.445861 -2.147744 -0.204116
H 4.587941 1.750180 -2.464811
H 5.235377 2.024961 -0.856741
H 5.497628 0.473641 -1.646531
H 0.124800 2.973172 0.600985
H -1.426561 1.743135 -1.694577
H -2.255796 3.977233 -1.021078
H -2.325500 3.462566 0.660929
H -3.928841 2.306532 -1.656069
H -3.157632 -0.039422 -1.072583
H -0.795744 -1.101570 0.741489
H -1.608676 1.120178 1.277378
H 1.198436 -5.732564 -1.734118
H 0.300080 -4.009826 -0.217553
H -5.890089 3.388712 -0.583248
H -4.658092 4.586266 -0.971668
H -4.852056 4.031469 0.694854
H -6.257759 -0.083231 1.575669
H -7.483300 0.242447 0.321666
H -6.762478 1.568641 1.287686
H 4.859824 -5.776926 0.495921
H 3.962961 -4.040843 1.998739
H 3.488803 -6.620653 -1.389937
H -1.670319 -3.202628 -3.207511
H 0.039585 -2.742188 -3.055487
H -1.121401 -1.558183 -3.649573
84
6-c38.out delta G = 2.0658 kcal/mol
conformer 19
O -0.094429 -0.644958 -1.710096
C -0.670947 -1.717905 -2.262705
C 2.675842 2.184036 -0.559658
O 0.667720 0.980443 2.376061
C 2.976407 3.725046 1.294942
C 1.359556 0.312908 1.613669
N 2.340667 -0.514979 2.004814
C 4.126351 4.627094 0.876703
O 3.119359 2.391957 0.793610
C 3.072312 -1.214644 0.971098
O 0.276597 3.718778 -0.948960
O -3.784815 0.699486 1.778376
C 2.416291 -0.692759 -0.349473
C 3.485292 -0.051812 -1.209444
O -2.819114 -1.469389 0.448571
C 3.656588 1.273344 -1.261387
O -5.331936 -0.088455 -1.347993
C 1.274461 1.585263 -0.626087
C 3.059914 -2.743136 1.161847
C 1.226041 0.216647 0.075313
C 4.358910 -1.061738 -1.901456
C 4.772710 1.989467 -1.965296
C 0.140412 2.505256 -0.184755
C -1.223492 1.853945 -0.450631
C -2.368206 2.706783 0.096977
C -3.745107 2.105274 -0.181761
C -3.829428 0.661948 0.352991
C -5.138062 0.010877 -0.151666
C -2.666010 -0.191129 -0.176415
C -0.130629 -0.442630 -0.261210
C -1.309851 0.439377 0.143541
C -0.476006 -3.885635 2.001626
C 0.773656 -3.283306 2.100501
C 1.708555 -3.399452 1.067558
C -4.864642 2.987567 0.371064
C -6.127885 -0.477771 0.864505
C 0.115679 -4.757822 -0.158059
C 1.359446 -4.143750 -0.061582
C -0.808346 -4.627423 0.872165

C -0.312864 -1.839937 -3.713110
O -1.377212 -2.500670 -1.659027
H 2.657496 3.143792 -1.077977
H 2.022878 4.150540 0.973736
H 2.947800 3.624108 2.381138
H 2.603210 -0.563313 2.978228
H 4.158257 4.755028 -0.206974
H 5.080481 4.209102 1.202822
H 4.009915 5.615593 1.327545
H 4.117245 -0.898363 1.001807
H -0.255944 4.402908 -0.529623
H -3.534846 -0.185314 2.078658
H 2.022870 -1.549640 -0.894083
H -2.311729 -2.104351 -0.087019
H 1.103994 1.389490 -1.687743
H 3.726265 -3.171473 0.411288
H 3.509825 -2.956879 2.135360
H 3.741347 -1.735915 -2.502575
H 4.891175 -1.689008 -1.179593
H 5.100402 -0.610866 -2.555371
H 5.516798 1.323102 -2.394342
H 4.381183 2.625398 -2.765734
H 5.284758 2.652656 -1.261899
H 0.229699 2.730969 0.877694
H -1.327301 1.780990 -1.539633
H -2.342391 3.703711 -0.349558
H -2.238603 2.832024 1.176174
H -3.870055 2.023430 -1.264647
H -2.798761 -0.285022 -1.256367
H -0.200156 -1.420844 0.209820
H -1.243309 0.526501 1.228266
H -1.188830 -3.778903 2.809781
H 1.026076 -2.722952 2.990896
H -4.782217 3.995852 -0.038207
H -4.805534 3.055340 1.457870
H -5.852016 2.603821 0.106589
H -5.676130 -1.279333 1.454287
H -7.019784 -0.847157 0.363129
H -6.381429 0.319502 1.564548
H -0.136098 -5.326521 -1.043830
H 2.073970 -4.248990 -0.869749
H -1.781402 -5.095647 0.794467
H -0.381008 -0.871423 -4.206750
H -0.964667 -2.562022 -4.196729
H 0.722692 -2.179125 -3.788678
84
6-c45.out delta G = 2.6211 kcal/mol
conformer 20
O -0.607301 -0.757943 -1.291268
C -1.192150 -1.929572 -1.563261
C 2.472697 1.986936 -0.520712
O 0.283515 1.478287 2.469667
C 3.970675 3.055747 1.079513
C 0.898950 0.615804 1.854799
N 1.732332 -0.280879 2.415230
C 4.224341 4.400275 0.414002
O 2.680330 2.517207 0.797857
C 2.225072 -1.336719 1.552261
O 0.210280 3.686115 -1.292636
O -4.239135 1.627896 1.578060
C 1.870183 -0.811006 0.128311
C 3.113924 -0.399978 -0.626902
O -3.427972 -0.833112 0.774892
C 3.424629 0.871895 -0.900225
O -5.637603 0.411030 -1.479973
C 1.031341 1.505953 -0.580204
C 1.587708 -2.695994 1.909267
C 0.803235 0.296429 0.342115
C 3.973520 -1.559193 -1.052497
C 4.673428 1.260611 -1.660800
C -0.009513 2.609772 -0.362483
C -1.420196 2.077026 -0.609928
C -2.486715 3.129834 -0.312240
C -3.901627 2.624152 -0.592654
C -4.187199 1.324263 0.185701
C -5.523065 0.723574 -0.310406
C -3.094391 0.273949 -0.071203
C -0.618413 -0.247609 0.078629
C -1.697308 0.821079 0.229292
C 1.796440 -5.291126 -0.866293
C 1.300100 -4.319246 -0.002237
C 2.088586 -3.810760 1.031053
C -4.947881 3.702751 -0.309142
C -6.637509 0.548222 0.678919
C 3.879934 -5.287142 0.336192
C 3.380545 -4.315495 1.195376
C 3.091139 -5.773161 -0.703777
C -0.921811 -2.370422 -2.968234

O -1.839276 -2.566593 -0.751055
H 2.591137 2.791111 -1.253159
H 3.994022 3.170305 2.164149
H 4.748182 2.333751 0.811384
H 1.865471 -0.294471 3.415903
H 3.454294 5.119256 0.700117
H 4.237002 4.325983 -0.674176
H 5.193857 4.791404 0.730259
H 3.306085 -1.422407 1.664089
H 0.865908 4.280497 -0.912652
H -4.122611 0.795016 2.055812
H 1.434362 -1.634063 -0.434262
H -2.952162 -1.612546 0.440621
H 0.887872 1.144403 -1.601614
H 1.818020 -2.909536 2.956178
H 0.502162 -2.613299 1.831148
H 4.732490 -1.268151 -1.774565
H 3.358298 -2.346582 -1.495335
H 4.483584 -2.014963 -0.199912
H 4.677561 0.840588 -2.670430
H 4.747066 2.342186 -1.764386
H 5.582547 0.915105 -1.163991
H 0.061463 2.988349 0.655892
H -1.479080 1.807652 -1.671772
H -2.302646 4.017167 -0.918398
H -2.409053 3.433036 0.736284
H -3.962943 2.351353 -1.649501
H -3.168529 -0.021650 -1.120451
H -0.827258 -1.084708 0.740332
H -1.679129 1.109243 1.280757
H 1.170450 -5.670742 -1.664315
H 0.286180 -3.959267 -0.126199
H -4.729191 4.596541 -0.895790
H -4.945165 3.979556 0.745732
H -5.955308 3.373813 -0.572480
H -6.334428 -0.173454 1.441697
H -7.529870 0.191264 0.169478
H -6.838587 1.485560 1.199032
H 4.885172 -5.664854 0.476259
H 4.002298 -3.939441 1.999691
H 3.480660 -6.527494 -1.375742
H -0.974336 -1.526216 -3.653658
H -1.627842 -3.144638 -3.255134
H 0.091766 -2.775868 -3.014240

84
6-c27.out delta G = 2.8746 kcal/mol
conformer 21
O -0.602214 -0.778296 -1.307312
C -1.221985 -1.936602 -1.560118
C 2.574764 1.906888 -0.548772
O 0.427411 1.448743 2.422023
C 3.545509 3.631102 0.882187
C 1.001115 0.562846 1.800443
N 1.809329 -0.364970 2.344869
C 2.559847 4.787633 0.921956
O 2.910921 2.355322 0.777406
C 2.271964 -1.422317 1.466025
O 0.390022 3.550139 -1.458385
O -4.084289 1.703438 1.666712
C 1.909485 -0.879326 0.048578
C 3.160299 -0.491976 -0.716268
O -3.358904 -0.797759 0.869283
C 3.496227 0.779907 -0.960987
O -5.628523 0.456693 -1.308339
C 1.120385 1.454204 -0.626945
C 1.614107 -2.771729 1.822707
C 0.870173 0.248434 0.290398
C 3.976938 -1.684124 -1.141538
C 4.767504 1.232339 -1.622423
C 0.094041 2.567783 -0.446675
C -1.338928 2.047677 -0.625512
C -2.376584 3.127490 -0.317772
C -3.812064 2.646744 -0.535962
C -4.094370 1.369707 0.280454
C -5.461135 0.787965 -0.150530
C -3.034091 0.293653 -0.000056
C -0.564526 -0.270799 0.062518
C -1.617681 0.815772 0.245335
C 1.774558 -5.379457 -0.945291
C 1.297550 -4.392499 -0.087383
C 2.094344 -3.896469 0.945483
C -4.825051 3.752731 -0.238442
C -6.537470 0.653681 0.885912
C 3.855159 -5.414661 0.261542
C 3.374997 -4.428180 1.114686
C 3.057878 -5.889114 -0.777294
C -1.007694 -2.382011 -2.973273
O -1.857158 -2.557996 -0.726867

H 2.699843 2.739448 -1.242855
H 4.119922 3.593390 1.809769
H 4.257902 3.764579 0.061354
H 1.969233 -0.380879 3.341498
H 1.852128 4.657926 1.742857
H 2.000836 4.857627 -0.010968
H 3.095868 5.727875 1.074012
H 3.352395 -1.526940 1.564187
H -0.082998 4.360936 -1.242893
H -3.968014 0.879179 2.159153
H 1.443044 -1.686287 -0.512566
H -2.912013 -1.588840 0.523228
H 0.990604 1.087815 -1.649405
H 1.842957 -2.989725 2.869055
H 0.529918 -2.671145 1.747452
H 4.757725 -1.428668 -1.852647
H 3.329686 -2.434872 -1.602581
H 4.450963 -2.176476 -0.288996
H 4.548247 1.804203 -2.529402
H 5.315670 1.904211 -0.955070
H 5.436895 0.417715 -1.886562
H 0.204484 3.014640 0.541852
H -1.435076 1.757267 -1.677980
H -2.217044 4.000314 -0.955433
H -2.255653 3.457655 0.718237
H -3.916877 2.353222 -1.583590
H -3.151824 -0.011692 -1.042505
H -0.765668 -1.106352 0.728388
H -1.555242 1.115858 1.291451
H 1.142191 -5.749550 -1.742754
H 0.291946 -4.011852 -0.216602
H -5.846896 3.437117 -0.458273
H -4.611239 4.629225 -0.852359
H -4.779316 4.051657 0.809297
H -7.454482 0.298586 0.420925
H -6.704656 1.606519 1.389854
H -6.215215 -0.053745 1.654130
H 4.851607 -5.813454 0.405924
H 4.003025 -4.062308 1.919014
H 3.431836 -6.655676 -1.444249
H -1.097952 -1.540462 -3.658389
H -1.719347 -3.162521 -3.227001
H 0.006790 -2.777396 -3.062863

**Table S26.** Geometry data of conformers of compound **7**.

75

7-c5.out delta G = 0.0000 kcal/mol

conformer 1

O 0.710019 -1.892225 -2.383351
H 1.905894 0.197048 -3.275140
C 1.074072 -0.896813 -1.758135
O 2.364286 -3.483452 -0.674113
H 1.806538 -3.096669 -1.368838
H -3.406293 0.646254 -1.574855
N 1.684217 0.172181 -2.290010
O -4.166622 -1.566817 -1.738407
C 2.290131 1.095197 -1.337072
H 3.354447 1.151752 -1.560164
C 0.914762 -0.630873 -0.247276
C 2.072460 0.353510 0.022714
H 1.807924 1.078016 0.793617
H -5.092175 -1.393768 -1.985230
O -6.346380 -0.700071 -0.679629
C 1.726401 2.523020 -1.425091
H 1.702526 2.806321 -2.480274
H 0.704403 2.551968 -1.050740
O -0.752106 2.503342 1.062107
C 3.502344 -1.662366 0.526857
C 2.327793 -2.615613 0.474657
H 2.432853 -3.294493 1.327571
C -0.483242 -0.008079 -0.037437
H -0.591669 0.927322 -0.575633
C 1.008274 -1.870810 0.652493
C 3.364893 -0.334141 0.422240
C 2.211549 3.967279 0.596396
H 1.246135 3.688329 0.997330
O -3.236610 0.835079 -0.640417
C -1.371159 -2.379227 0.058100
H 1.049291 -1.479350 1.674996
C 3.056098 4.799137 1.327586
H 2.743666 5.160297 2.299788
O -0.615288 0.271588 1.381422
C 4.518724 0.616002 0.659537
H 5.169159 0.707347 -0.214805
H 5.139502 0.287409 1.492280
H 4.152395 1.617021 0.889171
C 2.593863 3.489087 -0.657256

C -4.995860 -3.752921 0.159374
H -5.157679 -3.926751 -0.905776
H -5.933541 -3.400275 0.593026
H -4.754922 -4.709428 0.625466
C -5.602148 -0.352003 1.555655
H -5.105910 -1.021467 2.256924
H -6.658049 -0.263771 1.801159
H -5.135640 0.631993 1.656095
C 3.836932 3.872366 -1.168579
H 4.146921 3.514128 -2.143854
C 4.682279 4.701993 -0.441574
H 5.642098 4.988063 -0.853707
C 4.817809 -2.340492 0.795638
H 5.676100 -1.708152 0.579055
H 4.896937 -3.240516 0.181743
C -0.194586 -2.761545 0.545521
H -0.066258 -3.785883 0.881242
C -2.519208 -3.330291 -0.119542
H -2.627989 -3.562827 -1.184606
H -2.309297 -4.269215 0.395454
C -2.971601 -0.418291 -0.016450
H -2.960739 -0.225860 1.054376
C -0.748612 1.545323 1.801003
C -3.854463 -2.762219 0.375341
H -3.756533 -2.561097 1.445627
C 4.295520 5.165474 0.813847
H 4.953288 5.810632 1.382665
C -4.110186 -1.405108 -0.325667
C -5.447193 -0.802116 0.135179
C -1.602146 -0.971667 -0.442510
H -1.614795 -1.006558 -1.537270
C -0.873901 1.600439 3.294975
H -1.032381 2.626640 3.613775
H 0.037696 1.208578 3.749131
H -1.702151 0.972531 3.624520
H 4.885971 -2.661240 1.841060
75
7-c2.out delta G = 0.8340 kcal/mol
conformer 2
O 0.852900 -1.990579 -2.320373
H 2.173645 0.019322 -3.229412
C 1.229585 -0.988274 -1.715054
O 2.378967 -3.575184 -0.489251
H 1.868884 -3.194285 -1.222613
H -2.603813 1.585304 -0.465300
N 1.913390 0.032158 -2.253677
O -4.135615 -1.473720 -1.824854
C 2.460861 0.998650 -1.309060
H 3.534217 1.067769 -1.476779
C 1.012775 -0.661727 -0.221176
C 2.183458 0.301402 0.061127
H 1.913185 1.047556 0.808557
H -3.978791 -0.620084 -2.251446
O -5.393487 -0.347274 1.329097
C 1.876200 2.409926 -1.494999
H 2.056689 2.705603 -2.531696
H 0.796188 2.398414 -1.353160
O -1.460706 2.367824 0.812108
C 3.536401 -1.739276 0.671535
C 2.335187 -2.659087 0.620389
H 2.393635 -3.304723 1.502856
C -0.365938 0.015213 -0.089204
H -0.416948 0.917393 -0.690071
C 1.034488 -1.868528 0.727621
C 3.443399 -0.411826 0.517912
C 3.832174 3.813954 -0.747470
H 4.388166 3.447513 -1.602740
O -3.136109 0.868781 -0.850919
C -1.338902 -2.320093 0.071264
H 1.056439 -1.440257 1.735670
C 4.444322 4.682519 0.148585
H 5.470232 4.988763 -0.013992
O -0.466246 0.408777 1.311211
C 4.618132 0.509957 0.761518
H 5.203523 0.189640 1.622597
H 4.275819 1.527880 0.949957
H 5.297400 0.554836 -0.094570
C 2.510049 3.404232 -0.557072
C -5.002347 -3.593666 -0.008941
H -4.824221 -4.545807 0.493429
H -5.097479 -3.786278 -1.078287
H -5.954342 -3.202430 0.354593
C -6.446024 -0.301914 -0.809332
H -6.112693 0.442155 -1.537242
H -7.303568 0.080661 -0.260324
H -6.716761 -1.193424 -1.376345
C 1.811018 3.897214 0.545162
H 0.778047 3.608994 0.692405

C 2.419950 4.767397 1.445175
H 1.861020 5.139067 2.295000
C 4.822346 -2.445847 1.002470
H 5.705480 -1.848919 0.785360
H 4.889182 -3.371173 0.426019
C -0.192038 -2.724841 0.608629
H -0.105605 -3.744042 0.971779
C -2.504892 -3.241083 -0.142121
H -2.560207 -3.493898 -1.207008
H -2.347179 -4.174843 0.400584
C -2.877203 -0.323134 -0.102674
H -2.919957 -0.115838 0.968001
C -0.967100 1.609366 1.627109
C -3.848299 -2.631324 0.271000
H -3.819310 -2.421421 1.342985
C 3.740699 5.158855 1.252228
H 4.217102 5.834358 1.951479
C -4.045624 -1.266681 -0.417389
C -5.326534 -0.602685 0.142563
C -1.512855 -0.923314 -0.474935
H -1.502131 -0.989042 -1.568163
C -0.813987 1.904836 3.086095
H 0.234683 2.135914 3.286718
H -1.081359 1.033375 3.682025
H -1.428726 2.758389 3.356844
H 4.852764 -2.727009 2.060944
75
7-c6.out delta G = 1.0210 kcal/mol conformer 3
O 0.959758 -1.066628 -2.306004
H 2.299238 1.134504 -2.305383
C 1.351768 -0.409510 -1.343144
O 2.355625 -3.341573 -1.287303
H 1.881067 -2.667171 -1.801285
H -2.365143 1.606097 0.826465
N 2.047737 0.735424 -1.412872
O -4.030858 -0.611944 -1.586758
C 2.624262 1.201835 -0.155395
H 3.696009 1.324815 -0.302593
C 1.135375 -0.721432 0.153552
C 2.341482 -0.005090 0.791271
H 2.104050 0.358621 1.792297
H -3.825968 0.331225 -1.648478
O -5.258506 -0.740365 1.770906
C 2.070420 2.554702 0.320315
H 1.009405 2.475359 0.553113
H 2.583897 2.803419 1.251573
O -1.170950 1.808024 2.278992
C 3.606437 -2.164940 0.473258
C 2.362017 -2.940163 0.094805
H 2.396287 -3.884984 0.646920
C -0.214101 -0.091145 0.550250
H -0.222074 0.971985 0.337905
C 1.098913 -2.205367 0.535694
C 3.576908 -0.884537 0.865896
C 3.514183 4.304799 -0.784009
H 4.296726 4.072367 -0.070672
O -2.932236 1.116949 0.206537
C -1.291723 -2.258479 -0.208435
H 1.140268 -2.219945 1.630546
C 3.737725 5.264775 -1.765148
H 4.691599 5.775421 -1.811826
O -0.311619 -0.255950 1.994708
C 4.804804 -0.180533 1.400944
H 5.370963 -0.824793 2.072922
H 4.525981 0.714786 1.958863
H 5.483751 0.133652 0.603314
C 2.289111 3.638503 -0.702023
C -5.011281 -3.229537 -0.728974
H -5.109635 -2.984921 -1.787406
H -5.945734 -2.967720 -0.228998
H -4.881855 -4.309371 -0.640431
C -6.298663 0.162960 -0.174681
H -7.145996 0.331366 0.486309
H -6.596258 -0.426684 -1.042489
H -5.934429 1.119957 -0.557225
C 1.291439 3.955571 -1.625813
H 0.335692 3.447911 -1.571814
C 1.511901 4.914478 -2.610149
H 0.726711 5.150025 -3.317806
C 4.861579 -2.993569 0.450488
H 4.890078 -3.679168 1.304613
H 5.770028 -2.395315 0.467221
C -0.165324 -2.891041 0.106965
H -0.125755 -3.973472 0.037724
C -2.499142 -2.968726 -0.748408
H -2.560883 -2.781928 -1.826394
H -2.389701 -4.046544 -0.616074

C -2.734707 -0.282674 0.433277
H -2.774967 -0.503883 1.501137
C -0.738769 0.769968 2.744750
C -3.814098 -2.507252 -0.111620
H -3.781345 -2.733401 0.956984
C 2.736444 5.571481 -2.682995
H 2.908503 6.319536 -3.446595
C -3.942966 -0.974584 -0.210806
C -5.196954 -0.523948 0.576361
C -1.398136 -0.753560 -0.159062
H -1.382823 -0.385040 -1.190360
C -0.602608 0.476837 4.206231
H -1.123024 1.234151 4.785222
H 0.456714 0.478566 4.470994
H -0.997373 -0.513613 4.430445
H 4.877829 -3.609189 -0.451561

75

7-c1.out delta G = 2.6029 kcal/mol conformer 4

O 0.775502 -1.211695 -2.515814
H 2.875234 0.147491 -2.990658
C 1.515004 -0.672015 -1.701860
O 2.734505 -3.180155 -1.127092
H 1.923539 -3.480288 -1.558101
H -1.931176 2.006541 0.460240
N 2.597209 0.056872 -2.024724
O -3.838324 -0.240433 -1.711748
C 3.375119 0.613463 -0.939519
H 4.394319 0.228875 -1.007130
C 1.370693 -0.653864 -0.159806
C 2.685737 0.011862 0.330304
H 2.451089 0.822333 1.020559
H -3.552786 0.674593 -1.843021
O -4.944549 -0.051980 1.684169
C 3.481589 2.148751 -0.996571
H 4.145837 2.458669 -0.187778
H 3.988227 2.404745 -1.931338
O -0.856065 2.144764 1.938371
C 3.632889 -2.246360 0.894689
C 2.434861 -2.909961 0.255494
H 2.262230 -3.865935 0.765991
C 0.107157 0.149414 0.223242
H 0.189798 1.188193 -0.086148
C 1.197037 -2.050101 0.459321
C 3.683605 -0.913352 1.006246
C 1.844874 3.589367 0.266490
H 2.531172 3.581357 1.105183
O -2.554243 1.518052 -0.107606
C -1.179277 -1.990553 -0.284932
H 1.168773 -1.892862 1.541438
C 0.645608 4.286998 0.364257
H 0.400156 4.807177 1.280732
O 0.100052 0.116388 1.685891
C 4.760207 -0.160261 1.738916
H 4.307161 0.457203 2.520715
H 5.295845 0.523897 1.074569
H 5.493987 -0.809013 2.209024
C 2.182129 2.900904 -0.900231
C -4.969429 -2.714466 -0.647495
H -5.866648 -2.345704 -0.146860
H -4.918558 -3.791721 -0.480844
H -5.080241 -2.541377 -1.718693
C -5.990133 0.790262 -0.285203
H -6.801055 1.057490 0.388787
H -6.355455 0.169307 -1.103784
H -5.575965 1.694186 -0.739137
C 1.284667 2.931196 -1.972736
H 1.531014 2.416906 -2.892094
C 0.078815 3.615752 -1.872705
H -0.607387 3.620232 -2.710381
C 4.670182 -3.209391 1.400483
H 4.907991 -3.934863 0.617478
H 4.288578 -3.781089 2.252820
C -0.101829 -2.687670 0.064881
H -0.150477 -3.773212 0.085456
C -2.446868 -2.639749 -0.755065
H -2.524343 -2.510833 -1.840558
H -2.413303 -3.713220 -0.560184
C -2.444163 0.133911 0.232562
H -2.462989 0.005899 1.316285
C -0.307104 1.167167 2.406436
C -3.703969 -2.042698 -0.115365
H -3.651289 -2.198480 0.965041
C -0.244709 4.296827 -0.702616
H -1.184346 4.828804 -0.624002
C -3.722796 -0.515634 -0.317657
C -4.910995 0.078024 0.475799
C -1.166197 -0.480748 -0.358582

H -1.169077 -0.216509 -1.420009
C 0.019125 0.990265 3.858637
H -0.194343 -0.028521 4.178726
H -0.543388 1.704155 4.453780
H 1.087899 1.166287 3.999467
H 5.598247 -2.730747 1.702862
75
7-c10.out delta G = 3.3402 kcal/mol conformer 5
O 0.836219 -1.118404 -2.437218
H 2.995604 0.162048 -2.844041
C 1.566441 -0.605153 -1.597979
O 2.729639 -3.158238 -1.093399
H 1.897343 -3.378059 -1.531921
H -2.323028 1.885358 -0.588318
N 2.699763 0.057669 -1.885149
O -3.616395 -0.028197 -1.739500
C 3.350556 0.740891 -0.790707
H 4.413690 0.499097 -0.804208
C 1.385542 -0.596564 -0.054841
C 2.689137 0.069150 0.459673
H 2.454107 0.844307 1.187432
H -4.460838 0.422432 -1.914163
O -5.702939 0.796455 -0.474307
C 3.256014 2.278849 -0.910842
H 3.734825 2.707546 -0.028421
H 3.874242 2.562641 -1.767816
O 0.680262 2.019157 2.402635
C 3.651143 -2.212178 0.919808
C 2.444994 -2.873666 0.289507
H 2.277547 -3.827292 0.807231
C 0.114526 0.174586 0.359156
H 0.204531 1.239173 0.172365
C 1.207049 -2.018354 0.504976
C 3.702426 -0.882268 1.074215
C 1.136082 2.671824 -2.249840
H 1.557180 2.101955 -3.066123
O -2.421937 1.582980 0.325161
C -1.150469 -1.916309 -0.313012
H 1.157567 -1.906983 1.591994
C -0.140981 3.203897 -2.387222
H -0.699575 3.029782 -3.298454
O -0.072032 -0.022222 1.794747
C 4.780343 -0.157495 1.834651
H 4.331884 0.372252 2.681197
H 5.268221 0.602743 1.218592
H 5.551785 -0.816147 2.223521
C 1.878150 2.869761 -1.079235
C -4.940086 -2.565040 -0.836762
H -5.849719 -2.231852 -0.333123
H -4.899339 -3.652759 -0.761308
H -5.019332 -2.300560 -1.892472
C -5.120988 0.136698 1.738112
H -4.501058 0.901191 2.214540
H -4.815430 -0.828005 2.140438
H -6.163758 0.332650 1.977419
C 1.314019 3.647799 -0.067934
H 1.866677 3.808184 0.847348
C 0.042523 4.193868 -0.205526
H -0.377283 4.784892 0.598576
C 4.699918 -3.185436 1.383178
H 4.920893 -3.894826 0.580899
H 4.336852 -3.774303 2.231991
C -0.089297 -2.632289 0.053642
H -0.147239 -3.717641 0.037927
C -2.411515 -2.534115 -0.843491
H -2.455585 -2.362683 -1.924894
H -2.396788 -3.614425 -0.688882
C -2.409013 0.159538 0.327886
H -2.438413 -0.128320 1.376612
C 0.185120 0.952486 2.682607
C -3.687120 -1.946778 -0.222448
H -3.669220 -2.169901 0.847742
C -0.694758 3.966862 -1.362344
H -1.686920 4.387910 -1.469469
C -3.659878 -0.403023 -0.368733
C -4.913747 0.225242 0.256838
C -1.131479 -0.403200 -0.309852
H -1.123091 -0.070717 -1.351542
C -0.230893 0.525175 4.062190
H -1.318249 0.437021 4.100550
H 0.100514 1.258904 4.791709
H 0.187766 -0.453974 4.295236
H 5.634303 -2.712972 1.675318

**Table S27.** Geometry data of conformers of compound **8**.

76

8-c3.out delta G = 0.0000 kcal/mol

conformer 1

O -0.268690 -0.709230 -1.312482
C 0.076437 -1.983247 -1.590271
C 1.128944 3.210845 -0.554247
H 3.200254 4.896693 -0.411491
O -0.218199 1.943037 2.359014
C 0.610624 1.266998 1.763586
N 1.687347 0.680228 2.316123
O 0.334643 -2.809807 -0.745688
O 0.738044 4.376045 -0.522690
C 2.616442 0.035815 1.403748
H 3.608226 0.454601 1.570210
H -5.046455 -0.715860 2.220244
O -1.665541 3.424396 -1.481534
H -1.074119 4.178178 -1.333187
O -4.309971 -0.213500 1.829319
H 1.879947 0.777606 3.302416
C 2.071936 0.488180 0.007003
H 2.133812 -0.345811 -0.690906
C 2.959584 1.593307 -0.510043
O -2.407561 -2.098290 1.122993
H -2.651774 -1.782102 2.005359
C 2.556127 2.874462 -0.641328
O -5.875503 -2.170323 1.242028
C 0.156263 2.053262 -0.648675
H 0.254336 1.684102 -1.676009
C 2.714798 -1.486222 1.609711
H 2.852262 -1.666079 2.678768
H 1.783892 -1.967909 1.312679
C 0.602689 0.899574 0.262061
C 4.375900 1.179067 -0.807306
H 5.064016 1.510719 -0.025150
H 4.718636 1.619366 -1.743729
H 4.463146 0.096274 -0.879387
C 3.487008 4.007896 -0.973057
H 3.427876 4.268634 -2.033939
H 4.521234 3.767264 -0.739491
C -1.309527 2.472569 -0.471273
H -1.435498 2.920567 0.516553
C -2.254084 1.282511 -0.606549
H -2.181903 0.929967 -1.641165
C -3.703858 1.676863 -0.332102
H -4.002601 2.467533 -1.020391
H -3.789043 2.079059 0.681550
C -4.659899 0.493503 -0.486757
H -4.551640 0.111697 -1.506353
C -4.231024 -0.643515 0.473884
C -5.148298 -1.865882 0.313591
C -2.769383 -1.059855 0.215396
H -2.695256 -1.491593 -0.782395
C -0.377953 -0.278657 0.070989
H -0.114039 -1.115943 0.709206
C -1.824574 0.138950 0.323222
H -1.874812 0.496919 1.353262
C 0.121605 -2.210477 -3.071913
C 4.760625 -3.244389 -1.081831
H 4.587521 -3.799160 -1.995763
C 3.676608 -2.780901 -0.340091
H 2.667513 -2.983110 -0.673857
C 3.875793 -2.064069 0.840120
C -6.114126 0.909725 -0.274207
H -6.809919 0.091620 -0.471167
H -6.368924 1.730410 -0.946291
H -6.272048 1.250062 0.750406
C -5.114179 -2.650503 -0.962344
H -4.256913 -3.328591 -0.923471
H -4.986952 -2.013202 -1.836147
H -6.021768 -3.242626 -1.055771
C 6.269387 -2.293549 0.532189
H 7.277431 -2.104521 0.879898
C 5.185638 -1.832390 1.269699
H 5.358022 -1.282608 2.188099
C 6.059847 -2.998296 -0.651151
H 6.903186 -3.356627 -1.227947
H -0.787795 -1.837022 -3.541816
H 0.248282 -3.269407 -3.278307
H 0.963312 -1.655082 -3.490642

76

8-c10.out delta G = 0.5798 kcal/mol

conformer 2

O -0.050033 -0.679667 -1.290065
C -0.006536 -1.986976 -1.576643
C 1.137547 3.286803 -0.512886
H 3.715434 4.176740 -1.953047
O -0.245266 1.997626 2.364588
C 0.622060 1.348423 1.796321
N 1.717424 0.824105 2.375892

O -0.215492 -2.863458 -0.758609
O 0.708108 4.438224 -0.490436
C 2.658185 0.153304 1.495229
H 3.652831 0.560717 1.670039
H -4.789707 -0.816081 2.248094
O -1.638995 3.411821 -1.511686
H -1.077503 4.185007 -1.348106
O -4.199223 -0.203708 1.777080
H 1.880466 0.930608 3.366491
C 2.143390 0.588344 0.083221
H 2.244676 -0.241738 -0.614694
C 3.009878 1.719912 -0.410194
O -2.308160 -2.175656 0.985960
H -1.551427 -2.624150 0.573283
C 2.575108 2.988909 -0.561338
O -5.657651 -2.279749 1.326854
C 0.202996 2.099191 -0.630932
H 0.340339 1.732005 -1.654342
C 2.724848 -1.364319 1.745806
H 2.977744 -1.509615 2.799101
H 1.744273 -1.813326 1.591266
C 0.658765 0.963289 0.297494
C 4.447296 1.361084 -0.665519
H 4.799312 1.818353 -1.590864
H 4.582325 0.283065 -0.734359
H 5.098258 1.723364 0.135372
C 3.500629 4.131606 -0.880978
H 4.450907 4.038858 -0.356573
H 3.036073 5.074687 -0.600099
C -1.278964 2.472142 -0.491690
H -1.444174 2.915186 0.492424
C -2.181802 1.252939 -0.649534
H -2.073496 0.904239 -1.683035
C -3.650139 1.609174 -0.411466
H -3.957233 2.372971 -1.125907
H -3.762187 2.036903 0.588694
C -4.577008 0.398566 -0.543387
H -4.457012 -0.004514 -1.553028
C -4.119145 -0.700228 0.450444
C -5.005601 -1.951402 0.352058
C -2.666642 -1.095109 0.129335
H -2.641576 -1.427931 -0.913357
C -0.276022 -0.245338 0.084849
H 0.003514 -1.060146 0.745930
C -1.743527 0.116696 0.287158
H -1.837238 0.453308 1.320391
C 0.362830 -2.223468 -3.007743
C 6.062642 -2.386595 0.238327
H 7.115662 -2.206749 0.415633
C 5.113733 -1.819699 1.080950
H 5.436322 -1.199332 1.909125
C 3.749442 -2.036921 0.870230
C -6.040619 0.782376 -0.340980
H -6.713274 -0.057754 -0.527284
H -6.316453 1.585632 -1.025913
H -6.208511 1.131087 0.678852
C -5.032101 -2.737594 -0.923860
H -5.861127 -3.441226 -0.904443
H -4.096100 -3.295058 -1.011231
H -5.109012 -2.091828 -1.798206
C 4.306936 -3.418017 -1.042367
H 3.984486 -4.044348 -1.864797
C 3.359835 -2.848897 -0.195360
H 2.308213 -3.049728 -0.354863
C 5.661558 -3.184111 -0.830972
H 6.400343 -3.624209 -1.488741
H -0.207413 -1.565432 -3.662160
H 0.190574 -3.264107 -3.266706
H 1.420972 -1.987317 -3.139423
76
8-c1.out delta G = 1.4414 kcal/mol
conformer 3
O -0.062662 -0.699603 -1.280921
C -0.009281 -2.013917 -1.537010
C 1.125320 3.281633 -0.558723
H 3.021468 5.064772 -0.714380
O -0.244127 2.000941 2.353590
C 0.626373 1.360587 1.779783
N 1.733475 0.853805 2.351868
O -0.203653 -2.871042 -0.695245
O 0.698868 4.434238 -0.539904
C 2.667626 0.176499 1.469569
H 3.662331 0.590230 1.628471
H -3.736154 -1.047495 2.293672
O -1.669514 3.407206 -1.504317
H -1.103412 4.180613 -1.358759
O -4.276495 -0.425916 1.786841
H 1.903382 0.966802 3.340617

C 2.135561 0.590337 0.057614
H 2.229405 -0.250721 -0.627982
C 2.996066 1.712927 -0.464937
O -2.323631 -2.163175 1.013283
H -1.550885 -2.621548 0.643662
C 2.561086 2.980205 -0.628304
O -4.940779 -2.429551 -1.100659
C 0.185409 2.095216 -0.648673
H 0.302871 1.717860 -1.670928
C 2.742270 -1.337574 1.738782
H 2.996867 -1.468656 2.793571
H 1.763711 -1.793251 1.590570
C 0.652398 0.966570 0.282849
C 4.429056 1.347904 -0.735763
H 5.092213 1.722450 0.049313
H 4.767743 1.789580 -1.673598
H 4.562074 0.268768 -0.788983
C 3.483558 4.116048 -0.979708
H 3.689143 4.138402 -2.054279
H 4.438516 4.034130 -0.461883
C -1.291516 2.475839 -0.483029
H -1.433402 2.929499 0.499625
C -2.207146 1.262628 -0.611228
H -2.151327 0.927246 -1.653239
C -3.657258 1.621813 -0.287325
H -3.989400 2.426305 -0.944039
H -3.711315 1.999204 0.738271
C -4.604431 0.432299 -0.443483
H -4.553330 0.089448 -1.480336
C -4.131756 -0.763403 0.409069
C -4.972624 -2.007740 0.039170
C -2.663502 -1.103310 0.112298
H -2.618287 -1.460707 -0.918748
C -0.281587 -0.245098 0.087354
H 0.003933 -1.050113 0.757960
C -1.750325 0.110990 0.293325
H -1.840009 0.432400 1.332573
C 0.350026 -2.282123 -2.964713
C 4.334942 -3.422820 -1.020337
H 4.016077 -4.063952 -1.832679
C 3.384555 -2.846892 -0.181566
H 2.334273 -3.057468 -0.337442
C 3.769686 -2.016156 0.871106
C -6.050312 0.820528 -0.133140
H -6.741306 -0.004860 -0.316102
H -6.356078 1.653750 -0.767919
H -6.156565 1.128775 0.907477
C -5.805337 -2.649509 1.109426
H -6.447213 -1.910077 1.589911
H -5.150899 -3.048626 1.888594
H -6.398709 -3.454405 0.681322
C 6.084740 -2.360197 0.242809
H 7.136665 -2.170681 0.416487
C 5.132712 -1.786708 1.077259
H 5.451792 -1.151497 1.895481
C 5.688111 -3.176868 -0.813640
H 6.429289 -3.622344 -1.465049
H 1.403590 -2.035540 -3.112933
H -0.235391 -1.648650 -3.629895
H 0.188821 -3.331161 -3.195223

76

8-c5.out delta G = 1.5286 kcal/mol

conformer 4

O -0.400391 -0.183143 -1.999056
C -0.388331 -1.377697 -2.612213
C 0.972601 3.532020 -0.419533
H 2.738633 5.433078 -0.490796
O 0.494034 1.339507 2.186757
C 1.134481 0.942984 1.222887
N 2.318556 0.304753 1.270553
O -0.318762 -2.436158 -2.017138
O 0.569313 4.590770 0.056813
C 2.947888 0.015485 -0.008717
H 3.970761 0.388571 0.017155
H -2.967987 -1.777479 2.145585
O -1.973361 3.722949 -0.517749
H -1.378906 4.455659 -0.293991
O -3.621707 -1.079877 1.999949
H 2.757592 0.087330 2.152839
C 2.079362 0.866005 -0.990186
H 1.956555 0.329970 -1.931347
C 2.803137 2.157337 -1.276922
O -1.955114 -2.374544 0.278998
H -1.303016 -2.649729 -0.386452
C 2.346609 3.374721 -0.914921
O -5.047660 -2.231198 -1.071155
C 0.033670 2.360290 -0.636598
H -0.134181 2.325657 -1.719700

C 3.011915 -1.484586 -0.345160
H 2.007960 -1.888547 -0.470413
H 3.519040 -1.580769 -1.307517
C 0.726078 1.041872 -0.266690
C 4.137474 2.008137 -1.952703
H 4.228382 2.710188 -2.783080
H 4.283903 0.999475 -2.336755
H 4.958037 2.226539 -1.263533
C 3.169732 4.624196 -1.076388
H 3.195834 4.945505 -2.121889
H 4.200103 4.469933 -0.755294
C -1.339343 2.556532 0.020179
H -1.202156 2.675097 1.097080
C -2.265561 1.372239 -0.238166
H -2.487776 1.364491 -1.311499
C -3.576521 1.509647 0.536026
H -4.069254 2.440920 0.255805
H -3.355829 1.570520 1.605906
C -4.533978 0.345665 0.281214
H -4.761142 0.316243 -0.787753
C -3.854416 -1.003196 0.595422
C -4.768129 -2.152460 0.109419
C -2.521699 -1.133173 -0.156631
H -2.755865 -1.182119 -1.222374
C -0.236001 -0.128981 -0.552817
H 0.213975 -1.063020 -0.233152
C -1.589156 0.045889 0.128246
H -1.388139 0.046869 1.201321
C -0.462336 -1.230310 -4.099759
C 3.725459 -3.541472 2.772490
H 3.168344 -4.011497 3.573360
C 3.047338 -2.873503 1.757250
H 1.964930 -2.825818 1.772086
C 3.746349 -2.262436 0.714416
C -5.844142 0.518720 1.050157
H -6.303173 1.474445 0.792266
H -5.668661 0.504815 2.126376
H -6.563144 -0.267631 0.811565
C -5.280514 -3.132321 1.123516
H -4.439497 -3.682225 1.553672
H -5.969226 -3.828050 0.649322
H -5.766155 -2.610036 1.948761
C 5.822101 -3.005135 1.719747
H 6.903527 -3.057654 1.697663
C 5.141175 -2.336677 0.707942
H 5.697016 -1.871362 -0.098085
C 5.115330 -3.608319 2.756856
H 5.644176 -4.129765 3.544671
H -0.516163 -2.209818 -4.565516
H 0.420375 -0.696819 -4.456002
H -1.336456 -0.636686 -4.368939

76

8-c6.out delta G = 2.9982 kcal/mol

conformer 5

O -0.060402 -0.694154 -1.283912
C -0.007131 -2.004098 -1.562973
C 1.126563 3.277139 -0.527139
H 3.468305 4.355913 -1.980224
O -0.233975 1.980510 2.366855
C 0.635357 1.340221 1.790638
N 1.739434 0.824520 2.360039
O -0.192953 -2.876254 -0.735660
O 0.696434 4.428411 -0.509248
C 2.674199 0.154978 1.471715
H 3.668936 0.566062 1.636650
H -3.974578 0.495383 1.959283
O -1.661239 3.394319 -1.499715
H -1.098460 4.168738 -1.345966
O -4.306451 -0.398270 1.817635
H 1.909801 0.930219 3.349534
C 2.144503 0.584838 0.063751
H 2.240833 -0.247189 -0.632395
C 3.005276 1.716938 -0.440610
O -2.284381 -2.180500 1.027633
H -1.541360 -2.641929 0.605715
C 2.564670 2.984630 -0.584898
O -4.960077 -2.504022 -0.929961
C 0.193554 2.087955 -0.634448
H 0.322086 1.719185 -1.658484
C 2.747704 -1.362329 1.723421
H 3.001278 -1.506258 2.776766
H 1.769441 -1.816246 1.568561
C 0.660915 0.955300 0.291802
C 4.438530 1.349890 -0.714167
H 4.783063 1.804713 -1.642985
H 4.562630 0.270837 -0.788040
H 5.102132 1.701597 0.080662
C 3.469231 4.142074 -0.907149

H 4.495471 3.953334 -0.600674
H 3.113177 5.039567 -0.402726
C -1.287509 2.458420 -0.482218
H -1.444543 2.902545 0.502960
C -2.187623 1.235825 -0.629971
H -2.089142 0.885075 -1.663114
C -3.654771 1.585692 -0.382012
H -3.971728 2.349683 -1.091496
H -3.756514 2.043470 0.610735
C -4.582013 0.374383 -0.507810
H -4.476694 -0.029837 -1.517634
C -4.125382 -0.754501 0.447502
C -4.956591 -2.023006 0.186529
C -2.656096 -1.116349 0.154365
H -2.632737 -1.464523 -0.882137
C -0.271871 -0.256501 0.090073
H 0.013416 -1.068372 0.752139
C -1.740332 0.101707 0.303341
H -1.808885 0.443714 1.338925
C 0.342477 -2.245058 -2.998145
C 6.092247 -2.364942 0.218549
H 7.143956 -2.178747 0.397060
C 5.139007 -1.804452 1.060430
H 5.456987 -1.182492 1.889190
C 3.776213 -2.029663 0.848183
C -6.044376 0.761160 -0.290285
H -6.714093 -0.085514 -0.451800
H -6.330110 1.548317 -0.989681
H -6.208054 1.133473 0.722145
C -5.729074 -2.620230 1.322740
H -5.043530 -2.870291 2.134243
H -6.257532 -3.507739 0.981692
H -6.431526 -1.888400 1.725343
C 4.344173 -3.406297 -1.064471
H 4.026427 -4.034268 -1.887471
C 3.392572 -2.843630 -0.218131
H 2.342586 -3.051621 -0.379327
C 5.697083 -3.164109 -0.851721
H 6.439157 -3.599220 -1.509099
H 1.397648 -2.004889 -3.145922
H -0.240263 -1.592824 -3.647257
H 0.171408 -3.287695 -3.249740

76

8-c2.out delta G = 3.1269 kcal/mol

conformer 6

O 0.344280 -0.258770 -1.691708
C 0.415783 -1.454204 -2.294925
C 1.078173 3.571196 -0.432344
H 4.180869 4.691315 -1.430353
O 0.099662 1.519766 2.412584
C 1.041260 1.280203 1.667123
N 2.313228 1.113981 2.063496
O 0.249302 -2.512829 -1.725738
O 0.573017 4.636379 -0.088930
C 3.303627 0.860657 1.038897
H 4.065274 1.639803 1.098042
H -2.762646 -1.847389 2.096741
O -1.822297 3.482923 -0.920096
H -1.347079 4.294938 -0.689901
O -3.457061 -1.238075 1.810405
H 2.557664 1.205088 3.039049
C 2.483545 1.039443 -0.286548
H 2.641305 0.171166 -0.923073
C 3.048731 2.256073 -0.983311
O -1.471588 -2.501464 0.444431
H -0.706793 -2.751383 -0.101462
C 2.468586 3.471954 -0.908517
O -4.331925 -2.712619 -1.327904
C 0.263580 2.301889 -0.550673
H 0.268146 2.075435 -1.621388
C 4.038316 -0.480622 1.208153
H 4.817273 -0.519498 0.444641
H 4.552171 -0.446628 2.172632
C 0.987906 1.121799 0.128527
C 4.366069 1.998646 -1.649883
H 5.105932 1.612196 -0.943887
H 4.781381 2.878830 -2.131008
H 4.230695 1.224389 -2.410831
C 3.100509 4.766012 -1.342424
H 2.867739 5.542582 -0.613638
H 2.698035 5.100545 -2.302717
C -1.202435 2.449693 -0.141315
H -1.264153 2.702970 0.917535
C -1.974372 1.158933 -0.402150
H -2.033791 1.042960 -1.490791
C -3.392815 1.239568 0.162077
H -3.905202 2.103342 -0.262756
H -3.337912 1.395998 1.243571

C -4.213992 -0.015863 -0.127587
H -4.281581 -0.138729 -1.211807
C -3.486240 -1.274785 0.384570
C -4.226033 -2.531418 -0.130189
C -2.050110 -1.341188 -0.158064
H -2.118429 -1.475462 -1.239340
C 0.209128 -0.161364 -0.240810
H 0.685972 -1.031737 0.203930
C -1.259514 -0.073028 0.175003
H -1.247712 0.020172 1.261309
C 0.748240 -1.307865 -3.748713
C 1.574798 -3.242453 2.103020
H 0.919086 -3.506052 2.923208
C 2.343004 -2.086280 2.178437
H 2.289805 -1.468321 3.065078
C 3.191026 -1.721304 1.127904
C -5.630355 0.097719 0.436923
H -6.246755 -0.762180 0.166702
H -6.118069 0.990617 0.042295
H -5.610233 0.173900 1.524650
C -4.794598 -3.484954 0.879051
H -5.337668 -4.279154 0.371488
H -5.446694 -2.956692 1.576063
H -3.983353 -3.912280 1.473532
C 2.496376 -3.713563 -0.066325
H 2.555575 -4.339415 -0.946987
C 3.254541 -2.549993 0.006210
H 3.911367 -2.285566 -0.814175
C 1.651396 -4.061890 0.980493
H 1.054070 -4.962951 0.922163
H 1.797412 -1.018893 -3.841721
H 0.146509 -0.520245 -4.200442
H 0.587167 -2.251936 -4.261307
76
8-c11.out delta G = 3.6214 kcal/mol
conformer 7
O -0.404150 -0.164256 -2.012143
C -0.395181 -1.343855 -2.654234
C 0.976636 3.524198 -0.408991
H 4.204374 4.468346 -0.720253
O 0.500123 1.320777 2.181388
C 1.142288 0.927540 1.216996
N 2.326023 0.289411 1.263287
O -0.318415 -2.416160 -2.086658
O 0.567537 4.579548 0.069838
C 2.956779 0.006377 -0.016939
H 3.979817 0.378675 0.012411
H -3.267512 -0.249787 2.361740
O -1.971166 3.706190 -0.528384
H -1.381157 4.440878 -0.299225
O -3.616932 -1.090931 2.046792
H 2.764614 0.068500 2.144943
C 2.089993 0.863197 -0.994573
H 1.969733 0.334687 -1.940514
C 2.812952 2.158055 -1.268250
O -1.912674 -2.397299 0.248730
H -1.305180 -2.654398 -0.463912
C 2.353392 3.372043 -0.898273
O -5.018550 -2.343012 -0.933436
C 0.042094 2.351760 -0.637609
H -0.119526 2.323068 -1.721678
C 3.021049 -1.492092 -0.360490
H 2.017544 -1.895422 -0.490981
H 3.531430 -1.583789 -1.321520
C 0.735476 1.032378 -0.272385
C 4.149924 2.016322 -1.940371
H 4.241527 2.723332 -2.766491
H 4.300401 1.010266 -2.329621
H 4.967612 2.233253 -1.247340
C 3.175737 4.623639 -1.046385
H 2.740140 5.427934 -0.457808
H 3.207601 4.952190 -2.089465
C -1.336120 2.541344 0.010214
H -1.207881 2.656462 1.088854
C -2.253108 1.352395 -0.260307
H -2.448823 1.331992 -1.338009
C -3.588800 1.498800 0.467730
H -4.084676 2.412159 0.140134
H -3.403272 1.632061 1.541628
C -4.525867 0.311297 0.232918
H -4.712421 0.233224 -0.841086
C -3.829810 -1.011675 0.637669
C -4.716841 -2.203079 0.235872
C -2.503634 -1.156839 -0.133409
H -2.769718 -1.185048 -1.193868
C -0.226022 -0.136844 -0.567566
H 0.224644 -1.076203 -0.265623
C -1.574031 0.031885 0.127113

H -1.349370 0.047094 1.196633
C -0.478822 -1.158815 -4.137244
C 3.723559 -3.563953 2.749743
H 3.163494 -4.038377 3.545943
C 3.049000 -2.891563 1.735042
H 1.966506 -2.844947 1.745638
C 3.751794 -2.274852 0.698095
C -5.864535 0.502952 0.944710
H -6.563018 -0.304242 0.716762
H -6.325139 1.439349 0.626316
H -5.734715 0.542172 2.027385
C -5.179098 -3.145124 1.305328
H -5.732670 -2.599549 2.071463
H -4.312104 -3.582656 1.803497
H -5.800148 -3.923211 0.867068
C 5.824030 -3.020709 1.708205
H 6.905585 -3.072252 1.690293
C 5.146694 -2.347885 0.696887
H 5.705462 -1.878204 -0.104585
C 5.113544 -3.629514 2.739495
H 5.639629 -4.154322 3.526932
H -0.572199 -2.124639 -4.624888
H 0.422048 -0.652635 -4.488587
H -1.330622 -0.525492 -4.385586

**Table S28.** Geometry data of conformers of compound **9**.

75
9-c9-gfn0.out delta G = 0.0000 kcal/mol
conformer 1
O -0.642188 -0.121813 -1.966211
C -0.354626 -1.243629 -2.659144
C 1.037291 3.537054 -0.595537
O 0.511239 1.439242 2.131408
C 1.122720 1.011412 1.160540
N 2.308652 0.376754 1.205038
O 0.727179 4.619460 -0.115724
C 2.891402 0.000240 -0.071649
H -1.713058 3.610606 -0.441518
O -3.685940 -0.642077 2.135908
C 1.998504 0.796397 -1.077999
C 2.747871 2.035942 -1.499505
O 0.169765 -2.220302 -2.176064
O -2.097458 -2.188334 0.466970
C 2.365745 3.290586 -1.188905
O -5.410436 -2.431801 1.466595
C 0.036681 2.406454 -0.722021
C 2.924384 -1.518967 -0.307346
C 0.677878 1.065379 -0.319365
C 4.029562 1.784568 -2.246218
C 3.223656 4.490628 -1.486811
C -1.298922 2.685486 -0.037732
C -2.292705 1.543490 -0.251077
C -3.606028 1.787043 0.486856
C -4.608571 0.650956 0.273977
C -3.977746 -0.685340 0.742518
C -4.944787 -1.854564 0.500286
C -2.649753 -0.955495 0.006975
C -0.359066 -0.061908 -0.541202
C -1.674060 0.208281 0.185548
C -0.766604 -1.096699 -4.094769
C 3.669299 -3.380162 2.924360
C 2.982019 -2.769474 1.879520
C 3.669064 -2.235898 0.787168
C -5.942069 0.931708 0.963420
C -5.275776 -2.258769 -0.904054
C 5.753203 -2.940831 1.804101
C 5.062724 -2.329517 0.762962
C 5.057772 -3.466907 2.889827
H 2.761350 0.183827 2.086022
H 3.918344 0.362786 -0.104659
H -4.307045 -1.265437 2.551997
H 1.828229 0.187781 -1.966100
H -2.080089 -2.131598 1.433627
H -0.148128 2.321030 -1.799622
H 1.910530 -1.906048 -0.391489
H 3.410729 -1.688067 -1.270617
H 4.110925 0.746500 -2.566580
H 4.902799 2.013148 -1.628773
H 4.092554 2.422859 -3.129232
H 3.217221 4.724780 -2.555632
H 4.261865 4.325605 -1.195868
H 2.845708 5.359448 -0.952285
H -1.140449 2.839815 1.029023
H -2.514620 1.475386 -1.322038
H -4.052777 2.722444 0.140311
H -3.411735 1.901184 1.557089
H -4.783707 0.563463 -0.802463

H -2.855410 -1.095216 -1.053938
H 0.040536 -1.023824 -0.237837
H -1.436238 0.275374 1.249336
H -1.830772 -0.865459 -4.153156
H -0.554494 -2.014768 -4.635214
H -0.223626 -0.265830 -4.547879
H 3.120393 -3.790111 3.763081
H 1.900779 -2.705381 1.908976
H -6.690385 0.168696 0.739441
H -6.338943 1.892043 0.630936
H -5.815143 0.974754 2.046262
H -4.460592 -2.882505 -1.281624
H -5.366590 -1.402232 -1.570435
H -6.193376 -2.842782 -0.912974
H 6.833417 -3.009125 1.767302
H 5.610059 -1.924159 -0.080466
H 5.594123 -3.943884 3.700461
75
9-c5.out delta G = 0.0596 kcal/mol
conformer 2
O -0.326791 -0.658020 -1.317801
C 0.013538 -1.943996 -1.531678
C 1.164198 3.269466 -0.700835
O -0.246210 2.122806 2.258845
C 0.576169 1.420679 1.684334
N 1.646184 0.843975 2.261943
O 0.813299 4.441444 -0.662652
C 2.565744 0.145231 1.381544
H -1.509828 3.330795 -1.373078
O -4.406791 0.092014 1.719694
C 2.028551 0.539159 -0.035140
C 2.942380 1.589656 -0.616436
O 0.267617 -2.730871 -0.648106
O -2.545937 -1.883694 1.146373
C 2.580839 2.873059 -0.814141
O -6.011010 -1.851538 1.203526
C 0.159660 2.137094 -0.760200
C 2.640479 -1.366371 1.661190
C 0.569001 1.001981 0.196643
C 4.346867 1.129040 -0.899225
C 3.561107 3.937131 -1.228305
C -1.290057 2.587402 -0.605235
C -2.262936 1.415178 -0.732590
C -3.708461 1.853757 -0.515927
C -4.691569 0.688210 -0.636748
C -4.312684 -0.409264 0.389768
C -5.261763 -1.612047 0.273429
C -2.857885 -0.878690 0.182646
C -0.442940 -0.159286 0.043902
C -1.885319 0.299575 0.252638
C 0.058768 -2.245116 -3.000559
C 4.680221 -3.289507 -0.920199
C 3.597401 -2.775675 -0.210504
C 3.798340 -1.998106 0.930262
C -6.140362 1.146074 -0.481969
C -5.232063 -2.457125 -0.963160
C 6.191069 -2.269077 0.648522
C 5.108584 -1.758098 1.354263
C 5.979914 -3.033915 -0.496550
H 1.833487 0.972909 3.245574
H 3.563581 0.557462 1.528418
H -5.159557 -0.376037 2.122043
H 2.066042 -0.332181 -0.688001
H -2.783575 -1.512680 2.008766
H 0.277052 1.716981 -1.765988
H 2.767330 -1.496961 2.738789
H 1.703961 -1.846136 1.378980
H 5.046424 1.486029 -0.138033
H 4.694563 1.518176 -1.857003
H 4.413587 0.042579 -0.918871
H 3.148586 4.921707 -1.017134
H 3.767755 3.887626 -2.301910
H 4.512241 3.835934 -0.705696
H -1.423294 3.070502 0.362045
H -2.182410 1.007598 -1.746442
H -3.973358 2.617329 -1.251744
H -3.811490 2.310910 0.472226
H -4.566994 0.252819 -1.632740
H -2.776231 -1.360616 -0.791475
H -0.206454 -0.973601 0.721943
H -1.941925 0.707202 1.263967
H 0.895252 -1.705468 -3.449031
H -0.854457 -1.902314 -3.486322
H 0.191990 -3.312229 -3.153921
H 4.505941 -3.890079 -1.804512
H 2.587790 -2.983300 -0.539261
H -6.849291 0.335287 -0.661784
H -6.359066 1.941542 -1.195831

H -6.316707 1.534325 0.522385
H -5.091887 -1.862715 -1.864939
H -6.147521 -3.040151 -1.033817
H -4.384208 -3.143851 -0.888539
H 7.199328 -2.071796 0.991042
H 5.282326 -1.161512 2.242735
H 6.822146 -3.431161 -1.048996
75
9-c1.out delta G = 0.4719 kcal/mol
conformer 3
O -0.117028 -0.662667 -1.266652
C -0.092087 -1.989624 -1.443386
C 1.163046 3.337375 -0.738159
O -0.247554 2.206093 2.242625
C 0.611324 1.533614 1.687793
N 1.715971 1.039549 2.278224
O 0.778890 4.499259 -0.734823
C 2.631027 0.298771 1.428390
H -1.511661 3.316113 -1.401531
O -4.370007 -0.108117 1.708894
C 2.099130 0.647294 -0.000247
C 2.987387 1.711317 -0.596288
O -0.322242 -2.791639 -0.556634
O -2.450232 -1.942719 1.069946
C 2.590638 2.978111 -0.830242
O -5.045556 -2.255283 -1.070448
C 0.189645 2.176341 -0.774265
C 2.676382 -1.200030 1.779092
C 0.626531 1.075543 0.209359
C 4.406331 1.285499 -0.858335
C 3.544306 4.057456 -1.266210
C -1.270543 2.589218 -0.624612
C -2.214110 1.391671 -0.734730
C -3.662039 1.797569 -0.467641
C -4.636835 0.624228 -0.574496
C -4.209209 -0.529709 0.356089
C -5.074254 -1.773839 0.045675
C -2.744842 -0.923715 0.106869
C -0.340278 -0.122173 0.071460
C -1.805161 0.275756 0.235767
C 0.295868 -2.356532 -2.841573
C 5.982137 -2.381025 0.318921
C 5.050419 -1.746502 1.131931
C 3.680646 -1.945879 0.940510
C -6.078328 1.063993 -0.316260
C -5.923042 -2.344505 1.143380
C 4.197197 -3.447010 -0.891285
C 3.267470 -2.809568 -0.074216
C 5.557726 -3.230043 -0.700561
H 1.887102 1.189150 3.261862
H 3.635298 0.699501 1.559787
H -3.851059 -0.708726 2.261237
H 2.163672 -0.233373 -0.637844
H -1.679039 -2.434526 0.741951
H 0.319737 1.739154 -1.771278
H 2.938310 -1.279865 2.837140
H 1.686558 -1.641182 1.663476
H 4.508556 0.201987 -0.834691
H 5.092403 1.696348 -0.112000
H 4.743824 1.646399 -1.830928
H 3.767487 3.978678 -2.334713
H 4.490776 4.001473 -0.728417
H 3.100144 5.035530 -1.092697
H -1.415133 3.081064 0.336330
H -2.149794 0.992348 -1.753864
H -3.963496 2.570608 -1.179432
H -3.732766 2.238896 0.530854
H -4.574651 0.219529 -1.588045
H -2.693083 -1.338843 -0.902194
H -0.080389 -0.895388 0.788835
H -1.899456 0.657891 1.254158
H 1.385386 -2.315107 -2.916397
H -0.117362 -1.649741 -3.558424
H -0.033004 -3.368484 -3.061707
H 7.039774 -2.213711 0.480265
H 5.390295 -1.086151 1.921370
H -6.195103 1.439332 0.700978
H -6.785624 0.244659 -0.460252
H -6.353454 1.862548 -1.007249
H -6.558220 -1.571619 1.577985
H -5.278814 -2.708671 1.947709
H -6.524561 -3.163806 0.755880
H 3.856860 -4.113183 -1.674237
H 2.210803 -2.997070 -0.215157
H 6.282944 -3.723058 -1.335484
75
9-c3.out delta G = 1.1007 kcal/mol
conformer 4

O -0.500180 -0.168344 -1.976654
C -0.532170 -1.395745 -2.516898
C 1.015572 3.594618 -0.626146
O 0.537887 1.533410 2.110423
C 1.139694 1.081267 1.145824
N 2.310116 0.416087 1.193818
O 0.674118 4.679868 -0.175596
C 2.894063 0.042225 -0.084420
H -1.736684 3.621427 -0.490288
O -3.695334 -0.724916 2.102585
C 2.021042 0.862398 -1.085754
C 2.772844 2.111576 -1.474186
O -0.484583 -2.419917 -1.861749
O -2.093096 -2.177506 0.444618
C 2.365073 3.361466 -1.176046
O -5.197812 -2.007127 -0.878748
C 0.035009 2.445566 -0.756462
C 2.906823 -1.475198 -0.339663
C 0.695008 1.122801 -0.335976
C 4.079639 1.877150 -2.181893
C 3.217011 4.571857 -1.447571
C -1.308024 2.705647 -0.081081
C -2.284073 1.547117 -0.285941
C -3.584611 1.770979 0.483268
C -4.582384 0.625207 0.310035
C -3.941300 -0.723918 0.697988
C -4.897191 -1.870001 0.291340
C -2.621828 -0.943635 -0.057624
C -0.310821 -0.032577 -0.537031
C -1.651216 0.219603 0.147719
C -0.620160 -1.335806 -4.010221
C 3.684143 -3.387180 2.854520
C 2.985381 -2.755283 1.830113
C 3.660069 -2.215336 0.733434
C -5.876968 0.885234 1.080920
C -5.422200 -2.775327 1.366555
C 5.753281 -2.956221 1.703626
C 5.051819 -2.323758 0.682813
C 5.070696 -3.488670 2.794352
H 2.764585 0.226690 2.074686
H 3.927182 0.386634 -0.107416
H -3.057216 -1.429200 2.281549
H 1.851864 0.276374 -1.989211
H -1.451943 -2.507437 -0.206396
H -0.138491 2.351420 -1.835617
H 1.888137 -1.855783 -0.405647
H 3.374640 -1.637861 -1.312976
H 4.930522 2.109846 -1.535445
H 4.164285 2.522008 -3.058153
H 4.181745 0.842178 -2.506422
H 4.252562 4.411095 -1.145884
H 2.825185 5.430758 -0.907048
H 3.221246 4.820186 -2.513247
H -1.153700 2.868167 0.985089
H -2.521207 1.476740 -1.354210
H -4.053566 2.699634 0.147606
H -3.356745 1.895381 1.546002
H -4.823337 0.538374 -0.752722
H -2.870925 -1.047835 -1.116083
H 0.117018 -0.960229 -0.170673
H -1.435027 0.279429 1.216640
H -1.447902 -0.692532 -4.309014
H -0.755097 -2.335428 -4.412637
H 0.297444 -0.898563 -4.407895
H 3.145510 -3.802209 3.697339
H 1.905526 -2.680413 1.879848
H -6.623639 0.110094 0.896574
H -6.309041 1.838612 0.772400
H -5.688677 0.928618 2.154234
H -6.138475 -3.475435 0.942244
H -5.879161 -2.193146 2.167707
H -4.590978 -3.325379 1.815029
H 6.831786 -3.036150 1.646882
H 5.588648 -1.913463 -0.164931
H 5.615538 -3.982256 3.589194

75

9-c11-gfn0.out delta G = 2.4956 kcal/mol

conformer 5

O -0.592361 -0.100331 -2.005796
C -0.251168 -1.189642 -2.725080
C 1.028585 3.536456 -0.536066
O 0.446703 1.389606 2.148279
C 1.089456 0.985696 1.187239
N 2.283689 0.369364 1.254397
O 0.698788 4.609283 -0.047807
C 2.902176 0.013162 -0.010908
H -1.725753 3.583836 -0.438933
O -3.678782 -0.840538 2.004890

C 2.026228 0.811212 -1.030773
C 2.773240 2.062590 -1.419555
O 0.301754 -2.159804 -2.260724
O -2.075541 -2.239423 0.323430
C 2.372001 3.309722 -1.103029
O -5.243337 -2.002236 -0.997189
C 0.040701 2.399599 -0.700476
C 2.957314 -1.503289 -0.259782
C 0.683464 1.057441 -0.303323
C 4.074632 1.831900 -2.138437
C 3.225060 4.521159 -1.366837
C -1.310787 2.657255 -0.039339
C -2.291771 1.510312 -0.284695
C -3.610488 1.728046 0.450850
C -4.604625 0.591917 0.210950
C -3.985126 -0.766789 0.609227
C -4.924375 -1.913765 0.172183
C -2.635002 -0.993003 -0.100127
C -0.338713 -0.072841 -0.574204
C -1.670561 0.169773 0.130480
C -0.642548 -1.016028 -4.163478
C 5.749141 -2.907945 1.911678
C 5.077751 -2.296382 0.858260
C 3.682785 -2.221391 0.846869
C -5.937442 0.850948 0.916082
C -5.405232 -2.883970 1.211105
C 3.643060 -3.385053 2.973351
C 2.974977 -2.773980 1.916331
C 5.032992 -3.453040 2.974319
H 2.715077 0.169369 2.144450
H 3.925685 0.386782 -0.014305
H -4.429697 -0.539390 2.528248
H 1.885971 0.210235 -1.929108
H -2.037407 -2.215807 1.289616
H -0.120579 2.328568 -1.782806
H 1.949964 -1.899916 -0.372002
H 3.468606 -1.658629 -1.212338
H 4.930638 2.068177 -1.500166
H 4.149207 2.476092 -3.016323
H 4.176694 0.796821 -2.462527
H 4.257900 4.362177 -1.054172
H 2.826867 5.379971 -0.830828
H 3.240842 4.768091 -2.432675
H -1.173520 2.799386 1.031983
H -2.506104 1.462429 -1.358213
H -4.062970 2.668060 0.124546
H -3.416516 1.825758 1.523596
H -4.797867 0.520971 -0.862407
H -2.839389 -1.104020 -1.164152
H 0.062970 -1.038700 -0.286555
H -1.454089 0.212969 1.199851
H -0.117937 -0.156392 -4.583419
H -1.712247 -0.816559 -4.235361
H -0.391546 -1.911582 -4.724669
H 6.830776 -2.961491 1.902505
H 5.641378 -1.875982 0.033121
H -6.666611 0.062177 0.722009
H -6.365958 1.790134 0.563973
H -5.815421 0.947559 1.998932
H -6.021026 -3.648239 0.742501
H -5.986778 -2.365110 1.977397
H -4.552859 -3.337379 1.719277
H 3.078110 -3.809808 3.793866
H 1.892548 -2.724312 1.918531
H 5.554531 -3.930084 3.794528
75
9-c8-gfn0.out delta G = 2.6035 kcal/mol
conformer 6
O -0.633130 -0.133436 -1.986231
C -0.323622 -1.243745 -2.688988
C 1.037739 3.520366 -0.614202
O 0.496353 1.418747 2.116431
C 1.119811 0.997716 1.149743
N 2.311815 0.376478 1.202987
O 0.722599 4.602652 -0.137433
C 2.901549 -0.005554 -0.068751
H -1.713837 3.585552 -0.466866
O -3.747295 -0.818427 2.129570
C 2.010229 0.781637 -1.083635
C 2.756575 2.022226 -1.506919
O 0.221938 -2.212572 -2.213840
O -2.077397 -2.212507 0.448333
C 2.368654 3.276643 -1.202660
O -5.228387 -2.020883 -0.837396
C 0.040793 2.386391 -0.740804
C 2.940161 -1.526199 -0.294009
C 0.684225 1.047712 -0.333167
C 4.042348 1.772262 -2.247047

C 3.223257 4.478685 -1.501798
C -1.296000 2.664337 -0.058280
C -2.287661 1.518477 -0.262722
C -3.592207 1.758548 0.491434
C -4.597797 0.620779 0.299581
C -3.976161 -0.730772 0.716224
C -4.934251 -1.873113 0.332347
C -2.638170 -0.982635 -0.014537
C -0.349296 -0.081212 -0.562266
C -1.665286 0.182586 0.165355
C -0.740276 -1.093637 -4.122651
C 3.674150 -3.364556 2.953361
C 2.990385 -2.763244 1.900785
C 3.681496 -2.233170 0.809238
C -5.918261 0.900486 1.017529
C -5.470083 -2.758626 1.416485
C 5.762645 -2.922374 1.842912
C 5.075688 -2.320521 0.793958
C 5.063167 -3.445136 2.927642
H 2.759202 0.186542 2.087369
H 3.927370 0.360235 -0.099335
H -3.422262 0.029517 2.454423
H 1.848012 0.167564 -1.969426
H -2.087745 -2.168941 1.415696
H -0.141871 2.297922 -1.818378
H 1.927888 -1.916791 -0.380294
H 3.431415 -1.700406 -1.253819
H 4.102801 2.400998 -3.137137
H 4.132858 0.731286 -2.555257
H 4.911887 2.014872 -1.629807
H 3.217084 4.710884 -2.571037
H 4.261650 4.317234 -1.209589
H 2.842228 5.347386 -0.969246
H -1.136746 2.825710 1.007357
H -2.524532 1.450177 -1.329949
H -4.049498 2.690999 0.152172
H -3.376104 1.907901 1.557289
H -4.798244 0.528210 -0.771004
H -2.858000 -1.127384 -1.071636
H 0.051042 -1.044105 -0.263005
H -1.412306 0.248459 1.227491
H -0.214909 -0.248121 -4.569345
H -1.809040 -0.883861 -4.177785
H -0.510060 -2.002803 -4.670661
H 3.122055 -3.772092 3.791171
H 1.908661 -2.704024 1.923683
H -6.328997 1.853550 0.681113
H -5.779193 0.957961 2.098446
H -6.662651 0.128914 0.813080
H -6.147511 -3.494173 0.988634
H -5.980983 -2.160792 2.172924
H -4.642569 -3.257148 1.925355
H 6.843357 -2.985946 1.812968
H 5.626320 -1.917741 -0.048559
H 5.596827 -3.914785 3.744308
75
9-c2.out delta G = 2.6431 kcal/mol
conformer 7
O 0.289924 -0.220228 -1.703470
C 0.340487 -1.437457 -2.260691
C 1.124890 3.634039 -0.564201
O 0.099167 1.659043 2.356949
C 1.032503 1.407922 1.604502
N 2.311282 1.267753 1.993472
O 0.679540 4.717606 -0.213140
C 3.286308 0.942498 0.975078
H -1.612961 3.397399 -0.799242
O -3.558984 -0.982117 1.799524
C 2.457529 1.071880 -0.349562
C 3.042806 2.236648 -1.117608
O 0.151245 -2.471683 -1.653957
O -1.593753 -2.346469 0.497436
C 2.498311 3.469085 -1.089828
O -4.444824 -2.542957 -1.293520
C 0.268224 2.390469 -0.636790
C 3.989602 -0.407020 1.206305
C 0.966149 1.206754 0.072262
C 4.339715 1.904473 -1.793175
C 3.147551 4.723848 -1.604364
C -1.183522 2.576639 -0.221903
C -1.991943 1.301712 -0.473773
C -3.415094 1.433613 0.065134
C -4.265894 0.189448 -0.188818
C -3.574696 -1.068424 0.375437
C -4.342306 -2.323110 -0.101912
C -2.135412 -1.190933 -0.149559
C 0.151911 -0.067217 -0.255859
C -1.317790 0.067688 0.146739

C 0.682215 -1.353075 -3.717669
C 2.354428 -3.651267 0.085889
C 3.147550 -2.509087 0.104920
C 3.109522 -1.627044 1.186379
C -5.684820 0.357191 0.355564
C -4.941720 -3.227055 0.934825
C 1.447242 -3.050884 2.229059
C 2.251116 -1.916947 2.251810
C 1.498570 -3.923890 1.146183
H 2.561791 1.380807 2.965064
H 4.066745 1.704970 0.987508
H -2.878510 -1.593450 2.113705
H 2.590659 0.167816 -0.940482
H -0.825718 -2.628335 -0.027920
H 0.280245 2.123987 -1.698374
H 4.763765 -0.502197 0.442794
H 4.508227 -0.340989 2.166596
H 5.075311 1.517511 -1.082868
H 4.781282 2.748937 -2.313668
H 4.165317 1.107708 -2.522541
H 2.689191 5.050044 -2.542369
H 4.215100 4.605198 -1.767840
H 2.997002 5.528404 -0.883688
H -1.242951 2.852307 0.829459
H -2.047149 1.143355 -1.557675
H -3.904372 2.294345 -0.398781
H -3.374241 1.631925 1.140226
H -4.325139 0.029577 -1.268658
H -2.198246 -1.361373 -1.226192
H 0.605435 -0.934556 0.218385
H -1.308418 0.199501 1.229395
H 1.739046 -1.095323 -3.815798
H 0.103357 -0.567151 -4.201077
H 0.498864 -2.310746 -4.196531
H 2.394785 -4.319255 -0.764385
H 3.811261 -2.302873 -0.726543
H -5.674057 0.470387 1.440208
H -6.319920 -0.495952 0.108149
H -6.146272 1.247697 -0.074639
H -4.146067 -3.652217 1.551384
H -5.499683 -4.025395 0.450405
H -5.586696 -2.659062 1.606799
H 0.783419 -3.255407 3.059549
H 2.216416 -1.256798 3.108413
H 0.873517 -4.807712 1.128945
75
9-c3-gfn0.out delta G = 2.7472 kcal/mol
conformer 8
O -0.322515 -0.670907 -1.308958
C 0.005231 -1.960307 -1.526609
C 1.167910 3.257337 -0.703033
O -0.219765 2.100133 2.268258
C 0.604060 1.405895 1.685511
N 1.683845 0.837941 2.252445
O 0.814209 4.428202 -0.658053
C 2.595491 0.138390 1.364363
H -1.511176 3.312085 -1.356408
O -4.476210 -0.056721 1.786071
C 2.043737 0.528805 -0.047475
C 2.949641 1.580978 -0.638253
O 0.273468 -2.745327 -0.645871
O -2.516221 -1.890058 1.196530
C 2.583973 2.864019 -0.831037
O -5.169091 -2.214354 -0.917306
C 0.165599 2.122522 -0.755558
C 2.674009 -1.372690 1.645913
C 0.585450 0.988221 0.197804
C 4.351745 1.122603 -0.935815
C 3.557945 3.930521 -1.253623
C -1.283372 2.570638 -0.589128
C -2.255614 1.396566 -0.705084
C -3.698940 1.834369 -0.474667
C -4.687130 0.670062 -0.572622
C -4.302115 -0.450563 0.417837
C -5.196476 -1.679502 0.173524
C -2.836719 -0.899239 0.218547
C -0.425465 -0.174672 0.053202
C -1.867407 0.281420 0.276023
C 0.003690 -2.267232 -2.994733
C 6.211370 -2.277043 0.589295
C 5.138104 -1.764345 1.307756
C 3.822535 -2.005635 0.901402
C -6.133254 1.133621 -0.394937
C -6.068721 -2.169742 1.289479
C 4.680367 -3.301716 -0.957009
C 3.606797 -2.785972 -0.234765
C 5.985420 -3.044995 -0.550858
H 1.878030 0.965061 3.234998

H 3.594309 0.551755 1.500534
H -4.186178 0.856747 1.894102
H 2.076923 -0.343407 -0.699333
H -2.788395 -1.526183 2.051663
H 0.275922 1.703237 -1.762418
H 2.814414 -1.501541 2.722083
H 1.734104 -1.853287 1.376505
H 5.058589 1.479187 -0.181194
H 4.689233 1.513721 -1.896406
H 4.419479 0.036237 -0.957865
H 4.515052 3.829399 -0.742076
H 3.146529 4.914017 -1.035285
H 3.752327 3.884015 -2.329632
H -1.408999 3.055726 0.378306
H -2.188309 0.987662 -1.718751
H -3.976672 2.592257 -1.210962
H -3.781833 2.330171 0.501390
H -4.584835 0.226253 -1.566343
H -2.769500 -1.394763 -0.749282
H -0.182714 -0.988803 0.729288
H -1.900610 0.691290 1.289374
H 0.728326 -1.628739 -3.502253
H -0.978569 -2.050968 -3.416391
H 0.255620 -3.311803 -3.153377
H 7.223980 -2.078771 0.918131
H 5.323309 -1.165438 2.192338
H -6.366133 1.911554 -1.123445
H -6.298858 1.546325 0.601630
H -6.841777 0.317049 -0.544573
H -6.724697 -1.369068 1.634792
H -5.448368 -2.449781 2.143328
H -6.652194 -3.023106 0.951315
H 4.494669 -3.904699 -1.837345
H 2.593019 -2.994206 -0.550039
H 6.820453 -3.443690 -1.113098
75
9-c6-gfn0.out delta G = 3.2122 kcal/mol
conformer 9
O -0.323401 -0.672466 -1.314399
C 0.015301 -1.958175 -1.532447
C 1.167481 3.256175 -0.707941
O -0.217386 2.108037 2.261674
C 0.601387 1.406998 1.680521
N 1.676847 0.832177 2.249846
O 0.814690 4.427473 -0.665299
C 2.590921 0.135237 1.362314
H -1.511470 3.311183 -1.363284
O -4.369738 -0.022072 1.768934
C 2.041443 0.527256 -0.050099
C 2.948928 1.579257 -0.638786
O 0.291251 -2.741936 -0.652656
O -2.512053 -1.903799 1.169816
C 2.583886 2.862306 -0.832644
O -5.213904 -2.189748 -0.950096
C 0.164795 2.121890 -0.760965
C 2.671935 -1.376068 1.641996
C 0.583076 0.987842 0.193151
C 4.351658 1.120731 -0.933374
C 3.559177 3.928438 -1.253288
C -1.284498 2.569362 -0.595968
C -2.256775 1.395475 -0.713201
C -3.699227 1.833204 -0.480632
C -4.683487 0.668681 -0.586279
C -4.302272 -0.452359 0.405989
C -5.189449 -1.693072 0.158975
C -2.839112 -0.898452 0.206855
C -0.428351 -0.174910 0.048350
C -1.869931 0.281586 0.269665
C 0.017063 -2.264662 -3.000931
C 6.217624 -2.269444 0.603952
C 5.138881 -1.760876 1.317123
C 3.826259 -2.004872 0.902858
C -6.130622 1.133948 -0.411217
C -5.982896 -2.249408 1.304693
C 4.698257 -3.295357 -0.952921
C 3.619169 -2.783713 -0.235953
C 6.000294 -3.035964 -0.538853
H 1.871953 0.962173 3.231818
H 3.588871 0.550043 1.500841
H -5.185673 0.466567 1.923898
H 2.074997 -0.344483 -0.702534
H -2.706571 -1.532034 2.041220
H 0.276300 1.702261 -1.767629
H 2.807278 -1.505961 2.718709
H 1.734840 -1.858818 1.366855
H 4.418794 0.034358 -0.957169
H 5.056642 1.475422 -0.176117
H 4.692072 1.513394 -1.892287

H 3.756889 3.881032 -2.328658
H 4.514736 3.827795 -0.738729
H 3.147085 4.912114 -1.037052
H -1.411213 3.053926 0.371505
H -2.186788 0.987181 -1.727330
H -3.974300 2.596043 -1.213556
H -3.784419 2.298070 0.506585
H -4.589816 0.223997 -1.580213
H -2.776110 -1.385622 -0.765320
H -0.183893 -0.988961 0.723851
H -1.917672 0.691453 1.280443
H -0.961110 -2.040561 -3.427673
H 0.262757 -3.310817 -3.159058
H 0.749462 -1.631544 -3.504261
H 7.227804 -2.069065 0.938955
H 5.317421 -1.163028 2.203797
H -6.372121 1.883631 -1.165724
H -6.295907 1.603598 0.562604
H -6.842359 0.313718 -0.520270
H -6.521615 -3.136892 0.980433
H -6.691605 -1.505907 1.677767
H -5.316163 -2.488232 2.134679
H 4.519197 -3.897221 -1.835408
H 2.607741 -2.993706 -0.557536
H 6.839614 -3.431422 -1.096992

**Table S29.** Geometry data of conformers of compound **10**.

75
10-c2.out delta G = 0.0000 kcal/mol conformer 1
C 1.279457 0.615510 2.018003
C 3.449367 -0.148192 1.386475
C 2.768621 0.378579 0.078022
C 3.618172 1.463376 -0.537195
C 3.225005 2.766931 -0.577021
C 1.875330 3.120186 -0.204358
C 0.943923 2.217881 0.165155
C 1.316456 0.782865 0.469043
C 3.782645 -1.653188 1.397009
C 4.869130 1.012044 -1.116693
C 4.059050 3.911125 -1.085277
C -0.453650 2.566549 0.270418
C -1.437330 1.657174 0.316241
C -2.864795 2.075484 0.538679
C -3.844377 1.342464 -0.382526
C -3.646103 -0.187780 -0.261157
C -4.545279 -0.901241 -1.291273
C -2.197664 -0.548658 -0.644981
C -1.164987 0.177426 0.235169
C 0.267997 -0.116744 -0.215576
C -5.487073 -1.953518 -0.792437
C -5.289555 1.774360 -0.136237
C 2.624661 -2.555697 1.067427
C 1.593173 -2.784395 1.984263
C 0.502920 -3.577571 1.646605
C 0.432748 -4.172870 0.390706
C 1.460715 -3.970599 -0.522036
C 2.543104 -3.164240 -0.186593
C 0.398874 -0.744739 -2.569010
C 0.856093 -0.220573 -3.897185
C -2.594907 2.549972 2.895256
O 0.448898 0.211320 -1.629466
H 1.572390 4.151089 -0.351343
O 0.330910 0.843286 2.751038
O 0.044192 -1.885286 -2.359079
N 2.496335 0.212996 2.416863
H -0.698288 3.623488 0.296052
H -2.956991 3.155828 0.378681
H 4.380384 0.392641 1.557482
H -3.736523 0.029731 1.670522
O -3.977538 -0.666657 1.035270
H 2.666476 0.030334 3.394857
H 2.734005 -0.449015 -0.627761
H 3.628538 4.305126 -2.009925
O -2.064008 -1.957975 -0.545653
H -1.306390 -2.213110 -1.098663
O -4.461305 -0.595519 -2.464254
H 1.940839 -0.095757 -3.870178
H 4.587575 -1.803272 0.678954
H 4.179511 -1.894502 2.387219
H 0.415059 0.755768 -4.094056
O 5.214051 -0.164989 -1.157978
H 5.521348 1.777889 -1.560728
H 5.097482 3.658740 -1.271607
H 4.034270 4.723290 -0.354905
O -3.263850 1.784161 1.893778
H -3.568096 1.608850 -1.404943

H -2.077322 -0.230338 -1.683833
H 0.503610 -1.167233 -0.072199
H -1.266200 -0.243462 1.239386
H 0.592562 -0.924312 -4.681600
H -0.294235 -3.729082 2.363134
H 1.639689 -2.339035 2.969521
H -5.639777 1.444570 0.840034
H -5.951080 1.362726 -0.899429
H -5.370935 2.862113 -0.176914
H -4.920855 -2.718232 -0.258474
H -6.031052 -2.387508 -1.628622
H -6.177937 -1.519217 -0.067558
H 1.411109 -4.422341 -1.503893
H 3.335796 -3.000764 -0.906918
H -0.420888 -4.783255 0.125823
H -1.535557 2.297128 2.945471
H -3.077470 2.306250 3.839755
H -2.714942 3.619930 2.694297
75
10-c9.out delta G = 0.3188 kcal/mol conformer 2
C 1.013875 0.285187 1.671471
C 2.863408 -0.822637 0.655346
C 2.425579 0.311554 -0.323848
C 3.475723 1.396685 -0.396503
C 3.193862 2.706599 -0.144858
C 1.827386 3.118875 0.078714
C 0.791380 2.258704 0.149917
C 1.035895 0.772650 0.190356
C 2.705099 -2.235611 0.073278
C 4.771061 0.971398 -0.896998
C 4.193785 3.830196 -0.162379
C -0.573185 2.713156 0.257437
C -1.634457 1.895578 0.199441
C -3.019196 2.405083 0.489619
C -4.069504 1.849996 -0.477935
C -4.002234 0.303205 -0.511234
C -4.971855 -0.225768 -1.588701
C -2.593990 -0.133708 -0.959093
C -1.492333 0.417350 -0.038184
C -0.101791 0.059269 -0.564798
C -6.006098 -1.225609 -1.172656
C -5.468833 2.377453 -0.161563
C 3.088118 -3.297853 1.068554
C 2.118956 -3.942146 1.839377
C 2.481866 -4.882555 2.799368
C 3.822969 -5.191492 3.001333
C 4.798162 -4.556531 2.236375
C 4.432074 -3.616983 1.279446
C -0.042320 -0.403444 -2.938295
C 0.310293 0.204815 -4.260580
C -2.646674 2.565523 2.878110
O 0.046748 0.498560 -1.946088
H 1.630458 4.183682 0.134058
O 0.217529 0.623588 2.531918
O -0.356800 -1.563388 -2.764718
N 2.021366 -0.590198 1.817222
H -0.726190 3.775688 0.415074
H -3.021747 3.500006 0.441811
H 3.903701 -0.691996 0.944372
H -4.058565 0.335644 1.432718
O -4.356644 -0.275237 0.736600
H 2.155502 -1.079000 2.689944
H 2.338703 -0.122418 -1.319729
H 4.085732 4.417717 0.752318
O -2.568020 -1.554738 -0.996933
H -1.859801 -1.819549 -1.605775
O -4.865654 0.176549 -2.730204
H -0.199901 1.159263 -4.386112
H 1.674838 -2.385788 -0.253561
H 3.344844 -2.288565 -0.808296
H 0.044889 -0.478233 -5.062211
O 5.009457 -0.169280 -1.280638
H 5.561307 1.733484 -0.956099
H 3.980937 4.503795 -0.996935
H 5.227663 3.510286 -0.235748
O -3.423239 2.008379 1.817128
H -3.786013 2.191615 -1.475831
H -2.463291 0.271914 -1.965409
H 0.035585 -1.017740 -0.536798
H -1.609155 -0.095956 0.921264
H 1.384938 0.397295 -4.285495
H 1.716606 -5.375717 3.385865
H 1.072353 -3.708808 1.682475
H -5.457799 3.467839 -0.113374
H -5.830172 2.000425 0.793513
H -6.176221 2.087138 -0.939189
H -5.512543 -2.086855 -0.719347

H -6.594766 -1.527049 -2.036258
H -6.647450 -0.797037 -0.400361
H 5.843997 -4.796708 2.382909
H 5.194397 -3.128037 0.683770
H 4.106769 -5.925221 3.745260
H -2.623384 3.657177 2.795805
H -1.629180 2.172519 2.875721
H -3.148544 2.285705 3.802205
75
10-c1.out delta G = 1.5267 kcal/mol conformer 3
C 1.300825 0.543581 1.936154
C 3.465261 -0.180883 1.275364
C 2.759650 0.329025 -0.026160
C 3.587945 1.418943 -0.662706
C 3.190105 2.721250 -0.688803
C 1.854714 3.076209 -0.264046
C 0.936566 2.172773 0.131079
C 1.311700 0.728451 0.387170
C 3.745879 -1.697455 1.326786
C 4.835949 0.974304 -1.256688
C 4.005973 3.870715 -1.214552
C -0.457250 2.519123 0.308531
C -1.437127 1.605770 0.350575
C -2.861009 1.993909 0.637159
C -3.850626 1.339480 -0.330781
C -3.649208 -0.192411 -0.350861
C -4.559710 -0.805000 -1.433427
C -2.199187 -0.535919 -0.745129
C -1.166699 0.133685 0.176969
C 0.252128 -0.133278 -0.325335
C -5.538074 -1.863113 -1.022079
C -5.293870 1.748008 -0.033602
C 2.525409 -2.573087 1.215252
C 1.654364 -2.746452 2.296397
C 0.503355 -3.515357 2.171752
C 0.206387 -4.135202 0.960994
C 1.071893 -3.985231 -0.115933
C 2.220055 -3.211484 0.013084
C 0.787527 -0.525789 -2.676623
C 0.645339 0.115584 -4.027921
C -2.581673 2.269259 3.022134
O 0.293355 0.288922 -1.720333
H 1.551794 4.110477 -0.385557
O 0.350740 0.702051 2.685431
O 1.272209 -1.609360 -2.462211
N 2.553520 0.242316 2.320004
H -0.699609 3.573430 0.395654
H -2.963892 3.083253 0.577402
H 4.418842 0.330661 1.400958
H -3.724710 -0.164173 1.597791
O -3.955457 -0.805586 0.901804
H 2.736725 0.054860 3.294597
H 2.718657 -0.506331 -0.721352
H 4.037245 4.659838 -0.459138
O -2.019806 -1.946422 -0.762644
H -2.257572 -2.272647 0.116827
O -4.457844 -0.414937 -2.578840
H 1.106438 1.103772 -4.020749
H 4.444853 -1.916161 0.520156
H 4.263574 -1.901073 2.268685
H -0.412810 0.246525 -4.260565
O 5.205295 -0.195154 -1.264983
H 5.463798 1.738627 -1.737597
H 3.522678 4.298856 -2.096880
H 5.027481 3.614355 -1.474315
O -3.239598 1.572401 1.963665
H -3.585302 1.689110 -1.330918
H -2.049155 -0.201674 -1.768877
H 0.494742 -1.189704 -0.272348
H -1.255467 -0.337971 1.160138
H 1.112423 -0.511181 -4.782314
H -0.159632 -3.631373 3.020193
H 1.878636 -2.283104 3.248602
H -5.636149 1.337980 0.914919
H -5.964781 1.405101 -0.822191
H -5.373633 2.835229 0.019199
H -5.003369 -2.700628 -0.570046
H -6.103559 -2.196173 -1.889344
H -6.206310 -1.474667 -0.251571
H 0.845389 -4.451977 -1.065378
H 2.873930 -3.082909 -0.838830
H -0.691666 -4.731572 0.859028
H -3.030004 1.914711 3.948224
H -2.754836 3.346368 2.926722
H -1.511697 2.061589 3.025737

**Table S30.** Geometry data of conformers of compound **11**.

75

11-c18.out delta G = 0.0000 kcal/mol

conformer 1

O 0.175328 -0.038096 -1.650536
C -0.006162 -1.178830 -2.328232
C 1.831402 3.160466 -0.581779
O 0.582959 0.968907 2.661386
O -0.390632 -2.214904 -1.822138
C 1.437478 0.784441 1.791776
N 2.718632 0.509093 2.030482
C 0.352600 -1.021032 -3.774355
C 3.552714 0.203673 0.882050
O -0.639144 3.176298 1.656971
O -3.754643 0.072342 1.714536
C 2.590472 0.386073 -0.317687
C 3.159451 1.254690 -1.450403
O -2.213874 -1.832102 0.240738
C 3.140994 2.710845 -1.051701
O -5.533059 -1.530410 0.759017
C 0.910187 2.343956 -0.058225
C 4.161382 -1.213812 0.993229
C 1.231904 0.889840 0.260243
C 4.494446 0.730544 -1.968979
C 4.173283 3.551924 -1.169085
C -0.478746 2.856554 0.268832
C -1.560327 1.884630 -0.240619
C -2.961965 2.359537 0.141322
C -4.062632 1.392829 -0.302137
C -3.771198 -0.008637 0.297862
C -4.848229 -1.024418 -0.111992
C -2.407885 -0.496508 -0.215693
C 0.081419 -0.032219 -0.190299
C -1.296605 0.450312 0.245715
C 1.292418 -3.534756 1.946588
C 2.300761 -2.580420 2.033449
C 3.134420 -2.316084 0.942714
C -5.447443 1.905732 0.083705
C -5.016417 -1.376401 -1.559468
C 1.945023 -4.017828 -0.315096
C 2.946461 -3.057075 -0.227029
C 1.107199 -4.252897 0.769321
H 1.590048 4.211961 -0.704499
H 3.060530 0.457589 2.979596
H 1.439147 -1.087432 -3.869160
H 0.044126 -0.045719 -4.146642
H -0.102188 -1.817196 -4.357413
H 4.370951 0.924654 0.838537
H -0.362101 2.396468 2.174918
H -4.432880 -0.554808 2.018038
H 2.409403 -0.597719 -0.742589
H 2.434477 1.169645 -2.266199
H -1.538921 -2.225678 -0.339144
H 4.876705 -1.335246 0.179038
H 4.731610 -1.262769 1.924260
H 4.399477 -0.316302 -2.261925
H 5.287897 0.803509 -1.223503
H 4.813697 1.291768 -2.847674
H 5.140214 3.232593 -1.532726
H 4.072747 4.595406 -0.894956
H -0.612640 3.806652 -0.250688
H -1.481503 1.883955 -1.333451
H -3.146615 3.338321 -0.309812
H -3.017981 2.490023 1.221939
H -4.013361 1.298111 -1.390707
H -2.453418 -0.485787 -1.309236
H 0.269281 -1.044855 0.162086
H -1.299054 0.428845 1.335738
H 0.651429 -3.716880 2.800127
H 2.437869 -2.039366 2.960162
H -5.550528 1.947619 1.168857
H -6.244118 1.270087 -0.309100
H -5.600588 2.910913 -0.312371
H -4.956472 -0.499532 -2.203060
H -5.966339 -1.885452 -1.705997
H -4.206072 -2.050723 -1.848320
H 1.810667 -4.571432 -1.235429
H 3.589515 -2.875915 -1.080166
H 0.317680 -4.990279 0.699265

75

11-c1.out delta G = 0.6037 kcal/mol

conformer 2

O 0.154187 -0.063822 -1.630591
C -0.014882 -1.227962 -2.273626
C 1.801127 3.168142 -0.604374
O 0.599553 0.957878 2.667212
O -0.379903 -2.252904 -1.732232

C 1.448354 0.791255 1.788145
N 2.735354 0.531464 2.011687
C 0.330668 -1.107934 -3.726147
C 3.560361 0.241699 0.852253
O -0.617420 3.180908 1.692587
O -3.876412 -0.101076 1.669761
C 2.577661 0.401375 -0.333837
C 3.120663 1.261099 -1.485723
O -2.235172 -1.815398 0.300079
C 3.101380 2.721120 -1.101191
O -4.880498 -1.055316 -1.627931
C 0.894013 2.351755 -0.056435
C 4.200297 -1.162342 0.957140
C 1.224249 0.899852 0.259723
C 4.449824 0.740105 -2.022287
C 4.125280 3.567441 -1.250444
C -0.487270 2.862684 0.300718
C -1.580450 1.893039 -0.188969
C -2.968042 2.345169 0.261257
C -4.081700 1.408137 -0.205141
C -3.799917 -0.040228 0.247009
C -4.835072 -0.979304 -0.415196
C -2.410253 -0.491654 -0.217840
C 0.072176 -0.029042 -0.171911
C -1.307177 0.452289 0.265067
C 1.393666 -3.545694 1.942556
C 2.380410 -2.567986 2.017583
C 3.197428 -2.287216 0.918460
C -5.452253 1.898610 0.262375
C -5.757524 -1.762078 0.472366
C 2.035297 -4.018593 -0.324338
C 3.015201 -3.034906 -0.247948
C 1.213698 -4.270525 0.768609
H 1.553872 4.218368 -0.726111
H 3.088340 0.479885 2.956773
H 0.003995 -0.148932 -4.124340
H -0.116124 -1.927853 -4.281759
H 1.417434 -1.160110 -3.827134
H 4.362377 0.979853 0.795795
H -0.339084 2.397473 2.203972
H -3.430867 -0.913600 1.946287
H 2.398096 -0.589389 -0.742767
H 2.382003 1.163176 -2.287623
H -1.548215 -2.241537 -0.240973
H 4.908493 -1.268874 0.134912
H 4.782383 -1.198488 1.881376
H 4.358519 -0.311568 -2.298491
H 5.256516 0.830414 -1.293133
H 4.747691 1.291172 -2.914796
H 5.085729 3.250713 -1.632916
H 4.024018 4.613041 -0.984802
H -0.633127 3.813705 -0.213640
H -1.541850 1.914562 -1.283865
H -3.166861 3.348294 -0.126077
H -2.986410 2.416735 1.349126
H -4.078337 1.377807 -1.298030
H -2.434109 -0.518749 -1.309052
H 0.265584 -1.034899 0.197113
H -1.307414 0.417763 1.355576
H 0.765994 -3.741261 2.803043
H 2.514054 -2.022012 2.941908
H -5.515712 1.894663 1.351131
H -6.261911 1.279559 -0.129193
H -5.620789 2.919479 -0.084453
H -6.253105 -1.103134 1.186117
H -5.175389 -2.477244 1.059313
H -6.487764 -2.294454 -0.133130
H 1.904926 -4.577287 -1.242177
H 3.645779 -2.841327 -1.107637
H 0.441057 -5.026366 0.707616
75
11-c3.out delta G = 0.8220 kcal/mol
conformer 3
O -0.051684 -0.424413 -1.212903
C -0.317422 -1.699220 -1.528526
C 1.782666 2.567436 -1.242077
O 0.367173 1.878533 2.681412
O -0.760263 -2.510242 -0.735923
C 1.038791 1.093759 2.010918
N 1.883843 0.184012 2.511366
C 0.033935 -2.002085 -2.950951
C 2.663422 -0.573865 1.540079
O -0.590192 3.684475 0.926374
O -4.207316 0.807571 1.681267
C 2.354803 0.169035 0.220143
C 3.510295 1.106808 -0.188010
O -2.702962 -1.366933 0.949389
C 3.084440 1.928251 -1.378468

O -5.174524 -0.945344 -1.282655
C 0.855309 2.191080 -0.352955
C 2.348558 -2.078036 1.559226
C 1.022036 0.925761 0.470968
C 3.996040 2.052595 0.925948
C 3.859860 2.114297 -2.451509
C -0.464609 2.925469 -0.281263
C -1.648897 1.970337 -0.513606
C -2.990638 2.663481 -0.291170
C -4.180871 1.739272 -0.545678
C -4.069988 0.457290 0.305872
C -5.170002 -0.535104 -0.138167
C -2.716875 -0.230882 0.077912
C -0.200153 0.007101 0.169117
C -1.543017 0.711464 0.353273
C 3.283787 -4.234311 -1.438112
C 2.537555 -3.563450 -0.473098
C 3.163744 -2.835112 0.538819
C -5.507946 2.464515 -0.320047
C -6.201816 -0.951684 0.868235
C 5.309843 -3.471088 -0.392871
C 4.561139 -2.803825 0.570010
C 4.673114 -4.186571 -1.403991
H 1.563783 3.398106 -1.905592
H 2.009853 0.091464 3.509298
H 1.115202 -2.145778 -3.014886
H -0.232607 -1.170365 -3.600576
H -0.463564 -2.914621 -3.267212
H 3.720004 -0.461242 1.780416
H -0.338732 3.104121 1.674675
H -3.875412 0.064458 2.203245
H 2.227518 -0.543638 -0.589977
H 4.347324 0.466596 -0.476156
H -2.056817 -1.996092 0.588334
H 2.563125 -2.451581 2.564554
H 1.287072 -2.248371 1.381415
H 4.328410 1.508851 1.810146
H 3.216747 2.752294 1.229641
H 4.840753 2.636151 0.559348
H 4.827754 1.635874 -2.538160
H 3.545338 2.750527 -3.270431
H -0.479586 3.666162 -1.082195
H -1.586247 1.658987 -1.562207
H -3.068465 3.528344 -0.955622
H -3.034134 3.043704 0.729917
H -4.143672 1.401875 -1.584957
H -2.700646 -0.560024 -0.963186
H -0.154849 -0.874493 0.801157
H -1.600910 0.997179 1.405708
H 2.777462 -4.793316 -2.215301
H 1.456734 -3.611811 -0.501211
H -6.364043 1.837887 -0.578436
H -5.554153 3.358417 -0.944308
H -5.611759 2.771534 0.721236
H -6.951797 -1.577698 0.389844
H -6.664261 -0.076445 1.326153
H -5.718217 -1.508306 1.675089
H 6.391427 -3.435051 -0.352394
H 5.071722 -2.257933 1.354248
H 5.256067 -4.705043 -2.154700
75
11-c8.out delta G = 1.0172 kcal/mol
conformer 4
O -0.128689 -0.061376 -1.544428
C 0.056500 -1.231893 -2.190651
C 1.726653 3.036965 -0.584117
O 0.399167 0.824080 2.757775
O 0.227144 -2.292395 -1.636848
C 1.259656 0.677249 1.886014
N 2.542041 0.403019 2.119637
C 0.043700 -1.020437 -3.677019
C 3.405203 0.266068 0.956077
O -0.680833 3.146417 1.811271
O -4.108167 0.263525 1.654017
C 2.397521 0.269065 -0.218035
C 2.899526 1.027053 -1.457449
O -2.544053 -1.670960 0.403232
C 2.957453 2.508827 -1.165785
O -5.960462 -1.097076 0.495303
C 0.811791 2.288700 0.043182
C 4.306567 -0.980301 1.071049
C 1.056032 0.819457 0.357545
C 4.176390 0.432210 -2.042638
C 3.985833 3.308523 -1.466695
C -0.532815 2.878861 0.411412
C -1.662044 1.990349 -0.143850
C -3.040529 2.548841 0.196694
C -4.169174 1.671763 -0.346103

C -4.020928 0.242945 0.232994
C -5.134198 -0.675450 -0.294151
C -2.648807 -0.354016 -0.135395
C -0.151384 -0.034243 -0.089490
C -1.501282 0.541941 0.338478
C 2.094278 -3.978086 1.886478
C 2.772905 -2.770061 2.005317
C 3.590910 -2.303295 0.972165
C -5.542209 2.273738 -0.053803
C -5.165541 -1.035384 -1.748214
C 3.051493 -4.302306 -0.294242
C 3.722332 -3.089641 -0.174333
C 2.230233 -4.748706 0.735269
H 1.529436 4.096008 -0.720047
H 2.893056 0.368848 3.066300
H -0.799639 -0.394599 -3.966730
H -0.002217 -1.979515 -4.185349
H 0.958054 -0.500723 -3.971190
H 4.057276 1.141450 0.894019
H -0.459657 2.332632 2.301804
H -4.941580 -0.190214 1.870913
H 2.222119 -0.767894 -0.502364
H 2.112934 0.913218 -2.208736
H -2.752159 -1.595860 1.345901
H 5.065746 -0.919990 0.292798
H 4.839801 -0.910517 2.023252
H 4.412387 0.896800 -3.000536
H 4.049152 -0.637055 -2.215102
H 5.036425 0.572547 -1.386694
H 4.895577 2.937017 -1.917018
H 3.937841 4.371869 -1.263780
H -0.610270 3.856166 -0.066897
H -1.548969 1.996994 -1.233024
H -3.138360 3.553478 -0.223177
H -3.144212 2.640758 1.278298
H -4.044645 1.594615 -1.430290
H -2.595756 -0.468041 -1.217444
H -0.038231 -1.054189 0.268829
H -1.517652 0.528568 1.429357
H 1.463420 -4.320990 2.697239
H 2.667362 -2.190250 2.912548
H -5.725141 2.311835 1.021251
H -6.349208 1.701295 -0.515677
H -5.594615 3.291848 -0.442343
H -4.890521 -0.196856 -2.386662
H -6.152740 -1.405368 -2.015721
H -4.434645 -1.830676 -1.919820
H 3.168681 -4.895475 -1.192619
H 4.360196 -2.749761 -0.981313
H 1.702882 -5.689876 0.643909

75

11-c2.out delta G = 1.3115 kcal/mol

conformer 5

O -0.472343 0.240536 -1.969281
C -0.807801 -0.645578 -2.917349
C 1.724471 2.630291 -0.122016
O 0.004941 -0.310089 2.384711
O -1.315912 -1.725701 -2.681623
C 0.859356 -0.298047 1.495415
N 2.076559 -0.829974 1.595182
C -0.457264 -0.144086 -4.284195
C 2.933011 -0.722140 0.423519
O -0.673789 2.328454 2.253493
O -4.568846 0.063241 1.211714
C 1.972769 -0.180868 -0.659112
C 2.620710 0.844378 -1.603973
O -3.244303 -1.441087 -0.646077
C 2.882618 2.134119 -0.860542
O -5.545656 0.448841 -2.207564
C 0.694288 1.866407 0.258764
C 3.560022 -2.080950 0.080346
C 0.717664 0.355683 0.096196
C 3.819616 0.282147 -2.361364
C 4.025280 2.826796 -0.908839
C -0.556317 2.495244 0.835355
C -1.801218 1.997734 0.073925
C -3.086725 2.568727 0.667830
C -4.340249 2.096503 -0.068794
C -4.394756 0.556013 -0.114929
C -5.560804 0.124679 -1.035843
C -3.104347 -0.017013 -0.715571
C -0.586653 -0.137513 -0.564021
C -1.857701 0.465275 0.030405
C 6.526657 -2.527141 2.420278
C 5.728923 -2.088560 1.369778
C 4.440642 -2.599700 1.188413
C -5.605378 2.697792 0.544699
C -6.678673 -0.677913 -0.438718

C 4.767127 -4.001969 3.139595
C 3.971388 -3.561180 2.085154
C 6.046872 -3.485113 3.310605
H 1.687226 3.695434 0.085115
H 2.387585 -1.257290 2.456526
H 0.623361 -0.227220 -4.420467
H -0.724346 0.906808 -4.385425
H -0.960589 -0.741943 -5.038623
H 3.727020 0.001663 0.625820
H -0.600086 1.376498 2.453729
H -4.321507 -0.871612 1.204697
H 1.651091 -1.032567 -1.261266
H 1.850136 1.085696 -2.341587
H -2.623220 -1.826660 -1.286536
H 2.753810 -2.788610 -0.123419
H 4.139148 -1.972212 -0.836051
H 3.549210 -0.650091 -2.859860
H 4.668501 0.084291 -1.705351
H 4.151102 0.983963 -3.127213
H 4.886575 2.485218 -1.465495
H 4.124653 3.767787 -0.380865
H -0.485695 3.573806 0.688746
H -1.698205 2.362829 -0.954005
H -3.048641 3.660938 0.633649
H -3.154827 2.285600 1.718753
H -4.274200 2.417728 -1.111758
H -3.069549 0.304385 -1.758677
H -0.622058 -1.223921 -0.513320
H -1.924945 0.084261 1.050422
H 7.523976 -2.123594 2.543818
H 6.111237 -1.344753 0.680124
H -6.500270 2.429949 -0.020623
H -5.534203 3.786648 0.551711
H -5.737391 2.358973 1.572854
H -7.463404 -0.824584 -1.177661
H -7.071545 -0.182407 0.449856
H -6.294822 -1.647720 -0.111840
H 4.387103 -4.749924 3.824295
H 2.976877 -3.971462 1.954117
H 6.668120 -3.827683 4.128461
75
11-c13.out delta G = 1.4113 kcal/mol
conformer 6
O -0.141938 0.372703 -1.918046
C -0.061758 -0.682974 -2.755077
C 1.629566 3.213104 -0.886768
O 0.623992 0.942545 2.519814
O 0.047210 -1.829823 -2.388177
C 1.258748 0.531086 1.546794
N 2.184674 -0.433145 1.586645
C -0.119371 -0.226092 -4.183260
C 2.893791 -0.697743 0.339244
O -0.602345 3.196327 1.684606
O -3.928716 0.114101 1.560000
C 2.437180 0.482558 -0.548722
C 3.508509 1.592375 -0.614484
O -2.398035 -1.616987 0.028547
C 2.944016 2.784822 -1.345311
O -5.821972 -1.193226 0.406614
C 0.802638 2.444789 -0.167141
C 2.622521 -2.099348 -0.229482
C 1.100603 0.973538 0.072643
C 4.042611 2.052777 0.754703
C 3.617270 3.445127 -2.293240
C -0.541302 2.988115 0.268794
C -1.693172 2.109054 -0.249715
C -3.052021 2.580452 0.256630
C -4.193297 1.711977 -0.275292
C -3.949999 0.239226 0.141583
C -5.070345 -0.666371 -0.393986
C -2.590181 -0.264001 -0.379978
C -0.111795 0.162405 -0.483743
C -1.445378 0.635627 0.091493
C 4.661920 -4.553757 1.844564
C 4.315779 -3.594408 0.899169
C 2.989547 -3.180188 0.752663
C -5.555445 2.224088 0.188100
C -5.199156 -0.884814 -1.870662
C 2.359363 -4.708457 2.526091
C 2.016958 -3.748940 1.577597
C 3.683551 -5.113402 2.662596
H 1.313455 4.217712 -1.149780
H 2.401031 -0.909180 2.450438
H -1.038538 0.334919 -4.355943
H -0.076537 -1.084586 -4.847371
H 0.717777 0.443393 -4.386407
H 3.965712 -0.628673 0.528859
H -0.243112 2.396527 2.122572

H -4.732066 -0.385229 1.789943
H 2.269536 0.141276 -1.567775
H 4.348170 1.189216 -1.185329
H -2.563556 -1.636097 0.982688
H 1.579253 -2.196580 -0.522317
H 3.219450 -2.195906 -1.139342
H 3.262592 2.522018 1.355171
H 4.831238 2.789860 0.602374
H 4.464481 1.228164 1.328621
H 4.597200 3.119576 -2.620404
H 3.205360 4.329582 -2.764885
H -0.659305 3.980649 -0.168934
H -1.682148 2.205307 -1.340006
H -3.218824 3.614584 -0.056430
H -3.064993 2.568766 1.346821
H -4.159379 1.746506 -1.368198
H -2.613912 -0.277715 -1.469320
H 0.018003 -0.897985 -0.296049
H -1.387142 0.529726 1.176941
H 5.694101 -4.867017 1.941380
H 5.081932 -3.164268 0.264183
H -5.674812 3.271976 -0.090963
H -5.646111 2.150329 1.273015
H -6.379832 1.665967 -0.260432
H -6.198609 -1.244785 -2.104433
H -4.474585 -1.648407 -2.167542
H -4.977925 0.015041 -2.442755
H 1.591688 -5.141131 3.155628
H 0.984034 -3.438419 1.472888
H 3.951436 -5.861697 3.397858
75
11-c12.out delta G = 1.4276 kcal/mol conformer 7
O -0.161949 -0.311323 -1.302963
C -0.205263 -1.595301 -1.708418
C 1.789477 2.554137 -1.142156
O 0.236946 1.775736 2.707075
O -0.366631 -2.533269 -0.961371
C 0.904392 0.989549 2.032727
N 1.697219 0.033197 2.530339
C 0.014957 -1.695598 -3.188596
C 2.514913 -0.692621 1.565546
O -0.647082 3.648567 0.978445
O -4.266917 0.967712 1.556210
C 2.265044 0.097779 0.259918
C 3.450971 1.024397 -0.081391
O -2.806073 -1.300294 0.947780
C 3.079941 1.888657 -1.259695
O -6.183032 -0.532712 0.715846
C 0.827103 2.171763 -0.293988
C 2.203535 -2.196719 1.509640
C 0.938922 0.874203 0.490958
C 3.912289 1.928145 1.076951
C 3.890138 2.085108 -2.304900
C -0.482274 2.929583 -0.249171
C -1.672375 2.003163 -0.557386
C -3.010796 2.720273 -0.420831
C -4.189357 1.801211 -0.743779
C -4.148108 0.568371 0.194397
C -5.308613 -0.388304 -0.119600
C -2.810118 -0.185637 0.058017
C -0.296860 0.001980 0.107170
C -1.619031 0.741131 0.311249
C 5.493608 -3.663166 0.279586
C 4.544151 -3.031966 1.074742
C 3.214049 -2.925614 0.656790
C -5.521560 2.543644 -0.660230
C -5.327662 -1.120704 -1.426771
C 3.805744 -4.109108 -1.373945
C 2.857189 -3.475733 -0.574687
C 5.127485 -4.201805 -0.951756
H 1.609422 3.408472 -1.787092
H 1.790164 -0.091101 3.528417
H -0.512316 -0.902067 -3.715431
H -0.305515 -2.671373 -3.543346
H 1.082953 -1.577523 -3.387359
H 3.560138 -0.594107 1.857914
H -0.418242 3.043928 1.714917
H -5.137784 0.645253 1.848097
H 2.151407 -0.588296 -0.575699
H 4.287018 0.378423 -0.359605
H -3.062874 -0.951344 1.814170
H 2.233834 -2.583340 2.531899
H 1.198160 -2.366293 1.126831
H 4.204741 1.352668 1.955139
H 3.133646 2.631477 1.373694
H 4.778589 2.508180 0.758163
H 4.848836 1.586274 -2.377402

H 3.613915 2.750316 -3.114604
H -0.451452 3.696505 -1.024773
H -1.555196 1.693904 -1.600791
H -3.034545 3.577726 -1.098637
H -3.119745 3.109346 0.591864
H -4.057981 1.432778 -1.765693
H -2.741781 -0.600386 -0.947172
H -0.299946 -0.928422 0.665785
H -1.667441 1.033926 1.362275
H 6.518461 -3.738034 0.621642
H 4.839372 -2.622790 2.034195
H -6.360557 1.922513 -0.980357
H -5.498285 3.424929 -1.302950
H -5.714253 2.875027 0.361410
H -6.338380 -1.458022 -1.645349
H -4.679423 -1.996997 -1.336161
H -4.945340 -0.514989 -2.246949
H 3.509078 -4.531074 -2.326346
H 1.828138 -3.417101 -0.903018
H 5.865876 -4.693810 -1.572344

75

11-c4.out delta G = 2.0607 kcal/mol conformer 8

O -0.086393 0.281472 -1.920364
C -0.317900 -0.819193 -2.652397
C 1.635575 3.243884 -0.956591
O 0.742009 0.993641 2.491268
O -0.594108 -1.901506 -2.169989
C 1.344026 0.575354 1.501805
N 2.263437 -0.396994 1.513078
C -0.179263 -0.542143 -4.116701
C 2.933281 -0.664029 0.243965
O -0.513984 3.236123 1.668947
O -3.890805 0.020968 1.621692
C 2.461629 0.521779 -0.626424
C 3.528130 1.633650 -0.709309
O -2.401295 -1.597763 -0.017220
C 2.946494 2.823138 -1.432071
O -5.146379 -0.587811 -1.671960
C 0.825666 2.474290 -0.218951
C 2.630382 -2.059460 -0.327316
C 1.139665 1.009481 0.028586
C 4.085319 2.093776 0.650268
C 3.603097 3.487587 -2.388657
C -0.506260 3.011337 0.254348
C -1.671265 2.120670 -0.213414
C -3.002924 2.568069 0.382677
C -4.176764 1.706283 -0.082885
C -3.909453 0.214917 0.209164
C -5.015126 -0.632244 -0.464109
C -2.568242 -0.225673 -0.393154
C -0.074793 0.171182 -0.470736
C -1.409269 0.644399 0.098745
C 2.390287 -4.688878 2.411645
C 2.039870 -3.718172 1.477593
C 3.002552 -3.153316 0.638764
C -5.494243 2.183538 0.529036
C -5.892944 -1.479641 0.408744
C 4.680795 -4.552343 1.687149
C 4.326947 -3.582152 0.755878
C 3.712499 -5.108360 2.519470
H 1.309108 4.245473 -1.217961
H 2.505410 -0.872456 2.370395
H -0.489192 -1.412021 -4.688197
H 0.862752 -0.306160 -4.340362
H -0.779442 0.325458 -4.390752
H 4.011157 -0.608197 0.401716
H -0.140069 2.441228 2.103242
H -3.462158 -0.828964 1.791433
H 2.268669 0.187955 -1.643037
H 4.358461 1.232480 -1.295032
H -1.769608 -1.993721 -0.640106
H 1.577802 -2.145499 -0.593148
H 3.203673 -2.157863 -1.251794
H 4.870049 2.832185 0.485094
H 4.518151 1.268494 1.215203
H 3.315357 2.561170 1.265088
H 4.580869 3.168562 -2.728445
H 3.179100 4.368993 -2.855208
H -0.648282 3.998090 -0.189165
H -1.720327 2.230026 -1.302482
H -3.197250 3.607260 0.103352
H -2.936892 2.538992 1.470667
H -4.252723 1.780208 -1.170966
H -2.665070 -0.145129 -1.477949
H 0.080562 -0.871507 -0.215612
H -1.347946 0.515452 1.181424
H 1.630198 -5.118411 3.052368

H 1.008110 -3.397002 1.396621
H -5.657284 3.235126 0.287341
H -5.477773 2.083264 1.614881
H -6.348972 1.621475 0.147332
H -5.289812 -2.262058 0.876778
H -6.679948 -1.933772 -0.189254
H -6.316016 -0.884766 1.218864
H 5.711229 -4.877082 1.761659
H 5.085285 -3.155477 0.109334
H 3.986646 -5.865141 3.243619

**Table S31.** Geometry data of conformers of compound **12**.

78
12-c4.out delta G = 0.0000 kcal/mol conformer 1
O -0.391593 0.000245 -2.042093
C -0.803659 -1.106676 -2.678352
C 2.233857 2.929495 -0.090812
O 0.877844 1.021959 2.229259
C 1.313473 0.392925 1.268092
N 2.135587 -0.663846 1.333948
O -1.152389 -2.121877 -2.107296
O 2.127665 3.540071 1.194515
C 2.749139 -1.065843 0.075747
O -0.368978 4.218947 -0.377434
O -4.000578 0.444377 1.673469
C 2.227272 -0.011511 -0.933537
C 3.295122 1.031993 -1.358195
C -0.801211 -0.921630 -4.165103
O -2.816266 -1.415278 0.077495
C 3.450655 2.032151 -0.238171
O -5.472956 -0.098599 -1.541022
C 0.939262 2.183796 -0.520079
C 2.412150 -2.512835 -0.317408
C 1.024611 0.677361 -0.220524
C 4.584534 0.400564 -1.872295
C 4.498370 2.112430 0.574168
C -0.313443 2.854285 0.059028
C -1.597911 2.154833 -0.368217
C -2.820226 2.794981 0.289344
C -4.126157 2.093569 -0.082242
C -4.056777 0.590406 0.256432
C -5.297787 -0.116219 -0.339067
C -2.818576 -0.057115 -0.377889
C -0.302321 -0.018132 -0.584978
C -1.527493 0.668682 0.007257
C 2.364098 -4.816882 2.709351
C 1.948175 -3.953960 1.699744
C 2.846811 -3.499576 0.733240
C -5.331251 2.770942 0.571205
C -6.246798 -0.798274 0.602746
C 4.593679 -4.791986 1.806020
C 4.174352 -3.929336 0.799486
C 3.688830 -5.237639 2.765826
H 2.361774 3.769818 -0.777745
H 2.463722 -1.004629 2.225590
H 1.856397 2.857623 1.828956
H 3.830263 -0.990106 0.191485
H 0.269047 4.704441 0.160539
H -3.672348 -0.446485 1.856449
H 1.893452 -0.516840 -1.836928
H 2.855168 1.568052 -2.205673
H -0.840469 -1.890533 -4.655271
H 0.074418 -0.359629 -4.485823
H -1.687711 -0.347628 -4.444554
H -2.284072 -1.935148 -0.546397
H 0.857490 2.282046 -1.604758
H 1.339011 -2.602078 -0.495228
H 2.915303 -2.721440 -1.264507
H 5.138641 -0.128649 -1.097274
H 5.243676 1.168861 -2.278133
H 4.364063 -0.313107 -2.668986
H 5.366295 1.478961 0.449590
H 4.513108 2.822620 1.390006
H -0.270944 2.826966 1.147131
H -1.689885 2.240945 -1.456942
H -2.882145 3.843339 -0.002648
H -2.694660 2.770156 1.375986
H -4.247973 2.143495 -1.167720
H -2.961943 -0.024965 -1.460049
H -0.267276 -1.057691 -0.269706
H -1.424352 0.595109 1.092854
H 1.652642 -5.161860 3.449271
H 0.914970 -3.629964 1.659251
H -6.273813 2.326928 0.244211
H -5.355078 3.828491 0.303194
H -5.277480 2.696023 1.657859

H -5.739694 -1.643359 1.075889
H -7.116452 -1.153999 0.054637
H -6.542019 -0.121912 1.405522
H 5.625369 -5.119583 1.839866
H 4.883499 -3.588591 0.053407
H 4.013790 -5.910897 3.548974
78
12-c2.out delta G = 0.0289 kcal/mol
conformer 2
O -0.359549 -0.655868 -1.271096
C -0.762875 -1.915949 -1.481856
C 2.246283 2.738212 -0.415989
O 0.643659 1.924796 2.365577
C 1.128057 0.968320 1.765961
N 1.929714 0.038036 2.304835
O -1.223627 -2.630844 -0.612078
O 2.098199 3.807488 0.518065
C 2.592506 -0.851865 1.362752
O -0.275046 3.854734 -1.365728
O -4.201450 1.214158 1.694589
C 2.163233 -0.295292 -0.019794
C 3.285993 0.474418 -0.765827
C -0.539018 -2.337377 -2.901581
O -2.993461 -1.132102 1.015597
C 3.431447 1.833624 -0.125547
O -5.477761 -0.477863 -1.186547
C 0.948179 1.905775 -0.603089
C 2.211642 -2.324151 1.606564
C 0.947475 0.637103 0.270042
C 4.571271 -0.328985 -0.928042
C 4.444459 2.210562 0.646503
C -0.307249 2.771093 -0.426748
C -1.591329 1.981832 -0.645098
C -2.824882 2.843919 -0.377079
C -4.132104 2.073783 -0.557995
C -4.153577 0.811344 0.327965
C -5.379897 -0.050733 -0.053576
C -2.903698 -0.046611 0.085657
C -0.379334 -0.124571 0.090568
C -1.612605 0.752056 0.273448
C 4.950314 -4.295313 -0.146443
C 4.286547 -3.484752 0.766438
C 2.912713 -3.259475 0.656889
C -5.347335 2.966985 -0.306348
C -6.410933 -0.326388 1.002262
C 2.875962 -4.692681 -1.297083
C 2.215446 -3.880420 -0.379935
C 4.247044 -4.897723 -1.186462
H 2.442896 3.239036 -1.367277
H 2.199784 0.098996 3.275708
H 1.760911 3.437968 1.349059
H 3.669385 -0.749522 1.493863
H 0.347617 4.503510 -1.014452
H -3.934740 0.452755 2.227385
H 1.868313 -1.121180 -0.659990
H 2.898085 0.638649 -1.776648
H -1.108169 -3.238464 -3.111779
H 0.525007 -2.544425 -3.039234
H -0.814560 -1.538512 -3.588232
H -2.443085 -1.856163 0.675564
H 0.932496 1.567250 -1.640950
H 2.469928 -2.573488 2.639277
H 1.130416 -2.437888 1.513061
H 5.078680 -0.517145 0.017427
H 5.267985 0.207106 -1.573862
H 4.357735 -1.298477 -1.380233
H 5.291874 1.564133 0.831678
H 4.450493 3.186117 1.114012
H -0.333484 3.173750 0.584787
H -1.612352 1.644068 -1.687561
H -2.819916 3.701704 -1.049717
H -2.773288 3.235131 0.643476
H -4.177598 1.708773 -1.587566
H -2.969725 -0.423943 -0.937065
H -0.413451 -0.969080 0.774030
H -1.590261 1.098644 1.309508
H 6.016320 -4.457797 -0.047107
H 4.842635 -3.018708 1.571820
H -6.284325 2.446682 -0.515695
H -5.305887 3.844247 -0.954088
H -5.369962 3.308316 0.729083
H -6.737862 0.602152 1.471577
H -5.963056 -0.936071 1.791454
H -7.253166 -0.857245 0.563988
H 2.317616 -5.168522 -2.093774
H 1.144736 -3.739527 -0.459468
H 4.763105 -5.529091 -1.898644
78

12-c8.out delta G = 0.2805 kcal/mol
conformer 3
O -0.558412 -0.590804 -1.334911
C -0.614810 -1.912761 -1.594466
C 2.225895 2.635339 -0.375049
O 0.549549 1.819032 2.375250
C 1.014142 0.860012 1.762075
N 1.771335 -0.109360 2.294816
O -0.599584 -2.771153 -0.743581
O 2.111033 3.679041 0.592628
C 2.467799 -0.964414 1.344043
O -0.237444 3.855612 -1.342951
O -4.187053 1.415114 1.667781
C 2.040227 -0.396174 -0.036308
C 3.183641 0.343802 -0.780915
C -0.659637 -2.163360 -3.074178
O -3.109790 -1.059741 1.005563
C 3.379207 1.682889 -0.112506
O -6.356562 0.196455 1.007533
C 0.902738 1.853040 -0.588732
C 2.141989 -2.453099 1.551442
C 0.847060 0.566748 0.259231
C 4.439461 -0.502116 -0.961368
C 4.405420 2.004229 0.667566
C -0.322111 2.762944 -0.416877
C -1.628591 2.020735 -0.662535
C -2.839606 2.925840 -0.444544
C -4.158376 2.182565 -0.655488
C -4.226963 0.975810 0.314618
C -5.531419 0.190673 0.113060
C -3.020386 0.041104 0.105424
C -0.510430 -0.136972 0.048440
C -1.698186 0.795814 0.262267
C 5.232412 -4.256193 0.268197
C 4.376925 -3.507419 1.066953
C 3.035778 -3.333896 0.716353
C -5.362318 3.110244 -0.506428
C -5.747637 -0.564933 -1.163770
C 3.420608 -4.692854 -1.251310
C 2.566087 -3.942819 -0.447672
C 4.757155 -4.847171 -0.899776
H 2.441035 3.159252 -1.309887
H 2.029529 -0.070815 3.270268
H 1.746625 3.297479 1.406704
H 3.539269 -0.826206 1.490811
H 0.401402 4.478974 -0.975485
H -5.073492 1.235636 2.027499
H 1.723499 -1.218164 -0.671839
H 2.797817 0.539670 -1.786820
H -0.953783 -3.192056 -3.263480
H 0.336828 -1.994233 -3.488728
H -1.343682 -1.471980 -3.564235
H -3.251750 -0.678702 1.884476
H 0.886653 1.533291 -1.632409
H 2.279457 -2.685109 2.610973
H 1.094712 -2.633648 1.308201
H 4.191397 -1.453957 -1.433095
H 4.938516 -0.730027 -0.020760
H 5.154949 0.022450 -1.596206
H 5.229999 1.324043 0.833977
H 4.445283 2.966376 1.160546
H -0.348171 3.155451 0.598630
H -1.634269 1.674724 -1.701986
H -2.788591 3.769870 -1.132139
H -2.815807 3.332677 0.570427
H -4.151355 1.778203 -1.672808
H -3.072808 -0.385089 -0.896304
H -0.595763 -1.009885 0.689289
H -1.644927 1.146241 1.296035
H 6.269160 -4.379833 0.555946
H 4.755531 -3.049416 1.973995
H -5.257700 3.969032 -1.171143
H -5.436872 3.481598 0.516723
H -6.301040 2.610368 -0.754335
H -5.210366 -1.514724 -1.091770
H -5.359141 -0.032240 -2.030897
H -6.807362 -0.773577 -1.292379
H 3.039350 -5.159371 -2.151489
H 1.523910 -3.833448 -0.718449
H 5.422140 -5.430098 -1.524636
78
12-c3.out delta G = 0.5943 kcal/mol
conformer 4
O 0.113537 -0.321785 -1.730301
C -0.278246 -1.463831 -2.312334
C 2.272296 3.048554 -0.387250
O 0.840408 1.388677 2.379316
C 1.526955 0.765850 1.573995

N 2.623373 0.059559 1.887950
O -0.772678 -2.397293 -1.717070
O 2.181567 3.864820 0.784216
C 3.414524 -0.446582 0.782699
O -0.441903 3.970627 -0.809003
O -3.674830 0.167077 1.879817
C 2.638277 0.037582 -0.469673
C 3.478522 1.030534 -1.311702
C -0.009942 -1.436201 -3.787805
O -2.297853 -1.745955 0.518281
C 3.592724 2.307415 -0.513884
O -5.069418 -0.900389 -1.235978
C 1.072696 2.091615 -0.563069
C 3.676538 -1.960109 0.866155
C 1.315679 0.692576 0.048943
C 4.793081 0.426813 -1.794823
C 4.702038 2.739376 0.076641
C -0.242646 2.734294 -0.106274
C -1.442976 1.836714 -0.383765
C -2.723941 2.434140 0.197931
C -3.951808 1.565014 -0.067705
C -3.732292 0.131641 0.455369
C -4.890941 -0.764399 -0.042031
C -2.429492 -0.462495 -0.099379
C 0.091995 -0.191621 -0.272660
C -1.221267 0.431254 0.192748
C 0.441765 -3.741202 1.801935
C 1.564285 -2.923600 1.870894
C 2.460144 -2.845735 0.800416
C -5.219397 2.196547 0.508899
C -5.762948 -1.432775 0.981266
C 1.092208 -4.448494 -0.400268
C 2.205909 -3.618821 -0.334037
C 0.203501 -4.508705 0.666242
H 2.221357 3.767294 -1.208484
H 2.952841 0.039960 2.842262
H 2.045042 3.297429 1.555319
H 4.388346 0.044270 0.819605
H 0.112593 4.630882 -0.375985
H -3.245804 -0.650798 2.166233
H 2.401048 -0.817630 -1.098561
H 2.886539 1.249186 -2.205477
H -0.410259 -0.522007 -4.225963
H -0.454800 -2.307300 -4.259969
H 1.068927 -1.437258 -3.955172
H -1.712833 -2.274160 -0.050216
H 0.965071 1.933272 -1.637033
H 4.357795 -2.214089 0.051722
H 4.221444 -2.149864 1.795587
H 4.598922 -0.470425 -2.385901
H 5.457499 0.146781 -0.976744
H 5.329556 1.137740 -2.424526
H 5.646182 2.222630 -0.031711
H 4.691683 3.633492 0.685743
H -0.203558 2.931762 0.963588
H -1.550649 1.755540 -1.471746
H -2.888111 3.424522 -0.227776
H -2.598706 2.562536 1.277361
H -4.075475 1.464630 -1.149435
H -2.564606 -0.577849 -1.176265
H 0.223315 -1.183181 0.154514
H -1.139505 0.518828 1.277925
H -0.245896 -3.780393 2.637228
H 1.745202 -2.342225 2.765113
H -5.158142 2.264829 1.595542
H -6.112943 1.623739 0.252041
H -5.348279 3.204334 0.110530
H -6.135530 -0.703786 1.701766
H -5.167918 -2.155586 1.545370
H -6.585604 -1.943057 0.485059
H 0.909956 -5.035079 -1.291223
H 2.894060 -3.575951 -1.170468
H -0.671018 -5.144330 0.611520

**Table S32.** Geometry data of conformers of compound **13**.

78
13-c19.out delta G = 0.0000 kcal/mol
conformer 1
O -0.296652 -0.782790 -1.255355
C 0.178247 -2.017242 -1.511823
C 0.982360 3.252134 -0.348551
O -0.359557 1.782262 2.438282
C 0.535757 1.218442 1.817359
N 1.692231 0.787017 2.342733
O 0.530723 -2.797566 -0.656110
O 0.793830 4.116682 -1.499628
C 2.663508 0.228876 1.418411

O -1.814493 3.343802 -1.420670
O -4.390361 -0.383658 1.809493
C 2.012042 0.451010 0.028699
C 2.749586 1.513718 -0.828017
C 0.236317 -2.268658 -2.989727
O -2.426124 -2.222283 1.122962
C 2.440157 2.857117 -0.228303
O -5.891589 -2.378702 1.186102
C 0.069642 2.040066 -0.568220
C 2.978776 -1.244177 1.738578
C 0.529403 0.859141 0.315333
C 4.225548 1.201793 -1.045602
C 3.306805 3.644809 0.400882
C -1.419072 2.393744 -0.418034
C -2.324386 1.174698 -0.577164
C -3.790235 1.530108 -0.334487
C -4.712772 0.323537 -0.511333
C -4.273961 -0.805786 0.454090
C -5.156729 -2.049931 0.271844
C -2.797764 -1.182373 0.219695
C -0.426421 -0.335985 0.123957
C -1.886121 0.038689 0.354867
C 4.958717 -2.986199 -1.009130
C 3.899549 -2.576535 -0.203257
C 4.123909 -1.761671 0.907019
C -6.181235 0.701277 -0.327179
C -5.081990 -2.825567 -1.007834
C 6.494310 -1.785961 0.400681
C 5.434580 -1.378193 1.201965
C 6.258382 -2.588651 -0.712985
H 0.669881 3.794329 0.548641
H 1.892715 0.919785 3.323412
H 1.306086 4.922230 -1.355571
H 3.586823 0.802223 1.502800
H -1.053934 3.926720 -1.581191
H -5.118507 -0.909532 2.184651
H 2.046196 -0.481493 -0.526633
H 2.274654 1.482322 -1.813220
H 1.043178 -1.669564 -3.417253
H -0.693317 -1.961363 -3.467314
H 0.431403 -3.320763 -3.177525
H -2.679856 -1.916310 2.006035
H 0.201529 1.721834 -1.605471
H 3.230905 -1.313658 2.799781
H 2.084662 -1.847042 1.573529
H 4.343068 0.197533 -1.454459
H 4.809050 1.248668 -0.127052
H 4.658098 1.913507 -1.750452
H 4.361067 3.409610 0.462960
H 2.984898 4.566122 0.873518
H -1.579698 2.826024 0.574083
H -2.218736 0.832802 -1.612152
H -4.095092 2.314092 -1.027847
H -3.907835 1.927828 0.677857
H -4.574140 -0.052128 -1.529514
H -2.693698 -1.602161 -0.780491
H -0.139120 -1.160248 0.770886
H -1.960716 0.390145 1.385284
H 4.766759 -3.616349 -1.868950
H 2.890129 -2.891076 -0.435451
H -6.443395 1.518744 -1.000390
H -6.368908 1.031661 0.695695
H -6.851307 -0.133577 -0.542465
H -5.967814 -3.447604 -1.114675
H -4.202152 -3.473682 -0.964109
H -4.967532 -2.178491 -1.876278
H 7.503825 -1.478174 0.643775
H 5.625179 -0.750236 2.065027
H 7.082232 -2.905869 -1.340013
78
13-c21.out delta G = 0.5390 kcal/mol
conformer 2
O -0.350106 0.239540 -2.024208
C -0.835053 -0.707248 -2.839600
C 2.428851 2.546820 0.326148
O 0.515837 0.541276 2.376017
C 1.111765 0.062439 1.417387
N 1.971470 -0.966077 1.470119
O -1.358614 -1.732558 -2.445401
O 2.634181 3.856042 -0.265198
C 2.639506 -1.323201 0.230672
O -0.058570 4.189036 -0.004808
O -4.113895 0.553213 1.560210
C 2.162191 -0.239669 -0.768114
C 3.295636 0.725724 -1.198330
C -0.659725 -0.332482 -4.278887
O -3.056272 -1.174414 -0.265081
C 3.580707 1.626090 -0.027665

O -5.477905 0.658601 -1.744011
C 1.088077 2.040366 -0.219978
C 2.312682 -2.753833 -0.225563
C 0.990963 0.509136 -0.056923
C 4.496686 0.004596 -1.799553
C 4.695781 1.626285 0.696227
C -0.111008 2.802216 0.366564
C -1.448937 2.259968 -0.133939
C -2.622460 2.938736 0.572931
C -3.977914 2.444648 0.067536
C -4.082458 0.909616 0.179917
C -5.359330 0.437887 -0.554211
C -2.887117 0.228448 -0.502667
C -0.367939 0.012928 -0.582464
C -1.552090 0.740592 0.041034
C 2.204868 -5.231000 2.662382
C 1.807497 -4.314072 1.693007
C 2.729605 -3.787480 0.786804
C -5.133024 3.149620 0.779476
C -6.413291 -0.278153 0.238150
C 4.462921 -5.115375 1.839424
C 4.062266 -4.199164 0.873445
C 3.534181 -5.634124 2.738550
H 2.367501 2.660067 1.412539
H 2.179085 -1.422137 2.346435
H 3.439946 4.232550 0.110052
H 3.715268 -1.251660 0.391633
H 0.878568 4.442294 -0.041592
H -3.896066 -0.387217 1.618248
H 1.796432 -0.725636 -1.669712
H 2.873713 1.350018 -1.991805
H 0.378585 -0.064019 -4.473326
H -1.273532 0.543478 -4.496900
H -0.963655 -1.158298 -4.915427
H -2.520797 -1.647855 -0.923540
H 1.083300 2.240828 -1.294546
H 1.241314 -2.831325 -0.421050
H 2.830013 -2.922426 -1.172702
H 5.224387 0.727218 -2.171193
H 4.178740 -0.618003 -2.637855
H 5.008056 -0.639067 -1.083346
H 5.549627 1.012385 0.441674
H 4.794057 2.255789 1.573697
H -0.080891 2.718010 1.456521
H -1.502439 2.490668 -1.203804
H -2.563460 4.017404 0.424446
H -2.547468 2.756709 1.649235
H -4.044161 2.663661 -1.001503
H -2.965933 0.437968 -1.571818
H -0.450944 -1.058855 -0.416386
H -1.521503 0.510894 1.107029
H 1.475369 -5.631969 3.355105
H 0.770131 -4.005974 1.636354
H -6.102253 2.861372 0.367271
H -5.035129 4.230589 0.666906
H -5.133090 2.916983 1.844987
H -6.693982 0.304879 1.116104
H -6.008779 -1.224024 0.607375
H -7.280212 -0.470533 -0.390077
H 5.498687 -5.427774 1.888920
H 4.789627 -3.802397 0.174395
H 3.844443 -6.349526 3.489689
78
13-c11.out delta G = 0.6256 kcal/mol conformer 3
O -0.545572 -0.393962 -1.375866
C -0.665919 -1.667876 -1.800157
C 2.407465 2.447519 0.027218
O 0.353040 1.459535 2.612788
C 0.936893 0.623471 1.929662
N 1.751501 -0.332536 2.399086
O -0.752406 -2.621282 -1.059770
O 2.785193 3.436563 -0.945492
C 2.406078 -1.176234 1.414254
O 0.089552 3.993885 -0.903248
O -4.159036 1.457113 1.752776
C 1.994045 -0.541956 0.060290
C 3.173417 0.140349 -0.679512
C -0.644913 -1.733443 -3.298732
O -3.231105 -0.967660 0.773899
C 3.506612 1.403509 0.066702
O -6.375268 0.496540 0.864473
C 1.045441 1.822027 -0.328520
C 1.997553 -2.653270 1.566997
C 0.852866 0.471396 0.395918
C 4.339653 -0.802807 -0.951855
C 4.625172 1.619361 0.750504
C -0.116514 2.801573 -0.106203

C -1.481077 2.187257 -0.415017
C -2.625535 3.147720 -0.094069
C -3.990873 2.546863 -0.430942
C -4.176685 1.221178 0.348686
C -5.522875 0.568370 -0.002490
C -3.030816 0.237522 0.039049
C -0.532777 -0.111337 0.051776
C -1.667590 0.864343 0.340496
C 4.927559 -4.620141 0.156943
C 4.138148 -3.852842 1.005174
C 2.813834 -3.553275 0.676009
C -5.123639 3.536323 -0.164848
C -5.743305 0.030655 -1.383382
C 3.080528 -4.820821 -1.370927
C 2.292244 -4.052551 -0.518075
C 4.401513 -5.103107 -1.038969
H 2.330613 2.926731 1.010095
H 1.937254 -0.410629 3.388540
H 1.989834 3.981955 -1.073634
H 3.483977 -1.100577 1.558436
H -0.169855 3.796799 -1.813789
H -5.067185 1.285203 2.058228
H 1.621910 -1.326305 -0.592114
H 2.778215 0.436923 -1.656033
H -0.939510 -2.725737 -3.628587
H 0.369217 -1.523774 -3.645681
H -1.303924 -0.977966 -3.724917
H -3.367228 -0.703665 1.695721
H 1.062593 1.598816 -1.398792
H 2.140648 -2.938593 2.612296
H 0.934957 -2.755946 1.341731
H 4.834527 -1.138984 -0.041666
H 5.086848 -0.305259 -1.571988
H 3.991110 -1.691810 -1.478975
H 5.439244 0.906522 0.765998
H 4.765974 2.534545 1.313961
H -0.101242 3.151698 0.924614
H -1.503504 1.966516 -1.490197
H -2.501069 4.075179 -0.653933
H -2.596525 3.406282 0.968128
H -3.988737 2.296900 -1.496076
H -3.075684 -0.041382 -1.013385
H -0.695282 -1.046771 0.580039
H -1.634036 1.076803 1.410105
H 5.952620 -4.841464 0.427515
H 4.555491 -3.476822 1.932507
H -4.942969 4.465212 -0.707663
H -5.187597 3.772425 0.898464
H -6.092401 3.147995 -0.485256
H -5.288935 0.658193 -2.148656
H -6.809011 -0.080630 -1.570244
H -5.272525 -0.954577 -1.445446
H 2.661038 -5.198082 -2.295561
H 1.264222 -3.836760 -0.780021
H 5.015382 -5.699283 -1.702496
78
13-c12.out delta G = 0.8747 kcal/mol
conformer 4
O -0.328087 0.249291 -2.031939
C -0.775041 -0.694134 -2.872931
C 2.397981 2.570543 0.365667
O 0.421411 0.493102 2.387408
C 1.057302 0.040678 1.440769
N 1.942143 -0.964401 1.508544
O -1.314577 -1.721654 -2.507371
O 2.689283 3.857346 -0.204796
C 2.645294 -1.300203 0.282798
O 0.009578 4.160074 0.123791
O -4.182100 0.493532 1.456250
C 2.154057 -0.232827 -0.726489
C 3.272944 0.747833 -1.155492
C -0.514994 -0.322528 -4.300076
O -3.073373 -1.195308 -0.373727
C 3.552153 1.651678 0.014591
O -5.462336 0.665432 -1.878722
C 1.057563 2.034157 -0.174561
C 2.371291 -2.740486 -0.177145
C 0.964924 0.500289 -0.031370
C 4.479118 0.038192 -1.760770
C 4.670369 1.656357 0.731918
C -0.149391 2.752580 0.433313
C -1.477991 2.240309 -0.126682
C -2.673948 2.899278 0.561275
C -4.013328 2.413136 0.006002
C -4.116502 0.876414 0.084666
C -5.372980 0.416926 -0.692014
C -2.902289 0.211469 -0.580449
C -0.379762 0.003269 -0.594232

C -1.583708 0.715441 0.009385
C 4.563336 -5.027871 1.926778
C 4.148633 -4.126960 0.952389
C 2.804712 -3.759694 0.842930
C -5.189345 3.101713 0.699341
C -6.442076 -0.322490 0.056989
C 2.297032 -5.216830 2.712658
C 1.885630 -4.315170 1.734823
C 3.637496 -5.575580 2.811629
H 2.331361 2.667033 1.455723
H 2.138732 -1.421740 2.386788
H 1.854284 4.353875 -0.144109
H 3.714856 -1.193391 0.464806
H -0.457655 4.679591 0.786952
H -3.963310 -0.447401 1.502359
H 1.803054 -0.733680 -1.625888
H 2.842566 1.368180 -1.947725
H -0.890227 0.681595 -4.497914
H -0.990439 -1.040303 -4.962028
H 0.561911 -0.313080 -4.478227
H -2.516742 -1.655482 -1.024385
H 1.039684 2.248827 -1.246355
H 1.305766 -2.850420 -0.389044
H 2.908521 -2.893281 -1.115864
H 4.166065 -0.579239 -2.604825
H 4.992451 -0.608807 -1.048865
H 5.204021 0.767407 -2.124927
H 5.522012 1.038490 0.478405
H 4.772869 2.297194 1.600002
H -0.136874 2.625415 1.516269
H -1.497110 2.498724 -1.191375
H -2.628750 3.983077 0.434154
H -2.630821 2.696756 1.635626
H -4.048302 2.654536 -1.059528
H -2.952943 0.441736 -1.647035
H -0.461175 -1.071069 -0.443711
H -1.582069 0.464051 1.070994
H 5.607940 -5.305591 1.994107
H 4.873941 -3.706942 0.264887
H -5.092113 4.184943 0.610658
H -5.218500 2.847777 1.759500
H -6.145305 2.818960 0.254116
H -6.747065 0.238104 0.941419
H -6.041641 -1.275058 0.413220
H -7.292383 -0.503815 -0.596657
H 1.569857 -5.640548 3.394200
H 0.839799 -4.041369 1.660405
H 3.958772 -6.279000 3.569423

78

13-c15.out delta G = 0.8898 kcal/mol

conformer 5

O -0.300656 -0.785292 -1.254501
C 0.181893 -2.015404 -1.517943
C 0.998493 3.236776 -0.284347
O -0.378664 1.727533 2.461571
C 0.533649 1.204306 1.829830
N 1.719170 0.839941 2.340730
O 0.540451 -2.798098 -0.666921
O 0.730001 4.324103 -1.206549
C 2.683219 0.266146 1.419287
O -1.811297 3.354818 -1.376115
O -4.398019 -0.407190 1.803974
C 2.005383 0.434886 0.035124
C 2.722036 1.459738 -0.876958
C 0.240101 -2.258648 -2.997295
O -2.438198 -2.241574 1.091921
C 2.466081 2.838969 -0.328009
O -5.904331 -2.389110 1.150744
C 0.071589 2.038793 -0.546075
C 3.014249 -1.194213 1.780457
C 0.525198 0.844019 0.326900
C 4.183890 1.114064 -1.136345
C 3.402159 3.671141 0.117827
C -1.416477 2.393805 -0.385112
C -2.325014 1.178946 -0.559627
C -3.790148 1.535139 -0.312990
C -4.716578 0.333959 -0.506604
C -4.281646 -0.810284 0.442815
C -5.167324 -2.049519 0.242189
C -2.806490 -1.187386 0.203837
C -0.432905 -0.346401 0.127585
C -1.891925 0.029207 0.357764
C 4.951259 -3.015894 -0.945483
C 3.904741 -2.582946 -0.135334
C 4.146212 -1.735187 0.946450
C -6.183754 0.714127 -0.317012
C -5.092276 -2.808057 -1.047745
C 6.507982 -1.772758 0.402672

C 5.461011 -1.342098 1.208705
C 6.255027 -2.608595 -0.682521
H 0.760592 3.659483 0.692986
H 1.920166 0.976590 3.320848
H 1.208810 4.150699 -2.027841
H 3.601767 0.851456 1.469017
H -1.078993 3.989883 -1.450429
H -5.128157 -0.936024 2.170961
H 2.035345 -0.519160 -0.482170
H 2.213068 1.394174 -1.843892
H 1.044984 -1.654752 -3.421828
H -0.690702 -1.952133 -3.473069
H 0.438751 -3.309072 -3.190803
H -2.690844 -1.947796 1.979431
H 0.194359 1.733600 -1.588664
H 3.284633 -1.229248 2.838821
H 2.119086 -1.804454 1.650312
H 4.796886 1.172955 -0.237811
H 4.607838 1.796007 -1.874911
H 4.266930 0.097049 -1.520786
H 4.455842 3.431459 0.069072
H 3.138451 4.635314 0.536496
H -1.575010 2.813070 0.613011
H -2.219769 0.850325 -1.599155
H -4.092393 2.329516 -0.995452
H -3.906816 1.919346 0.704674
H -4.579255 -0.027777 -1.530022
H -2.702814 -1.592866 -0.802266
H -0.147758 -1.175262 0.769889
H -1.968192 0.367441 1.392308
H 4.746171 -3.671538 -1.782908
H 2.891772 -2.903206 -0.343912
H -6.443446 1.541126 -0.979443
H -6.369903 1.031894 0.710127
H -6.856714 -0.115621 -0.542725
H -4.215941 -3.461264 -1.009613
H -4.971527 -2.150073 -1.907053
H -5.980845 -3.424133 -1.165864
H 7.520908 -1.456954 0.619895
H 5.664964 -0.688474 2.049334
H 7.069040 -2.943683 -1.313081
78
13-c3.out delta G = 0.8942 kcal/mol
conformer 6
O 0.139855 -0.172328 -1.735969
C -0.276147 -1.211183 -2.470806
C 2.543635 2.710405 0.157924
O 0.663666 0.841013 2.573015
C 1.454202 0.413427 1.738534
N 2.614105 -0.197739 2.025394
O -0.855219 -2.177406 -2.015910
O 2.543455 4.105431 -0.229893
C 3.382084 -0.717752 0.910824
O -0.155665 4.000884 -0.347064
O -3.745961 0.151389 1.771270
C 2.615674 -0.190391 -0.328763
C 3.485848 0.747153 -1.188983
C 0.084226 -1.033903 -3.914823
O -2.492373 -1.655446 0.164994
C 3.773615 1.997546 -0.394652
O -5.109111 -0.317906 -1.501330
C 1.246385 1.997863 -0.250021
C 3.545032 -2.250050 0.968458
C 1.324317 0.512805 0.200822
C 4.709061 0.036299 -1.757793
C 4.989294 2.479734 -0.152505
C -0.031902 2.696853 0.239973
C -1.292469 1.925983 -0.154812
C -2.539101 2.550195 0.472997
C -3.827412 1.843560 0.051848
C -3.752247 0.333578 0.356202
C -4.962122 -0.374969 -0.295534
C -2.482877 -0.285316 -0.248149
C 0.058861 -0.220150 -0.275411
C -1.220715 0.441403 0.220504
C 0.697298 -4.344668 -0.440052
C 1.902305 -3.659897 -0.325014
C 2.256430 -3.022630 0.866108
C -5.056703 2.494941 0.686373
C -5.919916 -1.109718 0.595538
C 0.159699 -3.768448 1.830320
C 1.370527 -3.094724 1.945647
C -0.181973 -4.394675 0.635209
H 2.597709 2.742663 1.248692
H 2.886598 -0.342399 2.986766
H 2.898710 4.165494 -1.126573
H 4.382502 -0.284049 0.946341
H 0.729407 4.400659 -0.304443

H -3.396812 -0.734239 1.942387
H 2.329162 -1.033378 -0.952475
H 2.863611 1.035700 -2.041669
H 1.162351 -1.166818 -4.027010
H -0.160921 -0.025806 -4.247227
H -0.436038 -1.770970 -4.519830
H -1.889088 -2.133699 -0.429744
H 1.188786 1.997390 -1.340654
H 4.213845 -2.536719 0.154923
H 4.059685 -2.497638 1.900720
H 5.404910 -0.282693 -0.980113
H 5.253612 0.687439 -2.442616
H 4.397061 -0.849562 -2.313514
H 5.881558 2.012594 -0.546056
H 5.127498 3.374821 0.441915
H 0.004642 2.789796 1.328894
H -1.371979 2.002914 -1.245367
H -2.604467 3.601023 0.189240
H -2.445141 2.514947 1.562643
H -3.918360 1.917550 -1.035086
H -2.577640 -0.222379 -1.334381
H 0.099860 -1.263908 0.028850
H -1.184792 0.364451 1.307329
H 0.438568 -4.822903 -1.375476
H 2.578763 -3.619974 -1.170719
H -5.986195 2.049234 0.325950
H -5.083485 3.557831 0.440579
H -5.031087 2.397673 1.772336
H -5.398222 -1.933010 1.089634
H -6.748258 -1.498087 0.007028
H -6.283586 -0.452468 1.386598
H -0.516778 -3.805603 2.675000
H 1.629376 -2.623603 2.884651
H -1.126280 -4.916235 0.543881
78
13-c4.out delta G = 1.0015 kcal/mol
conformer 7
O 0.196273 0.141224 1.715145
C -0.213956 1.169259 2.469007
C 2.541560 -2.798251 -0.162055
O 0.659802 -0.895449 -2.606372
C 1.440943 -0.421392 -1.786607
N 2.544339 0.275503 -2.093007
O -0.810021 2.134708 2.035927
O 2.633504 -4.003627 0.615316
C 3.342053 0.773243 -0.989480
O -0.031845 -4.031557 0.266666
O -3.749522 -0.149323 -1.728430
C 2.647457 0.169976 0.258337
C 3.582412 -0.802550 1.014966
C 0.176255 0.979606 3.903551
O -2.463575 1.650554 -0.136891
C 3.797266 -1.993490 0.119664
O -5.059931 0.320001 1.566083
C 1.267779 -2.015864 0.203897
C 3.476432 2.307706 -0.992329
C 1.346612 -0.534136 -0.248468
C 4.843126 -0.116111 1.528344
C 4.954661 -2.331853 -0.438284
C -0.015242 -2.691107 -0.288771
C -1.273134 -1.937983 0.151068
C -2.535421 -2.553720 -0.453608
C -3.812849 -1.840664 -0.008961
C -3.735245 -0.331679 -0.313841
C -4.931908 0.380876 0.358494
C -2.454335 0.280331 0.272425
C 0.089700 0.201672 0.257436
C -1.202959 -0.450414 -0.218980
C 0.059644 3.833549 -1.704287
C 1.262053 3.155997 -1.872243
C 2.186241 3.066070 -0.826441
C -5.055287 -2.487006 -0.622517
C -5.899280 1.123304 -0.515285
C 0.681803 4.376920 0.552415
C 1.877981 3.687367 0.385406
C -0.234773 4.446748 -0.489960
H 2.510681 -3.059518 -1.227355
H 2.793205 0.435278 -3.058374
H 1.738211 -4.386570 0.595827
H 4.348657 0.365044 -1.086304
H -0.547282 -4.602182 -0.313536
H -3.404648 0.736928 -1.905135
H 2.379315 0.971873 0.942207
H 3.024729 -1.142536 1.892493
H -0.054896 -0.033692 4.230118
H -0.337254 1.706585 4.526259
H 1.255439 1.119186 3.995734
H -1.853137 2.125167 0.453520

H 1.213442 -2.003182 1.294645
H 4.165284 2.572979 -0.188248
H 3.961806 2.597560 -1.928322
H 4.575387 0.715898 2.182269
H 5.465578 0.279624 0.724659
H 5.452208 -0.815700 2.102493
H 5.874723 -1.802763 -0.227220
H 5.019632 -3.170042 -1.122340
H 0.008005 -2.762202 -1.376164
H -1.325456 -2.023048 1.242353
H -2.617995 -3.603344 -0.163520
H -2.463027 -2.523034 -1.544989
H -3.884124 -1.915395 1.079203
H -2.532269 0.213965 1.359575
H 0.128114 1.247509 -0.039591
H -1.186993 -0.370423 -1.306417
H -0.646836 3.884926 -2.523272
H 1.484979 2.696686 -2.826142
H -5.082733 -3.549894 -0.376852
H -5.048504 -2.388717 -1.708596
H -5.975885 -2.037413 -0.245023
H -6.276509 0.471586 -1.304584
H -5.381768 1.947676 -1.011990
H -6.717763 1.511498 0.086907
H 0.459412 4.844081 1.502773
H 2.584521 3.633218 1.205344
H -1.171617 4.973192 -0.358254
78
13-c7.out delta G = 1.2061 kcal/mol
conformer 8
O 0.150803 -0.168984 -1.734605
C -0.284045 -1.209273 -2.456815
C 2.528313 2.788288 0.063695
O 0.728823 0.961605 2.551515
C 1.482962 0.463596 1.721941
N 2.587460 -0.239307 2.013568
O -0.866467 -2.166516 -1.988394
O 2.508709 4.003001 -0.729923
C 3.360272 -0.754365 0.900579
O -0.168896 4.009308 -0.450773
O -3.695372 0.180911 1.821499
C 2.641767 -0.164896 -0.340607
C 3.564398 0.799715 -1.124790
C 0.059078 -1.044202 -3.906524
O -2.450782 -1.650735 0.232433
C 3.787074 1.995093 -0.238538
O -5.102139 -0.372298 -1.419589
C 1.254274 2.018425 -0.296379
C 3.494432 -2.288419 0.920813
C 1.350338 0.546944 0.184352
C 4.820077 0.113478 -1.649826
C 4.947676 2.341123 0.310226
C -0.029190 2.722682 0.173542
C -1.287627 1.931337 -0.182990
C -2.529403 2.560599 0.449519
C -3.818047 1.833804 0.065939
C -3.724484 0.331775 0.402960
C -4.937770 -0.400721 -0.215025
C -2.459083 -0.289319 -0.207401
C 0.083572 -0.201667 -0.273364
C -1.196157 0.457486 0.226890
C 0.671634 -4.388363 -0.527590
C 1.868041 -3.690924 -0.399613
C 2.202218 -3.050712 0.795330
C -5.042807 2.489197 0.705024
C -5.877738 -1.121939 0.705615
C 0.101026 -3.814285 1.735147
C 1.303626 -3.128649 1.864104
C -0.219074 -4.447311 0.537611
H 2.510401 3.064932 1.122799
H 2.859651 -0.382402 2.975255
H 3.278162 4.533811 -0.489541
H 4.369784 -0.347886 0.971408
H 0.724885 4.375407 -0.556319
H -3.339326 -0.699106 2.006746
H 2.360677 -0.972858 -1.011914
H 2.993251 1.130740 -1.996626
H -0.461203 -1.792400 -4.497648
H 1.137103 -1.166557 -4.030195
H -0.200677 -0.042111 -4.246318
H -1.857513 -2.135913 -0.366470
H 1.202647 1.984657 -1.386535
H 4.165307 -2.564348 0.105178
H 4.001262 -2.565083 1.849378
H 4.545348 -0.724886 -2.292403
H 5.455524 -0.273240 -0.852095
H 5.417851 0.810077 -2.239330
H 5.870722 1.824839 0.082615

H 5.015460 3.168990 1.007622
H 0.016382 2.850274 1.258301
H -1.384732 1.980707 -1.273469
H -2.608370 3.603868 0.141855
H -2.417845 2.552143 1.538102
H -3.926935 1.881790 -1.020810
H -2.570919 -0.248535 -1.292817
H 0.134499 -1.241583 0.042085
H -1.145911 0.405563 1.314850
H 0.428735 -4.870583 -1.465322
H 2.554573 -3.645908 -1.236927
H -4.998743 2.418234 1.792428
H -5.973897 2.026800 0.370709
H -5.083405 3.545632 0.434496
H -5.344311 -1.932897 1.207536
H -6.712840 -1.526254 0.137770
H -6.232907 -0.451013 1.489015
H -0.585469 -3.856566 2.571456
H 1.547093 -2.653964 2.805381
H -1.155995 -4.980322 0.436380
78
13-c14.out delta G = 1.2418 kcal/mol
conformer 9
O -0.348466 0.253983 -2.043543
C -0.810341 -0.670936 -2.896262
C 2.391539 2.459441 0.440645
O 0.365951 0.423949 2.374455
C 1.026360 -0.000648 1.432433
N 1.952918 -0.967514 1.509011
O -1.368363 -1.693522 -2.544563
O 2.619419 3.873365 0.216160
C 2.650750 -1.307767 0.282122
O -0.088850 4.150158 0.097124
O -4.205627 0.504199 1.447944
C 2.121968 -0.269779 -0.738338
C 3.218325 0.708333 -1.218883
C -0.540867 -0.287066 -4.318577
O -3.117043 -1.176181 -0.401640
C 3.559498 1.630855 -0.077228
O -5.484746 0.723200 -1.884418
C 1.052109 2.005130 -0.161661
C 2.401288 -2.761076 -0.150553
C 0.937622 0.467545 -0.038357
C 4.398194 -0.007410 -1.867301
C 4.758462 1.735618 0.487540
C -0.154709 2.754414 0.426730
C -1.483928 2.237110 -0.121860
C -2.669388 2.903511 0.576877
C -4.014942 2.439590 0.019620
C -4.135920 0.902777 0.080493
C -5.398016 0.465985 -0.699154
C -2.929283 0.231404 -0.592204
C -0.409773 -0.006825 -0.608048
C -1.604709 0.714001 0.002016
C 4.660035 -4.963721 1.972724
C 4.217209 -4.090974 0.985192
C 2.865044 -3.753185 0.882775
C -5.182377 3.133390 0.722275
C -6.478266 -0.262690 0.044849
C 2.406046 -5.183011 2.785810
C 1.966379 -4.309646 1.794663
C 3.754530 -5.512176 2.877939
H 2.334861 2.375063 1.527258
H 2.149502 -1.425094 2.387245
H 2.990967 3.984083 -0.669056
H 3.720279 -1.174382 0.448350
H 0.841621 4.412750 0.197826
H -3.991644 -0.438291 1.482940
H 1.756844 -0.796673 -1.617047
H 2.753132 1.314967 -2.002445
H -1.022060 -0.992244 -4.989873
H 0.536699 -0.287817 -4.492842
H -0.904527 0.723136 -4.506952
H -2.565890 -1.634449 -1.058181
H 1.061875 2.239171 -1.229669
H 1.335537 -2.893558 -0.348758
H 2.931533 -2.919737 -1.092246
H 4.046124 -0.642934 -2.681580
H 4.946472 -0.636659 -1.165398
H 5.102273 0.714318 -2.283369
H 5.616137 1.185123 0.125371
H 4.922307 2.396501 1.330517
H -0.149593 2.635824 1.513949
H -1.509473 2.502199 -1.185074
H -2.596875 3.985797 0.466791
H -2.624335 2.684281 1.647918
H -4.049927 2.693224 -1.043293
H -2.978234 0.473705 -1.656267

H -0.502920 -1.082150 -0.469924
H -1.604860 0.451839 1.060785
H 5.710746 -5.218680 2.034746
H 4.926738 -3.669696 0.282164
H -6.142992 2.866097 0.277124
H -5.072817 4.216314 0.644548
H -5.211740 2.869498 1.780042
H -7.332865 -0.422858 -0.608788
H -6.771758 0.293440 0.935865
H -6.093392 -1.225673 0.390129
H 1.694620 -5.607643 3.483205
H 0.914464 -4.058627 1.725757
H 4.097791 -6.193368 3.646320
78
13-c10.out delta G = 1.2770 kcal/mol
conformer 10
O -0.411781 -0.474545 -1.303878
C -0.904545 -1.664006 -1.672421
C 2.435558 2.517367 -0.074092
O 0.532499 1.535352 2.609957
C 1.106712 0.704492 1.914728
N 1.971797 -0.219473 2.360625
O -1.451801 -2.433573 -0.903970
O 2.660066 3.482293 -1.134513
C 2.558900 -1.097674 1.363812
O -0.025505 3.944123 -1.020609
O -4.119011 1.314915 1.886535
C 2.106256 -0.471957 0.021066
C 3.253476 0.225719 -0.755342
C -0.669952 -1.931976 -3.126495
O -3.118026 -1.017797 0.885002
C 3.566516 1.508722 -0.036850
O -5.529581 0.133951 -1.180682
C 1.081152 1.863025 -0.371847
C 2.102982 -2.556642 1.564576
C 0.960143 0.524069 0.386103
C 4.437559 -0.690807 -1.039196
C 4.689570 1.774570 0.622894
C -0.097213 2.819221 -0.129686
C -1.450854 2.152229 -0.365954
C -2.601113 3.080951 0.023667
C -3.973116 2.457649 -0.233663
C -4.102006 1.094774 0.477608
C -5.396882 0.397993 -0.001230
C -2.928587 0.173673 0.112091
C -0.414953 -0.109820 0.110713
C -1.577519 0.830561 0.399419
C 2.533381 -4.767876 -1.502370
C 1.954143 -3.965643 -0.523345
C 2.708869 -3.488491 0.549266
C -5.104425 3.412384 0.149190
C -6.450519 0.073604 1.016439
C 4.638450 -4.650441 -0.343550
C 4.056086 -3.847728 0.630290
C 3.879404 -5.108873 -1.417654
H 2.386695 3.052334 0.878884
H 2.190801 -0.288796 3.343869
H 3.476043 3.959100 -0.937154
H 3.643584 -1.058957 1.459396
H 0.915258 4.144379 -1.157418
H -3.914464 0.470748 2.311678
H 1.726651 -1.256722 -0.625883
H 2.828680 0.496095 -1.726989
H 0.386864 -2.165875 -3.272950
H -0.899149 -1.047526 -3.719290
H -1.272827 -2.776117 -3.448727
H -2.600760 -1.723940 0.463014
H 1.067207 1.622408 -1.437889
H 2.387005 -2.864116 2.574094
H 1.013804 -2.599621 1.510838
H 4.973250 -0.984267 -0.136961
H 5.148736 -0.190929 -1.698466
H 4.098596 -1.604594 -1.528918
H 5.530148 1.093454 0.629833
H 4.807133 2.698266 1.178608
H -0.054268 3.172576 0.904488
H -1.516963 1.940395 -1.438993
H -2.526441 4.012141 -0.538774
H -2.510171 3.336120 1.083922
H -4.052863 2.237642 -1.301520
H -3.019169 -0.054456 -0.952301
H -0.514861 -1.025739 0.688216
H -1.535406 1.043718 1.468664
H 1.932094 -5.126281 -2.328665
H 0.902458 -3.714509 -0.588541
H -6.085191 3.001920 -0.099690
H -4.994435 4.354667 -0.390073
H -5.087543 3.628406 1.218013

H -7.326553 -0.339018 0.520907
H -6.714857 0.962499 1.590618
H -6.054266 -0.653036 1.730281
H 5.684919 -4.918040 -0.265783
H 4.655091 -3.491261 1.460394
H 4.332845 -5.732158 -2.178044
78
13-c9.out delta G = 1.3598 kcal/mol
conformer 11
O -0.363133 0.246379 -2.038517
C -0.839198 -0.692626 -2.868832
C 2.429027 2.532210 0.313213
O 0.467417 0.520077 2.359463
C 1.085208 0.049170 1.409905
N 1.968286 -0.957572 1.477381
O -1.383492 -1.712799 -2.490392
O 2.719977 3.842786 -0.199032
C 2.645531 -1.316913 0.243320
O 0.004753 4.187923 0.111830
O -4.134605 0.519387 1.542119
C 2.141022 -0.262124 -0.772714
C 3.256764 0.704729 -1.238945
C -0.604393 -0.325858 -4.301527
O -3.083816 -1.173657 -0.315081
C 3.574904 1.620678 -0.087820
O -5.496839 0.705784 -1.759316
C 1.079196 2.020787 -0.224996
C 2.352959 -2.761898 -0.189451
C 0.970971 0.489317 -0.065898
C 4.441534 -0.022565 -1.865173
C 4.723663 1.641407 0.579305
C -0.114327 2.770362 0.383912
C -1.462964 2.250004 -0.114554
C -2.631454 2.916286 0.612927
C -3.988382 2.441699 0.092932
C -4.101170 0.905056 0.170173
C -5.380434 0.456845 -0.574986
C -2.909128 0.231506 -0.526542
C -0.387947 0.002751 -0.599635
C -1.571621 0.726381 0.028760
C 2.329575 -5.196753 2.736276
C 1.901509 -4.305471 1.756083
C 2.803017 -3.769676 0.834718
C -5.140153 3.136746 0.819731
C -6.437556 -0.272187 0.200694
C 4.577636 -5.036625 1.893617
C 4.146373 -4.145987 0.916989
C 3.669275 -5.564724 2.808001
H 2.376124 2.580944 1.407322
H 2.179297 -1.402313 2.358768
H 1.897854 4.349855 -0.082567
H 3.719155 -1.214124 0.402643
H -0.281577 4.346211 -0.798183
H -3.921968 -0.423305 1.580803
H 1.769081 -0.774515 -1.657229
H 2.812316 1.318701 -2.028507
H -0.974515 0.681312 -4.493613
H -1.099737 -1.040171 -4.952519
H 0.468757 -0.326523 -4.501193
H -2.547328 -1.637656 -0.979908
H 1.061685 2.224161 -1.299291
H 1.282266 -2.867754 -0.376117
H 2.868194 -2.931263 -1.137610
H 4.101998 -0.647752 -2.693069
H 4.966738 -0.664889 -1.157647
H 5.163424 0.695261 -2.256850
H 5.568699 1.028799 0.293309
H 4.859032 2.290981 1.436285
H -0.073935 2.692787 1.468785
H -1.528729 2.493772 -1.183593
H -2.571229 3.999444 0.498914
H -2.555482 2.702254 1.682853
H -4.051346 2.686525 -0.970725
H -2.985769 0.461904 -1.591665
H -0.476209 -1.070473 -0.445224
H -1.546256 0.480927 1.091168
H 1.615878 -5.605302 3.440926
H 0.856255 -4.024615 1.702869
H -5.141708 2.880859 1.879778
H -6.110152 2.861656 0.400767
H -5.037432 4.219411 0.730626
H -6.712587 0.289501 1.094147
H -6.038341 -1.230007 0.544096
H -7.306825 -0.442750 -0.430520
H 5.621416 -5.321691 1.939656
H 4.858013 -3.741298 0.206398
H 4.003388 -6.260209 3.567561
78

13-c1.out delta G = 1.3661 kcal/mol
conformer 12
O 0.152537 -0.166608 -1.745128
C -0.271614 -1.207761 -2.473443
C 2.537761 2.788324 0.062536
O 0.708428 0.948815 2.541413
C 1.476309 0.460934 1.717441
N 2.593994 -0.216582 2.015728
O -0.850591 -2.169736 -2.010644
O 2.602021 4.035361 -0.647406
C 3.369075 -0.737994 0.906546
O -0.100165 4.051028 -0.321953
O -3.699153 0.149515 1.803204
C 2.641329 -0.171037 -0.339563
C 3.550330 0.786186 -1.144334
C 0.079788 -1.037090 -3.920281
O -2.453741 -1.658388 0.194640
C 3.790638 2.002876 -0.289869
O -5.102827 -0.349768 -1.448048
C 1.259641 2.011963 -0.295945
C 3.509823 -2.271600 0.944937
C 1.348812 0.538132 0.179816
C 4.797954 0.090182 -1.676621
C 4.974396 2.383029 0.178912
C -0.020596 2.701491 0.198948
C -1.288221 1.928793 -0.169732
C -2.529599 2.547466 0.474645
C -3.817650 1.828349 0.074576
C -3.726653 0.321182 0.387550
C -4.939989 -0.400158 -0.244126
C -2.461143 -0.293246 -0.230107
C 0.083447 -0.207849 -0.284479
C -1.197973 0.446734 0.216612
C 0.113530 -3.785252 1.770610
C 1.319871 -3.105537 1.896094
C 2.217736 -3.035478 0.826149
C -5.042236 2.475228 0.722530
C -5.879410 -1.138049 0.663328
C 0.679116 -4.370324 -0.490621
C 1.879425 -3.678941 -0.365974
C -0.211336 -4.420106 0.575314
H 2.535490 2.994356 1.140436
H 2.865974 -0.350580 2.978781
H 1.713206 4.424123 -0.566196
H 4.375650 -0.322830 0.968872
H -0.405113 4.004237 -1.238314
H -3.346555 -0.734383 1.976469
H 2.361780 -0.992112 -0.995642
H 2.966749 1.101023 -2.014596
H 1.156497 -1.175666 -4.039110
H -0.162608 -0.029075 -4.254856
H -0.447991 -1.774005 -4.518920
H -1.856013 -2.137912 -0.404747
H 1.198309 1.982791 -1.386403
H 4.181592 -2.555070 0.132663
H 4.016238 -2.536597 1.877062
H 5.446003 -0.279379 -0.880612
H 5.386387 0.774400 -2.289437
H 4.513923 -0.761289 -2.297716
H 5.890528 1.868572 -0.079419
H 5.064981 3.242340 0.833050
H 0.029199 2.826313 1.278626
H -1.391289 1.985635 -1.262337
H -2.612347 3.597679 0.191667
H -2.417379 2.516090 1.562254
H -3.924115 1.895312 -1.011378
H -2.570795 -0.239323 -1.315375
H 0.132100 -1.249294 0.026002
H -1.152333 0.384022 1.304028
H -0.572381 -3.821044 2.607699
H 1.566575 -2.628753 2.835479
H -5.000207 2.385615 1.808557
H -5.972971 2.019687 0.378384
H -5.080932 3.536033 0.469617
H -6.235533 -0.480951 1.457943
H -5.344967 -1.956664 1.151600
H -6.713806 -1.533667 0.088424
H 0.433088 -4.854774 -1.426364
H 2.565360 -3.640424 -1.204070
H -1.151466 -4.947893 0.476576
78
13-c5.out delta G = 1.3749 kcal/mol
conformer 13
O -0.416128 -0.479625 -1.298733
C -0.898578 -1.673637 -1.667507
C 2.435267 2.534155 -0.113427
O 0.516239 1.527363 2.615974
C 1.101975 0.705128 1.918906

N 1.980866 -0.205774 2.362875
O -1.442987 -2.445820 -0.899836
O 2.739249 3.463294 -1.167433
C 2.571216 -1.081590 1.365725
O 0.075839 3.954813 -0.941946
O -4.134904 1.292305 1.881347
C 2.100861 -0.468684 0.023166
C 3.233984 0.228129 -0.772224
C -0.655996 -1.942186 -3.120011
O -3.124931 -1.034124 0.875356
C 3.558727 1.517987 -0.069802
O -5.532407 0.122390 -1.196361
C 1.070774 1.862686 -0.365889
C 2.131500 -2.544149 1.576944
C 0.953488 0.523007 0.391002
C 4.413457 -0.690821 -1.068429
C 4.690442 1.779020 0.575193
C -0.102683 2.802694 -0.080151
C -1.459032 2.149034 -0.350181
C -2.616920 3.069768 0.036187
C -3.985045 2.441664 -0.233282
C -4.112861 1.076382 0.472267
C -5.404399 0.378107 -0.014736
C -2.936065 0.159694 0.107125
C -0.420613 -0.115577 0.115756
C -1.588230 0.819158 0.403835
C 2.561578 -4.760935 -1.485919
C 1.981730 -3.960150 -0.506110
C 2.738404 -3.475313 0.561687
C -5.121684 3.391317 0.145987
C -6.458603 0.042385 0.998280
C 4.671233 -4.627057 -0.337250
C 4.088187 -3.825721 0.637295
C 3.910198 -5.092938 -1.406716
H 2.397923 3.073173 0.840397
H 2.203946 -0.270984 3.345508
H 1.916937 3.966173 -1.302262
H 3.656069 -1.029481 1.452705
H -0.365541 4.713443 -0.544914
H -3.931170 0.447262 2.305234
H 1.718739 -1.262005 -0.611775
H 2.794191 0.491489 -1.739292
H -0.882287 -1.058460 -3.714906
H -1.256140 -2.787253 -3.444990
H 0.401830 -2.175719 -3.259930
H -2.601772 -1.737501 0.455681
H 1.026279 1.624497 -1.431805
H 2.425291 -2.844479 2.585816
H 1.042357 -2.596915 1.530600
H 4.960455 -0.981858 -0.172225
H 5.116717 -0.193669 -1.738177
H 4.067645 -1.606230 -1.550555
H 5.520957 1.085329 0.588339
H 4.825243 2.713026 1.108419
H -0.057011 3.128246 0.959794
H -1.510637 1.947668 -1.425960
H -2.557828 4.003668 -0.526893
H -2.535765 3.324743 1.097223
H -4.055878 2.225983 -1.302483
H -3.021487 -0.064440 -0.958541
H -0.518470 -1.031738 0.693299
H -1.552322 1.025929 1.474656
H 1.958827 -5.125143 -2.308603
H 0.928173 -3.715808 -0.567151
H -6.098668 2.977936 -0.112022
H -5.011870 4.336373 -0.388374
H -5.113205 3.602111 1.215874
H -6.060207 -0.685391 1.709746
H -7.330911 -0.372678 0.498295
H -6.729621 0.927108 1.575866
H 5.719816 -4.887513 -0.263826
H 4.688634 -3.462896 1.463577
H 4.364166 -5.714920 -2.167860
78
13-c6.out delta G = 1.7759 kcal/mol
conformer 14
O -0.109888 -0.744246 -1.225750
C -0.011333 -2.060000 -1.456174
C 1.114204 3.291857 -0.290492
O -0.328872 1.819473 2.452294
C 0.596412 1.295814 1.841596
N 1.782425 0.956154 2.370672
O -0.210856 -2.909075 -0.606840
O 0.866072 4.370644 -1.226905
C 2.733170 0.313287 1.480879
O -1.686179 3.420609 -1.409441
O -4.380666 -0.439880 1.737351
C 2.088925 0.484956 0.083274

C 2.833979 1.491367 -0.825586
C 0.401672 -2.344468 -2.866675
O -2.424325 -2.188939 0.994968
C 2.579937 2.882590 -0.307097
O -4.990348 -2.398108 -1.192736
C 0.180386 2.098635 -0.552093
C 2.967304 -1.156838 1.883315
C 0.610908 0.913486 0.342689
C 4.296613 1.125553 -1.050207
C 3.516398 3.718947 0.129760
C -1.305739 2.465272 -0.407545
C -2.225643 1.257863 -0.578880
C -3.681914 1.626718 -0.294520
C -4.636145 0.448688 -0.491283
C -4.200506 -0.761937 0.359807
C -5.043343 -1.992816 -0.047564
C -2.728157 -1.112683 0.099129
C -0.341711 -0.276702 0.139207
C -1.808407 0.089267 0.320251
C 6.225287 -2.146492 0.193267
C 5.318119 -1.568598 1.073516
C 3.952924 -1.847213 0.978907
C -6.086273 0.848782 -0.217441
C -5.904061 -2.644253 0.994491
C 4.423275 -3.311444 -0.894848
C 3.518963 -2.732079 -0.009429
C 5.779361 -3.016220 -0.799031
H 0.865340 3.726260 0.679090
H 1.964624 1.093880 3.354367
H 1.351682 4.183615 -2.041197
H 3.682382 0.845836 1.535728
H -0.949656 4.050721 -1.483074
H -3.851849 -1.066526 2.250088
H 2.112825 -0.469510 -0.432820
H 2.344646 1.414997 -1.802019
H -0.139958 -1.703740 -3.560947
H 0.230807 -3.391973 -3.097943
H 1.466276 -2.122007 -2.969012
H -1.629640 -2.634706 0.657099
H 0.314253 1.780362 -1.589420
H 3.327108 -1.173960 2.915014
H 2.010894 -1.682533 1.867341
H 4.376240 0.097416 -1.405162
H 4.895459 1.205361 -0.143468
H 4.739894 1.782354 -1.800031
H 4.568832 3.470948 0.099381
H 3.254678 4.694533 0.522329
H -1.468326 2.893824 0.585699
H -2.140527 0.940344 -1.624338
H -3.988054 2.441379 -0.951361
H -3.762889 1.993247 0.733403
H -4.559135 0.117353 -1.530244
H -2.657985 -1.457505 -0.935020
H -0.065711 -1.082559 0.815414
H -1.914847 0.398855 1.360949
H 7.280258 -1.917903 0.279953
H 5.672308 -0.888733 1.839839
H -6.365951 1.691220 -0.852157
H -6.218400 1.148365 0.822805
H -6.780325 0.032357 -0.427325
H -5.269810 -3.056364 1.783472
H -6.491188 -3.440463 0.542180
H -6.553532 -1.907869 1.469458
H 4.067320 -3.993482 -1.656995
H 2.465993 -2.976053 -0.082383
H 6.484837 -3.464927 -1.486955
78
13-c2.out delta G = 1.7815 kcal/mol
conformer 15
O -0.410077 -0.472132 -1.316230
C -0.893755 -1.664340 -1.690239
C 2.442854 2.525297 -0.095345
O 0.514314 1.537765 2.594119
C 1.102138 0.710121 1.905395
N 1.980455 -0.197840 2.356863
O -1.442225 -2.437376 -0.926537
O 2.751988 3.501156 -1.104272
C 2.571391 -1.080823 1.365841
O 0.042052 3.994864 -0.936620
O -4.120219 1.281525 1.889270
C 2.106620 -0.473368 0.018828
C 3.242864 0.220017 -0.775268
C -0.645546 -1.930027 -3.142219
O -3.118666 -1.031583 0.856771
C 3.571177 1.511498 -0.076705
O -5.530098 0.152748 -1.198001
C 1.083738 1.857963 -0.380341
C 2.126718 -2.541155 1.582567

C 0.959085 0.520203 0.378661
C 4.420459 -0.702881 -1.066805
C 4.712149 1.776566 0.550225
C -0.093479 2.807774 -0.117780
C -1.455331 2.155142 -0.353889
C -2.603051 3.077959 0.057674
C -3.975111 2.459205 -0.210190
C -4.103824 1.084238 0.477476
C -5.399077 0.396358 -0.014101
C -2.930808 0.167824 0.097748
C -0.414850 -0.114553 0.099964
C -1.579360 0.821396 0.393512
C 2.550665 -4.769602 -1.472894
C 1.973082 -3.963255 -0.496316
C 2.730689 -3.478109 0.570785
C -5.105030 3.407831 0.191136
C -6.453724 0.056562 0.997123
C 4.659668 -4.640731 -0.322507
C 4.078929 -3.833847 0.648863
C 3.897819 -5.106945 -1.391224
H 2.393481 3.024871 0.879128
H 2.200733 -0.258160 3.340457
H 1.939122 4.025768 -1.206201
H 3.656122 -1.031283 1.455860
H -0.261661 3.782623 -1.829896
H -3.917552 0.430144 2.300892
H 1.726190 -1.268822 -0.614603
H 2.805868 0.480745 -1.744415
H -1.255783 -2.764792 -3.474952
H 0.409608 -2.179830 -3.274620
H -0.853369 -1.040737 -3.735404
H -2.597840 -1.732690 0.430332
H 1.060786 1.614960 -1.446129
H 2.419153 -2.838774 2.592615
H 1.037428 -2.590522 1.536146
H 5.126371 -0.210211 -1.737048
H 4.072602 -1.618737 -1.546564
H 4.964981 -0.992618 -0.168654
H 5.545442 1.086200 0.549366
H 4.852196 2.710911 1.081451
H -0.039682 3.170358 0.907237
H -1.536008 1.948363 -1.429663
H -2.533117 4.022898 -0.482668
H -2.508111 3.309940 1.122440
H -4.055781 2.258689 -1.281807
H -3.021524 -0.047193 -0.969455
H -0.515521 -1.032749 0.673722
H -1.539717 1.025871 1.464369
H 1.947230 -5.134035 -2.294983
H 0.920573 -3.714851 -0.559695
H -5.087476 3.603295 1.263792
H -6.085844 3.002542 -0.065285
H -4.994670 4.359924 -0.330415
H -6.718270 0.936938 1.584247
H -6.058756 -0.680904 1.700486
H -7.329367 -0.347956 0.494328
H 5.707074 -4.905317 -0.247115
H 4.680039 -3.470911 1.474609
H 4.349952 -5.733348 -2.149832

78

13-c18.out delta G = 3.4620 kcal/mol

conformer 16

O -0.007477 0.005149 -1.861657
C 0.143162 -0.997496 -2.746742
C 2.540905 2.699914 0.046331
O 0.656048 0.787970 2.476522
C 1.447387 0.368954 1.637013
N 2.630952 -0.196101 1.918655
O 0.361240 -2.146868 -2.445582
O 2.592379 3.994343 -0.574084
C 3.349635 -0.790668 0.809484
O -0.107508 4.011254 -0.235332
O -3.586068 0.014828 1.781181
C 2.597194 -0.242035 -0.433009
C 3.486639 0.714360 -1.259522
C 0.011197 -0.475237 -4.149699
O -2.440150 -1.724857 -0.119746
C 3.771342 1.926022 -0.408243
O -5.707272 -0.988798 0.727302
C 1.242144 1.963160 -0.314166
C 3.426056 -2.328007 0.921325
C 1.311669 0.466093 0.099238
C 4.708086 0.009385 -1.838616
C 4.982669 2.313955 -0.023394
C -0.023343 2.643835 0.237738
C -1.308966 1.894229 -0.127439
C -2.535990 2.485497 0.566328
C -3.830226 1.797452 0.124677

C -3.728010 0.273053 0.388697
C -4.990539 -0.452666 -0.099118
C -2.490289 -0.315602 -0.319488
C 0.038725 -0.227294 -0.419821
C -1.221627 0.388130 0.165553
C 0.300124 -4.321299 0.055385
C 1.539061 -3.696850 -0.044736
C 2.097764 -3.029024 1.044287
C -5.055149 2.417188 0.793608
C -5.296368 -0.497698 -1.565172
C 0.141491 -3.628047 2.350979
C 1.384234 -3.014447 2.248502
C -0.406854 -4.283137 1.251013
H 2.585936 2.828503 1.135483
H 2.911733 -0.327349 2.879397
H 1.704601 4.375538 -0.453390
H 4.373187 -0.415908 0.818275
H -0.413587 3.996093 -1.152559
H -4.402627 -0.443970 2.045570
H 2.311859 -1.075252 -1.071263
H 2.878258 1.042173 -2.108359
H -0.954381 0.016272 -4.273701
H 0.106945 -1.292645 -4.858812
H 0.786260 0.269716 -4.336297
H -2.390475 -1.880893 0.833836
H 1.156896 1.983277 -1.402229
H 3.962367 -2.693596 0.043476
H 4.047893 -2.563276 1.789460
H 4.392062 -0.837579 -2.450312
H 5.378297 -0.369299 -1.065613
H 5.282190 0.688212 -2.470603
H 5.882023 1.808471 -0.349248
H 5.113176 3.171754 0.625843
H 0.054209 2.735458 1.318699
H -1.436841 2.007824 -1.211914
H -2.611577 3.551231 0.346885
H -2.423922 2.384418 1.649425
H -3.918471 1.929846 -0.957646
H -2.594042 -0.176337 -1.395577
H 0.077958 -1.298736 -0.244014
H -1.161029 0.252238 1.245024
H -0.118478 -4.821823 -0.808116
H 2.069731 -3.715502 -0.987300
H -5.025596 2.259580 1.872781
H -5.987793 1.994775 0.414120
H -5.079122 3.492089 0.608309
H -4.655442 -1.253683 -2.026996
H -5.088384 0.450817 -2.058793
H -6.335685 -0.780539 -1.715957
H -0.396284 -3.599609 3.290592
H 1.805322 -2.526268 3.117608
H -1.375764 -4.760434 1.328003

**Table S33.** Experimental $^{13}C$ NMR chemical shifts and their deviations ($CDCl_3$) of **5** and **14**.

| No. | Exptl. 5 | Exptl. 14 | Dev. (5 minus 14 |
|---|---|---|---|
| 1 | 177.12 | 175.55 | 1.57 |
| 3 | 60.55 | 59.81 | 0.74 |
| 4 | 49.48 | 48.5 | 0.98 |
| 5 | 125.17 | 125.65 | -0.48 |
| 6 | 134.32 | 133.03 | 1.29 |
| 7 | 66.25 | 71.92 | -5.67 |
| 8 | 43.93 | 43.82 | 0.11 |
| 9 | 51.78 | 50.7 | 1.08 |
| 10 | 43.46 | 43.44 | 0.02 |
| 11 | 17.36 | 17.28 | 0.08 |
| 12 | 14.41 | 13.91 | 0.5 |
| 13 | 66.57 | 72.41 | -5.84 |
| 14 | 37.16 | 39.72 | -2.56 |
| 15 | 30.91 | 31.33 | -0.42 |
| 16 | 35.9 | 35.91 | -0.01 |
| 17 | 83.85 | 83.81 | 0.04 |
| 18 | 214.29 | 211.96 | 2.33 |
| 19 | 72.59 | 73.22 | -0.63 |
| 20 | 38.13 | 40.94 | -2.81 |
| 21 | 71.77 | 71.18 | 0.59 |
| 22 | 24.9 | 24.85 | 0.05 |
| 23 | 14.8 | 14.73 | 0.07 |
| 24 | 137.19 | 137.55 | -0.36 |
| 25 | 129.02 | 129.09 | -0.07 |
| 26 | 128.92 | 128.8 | 0.12 |
| 27 | 127.11 | 126.91 | 0.2 |
| 28 | 128.92 | 128.8 | 0.12 |
| 29 | 129.02 | 129.09 | -0.07 |
| 30 | 171.6 | 171.76 | -0.16 |
| 31 | 21.02 | 21.05 | -0.03 |
